# Abnormal Expression of N6-Methyladenosine RNA Methylation Regulator IGF2BP3 in Colon Cancer Predicts a Poor Prognosis

**DOI:** 10.1155/2022/5883101

**Published:** 2022-05-30

**Authors:** Tao Wu, Xuan Zhang, Lu Xing, Dingguo Pan, Ping Liu, Rong Ding, Renfang Yang, Xudong Yang, Yunfeng Li

**Affiliations:** ^1^Department of Colorectal Surgery, Third Affiliated Hospital of Kunming Medical University, Tumor Hospital of Yunnan Province, Kunming, China; ^2^Dermatology, Kunming Children's Hospital, Kunming, China; ^3^Department of Minimally Invasive Intervention, Third Affiliated Hospital of Kunming Medical University, Tumor Hospital of Yunnan Province, Kunming, China

## Abstract

The value of insulin-like growth factor 2 mRNA-binding protein 3 (IGF2BP3), an N6-methyladenosine (m6A) RNA methylation regulatory factor, in the prognosis of colon cancer was still unclear. High levels of IGF2BP3 were expressed in colon adenocarcinoma (COAD) samples and in human colon cancer tissues, which was associated with poorer overall survival (OS). We validated IGF2BP3 as an independent prognostic risk biomarker in COAD patients. Moreover, functional enrichment analysis suggested that differentially expressed genes (DEGs) of groups with high versus low IGF2BP3 expression were related to immune- and cancer-related pathways. Furthermore, the tumor microenvironments of high- versus low-IGF2BP3 expression groups showed significant differences and IGF2BP3 predicted the efficiency of immunotherapy. Finally, protein-protein interaction network analysis suggested that there was a direct or indirect interaction among IGF2BP3, WNT7B, VANGL2, NKD1, AXIN2, RNF43, and CDKN2A. In brief, IGF2BP3 was confirmed as an independent prognostic signature in COAD patients and might be a therapeutic target in this study. Moreover, IGF2BP3 could be used in personalized immunotherapy for COAD.

## 1. Introduction

Colon cancer is one of the most common digestive cancers in the world. It ranks the third in the incidence rate of cancer worldwide. According to global cancer statistics, in 2020, there were 1.9316 million new cases of colorectal cancer and 935,200 deaths [1]. If left untreated, the prognosis of colon cancer is poor and the median survival is less than 1 year and the 5-year survival is less than 5% [2]. Although diagnosis and treatment technologies for colon cancer have progressed rapidly in the last few years, survival of colon cancer patients is not ideal. Further research into diagnostic, prognostic, and molecular markers related to the clinical characteristics of colon cancer is needed.

Epigenetic modifications, such as posttranscriptional RNA and DNA methylation, are considered key regulatory mechanisms in many biological processes [3]. M6A RNA methylation is an important content in epigenetic studies and is common in eukaryotic cells. M6A RNA modifications regulate RNA biogenesis, degradation, transport, and cellular localization [4, 5]. The most widely studied m6A RNA methylation regulatory factors are IGF2BP3 and the YTHDF proteins [6–9]. Different from YTHDFs, IGF2BPs enable more rapid recognition of modified m6A mRNAs, resulting in improved stability and translation of modified m6A mRNAs [8]. Gene IGF2BP3, or IMP3 as it has been known in other studies, is upregulated in many tumors [10].

IGF2BP3 overexpression regulates IGF2/IGF1 receptor signaling (IGF1R) through mitogen-activated protein kinase (MAPK) and phosphatidylinositol-3-kinase (PI3K), thereby promoting proliferation, invasion, and transformation of cells [11]. However, silencing IGF2BP3 expression suppresses breast cancer cell proliferation by reducing tripartite motif-containing 25 (TRIM25) expression [12]. Furthermore, the IGF2BP3-activated Janus kinase 2/signal transduction and transcription activator (JAK/STAT) pathway can significantly promote bladder cancer cell proliferation and occurrence [13]. Some studies have indicated that IGF2BP3 can be used as a prognostic signature in colon cancer [14, 15] but its mechanism has not been systematically analyzed.

Immunotherapy is a common cancer treatment that works by stimulating the immune system and increasing its ability to suppress the growth of cancer cells. M6A RNA methylation is believed to affect the efficiency of immunotherapy. For example, the therapeutic efficiency blocking the PD-L1 checkpoint was a significance enhancement by deletion of YTHDF1 [16]. Moreover, knockdown of alpha-ketoglutarate-dependent dioxygenase (FTO) in tumor cells sensitized to interferon gamma (IFN-*γ*) in vitro enhances the PD-1 treatment in murine melanoma [17].

The important value of IGF2BP3 in colon cancer prognosis and treatment was evaluated using a bioinformatics detection system, and we verified findings using clinical tissue specimens. And the function of gene IGF2BP3 in immunotherapy was identified, which may contribute to more effective treatment in patients with colon cancer.

## 2. Materials and Methods

### 2.1. Sample Source

This study included 12 cancerous tissues and 12 matched paracancerous tissues from 12 COAD patients admitted to Kunming Medical University Third Affiliated Hospital. All participants were informed and agreed to take part in the study. This study was authorized by the ethics committee of Kunming Medical University Third Affiliated Hospital.

### 2.2. Analysis of the Expression of IGF2BP3

Firstly, the expression of IGF2BP3 in pancancer was examined based on the Oncomine database (https://www.oncomine.org/resource/login.html) [18]. Using the Tumor Immune Estimation Resource (TIMER) site to further analyze the accumulation of IGF2BP3 expression in different types of tumors, *P* < 0.05 [19].

### 2.3. Data Collection

The fragments per kilobase of exon per million reads mapped (FPKM) and survival data of 393 patients and 34 normal control tissues were obtained from The Cancer Genome Atlas (TCGA) (https://portal.gdc.cancer.gov/) database. Moreover, the GSE 41258 dataset was extracted from the Gene Expression Omnibus (GEO) (https://www.ncbi.nlm.nih.gov/geo/query/acc.cgi), which contains survival information of 155 COAD samples and 54 normal control samples. The clinical information of the samples with survival information in the TCGA and GEO database was shown in Table S1 and Table S2.

### 2.4. Expression Analysis of IGF2BP3 in COAD

The IGF2BP3 expression levels in COAD and normal samples were assessed using the TCGA database and the GSE 41258 dataset by the Wilcoxon test. Moreover, the Human Protein Atlas (HPA) database was used to investigate IGF2BP3 protein expression in COAD and normal samples.

### 2.5. Correlation Analysis between IGF2BP3 Expression and Clinicopathological Features

Associations between the expression of IGF2BP3 and clinicopathological features (age, gender, cancer stage, and pathologic T-M-N-stage) were measured. And the clinicopathological features were provided by TCGA and the GSA 41258 dataset. CA19-9 is only in TCGA, and microsatellite instable/microsatellite stable (MSI\MSS) is only in the GSE 41258 dataset. Statistical analysis was performed by the Wilcoxon test.

### 2.6. Survival Analysis

The relationship between the IGF2BP3 expression and OS of COAD patients was analyzed in the GSE 41258 dataset and TCGA using the “survminer” R package (version 0.4.6) [20]. The prognostic value of IGF2BP3 was evaluated by a combination of K-M survival analysis and Wilcoxon testing.

### 2.7. Independent Prognostic Analysis

The R software package “survival” (version 3.2-7) was applied for conducting univariate and multivariate Cox proportional risk regression analysis for biomarkers, age, sex, tumor stage, and pathological TNM stage, and the ability to make independent prognostic was analyzed by univariate (uni-) and multivariate (mul-) Cox regression analyses.

### 2.8. GSEA Analysis

To examine the biological functions of DEGs (high expression of IGF2BP3 vs low expression of IGF2BP3), the “clusterProfiler” software package (version 3.18.0) was applied for gene set enrichment analysis (GSEA).

### 2.9. Association between IGF2BP3 Expression and the Tumor Microenvironment

Differences in the tumor microenvironment between the high- and low-IGF2BP3 expression groups were analyzed using the ESTIMATE algorithm [21], ABSOLUTE database [22], and single-sample gene set enrichment analysis (ssGSEA) [23]. Firstly, the ESTIMATE algorithm was applied to compare differences in the stromalscore, immunescore, and ESTIMATEscore. Secondly, the tumor purity was calculated by the ABSOLUTE algorithm based on the copy number variation (CNV) of COAD. Lastly, ssGSEA was used to infer the proportion and composition of infiltrating immune cells, based on 24 gene sets, and the activity of immune-related pathways. And the correlation analysis between IGF2BP3 expression and the proportion of the 24 infiltrating immune cell gene sets was conducted. The Wilcoxon test was applied to explore the differences of the tumor microenvironment.

### 2.10. Association between IGF2BP3 Expression and Immune Therapy

Immune checkpoint blockade therapies, especially those targeting CTLA-4 and PD-1, have been proved to be a promising treatment for treating multiple cancers [24]. Therefore, the association between IGF2BP3 expression and immune therapy was investigated by comparing the expression of immune checkpoint molecules in the high- and low-IGF2BP3 expression groups, with a threshold of *P* < 0.05. The Tumor Immune Dysfunction and Exclusion (TIDE) algorithm and chi-squared testing were applied to model and compare the influence of CTLA-4 and PD-1 blockade therapies [25].

### 2.11. Mechanistic Analysis of IGF2BP3 Regulation

We initially investigated the mechanism of IGF2BP3 regulation by screening genes coexpressed with IGF2BP3 in the LinkedOmics database. A false discovery rate (FDR) < 0.05 and |Pearson′s correlation| ≥ 0.3 were applied as cutoff values. Then, we screened DEGs using the “DEseq2” package (version 1.30.0) in R (high-IGF2BP3 expression group vs low-IGF2BP3 expression group). Genes with |log2 (fold change)| > 0.5 and *P* < 0.01 were identified as DEGs. Genes that were coexpressed with IGF2BP3 and DEGs were defined as IGF2BP3-related genes.

The function of IGF2BP3-related genes was analyzed by performing gene ontology (GO) and Kyoto Encyclopedia of Genes and Genomes (KEGG) pathway enrichment analysis using R package “clusterProfiler” (version 3.18.0). We also built a protein-protein interaction (PPI) network to visualize the interactions among IGF2BP3 and IGF2BP3-related genes, and the interactions of these proteins were visualized by Cytoscape (version 3.8.0). We selected a hub IGF2BP3-related gene and explored the interaction network of the biological process for them using the Cytoscape ClueGO plug-in.

### 2.12. Immunohistochemical Staining

A total of 12 COAD tissues and 12 matched paracancer tissues from 12 COAD patients were embedded with paraffin, and cut the sample into 5–7 *μ*m thick slices using a microtome (Leica Co. Ltd., Shanghai, China) and baked at 50°C. Xylene was dewaxed twice for 5 minutes each, followed by gradient dehydration with ethanol for 3 minutes each. Block endogenous tissue peroxidase with methanol containing 0.3% H_2_O_2_. The sections were then incubated with the anti-IGF2BP3 antibody (1:100;57145, Cell Signaling, Technology, Massachusetts, USA) at 4°C overnight. And they were detected using two-step streptavidin biotin peroxidase (SP) coupling and a standard SP kit. Pathological changes were observed and photographed with an optical microscope.

### 2.13. RNA Isolation and Quantification

TRIzol (lot: AKF0722A, cat: 9109, TaKaRa, Dalian, China) was applied for isolating total RNA from 12 tumor tissues and 12 matched paracancer tissues; cDNA was transcribed using a reverse transcription kit (lot: U8219, cat: KR118-02, Tiangen, Beijing, China) and analyzed by quantitative PCR (qPCR) amplification using a dNTP mixture on a 7500 Real-Time PCR System (Thermo Fisher Scientific, Foster City, California). Primers to amplify IGF2BP3 and *β*-actin were designed and then obtained from Sangon Company (Sangon Biotech, Shanghai, China). The internal reference in this study was *β*-actin. Primer sequences of IGF2BP3 were “F:AGTTGTTGTCCCTCGTGACC, R:GTCCACTTTGCAGAGCCTTC.” Primer sequences of *β*-actin were “F:TGACGTGGACATCCGCAAAG, R:.CTGGAAGGTGGACAGCGAGG.” Gene expression rates in colon cancer tissues and corresponding paracellular tissues were calculated using the 2^−Δ*Ct*^ algorithm.

### 2.14. Statistical Analysis

R Studio software was applied for statistical analysis in this study, and Wilcoxon testing was used in the comparative analysis. Chi-squared testing was used to compare the responses to immune therapy. Paired student *t*-test and Mann–Whitney *U* test were used for the normal distribution and non-normal distribution groups, respectively.

## 3. Results and Discussion

### 3.1. IGF2BP3 mRNA Expression Levels in Pancancer

**Figure 1:**
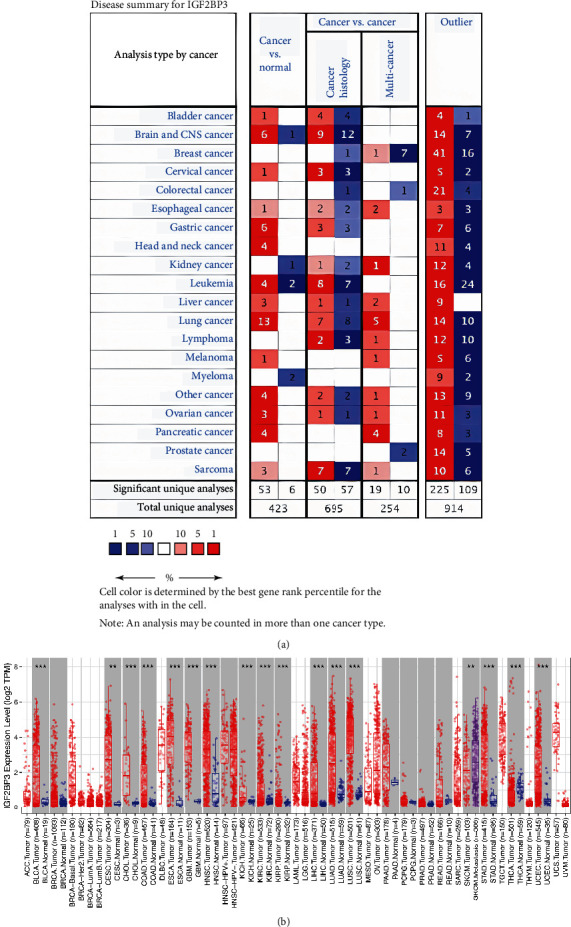
IGF2BP3 expression levels in pancancer. (a) Relative expression of IGF2BP3 in pancancer and normal tissues, based on the Oncomine database; (b) relative expression of IGF2BP3 in pancancer and normal tissues, based on the TIMER database.

We found that IGF2BP3 expression is high in most tumors except kidney and myeloma cancers (Figure 1(a)). We also evaluated the IGF2BP3 expression in human cancers by the TIMER set, and the analysis explored that most tumor tissues had higher IGF2BP3 expression, except skin cutaneous melanoma (Figure 1(b)). These findings indicate that IGF2BP3 is an important gene in tumors.

### 3.2. The Expression Levels of IGF2BP3 mRNA and Protein in COAD

**Figure 2:**
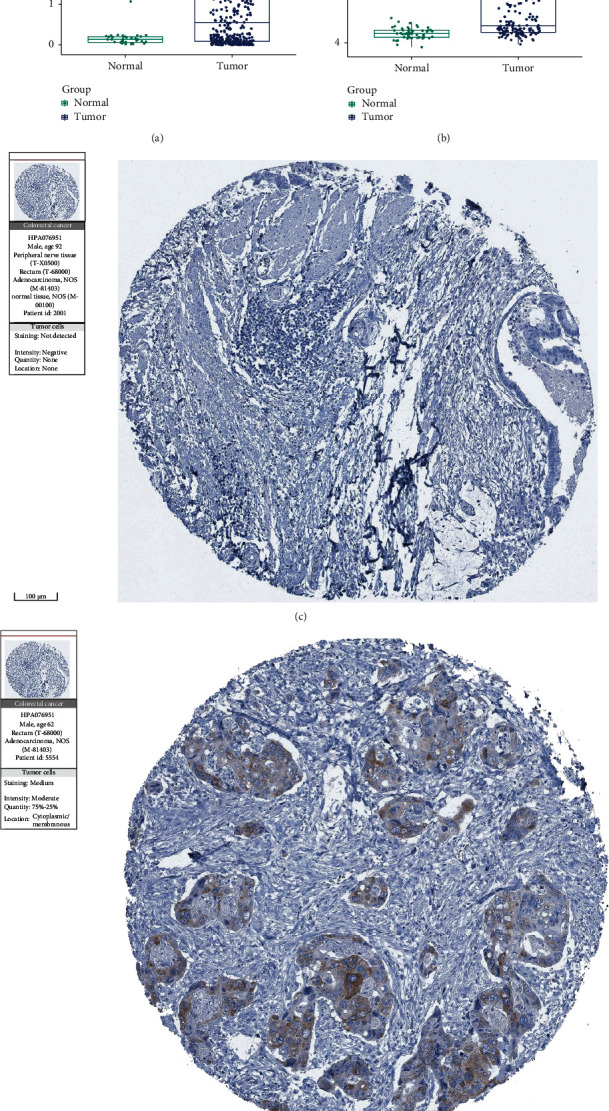
The expression levels of IGF2BP3 mRNA and protein in COAD. (a, b) IGF2BP3 mRNA expression of COAD samples and normal tissues in TCGA database and in the GSE 41258 dataset; (c, d) IGF2BP3 protein expression is upregulated in a COAD sample compared to normal tissue, based on the HPA database.

We compared IGF2BP3 mRNA expression between COAD samples and normal tissues in TCGA and the GSE 41258 dataset. Interestingly, the higher expression of IGF2BP3 was found in COAD samples in both TCGA and the GSE 41258 dataset (Figures 2(a) and 2(b)). In addition, measuring IGF2BP3 protein levels in the HPA database revealed that IGF2BP3 protein expression is elevated in COAD tissue (Figures 2(c) and 2(d)).

### 3.3. Correlation Analysis of IGF2BP3 Expression and Clinicopathological Features

**Figure 3:**
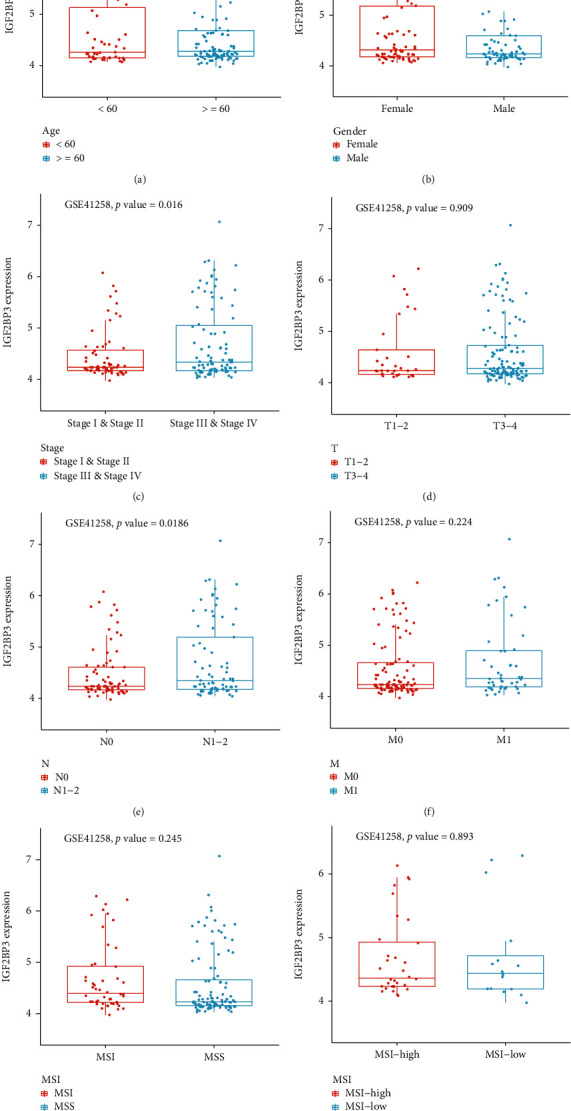
Relationship between the expression of IGF2BP3 and clinicopathological features in the GSE 41258 dataset. (a) Age, (b) gender, (c) stage, (d) pathologic T-stage, (e) pathologic N-stage, (f) pathologic M-stage, (g) MSI and MSS, and (h) MSI high and MSI low.

We compared IGF2BP3 expression in TCGA with clinical traits in the GSE 41258 dataset to explore whether the expression of IGF2BP3 promotes the progression of COAD (Figure 3). As expected, IGF2BP3 expression is associated with the stage and pathologic N-stage in the GSE 41258 dataset (Figures 3(c) and 3(e)). Notably, in TCGA, the expression of IGF2BP3 was not related to any clinical trait (Figure S1), which might be because of limitations in the COAD samples. Therefore, it is necessary to investigate the impact of IGF2BP3 in the development of COAD.

### 3.4. IGF2BP3 Expression Is an Independent Prognostic Factor in COAD Patients

**Figure 4:**
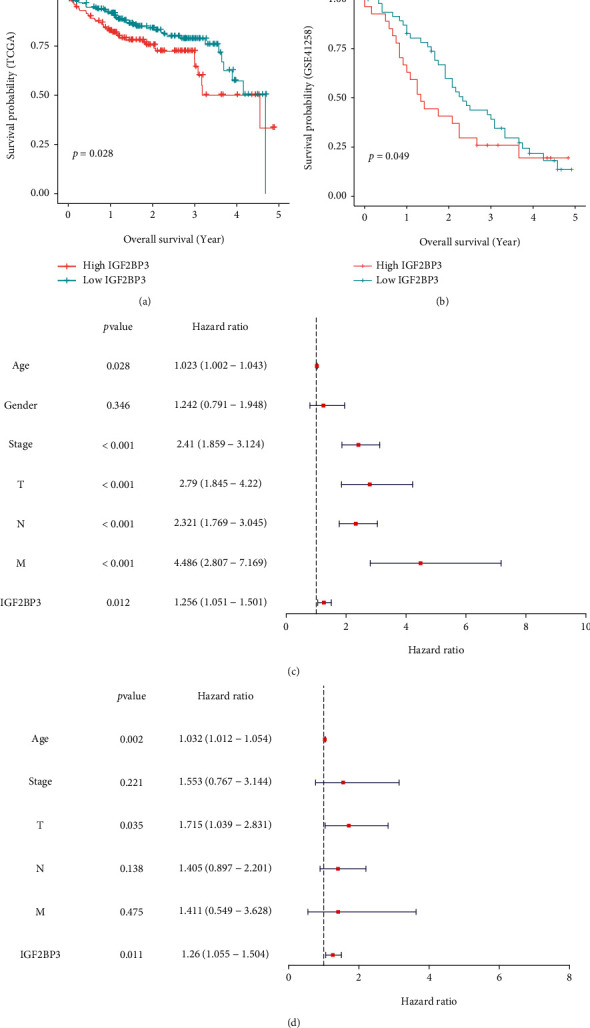
The prognosis value of IGF2BP3 expression. (a, b) Kaplan-Meier survival curves of IGF2BP3 expression and prognosis in TCGA and the GSE 41258 dataset; (c, d) univariate and multivariate Cox regression analyses of IGF2BP3 expression in TCGA.

The correlations of IGF2BP3 expression groups and OS in COAD patients in TGCA and the GSE 41258 dataset were analyzed to investigate the prognostic value of IGF2BP3. We can see that the expression of IGF2BP3 was statistically related to OS in both datasets (Figures 4(a) and 4(b)). The high expression IGF2BP3 group had higher OS (Figures 4(a) and 4(b)). And we found that IGF2BP3 had a good independent value in COAD patients (Figures 4(c) and 4(d)).

### 3.5. Function Analysis of IGF2BP3

**Figure 5:**
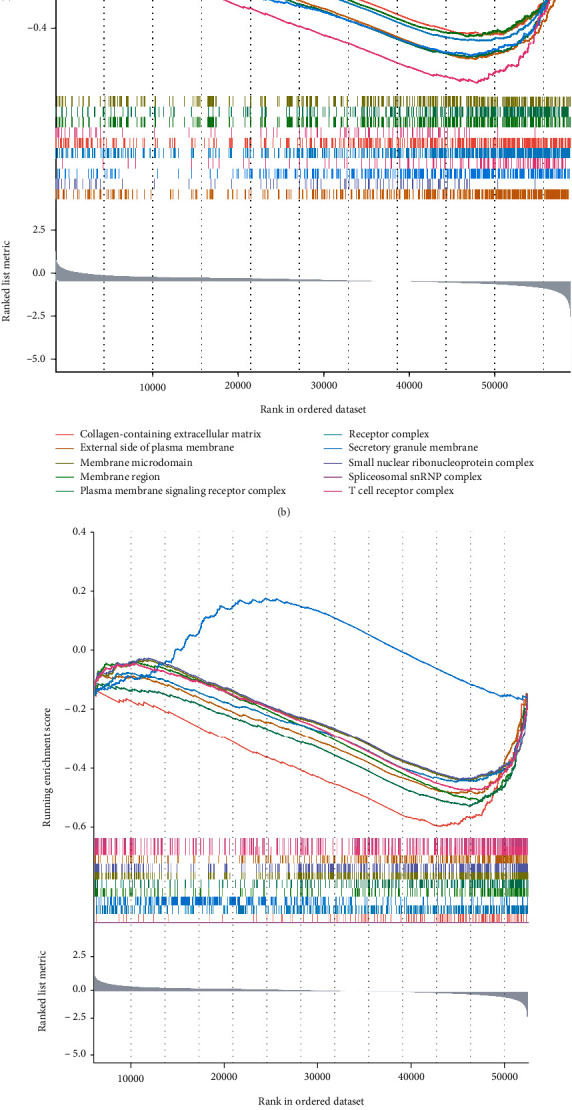
Enriched analysis of DEGs between high- and low-IGF2BP3 expression groups. (a) Biological processes, (b) cellular components, (c) molecular functions, and (d) KEGG pathways.

The GSEA was applied for exploring the biological processes and pathways of DEGs. The DEGs were primarily involved in the regulation of cell killing and immune-related biological processes, including the natural killer cell-mediated immunity and neutrophil activation involved in the immune response (Figure 5(a)). DEGs were primarily related to the cellular components of the extracellular matrix and plasma membrane transport and the T-cell receptor complex (Figure 5(b)). DEGs were associated with the molecular functions of antigen, carbohydrate, and channel binding and cytokine and endopeptidase activities (Figure 5(c)). Furthermore, the DEGs are most enriched in several cancer-related signaling pathways, such as MAPK, P13K-AKT, and Ras (Figure 5(d)); all of which play important roles in COAD tumors.

### 3.6. Relationship of IGF2BP3 to the Tumor Microenvironment of COAD

**Figure 6:**
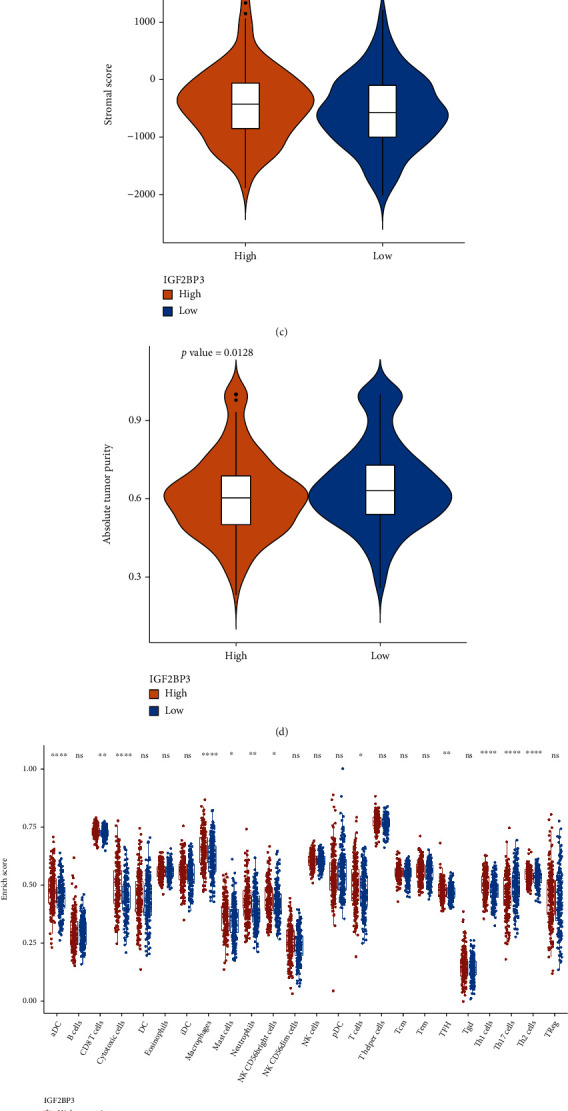
Relationship between IGF2BP3 expression and infiltrating immune cells. (a) The distributions of estimate scores; (b) the distributions of immune scores; (c) the distributions of stromal scores; (d) the distributions of tumor purity; (e) ssGSEA scores for 24 immune cell types; (f) correlations between IGF2BP3 expression and 24 immune cell types.

In this study, we found that IGF2BP3 might affect the immune response in COAD patients. The stromalscore, immunescore, and ESTIMATEscore of high-IGF2BP3 expression groups were statistically higher than those of the low-IGF2BP3 expression group (Figures 6(a)–6(c)). And the tumor purity of low-IGF2BP3 expression groups was higher (Figure 6(d)). 12 types of infiltrating immune cell, including aDC, T cells, TFH, CD8 T-cells, mast cells, cytotoxic cells, macrophages, neutrophils, NK CD56bright cells, Th1, Th2, and Th17 cells, showed significant differences in two groups (Figure 6(e)). Moreover, the correlations of IGF2BP3 expression and the proportions of infiltrating immune cell types were analyzed and the results indicated that the expression of gene IGF2BP3 was positively related with NK CD56dim cells, T helper cells, eosinophils, DC, and macrophage neutrophils and negatively correlated with CD8 T cells (Figure 6(f)). These findings explored that IGF2BP3 expression can impact the composition of the COAD tumor microenvironment.

### 3.7. IGF2BP3 Predicts the Response to Immune Therapy

**Figure 7:**
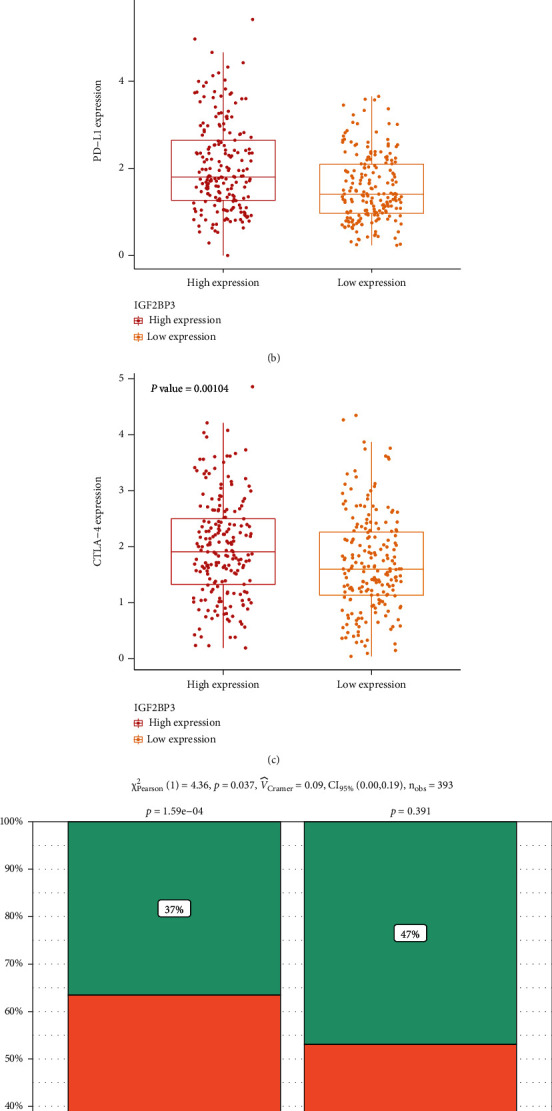
Relationship between IGF2BP3 expression and immune therapy. (a) PD-1 expression levels; (b) PD-L1 expression levels; (c) CTLA-4 expression levels; (d) response to immune checkpoint blockade therapy.

As expected, we found significant upregulation of PD-1, CTLA-4, and PD-L1 in the group with high IGF2BP3 expression (Figures 7(a)–7(c)). We also found that the high-IGF2BP3 expression group had more substantial responses to PD-1 and CTLA-4 (Figure 7(d)). Therefore, IGF2BP3 may serve as a biomarker for predicting immunotherapy response.

### 3.8. IGF2BP3 Predicts the Response to Immune Therapy

**Figure 8:**
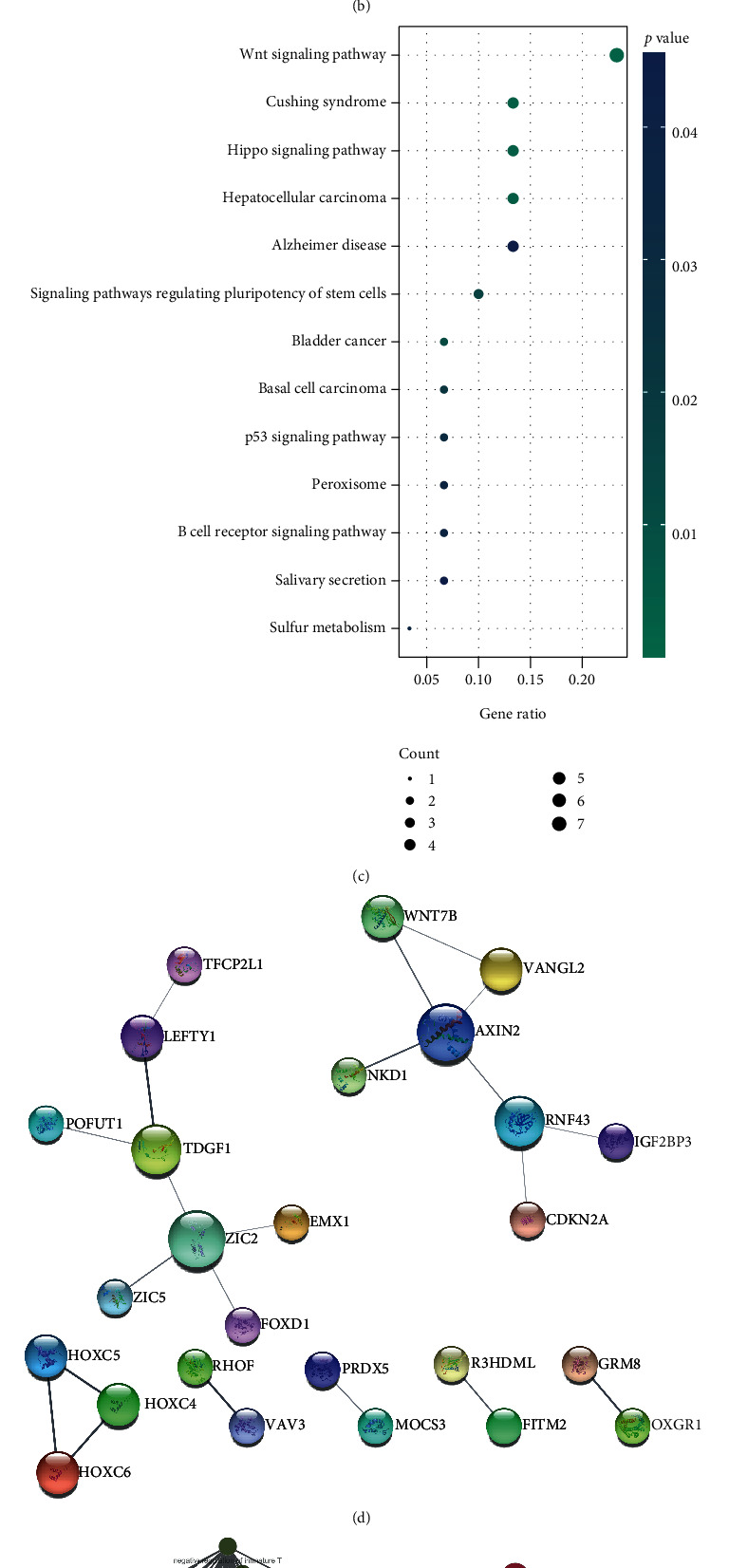
The regulatory mechanism of IGF2BP3. (a) The overlapping genes between the DEGs (high vs low IGF2BP3 expression, blue circle) and genes that are coexpressed with IGF2BP3 (green circle); (b) enriched biological processes of IGF2BP3-related genes; (c) enriched KEGG pathways of IGF2BP3-related genes; (d) PPI network of IGF2BP3 and IGF2BP3-related genes; (e) interaction networks of biological processes related to IGF2BP3 and IGF2BP3-related genes.

We identified 162 genes that are coexpressed with IGF2BP3 using the LinkedOmics database (Table S3) to investigate the regulatory mechanism of IGF2BP3. We also screened 2767 DEGs (1292 upregulated and 1475 downregulated) (Figure S2, Table S4). A Venn diagram was constructed with coexpressed genes and DEGs, and 68 IGF2BP3-related genes of interest were identified in the overlapping portion and analyzed further (Figure 8(a), Table S5).

GO annotation of biological processes showed that the 68 IGF2BP3-related genes are primarily involved in regionalization, pattern specification, and anterior/posterior pattern specification (Figure 8(b)). KEGG pathway analysis pointed out that these genes were enriched in Wnt, Hippo, P53, and B-cell receptor signaling pathways (Figure 8(c), Table S6). A PPI network consisting of IGF2BP3 and IGF2BP3-related genes showed that there were direct and indirect interactions between IGF2BP3 and WNT7B, VANGL2, NKD1, AXIN2, RNF43, and CDKN2A (Figure 8(d)). The interaction networks of biological processes for IGF2BP3 and IGF2BP3-related genes were shown in Figure 8(e).

### 3.9. IGF2BP3 Expression Is Elevated in Human Colon Cancer Tissues

**Figure 9:**
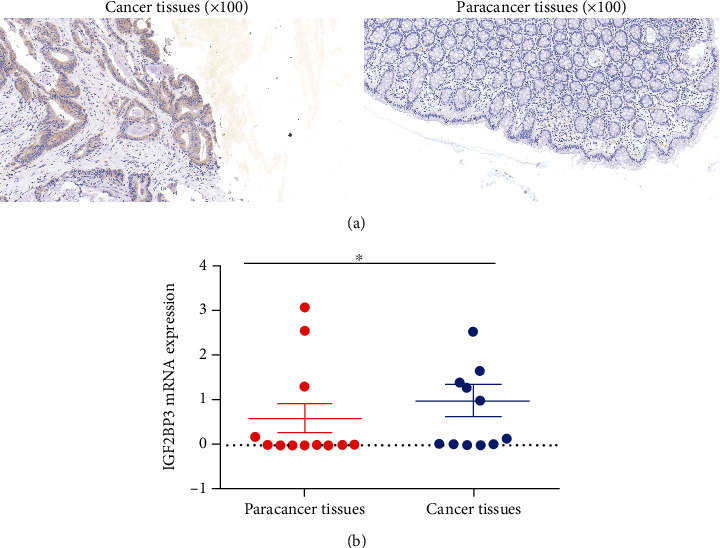
IGF2BP3 expression in human colon cancer tissues. (a) Immunohistochemical staining of IGF2BP3 protein in colon cancer tissues and paracancer tissues; (b) quantitative analysis of IGF2BP3 mRNA.

We performed IHC staining and real-time qPCR using 12 colon cancer tissues and 12 matched paracancer tissues from 12 COAD patients to measure the expression of IGF2BP3 in COAD patient tissues. As expected, IHC staining suggested that IGF2BP3 protein levels were significantly higher in colon cancer tissues (Figure 9(a)). Moreover, real-time qPCR results indicated that IGF2BP3 mRNA levels in colon cancer tissues were elevated relative to paracancer tissues (Figure 9(b)).

## 4. Discussion

In previous studies, IGF2BP3 was highly expressed in most tumor tissues except cutaneous melanoma. We found increased accumulation of IGF2BP3 mRNA and protein expression in COAD and human colon cancer tissues. We learned that high expression of IGF2BP3 is often related to a poorer OS in COAD samples of TCGA and the GSE 41258 dataset and IGF2BP3 is an independent prognostic biomarker for COAD patients. Functional annotation revealed that IGF2BP3 might affect the immune response in COAD patients. Notably, we found that IGF2BP3 may participate in MAPK, P13K-AKT, and Ras signaling pathways, which are significantly related to tumorigenesis. And the significant differences of the tumor microenvironment in different IGF2BP3 expression groups were explored. Applying the TIDE algorithm suggested that IGF2BP3 predicts the efficiency of immunotherapy. Overall, in this study, we proved that IGF2BP3 is an independent prognostic biomarker in COAD patients and could be a therapeutic target for COAD. Moreover, IGF2BP3 could be used to develop personalized immunotherapies for COAD patients.

The evidence that IGF2BP3 induces carcinogenesis and participates in multiple biological processes is increasing. For instance, Yang et al. found that downregulation of IGF2BP3 represses DNA replication in the cell cycle S phase and stimulates angiogenesis by regulating m6A modifications of cyclin D1 (CCND1) and vascular endothelial growth factor (VEGF) mRNAs, respectively [15]. Moreover, the IGF2BP3/ELAV-like RNA binding protein 1 (ELAVL1) complex is involved in the stabilization of oncogenic transcripts, thus promoting tumorigenicity of colorectal cancer [26]. You et al. demonstrated that IGF2BP3 is confirmed to be involved in colorectal epithelial-mesenchymal transition (EMT) of cancer cells [27]. Furthermore, chemoresistance of HCT8 cells can be triggered by IGF2BP3 and M6A-modified RNA complexes via upregulation of ATP-binding box subfamily B member 1 (ABCB1) [28]. Some research has indicated that IGF2BP3 has good prognostic value as a prognostic biomarker for patients with colon cancer [14, 29]. Therefore, our study adds to this evidence.

According to the latest NCCN colon cancer guidelines (2021 V2), PD-1 inhibitors or PD-1 inhibitors combined with low-dose CTLA4 inhibitors can be used as first-line treatments for dMMR/MSI-H metastatic colon cancer [30]. The results of the second interim analysis of KEYNOTE-177 showed that pablizumab as a first-line treatment significantly improves the progression-free survival (PFS) of dMMR/MSI-H metastatic colon cancer patients [31]. Moreover, the Checkmate 142 study showed that the overall response rate (ORR) to first-line use of nivolumab combined with ipilimumab was significantly higher than navolizumab alone (55% vs. 31%) and the toxicity was controllable [32]. However, there are still some patients with dMMR/MSI-H metastatic colon cancer who do not benefit from immunotherapy. We found that IGF2BP3 may be a biomarker that predicts the effect of immunotherapy, similar to MSI. However, determining whether the combination of MSI and IGF2BP3 improves the accuracy of predicting the effect of immunotherapy relative to MSI alone requires additional study.

PPI network analysis showed that there are direct and indirect interactions between IGF2BP3 and Wnt7b and Axin2. Wnt7b and Axin2 are two key proteins in the Wnt signaling pathway, a complex protein interaction network involved in embryonic development, tissue homeostasis, and cell carcinogenesis [33, 34]. The m6A RNA methylation regulator YTHDF1 has been shown to amplify Wnt/*β*-catenin signaling during translation. This amplification is necessary to maintain intestinal stem cells during regeneration and tumorigenesis [35]. The reduction of m6A RNA modification can activate the Wnt/PI3K-Akt signaling pathway, leading to the accelerated occurrence of the malignant phenotype in gastric cancer cells [36]. Nevertheless, the correlation between IGF2BP3 and the Wnt signaling pathway remains unclear; it is necessary to explore it.

## 5. Conclusions

Independent prognostic analysis, GSEA analysis, survival analysis, and other bioinformatics methods were used to investigate the mechanism of IGF2BP3 in colon cancer. Immunohistochemistry and qPCR were used to analyze the difference between IGF2BP3 protein and mRNA expression in cancer tumors and adjacent tissues. IGF2BP3 expression is elevated in COAD and colon cancer tissues. IGF2BP3 is an independent prognostic biomarker in COAD patients and could be used as a therapeutic target. Moreover, IGF2BP3 could be used to personalize immunotherapies for COAD patients. IGF2BP3 might also be a reference to monitor the treatment of colon cancer and explore molecular mechanisms related to the progression of colon cancer. However, it is necessary to further verify the mechanisms and functions of IGF2BP3 in colon cancer by in vivo and in vitro experiments.

## Data Availability

Our data are freely downloaded from TCGA (https://portal.gdc.cancer.gov/) and the GSE 41258 dataset (https://www.ncbi.nlm.nih.gov/geo/query/acc.cgi).

## Supplementary Materials

Supplementary Materials See Figures S1 in the Supplementary Material for correlations between expression of IGF2BP3 and clinicopathological features in TCGA database, Figure S2 and Table S4 for the DEGs in groups with different expression of IGF2BP3, Table S1 and Table S2 for the clinical information of the samples, Table S3 for gene coexpressed with IGF2BP3, Table S5 for the genes related to IGF2BP3, and Table S6 for the results of KEGG enrichment analysis.
