# Supplementary material for: Abnormal Expression of N6-Methyladenosine RNA Methylation Regulator IGF2BP3 in Colon Cancer Predicts a Poor Prognosis
Source: Dis Markers. 2022 May 30;2022:5883101. doi: 10.1155/2022/5883101 (PMC9170420; doi:10.1155/2022/5883101)
Supplement: Supplementary Materials — See Figures S1 in the Supplementary Material for correlations between expression of IGF2BP3 and clinicopathological features in TCGA database, Figure S2 and Table S4 for the DEGs in groups with different expression of IGF2BP3, Table S1 and Table S2 for the clinical information of the samples, Table S3 for gene coexpressed with IGF2BP3, Table S5 for the genes related to IGF2BP3, and Table S6 for the results of KEGG enrichment analysis. [file 5883101.f1.zip › Table S1 (2).docx]

| Query | Statistic | P-value | FDR (BH) | Event_SD | Event_TD |
| --- | --- | --- | --- | --- | --- |
| A1BG | 0.0498842094062108 | 0.332776162942512 | 0.493551182498626 | 379 | 379 |
| A1CF | -0.119090775693246 | 0.0203923044974549 | 0.0631384468419011 | 379 | 375 |
| A2BP1 | -0.261408844479531 | 2.43916909094348E-07 | 9.87017239494435E-06 | 379 | 270 |
| A2LD1 | 0.000357721648840262 | 0.994461852813784 | 0.996673555276573 | 379 | 379 |
| A2ML1 | 0.144468723372043 | 0.00483218031713539 | 0.0208650852195471 | 379 | 178 |
| A2M | 0.11715037864874 | 0.0225484144612968 | 0.0681955402590898 | 379 | 379 |
| A4GALT | 0.197184635736445 | 0.000111499872985413 | 0.00108479856798565 | 379 | 379 |
| A4GNT | 0.0253976633091457 | 0.622093116273618 | 0.748838168375018 | 379 | 146 |
| AAA1 | 0.267318265572372 | 1.2681138906633E-07 | 6.00099337090023E-06 | 379 | 211 |
| AAAS | 0.195935253735595 | 0.000123471712775417 | 0.0011741952618278 | 379 | 379 |
| AACSL | 0.109768075379618 | 0.0326503192772782 | 0.0907217671846793 | 379 | 121 |
| AACS | 0.0137046950441243 | 0.790290704294682 | 0.871614422335908 | 379 | 379 |
| AADACL2 | -0.00364823303287286 | 0.943565567087957 | 0.968112306656595 | 379 | 40 |
| AADACL3 | -0.0429941498522041 | 0.403927197893164 | 0.563067244082231 | 379 | 29 |
| AADACL4 | 0.0319691008818528 | 0.534945246689158 | 0.680801948097088 | 379 | 42 |
| AADAC | 0.128854902305584 | 0.0120483136108213 | 0.0421909657615536 | 379 | 358 |
| AADAT | 0.033281644936786 | 0.518303156183852 | 0.666380169939919 | 379 | 379 |
| AAGAB | 0.181594066452142 | 0.000380621798826854 | 0.002842549539412 | 379 | 379 |
| AAK1 | -0.0296374738447963 | 0.565155672875903 | 0.705447886174951 | 379 | 379 |
| AAMP | 0.103877636544309 | 0.0432722693358778 | 0.112098351754198 | 379 | 379 |
| AANAT | 0.153477588275138 | 0.0027373606767634 | 0.0134218849634558 | 379 | 221 |
| AARS2 | 0.175369225286377 | 0.000604893001361075 | 0.00411875632932259 | 379 | 379 |
| AARSD1 | 0.0499403627989419 | 0.332231555308035 | 0.493038291814045 | 379 | 379 |
| AARS | 0.0644764115480726 | 0.210431783427098 | 0.356985061755005 | 379 | 379 |
| AASDHPPT | 0.01239862625943 | 0.809874887854004 | 0.8847492714253 | 379 | 379 |
| AASDH | -0.0111005691676002 | 0.829457166486542 | 0.897341591941027 | 379 | 379 |
| AASS | -0.0835167425391377 | 0.10451161380423 | 0.215837545933785 | 379 | 379 |
| AATF | 0.0872363510825741 | 0.0898981528368209 | 0.19414424966784 | 379 | 379 |
| AATK | 0.0670513445587131 | 0.192743878378073 | 0.335828261905134 | 379 | 379 |
| ABAT | -0.330227160773132 | 4.29652223250339E-11 | 1.11594329160245E-08 | 379 | 379 |
| ABCA10 | -0.0157571456722725 | 0.759783886361881 | 0.850281791069525 | 379 | 375 |
| ABCA11P | -0.176132958475424 | 0.000571940302843151 | 0.00393082576248665 | 379 | 379 |
| ABCA12 | 0.05341667844881 | 0.299634547871886 | 0.458634693160704 | 379 | 294 |
| ABCA13 | 0.0276444822807465 | 0.59160925037535 | 0.725713203194904 | 379 | 375 |
| ABCA17P | -0.0313389284073061 | 0.543029658113856 | 0.687691898900271 | 379 | 328 |
| ABCA1 | -0.0294170702987389 | 0.568053112534101 | 0.707540431406564 | 379 | 379 |
| ABCA2 | 0.029312317713819 | 0.569432674069004 | 0.708725821444995 | 379 | 379 |
| ABCA3 | 0.0984591682365628 | 0.0554772589316373 | 0.135085728858714 | 379 | 379 |
| ABCA4 | 0.00681991904563709 | 0.894721197256393 | 0.939384222160202 | 379 | 347 |
| ABCA5 | -0.116729650621175 | 0.023041122567378 | 0.0693156392453302 | 379 | 379 |
| ABCA6 | 0.062001052920548 | 0.228510938598333 | 0.378332906690693 | 379 | 371 |
| ABCA7 | 0.0758617732115661 | 0.140449908210696 | 0.26774740698026 | 379 | 379 |
| ABCA8 | 0.0311360629106275 | 0.545645032668401 | 0.689902417277711 | 379 | 348 |
| ABCA9 | 0.0454330735723152 | 0.377765197536905 | 0.538193766512405 | 379 | 363 |
| ABCB10 | -0.194951084453847 | 0.000133741191995853 | 0.00125500253426113 | 379 | 379 |
| ABCB11 | 0.217055541329967 | 0.0000202073310444769 | 0.000296573619503988 | 379 | 264 |
| ABCB1 | -0.0497433035866393 | 0.334145274293693 | 0.495248355411522 | 379 | 379 |
| ABCB4 | -0.0579964580920687 | 0.260043100793414 | 0.41424717622976 | 379 | 378 |
| ABCB5 | 0.0965739013567474 | 0.0603435096586093 | 0.143703233543228 | 379 | 210 |
| ABCB6 | 0.033605047856268 | 0.514243969111783 | 0.662943467386616 | 379 | 379 |
| ABCB7 | 0.0475627414847562 | 0.355792596013901 | 0.516900321934615 | 379 | 379 |
| ABCB8 | 0.196462328993088 | 0.000118281019780425 | 0.0011357269056689 | 379 | 379 |
| ABCB9 | 0.00918292777025848 | 0.858577088041486 | 0.917085950637644 | 379 | 379 |
| ABCC10 | -0.151707110756239 | 0.00306819169138719 | 0.0146663705055027 | 379 | 379 |
| ABCC11 | -0.0329477625146177 | 0.522511118258686 | 0.670009083155482 | 379 | 346 |
| ABCC12 | 0.069610446050072 | 0.176268075015411 | 0.315337311149616 | 379 | 18 |
| ABCC13 | 0.00827744134290132 | 0.872396601412092 | 0.925515238780041 | 379 | 352 |
| ABCC1 | -0.0517189461663465 | 0.31527894787434 | 0.475134983541265 | 379 | 379 |
| ABCC2 | 0.0703774676076454 | 0.171539676541591 | 0.309125730104171 | 379 | 379 |
| ABCC3 | 0.00743847485912613 | 0.885235696641932 | 0.933244012814559 | 379 | 379 |
| ABCC4 | -0.154914729741341 | 0.00249305818317628 | 0.0124326855271678 | 379 | 379 |
| ABCC5 | -0.324156011623341 | 1.01169297515164E-10 | 2.18041829470726E-08 | 379 | 379 |
| ABCC6P1 | -0.132289840174151 | 0.00993043816827983 | 0.0363956983365347 | 379 | 377 |
| ABCC6P2 | -0.291405385923969 | 7.45931821101577E-09 | 6.97657365509532E-07 | 379 | 379 |
| ABCC6 | -0.183954976629318 | 0.00031801941230084 | 0.00246412227710084 | 379 | 379 |
| ABCC8 | 0.0108442319242922 | 0.833337148943949 | 0.900023915993331 | 379 | 295 |
| ABCC9 | 0.127466997513162 | 0.0130113345420589 | 0.0447741654460159 | 379 | 379 |
| ABCD1 | 0.124746618537724 | 0.0150973734605526 | 0.0501593031125734 | 379 | 379 |
| ABCD2 | 0.11861089414067 | 0.0209081483373937 | 0.0643937193590934 | 379 | 369 |
| ABCD3 | -0.0283386733530625 | 0.582330883211474 | 0.718599499148439 | 379 | 379 |
| ABCD4 | 0.0543658573649118 | 0.291116657692278 | 0.449553044293029 | 379 | 379 |
| ABCE1 | 0.125571254340247 | 0.0144360302523146 | 0.0485630635320506 | 379 | 379 |
| ABCF1 | 0.136005964400153 | 0.00801735705082962 | 0.0309096161003013 | 379 | 379 |
| ABCF2 | 0.0406089847470544 | 0.430529211234224 | 0.587390367714412 | 379 | 379 |
| ABCF3 | -0.10396072114105 | 0.0431043194150131 | 0.111809582072329 | 379 | 379 |
| ABCG1 | -0.129472950252095 | 0.011640113964086 | 0.0410457370940598 | 379 | 379 |
| ABCG2 | 0.160921339477093 | 0.00167246619899812 | 0.00910284375342702 | 379 | 378 |
| ABCG4 | 0.14368720803741 | 0.00506911697287835 | 0.0216897385441243 | 379 | 364 |
| ABCG5 | -0.0346300298506599 | 0.501488558608875 | 0.651393065188128 | 379 | 360 |
| ABCG8 | 0.0232155352070145 | 0.65233116193165 | 0.771237390661306 | 379 | 289 |
| ABHD10 | -0.0286819502184172 | 0.57776788558562 | 0.714837241694227 | 379 | 379 |
| ABHD11 | -0.0017215320591875 | 0.973352441781374 | 0.985932680237092 | 379 | 379 |
| ABHD12B | 0.138274032372551 | 0.00701822289545192 | 0.0280107334080154 | 379 | 320 |
| ABHD12 | -0.0232061712913021 | 0.652462200277077 | 0.771270329057249 | 379 | 379 |
| ABHD13 | -0.0826053546424048 | 0.108364532399377 | 0.221689331144088 | 379 | 379 |
| ABHD14A | -0.0577514240889514 | 0.262065419605683 | 0.41655141579882 | 379 | 379 |
| ABHD14B | -0.178572704529602 | 0.000477496025821216 | 0.00339712637243742 | 379 | 379 |
| ABHD15 | 0.137595712164777 | 0.007304677362274 | 0.028850677862466 | 379 | 379 |
| ABHD1 | -0.0264963443686953 | 0.607101545746399 | 0.736515507162237 | 379 | 373 |
| ABHD2 | 0.0347673357798066 | 0.499792616470134 | 0.650091410001845 | 379 | 379 |
| ABHD3 | 0.178344139736082 | 0.00048568634857139 | 0.00344427357634961 | 379 | 379 |
| ABHD4 | 0.152489791586469 | 0.00291770181797127 | 0.0141268527161128 | 379 | 379 |
| ABHD5 | -0.0647437966212904 | 0.208542395833241 | 0.354903323713115 | 379 | 379 |
| ABHD6 | -0.118309979805893 | 0.0212373596666102 | 0.0651143292824411 | 379 | 379 |
| ABHD8 | -0.0393298237476752 | 0.445203272872959 | 0.60053504215988 | 379 | 379 |
| ABI1 | -0.139512607329615 | 0.00652098634669605 | 0.0264522079890254 | 379 | 379 |
| ABI2 | 0.0019437249765669 | 0.96991465344462 | 0.983908101325076 | 379 | 379 |
| ABI3BP | 0.0230305112186298 | 0.654922358241256 | 0.772792743973571 | 379 | 379 |
| ABI3 | 0.2259893506332 | 8.88722501711949E-06 | 0.000159615849311092 | 379 | 379 |
| ABL1 | -0.132290494287804 | 0.0099300685327344 | 0.0363956983365347 | 379 | 379 |
| ABL2 | -0.0336901636095789 | 0.513178383641439 | 0.662068590854608 | 379 | 379 |
| ABLIM1 | -0.0330791007948357 | 0.520853761918564 | 0.668459221329175 | 379 | 379 |
| ABLIM2 | -0.213840069244407 | 0.0000269368329523185 | 0.000367333922818825 | 379 | 379 |
| ABLIM3 | 0.166100770812548 | 0.00117232585575756 | 0.00695746096017987 | 379 | 379 |
| ABO | -0.193931196595184 | 0.000145225539189192 | 0.00133497078861535 | 379 | 379 |
| ABP1 | -0.149837905811263 | 0.00345652214755896 | 0.0161298943614495 | 379 | 379 |
| ABRA | 0.0829140929456878 | 0.107046985813564 | 0.219677875668738 | 379 | 242 |
| ABR | -0.0342462871838653 | 0.506244426738363 | 0.655805482703673 | 379 | 379 |
| ABT1 | -0.0200954300230272 | 0.696563611459882 | 0.803365710099264 | 379 | 379 |
| ABTB1 | -0.0566113233104432 | 0.271617073537932 | 0.427662641112395 | 379 | 379 |
| ABTB2 | -0.100504637106451 | 0.0505705161326026 | 0.125953045330642 | 379 | 379 |
| ACAA1 | -0.10775696518064 | 0.0359949707572592 | 0.0975544396083837 | 379 | 379 |
| ACAA2 | 0.00298063447062004 | 0.95387988634125 | 0.9747735085489 | 379 | 379 |
| ACACA | 0.0777792386943462 | 0.130663896385507 | 0.254274584113439 | 379 | 379 |
| ACACB | -0.165405585746033 | 0.00123032539133923 | 0.00720463433534387 | 379 | 379 |
| ACAD10 | -0.0357446845749844 | 0.487808962727835 | 0.639573901538552 | 379 | 379 |
| ACAD11 | -0.030646912393814 | 0.551976721453107 | 0.695550178602624 | 379 | 379 |
| ACAD8 | 0.00361551256002938 | 0.944070889074399 | 0.968303067396402 | 379 | 379 |
| ACAD9 | -0.0775358756202129 | 0.131875917783733 | 0.255879802115262 | 379 | 379 |
| ACADL | -0.00142506176240552 | 0.977940213226942 | 0.988231086410077 | 379 | 173 |
| ACADM | -0.0898410789670918 | 0.0806762542091223 | 0.178831611901451 | 379 | 379 |
| ACADSB | -0.180987445584175 | 0.000398465662169294 | 0.00294805117518386 | 379 | 379 |
| ACADS | 0.0294090177508403 | 0.568159105752234 | 0.707540431406564 | 379 | 379 |
| ACADVL | 0.0284371658459618 | 0.581019961128506 | 0.717428309207624 | 379 | 379 |
| ACAN | 0.0672849503696371 | 0.191194711820503 | 0.334041117088007 | 379 | 379 |
| ACAP1 | 0.225018050540863 | 9.73333016581146E-06 | 0.000170488048169355 | 379 | 379 |
| ACAP2 | -0.0410171530019803 | 0.4259063633731 | 0.583370501033561 | 379 | 379 |
| ACAP3 | -0.00230941434452136 | 0.964257917341738 | 0.980657246370376 | 379 | 379 |
| ACAT1 | -0.0758724447021982 | 0.140393932934254 | 0.267692172537782 | 379 | 379 |
| ACAT2 | -0.0542050052123728 | 0.292548608187887 | 0.450890976969159 | 379 | 379 |
| ACBD3 | 0.00189702412395756 | 0.970637169056971 | 0.984489937493561 | 379 | 379 |
| ACBD4 | -0.000308945415110855 | 0.995216983192846 | 0.997016569980589 | 379 | 379 |
| ACBD5 | 0.0586701076181592 | 0.254538869909 | 0.408796105018277 | 379 | 379 |
| ACBD6 | -0.198753491498268 | 0.0000980090494398848 | 0.000991997668348155 | 379 | 379 |
| ACBD7 | -0.0831948774667157 | 0.105859739135164 | 0.217737231075937 | 379 | 379 |
| ACCN1 | -0.0745445211989185 | 0.147491454309566 | 0.277621089429474 | 379 | 345 |
| ACCN2 | -0.106164081780851 | 0.0388465648351096 | 0.103430869820136 | 379 | 379 |
| ACCN3 | 0.213691628124243 | 0.0000272938245232436 | 0.000370926629641449 | 379 | 372 |
| ACCN4 | -0.00253222965139578 | 0.960812116154866 | 0.978847004833521 | 379 | 309 |
| ACCN5 | 0.105580180229482 | 0.0399386893175927 | 0.105512753056345 | 379 | 34 |
| ACCSL | 0.0411390495363531 | 0.424531397842516 | 0.582050100706777 | 379 | 20 |
| ACCS | -0.0408642572612271 | 0.427634646962324 | 0.584848929505378 | 379 | 379 |
| ACD | 0.0316083800976786 | 0.539565487010492 | 0.684748110371482 | 379 | 379 |
| ACE2 | -0.150853949792713 | 0.00324023639728517 | 0.0152824470231614 | 379 | 379 |
| ACER1 | 0.0194981492590052 | 0.705155317277154 | 0.810023731589793 | 379 | 105 |
| ACER2 | 0.0632390119966022 | 0.219336140946292 | 0.367407029034644 | 379 | 379 |
| ACER3 | 0.209216412897958 | 0.0000404185915411548 | 0.000505627654938813 | 379 | 379 |
| ACE | -0.099772437795346 | 0.0522836078999173 | 0.129049829704265 | 379 | 379 |
| ACHE | -0.00917687809280796 | 0.858669279884984 | 0.917135018399023 | 379 | 379 |
| ACIN1 | 0.097529935281913 | 0.0578334753812399 | 0.139114660907342 | 379 | 379 |
| ACLY | 0.189741701218841 | 0.000202816809097108 | 0.00173637810482619 | 379 | 379 |
| ACMSD | 0.0828516767726312 | 0.107312323484803 | 0.220062958946807 | 379 | 79 |
| ACN9 | 0.0481418570514191 | 0.349959334175602 | 0.511423472732446 | 379 | 379 |
| ACO1 | -0.110710829503234 | 0.0311760365655912 | 0.0874216451736024 | 379 | 379 |
| ACO2 | 0.0132441134603175 | 0.797182655478872 | 0.87624245761046 | 379 | 379 |
| ACOT11 | -0.0541286557175678 | 0.293229938589405 | 0.451620570323965 | 379 | 379 |
| ACOT12 | -0.0796000618520847 | 0.121866947699018 | 0.241251781047937 | 379 | 27 |
| ACOT13 | 0.0208098095707619 | 0.686338250208619 | 0.796359913789205 | 379 | 379 |
| ACOT1 | -0.0631485837363827 | 0.219997272539439 | 0.368265590537104 | 379 | 379 |
| ACOT2 | -0.0921174886344644 | 0.073257577096094 | 0.166005855847012 | 379 | 379 |
| ACOT4 | -0.0408064204383487 | 0.428289474464404 | 0.585421460063435 | 379 | 379 |
| ACOT6 | -0.0235013060597025 | 0.648337249910367 | 0.768926983428873 | 379 | 223 |
| ACOT7 | 0.308421006774611 | 8.53353086187727E-10 | 1.29162480862063E-07 | 379 | 379 |
| ACOT8 | -0.236331469624376 | 3.29139994710213E-06 | 0.0000739092617793218 | 379 | 379 |
| ACOT9 | -0.0372290518594543 | 0.469907974492676 | 0.62273327885582 | 379 | 379 |
| ACOX1 | -0.277619364372226 | 3.90246413168325E-08 | 2.48006598727614E-06 | 379 | 379 |
| ACOX2 | -0.232587423970998 | 4.74079736322863E-06 | 0.0000975109233590221 | 379 | 379 |
| ACOX3 | -0.0967516377285327 | 0.0598701797865668 | 0.142869891058857 | 379 | 379 |
| ACOXL | 0.168961727125661 | 0.000959155346469263 | 0.00595060457127426 | 379 | 344 |
| ACP1 | 0.0122437767169604 | 0.812204960671284 | 0.886253994962541 | 379 | 379 |
| ACP2 | 0.157030254357851 | 0.00216945110753176 | 0.0111353550505151 | 379 | 379 |
| ACP5 | 0.125302878602669 | 0.0146484139693757 | 0.0490602940497404 | 379 | 379 |
| ACP6 | -0.119605206596962 | 0.0198516367418084 | 0.0619091307512705 | 379 | 379 |
| ACPL2 | -0.0641871449487056 | 0.212489647962989 | 0.359233014471257 | 379 | 379 |
| ACPP | 0.112892503736028 | 0.0279810183449584 | 0.0806406441488133 | 379 | 378 |
| ACPT | -0.038275859118022 | 0.457504454277621 | 0.611816167762641 | 379 | 135 |
| ACRBP | 0.125722863088023 | 0.0143172516163411 | 0.04823831181798 | 379 | 379 |
| ACRC | -0.0238358144616778 | 0.643674961630345 | 0.765889770715703 | 379 | 379 |
| ACRV1 | 0.133113109044883 | 0.0094747634733309 | 0.0350644993060458 | 379 | 336 |
| ACR | -0.0633212299334774 | 0.218736270160217 | 0.366649992792019 | 379 | 361 |
| ACSBG1 | 0.100244061107974 | 0.0511747254717022 | 0.127011197478146 | 379 | 378 |
| ACSBG2 | 0.03447505061102 | 0.503406433854738 | 0.653028640528083 | 379 | 295 |
| ACSF2 | -0.19718731048938 | 0.000111475454787379 | 0.00108479856798565 | 379 | 379 |
| ACSF3 | -0.274240097261759 | 5.77546210399203E-08 | 3.24407542770408E-06 | 379 | 379 |
| ACSL1 | 0.134873186826234 | 0.00856236635513187 | 0.0324741010117741 | 379 | 379 |
| ACSL3 | 0.010799746781111 | 0.834010901503462 | 0.900459621155699 | 379 | 379 |
| ACSL4 | 0.00140535097063549 | 0.978245258093752 | 0.988271851564948 | 379 | 379 |
| ACSL5 | -0.241962131346692 | 1.87972959775706E-06 | 0.0000477225076367824 | 379 | 379 |
| ACSL6 | -0.264067050131821 | 1.82098628473527E-07 | 0.000007900769377184 | 379 | 374 |
| ACSM1 | -0.0865222886865504 | 0.0925686820967122 | 0.198448678626188 | 379 | 367 |
| ACSM2A | 0.0408971059135303 | 0.427262993808096 | 0.58458257253843 | 379 | 69 |
| ACSM2B | -0.054471802189333 | 0.29017607594665 | 0.448649993283647 | 379 | 45 |
| ACSM3 | -0.0459747787445668 | 0.372098735605116 | 0.532705684446082 | 379 | 379 |
| ACSM4 | 0.0358826228211211 | 0.486130172260345 | 0.637921181706031 | 379 | 25 |
| ACSM5 | 0.0766593551393961 | 0.136313254266858 | 0.261926466285809 | 379 | 304 |
| ACSS1 | -0.1353686419246 | 0.00832005584348454 | 0.0318167921436088 | 379 | 379 |
| ACSS2 | -0.144204767617752 | 0.00491106511380396 | 0.0211111426516689 | 379 | 379 |
| ACSS3 | 0.0181531156888487 | 0.724639440415233 | 0.824618389838914 | 379 | 375 |
| ACTA1 | 0.00078039961644438 | 0.987918433191768 | 0.993429693342448 | 379 | 367 |
| ACTA2 | 0.10550588713181 | 0.0400794894858916 | 0.105790217988054 | 379 | 379 |
| ACTBL2 | 0.176357769319191 | 0.000562562400104344 | 0.00388117163161758 | 379 | 195 |
| ACTB | 0.227190885956632 | 7.93721468159702E-06 | 0.000145452026531151 | 379 | 379 |
| ACTC1 | 0.0792880589719767 | 0.123340678919608 | 0.243464308772324 | 379 | 366 |
| ACTG1 | 0.100995969509871 | 0.0494474336220009 | 0.123934232569465 | 379 | 379 |
| ACTG2 | 0.0218486971617358 | 0.671569927705642 | 0.785551797920327 | 379 | 379 |
| ACTL6A | -0.111057376566019 | 0.0306485950214437 | 0.0863088115445512 | 379 | 379 |
| ACTL6B | 0.06479325347258 | 0.208194271750967 | 0.354576827105444 | 379 | 57 |
| ACTL7A | -0.0245257310686936 | 0.634103244673493 | 0.758460465427159 | 379 | 33 |
| ACTL7B | -0.0174509548249188 | 0.734882886175316 | 0.831787753572564 | 379 | 97 |
| ACTL8 | 0.210680612026652 | 0.0000355785639616663 | 0.000456951737128158 | 379 | 368 |
| ACTL9 | 0.0322426744993211 | 0.531454518265398 | 0.6777950851075 | 379 | 12 |
| ACTN1 | -0.00103178063959369 | 0.984027193798403 | 0.990974208879818 | 379 | 379 |
| ACTN2 | 0.0960716990400039 | 0.0616976474486505 | 0.145983407352249 | 379 | 254 |
| ACTN3 | 0.0557166648691962 | 0.279276777947192 | 0.436092294309097 | 379 | 325 |
| ACTN4 | 0.0319756027434792 | 0.534862151213583 | 0.680739889226711 | 379 | 379 |
| ACTR10 | -0.021204338309275 | 0.680715385285552 | 0.79223012616317 | 379 | 379 |
| ACTR1A | 0.154265823936057 | 0.00260079543150866 | 0.0128824810931686 | 379 | 379 |
| ACTR1B | -0.121331829080743 | 0.0181270548264205 | 0.0577293997909196 | 379 | 379 |
| ACTR2 | -0.0100280978800297 | 0.845716861618848 | 0.908438915010484 | 379 | 379 |
| ACTR3B | -0.0467900492675777 | 0.363670207093658 | 0.524769148260902 | 379 | 379 |
| ACTR3C | -0.0511526352773347 | 0.320614226023319 | 0.481054776662154 | 379 | 379 |
| ACTR3 | 0.0411206391460574 | 0.424738897086734 | 0.582294327002404 | 379 | 379 |
| ACTR5 | -0.173978280537038 | 0.000669469701934575 | 0.00448454231417525 | 379 | 379 |
| ACTR6 | 0.019467801472576 | 0.705592873729506 | 0.81024471537086 | 379 | 379 |
| ACTR8 | -0.319966030116169 | 0.0000000001806968226 | 0.0000000360693221055 | 379 | 379 |
| ACTRT1 | -0.026602267450478 | 0.605664747810118 | 0.735939520132573 | 379 | 39 |
| ACVR1B | -0.205555465388406 | 0.0000553858244891257 | 0.000641466196244383 | 379 | 379 |
| ACVR1C | -0.0841838884437826 | 0.101760549776543 | 0.212122390766326 | 379 | 379 |
| ACVR1 | -0.014613160174936 | 0.776745066907077 | 0.862528068247846 | 379 | 379 |
| ACVR2A | -0.133493521146249 | 0.00927055416129838 | 0.0345195395136572 | 379 | 379 |
| ACVR2B | -0.175069709169709 | 0.000618290021795874 | 0.00419400577969496 | 379 | 379 |
| ACVRL1 | -0.177262611073219 | 0.000526241231973831 | 0.00367923524244609 | 379 | 379 |
| ACY1 | -0.0422076941888552 | 0.412588410040119 | 0.570926302901492 | 379 | 379 |
| ACY3 | 0.069153609553035 | 0.179129906193116 | 0.318688899057613 | 379 | 379 |
| ACYP1 | 0.0411681644660539 | 0.424203370700654 | 0.58182872647379 | 379 | 379 |
| ACYP2 | -0.158247643099047 | 0.00200109504766624 | 0.0104442518044555 | 379 | 379 |
| ADAD1 | 0.0385035643499059 | 0.454830846670266 | 0.6093504072823 | 379 | 9 |
| ADAD2 | 0.0207830150042492 | 0.686720761307023 | 0.796554302983248 | 379 | 210 |
| ADAL | 0.0115231750069667 | 0.823069580906541 | 0.893573939921691 | 379 | 379 |
| ADAM10 | 0.15282031506796 | 0.00285619029145683 | 0.0138805247791681 | 379 | 379 |
| ADAM11 | 0.105008854085055 | 0.0410322941667696 | 0.107745437530567 | 379 | 367 |
| ADAM12 | 0.162222384326133 | 0.00153114488975845 | 0.00851122536420817 | 379 | 379 |
| ADAM15 | 0.0803072212738451 | 0.118577489701337 | 0.236629877797717 | 379 | 379 |
| ADAM17 | 0.0801119293807838 | 0.119478910038368 | 0.23787808296423 | 379 | 379 |
| ADAM18 | 0.0450147686796396 | 0.382176872880752 | 0.542123553833134 | 379 | 29 |
| ADAM19 | 0.148255590402084 | 0.00381950257674949 | 0.0174139105752561 | 379 | 379 |
| ADAM20 | 0.0200335488213433 | 0.697451978381063 | 0.804074961765767 | 379 | 267 |
| ADAM21P1 | 0.0387233864834969 | 0.452258136146837 | 0.607216571202565 | 379 | 194 |
| ADAM21 | 0.129457506380123 | 0.0116501633426629 | 0.0410738689115077 | 379 | 311 |
| ADAM22 | 0.144445258828812 | 0.00483914630931412 | 0.0208906146355498 | 379 | 379 |
| ADAM23 | 0.0928412059350076 | 0.0710189678162278 | 0.16223088638942 | 379 | 372 |
| ADAM28 | 0.127396469732531 | 0.0130620331580788 | 0.0449030044467974 | 379 | 379 |
| ADAM29 | 0.0274268746091331 | 0.594531597494472 | 0.727767163546141 | 379 | 87 |
| ADAM2 | 0.128527273697011 | 0.0122697853090049 | 0.042786722319196 | 379 | 33 |
| ADAM30 | 0.0413847922542661 | 0.421767344623119 | 0.579743702543308 | 379 | 10 |
| ADAM32 | -0.237297480887544 | 2.99272408054881E-06 | 0.0000684958527613696 | 379 | 369 |
| ADAM33 | -0.014177227048582 | 0.783236873568305 | 0.866678007663908 | 379 | 377 |
| ADAM3A | -0.115631931283455 | 0.0243707177201953 | 0.072392897521503 | 379 | 4 |
| ADAM5P | 0.0243827717090429 | 0.636081692248873 | 0.759878900816054 | 379 | 5 |
| ADAM6 | 0.0989487507323869 | 0.0542682440171708 | 0.132876110443623 | 379 | 379 |
| ADAM7 | -0.000634685459603333 | 0.990174144836456 | 0.994739471237637 | 379 | 3 |
| ADAM8 | 0.162817723168647 | 0.001470205573251 | 0.00824179703319784 | 379 | 379 |
| ADAM9 | 0.130583208603749 | 0.0109374363772494 | 0.0390796107088828 | 379 | 379 |
| ADAMDEC1 | 0.0654976334200412 | 0.2032816617695 | 0.348764280485044 | 379 | 379 |
| ADAMTS10 | 0.0790891259931515 | 0.124287541206891 | 0.244894501346541 | 379 | 379 |
| ADAMTS12 | 0.0993372412203016 | 0.0533245276637602 | 0.131034667804813 | 379 | 379 |
| ADAMTS13 | -0.131237834813347 | 0.0105409570355102 | 0.0380011083818356 | 379 | 379 |
| ADAMTS14 | 0.0754092542113882 | 0.142839277264174 | 0.271039951077593 | 379 | 379 |
| ADAMTS15 | 0.0457896616660401 | 0.374029207036153 | 0.534851515730048 | 379 | 369 |
| ADAMTS16 | 0.0539947117233256 | 0.294427793563508 | 0.452797199315694 | 379 | 373 |
| ADAMTS17 | -0.0054246255762297 | 0.916170986821312 | 0.952038065441694 | 379 | 378 |
| ADAMTS18 | -0.0722933760834492 | 0.160142824327961 | 0.294692521649634 | 379 | 357 |
| ADAMTS19 | -0.0153516781754945 | 0.76578282177223 | 0.85466293989079 | 379 | 254 |
| ADAMTS1 | 0.129714440781879 | 0.0114839686402455 | 0.0406106884606364 | 379 | 379 |
| ADAMTS20 | 0.151414040861698 | 0.00312633221996446 | 0.0148583210108953 | 379 | 60 |
| ADAMTS2 | 0.181499829699342 | 0.00038334409511323 | 0.00285749876612975 | 379 | 379 |
| ADAMTS3 | 0.0685841736279346 | 0.18274511326233 | 0.323091404883235 | 379 | 378 |
| ADAMTS4 | 0.220965681470432 | 0.0000141634238864176 | 0.000226660507522105 | 379 | 379 |
| ADAMTS5 | 0.10541421263621 | 0.0402538086673733 | 0.106179661867325 | 379 | 379 |
| ADAMTS6 | 0.0147071073600789 | 0.775348043648825 | 0.861492156708413 | 379 | 378 |
| ADAMTS7 | 0.114926502416973 | 0.0252596823876608 | 0.0743761482599551 | 379 | 379 |
| ADAMTS8 | -0.0229347632947683 | 0.656264904084799 | 0.773811876676581 | 379 | 376 |
| ADAMTS9 | -0.0316838775311237 | 0.538596844514955 | 0.683958163467386 | 379 | 379 |
| ADAMTSL1 | -0.0626081814955921 | 0.223977911626589 | 0.372945417511926 | 379 | 379 |
| ADAMTSL2 | -0.202672250965302 | 0.0000707077399480283 | 0.000771597725750966 | 379 | 379 |
| ADAMTSL3 | 0.00087241675154125 | 0.986494020677343 | 0.992708164851414 | 379 | 376 |
| ADAMTSL4 | 0.0394256421979267 | 0.44409433377706 | 0.599666470316776 | 379 | 379 |
| ADAMTSL5 | 0.170203764670739 | 0.000878257370172583 | 0.0055494222867374 | 379 | 379 |
| ADAP1 | 0.0626316077782157 | 0.223804295481957 | 0.372768016358985 | 379 | 379 |
| ADAP2 | 0.242105529110078 | 1.85276913695467E-06 | 0.0000472692061250472 | 379 | 379 |
| ADARB1 | 0.161917182650819 | 0.00156327608397465 | 0.00865344449833871 | 379 | 379 |
| ADARB2 | -0.128609124605018 | 0.0122141213717672 | 0.0426301000808661 | 379 | 299 |
| ADAR | 0.151959261960346 | 0.00301895668123832 | 0.0144833953727543 | 379 | 379 |
| ADAT1 | -0.0676375899480577 | 0.188873484020487 | 0.331004369909688 | 379 | 379 |
| ADAT2 | -0.121296946347964 | 0.0181605593846073 | 0.0578082471468925 | 379 | 379 |
| ADAT3 | -0.111322457754088 | 0.030250313139147 | 0.0855029520916618 | 379 | 379 |
| ADA | 0.157173901774771 | 0.00214893382194966 | 0.0110472024427321 | 379 | 379 |
| ADCK1 | -0.100407892638623 | 0.0507941434076941 | 0.126462365078825 | 379 | 379 |
| ADCK2 | -0.0694432031258752 | 0.17731179545964 | 0.316334198342067 | 379 | 379 |
| ADCK4 | 0.0404565013068109 | 0.432263627676263 | 0.588945455202703 | 379 | 379 |
| ADCK5 | -0.0568225561355034 | 0.269829695739999 | 0.425529563917338 | 379 | 379 |
| ADCY10 | 0.0642755896351974 | 0.211858915024606 | 0.358516562866595 | 379 | 281 |
| ADCY1 | -0.0230427712378205 | 0.654750532400264 | 0.772669536747556 | 379 | 379 |
| ADCY2 | -0.0306465562606025 | 0.551981344392547 | 0.695550178602624 | 379 | 376 |
| ADCY3 | -0.117521678483141 | 0.0221211940359508 | 0.0672110075612677 | 379 | 379 |
| ADCY4 | 0.0676439839996833 | 0.188831587470436 | 0.330960197680881 | 379 | 379 |
| ADCY5 | 0.0044759123153386 | 0.930791283222074 | 0.96033560015232 | 379 | 375 |
| ADCY6 | -0.04861030435629 | 0.345285284787395 | 0.506834218741817 | 379 | 379 |
| ADCY7 | 0.229306656259226 | 6.49475500794831E-06 | 0.000124182979909859 | 379 | 379 |
| ADCY8 | 0.0293965792215059 | 0.568322848814483 | 0.707643483110396 | 379 | 73 |
| ADCY9 | 0.0613034334471642 | 0.233799491298944 | 0.384967307214372 | 379 | 379 |
| ADCYAP1R1 | 0.0646584754553892 | 0.209143954321468 | 0.355530377768009 | 379 | 129 |
| ADCYAP1 | 0.0365798525715487 | 0.477692297130446 | 0.629724278139916 | 379 | 359 |
| ADC | -0.0258445570583334 | 0.615975893570258 | 0.744140012046004 | 379 | 379 |
| ADD1 | 0.0328343776570542 | 0.523944088022258 | 0.671152101382863 | 379 | 379 |
| ADD2 | 0.097206335219307 | 0.0586732192541105 | 0.14069084428232 | 379 | 346 |
| ADD3 | -0.119574453342831 | 0.0198836045600276 | 0.0619795804458776 | 379 | 379 |
| ADH1A | 0.061246698876367 | 0.234233356161763 | 0.385233824317803 | 379 | 247 |
| ADH1B | 0.0622133946610606 | 0.226918181542457 | 0.376481775886858 | 379 | 361 |
| ADH1C | -0.14713144353875 | 0.00409797519977414 | 0.0184125656608025 | 379 | 377 |
| ADH4 | -0.000402568462753133 | 0.993767560181467 | 0.996418049072748 | 379 | 333 |
| ADH5 | 0.0430454425061561 | 0.403366104506414 | 0.562482813148125 | 379 | 379 |
| ADH6 | -0.0232867052642779 | 0.65133555992212 | 0.770795671867251 | 379 | 378 |
| ADH7 | 0.0786268460529862 | 0.12650966488933 | 0.248015981355115 | 379 | 18 |
| ADHFE1 | -0.022794980892674 | 0.658226872870104 | 0.774946046933354 | 379 | 372 |
| ADI1 | 0.00423120980685869 | 0.934566286729195 | 0.962538356099231 | 379 | 379 |
| ADIG | 0.00568997216050278 | 0.912086656319102 | 0.949466023929778 | 379 | 12 |
| ADIPOQ | 0.0842704693811191 | 0.101407770886558 | 0.211743184618646 | 379 | 160 |
| ADIPOR1 | -0.11333585678964 | 0.027367298605409 | 0.0793099673703669 | 379 | 379 |
| ADIPOR2 | 0.107031798507754 | 0.0372703369745734 | 0.100202880207707 | 379 | 379 |
| ADK | -0.119348263676905 | 0.0201201057170415 | 0.062539811280373 | 379 | 379 |
| ADM2 | -0.0265308116057769 | 0.606633847118712 | 0.736398672748245 | 379 | 379 |
| ADM | 0.190104581154845 | 0.000197088046481857 | 0.00169667648626004 | 379 | 379 |
| ADNP2 | 0.264191772546184 | 1.79604296376145E-07 | 7.84403962234846E-06 | 379 | 379 |
| ADNP | -0.26496013637033 | 1.64948109748032E-07 | 7.31675865790598E-06 | 379 | 379 |
| ADORA1 | 0.018327571694734 | 0.722101909621111 | 0.822710301379405 | 379 | 378 |
| ADORA2A | 0.208766532351947 | 0.0000420265267957467 | 0.00051873624009492 | 379 | 379 |
| ADORA2B | -0.0146284573265568 | 0.776517544447432 | 0.862372010266814 | 379 | 379 |
| ADORA3 | 0.221000509627389 | 0.0000141182535485191 | 0.000226302935618461 | 379 | 379 |
| ADO | 0.0533597358697111 | 0.300150759607281 | 0.459105859869874 | 379 | 379 |
| ADPGK | 0.216974274710053 | 0.0000203557524868194 | 0.000298310318040395 | 379 | 379 |
| ADPRHL1 | -0.0243818896446842 | 0.636093907390804 | 0.759878900816054 | 379 | 379 |
| ADPRHL2 | 0.219159813783866 | 0.0000167030518531381 | 0.000256536105456253 | 379 | 379 |
| ADPRH | -0.0256740462896882 | 0.618306796218789 | 0.745502411397151 | 379 | 379 |
| ADRA1A | 0.0607761599791012 | 0.237853600794028 | 0.38950786228477 | 379 | 217 |
| ADRA1B | 0.0287000295865843 | 0.577528031434253 | 0.714674268693651 | 379 | 367 |
| ADRA1D | 0.0402190999047432 | 0.434971946251816 | 0.59177787501924 | 379 | 369 |
| ADRA2A | 0.00271446630624729 | 0.957994396039628 | 0.977024388310927 | 379 | 379 |
| ADRA2B | 0.0440184604914913 | 0.392810513175148 | 0.552660672336396 | 379 | 379 |
| ADRA2C | -0.017963402693008 | 0.727402312814966 | 0.826529115100008 | 379 | 379 |
| ADRB1 | 0.164466963525526 | 0.00131282591048069 | 0.00755389209315473 | 379 | 372 |
| ADRB2 | 0.077918140002342 | 0.129975994361907 | 0.253184400845653 | 379 | 378 |
| ADRB3 | 0.0418642540660258 | 0.416404838719068 | 0.574443410709084 | 379 | 229 |
| ADRBK1 | 0.025419353350934 | 0.621795610476322 | 0.748525491137424 | 379 | 379 |
| ADRBK2 | -0.0101428905974408 | 0.843973234079896 | 0.907254068058346 | 379 | 379 |
| ADRM1 | -0.0809050789547545 | 0.115850917200145 | 0.232640468527899 | 379 | 379 |
| ADSL | 0.0538384267550034 | 0.295829567188587 | 0.454248327903299 | 379 | 379 |
| ADSSL1 | 0.156083257091635 | 0.00230924921054089 | 0.0117102837302712 | 379 | 379 |
| ADSS | -0.138435991327511 | 0.00695133142232467 | 0.0277829065595351 | 379 | 379 |
| AEBP1 | 0.11557727656744 | 0.0244386138504886 | 0.0725402448244742 | 379 | 379 |
| AEBP2 | 0.0643701976240153 | 0.21118572218435 | 0.35773908830811 | 379 | 379 |
| AEN | 0.112762215824507 | 0.0281636033259306 | 0.0809667865371251 | 379 | 379 |
| AES | -0.0842154140459973 | 0.101631984448278 | 0.211956540905944 | 379 | 379 |
| AFAP1L1 | 0.142938197571439 | 0.00530596316413629 | 0.0224512670974166 | 379 | 379 |
| AFAP1L2 | 0.209470814689353 | 0.0000395352446104558 | 0.000498350178090348 | 379 | 379 |
| AFAP1 | -0.172115768568721 | 0.00076595724386214 | 0.00499093007929626 | 379 | 379 |
| AFARP1 | 0.0505816743708749 | 0.326052460834428 | 0.486490194403269 | 379 | 378 |
| AFF1 | 0.0419056483749787 | 0.415943753828941 | 0.574166858181582 | 379 | 379 |
| AFF2 | 0.20629732234961 | 0.0000519837311852277 | 0.000609540758096212 | 379 | 358 |
| AFF3 | 0.0275689859689517 | 0.592622378183435 | 0.72650721983193 | 379 | 377 |
| AFF4 | -0.101343521179401 | 0.0486656516064608 | 0.122408035018762 | 379 | 379 |
| AFG3L1 | 0.0107944708977971 | 0.834090815791687 | 0.900459621155699 | 379 | 379 |
| AFG3L2 | 0.251103740501121 | 7.34990894946954E-07 | 0.0000232430613477005 | 379 | 379 |
| AFMID | -0.110428407423719 | 0.0316115998214221 | 0.088380541632707 | 379 | 379 |
| AFM | 0.0680803635537679 | 0.185988348220779 | 0.327307798750476 | 379 | 28 |
| AFP | -0.0731847854161003 | 0.155038758336917 | 0.287867365887063 | 379 | 176 |
| AFTPH | -0.114518197911125 | 0.0257868826450111 | 0.07565637036992 | 379 | 379 |
| AG2 | 0.110364256003329 | 0.0317112581312182 | 0.0886216809338681 | 379 | 379 |
| AGAP11 | 0.0452458370346957 | 0.379736020705171 | 0.539711733391766 | 379 | 375 |
| AGAP1 | 0.0663190825044456 | 0.197659412348219 | 0.342048422764922 | 379 | 379 |
| AGAP2 | 0.298546745388937 | 3.05472768211788E-09 | 3.5839728095286E-07 | 379 | 379 |
| AGAP3 | 0.102645663883929 | 0.0458279406776525 | 0.117131707605564 | 379 | 379 |
| AGAP4 | -0.0625920704888465 | 0.224097368673974 | 0.373030587419052 | 379 | 379 |
| AGAP5 | -0.00902325104028797 | 0.861011063026815 | 0.918300648577037 | 379 | 378 |
| AGAP6 | -0.0646673216423774 | 0.209081526000223 | 0.355494414109602 | 379 | 379 |
| AGAP7 | 0.0443624806602263 | 0.389118746648328 | 0.548907691273694 | 379 | 377 |
| AGAP8 | -0.0863021420905672 | 0.0934046865604277 | 0.199787284263232 | 379 | 379 |
| AGA | -0.0514489821011118 | 0.317815021231794 | 0.477976049831918 | 379 | 379 |
| AGBL1 | 0.00637136312138632 | 0.901609234766349 | 0.94346992073503 | 379 | 61 |
| AGBL2 | -0.0601230074524663 | 0.242943803007679 | 0.395491767326459 | 379 | 378 |
| AGBL3 | 0.109705774072058 | 0.0327498070135314 | 0.0908963008768618 | 379 | 371 |
| AGBL4 | 0.112651378245971 | 0.0283197348226246 | 0.0812742368017079 | 379 | 269 |
| AGBL5 | -0.071705743996435 | 0.163576058679109 | 0.299099300394623 | 379 | 379 |
| AGER | 0.0461317628993119 | 0.370466475098914 | 0.531059085328315 | 379 | 379 |
| AGFG1 | -0.0832703440128153 | 0.1055424225812 | 0.217242308205132 | 379 | 379 |
| AGFG2 | -0.120321809995466 | 0.0191192827073399 | 0.0602123788947166 | 379 | 379 |
| AGGF1 | -0.0823113858596099 | 0.10963089701158 | 0.223592000200124 | 379 | 379 |
| AGK | -0.136449254476124 | 0.00781264564720141 | 0.0303089684783231 | 379 | 379 |
| AGL | 0.125547190982979 | 0.0144549621904892 | 0.0486031864190299 | 379 | 379 |
| AGMAT | 0.014855187540948 | 0.773147517485148 | 0.85973691754223 | 379 | 379 |
| AGPAT1 | 0.148730030876218 | 0.00370720288721814 | 0.017056712562553 | 379 | 379 |
| AGPAT2 | 0.0985560869878595 | 0.0552361611473001 | 0.134706866891593 | 379 | 379 |
| AGPAT3 | -0.0491930739379864 | 0.339526166908675 | 0.500692186825601 | 379 | 379 |
| AGPAT4 | 0.0649584756710176 | 0.207034324775296 | 0.353368046108683 | 379 | 379 |
| AGPAT5 | 0.0479345283561963 | 0.352040712671532 | 0.513330140524425 | 379 | 379 |
| AGPAT6 | -0.0886699419095058 | 0.0847232596915072 | 0.185891488979845 | 379 | 379 |
| AGPAT9 | 0.208599864586869 | 0.0000426374210221969 | 0.00052347664645704 | 379 | 379 |
| AGPHD1 | -0.115562447217301 | 0.0244570641016879 | 0.0725732815037815 | 379 | 379 |
| AGPS | 0.103900313065008 | 0.0432263757217213 | 0.112038245465397 | 379 | 379 |
| AGR2 | 0.257149974750573 | 0.000000387011419307 | 0.0000141580487491128 | 379 | 379 |
| AGR3 | 0.130192126572683 | 0.011180546345226 | 0.039778911346338 | 379 | 378 |
| AGRN | 0.212579067281006 | 0.0000301156906741673 | 0.000400223803409778 | 379 | 379 |
| AGRP | 0.103561446742159 | 0.0439164702761355 | 0.113367500668561 | 379 | 136 |
| AGTPBP1 | -0.0977196732198763 | 0.0573457485648825 | 0.138327433399573 | 379 | 379 |
| AGTR1 | -0.02735670517693 | 0.595475331993682 | 0.728427718105418 | 379 | 336 |
| AGTR2 | 0.031090704390319 | 0.546230652146147 | 0.690466745553602 | 379 | 61 |
| AGTRAP | 0.193814918914662 | 0.000146592065885298 | 0.00134441604180097 | 379 | 379 |
| AGT | -0.098659692264574 | 0.0549793923765573 | 0.134186415715893 | 379 | 379 |
| AGXT2L1 | 0.0270690682975773 | 0.599350960450189 | 0.731407784334065 | 379 | 56 |
| AGXT2L2 | -0.104652893314432 | 0.041726352251857 | 0.109134693635381 | 379 | 379 |
| AGXT2 | 0.0136918317481157 | 0.790482966451275 | 0.871708606204635 | 379 | 66 |
| AGXT | -0.200440578530546 | 0.0000852221586228521 | 0.000888892667634882 | 379 | 358 |
| AHCTF1 | 0.00975668903700374 | 0.849842366918963 | 0.910767217779849 | 379 | 379 |
| AHCYL1 | 0.132675969340674 | 0.00971435083914361 | 0.0357490995617186 | 379 | 379 |
| AHCYL2 | 0.0998672415212828 | 0.0520591120489675 | 0.128688727432866 | 379 | 379 |
| AHCY | -0.153474036203706 | 0.00273799058732575 | 0.0134218849634558 | 379 | 379 |
| AHDC1 | 0.202663374142632 | 0.000070760555870452 | 0.000771749340923719 | 379 | 379 |
| AHI1 | -0.0746658455024109 | 0.146831871289443 | 0.276642183953543 | 379 | 379 |
| AHNAK2 | 0.192965827325383 | 0.000156942485123371 | 0.00142549500459285 | 379 | 379 |
| AHNAK | 0.196682579735092 | 0.000116173279342344 | 0.00112200866186069 | 379 | 379 |
| AHRR | 0.109013553063094 | 0.0338727071623296 | 0.093178140623567 | 379 | 379 |
| AHR | 0.148310156101387 | 0.00380643205844981 | 0.0173742943957051 | 379 | 379 |
| AHSA1 | 0.0356996351298295 | 0.488357915461267 | 0.639955108569559 | 379 | 379 |
| AHSA2 | -0.101432194669498 | 0.0484678531098683 | 0.122049859215452 | 379 | 379 |
| AHSG | 0.0184056577608636 | 0.720967101184051 | 0.822043454990073 | 379 | 271 |
| AHSP | 0.107770965440458 | 0.0359707175037371 | 0.0975020350873684 | 379 | 106 |
| AICDA | 0.128530926318462 | 0.012267296525293 | 0.0427855682503976 | 379 | 193 |
| AIDA | 0.0743294967053535 | 0.148665979342948 | 0.27924867737893 | 379 | 379 |
| AIF1L | 0.184220187423315 | 0.00031162130232686 | 0.00242592351100784 | 379 | 379 |
| AIF1 | 0.202377818142241 | 0.0000724795424188098 | 0.000786603375522803 | 379 | 379 |
| AIFM1 | 0.0271576226270995 | 0.598156564257622 | 0.730795451177404 | 379 | 379 |
| AIFM2 | -0.0444209275843672 | 0.388493634712926 | 0.548142872631317 | 379 | 379 |
| AIFM3 | -0.243356863002047 | 1.63269916048666E-06 | 0.0000435123104222171 | 379 | 379 |
| AIG1 | -0.0483405557370038 | 0.347971906591944 | 0.509676781377879 | 379 | 379 |
| AIM1L | 0.185163776254135 | 0.0002898178810031 | 0.00229768450401018 | 379 | 379 |
| AIM1 | 0.033239766298289 | 0.518829999228254 | 0.666754891742681 | 379 | 379 |
| AIM2 | 0.346248623424851 | 4.08737225947064E-12 | 2.05691100680877E-09 | 379 | 373 |
| AIMP1 | -0.000738683256106178 | 0.988564209583757 | 0.993827062192706 | 379 | 379 |
| AIMP2 | 0.0660453068960973 | 0.199520484955863 | 0.344427318100719 | 379 | 379 |
| AIPL1 | 0.144687477807607 | 0.00476767154581044 | 0.0206449861127603 | 379 | 59 |
| AIP | 0.0198931180386214 | 0.699469534912146 | 0.805785009518592 | 379 | 379 |
| AIRE | -0.113020763765287 | 0.0278022688664412 | 0.0802820447886262 | 379 | 255 |
| AJAP1 | 0.181475606135671 | 0.00038404678394315 | 0.00286166089140352 | 379 | 361 |
| AK1 | 0.00916998063674463 | 0.858774393481896 | 0.917197881710694 | 379 | 379 |
| AK2 | 0.151338949494352 | 0.0031413893090648 | 0.0149048737066611 | 379 | 379 |
| AK3L1 | -0.0922350070517031 | 0.0728902065871324 | 0.165457833772788 | 379 | 379 |
| AK3 | -0.281159110948531 | 2.57352472439502E-08 | 1.82895513388188E-06 | 379 | 379 |
| AK5 | 0.0398409384433171 | 0.439306166629576 | 0.595758659769206 | 379 | 364 |
| AK7 | 0.13772874053904 | 0.00724768939339018 | 0.0286783447000879 | 379 | 379 |
| AKAP10 | 0.0435769826692985 | 0.397578977308362 | 0.557037589179635 | 379 | 379 |
| AKAP11 | -0.151754904957514 | 0.00305880375338054 | 0.0146462112586403 | 379 | 379 |
| AKAP12 | 0.17345623992238 | 0.000695311904272762 | 0.00461709458738122 | 379 | 379 |
| AKAP13 | 0.0743344202764718 | 0.14863900605356 | 0.279232713298952 | 379 | 379 |
| AKAP14 | 0.142710299716337 | 0.00537997711337129 | 0.0226939464118379 | 379 | 194 |
| AKAP1 | -0.238053277764041 | 0.0000027772865501896 | 0.0000644824797624817 | 379 | 379 |
| AKAP2 | 0.109078881712429 | 0.0337653501422351 | 0.0929731096542478 | 379 | 378 |
| AKAP3 | 0.0838046054572958 | 0.10331744258678 | 0.214286427992748 | 379 | 377 |
| AKAP4 | 0.0594939596845229 | 0.247917791072949 | 0.401545005831926 | 379 | 238 |
| AKAP5 | -0.00713744794089211 | 0.889849958560018 | 0.936316332961581 | 379 | 379 |
| AKAP6 | -0.0435936937661356 | 0.397397847849622 | 0.556980598512921 | 379 | 379 |
| AKAP7 | 0.00827388692820814 | 0.872450928167447 | 0.925523353683812 | 379 | 379 |
| AKAP8L | -0.12247261099841 | 0.0170606537524787 | 0.0550046573340078 | 379 | 379 |
| AKAP8 | -0.020923732148814 | 0.684712820344325 | 0.795248699729807 | 379 | 379 |
| AKAP9 | -0.0262526585918667 | 0.610412772263314 | 0.739401579109108 | 379 | 379 |
| AKD1 | -0.204846913749965 | 0.0000588304461213629 | 0.000672945014607394 | 379 | 379 |
| AKIRIN1 | 0.17527153505779 | 0.000609232717020952 | 0.00414688167287725 | 379 | 379 |
| AKIRIN2 | -0.0349416185917664 | 0.497644326947047 | 0.648419932649066 | 379 | 379 |
| AKNAD1 | -0.196144381514633 | 0.000121387115531285 | 0.00115770261027144 | 379 | 352 |
| AKNA | -0.026628217246942 | 0.605312982148685 | 0.735774914848356 | 379 | 379 |
| AKR1A1 | 0.0963281885215371 | 0.0610029518083233 | 0.144806240686632 | 379 | 379 |
| AKR1B10 | 0.0309649957239984 | 0.547855284233691 | 0.691990990940606 | 379 | 373 |
| AKR1B15 | 0.0366303535163796 | 0.477084248950536 | 0.629173693926919 | 379 | 212 |
| AKR1B1 | 0.0241718489726544 | 0.639005425724203 | 0.762163112443425 | 379 | 379 |
| AKR1C1 | 0.000523073940774872 | 0.991901988280268 | 0.995567330985632 | 379 | 379 |
| AKR1C2 | -0.00623121411020422 | 0.90376290545889 | 0.944639477566624 | 379 | 378 |
| AKR1C3 | -0.107607033786418 | 0.0362555752405634 | 0.0980596843363648 | 379 | 379 |
| AKR1C4 | 0.0616761826896855 | 0.230963081249715 | 0.381341991424711 | 379 | 341 |
| AKR1CL1 | 0.0079327338675463 | 0.877667966945345 | 0.928573739319796 | 379 | 163 |
| AKR1D1 | 0.16713279017488 | 0.00109085274574542 | 0.00660292792411868 | 379 | 95 |
| AKR1E2 | -0.0255435596908848 | 0.620093153129603 | 0.747055109623642 | 379 | 369 |
| AKR7A2 | 0.188587275500055 | 0.000222094899739587 | 0.00186834860926455 | 379 | 379 |
| AKR7A3 | 0.00461858345384097 | 0.928591018450347 | 0.959613421266142 | 379 | 379 |
| AKR7L | 0.0294395148069265 | 0.567757732098351 | 0.707529401800396 | 379 | 379 |
| AKT1S1 | 0.123145305948019 | 0.0164578634681062 | 0.0535576098548514 | 379 | 379 |
| AKT1 | 0.142166847595686 | 0.00556023357409259 | 0.0233527454579767 | 379 | 379 |
| AKT2 | 0.014526248649146 | 0.778038108609388 | 0.863577515237131 | 379 | 379 |
| AKT3 | 0.103748329945744 | 0.0435347492902451 | 0.112631394692978 | 379 | 379 |
| AKTIP | -0.209007436475148 | 0.0000411581324932341 | 0.000511010301237223 | 379 | 379 |
| ALAD | -0.286769259515797 | 1.31439699218097E-08 | 1.07250467329071E-06 | 379 | 379 |
| ALAS1 | 0.0901856945579408 | 0.079515631887126 | 0.176747405941982 | 379 | 379 |
| ALAS2 | 0.0562921635606354 | 0.27433298035858 | 0.430576611616395 | 379 | 232 |
| ALB | -0.0175551039077853 | 0.733360490057474 | 0.830918388391977 | 379 | 181 |
| ALCAM | -0.100095265770698 | 0.0515224361291525 | 0.12766644133577 | 379 | 379 |
| ALDH16A1 | 0.203741357984192 | 0.0000646113703483057 | 0.000721754507755608 | 379 | 379 |
| ALDH18A1 | 0.100530069680336 | 0.0505118646698137 | 0.125854392142883 | 379 | 379 |
| ALDH1A1 | 0.106005769633564 | 0.0391401440226601 | 0.104013184257502 | 379 | 379 |
| ALDH1A2 | 0.0984210401925548 | 0.0555723464035827 | 0.1352840373837 | 379 | 361 |
| ALDH1A3 | 0.00973933778499764 | 0.85010625233276 | 0.910982368872829 | 379 | 379 |
| ALDH1B1 | -0.215393800654669 | 0.00002345624318191 | 0.000328918238904463 | 379 | 379 |
| ALDH1L1 | 0.0348060445651652 | 0.499315051148814 | 0.649889643834757 | 379 | 373 |
| ALDH1L2 | 0.123440821259401 | 0.0161990031102459 | 0.0529847960524505 | 379 | 379 |
| ALDH2 | -0.140199003643095 | 0.00625923461975578 | 0.0256315786948611 | 379 | 379 |
| ALDH3A1 | -0.0379671484496351 | 0.461143182599228 | 0.614860642758719 | 379 | 379 |
| ALDH3A2 | 0.0790470184020936 | 0.124488683314336 | 0.245193048559629 | 379 | 379 |
| ALDH3B1 | 0.0104453542800185 | 0.839382672155197 | 0.904182084179565 | 379 | 379 |
| ALDH3B2 | 0.0880428679602022 | 0.0869565152615459 | 0.189428014129415 | 379 | 374 |
| ALDH4A1 | -0.0899418926545414 | 0.0803353170363677 | 0.178215335220083 | 379 | 379 |
| ALDH5A1 | -0.0721749259237823 | 0.160830465378525 | 0.295683492584645 | 379 | 379 |
| ALDH6A1 | -0.0289487829468619 | 0.574232637886835 | 0.712107370318354 | 379 | 379 |
| ALDH7A1 | -0.212676071683574 | 0.0000298590661447293 | 0.000397880083009202 | 379 | 379 |
| ALDH8A1 | 0.0280337383508617 | 0.586398224704902 | 0.721799419258202 | 379 | 373 |
| ALDH9A1 | -0.117451663464636 | 0.0222012131639352 | 0.0674170514107946 | 379 | 379 |
| ALDOA | 0.17644049005536 | 0.000559147780732103 | 0.00386299031231921 | 379 | 379 |
| ALDOB | -0.0860470977666398 | 0.0943807468604756 | 0.201310396810403 | 379 | 379 |
| ALDOC | 0.0682930116697095 | 0.184614312568193 | 0.325641187581366 | 379 | 379 |
| ALG10B | -0.132847096352072 | 0.00961992632249897 | 0.0354938405512671 | 379 | 379 |
| ALG10 | 0.0968001935517347 | 0.0597414054439498 | 0.142623196756489 | 379 | 379 |
| ALG11 | -0.103716468118951 | 0.043599631036919 | 0.112755117281861 | 379 | 379 |
| ALG12 | 0.161482640124511 | 0.00161009185653007 | 0.00885572852462641 | 379 | 379 |
| ALG13 | 0.0783633879632146 | 0.127789788483167 | 0.249889903820082 | 379 | 379 |
| ALG14 | -0.0116300060310415 | 0.821456685295255 | 0.892843300641695 | 379 | 379 |
| ALG1L2 | 0.0620942398595351 | 0.227810977738755 | 0.37745768083931 | 379 | 264 |
| ALG1L | -0.109377169687809 | 0.0332788375136943 | 0.0919681538929795 | 379 | 379 |
| ALG1 | -0.100876205402183 | 0.0497192502344681 | 0.12441106684112 | 379 | 379 |
| ALG2 | -0.0603710021132427 | 0.241002201367255 | 0.393145317383938 | 379 | 379 |
| ALG3 | -0.0118200222073452 | 0.818589751797682 | 0.89073634061269 | 379 | 379 |
| ALG5 | -0.157946915304807 | 0.00204153453485957 | 0.0106161937469697 | 379 | 379 |
| ALG6 | 0.108000824731521 | 0.0355745025370933 | 0.096586503670476 | 379 | 379 |
| ALG8 | 0.0358394037814437 | 0.486655838637306 | 0.638484216800139 | 379 | 379 |
| ALG9 | 0.0383593019852453 | 0.456523688663958 | 0.610834179015383 | 379 | 379 |
| ALKBH1 | 0.0995254275210758 | 0.052872319784505 | 0.13018159154193 | 379 | 379 |
| ALKBH2 | 0.100081797670043 | 0.0515540058712548 | 0.12771380422941 | 379 | 379 |
| ALKBH3 | -0.0696444848067651 | 0.176056207666315 | 0.315029553795479 | 379 | 379 |
| ALKBH4 | 0.039884873081722 | 0.438801354141224 | 0.595437108694222 | 379 | 379 |
| ALKBH5 | 0.267646182517104 | 1.22234009628145E-07 | 5.83042464112369E-06 | 379 | 379 |
| ALKBH6 | -0.0589679518789987 | 0.252131175576897 | 0.406212476585578 | 379 | 379 |
| ALKBH7 | -0.1084085237453 | 0.0348808602588438 | 0.0953166616885825 | 379 | 379 |
| ALKBH8 | 0.0652069608162718 | 0.205298635465358 | 0.351312793993882 | 379 | 379 |
| ALK | 0.0887070043516555 | 0.0845927257831394 | 0.185665770071739 | 379 | 244 |
| ALLC | 0.0226619538906002 | 0.66009620339393 | 0.776298192223894 | 379 | 116 |
| ALMS1P | 0.0329144876807896 | 0.522931441095389 | 0.67041798875206 | 379 | 244 |
| ALMS1 | 0.0659371231085834 | 0.200259400806996 | 0.345207569730829 | 379 | 379 |
| ALOX12B | 0.209388521598554 | 0.0000398189704015965 | 0.000500019344599656 | 379 | 324 |
| ALOX12P2 | 0.128486835484356 | 0.0122973684470045 | 0.0428678307959223 | 379 | 222 |
| ALOX12 | 0.101752643427228 | 0.0477586455895981 | 0.12081633385437 | 379 | 374 |
| ALOX15B | 0.201890157349846 | 0.0000755064445686909 | 0.000810580283112075 | 379 | 376 |
| ALOX15 | -0.127090870697398 | 0.0132837278989623 | 0.0454120270311423 | 379 | 371 |
| ALOX5AP | 0.15916948755144 | 0.00188165227965584 | 0.00998111326939967 | 379 | 379 |
| ALOX5 | 0.171569487811253 | 0.000796606226588032 | 0.00513662057261382 | 379 | 379 |
| ALOXE3 | 0.31210127238153 | 5.24038075459986E-10 | 8.58729500844677E-08 | 379 | 332 |
| ALPI | 0.125300520021497 | 0.0146502925695235 | 0.0490602940497404 | 379 | 371 |
| ALPK1 | 0.0807796947648246 | 0.116418635027329 | 0.23347311574005 | 379 | 379 |
| ALPK2 | 0.191184116675629 | 0.000180928083103003 | 0.00159371036506723 | 379 | 379 |
| ALPK3 | -0.0128971273817877 | 0.802385264760525 | 0.879279690702021 | 379 | 379 |
| ALPL | 0.183913703757151 | 0.000319026048830787 | 0.00246902751608776 | 379 | 379 |
| ALPPL2 | 0.213121820265535 | 0.0000287062237807254 | 0.000385889494999474 | 379 | 352 |
| ALPP | 0.244906126983083 | 1.39477401282486E-06 | 0.0000383041262136998 | 379 | 312 |
| ALS2CL | -0.223526574971761 | 0.0000111833018398722 | 0.000188717028834881 | 379 | 379 |
| ALS2CR11 | -0.0989147506683234 | 0.0543514930189868 | 0.133013677942325 | 379 | 317 |
| ALS2CR12 | 0.0072019486320771 | 0.888860947630501 | 0.935474249979701 | 379 | 303 |
| ALS2CR4 | 0.0098397266019582 | 0.848579727829777 | 0.910259206133489 | 379 | 379 |
| ALS2CR8 | -0.255602800360218 | 4.56750543107946E-07 | 0.0000162593353119288 | 379 | 379 |
| ALS2 | 0.218635154630526 | 0.0000175183906361099 | 0.000266376264979131 | 379 | 379 |
| ALX1 | 0.0650038446358941 | 0.206716629780557 | 0.352947329311021 | 379 | 133 |
| ALX3 | -0.0124008189077549 | 0.809841906338137 | 0.8847492714253 | 379 | 378 |
| ALX4 | 0.0607016916527907 | 0.238430135020368 | 0.390215165368812 | 379 | 47 |
| AMAC1L2 | 0.0291159295220131 | 0.572023334083805 | 0.710667218964216 | 379 | 222 |
| AMAC1L3 | 0.0628465651458368 | 0.222215683983033 | 0.37078958024199 | 379 | 153 |
| AMAC1 | 0.0134807591940985 | 0.793639583121575 | 0.873801191300715 | 379 | 31 |
| AMACR | -0.274943997458697 | 5.32493055081358E-08 | 3.06439303956849E-06 | 379 | 379 |
| AMBN | 0.0166466644730074 | 0.746674129053218 | 0.840241465997004 | 379 | 65 |
| AMBP | -0.0596531531130618 | 0.246652363312622 | 0.40023753631856 | 379 | 365 |
| AMBRA1 | -0.0695437482688681 | 0.176683770661564 | 0.315753565090354 | 379 | 379 |
| AMD1 | 0.075028872876881 | 0.144871681613257 | 0.27375542771635 | 379 | 379 |
| AMDHD1 | -0.0606292979599006 | 0.23899154852408 | 0.39085486837145 | 379 | 363 |
| AMDHD2 | 0.0530399292685377 | 0.303060926225661 | 0.461989086276805 | 379 | 379 |
| AMELX | -0.149844868602955 | 0.00345499647426268 | 0.0161295952455281 | 379 | 308 |
| AMELY | -0.0240464863350416 | 0.640745811587446 | 0.763595862012014 | 379 | 36 |
| AMFR | -0.0467789771695197 | 0.363783871274668 | 0.524894964316265 | 379 | 379 |
| AMHR2 | 0.0979951784414125 | 0.0566436236478367 | 0.137033890884493 | 379 | 160 |
| AMH | 0.219671483837107 | 0.0000159426871708989 | 0.000247542365876729 | 379 | 364 |
| AMICA1 | 0.101531143270155 | 0.0482479289015891 | 0.121650551152175 | 379 | 379 |
| AMIGO1 | -0.0561313038311483 | 0.275708804393312 | 0.431949602837436 | 379 | 379 |
| AMIGO2 | 0.119255076894291 | 0.0202182495852482 | 0.0626974433494372 | 379 | 379 |
| AMIGO3 | 0.0796445821834509 | 0.121657780543491 | 0.240957993468818 | 379 | 379 |
| AMMECR1L | -0.173590775382621 | 0.000688565196207698 | 0.00458710373814037 | 379 | 379 |
| AMMECR1 | 0.173506688999412 | 0.0006927748363335 | 0.00460641832824301 | 379 | 379 |
| AMN1 | 0.170570249082338 | 0.000855619674427361 | 0.00543924533040682 | 379 | 379 |
| AMN | 0.0429273914749907 | 0.404658168264635 | 0.563769123127543 | 379 | 379 |
| AMOTL1 | 0.0968202861460778 | 0.0596881850841188 | 0.142572862769294 | 379 | 379 |
| AMOTL2 | -0.0929681233009706 | 0.070632203391301 | 0.16164535189782 | 379 | 379 |
| AMOT | -0.126701386059147 | 0.0135710745059352 | 0.0461662474490792 | 379 | 379 |
| AMPD1 | 0.0142060590826894 | 0.782807044184308 | 0.866445130740563 | 379 | 284 |
| AMPD2 | 0.0633422083775395 | 0.218583397342427 | 0.366506955887624 | 379 | 379 |
| AMPD3 | -0.0137354401273904 | 0.789831222272767 | 0.871592468567699 | 379 | 379 |
| AMPH | 0.101949981952351 | 0.0473262397366165 | 0.119967358923246 | 379 | 376 |
| AMTN | 0.0741023273214289 | 0.149914556513458 | 0.280875538745994 | 379 | 21 |
| AMT | -0.202759329569063 | 0.0000701915994277708 | 0.000768503055468713 | 379 | 379 |
| AMY1A | -0.133277205675002 | 0.00938618957427167 | 0.0348454160042424 | 379 | 287 |
| AMY2A | -0.122940171184851 | 0.0166396752387502 | 0.053954453088134 | 379 | 219 |
| AMY2B | -0.208174310503982 | 0.0000442355074236759 | 0.000537439731125396 | 379 | 379 |
| AMZ1 | 0.200514682687677 | 0.0000846982018496808 | 0.000885290430298087 | 379 | 365 |
| AMZ2P1 | -0.12005221607415 | 0.0193919946377283 | 0.0608489428195721 | 379 | 379 |
| AMZ2 | -0.164686613768535 | 0.00129307633689665 | 0.00746408081746339 | 379 | 379 |
| ANAPC10 | 0.0630926383755796 | 0.220407008043742 | 0.368795793712347 | 379 | 379 |
| ANAPC11 | 0.0239385383330504 | 0.642246011897685 | 0.764878006120926 | 379 | 379 |
| ANAPC13 | -0.119334454552536 | 0.0201346232337813 | 0.0625653204010994 | 379 | 379 |
| ANAPC16 | -0.0636792739981733 | 0.216137629126061 | 0.363646746738357 | 379 | 379 |
| ANAPC1 | 0.00586281002430154 | 0.909427497168589 | 0.947862090720079 | 379 | 379 |
| ANAPC2 | 0.0110640811813945 | 0.830009206966592 | 0.897644952314475 | 379 | 379 |
| ANAPC4 | -0.113555019814584 | 0.027068214719772 | 0.0786616681025411 | 379 | 379 |
| ANAPC5 | 0.0308985590666591 | 0.548714857341629 | 0.692767793146757 | 379 | 379 |
| ANAPC7 | -0.0407630356544741 | 0.428781057472483 | 0.585891448388421 | 379 | 379 |
| ANGEL1 | -0.194926128443543 | 0.000134011733697454 | 0.00125633565475645 | 379 | 379 |
| ANGEL2 | -0.235962462992214 | 3.41283989915536E-06 | 0.0000759481363865909 | 379 | 379 |
| ANGPT1 | 0.0351382116548891 | 0.495226920374173 | 0.646308127241434 | 379 | 378 |
| ANGPT2 | 0.14636905169136 | 0.0042971434366881 | 0.0190825890397876 | 379 | 379 |
| ANGPT4 | 0.133892634368811 | 0.00906051990113667 | 0.0338646538359544 | 379 | 159 |
| ANGPTL1 | 0.0676968305940905 | 0.188485575380955 | 0.330558286631308 | 379 | 368 |
| ANGPTL2 | 0.140765287804133 | 0.00605042147154539 | 0.0249257753870355 | 379 | 379 |
| ANGPTL3 | -0.00535178512098562 | 0.917292569209433 | 0.952529254823546 | 379 | 186 |
| ANGPTL4 | 0.265587280388426 | 0.0000001538460039824 | 6.98796742150672E-06 | 379 | 379 |
| ANGPTL5 | 0.0605885861994543 | 0.239307676214654 | 0.391146039401876 | 379 | 173 |
| ANGPTL6 | 0.201014909903554 | 0.000081239847675633 | 0.0008577335994209 | 379 | 370 |
| ANGPTL7 | -0.00899252485957045 | 0.86147957817291 | 0.918454515324899 | 379 | 232 |
| ANG | 0.0094381867232034 | 0.854688928343022 | 0.914608023702598 | 379 | 379 |
| ANK1 | 0.18560738895459 | 0.000280067005450164 | 0.00224878899482816 | 379 | 379 |
| ANK2 | 0.073412609708583 | 0.153754230304702 | 0.286391627851726 | 379 | 379 |
| ANK3 | -0.0676907618974831 | 0.188525286368192 | 0.33056945331699 | 379 | 379 |
| ANKAR | -0.135003982427822 | 0.00849779560993727 | 0.0323095477188564 | 379 | 378 |
| ANKDD1A | 0.0521423453105748 | 0.311328232546561 | 0.470863172763784 | 379 | 379 |
| ANKFN1 | -0.0763447190986802 | 0.137933782503813 | 0.264318291122272 | 379 | 264 |
| ANKFY1 | 0.148080302777323 | 0.00386176578081534 | 0.0175783039260805 | 379 | 379 |
| ANKHD1-EIF4EBP3 | 0.0993276292825782 | 0.0533477110620144 | 0.131059151894142 | 379 | 379 |
| ANKHD1 | -0.238128683657456 | 2.75662476313936E-06 | 0.0000642283851980343 | 379 | 379 |
| ANKH | -0.344570291729365 | 5.26311660786968E-12 | 2.2260333825478E-09 | 379 | 379 |
| ANKIB1 | -0.0413083486302015 | 0.422626035305763 | 0.580360760997484 | 379 | 379 |
| ANKK1 | -0.185040878428249 | 0.000292574757619045 | 0.00231583724314189 | 379 | 358 |
| ANKLE1 | -0.0446887572134079 | 0.385636894892206 | 0.545276214213981 | 379 | 374 |
| ANKLE2 | 0.213620093224043 | 0.0000274674570280233 | 0.000373030642432635 | 379 | 379 |
| ANKMY1 | -0.0677071874886942 | 0.188417818430844 | 0.330530006782339 | 379 | 379 |
| ANKMY2 | 0.197521149530964 | 0.000108466929842052 | 0.00106786607989484 | 379 | 379 |
| ANKRA2 | -0.291523837771691 | 7.35114276955874E-09 | 6.90798383103368E-07 | 379 | 379 |
| ANKRD10 | -0.187049282834782 | 0.000250447318834431 | 0.00206395238480844 | 379 | 379 |
| ANKRD11 | 0.0989359241932599 | 0.0542996372832888 | 0.132903741273059 | 379 | 379 |
| ANKRD12 | 0.124150893748935 | 0.0155915275505547 | 0.0514818997955702 | 379 | 379 |
| ANKRD13A | -0.0368090045951167 | 0.474936621254778 | 0.62713394554074 | 379 | 379 |
| ANKRD13B | 0.0220630769234899 | 0.668537834985676 | 0.783021335701812 | 379 | 379 |
| ANKRD13C | 0.027340977511882 | 0.595686952491578 | 0.728596687064524 | 379 | 379 |
| ANKRD13D | 0.0801630192985249 | 0.1192425764133 | 0.23755066865497 | 379 | 379 |
| ANKRD16 | -0.199515625899949 | 0.0000920240434514245 | 0.000943948646432925 | 379 | 379 |
| ANKRD17 | -0.0212542611005995 | 0.680005130629285 | 0.791542898327901 | 379 | 379 |
| ANKRD19 | -0.0702630437433032 | 0.172238992047181 | 0.309989537470409 | 379 | 379 |
| ANKRD1 | 0.140115566023864 | 0.00629054181739845 | 0.0257438314046185 | 379 | 271 |
| ANKRD20A3 | -0.0700366830833335 | 0.173628677186951 | 0.311760053916305 | 379 | 367 |
| ANKRD20A4 | -0.0058413933906144 | 0.90975694386175 | 0.948055956424595 | 379 | 219 |
| ANKRD20B | -0.0516016754010188 | 0.316378971561624 | 0.476249791081376 | 379 | 349 |
| ANKRD22 | -0.0775167553556619 | 0.131971508891635 | 0.255990127010697 | 379 | 379 |
| ANKRD23 | -0.0921207837873479 | 0.0732472557071402 | 0.16600143858283 | 379 | 379 |
| ANKRD24 | -0.00106760777211227 | 0.983472638243314 | 0.990734124766019 | 379 | 379 |
| ANKRD26P1 | 0.0882488281435468 | 0.086217848940957 | 0.188294692014682 | 379 | 101 |
| ANKRD26 | -0.0601455853111379 | 0.242766584423822 | 0.395268175066147 | 379 | 379 |
| ANKRD27 | -0.184818136365722 | 0.000297633822694279 | 0.00234931665461074 | 379 | 379 |
| ANKRD28 | -0.149293912140986 | 0.00357762596023305 | 0.0165676858417648 | 379 | 379 |
| ANKRD29 | 0.0410214637696577 | 0.425857694777565 | 0.583369207073226 | 379 | 378 |
| ANKRD2 | 0.0254245145134438 | 0.621724827800865 | 0.748525491137424 | 379 | 202 |
| ANKRD30A | 0.0173292520979975 | 0.736663187289142 | 0.833041957201387 | 379 | 21 |
| ANKRD30B | 0.110735950135184 | 0.0311375440859117 | 0.0873384105439888 | 379 | 218 |
| ANKRD31 | -0.0484262444757434 | 0.347117039346371 | 0.508844939831424 | 379 | 254 |
| ANKRD32 | 0.155426841431779 | 0.0024109214399531 | 0.0121237003072255 | 379 | 379 |
| ANKRD33 | 0.112005686340963 | 0.0292441344595124 | 0.0832284624749838 | 379 | 280 |
| ANKRD34A | 0.231837652228738 | 5.09649285617195E-06 | 0.000102905560440099 | 379 | 378 |
| ANKRD34B | -0.0612864933627019 | 0.233928977332583 | 0.385023204760807 | 379 | 163 |
| ANKRD34C | 0.0968715125055984 | 0.0595526762311434 | 0.142283463587313 | 379 | 111 |
| ANKRD35 | 0.0452196871525133 | 0.38001177060369 | 0.539967437154733 | 379 | 377 |
| ANKRD36BP1 | 0.0124936934820714 | 0.808445209266308 | 0.883917927175758 | 379 | 379 |
| ANKRD36B | -0.0311786787915701 | 0.545095105921925 | 0.689558872031385 | 379 | 378 |
| ANKRD36 | -0.0321120140994667 | 0.533120275239655 | 0.679351466417215 | 379 | 379 |
| ANKRD37 | 0.160458271254594 | 0.0017255829235768 | 0.00933811632332991 | 379 | 379 |
| ANKRD39 | 0.0851358977159043 | 0.0979346090368621 | 0.206689454814572 | 379 | 379 |
| ANKRD40 | 0.158947815719597 | 0.00190976295060958 | 0.0100902639083713 | 379 | 379 |
| ANKRD42 | 0.140213605529859 | 0.00625377013261666 | 0.0256144916731095 | 379 | 379 |
| ANKRD43 | -0.316624477023414 | 2.85148226327486E-10 | 5.33388587888812E-08 | 379 | 379 |
| ANKRD44 | -0.0087964929740752 | 0.864469823509103 | 0.920553580050403 | 379 | 379 |
| ANKRD45 | 0.11330220456427 | 0.0274134731740207 | 0.0793861883533735 | 379 | 357 |
| ANKRD46 | -0.160223816041069 | 0.00175306152509691 | 0.00944664140558033 | 379 | 379 |
| ANKRD49 | -0.137942728891123 | 0.00715685245424994 | 0.0284266968074655 | 379 | 379 |
| ANKRD50 | -0.0985249158337753 | 0.0553136083052692 | 0.134852849560664 | 379 | 379 |
| ANKRD52 | 0.134437766852385 | 0.00878048266204446 | 0.0330946503634173 | 379 | 379 |
| ANKRD53 | 0.0579911499853507 | 0.26008679542451 | 0.414283497724709 | 379 | 348 |
| ANKRD54 | 0.0163170946995092 | 0.751523057096613 | 0.843830294813503 | 379 | 379 |
| ANKRD55 | 0.077262732862887 | 0.133246565728206 | 0.257807660544386 | 379 | 308 |
| ANKRD56 | -0.110815704008173 | 0.0310156059047429 | 0.087045638199468 | 379 | 379 |
| ANKRD57 | 0.0181104839428808 | 0.725259995411076 | 0.824993126556756 | 379 | 379 |
| ANKRD58 | 0.238821587580301 | 2.57351828230817E-06 | 0.0000607472863114361 | 379 | 379 |
| ANKRD5 | 0.108086112362995 | 0.0354284360822745 | 0.0963743148389728 | 379 | 379 |
| ANKRD6 | -0.0630630182341878 | 0.220624162150652 | 0.36903457795876 | 379 | 379 |
| ANKRD7 | -0.0732671228906609 | 0.154573586503553 | 0.287415540568897 | 379 | 104 |
| ANKRD9 | -0.142484648670516 | 0.00545417488997439 | 0.0229705564397647 | 379 | 379 |
| ANKS1A | -0.000283552347932052 | 0.995610108275311 | 0.997219500246659 | 379 | 379 |
| ANKS1B | 0.0505496971563624 | 0.326358790282822 | 0.486837352823337 | 379 | 314 |
| ANKS3 | -0.0585790678085257 | 0.255277980228619 | 0.409651326640746 | 379 | 379 |
| ANKS4B | -0.0907486290017342 | 0.0776488526056792 | 0.173361271193042 | 379 | 378 |
| ANKS6 | 0.0461186153183341 | 0.370603008392471 | 0.53117799988477 | 379 | 379 |
| ANKZF1 | -0.171933717127125 | 0.000776047921375176 | 0.00504176873690268 | 379 | 379 |
| ANLN | 0.296473673233854 | 3.96850105013631E-09 | 4.35246114025328E-07 | 379 | 379 |
| ANO10 | -0.347010341604289 | 3.64245908434389E-12 | 1.93878050505414E-09 | 379 | 379 |
| ANO1 | 0.233501934199722 | 4.33896166089132E-06 | 0.0000909439025498448 | 379 | 379 |
| ANO2 | 0.129082525923529 | 0.0118965287480179 | 0.0417864255120813 | 379 | 378 |
| ANO3 | 0.0612950369586517 | 0.233863665762715 | 0.38500587954957 | 379 | 253 |
| ANO4 | 0.0589815523107368 | 0.252021612114669 | 0.406115209975968 | 379 | 348 |
| ANO5 | -0.0631814108751649 | 0.219757105020432 | 0.36792568423078 | 379 | 364 |
| ANO6 | 0.0743459464066988 | 0.148575875864108 | 0.279160974769526 | 379 | 379 |
| ANO7 | 0.0231641592251855 | 0.65305024727693 | 0.771397611283628 | 379 | 379 |
| ANO8 | -0.102212758312276 | 0.0467555421315145 | 0.118885469272079 | 379 | 379 |
| ANO9 | -0.218488648178882 | 0.0000177527273683493 | 0.000268767772078959 | 379 | 379 |
| ANP32A | 0.1481356616997 | 0.00384837282230455 | 0.0175312558186057 | 379 | 379 |
| ANP32B | 0.0299343601341416 | 0.561264007761004 | 0.702255520340105 | 379 | 379 |
| ANP32C | -0.0217309889849499 | 0.673237010902252 | 0.786759206233857 | 379 | 371 |
| ANP32D | 0.0221330275753717 | 0.667549644999467 | 0.782262937525421 | 379 | 128 |
| ANP32E | 0.204370336811552 | 0.0000612599295062628 | 0.000693696106367892 | 379 | 379 |
| ANPEP | 0.259379057541381 | 3.04252687042759E-07 | 0.0000114908995784454 | 379 | 379 |
| ANTXR1 | 0.0720295332880012 | 0.161677550853603 | 0.296828007252337 | 379 | 379 |
| ANTXR2 | 0.232077757232808 | 4.97990385313997E-06 | 0.000101169604098422 | 379 | 379 |
| ANTXRL | -0.0488708900575165 | 0.342702466964519 | 0.504200082731504 | 379 | 21 |
| ANUBL1 | -0.0750820028620627 | 0.144586485788934 | 0.273436981947538 | 379 | 379 |
| ANXA10 | 0.203754242033789 | 0.0000645410180015145 | 0.000721375030966195 | 379 | 165 |
| ANXA11 | 0.127492770688918 | 0.0129928508944626 | 0.0447183210441597 | 379 | 379 |
| ANXA13 | -0.0139008827070113 | 0.787359953594541 | 0.869458760884774 | 379 | 379 |
| ANXA1 | 0.287152979299547 | 1.25467738184223E-08 | 0.0000010322714990526 | 379 | 379 |
| ANXA2P1 | 0.118631166201816 | 0.0208861300976629 | 0.0643458961430176 | 379 | 379 |
| ANXA2P2 | 0.157489268671714 | 0.00210450983698627 | 0.0108695548444292 | 379 | 379 |
| ANXA2P3 | 0.0722192006995393 | 0.160573176559452 | 0.295320002302273 | 379 | 378 |
| ANXA2 | 0.167382066012304 | 0.0010719718655326 | 0.00651845049849414 | 379 | 379 |
| ANXA3 | -0.0685899340787955 | 0.182708273851779 | 0.323068023585734 | 379 | 379 |
| ANXA4 | -0.00364263778009949 | 0.943651976351687 | 0.968112306656595 | 379 | 379 |
| ANXA5 | 0.147769461246765 | 0.00393775384604628 | 0.01785451252216 | 379 | 379 |
| ANXA6 | -0.0849191155693765 | 0.0987955913729301 | 0.207953183199836 | 379 | 379 |
| ANXA7 | -0.103544128862196 | 0.0439519852253466 | 0.113400125315312 | 379 | 379 |
| ANXA8L1 | 0.0689497718910565 | 0.180417884175556 | 0.320204601453001 | 379 | 16 |
| ANXA8L2 | 0.258413189287584 | 3.37776208614965E-07 | 0.0000126605418987099 | 379 | 315 |
| ANXA8 | 0.141598971846675 | 0.00575437324554038 | 0.0240205710973841 | 379 | 333 |
| ANXA9 | -0.133181225513343 | 0.00943790673016802 | 0.0349790793347228 | 379 | 379 |
| AOAH | -0.0567462159637725 | 0.270474729800656 | 0.426207815504046 | 379 | 379 |
| AOC2 | 0.251780482290697 | 0.0000006846286270475 | 0.0000221088215262179 | 379 | 378 |
| AOC3 | 0.106002362606032 | 0.0391464826975021 | 0.104013184257502 | 379 | 379 |
| AOX1 | 0.0705691763482129 | 0.170372772944867 | 0.307496026028656 | 379 | 377 |
| AOX2P | 0.0553952862785021 | 0.282063665881758 | 0.439371385584374 | 379 | 52 |
| AP1AR | 0.032811577390886 | 0.524232482340025 | 0.671391400325412 | 379 | 379 |
| AP1B1 | 0.233185431369577 | 4.47420662935011E-06 | 0.0000932855615633584 | 379 | 379 |
| AP1G1 | 0.0456067463274845 | 0.37594276747832 | 0.536581715632028 | 379 | 379 |
| AP1G2 | -0.0705263759291224 | 0.170632777899195 | 0.307881219418077 | 379 | 379 |
| AP1M1 | 0.089361886241352 | 0.0823128493221098 | 0.18172800093072 | 379 | 379 |
| AP1M2 | -0.121077296605033 | 0.0183727744389032 | 0.0583442610718501 | 379 | 379 |
| AP1S1 | -0.0386625613977304 | 0.452969187832091 | 0.60775971419236 | 379 | 379 |
| AP1S2 | 0.0591033999484388 | 0.251041497007575 | 0.405082259331559 | 379 | 379 |
| AP1S3 | 0.243563820131751 | 1.59880365261087E-06 | 0.0000427238259083132 | 379 | 379 |
| AP2A1 | 0.136086040471796 | 0.00798002762363346 | 0.0307896454021024 | 379 | 379 |
| AP2A2 | 0.00194573837994903 | 0.969883504339894 | 0.983908101325076 | 379 | 379 |
| AP2B1 | 0.0817516604648783 | 0.112074278235888 | 0.227242948037753 | 379 | 379 |
| AP2M1 | 0.0637495946752065 | 0.21562986188988 | 0.362977239286234 | 379 | 379 |
| AP2S1 | 0.188207744184817 | 0.000228798224373372 | 0.00191579864563987 | 379 | 379 |
| AP3B1 | -0.0530689473225374 | 0.302796100462958 | 0.461727376757635 | 379 | 379 |
| AP3B2 | 0.0199896039067018 | 0.698083102855978 | 0.804650143205926 | 379 | 330 |
| AP3D1 | 0.00796500944107603 | 0.8771741665768 | 0.928199881251189 | 379 | 379 |
| AP3M1 | 0.0085804286724728 | 0.867767880130071 | 0.922602899023933 | 379 | 379 |
| AP3M2 | 0.137624706673822 | 0.00729222239959271 | 0.0288143056474939 | 379 | 379 |
| AP3S1 | 0.0945520042451533 | 0.0659486989104602 | 0.153261931785819 | 379 | 379 |
| AP3S2 | 0.0359611550089729 | 0.485175780957176 | 0.637048234210905 | 379 | 379 |
| AP4B1 | -0.0454558453707667 | 0.377525933959695 | 0.538130761230508 | 379 | 379 |
| AP4E1 | 0.0755189449584294 | 0.142257259686817 | 0.270225817556641 | 379 | 379 |
| AP4M1 | -0.147244797736306 | 0.00406908577111434 | 0.0183045647701971 | 379 | 379 |
| AP4S1 | 0.0354224024828788 | 0.491743441500524 | 0.643031653239623 | 379 | 379 |
| APAF1 | -0.101169050179258 | 0.0490568044739192 | 0.123141957096958 | 379 | 379 |
| APBA1 | -0.103590639132998 | 0.0438566580918119 | 0.113242585837276 | 379 | 379 |
| APBA2 | 0.0436279014969377 | 0.397027229189787 | 0.55665789141388 | 379 | 379 |
| APBA3 | -0.0551483390355739 | 0.284217831492954 | 0.44179983531182 | 379 | 379 |
| APBB1IP | 0.217554356413731 | 0.0000193185624348069 | 0.000286927682365056 | 379 | 378 |
| APBB1 | -0.0131641622923912 | 0.798380633856823 | 0.876878702050246 | 379 | 379 |
| APBB2 | -0.0596457982806391 | 0.246710727341889 | 0.400276597801733 | 379 | 379 |
| APBB3 | -0.133553662504215 | 0.00923863025771506 | 0.0344071301183272 | 379 | 379 |
| APC2 | 0.194458399876742 | 0.000139178255283416 | 0.00129135537939147 | 379 | 378 |
| APCDD1L | 0.144333169299471 | 0.00487254770634824 | 0.020979994771221 | 379 | 336 |
| APCDD1 | -0.275407679330818 | 5.04689346406194E-08 | 2.97826796444703E-06 | 379 | 379 |
| APCS | 0.0176145816794019 | 0.732491543393028 | 0.830414066686545 | 379 | 21 |
| APC | 0.120029874611914 | 0.0194147456439287 | 0.0609106925044017 | 379 | 379 |
| APEH | -0.062794663246158 | 0.222598518963603 | 0.371240931450106 | 379 | 379 |
| APEX1 | -0.0181340219208962 | 0.724917350322839 | 0.824745307677373 | 379 | 379 |
| APEX2 | 0.0348439109638147 | 0.498848112367276 | 0.649367146272213 | 379 | 379 |
| APH1A | -0.119820342788348 | 0.0196292513672685 | 0.0613990844155544 | 379 | 379 |
| APH1B | -0.133143026286315 | 0.00945856001047677 | 0.0350281219693612 | 379 | 379 |
| API5 | 0.0106736734924363 | 0.83592101051815 | 0.901677825946789 | 379 | 379 |
| APIP | -0.166020721212512 | 0.00117887381421713 | 0.00698795515345207 | 379 | 379 |
| APITD1 | 0.20830525763038 | 0.0000437378310184731 | 0.000532371831451372 | 379 | 379 |
| APLF | -0.305889012675672 | 1.18885652611348E-09 | 1.69587389926461E-07 | 379 | 379 |
| APLNR | 0.112623459646304 | 0.0283591793058012 | 0.0813285807456504 | 379 | 379 |
| APLN | 0.106752524734203 | 0.0377716189722761 | 0.101262258110099 | 379 | 379 |
| APLP1 | 0.180736293333416 | 0.000406078740777492 | 0.00299104474364479 | 379 | 378 |
| APLP2 | 0.113765974030982 | 0.026782991579094 | 0.0780157421816183 | 379 | 379 |
| APOA1BP | -0.0212772796237905 | 0.67967773985414 | 0.79130116997404 | 379 | 379 |
| APOA1 | 0.106191120809154 | 0.0387966095484277 | 0.103325611031058 | 379 | 339 |
| APOA2 | 0.110874432624918 | 0.0309260750926157 | 0.0868804501185016 | 379 | 192 |
| APOA4 | 0.0707778998797121 | 0.16910905022091 | 0.305716105742178 | 379 | 109 |
| APOA5 | 0.033415468042488 | 0.516621478719997 | 0.664933271227964 | 379 | 129 |
| APOB48R | -0.0782138771853883 | 0.128520700183885 | 0.25113909956106 | 379 | 379 |
| APOBEC1 | 0.0460192888093371 | 0.371635486764146 | 0.532157754860944 | 379 | 378 |
| APOBEC2 | 0.0683742314918812 | 0.184091486530681 | 0.324834563934355 | 379 | 351 |
| APOBEC3A | 0.198244907350948 | 0.000102204533558934 | 0.00101755707447616 | 379 | 379 |
| APOBEC3B | 0.120351798215428 | 0.019089155028432 | 0.0601557161321916 | 379 | 379 |
| APOBEC3C | 0.0656953977536791 | 0.201917617197509 | 0.347265375469877 | 379 | 379 |
| APOBEC3D | 0.139814951744946 | 0.00640450470992585 | 0.0261078370453145 | 379 | 379 |
| APOBEC3F | 0.155314931852443 | 0.00242865683037886 | 0.0121943296107247 | 379 | 379 |
| APOBEC3G | 0.173287504297047 | 0.000703860187867934 | 0.00466136934036252 | 379 | 379 |
| APOBEC3H | 0.195913445937815 | 0.000123691018858442 | 0.00117459076720555 | 379 | 365 |
| APOBEC4 | -0.0541635163684032 | 0.292918716373226 | 0.451281453632348 | 379 | 332 |
| APOB | 0.0784496960332684 | 0.127369323432048 | 0.249331517919899 | 379 | 298 |
| APOC1P1 | 0.110711552437759 | 0.0311749282409938 | 0.0874216451736024 | 379 | 86 |
| APOC1 | 0.190462263471407 | 0.000191589822726294 | 0.00166178609143349 | 379 | 379 |
| APOC2 | 0.10621959717038 | 0.03874405753705 | 0.103199512741084 | 379 | 379 |
| APOC3 | -0.00446229417089939 | 0.931001329149618 | 0.960502333856008 | 379 | 80 |
| APOC4 | 0.0982244240682612 | 0.0560648365505644 | 0.136081965861744 | 379 | 169 |
| APOD | 0.0700979522825288 | 0.173251709132581 | 0.311331782552186 | 379 | 379 |
| APOE | 0.244340875986727 | 1.47745228821371E-06 | 0.0000400203879381168 | 379 | 379 |
| APOF | 0.0675884189352183 | 0.18919590157937 | 0.331481517762283 | 379 | 128 |
| APOH | 0.0291555294823987 | 0.571500501343599 | 0.710318557051393 | 379 | 332 |
| APOL1 | 0.295316708977256 | 4.58842276578834E-09 | 4.81064993596178E-07 | 379 | 379 |
| APOL2 | 0.292818720755241 | 6.26340711309282E-09 | 6.20954181192022E-07 | 379 | 379 |
| APOL3 | 0.198512328043739 | 0.0000999778831146272 | 0.00100474478783417 | 379 | 379 |
| APOL4 | 0.175258331719552 | 0.000609821460726385 | 0.00414803295876618 | 379 | 379 |
| APOL5 | 0.106027350266941 | 0.0391000140492759 | 0.103924273266628 | 379 | 53 |
| APOL6 | 0.22567014038089 | 9.15725235901709E-06 | 0.000163282373898013 | 379 | 379 |
| APOLD1 | -0.156694421541416 | 0.0022181181219463 | 0.0113235958089473 | 379 | 379 |
| APOM | 0.0291134432782682 | 0.57205616716452 | 0.710667218964216 | 379 | 379 |
| APOOL | 0.0216006815698088 | 0.675084401697327 | 0.787919696529416 | 379 | 379 |
| APOO | 0.0539979964358194 | 0.294398379510083 | 0.452787082603625 | 379 | 379 |
| APPBP2 | 0.0293271530799419 | 0.569237199765355 | 0.708571485777353 | 379 | 379 |
| APPL1 | -0.23318926645508 | 4.47254404117155E-06 | 0.0000932855615633584 | 379 | 379 |
| APPL2 | 0.0289740034109238 | 0.573899022126168 | 0.711871742928849 | 379 | 379 |
| APP | -0.141158153131283 | 0.00590925006818248 | 0.0245104213422834 | 379 | 379 |
| APRT | 0.0565521565582568 | 0.2721191663282 | 0.428254530514767 | 379 | 379 |
| APTX | -0.107175585719986 | 0.037014453548792 | 0.0996229923938437 | 379 | 379 |
| AQP10 | 0.0959043919237711 | 0.0621542999887962 | 0.146748685422464 | 379 | 175 |
| AQP11 | 0.0577238569432615 | 0.262293612669341 | 0.41686099326769 | 379 | 379 |
| AQP12A | -0.0193910975579027 | 0.706699228065943 | 0.811044816223157 | 379 | 257 |
| AQP12B | -0.0801180889988979 | 0.119450397287022 | 0.237869084805369 | 379 | 369 |
| AQP1 | -0.0187070997478555 | 0.716592066440782 | 0.819033173471745 | 379 | 379 |
| AQP2 | 0.101656497835234 | 0.0479705140290165 | 0.121213119939765 | 379 | 218 |
| AQP3 | 0.0686438274005675 | 0.182363879241665 | 0.32276274190875 | 379 | 379 |
| AQP4 | 0.0244615120865798 | 0.634991664903504 | 0.759110981050686 | 379 | 166 |
| AQP5 | 0.188497911175897 | 0.000223656527007764 | 0.00187909390572456 | 379 | 354 |
| AQP6 | -0.0464852877911281 | 0.366806908668587 | 0.527759043979446 | 379 | 367 |
| AQP7P1 | -0.128057004931214 | 0.0125939571353506 | 0.0436713854634019 | 379 | 361 |
| AQP7P3 | -0.0814028100080182 | 0.113618631357329 | 0.229412446288504 | 379 | 254 |
| AQP7 | -0.0925431354580542 | 0.0719341022554139 | 0.163849440496306 | 379 | 379 |
| AQP8 | -0.0593221494151325 | 0.249288579851719 | 0.403073796077623 | 379 | 351 |
| AQP9 | 0.209581916381037 | 0.00003915522461197 | 0.000494818224095692 | 379 | 378 |
| AQPEP | 0.132045162539577 | 0.0100695633405588 | 0.0367967751412828 | 379 | 332 |
| AQR | 0.0715485817359529 | 0.164503576187597 | 0.300169801591453 | 379 | 379 |
| ARAF | -0.0488922460834721 | 0.342491341618126 | 0.504001656642734 | 379 | 379 |
| ARAP1 | -0.0214686347058907 | 0.676958444075945 | 0.789572472302226 | 379 | 379 |
| ARAP2 | 0.190487417500901 | 0.000191208614134021 | 0.00166036982261182 | 379 | 379 |
| ARAP3 | -0.0824970725675239 | 0.108829644098894 | 0.222392474821484 | 379 | 379 |
| ARCN1 | 0.203381119877516 | 0.0000666079433334167 | 0.000737822514198316 | 379 | 379 |
| ARC | 0.140014999413326 | 0.00632846263050559 | 0.0258563274340954 | 379 | 370 |
| AREG | -0.0348547354105685 | 0.49871467626043 | 0.649265185858852 | 379 | 379 |
| ARF1 | 0.0113238419946229 | 0.826081024003493 | 0.895264675809092 | 379 | 379 |
| ARF3 | 0.169731491038441 | 0.000908245295394667 | 0.00569534715910356 | 379 | 379 |
| ARF4 | -0.0257840797895392 | 0.616802185322851 | 0.744548504236058 | 379 | 379 |
| ARF5 | -0.0316335315537146 | 0.5392426938672 | 0.684469888867476 | 379 | 379 |
| ARF6 | 0.133187608842735 | 0.00943445934771592 | 0.0349790793347228 | 379 | 379 |
| ARFGAP1 | -0.128885088294927 | 0.012028087033116 | 0.0421728096480998 | 379 | 379 |
| ARFGAP2 | -0.0867186405441481 | 0.0918280967096327 | 0.197373170900661 | 379 | 379 |
| ARFGAP3 | 0.204964816340575 | 0.0000582435849188808 | 0.000667545550156976 | 379 | 379 |
| ARFGEF1 | -0.166418463623698 | 0.00114666766474718 | 0.00684616876139929 | 379 | 379 |
| ARFGEF2 | -0.304712618829656 | 0.000000001385373152 | 1.93444921534275E-07 | 379 | 379 |
| ARFIP1 | -0.126092356563496 | 0.014031359741537 | 0.0474362831978169 | 379 | 379 |
| ARFIP2 | -0.0172475353849154 | 0.737859353126822 | 0.833823962943043 | 379 | 379 |
| ARFRP1 | -0.195829823192082 | 0.000124535350132526 | 0.0011797835271991 | 379 | 379 |
| ARG1 | 0.014661985811187 | 0.77601892474998 | 0.861963096742065 | 379 | 217 |
| ARG2 | 0.078687819531597 | 0.126214821100945 | 0.247584831103041 | 379 | 379 |
| ARGFXP2 | 0.0140275202609553 | 0.785469766459326 | 0.868402071223073 | 379 | 308 |
| ARGFX | 0.0149021106910021 | 0.772450600025734 | 0.859299287326652 | 379 | 13 |
| ARGLU1 | -0.13456714699789 | 0.00871516117264873 | 0.0329088203639838 | 379 | 379 |
| ARHGAP10 | 0.231931872742967 | 5.05043470758318E-06 | 0.000102288068827333 | 379 | 379 |
| ARHGAP11A | 0.219926193005434 | 0.0000155765430029056 | 0.000243835223980131 | 379 | 379 |
| ARHGAP11B | 0.164392081583069 | 0.00131962194375685 | 0.00757760321483081 | 379 | 379 |
| ARHGAP12 | 0.0334112255563133 | 0.516674748326066 | 0.664933271227964 | 379 | 379 |
| ARHGAP15 | 0.144350020295696 | 0.00486751307119916 | 0.0209719793949885 | 379 | 379 |
| ARHGAP17 | -0.00952975454989106 | 0.853294998334268 | 0.913461463501343 | 379 | 379 |
| ARHGAP18 | -0.203836439421877 | 0.0000640938827857147 | 0.000718024508996667 | 379 | 379 |
| ARHGAP19 | 0.186281077763686 | 0.000265842861112612 | 0.00216473603701884 | 379 | 379 |
| ARHGAP1 | 0.0301607373386954 | 0.558305298410688 | 0.699929024841118 | 379 | 379 |
| ARHGAP20 | 0.10251569621207 | 0.0461047907600226 | 0.117580382056193 | 379 | 377 |
| ARHGAP21 | -0.00809261446276828 | 0.875222340608195 | 0.927223155032021 | 379 | 379 |
| ARHGAP22 | 0.0740270712910245 | 0.150329933541249 | 0.281387890329074 | 379 | 379 |
| ARHGAP23 | 0.0691766241835149 | 0.178984913854261 | 0.318533449142516 | 379 | 379 |
| ARHGAP24 | 0.0468801459429135 | 0.362746113447657 | 0.524089458690002 | 379 | 379 |
| ARHGAP25 | 0.178721794791813 | 0.000472222915979517 | 0.00336807049569851 | 379 | 379 |
| ARHGAP26 | -0.0836267994461193 | 0.104053770806879 | 0.215291328401709 | 379 | 379 |
| ARHGAP27 | 0.0408185059494123 | 0.428152594508573 | 0.585290681613898 | 379 | 379 |
| ARHGAP28 | -0.0691584633257865 | 0.179099320152925 | 0.31866307609406 | 379 | 379 |
| ARHGAP29 | 0.196809465209963 | 0.000114975089113089 | 0.00111314749362028 | 379 | 379 |
| ARHGAP30 | 0.206575154558527 | 0.0000507611531124766 | 0.000598390097451953 | 379 | 379 |
| ARHGAP31 | 0.0610578301447643 | 0.23568178014407 | 0.386845888799389 | 379 | 379 |
| ARHGAP32 | 0.149992637380892 | 0.00342276129349984 | 0.0159949354059662 | 379 | 379 |
| ARHGAP33 | -0.13885782154002 | 0.00677977883693869 | 0.027228976054045 | 379 | 379 |
| ARHGAP36 | 0.0139932767265549 | 0.785980760352863 | 0.868697130227233 | 379 | 157 |
| ARHGAP39 | -0.110447056428152 | 0.0315826790995527 | 0.0883121366783149 | 379 | 379 |
| ARHGAP42 | 0.0622236778674886 | 0.226841248785195 | 0.376385630201912 | 379 | 379 |
| ARHGAP4 | 0.0457621992931167 | 0.3743161198792 | 0.535146010885052 | 379 | 379 |
| ARHGAP5 | 0.0449668754372877 | 0.382683981511324 | 0.542609981793945 | 379 | 379 |
| ARHGAP6 | -0.0257858640335294 | 0.616777800539692 | 0.744548504236058 | 379 | 379 |
| ARHGAP8 | 0.00988753444634049 | 0.847852954367231 | 0.909796968242963 | 379 | 379 |
| ARHGAP9 | 0.219442809813718 | 0.0000162783537960751 | 0.000250595651450759 | 379 | 379 |
| ARHGDIA | 0.14810421599985 | 0.00385597530025632 | 0.0175559766368501 | 379 | 379 |
| ARHGDIB | 0.169968174095393 | 0.000893100459920541 | 0.00562528459952493 | 379 | 379 |
| ARHGDIG | -0.190509175189414 | 0.000190879449783818 | 0.00165925371780515 | 379 | 342 |
| ARHGEF10L | -0.153903848638577 | 0.00266271974455367 | 0.0131138616728788 | 379 | 379 |
| ARHGEF10 | 0.0313810302943729 | 0.54248765412086 | 0.687312792709803 | 379 | 379 |
| ARHGEF11 | -0.117366615581091 | 0.0222987502818323 | 0.0676188610055371 | 379 | 379 |
| ARHGEF12 | 0.00475552034691071 | 0.926479697917201 | 0.958267068657967 | 379 | 379 |
| ARHGEF15 | 0.149805140046074 | 0.00346370992072369 | 0.0161406440207073 | 379 | 379 |
| ARHGEF16 | 0.111014959729534 | 0.030712740374512 | 0.0864526144443247 | 379 | 379 |
| ARHGEF17 | 0.096571528372217 | 0.0603498499956858 | 0.143703233543228 | 379 | 379 |
| ARHGEF18 | 0.0595844228194313 | 0.247198146054298 | 0.400706739696257 | 379 | 379 |
| ARHGEF19 | -0.0490372383918565 | 0.34106014074493 | 0.502324727411519 | 379 | 379 |
| ARHGEF1 | 0.00342804763653213 | 0.946966448916672 | 0.97010853780004 | 379 | 379 |
| ARHGEF2 | 0.0132288953799392 | 0.797410644734438 | 0.876347315363842 | 379 | 379 |
| ARHGEF33 | -0.0595240246682899 | 0.247678458702797 | 0.401354076426861 | 379 | 370 |
| ARHGEF35 | -0.142305745473312 | 0.00551365370339537 | 0.0231767491267592 | 379 | 379 |
| ARHGEF37 | -0.0859998757959864 | 0.0945623566936789 | 0.201577015899211 | 379 | 379 |
| ARHGEF38 | -0.100186639158427 | 0.0513086787068565 | 0.127263475244086 | 379 | 379 |
| ARHGEF3 | -0.136742036039509 | 0.0076800095286145 | 0.0299484541660798 | 379 | 379 |
| ARHGEF4 | 0.141064253088792 | 0.00594272032315864 | 0.0246188242310506 | 379 | 379 |
| ARHGEF5 | -0.145685577003289 | 0.00448308271560905 | 0.0196834730037857 | 379 | 379 |
| ARHGEF6 | 0.125153210161029 | 0.0147680442413323 | 0.0493795583840028 | 379 | 379 |
| ARHGEF7 | -0.140735397706733 | 0.00606128499036061 | 0.0249601575885504 | 379 | 379 |
| ARHGEF9 | -0.15607078734927 | 0.00231114363238423 | 0.0117140480426673 | 379 | 379 |
| ARID1A | 0.100572508432722 | 0.0504141207256851 | 0.125674023855781 | 379 | 379 |
| ARID1B | -0.127067052934929 | 0.0133011446915947 | 0.0454414651093482 | 379 | 379 |
| ARID2 | 0.035599565994102 | 0.489578500001619 | 0.641046192830489 | 379 | 379 |
| ARID3A | -0.0431538061685088 | 0.40218223786811 | 0.561503268022031 | 379 | 379 |
| ARID3B | 0.173896542045915 | 0.000673456328410425 | 0.00450667974340935 | 379 | 379 |
| ARID3C | 0.083017034054408 | 0.106610503774186 | 0.218918089150225 | 379 | 281 |
| ARID4A | -0.104793242164157 | 0.0414515247121559 | 0.108659549443764 | 379 | 379 |
| ARID4B | -0.0879858912422024 | 0.0871617555289693 | 0.189729200639851 | 379 | 379 |
| ARID5A | -0.13633713823649 | 0.0078639754151816 | 0.0304425819078916 | 379 | 379 |
| ARID5B | 0.110321353799767 | 0.0317780557196894 | 0.0887708211904763 | 379 | 379 |
| ARIH1 | 0.1216924502353 | 0.0177838290221499 | 0.0568300824086938 | 379 | 379 |
| ARIH2 | -0.164209603757258 | 0.00133631863892043 | 0.00765795548338563 | 379 | 379 |
| ARL10 | -0.0223536516551945 | 0.664436663781794 | 0.779645530208629 | 379 | 378 |
| ARL11 | -0.0708877257722042 | 0.168446924595719 | 0.304933348967035 | 379 | 379 |
| ARL13A | 0.032757235179153 | 0.524920168633776 | 0.671968306777101 | 379 | 243 |
| ARL13B | 0.0938360091534866 | 0.068033315968014 | 0.156984125336178 | 379 | 378 |
| ARL14 | 0.014466166841044 | 0.778932344646571 | 0.864230906477097 | 379 | 379 |
| ARL15 | -0.208518878330756 | 0.00004293728336469 | 0.000525987095115282 | 379 | 379 |
| ARL16 | -0.036682286302222 | 0.476459401280999 | 0.628516865717494 | 379 | 379 |
| ARL17A | 0.139156110271156 | 0.00666076653395915 | 0.0268652723424211 | 379 | 379 |
| ARL17B | 0.0154597806441968 | 0.764182049227955 | 0.853693260019826 | 379 | 332 |
| ARL1 | 0.0401633416377118 | 0.435609458650561 | 0.592283093062012 | 379 | 379 |
| ARL2BP | -0.0616893585318403 | 0.230863268169247 | 0.381217888291459 | 379 | 379 |
| ARL2 | 0.0856872775699351 | 0.095771616229779 | 0.203401843038138 | 379 | 379 |
| ARL3 | -0.0278160583441656 | 0.589309726138732 | 0.724101955126652 | 379 | 379 |
| ARL4A | 0.0381936660699317 | 0.45847168062121 | 0.612861624981955 | 379 | 379 |
| ARL4C | 0.176462136261373 | 0.000558257428879785 | 0.00385952869589553 | 379 | 379 |
| ARL4D | -0.0244371942745245 | 0.635328219923225 | 0.75933019557792 | 379 | 379 |
| ARL5A | -0.161259097345088 | 0.00163467288844473 | 0.00895367238455304 | 379 | 379 |
| ARL5B | 0.0816695905382145 | 0.112436108299413 | 0.227673933349751 | 379 | 379 |
| ARL5C | 0.0386835110855293 | 0.452724212813367 | 0.607566082793569 | 379 | 41 |
| ARL6IP1 | 0.0256313273145477 | 0.61889137079653 | 0.745980431620279 | 379 | 379 |
| ARL6IP4 | 0.0089348369231805 | 0.862359339246043 | 0.919095945956275 | 379 | 379 |
| ARL6IP5 | -0.129853208301385 | 0.0113950831618341 | 0.0403395302504635 | 379 | 379 |
| ARL6IP6 | 0.0813215323145895 | 0.113980832722483 | 0.22993305028196 | 379 | 379 |
| ARL6 | -0.0455266502843102 | 0.376782580326098 | 0.537509533254614 | 379 | 379 |
| ARL8A | -0.165730160198479 | 0.00120292518878707 | 0.00709024989395659 | 379 | 379 |
| ARL8B | -0.0968061538199199 | 0.0597256140654063 | 0.142623196756489 | 379 | 379 |
| ARL9 | 0.0532984444021822 | 0.300707055629255 | 0.459708519584955 | 379 | 342 |
| ARMC10 | -0.121014138053891 | 0.0184341940397683 | 0.0585101968017489 | 379 | 379 |
| ARMC1 | -0.0420794110645606 | 0.414011515713551 | 0.572376260881906 | 379 | 379 |
| ARMC2 | -0.291803936573109 | 7.10135417416842E-09 | 6.79571975534486E-07 | 379 | 379 |
| ARMC3 | 0.0219501869866452 | 0.670133833050554 | 0.784473588483078 | 379 | 113 |
| ARMC4 | 0.0343660609569633 | 0.504757492870712 | 0.654567139871843 | 379 | 327 |
| ARMC5 | 0.0342934868882575 | 0.505658191232091 | 0.655392248382135 | 379 | 379 |
| ARMC6 | 0.0820347001636609 | 0.110833425377196 | 0.225266368596408 | 379 | 379 |
| ARMC7 | 0.181619770613446 | 0.000379882391161924 | 0.00283916624649779 | 379 | 379 |
| ARMC8 | 0.0205744608367464 | 0.689700742546958 | 0.798942771418491 | 379 | 379 |
| ARMC9 | 0.0972009041451412 | 0.0586873985162246 | 0.140697500003038 | 379 | 379 |
| ARMCX1 | 0.0158271533921247 | 0.75874955886549 | 0.849586980640667 | 379 | 379 |
| ARMCX2 | -0.022826817998303 | 0.657779804315829 | 0.774768798857922 | 379 | 379 |
| ARMCX3 | -0.170210772189177 | 0.000877819379049595 | 0.00554842290334567 | 379 | 379 |
| ARMCX5 | -0.127103818073078 | 0.0132742685284832 | 0.0453875144650396 | 379 | 379 |
| ARMCX6 | -0.134724658982589 | 0.00863622055670179 | 0.0326604961278434 | 379 | 379 |
| ARMS2 | -0.0106410421858868 | 0.836415557879813 | 0.90190662018126 | 379 | 117 |
| ARNT2 | 0.038164661933231 | 0.458813266142739 | 0.613111567669378 | 379 | 379 |
| ARNTL2 | 0.357354369521972 | 7.38233323779667E-13 | 6.27101536085147E-10 | 379 | 379 |
| ARNTL | 0.0410508943275458 | 0.425525509965514 | 0.583130818411515 | 379 | 379 |
| ARNT | -0.0743793050415017 | 0.148393280989279 | 0.278947855086786 | 379 | 379 |
| ARPC1A | -0.00604228585008266 | 0.906667280755987 | 0.946079299170072 | 379 | 379 |
| ARPC1B | 0.067912602897029 | 0.187077643381084 | 0.328757911278927 | 379 | 379 |
| ARPC2 | 0.154687827869756 | 0.00253025922760387 | 0.0125865479089136 | 379 | 379 |
| ARPC3 | -0.010327700452501 | 0.841167694067782 | 0.905514579400401 | 379 | 379 |
| ARPC4 | -0.0875775010836985 | 0.0886442714148104 | 0.19200585806541 | 379 | 379 |
| ARPC5L | 0.0486727127166302 | 0.344665595998327 | 0.506299410094445 | 379 | 379 |
| ARPC5 | -0.126918751630913 | 0.0134100433798752 | 0.045749198234027 | 379 | 379 |
| ARPM1 | -0.253532159652257 | 5.69183300144058E-07 | 0.0000191284177546718 | 379 | 379 |
| ARPP19 | 0.0896764439216736 | 0.0812355412936092 | 0.179850191242707 | 379 | 379 |
| ARPP21 | 0.0333975157130552 | 0.51684691156801 | 0.665111666833495 | 379 | 129 |
| ARR3 | 0.0866947231948815 | 0.0919180519798628 | 0.19745949454569 | 379 | 78 |
| ARRB1 | 0.0908440558380045 | 0.0773359549955242 | 0.172818360830751 | 379 | 379 |
| ARRB2 | 0.0965061995411071 | 0.0605246171118794 | 0.14394651650406 | 379 | 379 |
| ARRDC1 | -0.0482762276300839 | 0.348614546472014 | 0.510247968365475 | 379 | 379 |
| ARRDC2 | 0.0871796295443851 | 0.090108002377452 | 0.194371352386871 | 379 | 379 |
| ARRDC3 | -0.0916099335906275 | 0.074861593977546 | 0.168809056893565 | 379 | 379 |
| ARRDC4 | 0.0579283420842476 | 0.260604194184084 | 0.414674581677395 | 379 | 379 |
| ARRDC5 | 0.101306850688891 | 0.0487476472988398 | 0.122552092131532 | 379 | 288 |
| ARSA | 0.108001689162344 | 0.0355730195209207 | 0.096586503670476 | 379 | 379 |
| ARSB | 0.131033941321499 | 0.0106630590596009 | 0.0383297924281664 | 379 | 379 |
| ARSD | -0.200996675836404 | 0.0000813635402763422 | 0.000858582371793142 | 379 | 379 |
| ARSE | -0.0279288848440489 | 0.58779982629604 | 0.722959375465951 | 379 | 377 |
| ARSF | 0.0773598631895857 | 0.132757906718235 | 0.257087974842188 | 379 | 104 |
| ARSG | -0.118562509619962 | 0.0209607820167896 | 0.0645257546699121 | 379 | 379 |
| ARSH | -0.0288335225966433 | 0.575758462223287 | 0.713144841121709 | 379 | 305 |
| ARSI | 0.175543828227811 | 0.000597207875129994 | 0.00407062143282142 | 379 | 378 |
| ARSJ | 0.209497065060548 | 0.0000394451427171528 | 0.000497530718699559 | 379 | 379 |
| ARSK | 0.0514394314116013 | 0.317904984704282 | 0.478038831845632 | 379 | 379 |
| ART1 | -0.0649547563001932 | 0.207060385217045 | 0.353382106910275 | 379 | 109 |
| ART3 | -0.0658389295825535 | 0.200931802231528 | 0.345990080299326 | 379 | 360 |
| ART4 | 0.0441305793435406 | 0.391605021615078 | 0.551512491553644 | 379 | 237 |
| ART5 | 0.00289187907225933 | 0.955251764069016 | 0.975672589396819 | 379 | 344 |
| ARTN | 0.0467491774125043 | 0.364089900174726 | 0.52521378040309 | 379 | 379 |
| ARV1 | -0.177233535578907 | 0.000527373679212455 | 0.00368585312351941 | 379 | 379 |
| ARVCF | -0.106243533292878 | 0.0386999309707927 | 0.103138069199622 | 379 | 379 |
| ARX | 0.150423815679008 | 0.00333025411127828 | 0.015636343480565 | 379 | 302 |
| AR | 0.00139141145741545 | 0.9784609883779 | 0.988292336739594 | 379 | 343 |
| AS3MT | -0.178079325811967 | 0.000495338719882992 | 0.00349646711920255 | 379 | 378 |
| ASAH1 | 0.0372818954209637 | 0.469277433125953 | 0.622022390802954 | 379 | 379 |
| ASAH2B | -0.0466068201449089 | 0.365554050750105 | 0.526529545130981 | 379 | 371 |
| ASAH2 | 0.059041969172844 | 0.251535300752408 | 0.405582007263459 | 379 | 374 |
| ASAM | 0.0944811479507581 | 0.0661526308179207 | 0.153573862997042 | 379 | 379 |
| ASAP1IT1 | 0.0942827026468736 | 0.0667265331268084 | 0.15468252292597 | 379 | 12 |
| ASAP1 | -0.0360084828901027 | 0.484601099881881 | 0.636504412324982 | 379 | 379 |
| ASAP2 | 0.117256461753854 | 0.0224256315974959 | 0.0679302292161193 | 379 | 379 |
| ASAP3 | 0.0480587641176173 | 0.350792570807175 | 0.512075027163709 | 379 | 379 |
| ASB10 | 0.120295165225794 | 0.01914608611345 | 0.06028721541329 | 379 | 18 |
| ASB11 | 0.0743461982348762 | 0.148574496794478 | 0.279160974769526 | 379 | 14 |
| ASB12 | -0.206957181336505 | 0.0000491243391644932 | 0.00058395527395298 | 379 | 337 |
| ASB13 | -0.273440351905638 | 6.33200887591134E-08 | 3.48752977754361E-06 | 379 | 379 |
| ASB14 | -0.110015237968798 | 0.032258166355161 | 0.0898173020011665 | 379 | 378 |
| ASB15 | 0.0375330996339482 | 0.466286390111231 | 0.619341274325126 | 379 | 37 |
| ASB16 | -0.108074821128117 | 0.0354477445088072 | 0.0963743148389728 | 379 | 379 |
| ASB17 | 0.0481213061996968 | 0.350165296829373 | 0.511649042412144 | 379 | 9 |
| ASB18 | -0.100780638531803 | 0.0499370431140818 | 0.124798549390725 | 379 | 49 |
| ASB1 | -0.0369173913671369 | 0.4736362463149 | 0.626009495162031 | 379 | 379 |
| ASB2 | 0.0476681323236701 | 0.354726503303523 | 0.515842838834048 | 379 | 379 |
| ASB3 | 0.0933625731222282 | 0.0694411393647871 | 0.159582627645457 | 379 | 379 |
| ASB4 | -0.0218734733710131 | 0.671219230565342 | 0.785272604741765 | 379 | 276 |
| ASB5 | -0.0395660218105544 | 0.442472512971851 | 0.598203773946496 | 379 | 167 |
| ASB6 | -0.0507135749360276 | 0.32479087236581 | 0.485291679540912 | 379 | 379 |
| ASB7 | 0.100504968182004 | 0.0505697522560545 | 0.125953045330642 | 379 | 379 |
| ASB8 | -0.0936291233826723 | 0.0686456150502265 | 0.158084234055272 | 379 | 379 |
| ASB9 | -0.105704296454896 | 0.0397043968968218 | 0.10509395029638 | 379 | 379 |
| ASCC1 | 0.0406971342293013 | 0.429528396283183 | 0.58662539770408 | 379 | 379 |
| ASCC2 | 0.0830929109759455 | 0.106289677393852 | 0.218372367978996 | 379 | 379 |
| ASCC3 | 0.0616886426035482 | 0.230868690871689 | 0.381217888291459 | 379 | 379 |
| ASCL1 | 0.139022271357666 | 0.00671393272709643 | 0.0270235780742927 | 379 | 174 |
| ASCL2 | -0.20054579510872 | 0.0000844791250475145 | 0.000883466292954703 | 379 | 379 |
| ASCL3 | -0.0595938545076318 | 0.247123199812541 | 0.400683523254809 | 379 | 46 |
| ASCL4 | 0.00559706585379127 | 0.913516453196098 | 0.950425240527454 | 379 | 93 |
| ASF1A | -0.110686381326814 | 0.0312135376837856 | 0.087489684081722 | 379 | 379 |
| ASF1B | 0.123259275560969 | 0.0163576047133324 | 0.0533100897856601 | 379 | 379 |
| ASFMR1 | -0.0255690920789496 | 0.619743439444969 | 0.746780487226669 | 379 | 279 |
| ASGR1 | -0.189677649438844 | 0.000203844028182121 | 0.00174141292149724 | 379 | 379 |
| ASGR2 | 0.135784114172022 | 0.0081215926713072 | 0.0312204225449165 | 379 | 371 |
| ASH1L | -0.13936136210678 | 0.00657996446631216 | 0.0266205948659534 | 379 | 379 |
| ASH2L | 0.0231529199140136 | 0.653207601439455 | 0.771491560718461 | 379 | 379 |
| ASIP | 0.023826629548199 | 0.643802793236487 | 0.765986468736246 | 379 | 203 |
| ASL | -0.106142757811997 | 0.0388859997834978 | 0.1035219661261 | 379 | 379 |
| ASMTL | -0.0823474166323373 | 0.109475059268382 | 0.223389057854635 | 379 | 379 |
| ASMT | -0.0283719376275741 | 0.581887986286566 | 0.718276473391647 | 379 | 266 |
| ASNA1 | 0.0775009729461613 | 0.13205045278696 | 0.256102713507089 | 379 | 379 |
| ASNSD1 | -0.124755837840959 | 0.0150898352504587 | 0.050153507603285 | 379 | 379 |
| ASNS | -0.0113748307616071 | 0.825310461727753 | 0.894951386226961 | 379 | 379 |
| ASPA | 0.155635724804488 | 0.0023781332185562 | 0.0119953257332822 | 379 | 338 |
| ASPDH | 0.00571532374166823 | 0.911696553022168 | 0.949229114331209 | 379 | 192 |
| ASPG | -0.0762432341509703 | 0.138459621721271 | 0.265048984310616 | 379 | 333 |
| ASPHD1 | -0.0465057368204312 | 0.366595916477123 | 0.527608610866546 | 379 | 379 |
| ASPHD2 | 0.116049769885998 | 0.0238570079156354 | 0.0711095441143076 | 379 | 379 |
| ASPH | 0.102636609196206 | 0.0458471831733459 | 0.117131707605564 | 379 | 379 |
| ASPM | 0.0928707557815232 | 0.0709287643368603 | 0.162155602360344 | 379 | 379 |
| ASPN | 0.0636395372071103 | 0.216424936980412 | 0.363931862443351 | 379 | 379 |
| ASPRV1 | 0.190868315728917 | 0.000185522200005678 | 0.00162407690141836 | 379 | 379 |
| ASPSCR1 | -0.146029701065794 | 0.00438857502463951 | 0.0193742261219088 | 379 | 379 |
| ASRGL1 | 0.0629100529602784 | 0.221748028692137 | 0.370227341942379 | 379 | 379 |
| ASS1 | 0.124653559495116 | 0.0151736488099503 | 0.0503452323634028 | 379 | 379 |
| ASTE1 | -0.149428730389784 | 0.00354725965670592 | 0.0164526466603895 | 379 | 379 |
| ASTL | 0.0751572881770417 | 0.144183096718274 | 0.272922428804767 | 379 | 332 |
| ASTN1 | 0.0602028184845668 | 0.242317754316654 | 0.394729378828045 | 379 | 250 |
| ASTN2 | 0.0206137646831408 | 0.689138770796469 | 0.798611545724862 | 379 | 379 |
| ASXL1 | -0.250748727912581 | 7.62813157907569E-07 | 0.0000238189910157343 | 379 | 379 |
| ASXL2 | 0.0104378375569187 | 0.839496689973041 | 0.90422120986295 | 379 | 379 |
| ASXL3 | -0.0904882242648379 | 0.0785079284932464 | 0.175023072426815 | 379 | 358 |
| ASZ1 | 0.023562452409513 | 0.647483975262675 | 0.768347133970215 | 379 | 18 |
| ATAD1 | 0.0159143154394862 | 0.757462385999721 | 0.848624940083764 | 379 | 379 |
| ATAD2B | -0.106948472537168 | 0.0374193089650343 | 0.100526473809364 | 379 | 379 |
| ATAD2 | 0.112834664122983 | 0.0280619487350231 | 0.0808075624450612 | 379 | 379 |
| ATAD3A | 0.185860766349139 | 0.000274635891823695 | 0.00221541109157047 | 379 | 379 |
| ATAD3B | 0.178803507607109 | 0.000469355854260698 | 0.00335244520110992 | 379 | 379 |
| ATAD3C | 0.0447780778534914 | 0.384687027372843 | 0.544632229828542 | 379 | 379 |
| ATAD5 | 0.169951046638186 | 0.00089418853390183 | 0.0056303493966991 | 379 | 379 |
| ATCAY | 0.0301706005290995 | 0.558176560170128 | 0.699888862165494 | 379 | 276 |
| ATE1 | -0.00519900003906542 | 0.919645652622051 | 0.954347052774065 | 379 | 379 |
| ATF1 | -0.0204410333527212 | 0.691609777222445 | 0.800295333170122 | 379 | 379 |
| ATF2 | 0.018839907019866 | 0.714667469413122 | 0.817614999601895 | 379 | 379 |
| ATF3 | 0.000791630151931058 | 0.987744583911248 | 0.993355630441886 | 379 | 379 |
| ATF4 | 0.11349923512369 | 0.0271440742333738 | 0.0788359021384702 | 379 | 379 |
| ATF5 | 0.267660470402328 | 1.22038229640521E-07 | 5.83042464112369E-06 | 379 | 379 |
| ATF6B | -0.2144844772128 | 0.0000254377166511399 | 0.000349535028245878 | 379 | 379 |
| ATF6 | -0.0740511481033146 | 0.150196946160116 | 0.28124148690536 | 379 | 379 |
| ATF7IP2 | -0.115336310292972 | 0.0247399102255723 | 0.0732180811896873 | 379 | 379 |
| ATF7IP | -0.00399354403456667 | 0.938234139315318 | 0.96480170699845 | 379 | 379 |
| ATF7 | -0.043217224041277 | 0.401490365188489 | 0.560932283043782 | 379 | 379 |
| ATG10 | -0.142321077817809 | 0.00550853350715313 | 0.0231601362128567 | 379 | 379 |
| ATG12 | 0.0285709908470354 | 0.579240975667439 | 0.716078936687697 | 379 | 379 |
| ATG16L1 | 0.0959308665214014 | 0.0620818545602793 | 0.146664960350437 | 379 | 379 |
| ATG16L2 | 0.0826838567671837 | 0.108028317377411 | 0.221174848381583 | 379 | 379 |
| ATG2A | 0.0837263905897902 | 0.103640835222709 | 0.214710111879206 | 379 | 379 |
| ATG2B | -0.0872762368203034 | 0.0897508245508305 | 0.19389620278861 | 379 | 379 |
| ATG3 | -0.175867093992978 | 0.000583217799738894 | 0.00399586818701548 | 379 | 379 |
| ATG4A | 0.0687153296073793 | 0.181907698900329 | 0.322127878342032 | 379 | 379 |
| ATG4B | -0.0170779775051645 | 0.740343349241408 | 0.835583329278156 | 379 | 379 |
| ATG4C | 0.121804869556083 | 0.0176779986194185 | 0.0565353801009403 | 379 | 379 |
| ATG4D | 0.0110722305288461 | 0.829885905161891 | 0.897609520376935 | 379 | 379 |
| ATG5 | -0.0713052760146099 | 0.16594725602066 | 0.302010297602354 | 379 | 379 |
| ATG7 | -0.0304101154633112 | 0.555054738469196 | 0.697706691667759 | 379 | 379 |
| ATG9A | 0.0676644904725237 | 0.188697266373722 | 0.330754013230036 | 379 | 379 |
| ATG9B | -0.0557584317346859 | 0.27891596368351 | 0.435752468666328 | 379 | 379 |
| ATHL1 | -0.027377770115111 | 0.595191950397927 | 0.728288032768751 | 379 | 379 |
| ATIC | -0.118153969485452 | 0.0214098040959335 | 0.0655428688451178 | 379 | 379 |
| ATL1 | -0.147372712520183 | 0.00403670663763781 | 0.0181991403390365 | 379 | 379 |
| ATL2 | 0.0800476398447245 | 0.119776822130351 | 0.23830371555294 | 379 | 379 |
| ATL3 | 0.249120920123434 | 9.03848409299321E-07 | 0.0000271950019113611 | 379 | 379 |
| ATMIN | 0.00457724833698832 | 0.929228430663969 | 0.959871910560311 | 379 | 379 |
| ATM | 0.0260446324808777 | 0.613245750809084 | 0.741971976265714 | 379 | 379 |
| ATN1 | 0.0732151514572011 | 0.154867079993889 | 0.28770771686675 | 379 | 379 |
| ATOH1 | -0.00184996056031264 | 0.971365320506856 | 0.985037029408142 | 379 | 365 |
| ATOH7 | -0.0732776371688129 | 0.154514261405654 | 0.287348412600949 | 379 | 359 |
| ATOH8 | -0.162755739064708 | 0.00147644486370532 | 0.00826508999366154 | 379 | 379 |
| ATOX1 | 0.00404460699910523 | 0.93744598111191 | 0.964308939208039 | 379 | 379 |
| ATP10A | 0.13336088165578 | 0.00934130791567971 | 0.0347243069651476 | 379 | 379 |
| ATP10B | -0.219178296267262 | 0.0000166749968234657 | 0.000256303749624556 | 379 | 379 |
| ATP10D | 0.0772957046889261 | 0.133080530500573 | 0.257561811494911 | 379 | 379 |
| ATP11A | -0.0914419835945444 | 0.0753986121076893 | 0.169713211587157 | 379 | 379 |
| ATP11B | -0.126922180501197 | 0.0134075167001117 | 0.045749198234027 | 379 | 379 |
| ATP11C | 0.137314408014368 | 0.00742650569225331 | 0.0291705140384308 | 379 | 379 |
| ATP12A | -0.0244647733625312 | 0.634946535024425 | 0.759102797495587 | 379 | 237 |
| ATP13A1 | 0.115919882550104 | 0.0240156859291343 | 0.0715007260565085 | 379 | 379 |
| ATP13A2 | 0.101813605961057 | 0.0476247135354112 | 0.120554426143257 | 379 | 379 |
| ATP13A3 | -0.0939170291077532 | 0.0677947515269508 | 0.156542952518502 | 379 | 379 |
| ATP13A4 | -0.081218994770494 | 0.114439066306282 | 0.230503748086693 | 379 | 358 |
| ATP13A5 | -0.000586393712281775 | 0.990921737951669 | 0.995137571925937 | 379 | 44 |
| ATP1A1 | -0.185822307080912 | 0.000275453883571092 | 0.00221839951399172 | 379 | 379 |
| ATP1A2 | -0.0603581416929794 | 0.241102619911039 | 0.393269393517282 | 379 | 363 |
| ATP1A3 | 0.140275854361913 | 0.00623052261018463 | 0.0255403767448296 | 379 | 379 |
| ATP1A4 | 0.0304481935541842 | 0.554559215244536 | 0.697273647141303 | 379 | 178 |
| ATP1B1 | -0.0696804214919385 | 0.175832732339746 | 0.31468647141732 | 379 | 379 |
| ATP1B2 | 0.0330968621048671 | 0.520629839323942 | 0.66833418818532 | 379 | 377 |
| ATP1B3 | -0.177076249523968 | 0.000533539050507192 | 0.00371976522273439 | 379 | 379 |
| ATP1B4 | 0.0332754195876748 | 0.518381455094077 | 0.666394417246198 | 379 | 15 |
| ATP2A1 | 0.174838527612347 | 0.000628817978221215 | 0.00425681217895878 | 379 | 378 |
| ATP2A2 | 0.17836835101444 | 0.000484812629696046 | 0.00344053859041274 | 379 | 379 |
| ATP2A3 | -0.052722373166296 | 0.305969040600725 | 0.465241881674169 | 379 | 379 |
| ATP2B1 | 0.231112741314049 | 5.46449488178687E-06 | 0.000108567138793657 | 379 | 379 |
| ATP2B2 | 0.0959689031359174 | 0.061977892506969 | 0.146471710682739 | 379 | 287 |
| ATP2B3 | 0.0845952437575058 | 0.100093096584022 | 0.209656392958519 | 379 | 179 |
| ATP2B4 | 0.0599051541629017 | 0.244658435933664 | 0.397793150282303 | 379 | 379 |
| ATP2C1 | -0.0826425612102153 | 0.108205078536177 | 0.221458536046172 | 379 | 379 |
| ATP2C2 | -0.2334489611507 | 4.36132243193217E-06 | 0.0000912476256958835 | 379 | 379 |
| ATP4A | 0.0613438158409409 | 0.233491021847239 | 0.384587139158253 | 379 | 86 |
| ATP4B | -0.10135437272432 | 0.0486414095392634 | 0.122362581621989 | 379 | 159 |
| ATP5A1 | 0.203994363693482 | 0.0000632429939005046 | 0.00071196161135464 | 379 | 379 |
| ATP5B | 0.137702748669478 | 0.00725879270156267 | 0.02870509407391 | 379 | 379 |
| ATP5C1 | -0.0992576859408408 | 0.0535166626937107 | 0.131371097281981 | 379 | 379 |
| ATP5D | 0.0909276406064539 | 0.0770627279857036 | 0.172343759361666 | 379 | 379 |
| ATP5EP2 | -0.138229645535425 | 0.00703665556087229 | 0.0280673519334089 | 379 | 379 |
| ATP5E | -0.24619933047421 | 0.0000012219592547349 | 0.0000348147024604165 | 379 | 379 |
| ATP5F1 | -0.0287574133279852 | 0.576767046128936 | 0.71399993698224 | 379 | 379 |
| ATP5G1 | 0.0386980593503168 | 0.452554136646188 | 0.607463467063629 | 379 | 379 |
| ATP5G2 | -0.0690575243390561 | 0.179736183586721 | 0.31942359488729 | 379 | 379 |
| ATP5G3 | -0.0134659887013818 | 0.793860604462174 | 0.873826105390064 | 379 | 379 |
| ATP5H | 0.0128276206955801 | 0.80342848574064 | 0.880082869193161 | 379 | 379 |
| ATP5I | 0.00702992612767725 | 0.891499005763287 | 0.937404798550907 | 379 | 379 |
| ATP5J2 | -0.0478984793175976 | 0.352403405023448 | 0.513698380249589 | 379 | 379 |
| ATP5J | 0.00707076931572808 | 0.890872543982084 | 0.93685206618604 | 379 | 379 |
| ATP5L2 | 0.00996527009188697 | 0.846671494654836 | 0.90897192029975 | 379 | 262 |
| ATP5L | 0.029412818140959 | 0.568109081208665 | 0.707540431406564 | 379 | 379 |
| ATP5O | -0.061440403621598 | 0.232754381725948 | 0.383692540809952 | 379 | 379 |
| ATP5SL | 0.107730094338361 | 0.0360415591830226 | 0.0976540086746339 | 379 | 379 |
| ATP5S | -0.0989980273067129 | 0.0541477786592233 | 0.13263028477518 | 379 | 379 |
| ATP6AP1L | -0.183263367494062 | 0.000335284988900367 | 0.0025727673219491 | 379 | 379 |
| ATP6AP1 | 0.0488511384372119 | 0.342897804924182 | 0.50442780571676 | 379 | 379 |
| ATP6AP2 | -0.0764064474677485 | 0.137614690723113 | 0.263829955581225 | 379 | 379 |
| ATP6V0A1 | 0.0329329952340301 | 0.522697635048041 | 0.670191447030313 | 379 | 379 |
| ATP6V0A2 | 0.00388548562300966 | 0.939902221542163 | 0.965565579438268 | 379 | 379 |
| ATP6V0A4 | 0.111282394905194 | 0.0303102215503231 | 0.0856112639458412 | 379 | 242 |
| ATP6V0B | 0.103886193834929 | 0.0432549459469769 | 0.112097643214829 | 379 | 379 |
| ATP6V0C | 0.113059780981886 | 0.0277480874650117 | 0.0801557514942093 | 379 | 379 |
| ATP6V0D1 | 0.00537447187422213 | 0.916943225550937 | 0.952497338273511 | 379 | 379 |
| ATP6V0D2 | 0.140813486612275 | 0.0060329404691991 | 0.0248692606285405 | 379 | 375 |
| ATP6V0E1 | -0.127746778377554 | 0.0128119190919006 | 0.044249212986275 | 379 | 379 |
| ATP6V0E2 | -0.16897920950488 | 0.000957970470924366 | 0.00594530423542406 | 379 | 379 |
| ATP6V1A | -0.109051506615678 | 0.0338103013377741 | 0.0930581142317304 | 379 | 379 |
| ATP6V1B1 | 0.119078345132976 | 0.0204055258090507 | 0.0631695184608676 | 379 | 370 |
| ATP6V1B2 | 0.143899107467243 | 0.0050038617937537 | 0.0214522317073618 | 379 | 379 |
| ATP6V1C1 | -0.0497759514955412 | 0.333827729348822 | 0.494851690903742 | 379 | 379 |
| ATP6V1C2 | -0.0876690480786035 | 0.0883101945181196 | 0.191535171396333 | 379 | 379 |
| ATP6V1D | 0.0529735779372435 | 0.303667040600316 | 0.462668903776952 | 379 | 379 |
| ATP6V1E1 | 0.0379616235139149 | 0.461208450610932 | 0.614903251661751 | 379 | 379 |
| ATP6V1E2 | -0.0265424093659579 | 0.606476508915697 | 0.736388010947976 | 379 | 379 |
| ATP6V1F | 0.0202224289226618 | 0.694741713400517 | 0.801964178454064 | 379 | 379 |
| ATP6V1G1 | -0.139844114890009 | 0.00639336847755218 | 0.026067799747667 | 379 | 379 |
| ATP6V1G2 | 0.0213706336349124 | 0.678350589793658 | 0.790359354473419 | 379 | 377 |
| ATP6V1G3 | 0.0254836372710141 | 0.620914238515155 | 0.747691456411909 | 379 | 3 |
| ATP6V1H | -0.200677496521878 | 0.0000835576489068102 | 0.000877067793819075 | 379 | 379 |
| ATP7A | -0.0134971827656867 | 0.793393844972267 | 0.873697778450161 | 379 | 379 |
| ATP7B | -0.182760054635266 | 0.000348394788758982 | 0.0026487622206722 | 379 | 379 |
| ATP8A1 | 0.00456368583326713 | 0.9294375824536 | 0.959987934827837 | 379 | 379 |
| ATP8A2 | 0.00774754407428197 | 0.880502190312872 | 0.930083502718215 | 379 | 351 |
| ATP8B1 | 0.0183841613138393 | 0.721279443625689 | 0.82209052829106 | 379 | 379 |
| ATP8B2 | 0.124760074779843 | 0.0150863720033965 | 0.050153507603285 | 379 | 379 |
| ATP8B3 | 0.263300835242449 | 1.98169960018431E-07 | 8.37766313695494E-06 | 379 | 379 |
| ATP8B4 | 0.155606522888534 | 0.0023826924339012 | 0.0120091574934908 | 379 | 379 |
| ATP8B5P | 0.0163210082139359 | 0.751465420145919 | 0.843813362252423 | 379 | 379 |
| ATP9A | -0.386059262411769 | 6.430115213488E-15 | 1.593704055663E-11 | 379 | 379 |
| ATP9B | 0.208958706554465 | 0.0000413324107817729 | 0.000512236311394607 | 379 | 379 |
| ATPAF1 | -0.0827554507183832 | 0.107722406798567 | 0.220766912868423 | 379 | 379 |
| ATPAF2 | 0.0880804383962897 | 0.0868213925797937 | 0.189216813813162 | 379 | 379 |
| ATPBD4 | -0.0373787928332578 | 0.468122442926432 | 0.621155845435675 | 379 | 379 |
| ATPIF1 | 0.0757505566340419 | 0.141034291573833 | 0.268647083891572 | 379 | 379 |
| ATRIP | -0.099791191490511 | 0.0522391351623661 | 0.129007578930073 | 379 | 379 |
| ATRNL1 | -0.0192192022585668 | 0.709180833801537 | 0.812858406417531 | 379 | 344 |
| ATRN | 0.046275351468628 | 0.368977381160964 | 0.529847847347132 | 379 | 379 |
| ATRX | -0.0385879130636219 | 0.453842693376452 | 0.608437655461007 | 379 | 379 |
| ATR | -0.157728781794755 | 0.00207133321240093 | 0.0107261412733052 | 379 | 379 |
| ATXN10 | 0.159738041837234 | 0.00181127462640356 | 0.00969363606230526 | 379 | 379 |
| ATXN1L | -0.0655203668601024 | 0.203124522788661 | 0.348615341283957 | 379 | 379 |
| ATXN1 | -0.105390672605913 | 0.0402986732635402 | 0.106269729148753 | 379 | 379 |
| ATXN2L | 0.0386827640481281 | 0.452732947008018 | 0.607566082793569 | 379 | 379 |
| ATXN2 | 0.00228111682289003 | 0.964695582669733 | 0.980922257085921 | 379 | 379 |
| ATXN3L | 0.0501698042409559 | 0.330012264409949 | 0.490736701568956 | 379 | 6 |
| ATXN3 | -0.133140112921374 | 0.00946013683406947 | 0.0350281219693612 | 379 | 379 |
| ATXN7L1 | -0.09410988295834 | 0.0672296470594454 | 0.155582334488175 | 379 | 379 |
| ATXN7L2 | 0.0285211318477507 | 0.579903472152669 | 0.716455159110121 | 379 | 379 |
| ATXN7L3B | -0.03317957351161 | 0.519587720859117 | 0.667344918758997 | 379 | 379 |
| ATXN7L3 | 0.164623052202664 | 0.00129876312609619 | 0.00748818704979216 | 379 | 379 |
| ATXN7 | -0.101313563288611 | 0.0487326292065462 | 0.122529872166802 | 379 | 379 |
| AUH | -0.149816479717455 | 0.00346122082351198 | 0.0161366297880544 | 379 | 379 |
| AUP1 | 0.103731615851431 | 0.0435687748551926 | 0.112704718568658 | 379 | 379 |
| AURKAIP1 | 0.120787116635329 | 0.0186564452467495 | 0.0590360670846711 | 379 | 379 |
| AURKAPS1 | 0.032164575341938 | 0.532449868975966 | 0.678715268534585 | 379 | 379 |
| AURKA | -0.0172100795071382 | 0.738407842285727 | 0.834128724621746 | 379 | 379 |
| AURKB | 0.14656660173211 | 0.0042447130239323 | 0.0189218007730507 | 379 | 379 |
| AURKC | 0.102512802558048 | 0.0461109705942078 | 0.117580382056193 | 379 | 368 |
| AUTS2 | -0.119585539315006 | 0.0198720756042264 | 0.0619533828743084 | 379 | 379 |
| AVEN | -0.136524217596559 | 0.00777849254158526 | 0.0302127560260706 | 379 | 379 |
| AVIL | -0.207407351086693 | 0.0000472595287622405 | 0.00056826072546859 | 379 | 379 |
| AVL9 | -0.0761831628767617 | 0.138771602010395 | 0.265374940940599 | 379 | 379 |
| AVPI1 | 0.0228839052073248 | 0.656978469669369 | 0.774238876469792 | 379 | 379 |
| AVPR1A | 0.202770538076886 | 0.0000701254217823967 | 0.000768202686796333 | 379 | 358 |
| AVPR1B | 0.0486422714859562 | 0.344967776289774 | 0.506555659355227 | 379 | 102 |
| AVPR2 | 0.0853621264400464 | 0.0970424859326106 | 0.205375003850123 | 379 | 378 |
| AVP | 0.0686250602477735 | 0.182483752565885 | 0.322862315430982 | 379 | 5 |
| AWAT1 | 0.084568383925007 | 0.100201308150728 | 0.209842790242146 | 379 | 21 |
| AWAT2 | 0.0740868376920516 | 0.149999980460786 | 0.280955942997965 | 379 | 54 |
| AXIN1 | -0.226961783388521 | 8.11055302790758E-06 | 0.000148166231734429 | 379 | 379 |
| AXIN2 | -0.323149657020693 | 1.16387299642138E-10 | 2.45502912479182E-08 | 379 | 379 |
| AXL | 0.175832463589497 | 0.000584701844110473 | 0.0040013597842975 | 379 | 379 |
| AZGP1 | -0.161503016910799 | 0.00160786813790786 | 0.008848264818883 | 379 | 379 |
| AZI1 | 0.0723720992817454 | 0.15968703709661 | 0.294208750376472 | 379 | 379 |
| AZI2 | -0.111900585240856 | 0.0293970228379591 | 0.083603581301069 | 379 | 379 |
| AZIN1 | -0.0504132710499073 | 0.327667791970983 | 0.488092328089599 | 379 | 379 |
| AZU1 | 0.122232361639564 | 0.0172805659425843 | 0.0555420751352832 | 379 | 179 |
| B2M | 0.115233652385313 | 0.0248692405947643 | 0.0735322550720229 | 379 | 379 |
| B3GALNT1 | 0.0227938357586225 | 0.658242955540223 | 0.774946046933354 | 379 | 379 |
| B3GALNT2 | -0.0281393984473609 | 0.584987404457781 | 0.721114719029461 | 379 | 379 |
| B3GALT1 | -0.0745172970518138 | 0.147639768560457 | 0.27784750674039 | 379 | 306 |
| B3GALT2 | -0.0840856016013876 | 0.102162203690194 | 0.212691324524272 | 379 | 378 |
| B3GALT4 | 0.0409658227336537 | 0.426486130819685 | 0.583841963676658 | 379 | 379 |
| B3GALT5 | 0.243072711871051 | 1.68035993326522E-06 | 0.0000443650822327335 | 379 | 379 |
| B3GALT6 | 0.131959754419424 | 0.0101185324161195 | 0.0369416793862674 | 379 | 379 |
| B3GALTL | -0.0742333042525993 | 0.149193707167631 | 0.280024411270774 | 379 | 379 |
| B3GAT1 | 0.261867517438055 | 2.31973547008716E-07 | 9.50324688034881E-06 | 379 | 374 |
| B3GAT2 | -0.00621885974342335 | 0.903952789148816 | 0.944788145234449 | 379 | 378 |
| B3GAT3 | 0.145892140457962 | 0.00442613572147661 | 0.019493873630706 | 379 | 379 |
| B3GNT1 | 0.0875402197402885 | 0.0887806103171816 | 0.192219037057117 | 379 | 379 |
| B3GNT2 | 0.187769111673338 | 0.000236780855956203 | 0.00197098690675886 | 379 | 379 |
| B3GNT3 | -0.161086141946878 | 0.00165392677189472 | 0.00903168824927801 | 379 | 379 |
| B3GNT4 | 0.228269724770225 | 7.16731338822532E-06 | 0.000134832533075647 | 379 | 379 |
| B3GNT5 | 0.032155020034224 | 0.532571713054314 | 0.678783308249723 | 379 | 379 |
| B3GNT6 | -0.018625562396392 | 0.717774567325291 | 0.819675984618204 | 379 | 345 |
| B3GNT7 | 0.0567369770402374 | 0.270552865179873 | 0.42626318719003 | 379 | 379 |
| B3GNT8 | -0.193430505021276 | 0.000151196127294324 | 0.00137898657405329 | 379 | 379 |
| B3GNT9 | 0.0916075424945445 | 0.0748692175873516 | 0.168809056893565 | 379 | 379 |
| B3GNTL1 | 0.0900444611594628 | 0.0799896426810826 | 0.177567693134853 | 379 | 379 |
| B4GALNT1 | 0.108092555452802 | 0.0354174221679536 | 0.0963711605250698 | 379 | 379 |
| B4GALNT2 | 0.00582117324604255 | 0.910067999449816 | 0.948180773111815 | 379 | 291 |
| B4GALNT3 | -0.194041383817687 | 0.000143941622520907 | 0.00132522493700404 | 379 | 379 |
| B4GALNT4 | -0.0974889808691348 | 0.0579391992381371 | 0.139316319418347 | 379 | 372 |
| B4GALT1 | 0.0761015916418276 | 0.139196105373528 | 0.265894063328161 | 379 | 379 |
| B4GALT2 | 0.135268737980277 | 0.00836841786007964 | 0.0319585880835245 | 379 | 379 |
| B4GALT3 | -0.0363409216329143 | 0.480574820683704 | 0.632430977932999 | 379 | 379 |
| B4GALT4 | -0.00275128289524549 | 0.957425204926056 | 0.976835801341661 | 379 | 379 |
| B4GALT5 | -0.0879073356504381 | 0.0874453650785116 | 0.190179521638338 | 379 | 379 |
| B4GALT6 | 0.0882149424149356 | 0.0863390303284729 | 0.188537926647837 | 379 | 379 |
| B4GALT7 | -0.106271810497011 | 0.0386478563729009 | 0.103040163528557 | 379 | 379 |
| B9D1 | 0.110519782646046 | 0.0314701117228746 | 0.0880470403896088 | 379 | 379 |
| B9D2 | 0.122898360896013 | 0.0166769466262075 | 0.0540487900791832 | 379 | 379 |
| BAALC | 0.122268314333235 | 0.0172474999032967 | 0.0554807638031421 | 379 | 373 |
| BAAT | 0.086623002788691 | 0.0921882216195436 | 0.197889797366278 | 379 | 370 |
| BACE1 | -0.0451876392446537 | 0.380349882178366 | 0.540305019618329 | 379 | 379 |
| BACE2 | -0.116415654630794 | 0.0234148818756938 | 0.0701526560639553 | 379 | 379 |
| BACH1 | 0.101288325128386 | 0.0487891145965175 | 0.122625725976356 | 379 | 379 |
| BACH2 | 0.14209540806423 | 0.00558432853093702 | 0.0234340880659088 | 379 | 379 |
| BAD | 0.0250589223313797 | 0.626747323974894 | 0.752521856592843 | 379 | 379 |
| BAG1 | -0.0987284160219585 | 0.054809619166569 | 0.133871043216892 | 379 | 379 |
| BAG2 | 0.315347940936885 | 0.0000000003389304114 | 6.09955951484384E-08 | 379 | 379 |
| BAG3 | 0.147504670245807 | 0.00400354839736976 | 0.0180784235078678 | 379 | 379 |
| BAG4 | 0.146847416291195 | 0.00417117832628416 | 0.0186358999219384 | 379 | 379 |
| BAG5 | -0.0122885789467212 | 0.811530635270662 | 0.885857026318269 | 379 | 379 |
| BAGE2 | -0.0172483452709715 | 0.737847494940541 | 0.833823962943043 | 379 | 348 |
| BAGE | 0.107359686877063 | 0.0366890040588616 | 0.0989614640233866 | 379 | 43 |
| BAHCC1 | 0.166798897520083 | 0.00111662283621037 | 0.00671735363967816 | 379 | 379 |
| BAHD1 | -0.025968126054958 | 0.614289096324926 | 0.743007637523982 | 379 | 379 |
| BAI1 | 0.016451148667721 | 0.749549542732778 | 0.842377619073033 | 379 | 368 |
| BAI2 | 0.194273851726182 | 0.000141267719765658 | 0.00130810819536803 | 379 | 379 |
| BAI3 | 0.0812701329484635 | 0.114210352945988 | 0.230128187766846 | 379 | 298 |
| BAIAP2L1 | -0.167184220215202 | 0.00108693238365531 | 0.00658469150721583 | 379 | 379 |
| BAIAP2L2 | -0.0321714314475035 | 0.532362452314147 | 0.678715268534585 | 379 | 379 |
| BAIAP2 | 0.107514931137591 | 0.0364164570912827 | 0.0984142716649793 | 379 | 379 |
| BAIAP3 | 0.00613313923019809 | 0.905270443216907 | 0.94521865972116 | 379 | 379 |
| BAK1 | 0.123093554119519 | 0.0165035666267446 | 0.0536532119532744 | 379 | 379 |
| BAMBI | -0.0196608619705429 | 0.702810973808833 | 0.80817351903274 | 379 | 379 |
| BANF1 | 0.114052909592516 | 0.0263991887075012 | 0.0770789447345506 | 379 | 379 |
| BANF2 | -0.00565981167596704 | 0.912550785204751 | 0.949719555376852 | 379 | 85 |
| BANK1 | -0.00904958405675024 | 0.860609573941203 | 0.918020584899192 | 379 | 379 |
| BANP | 0.102047282332613 | 0.0471142462447029 | 0.119552249109287 | 379 | 379 |
| BAP1 | -0.231777634598418 | 5.12604006123644E-06 | 0.000103291790990037 | 379 | 379 |
| BARD1 | 0.0476753755992315 | 0.354653306998412 | 0.515774224084239 | 379 | 379 |
| BARHL1 | -0.019960229935224 | 0.698505079571066 | 0.805089735379591 | 379 | 52 |
| BARHL2 | -0.0293907517976889 | 0.568399569812439 | 0.707662104121628 | 379 | 28 |
| BARX1 | 0.0145861372297146 | 0.777147039774714 | 0.862781159275086 | 379 | 304 |
| BARX2 | 0.242514740219374 | 1.77785228874695E-06 | 0.000046370476911658 | 379 | 379 |
| BASP1 | 0.122400319018296 | 0.0171265675107994 | 0.0551633496757845 | 379 | 379 |
| BAT1 | 0.0137391013036087 | 0.78977651122683 | 0.871580601358356 | 379 | 379 |
| BAT2L1 | 0.00485210237042435 | 0.924990886035234 | 0.957343558317501 | 379 | 379 |
| BAT2L2 | -0.0143022306277683 | 0.781373792192897 | 0.86574667691519 | 379 | 379 |
| BAT2 | -0.0107870990015536 | 0.834202481438511 | 0.900509952197452 | 379 | 379 |
| BAT3 | -0.0213527411573415 | 0.678604878088079 | 0.790562721664537 | 379 | 379 |
| BAT4 | -0.0671722601305882 | 0.191940878628392 | 0.334958963337771 | 379 | 379 |
| BAT5 | 0.108403159406173 | 0.0348899117010981 | 0.0953282581244831 | 379 | 379 |
| BATF2 | 0.173611349538557 | 0.000687538798715473 | 0.00458390023568608 | 379 | 379 |
| BATF3 | 0.217407760665497 | 0.0000195758375601172 | 0.000290096941062784 | 379 | 379 |
| BATF | 0.157584165309685 | 0.00209130741232564 | 0.0108126319091507 | 379 | 379 |
| BAX | 0.0245066266595682 | 0.634367484681589 | 0.758580377755407 | 379 | 379 |
| BAZ1A | -0.0231325870580686 | 0.653492307343519 | 0.771728456901385 | 379 | 379 |
| BAZ1B | 0.128095340491942 | 0.012567251256638 | 0.0436169189421702 | 379 | 379 |
| BAZ2A | 0.111490156860312 | 0.0300006411420553 | 0.0849535131795442 | 379 | 379 |
| BAZ2B | -0.19692822355148 | 0.000113864169939907 | 0.00110454929626637 | 379 | 379 |
| BBC3 | -0.240791217518239 | 2.11436747221959E-06 | 0.0000522739130164214 | 379 | 379 |
| BBOX1 | 0.0506799421929966 | 0.3251122582714 | 0.485562357412271 | 379 | 225 |
| BBS10 | -0.119719247601043 | 0.0197334816679693 | 0.0616375983794102 | 379 | 379 |
| BBS12 | 0.0366090119202272 | 0.477341157373296 | 0.629428773585004 | 379 | 379 |
| BBS1 | -0.084963297615904 | 0.0986196292623727 | 0.207670986513841 | 379 | 379 |
| BBS2 | -0.177301617354061 | 0.000524725540382303 | 0.00367122724583638 | 379 | 379 |
| BBS4 | 0.19123204167576 | 0.000180240276130742 | 0.00159158874218455 | 379 | 379 |
| BBS5 | -0.262079684694512 | 2.26641228823227E-07 | 9.38171667036942E-06 | 379 | 379 |
| BBS7 | 0.182319475194994 | 0.000360260974266819 | 0.00271916809964313 | 379 | 379 |
| BBS9 | -0.133142428436247 | 0.00945888357056471 | 0.0350281219693612 | 379 | 379 |
| BBX | 0.0300967399607528 | 0.559140966464044 | 0.700666566583396 | 379 | 379 |
| BCAM | 0.00658324637342424 | 0.898354597760523 | 0.941567552827765 | 379 | 379 |
| BCAN | 0.00576404113157185 | 0.910946962542271 | 0.948601232206869 | 379 | 379 |
| BCAP29 | -0.0136147171689189 | 0.791635830834652 | 0.872515578309588 | 379 | 379 |
| BCAP31 | 0.0923735453568899 | 0.0724590564530147 | 0.164792131213011 | 379 | 379 |
| BCAR1 | 0.181132890772577 | 0.000394117785751494 | 0.0029246135688176 | 379 | 379 |
| BCAR3 | 0.0720330016460909 | 0.161657304545746 | 0.29681831970859 | 379 | 379 |
| BCAR4 | 0.00395146571077624 | 0.93888366468504 | 0.964891558737841 | 379 | 86 |
| BCAS1 | -0.0930387294727009 | 0.070417786090637 | 0.161303588563441 | 379 | 379 |
| BCAS2 | 0.0465061550464374 | 0.366591602026188 | 0.527608610866546 | 379 | 379 |
| BCAS3 | -0.140473635465141 | 0.00615717242312035 | 0.0252815106244834 | 379 | 379 |
| BCAS4 | -0.028111308898424 | 0.58536231514595 | 0.721144594318442 | 379 | 379 |
| BCAT1 | 0.198448812187975 | 0.000100502572899004 | 0.00100797421114894 | 379 | 379 |
| BCAT2 | 0.143320724589459 | 0.00518378751787226 | 0.0220727150209028 | 379 | 379 |
| BCCIP | 0.151669319652286 | 0.00307563329861939 | 0.0146877786717305 | 379 | 379 |
| BCDIN3D | 0.0384899466208334 | 0.454990492774869 | 0.609440754626771 | 379 | 379 |
| BCHE | 0.0911713928373172 | 0.0762704075494648 | 0.171049870733115 | 379 | 365 |
| BCKDHA | 0.0961768523054814 | 0.061412057482443 | 0.145385692900196 | 379 | 379 |
| BCKDHB | -0.105120139121337 | 0.0408173179206903 | 0.107309172597646 | 379 | 379 |
| BCKDK | 0.0782931013736001 | 0.128132995711872 | 0.250529636029483 | 379 | 379 |
| BCL10 | 0.0307078035174268 | 0.551186576750442 | 0.694841286945958 | 379 | 379 |
| BCL11A | -0.279262528535561 | 3.21900931792836E-08 | 2.18583961492752E-06 | 379 | 379 |
| BCL11B | 0.0027020037386924 | 0.958187074431317 | 0.977110332844279 | 379 | 379 |
| BCL2A1 | 0.254518207601053 | 5.12676625387038E-07 | 0.0000177096726971676 | 379 | 379 |
| BCL2L10 | -0.125913984380311 | 0.0141687407508362 | 0.0478273393952298 | 379 | 357 |
| BCL2L11 | 0.109800398478802 | 0.0325988048040262 | 0.0906294309666617 | 379 | 379 |
| BCL2L12 | 0.18073362112144 | 0.000406160465265115 | 0.00299104474364479 | 379 | 379 |
| BCL2L13 | 0.0144842812925629 | 0.77866270490817 | 0.864028435442341 | 379 | 379 |
| BCL2L14 | 0.038019267931829 | 0.460527729862482 | 0.614367478147972 | 379 | 379 |
| BCL2L15 | -0.0575735907000386 | 0.263539877525564 | 0.418430596924762 | 379 | 379 |
| BCL2L1 | -0.109124815565728 | 0.0336900389586587 | 0.0927915116644373 | 379 | 379 |
| BCL2L2 | -0.0268274363363888 | 0.602615485849078 | 0.734200582818972 | 379 | 379 |
| BCL2 | 0.00617678709035473 | 0.904599476833378 | 0.945043022993739 | 379 | 379 |
| BCL3 | 0.168862211129238 | 0.000965925875235278 | 0.00598137984202532 | 379 | 379 |
| BCL6B | 0.168426889974989 | 0.000996064126449051 | 0.00612781864698473 | 379 | 379 |
| BCL6 | 0.147339290906337 | 0.00404514410898007 | 0.0182330341879647 | 379 | 379 |
| BCL7A | -0.0265973135986252 | 0.60573191072084 | 0.735939520132573 | 379 | 379 |
| BCL7B | 0.122794251825061 | 0.0167700710863152 | 0.0542973496896567 | 379 | 379 |
| BCL7C | -0.068624822459338 | 0.182485271789138 | 0.322862315430982 | 379 | 379 |
| BCL8 | 0.0522518998593877 | 0.310311303787566 | 0.469839316524573 | 379 | 222 |
| BCL9L | 0.242025178877567 | 1.86782999793296E-06 | 0.0000475421478806352 | 379 | 379 |
| BCL9 | -0.211826932339695 | 0.0000321775054844488 | 0.000421689080466391 | 379 | 379 |
| BCLAF1 | 0.0321602616815061 | 0.532504872683403 | 0.678741746950791 | 379 | 379 |
| BCMO1 | 0.193815526572091 | 0.000146584893288991 | 0.00134441604180097 | 379 | 379 |
| BCO2 | -0.0763400506772688 | 0.137957938008089 | 0.264318291122272 | 379 | 378 |
| BCORL1 | 0.0701952150596978 | 0.172654536988023 | 0.310596457938534 | 379 | 379 |
| BCORL2 | 0.0848874194110937 | 0.0989219801398982 | 0.207977672990894 | 379 | 161 |
| BCOR | -0.0017157157284737 | 0.973442439461997 | 0.985973472090952 | 379 | 379 |
| BCR | 0.107200080421962 | 0.0369710117000434 | 0.099546607820269 | 379 | 379 |
| BCS1L | -0.0376362952175674 | 0.465060720540443 | 0.618044501801334 | 379 | 379 |
| BCYRN1 | -0.06669083528591 | 0.195152608916783 | 0.338745157104262 | 379 | 19 |
| BDH1 | -0.212057353569457 | 0.0000315320745744097 | 0.00041460077895318 | 379 | 379 |
| BDH2 | -0.225826160008106 | 9.02431066987075E-06 | 0.000161638691926104 | 379 | 379 |
| BDKRB1 | 0.123087535783419 | 0.0165088887495928 | 0.0536532119532744 | 379 | 379 |
| BDKRB2 | 0.10029100245569 | 0.0510654380259599 | 0.126866997265848 | 379 | 379 |
| BDNFOS | -0.257430819657939 | 3.75502131659985E-07 | 0.0000138649092487043 | 379 | 378 |
| BDNF | 0.0342265939573632 | 0.506489128177033 | 0.655869019951294 | 379 | 378 |
| BDP1 | -0.0449599820770065 | 0.382757004365877 | 0.542674714520316 | 379 | 379 |
| BEAN | -0.0347035291015377 | 0.500580351025222 | 0.650534008189825 | 379 | 378 |
| BECN1 | -0.109458422441475 | 0.0331473529209241 | 0.0916941010441934 | 379 | 379 |
| BEGAIN | 0.0874651049795881 | 0.0890558182116007 | 0.192772790775067 | 379 | 379 |
| BEND2 | 0.0122417408559304 | 0.812235606034865 | 0.886253994962541 | 379 | 23 |
| BEND3 | 0.0240336284086011 | 0.640924427262917 | 0.763716919697664 | 379 | 379 |
| BEND4 | 0.151222773114636 | 0.00316481440879305 | 0.0150016591196626 | 379 | 320 |
| BEND5 | 0.046831851931325 | 0.363241267907253 | 0.524570128191188 | 379 | 372 |
| BEND6 | 0.133697290598067 | 0.00916278539452058 | 0.0341780949383671 | 379 | 375 |
| BEND7 | -0.342883503725213 | 6.77539856803449E-12 | 2.53416028682609E-09 | 379 | 379 |
| BEST1 | 0.110093012804078 | 0.032135601770122 | 0.0895425396146682 | 379 | 379 |
| BEST2 | -0.0656044637738236 | 0.202543990620279 | 0.347859874059669 | 379 | 307 |
| BEST3 | -0.0899757023959028 | 0.0802212388141359 | 0.178041943497502 | 379 | 314 |
| BEST4 | 0.0448069268264236 | 0.384380541839916 | 0.544276039677344 | 379 | 376 |
| BET1L | -0.0123818181028899 | 0.810127725008631 | 0.884830479865106 | 379 | 379 |
| BET1 | -0.0539875213160846 | 0.294492189311701 | 0.452861108242004 | 379 | 379 |
| BET3L | 0.0972769245566688 | 0.0584891846448632 | 0.140334408656625 | 379 | 199 |
| BEX1 | 0.00114501390231763 | 0.982274523549832 | 0.989867023729023 | 379 | 315 |
| BEX2 | -0.229587258382119 | 6.32336407114062E-06 | 0.000121491921320326 | 379 | 368 |
| BEX4 | -0.0976426446354788 | 0.0575433403836802 | 0.13860171928178 | 379 | 379 |
| BEX5 | -0.0970062829311205 | 0.0591973824308349 | 0.141622309222803 | 379 | 368 |
| BFAR | -0.198127021518728 | 0.000103200830186077 | 0.00102211091954522 | 379 | 379 |
| BFSP1 | -0.10963940619492 | 0.0328560728748563 | 0.091076501183091 | 379 | 379 |
| BFSP2 | 0.121778798445888 | 0.0177024926096656 | 0.0566030637412083 | 379 | 269 |
| BGLAP | -0.0346101231812791 | 0.501734688664801 | 0.651627392863409 | 379 | 379 |
| BGN | 0.164672604113758 | 0.00129432779631025 | 0.00746695709782937 | 379 | 379 |
| BHLHA15 | 0.152759511504807 | 0.00286741675632932 | 0.0139243856132413 | 379 | 375 |
| BHLHB9 | -0.149224585472871 | 0.00359333253706004 | 0.0166313252905757 | 379 | 379 |
| BHLHE22 | 0.0356885658027482 | 0.488492852171772 | 0.639974098544926 | 379 | 379 |
| BHLHE23 | 0.0765613492469295 | 0.136816454880202 | 0.262664278404787 | 379 | 5 |
| BHLHE40 | 0.0364028981142437 | 0.479826215195953 | 0.631739322370873 | 379 | 379 |
| BHLHE41 | 0.080096002928303 | 0.119552657893997 | 0.238001014128732 | 379 | 379 |
| BHMT2 | 0.0603278580405125 | 0.241339200890561 | 0.393545935214667 | 379 | 375 |
| BHMT | -0.0138325263596091 | 0.788380757228144 | 0.870234017386831 | 379 | 248 |
| BICC1 | 0.0377348568624865 | 0.463891756776246 | 0.617017468261734 | 379 | 379 |
| BICD1 | 0.266839471342282 | 1.33792670184723E-07 | 6.30128518865248E-06 | 379 | 379 |
| BICD2 | 0.00653556255232571 | 0.899086899563661 | 0.941737720261398 | 379 | 379 |
| BID | -0.0553326042686558 | 0.282609405126548 | 0.440048632389602 | 379 | 379 |
| BIK | 0.0981244409596566 | 0.0563166625539668 | 0.136492700784752 | 379 | 379 |
| BIN1 | -0.0461445791268847 | 0.370333412751308 | 0.53091672459857 | 379 | 379 |
| BIN2 | 0.19264928187736 | 0.00016097354124966 | 0.0014587675392588 | 379 | 379 |
| BIN3 | 0.0806377036665652 | 0.117064172034797 | 0.234435754277947 | 379 | 379 |
| BIRC2 | 0.0342379192899852 | 0.506348395817766 | 0.655815271557558 | 379 | 379 |
| BIRC3 | 0.29741256030203 | 3.52585932992313E-09 | 0.00000040202573206 | 379 | 379 |
| BIRC5 | 0.206055645989267 | 0.0000530697605907491 | 0.000618980713525514 | 379 | 379 |
| BIRC6 | -0.017640220587172 | 0.732117074366416 | 0.830315240847568 | 379 | 379 |
| BIRC7 | 0.108938349150067 | 0.0339966523650188 | 0.0934118900782369 | 379 | 368 |
| BIRC8 | 0.0467271269930756 | 0.36431645034726 | 0.525472217755544 | 379 | 3 |
| BIVM | -0.26998559210461 | 9.39001844130059E-08 | 0.0000048109892933878 | 379 | 379 |
| BLCAP | -0.132113183270958 | 0.0100307138811404 | 0.0366818507626802 | 379 | 379 |
| BLID | 0.0349209696085349 | 0.497898599789217 | 0.648632124707221 | 379 | 12 |
| BLK | 0.0965812683264238 | 0.060323829481349 | 0.14369304312304 | 379 | 344 |
| BLMH | 0.186069022082865 | 0.000270245735229504 | 0.00219158790925587 | 379 | 379 |
| BLM | 0.189138370845288 | 0.000212687668145624 | 0.0018022098649536 | 379 | 379 |
| BLNK | -0.0953816219759155 | 0.0635991415967087 | 0.149306627939799 | 379 | 379 |
| BLOC1S1 | 0.012206732702007 | 0.812762620770945 | 0.886487554026421 | 379 | 379 |
| BLOC1S2 | 0.23446648441149 | 3.95044598819851E-06 | 0.0000847743515668264 | 379 | 379 |
| BLOC1S3 | -0.00687340314115055 | 0.893900410944683 | 0.938858625743769 | 379 | 379 |
| BLVRA | 0.245169343991686 | 1.35780371333878E-06 | 0.0000375488591744509 | 379 | 379 |
| BLVRB | 0.101583826698749 | 0.0481311751851941 | 0.121438999135893 | 379 | 379 |
| BLZF1 | 0.0228216002876709 | 0.657853065014335 | 0.774808013686761 | 379 | 379 |
| BMF | -0.0908040758001914 | 0.0774669218484227 | 0.173072014243439 | 379 | 379 |
| BMI1 | -0.0296246157524302 | 0.565324511671739 | 0.705453175916651 | 379 | 379 |
| BMP10 | 0.0445556339048849 | 0.387055221313751 | 0.546385513897839 | 379 | 22 |
| BMP15 | -0.0579860182374156 | 0.260129043138407 | 0.414317508823868 | 379 | 21 |
| BMP1 | 0.133828567601972 | 0.0090939474319505 | 0.0339767834333361 | 379 | 379 |
| BMP2K | 0.0942764700491251 | 0.0667446236835995 | 0.15468252292597 | 379 | 379 |
| BMP2 | 0.157200441649899 | 0.0021451625584659 | 0.0110371368202937 | 379 | 379 |
| BMP3 | -0.0699462776685207 | 0.174186024614344 | 0.312585799262667 | 379 | 350 |
| BMP4 | 0.0142899620938467 | 0.781556590064454 | 0.865783739726839 | 379 | 379 |
| BMP5 | -0.164733377188888 | 0.00128890708653103 | 0.00744868834501232 | 379 | 327 |
| BMP6 | 0.0584661485546778 | 0.256196782579601 | 0.410507875599792 | 379 | 379 |
| BMP7 | -0.00903146485588435 | 0.860885826385241 | 0.918216469368831 | 379 | 379 |
| BMP8A | 0.0965524924752672 | 0.0604007315916291 | 0.143791087380815 | 379 | 379 |
| BMP8B | 0.106306480501486 | 0.038584089898545 | 0.102925512512895 | 379 | 379 |
| BMPER | -0.149788242361827 | 0.00346742203169144 | 0.0161541456871189 | 379 | 376 |
| BMPR1A | -0.145388772026144 | 0.00456606603138311 | 0.0199567503713343 | 379 | 379 |
| BMPR1B | 0.0399083700728577 | 0.438531508154639 | 0.595170074952131 | 379 | 362 |
| BMPR2 | -0.121097748573745 | 0.0183529238541282 | 0.0583269392818808 | 379 | 379 |
| BMS1P4 | -0.0500257917911661 | 0.331404116827347 | 0.492202908356297 | 379 | 379 |
| BMS1P5 | -0.0271281430818152 | 0.598554056658744 | 0.730885454760556 | 379 | 379 |
| BMS1 | 0.206661094843418 | 0.0000503885103007871 | 0.000596123736422438 | 379 | 379 |
| BMX | -0.0930859805306884 | 0.0702745910392862 | 0.161049998974453 | 379 | 377 |
| BNC1 | 0.131933535119969 | 0.0101336076640281 | 0.0369710728876605 | 379 | 218 |
| BNC2 | 0.0675032526143943 | 0.189755298526368 | 0.332197427086422 | 379 | 379 |
| BNIP1 | 0.0939418885170946 | 0.0677216901899831 | 0.156441761976942 | 379 | 379 |
| BNIP2 | 0.135195454194803 | 0.00840405253349722 | 0.0320452987758044 | 379 | 379 |
| BNIP3L | 0.181409200853173 | 0.000385979253926526 | 0.00287282156413482 | 379 | 379 |
| BNIP3 | -0.112768851027993 | 0.0281542801233208 | 0.0809517207490147 | 379 | 379 |
| BNIPL | -0.0572688373604579 | 0.266079893156139 | 0.421325037653723 | 379 | 374 |
| BOC | 0.0394856258064088 | 0.443400923459392 | 0.599056521555794 | 379 | 379 |
| BOD1L | -0.0300432725123295 | 0.559839599713884 | 0.701231811947371 | 379 | 379 |
| BOD1 | -0.118654157012694 | 0.0208611832838074 | 0.0642890180527405 | 379 | 379 |
| BOK | 0.137081086333789 | 0.00752892730427326 | 0.0294968525171172 | 379 | 379 |
| BOLA1 | -0.0162753445361518 | 0.752138025609742 | 0.844377350910993 | 379 | 379 |
| BOLA2 | -0.0279848880734506 | 0.587051025882073 | 0.722356195928369 | 379 | 379 |
| BOLA3 | 0.137332308225815 | 0.0074186996570964 | 0.0291514024575718 | 379 | 379 |
| BOLL | 0.0129489966020694 | 0.801606991671797 | 0.878767259958445 | 379 | 127 |
| BOP1 | 0.0078353723845456 | 0.879157836139002 | 0.929533018536216 | 379 | 379 |
| BPESC1 | 0.115371171594798 | 0.0246961234791954 | 0.073129440911811 | 379 | 53 |
| BPGM | 0.136135390348471 | 0.0079570992494875 | 0.0307360928571765 | 379 | 379 |
| BPHL | -0.0714410430810948 | 0.165140501894994 | 0.300901109315745 | 379 | 379 |
| BPIL1 | -0.0281963956318915 | 0.58422700591497 | 0.720311719517599 | 379 | 84 |
| BPIL2 | -0.0306556943681626 | 0.551862729271821 | 0.695546287566849 | 379 | 26 |
| BPI | -0.108616043888461 | 0.0345322338417441 | 0.0945072646810355 | 379 | 304 |
| BPNT1 | -0.225609707501311 | 9.20924525036746E-06 | 0.000163988130997326 | 379 | 379 |
| BPTF | -0.0354050206423695 | 0.49195612326562 | 0.643182514315621 | 379 | 379 |
| BPY2 | 0.000397161379203807 | 0.993851269306648 | 0.996418049072748 | 379 | 3 |
| BRAF | -0.0578892450203024 | 0.260926625791898 | 0.415087703482168 | 379 | 379 |
| BRAP | 0.0819590766011024 | 0.11116389830649 | 0.225789569311727 | 379 | 379 |
| BRCA1 | 0.109457872915243 | 0.0331482406841224 | 0.0916941010441934 | 379 | 379 |
| BRCA2 | -0.00584805586429307 | 0.909654455044821 | 0.947998976906797 | 379 | 379 |
| BRCC3 | -0.11212557303463 | 0.0290705702770517 | 0.0828058134540125 | 379 | 379 |
| BRD1 | -0.0317046915173564 | 0.538329951020738 | 0.683880463149615 | 379 | 379 |
| BRD2 | 0.143101426256779 | 0.0052535160348178 | 0.0223007313077215 | 379 | 379 |
| BRD3 | -0.0768865271134047 | 0.135152336874433 | 0.260275887290818 | 379 | 379 |
| BRD4 | 0.0863945525639979 | 0.0930530268860675 | 0.199250045042867 | 379 | 379 |
| BRD7P3 | -0.00684639881482856 | 0.894314815034491 | 0.939070812503516 | 379 | 144 |
| BRD7 | -0.139162603507971 | 0.00665819677477092 | 0.0268603714445896 | 379 | 379 |
| BRD8 | -0.0282361048171332 | 0.583697517243694 | 0.71983793829506 | 379 | 379 |
| BRD9 | 0.0482921503396801 | 0.348455408180064 | 0.510165682152722 | 379 | 379 |
| BRDT | 0.055635391126709 | 0.279979788677817 | 0.437052373634368 | 379 | 56 |
| BREA2 | -0.0826808042090139 | 0.108041375724758 | 0.221174848381583 | 379 | 372 |
| BRE | -0.123835869839241 | 0.0158585258889125 | 0.0521204792516752 | 379 | 379 |
| BRF1 | 0.0186602760901616 | 0.717271047139003 | 0.819384128747604 | 379 | 379 |
| BRF2 | 0.058535745738678 | 0.255630213494863 | 0.409948879869938 | 379 | 379 |
| BRI3BP | 0.242707353855859 | 1.74360124118074E-06 | 0.0000456974212884126 | 379 | 379 |
| BRI3 | -0.0583004777646527 | 0.257548950592213 | 0.41189844307708 | 379 | 379 |
| BRIP1 | 0.0918207399869721 | 0.0741919510720413 | 0.167701551055225 | 379 | 379 |
| BRIX1 | 0.0273712275618084 | 0.59527995924508 | 0.728323524121403 | 379 | 379 |
| BRMS1L | 0.0618507961718012 | 0.229642781843384 | 0.379889627764944 | 379 | 379 |
| BRMS1 | 0.131851617358719 | 0.0101808361360519 | 0.0370940130293342 | 379 | 379 |
| BRP44L | -0.0639880928428779 | 0.213914103504568 | 0.360916341413256 | 379 | 379 |
| BRP44 | -0.210540594317253 | 0.0000360165531133851 | 0.000461494583044768 | 379 | 379 |
| BRPF1 | -0.0752941259953009 | 0.143452101993872 | 0.271928133683986 | 379 | 379 |
| BRPF3 | -0.100589131992918 | 0.0503758767835091 | 0.125641872309864 | 379 | 379 |
| BRS3 | 0.0103893205916398 | 0.840232702778579 | 0.904753667356015 | 379 | 53 |
| BRSK1 | -0.00140481439711118 | 0.978253562167906 | 0.988271851564948 | 379 | 379 |
| BRSK2 | -0.109616782933198 | 0.0328923635647027 | 0.0911516121259155 | 379 | 376 |
| BRWD1 | -0.0188989348692245 | 0.713812637197234 | 0.816861547927629 | 379 | 379 |
| BRWD3 | -0.0165678182785333 | 0.74783328496182 | 0.841116250168641 | 379 | 379 |
| BSCL2 | 0.105610106716604 | 0.0398820908954777 | 0.105445251844909 | 379 | 379 |
| BSDC1 | 0.0154350590726925 | 0.764548036001748 | 0.853957777030344 | 379 | 379 |
| BSG | 0.147665097153626 | 0.00396356827978896 | 0.0179387427189353 | 379 | 379 |
| BSND | 0.0746930590911154 | 0.146684232838185 | 0.276495386321469 | 379 | 112 |
| BSN | 0.000201162644027628 | 0.996885638800324 | 0.997804556718965 | 379 | 379 |
| BSPH1 | -0.0602886004548109 | 0.241646129956715 | 0.393872617051441 | 379 | 7 |
| BSPRY | -0.199289444370836 | 0.000093763365653621 | 0.000959308572848295 | 379 | 379 |
| BST1 | 0.0208031939460969 | 0.686432685423205 | 0.796359913789205 | 379 | 379 |
| BST2 | 0.275656034284528 | 4.90379102623498E-08 | 2.92167971692431E-06 | 379 | 379 |
| BSX | 0.0233786471134618 | 0.650050296556669 | 0.7698761009807 | 379 | 6 |
| BTAF1 | 0.0875648091984872 | 0.0886906671083587 | 0.192045271095832 | 379 | 379 |
| BTBD10 | 0.0522811477852329 | 0.310040182754943 | 0.469631531219633 | 379 | 379 |
| BTBD11 | 0.000473367214689234 | 0.992671504313511 | 0.995936375425203 | 379 | 379 |
| BTBD12 | 0.00901789118004163 | 0.861092787081469 | 0.918338413417134 | 379 | 379 |
| BTBD16 | -0.0278889892987611 | 0.588333524470775 | 0.723395580007846 | 379 | 372 |
| BTBD17 | 0.177225220557142 | 0.000527697952626502 | 0.00368681994527071 | 379 | 130 |
| BTBD18 | -0.0354182135495098 | 0.491794692206525 | 0.643056262006791 | 379 | 302 |
| BTBD19 | 0.0824343239567212 | 0.109099891610766 | 0.222738123029065 | 379 | 378 |
| BTBD1 | 0.0438188977719391 | 0.394961727621064 | 0.554674381238692 | 379 | 379 |
| BTBD2 | -0.0227589115510438 | 0.658733519159974 | 0.775294666862333 | 379 | 379 |
| BTBD3 | 0.0865469797709453 | 0.0924752923560983 | 0.198280561330011 | 379 | 379 |
| BTBD6 | 0.026891814208197 | 0.601744940969 | 0.733517686556826 | 379 | 379 |
| BTBD7 | 0.0115804986521676 | 0.822204036515182 | 0.893072547661369 | 379 | 379 |
| BTBD8 | -0.0503589791186458 | 0.32818966308908 | 0.488649443548117 | 379 | 373 |
| BTBD9 | -0.171316149662214 | 0.000811201063100211 | 0.00520533808386763 | 379 | 379 |
| BTC | -0.0853872974643724 | 0.0969436265114755 | 0.205253414465514 | 379 | 378 |
| BTD | -0.140936509664994 | 0.0059885270909777 | 0.0247415205308329 | 379 | 379 |
| BTF3L1 | -0.0248755847448331 | 0.629272534588439 | 0.754457359766572 | 379 | 123 |
| BTF3L4 | -0.0146790454541366 | 0.775765257118724 | 0.86177788773321 | 379 | 379 |
| BTF3 | -0.11302310589202 | 0.0277990139021216 | 0.0802820447886262 | 379 | 379 |
| BTG1 | 0.0205907414575751 | 0.68946793930967 | 0.798849255687417 | 379 | 379 |
| BTG2 | -0.209722291815388 | 0.0000386800112890379 | 0.000489749210625188 | 379 | 379 |
| BTG3 | 0.0723610598356838 | 0.159750893699827 | 0.294271713143828 | 379 | 379 |
| BTG4 | 0.0591096140442557 | 0.250991583315843 | 0.405034680067269 | 379 | 35 |
| BTK | 0.181447432443569 | 0.000384865568166147 | 0.00286668463020224 | 379 | 379 |
| BTLA | 0.118498305197634 | 0.0210308024415535 | 0.0646594109979966 | 379 | 376 |
| BTN1A1 | 0.241242406191057 | 2.02081513497645E-06 | 0.0000505469295048028 | 379 | 364 |
| BTN2A1 | -0.0544641990111903 | 0.290243509288095 | 0.448651920207125 | 379 | 379 |
| BTN2A2 | 0.00840535587903549 | 0.870441917624421 | 0.924396839415713 | 379 | 379 |
| BTN2A3 | 0.0239104790403597 | 0.642636202598701 | 0.76502105098025 | 379 | 378 |
| BTN3A1 | 0.127303872206148 | 0.013128860700388 | 0.0450297756901847 | 379 | 379 |
| BTN3A2 | 0.137333019650383 | 0.00741838956616972 | 0.0291514024575718 | 379 | 379 |
| BTN3A3 | 0.148887668785514 | 0.00367055717100917 | 0.0169094728706263 | 379 | 379 |
| BTNL2 | 0.107637775039367 | 0.0362020119243902 | 0.0979548979853723 | 379 | 34 |
| BTNL3 | -0.0413000066898478 | 0.422719801951348 | 0.580391601599666 | 379 | 378 |
| BTNL8 | 0.0872664198087571 | 0.0897870682472995 | 0.19395337065121 | 379 | 378 |
| BTNL9 | 0.162534378507278 | 0.00149892580074738 | 0.00836731440800085 | 379 | 379 |
| BTRC | -0.0333835721196993 | 0.517022040516632 | 0.665250682632303 | 379 | 379 |
| BUB1B | 0.117874236600924 | 0.0217220462782663 | 0.0662316982324258 | 379 | 379 |
| BUB1 | 0.223323164741459 | 0.0000113962862404455 | 0.00019133409278201 | 379 | 379 |
| BUB3 | 0.165204938641442 | 0.00124754973371801 | 0.00726686725034097 | 379 | 379 |
| BUD13 | 0.226515482732432 | 8.45862517885759E-06 | 0.00015302702558977 | 379 | 379 |
| BUD31 | 0.0943135847504384 | 0.0666369549531647 | 0.154530108925376 | 379 | 379 |
| BVES | 0.0548652318770599 | 0.286701038786757 | 0.444778045306613 | 379 | 379 |
| BYSL | 0.0296254147175743 | 0.565314019807596 | 0.705453175916651 | 379 | 379 |
| BZRAP1 | -0.104889011462085 | 0.0412648693615309 | 0.108256129888917 | 379 | 379 |
| BZW1 | 0.0903853418816737 | 0.0788494538961771 | 0.175626485267737 | 379 | 379 |
| BZW2 | 0.00552957991580094 | 0.914555214233274 | 0.951056841024669 | 379 | 379 |
| C10orf105 | 0.0535800329667369 | 0.298156935751538 | 0.456897960061969 | 379 | 344 |
| C10orf107 | -0.146306860629123 | 0.0043137697797081 | 0.0191331191627165 | 379 | 339 |
| C10orf108 | -0.27680046583717 | 4.29345232542537E-08 | 2.68550702550581E-06 | 379 | 379 |
| C10orf10 | 0.140717022792381 | 0.00606797201310328 | 0.0249686772224754 | 379 | 379 |
| C10orf110 | -0.0499670353141396 | 0.331973071061405 | 0.492802429662764 | 379 | 378 |
| C10orf111 | -0.0847011794780939 | 0.0996672125661233 | 0.208989159344447 | 379 | 379 |
| C10orf113 | 0.0238527422202477 | 0.64343939600609 | 0.765793927731435 | 379 | 55 |
| C10orf114 | 0.217542092364694 | 0.0000193399622566922 | 0.000287030517683902 | 379 | 379 |
| C10orf116 | -0.0591461799518668 | 0.250698013469647 | 0.404751720106688 | 379 | 379 |
| C10orf118 | -0.042255742709684 | 0.412056129197445 | 0.570555736760037 | 379 | 379 |
| C10orf119 | 0.109072892641009 | 0.0337751801266521 | 0.092985048560463 | 379 | 379 |
| C10orf11 | -0.254578289625579 | 5.09413654603277E-07 | 0.0000176276683132178 | 379 | 377 |
| C10orf120 | -0.0108635535583059 | 0.833044549848722 | 0.899945915571563 | 379 | 10 |
| C10orf122 | -0.00325811094608712 | 0.949591873940543 | 0.971994614449078 | 379 | 6 |
| C10orf125 | 0.219813849433061 | 0.000015737041572043 | 0.000245085366722201 | 379 | 379 |
| C10orf128 | 0.128354648070059 | 0.0123879163741824 | 0.0431076879373971 | 379 | 364 |
| C10orf129 | 0.0628619828575533 | 0.222102051514463 | 0.370660202501534 | 379 | 41 |
| C10orf12 | 0.279065257356223 | 3.29450219712632E-08 | 0.0000021994407260815 | 379 | 379 |
| C10orf131 | 0.105775684460117 | 0.0395701658136555 | 0.104836617818434 | 379 | 254 |
| C10orf137 | 0.0821287617174794 | 0.110423457640917 | 0.224607747035711 | 379 | 379 |
| C10orf140 | 0.133916305785017 | 0.00904819675489835 | 0.0338441134231512 | 379 | 370 |
| C10orf18 | 0.0369135757557858 | 0.473681991135674 | 0.626009495162031 | 379 | 379 |
| C10orf25 | 0.100041087727037 | 0.051649529579447 | 0.12784116730389 | 379 | 379 |
| C10orf26 | 0.0542498097260204 | 0.292149273438809 | 0.450469070378585 | 379 | 379 |
| C10orf27 | 0.00409946411691088 | 0.936599328086119 | 0.963920454546432 | 379 | 82 |
| C10orf28 | 0.093264003008558 | 0.0697372269327103 | 0.160133148305939 | 379 | 379 |
| C10orf2 | 0.0413126252132637 | 0.422577969558979 | 0.580360760997484 | 379 | 379 |
| C10orf32 | -0.00127983507669929 | 0.980187818699823 | 0.989016542118981 | 379 | 379 |
| C10orf35 | -0.0149978638624915 | 0.771029011224221 | 0.85824752902677 | 379 | 379 |
| C10orf40 | -0.0348027762293738 | 0.499355364543948 | 0.649899453113056 | 379 | 5 |
| C10orf41 | -0.204076555866091 | 0.0000628043712471809 | 0.000708612486004487 | 379 | 379 |
| C10orf46 | 0.21728434475003 | 0.000019794948606756 | 0.000292469628148105 | 379 | 379 |
| C10orf47 | -0.171769443840639 | 0.000785258550412731 | 0.0050783126345674 | 379 | 379 |
| C10orf4 | 0.0541310598980765 | 0.29320846791555 | 0.451620570323965 | 379 | 379 |
| C10orf50 | 0.0843679682384209 | 0.10101166837766 | 0.211160712766711 | 379 | 112 |
| C10orf53 | -0.0326188523186074 | 0.526673441847123 | 0.673435116634498 | 379 | 11 |
| C10orf54 | 0.185245031779535 | 0.000288008466779767 | 0.00229059209898011 | 379 | 379 |
| C10orf55 | 0.0713102548460976 | 0.165917618781502 | 0.30198407795113 | 379 | 377 |
| C10orf57 | -0.0265136991695212 | 0.606866032017726 | 0.736500164209051 | 379 | 379 |
| C10orf58 | -0.162796938623185 | 0.00147229503370097 | 0.00824884598141364 | 379 | 379 |
| C10orf62 | -0.0469111431368856 | 0.362428523706051 | 0.523739725096099 | 379 | 129 |
| C10orf67 | -0.146650510314655 | 0.00422261880256404 | 0.0188275434264088 | 379 | 285 |
| C10orf68 | -0.117225610344493 | 0.0224612798398947 | 0.0679907347098666 | 379 | 351 |
| C10orf71 | 0.0492670485275284 | 0.338799539826734 | 0.49997895770203 | 379 | 37 |
| C10orf72 | 0.0996266828598606 | 0.0526303289608961 | 0.129746880845039 | 379 | 379 |
| C10orf75 | -0.0590941310679624 | 0.25111596056935 | 0.405169441465463 | 379 | 379 |
| C10orf76 | 0.070572171846124 | 0.170354586903769 | 0.307496026028656 | 379 | 379 |
| C10orf78 | 0.134091609598539 | 0.00895739800376196 | 0.0335805043710705 | 379 | 379 |
| C10orf79 | 0.118760029769943 | 0.0207466366716528 | 0.0640056499028369 | 379 | 371 |
| C10orf81 | -0.0446743802725716 | 0.385789917634584 | 0.545375908089158 | 379 | 379 |
| C10orf82 | -0.146993295100831 | 0.00413343427568942 | 0.0185173372838612 | 379 | 319 |
| C10orf84 | -0.0163585406902346 | 0.750912724374539 | 0.843431569642461 | 379 | 379 |
| C10orf88 | 0.0774580707289993 | 0.132265234624558 | 0.256459522016012 | 379 | 379 |
| C10orf90 | -0.0427623519462753 | 0.406468646165709 | 0.565290749498614 | 379 | 169 |
| C10orf91 | 0.161287572638454 | 0.00163152270698395 | 0.00894382975783185 | 379 | 372 |
| C10orf93 | 0.165103712860851 | 0.0012563232665789 | 0.00730509610842419 | 379 | 201 |
| C10orf95 | -0.0574618384933728 | 0.264469352509126 | 0.419377664871317 | 379 | 379 |
| C10orf99 | -0.215063588583218 | 0.0000241582494433434 | 0.00033567608266476 | 379 | 378 |
| C11orf10 | 0.0870531481362013 | 0.090577352720186 | 0.195220887775526 | 379 | 379 |
| C11orf16 | 0.0533326470388913 | 0.30039653999101 | 0.459304641806119 | 379 | 206 |
| C11orf17 | -0.0575482048932026 | 0.263750821216753 | 0.41860652229935 | 379 | 379 |
| C11orf1 | -0.159729430930788 | 0.00181232229757442 | 0.00969636441346615 | 379 | 379 |
| C11orf20 | 0.215086701502524 | 0.0000241084721693005 | 0.000335454586787993 | 379 | 350 |
| C11orf21 | 0.083689866910042 | 0.103792122768574 | 0.214948602257571 | 379 | 361 |
| C11orf24 | 0.2689191621952 | 1.05927450481186E-07 | 5.26188799381533E-06 | 379 | 379 |
| C11orf2 | -0.124799329213306 | 0.0150543188189654 | 0.050083763995806 | 379 | 379 |
| C11orf30 | 0.0914887167711972 | 0.0752488697656858 | 0.16949160396615 | 379 | 379 |
| C11orf31 | -0.0265178830451059 | 0.606809260719174 | 0.73647634336413 | 379 | 379 |
| C11orf34 | 0.0169601346635794 | 0.742071319757337 | 0.836866689122312 | 379 | 97 |
| C11orf35 | -0.0579678662392555 | 0.260278519542901 | 0.414388335718607 | 379 | 379 |
| C11orf36 | -0.00238149948286876 | 0.963143056961558 | 0.979900544371555 | 379 | 38 |
| C11orf41 | 0.316510678484544 | 2.89584067420615E-10 | 5.36623634468781E-08 | 379 | 376 |
| C11orf42 | 0.0246549686637294 | 0.632316934074374 | 0.757054535887126 | 379 | 328 |
| C11orf45 | 0.164573396648068 | 0.00130322174441122 | 0.00750952651792664 | 379 | 379 |
| C11orf46 | -0.156325176751261 | 0.00227277562325066 | 0.0115609530676794 | 379 | 379 |
| C11orf48 | 0.166571624495923 | 0.00113448336750604 | 0.00678772969550689 | 379 | 379 |
| C11orf49 | -0.146871838100735 | 0.00416483790789602 | 0.0186163407464518 | 379 | 379 |
| C11orf51 | 0.0343617656600248 | 0.504810777251232 | 0.654593426939862 | 379 | 379 |
| C11orf52 | -0.122420744812848 | 0.0171079213391006 | 0.0551375338148869 | 379 | 379 |
| C11orf53 | -0.0348529210703602 | 0.49873704084205 | 0.649265185858852 | 379 | 368 |
| C11orf54 | -0.161323799102723 | 0.00162752306733696 | 0.00893178172686335 | 379 | 379 |
| C11orf57 | -0.00165789674076256 | 0.974337106649138 | 0.986476519129857 | 379 | 379 |
| C11orf58 | -0.13516076082203 | 0.00842096948797797 | 0.0320912902186483 | 379 | 379 |
| C11orf59 | 0.100922033268004 | 0.0496150922547237 | 0.124197456031645 | 379 | 379 |
| C11orf61 | 0.0110259049790642 | 0.830586878580189 | 0.898024790255084 | 379 | 379 |
| C11orf63 | -0.0542481315010278 | 0.29216422458486 | 0.450469070378585 | 379 | 379 |
| C11orf65 | 0.0176109989063832 | 0.732543876632776 | 0.830414066686545 | 379 | 372 |
| C11orf66 | -0.0384568418460306 | 0.455378724400232 | 0.609631311012612 | 379 | 379 |
| C11orf67 | -0.200229140491751 | 0.0000867339603373695 | 0.000901814874446441 | 379 | 379 |
| C11orf68 | -0.0184516961774695 | 0.720298320998987 | 0.821698903192802 | 379 | 379 |
| C11orf70 | -0.0146749547153262 | 0.775826082013915 | 0.861797173903188 | 379 | 372 |
| C11orf71 | -0.0361106912321474 | 0.483361284714126 | 0.635321784898101 | 379 | 379 |
| C11orf73 | 0.0857449474883498 | 0.0955476022616058 | 0.203100113383696 | 379 | 379 |
| C11orf74 | -0.0632827863039523 | 0.219016612692092 | 0.366964796050262 | 379 | 379 |
| C11orf75 | 0.137761197773616 | 0.00723384550150414 | 0.0286464327149639 | 379 | 379 |
| C11orf80 | -0.0215676437806839 | 0.675553094716372 | 0.788211531248454 | 379 | 379 |
| C11orf82 | 0.200414626065467 | 0.0000854063768465291 | 0.00088972089040151 | 379 | 379 |
| C11orf83 | 0.130660341529741 | 0.0108900441374473 | 0.038955041522155 | 379 | 379 |
| C11orf84 | -0.030123627764656 | 0.558789795932259 | 0.700315028995944 | 379 | 379 |
| C11orf85 | 0.188767562878643 | 0.000218975388785912 | 0.00184445369959519 | 379 | 91 |
| C11orf86 | 0.127373459765315 | 0.0130786113890095 | 0.0449277038498405 | 379 | 358 |
| C11orf87 | -0.084767167976872 | 0.0994026533719811 | 0.208655072100322 | 379 | 229 |
| C11orf88 | -0.0192290124394264 | 0.709039123856085 | 0.812789948998003 | 379 | 214 |
| C11orf90 | 0.0971018280902112 | 0.0589465607067301 | 0.14117555329062 | 379 | 330 |
| C11orf92 | -0.0145232910418996 | 0.778082121732467 | 0.863577515237131 | 379 | 379 |
| C11orf93 | 0.0365410899966483 | 0.478159297666098 | 0.630124498134986 | 379 | 379 |
| C11orf94 | -0.0354018672957258 | 0.491994712440742 | 0.643190555698229 | 379 | 35 |
| C11orf95 | -0.0589811395605636 | 0.252024936694456 | 0.406115209975968 | 379 | 379 |
| C11orf9 | 0.253050605120272 | 5.98907673543434E-07 | 0.0000198521723357779 | 379 | 379 |
| C12orf10 | 0.14319331304211 | 0.00522419745820118 | 0.0222115612293936 | 379 | 379 |
| C12orf11 | 0.136097343811674 | 0.0079747707989104 | 0.0307753513820154 | 379 | 379 |
| C12orf12 | -0.0176448691257878 | 0.732049186943657 | 0.830315240847568 | 379 | 7 |
| C12orf23 | 0.175173807466231 | 0.000613602952338233 | 0.0041698633548574 | 379 | 379 |
| C12orf24 | 0.0777711410037314 | 0.130704086418191 | 0.254279638990557 | 379 | 379 |
| C12orf26 | -0.00106332297742469 | 0.983538960656361 | 0.990734124766019 | 379 | 379 |
| C12orf27 | -0.141100412070893 | 0.0059298115199453 | 0.0245821247788993 | 379 | 377 |
| C12orf29 | -0.00347480535419452 | 0.946244168230245 | 0.969694641748316 | 379 | 379 |
| C12orf32 | -0.00773746787909064 | 0.880656444769384 | 0.930155107879298 | 379 | 379 |
| C12orf34 | 0.103088785571982 | 0.044894490625709 | 0.11524970814465 | 379 | 379 |
| C12orf35 | 0.186320355407391 | 0.000265034734367665 | 0.00215992959845543 | 379 | 379 |
| C12orf36 | 0.10937060692065 | 0.0332894768702128 | 0.0919821275616748 | 379 | 379 |
| C12orf39 | 0.0132319909713732 | 0.797364266820527 | 0.876347315363842 | 379 | 47 |
| C12orf40 | 0.00813937678104542 | 0.874507260285595 | 0.926861768064078 | 379 | 50 |
| C12orf41 | 0.0671144506099516 | 0.192324485146955 | 0.335292032835157 | 379 | 379 |
| C12orf42 | 0.041618101421547 | 0.419152893512933 | 0.57707009947052 | 379 | 245 |
| C12orf43 | 0.282598430483144 | 2.16909656947187E-08 | 1.62297535016937E-06 | 379 | 379 |
| C12orf44 | 0.206261257143647 | 0.0000521444561331371 | 0.000610703057417509 | 379 | 379 |
| C12orf45 | -0.0620011375238109 | 0.228510302421514 | 0.378332906690693 | 379 | 379 |
| C12orf47 | -0.060077262156639 | 0.24330314554937 | 0.396044230354889 | 379 | 379 |
| C12orf48 | 0.0100447265267506 | 0.845464236340842 | 0.908216755778861 | 379 | 379 |
| C12orf49 | 0.0684341931125336 | 0.183706204432048 | 0.324472351815309 | 379 | 379 |
| C12orf4 | 0.0175283163363867 | 0.733751957098531 | 0.831246320009106 | 379 | 379 |
| C12orf50 | 0.00842288592919144 | 0.870174099079735 | 0.924245341576654 | 379 | 107 |
| C12orf51 | -0.0354473572531949 | 0.491438184387072 | 0.642802052907637 | 379 | 379 |
| C12orf52 | 0.0728688418546358 | 0.156833534961586 | 0.290137649861758 | 379 | 379 |
| C12orf53 | 0.0496298497509219 | 0.335250273681416 | 0.496329607000308 | 379 | 378 |
| C12orf54 | -0.0137149325887948 | 0.790137697198754 | 0.871614422335908 | 379 | 120 |
| C12orf56 | 0.0163704561962521 | 0.75073728560996 | 0.84333006056735 | 379 | 180 |
| C12orf57 | 0.185633565240198 | 0.000279501311643305 | 0.00224572312981744 | 379 | 379 |
| C12orf59 | 0.126895598644927 | 0.0134271154184794 | 0.0457985551331573 | 379 | 355 |
| C12orf5 | -0.0141852520894333 | 0.783117229239909 | 0.866643323177369 | 379 | 379 |
| C12orf60 | 0.100011413941877 | 0.0517192504492606 | 0.12799269498883 | 379 | 378 |
| C12orf61 | 0.0107499394116405 | 0.834765406222187 | 0.900843096355411 | 379 | 379 |
| C12orf62 | 0.077235810385928 | 0.133382256824013 | 0.257994672549656 | 379 | 379 |
| C12orf63 | 0.0664450865839125 | 0.196807125801498 | 0.340900820336517 | 379 | 253 |
| C12orf65 | 0.0893263393424796 | 0.0824353138033658 | 0.181906157981357 | 379 | 379 |
| C12orf66 | 0.0488720890058873 | 0.342690611984259 | 0.504200082731504 | 379 | 379 |
| C12orf68 | 0.107834935421337 | 0.035860076056602 | 0.0972706626306516 | 379 | 379 |
| C12orf69 | 0.0598450041584433 | 0.245133338490858 | 0.398434612312216 | 379 | 357 |
| C12orf70 | 0.26900276241278 | 1.04933333753624E-07 | 5.24085174223389E-06 | 379 | 341 |
| C12orf71 | 0.0640838843912878 | 0.21322774646946 | 0.360149085800246 | 379 | 195 |
| C12orf72 | 0.0918838883947873 | 0.0739923070242192 | 0.167345667124013 | 379 | 379 |
| C12orf73 | -0.0830464385441659 | 0.106486083715061 | 0.21870790013489 | 379 | 379 |
| C12orf74 | -0.107972070594704 | 0.0356238630353861 | 0.0967072776924474 | 379 | 375 |
| C12orf75 | 0.116816909027137 | 0.0229381769669031 | 0.0690898029621381 | 379 | 379 |
| C12orf76 | -0.0186960623765663 | 0.716752097126343 | 0.819046979107827 | 379 | 379 |
| C12orf77 | 0.0309047830515183 | 0.548634301817651 | 0.692710197175266 | 379 | 47 |
| C13orf15 | -0.0152117677907854 | 0.767856082243913 | 0.855965053057419 | 379 | 379 |
| C13orf16 | 0.02941666811648 | 0.568058406112266 | 0.707540431406564 | 379 | 310 |
| C13orf18 | -0.339917767087208 | 1.05244562536146E-11 | 0.0000000035979123896 | 379 | 379 |
| C13orf1 | -0.247244300089133 | 1.09749712847968E-06 | 0.0000320488557636158 | 379 | 379 |
| C13orf23 | -0.275101001955871 | 5.22917016752757E-08 | 3.04058610210371E-06 | 379 | 379 |
| C13orf26 | 0.00828556770915505 | 0.872272397810638 | 0.925482507694206 | 379 | 67 |
| C13orf27 | -0.038135713692622 | 0.45915433498685 | 0.613282246762202 | 379 | 379 |
| C13orf29 | 0.0892838825448848 | 0.082581776696934 | 0.182139206712659 | 379 | 379 |
| C13orf30 | -0.0243513842240428 | 0.6365164179742 | 0.760258038932867 | 379 | 274 |
| C13orf31 | -0.207459536020163 | 0.0000470477316272242 | 0.000566400985248695 | 379 | 379 |
| C13orf33 | 0.0899694248084688 | 0.0802424102496784 | 0.178067271376961 | 379 | 379 |
| C13orf34 | -0.0912785804862291 | 0.0759240935426148 | 0.170508882859097 | 379 | 379 |
| C13orf35 | -0.0347046400997546 | 0.500566629429888 | 0.650534008189825 | 379 | 90 |
| C13orf36 | -0.0405442346933546 | 0.431265216086098 | 0.588231870713019 | 379 | 104 |
| C13orf37 | -0.0631093059203487 | 0.220284880449964 | 0.368622551233175 | 379 | 379 |
| C13orf38 | -0.0465767022628319 | 0.365864283006638 | 0.526861573349962 | 379 | 192 |
| C13orf39 | 0.0220386767873367 | 0.668882669428044 | 0.783240156464848 | 379 | 36 |
| C14orf101 | -0.268900495768974 | 1.06150655513725E-07 | 5.26188799381533E-06 | 379 | 379 |
| C14orf102 | 0.0540089723049747 | 0.294300106661402 | 0.452671050723937 | 379 | 379 |
| C14orf104 | -0.0427511137180862 | 0.406592104002175 | 0.565311565679485 | 379 | 379 |
| C14orf105 | 0.124901802040677 | 0.0149709260838283 | 0.0498896676285961 | 379 | 236 |
| C14orf106 | -0.0304473977535808 | 0.554569569064876 | 0.697273647141303 | 379 | 379 |
| C14orf109 | -0.0175157327881255 | 0.733935873816592 | 0.831246320009106 | 379 | 379 |
| C14orf115 | 0.198686964363563 | 0.0000985484973268081 | 0.000994632628615377 | 379 | 308 |
| C14orf118 | 0.0669633690193005 | 0.193329666985285 | 0.33663046015852 | 379 | 379 |
| C14orf119 | 0.0365411311117955 | 0.47815880219079 | 0.630124498134986 | 379 | 379 |
| C14orf126 | -0.0678280956150256 | 0.187628135947852 | 0.329491690689399 | 379 | 379 |
| C14orf128 | 0.0824527769489853 | 0.109020362926014 | 0.222644531475642 | 379 | 379 |
| C14orf129 | 0.0698264856638519 | 0.17492658830246 | 0.313630924392909 | 379 | 379 |
| C14orf132 | 0.0370770118970886 | 0.471724747862397 | 0.624556510457773 | 379 | 379 |
| C14orf135 | 0.0964770816782412 | 0.0606026474574644 | 0.144097528934717 | 379 | 379 |
| C14orf138 | 0.116745414318146 | 0.0230224953703915 | 0.0692701120188351 | 379 | 379 |
| C14orf139 | 0.146453345687584 | 0.00427470041460362 | 0.0190127321266847 | 379 | 379 |
| C14orf142 | 0.0370536189027 | 0.472004620053977 | 0.624801896417 | 379 | 379 |
| C14orf143 | 0.0195628652020538 | 0.704222562937698 | 0.809254296947767 | 379 | 379 |
| C14orf145 | 0.130770944492255 | 0.0108224043639194 | 0.0387830532672681 | 379 | 379 |
| C14orf147 | -0.0194819069569072 | 0.705389487824013 | 0.810165537031928 | 379 | 379 |
| C14orf148 | -0.0727506535966089 | 0.157508942939707 | 0.291088388536538 | 379 | 372 |
| C14orf149 | 0.0750435603187749 | 0.144792798169457 | 0.273684614118588 | 379 | 379 |
| C14orf153 | 0.00686795934544913 | 0.893983948248585 | 0.938858625743769 | 379 | 379 |
| C14orf156 | 0.075798535237453 | 0.14078196187135 | 0.268251464538259 | 379 | 379 |
| C14orf159 | -0.01659357588452 | 0.747454547380381 | 0.84083366916932 | 379 | 379 |
| C14orf162 | 0.120356150697866 | 0.0190847857420547 | 0.0601515071838278 | 379 | 95 |
| C14orf165 | -0.0449964226739661 | 0.382371077610193 | 0.542321439689192 | 379 | 12 |
| C14orf166B | -0.195036540489647 | 0.000132818662481134 | 0.00124764848091864 | 379 | 175 |
| C14orf166 | 0.00770383218552217 | 0.881171399498029 | 0.930443418321809 | 379 | 379 |
| C14orf167 | -0.016821155235667 | 0.744110888222735 | 0.838308562027295 | 379 | 379 |
| C14orf169 | 0.0142633475397816 | 0.781953181106605 | 0.865935083774463 | 379 | 379 |
| C14orf174 | 0.0464463547198071 | 0.36720882679512 | 0.528069090346217 | 379 | 379 |
| C14orf176 | 0.0453055537606683 | 0.379106768804236 | 0.53919582611365 | 379 | 379 |
| C14orf178 | -0.0435700627804735 | 0.397653995617203 | 0.55710332968967 | 379 | 188 |
| C14orf179 | 0.00260687035612331 | 0.959657970064257 | 0.978157519685092 | 379 | 379 |
| C14orf180 | 0.0610280387417674 | 0.235910823900075 | 0.387125698608847 | 379 | 130 |
| C14orf181 | 0.00429259479509251 | 0.933619165004753 | 0.961866744648248 | 379 | 379 |
| C14orf182 | 0.0599146878659895 | 0.244583223544695 | 0.397703473547991 | 379 | 378 |
| C14orf183 | 0.136050244890619 | 0.0079966954100645 | 0.0308419522642986 | 379 | 71 |
| C14orf184 | -0.0847110369331474 | 0.0996276568362948 | 0.208936669345385 | 379 | 332 |
| C14orf19 | -0.0547318033440774 | 0.287876419687019 | 0.44618257246574 | 379 | 376 |
| C14orf1 | 0.0468767072813214 | 0.362781355832588 | 0.524089458690002 | 379 | 379 |
| C14orf21 | 0.035740240815683 | 0.487863097677682 | 0.639580584694347 | 379 | 379 |
| C14orf23 | -0.0292566623939691 | 0.570166286051643 | 0.709125106738723 | 379 | 48 |
| C14orf28 | -0.05414740525446 | 0.293062522619138 | 0.451467927782788 | 379 | 379 |
| C14orf2 | 0.0284872538780655 | 0.580353824121358 | 0.716784329430565 | 379 | 379 |
| C14orf33 | -0.0451452328945619 | 0.380797560183675 | 0.540592398032642 | 379 | 379 |
| C14orf34 | 0.29084059723209 | 7.99666896126653E-09 | 7.34064593351819E-07 | 379 | 349 |
| C14orf37 | 0.0658718795063473 | 0.20070598783008 | 0.345691307044374 | 379 | 378 |
| C14orf39 | 0.00363639574939849 | 0.943748374791473 | 0.968112306656595 | 379 | 43 |
| C14orf43 | 0.112220336873613 | 0.0289340029071922 | 0.0824879093664711 | 379 | 379 |
| C14orf45 | -0.0307221962859163 | 0.550999892086178 | 0.694752011492899 | 379 | 379 |
| C14orf48 | -0.01687042795392 | 0.743387589583826 | 0.837779306938053 | 379 | 20 |
| C14orf49 | 0.147778954917461 | 0.00393541315159189 | 0.0178479807799094 | 379 | 379 |
| C14orf4 | 0.0122142109916463 | 0.812650035071379 | 0.886462281751406 | 379 | 379 |
| C14orf50 | -0.070380005252102 | 0.171524191446035 | 0.309125730104171 | 379 | 379 |
| C14orf53 | -0.0589413371222332 | 0.252345676577473 | 0.406326951045812 | 379 | 247 |
| C14orf64 | 0.0688248686935564 | 0.181210481359859 | 0.32121404776406 | 379 | 336 |
| C14orf68 | -0.0107359094739799 | 0.834977965489855 | 0.900954674560995 | 379 | 351 |
| C14orf70 | 0.0079274146893779 | 0.877749352306044 | 0.92861029546069 | 379 | 61 |
| C14orf72 | 0.151514998231936 | 0.0031061918414838 | 0.0147945164143504 | 379 | 378 |
| C14orf73 | 0.0390859836813942 | 0.448032396554483 | 0.603094796937019 | 379 | 379 |
| C14orf79 | -0.0265691180287105 | 0.606114241444858 | 0.736149633223278 | 379 | 379 |
| C14orf80 | 0.250483795939978 | 7.84231326088772E-07 | 0.0000242735053990205 | 379 | 379 |
| C14orf86 | -0.0502268066166837 | 0.32946239040797 | 0.490139576606335 | 379 | 8 |
| C14orf93 | -0.236935752871254 | 3.10139767878693E-06 | 0.0000705212307052607 | 379 | 379 |
| C15orf17 | -0.00448271378325851 | 0.930686379343695 | 0.96033560015232 | 379 | 379 |
| C15orf21 | 0.113371837470564 | 0.0273180030873384 | 0.0791996377013423 | 379 | 379 |
| C15orf23 | 0.155916124589844 | 0.00233475835858429 | 0.0118126023817324 | 379 | 379 |
| C15orf24 | 0.038895990168648 | 0.450243805652857 | 0.60541395486809 | 379 | 379 |
| C15orf26 | 0.0856921022939685 | 0.0957528589556688 | 0.203401843038138 | 379 | 298 |
| C15orf27 | 0.0198314010220087 | 0.700356887714695 | 0.806286731092549 | 379 | 379 |
| C15orf28 | -0.0763183598029177 | 0.138070214265923 | 0.264456743476112 | 379 | 356 |
| C15orf29 | 0.00116084241098418 | 0.982029530085298 | 0.989716454332179 | 379 | 379 |
| C15orf2 | 0.028064156017456 | 0.585991913562718 | 0.721679979013762 | 379 | 82 |
| C15orf32 | -0.0327348909406255 | 0.525203062448868 | 0.672152628586988 | 379 | 12 |
| C15orf33 | -0.0557865879650034 | 0.278672906830137 | 0.435493927034747 | 379 | 378 |
| C15orf34 | 0.00807078321420505 | 0.875556214622925 | 0.927378665787573 | 379 | 378 |
| C15orf37 | 0.0379938085030264 | 0.460828310265628 | 0.614561725581576 | 379 | 379 |
| C15orf38 | 0.00346861860534917 | 0.946339734308054 | 0.969694641748316 | 379 | 379 |
| C15orf39 | 0.102668198696699 | 0.0457800804209407 | 0.117089848316585 | 379 | 379 |
| C15orf40 | 0.0358222305628606 | 0.486864798703932 | 0.638660560629604 | 379 | 379 |
| C15orf41 | 0.110345727508489 | 0.0317400917779412 | 0.088689760396423 | 379 | 379 |
| C15orf42 | 0.136650876707107 | 0.0077210894678783 | 0.0300616070390515 | 379 | 379 |
| C15orf43 | 0.0516338446721931 | 0.316076967832132 | 0.47597224314682 | 379 | 7 |
| C15orf44 | -0.000196708829052268 | 0.996954591566218 | 0.997810087404723 | 379 | 379 |
| C15orf48 | 0.0655207376517636 | 0.203121960518376 | 0.348615341283957 | 379 | 379 |
| C15orf50 | -0.0116128048822969 | 0.821716331347677 | 0.892854176313355 | 379 | 119 |
| C15orf51 | 0.0372572239184472 | 0.469571761078124 | 0.622370914348732 | 379 | 326 |
| C15orf52 | -0.0595432784518807 | 0.247525273553843 | 0.401138628853747 | 379 | 379 |
| C15orf53 | 0.0538994466450847 | 0.295281730173615 | 0.453758517079938 | 379 | 244 |
| C15orf54 | 0.156294039745189 | 0.00227744040903603 | 0.0115817102924766 | 379 | 282 |
| C15orf55 | 0.180689160817942 | 0.000407522445860466 | 0.0029971643384723 | 379 | 203 |
| C15orf56 | -0.0877032744717968 | 0.0881855541557182 | 0.191348562902121 | 379 | 377 |
| C15orf57 | -0.0670619515498754 | 0.192673339163523 | 0.335734859735858 | 379 | 379 |
| C15orf58 | 0.0726270674598955 | 0.158217543529203 | 0.291989710824371 | 379 | 379 |
| C15orf59 | 0.0656290585276401 | 0.202374437550449 | 0.347719267569351 | 379 | 376 |
| C15orf5 | -0.0118139188120134 | 0.81868180151739 | 0.89078007177077 | 379 | 216 |
| C15orf60 | 0.0193273837575409 | 0.707618686450166 | 0.811706398660854 | 379 | 54 |
| C15orf61 | 0.0505552493698988 | 0.326305588847294 | 0.486794614074498 | 379 | 379 |
| C15orf62 | -0.0825709069489458 | 0.108512327788387 | 0.221835491843297 | 379 | 379 |
| C15orf63 | 0.0543543715243667 | 0.291218751925619 | 0.449675680490707 | 379 | 379 |
| C16orf11 | -0.0119420804661381 | 0.816749427468337 | 0.889466010207183 | 379 | 106 |
| C16orf13 | -0.0313659285232594 | 0.542682037793267 | 0.687383380948313 | 379 | 379 |
| C16orf3 | -0.166266718502681 | 0.0011588580377 | 0.00689610959529281 | 379 | 350 |
| C16orf42 | 0.0719538318715081 | 0.162119927929499 | 0.297447388820775 | 379 | 379 |
| C16orf45 | 0.0693898023650871 | 0.177646019741153 | 0.316816448950133 | 379 | 379 |
| C16orf46 | 0.152188290724196 | 0.00297485981100812 | 0.0143273063717923 | 379 | 379 |
| C16orf48 | -0.0709865327795027 | 0.167852888221233 | 0.304249663374222 | 379 | 379 |
| C16orf52 | -0.119061756641888 | 0.0204231811518669 | 0.0632044382517899 | 379 | 379 |
| C16orf53 | 0.0906591460670063 | 0.0779431928978838 | 0.173900937186817 | 379 | 379 |
| C16orf54 | 0.171519148556009 | 0.000799486808710972 | 0.00515110011317888 | 379 | 379 |
| C16orf55 | -0.0723315033346085 | 0.159921955093828 | 0.294464333314698 | 379 | 379 |
| C16orf57 | 0.0438130323030967 | 0.395025062410307 | 0.554674381238692 | 379 | 379 |
| C16orf58 | -0.0335078092026346 | 0.515462725195947 | 0.663933669948372 | 379 | 379 |
| C16orf59 | 0.111265135784911 | 0.0303360614351432 | 0.0856483876634667 | 379 | 379 |
| C16orf5 | -0.0436889157050998 | 0.396366695452855 | 0.556029407216012 | 379 | 379 |
| C16orf61 | 0.0563881880598497 | 0.273513918579448 | 0.429767333195442 | 379 | 379 |
| C16orf62 | -0.0561721085479269 | 0.275359360968408 | 0.431671036551643 | 379 | 379 |
| C16orf63 | -0.149374373198688 | 0.00355947479476517 | 0.016493869182193 | 379 | 379 |
| C16orf68 | -0.0954341240033311 | 0.0634527982732822 | 0.149051307210359 | 379 | 379 |
| C16orf70 | -0.0120059149079591 | 0.815787368358993 | 0.888662341491161 | 379 | 379 |
| C16orf71 | 0.0871352921387942 | 0.0902723083035214 | 0.194641075363443 | 379 | 378 |
| C16orf72 | -0.0783879141944553 | 0.127670195397269 | 0.249769605295755 | 379 | 379 |
| C16orf73 | -0.0464117670140558 | 0.367566115557991 | 0.52827822732394 | 379 | 148 |
| C16orf74 | 0.204556071967941 | 0.0000603020525232545 | 0.000687166147948902 | 379 | 378 |
| C16orf75 | -0.0503927575233258 | 0.32786491135521 | 0.488312586370548 | 379 | 379 |
| C16orf78 | -0.00255688402261938 | 0.960430884085536 | 0.978643484744746 | 379 | 9 |
| C16orf79 | -0.0326900252631035 | 0.525771328137661 | 0.672710453269249 | 379 | 379 |
| C16orf7 | -0.0267517288758346 | 0.603639956294936 | 0.734878925119175 | 379 | 379 |
| C16orf80 | -0.0369198124114647 | 0.473607222014154 | 0.626009495162031 | 379 | 379 |
| C16orf81 | 0.0673811443275532 | 0.190559457265023 | 0.333310949069413 | 379 | 137 |
| C16orf82 | 0.0254423285331987 | 0.621480544507979 | 0.748282501609437 | 379 | 19 |
| C16orf86 | -0.0362757406056178 | 0.481362816787458 | 0.633299842828062 | 379 | 379 |
| C16orf87 | -0.0656661146159678 | 0.202119172217141 | 0.347461327095671 | 379 | 379 |
| C16orf88 | -0.147088650773311 | 0.00410892947293003 | 0.018447157125308 | 379 | 379 |
| C16orf89 | -0.0134336538344583 | 0.794344511506194 | 0.874091957053378 | 379 | 364 |
| C16orf90 | -0.0116338793953079 | 0.821398220738568 | 0.892843300641695 | 379 | 32 |
| C16orf91 | -0.0474536784034308 | 0.35689795241677 | 0.51797486462595 | 379 | 379 |
| C16orf92 | 0.0883266518820187 | 0.085940055436891 | 0.187915683635055 | 379 | 53 |
| C16orf93 | 0.079597131970666 | 0.121880722796933 | 0.241254963723428 | 379 | 379 |
| C17orf100 | -0.0427872522833741 | 0.406195182292335 | 0.56503704745983 | 379 | 379 |
| C17orf101 | 0.182027863104679 | 0.000368320878481239 | 0.00276841030270129 | 379 | 379 |
| C17orf102 | 0.0619975878748895 | 0.22853699519433 | 0.378344455265357 | 379 | 38 |
| C17orf103 | -0.102080565983092 | 0.0470419122559029 | 0.119460429842475 | 379 | 379 |
| C17orf104 | 0.0233344213740821 | 0.650668403007776 | 0.770240866879987 | 379 | 379 |
| C17orf105 | 0.0315264942970499 | 0.540617074462385 | 0.68564381172062 | 379 | 11 |
| C17orf106 | 0.00224633050259414 | 0.96523362189757 | 0.981258735677474 | 379 | 379 |
| C17orf107 | 0.08834004425987 | 0.0858923237801656 | 0.187873468936914 | 379 | 379 |
| C17orf108 | -0.0970405019066452 | 0.0591074507885978 | 0.141458362611505 | 379 | 379 |
| C17orf28 | 0.0303704072350338 | 0.555571704676971 | 0.698031211514076 | 379 | 379 |
| C17orf37 | -0.0135170218749464 | 0.793097028992505 | 0.873446339194812 | 379 | 379 |
| C17orf39 | -0.00835579960029528 | 0.871199102685076 | 0.924763445328017 | 379 | 379 |
| C17orf42 | 0.0225218368040371 | 0.662067453147683 | 0.777832165729233 | 379 | 379 |
| C17orf44 | -0.0956764632086493 | 0.0627808931611464 | 0.147858361990641 | 379 | 379 |
| C17orf46 | -0.00800387447223042 | 0.876579613946533 | 0.928015862350425 | 379 | 368 |
| C17orf47 | -0.0711906889285473 | 0.166630449320421 | 0.302805163916068 | 379 | 345 |
| C17orf48 | 0.0160932992141061 | 0.754821330785543 | 0.846574882448992 | 379 | 379 |
| C17orf49 | 0.0378002113954953 | 0.463117536470245 | 0.616495099908159 | 379 | 379 |
| C17orf50 | 0.00771474058756574 | 0.881004388879355 | 0.930316611956108 | 379 | 272 |
| C17orf51 | 0.0371923527636136 | 0.470346150641585 | 0.623230651892632 | 379 | 379 |
| C17orf53 | 0.198897405851768 | 0.000096851578512243 | 0.000983440404964195 | 379 | 379 |
| C17orf54 | 0.0230608054600953 | 0.654497813415519 | 0.772463252643031 | 379 | 82 |
| C17orf55 | 0.140085477090456 | 0.00630186608647178 | 0.0257689009615514 | 379 | 378 |
| C17orf56 | -0.04906442941677 | 0.340792166885235 | 0.502134731738162 | 379 | 379 |
| C17orf57 | -0.0287337506118953 | 0.577080788403589 | 0.714254548843094 | 379 | 378 |
| C17orf58 | -0.0190686761104076 | 0.711356467095188 | 0.81487053099293 | 379 | 379 |
| C17orf59 | 0.100358750868996 | 0.0509080514490216 | 0.126625069669315 | 379 | 379 |
| C17orf60 | 0.219613294026182 | 0.000016027474111333 | 0.000248469708115333 | 379 | 369 |
| C17orf61 | 0.0758858610287179 | 0.140323584393304 | 0.267635247340365 | 379 | 379 |
| C17orf62 | 0.0506330610862811 | 0.325560585893813 | 0.485904892571316 | 379 | 379 |
| C17orf63 | -0.00323712882699523 | 0.949916074108949 | 0.972075553129245 | 379 | 379 |
| C17orf64 | 0.152259287983662 | 0.00296130943082786 | 0.0142793879850328 | 379 | 121 |
| C17orf65 | -0.0567511174741043 | 0.270433283027598 | 0.426207815504046 | 379 | 379 |
| C17orf66 | 0.147079574832243 | 0.00411125616989793 | 0.018447157125308 | 379 | 134 |
| C17orf67 | 0.0537915312991763 | 0.296251055201227 | 0.454649065211295 | 379 | 379 |
| C17orf68 | 0.0627276486170455 | 0.223093522634497 | 0.371847542602287 | 379 | 379 |
| C17orf69 | -0.0323966929607455 | 0.529494355817207 | 0.676077924344361 | 379 | 379 |
| C17orf70 | 0.0823323985591459 | 0.109539993160845 | 0.223452570410826 | 379 | 379 |
| C17orf71 | -0.0557520301986766 | 0.278971244565784 | 0.435752468666328 | 379 | 379 |
| C17orf72 | 0.172552931629239 | 0.000742219788060089 | 0.00487147764238843 | 379 | 379 |
| C17orf73 | -0.0748449158869863 | 0.145862458952939 | 0.275391433643009 | 379 | 379 |
| C17orf74 | 0.0273569775503054 | 0.595471667428337 | 0.728427718105418 | 379 | 25 |
| C17orf75 | -0.00119582952379609 | 0.981488006001403 | 0.989628904408961 | 379 | 379 |
| C17orf76 | -0.165563516743861 | 0.00121692211670607 | 0.00715573301602843 | 379 | 379 |
| C17orf77 | -0.135939402326101 | 0.00804850503859905 | 0.0309935439707403 | 379 | 310 |
| C17orf78 | 0.113316068139353 | 0.0273944427160156 | 0.0793654310597832 | 379 | 246 |
| C17orf79 | 0.100665378142682 | 0.0502007760158225 | 0.125315496266112 | 379 | 379 |
| C17orf80 | -0.103828260400416 | 0.0433723405422354 | 0.112299134012985 | 379 | 379 |
| C17orf81 | 0.215341391464568 | 0.0000235663553803909 | 0.000329995546950841 | 379 | 379 |
| C17orf82 | 0.12058605639009 | 0.0188552268629463 | 0.0595605286344589 | 379 | 376 |
| C17orf85 | 0.216835495917754 | 0.0000206116044293361 | 0.000300284270848549 | 379 | 379 |
| C17orf86 | -0.0886813785901846 | 0.0846829624045153 | 0.185843251638819 | 379 | 379 |
| C17orf87 | 0.212219514217279 | 0.00003108521407053 | 0.000410623092320156 | 379 | 377 |
| C17orf88 | 0.107104425887719 | 0.0371409025992417 | 0.0998684318874104 | 379 | 95 |
| C17orf89 | 0.0348796889396085 | 0.498407138854791 | 0.649115076356199 | 379 | 379 |
| C17orf90 | -0.0489751110442667 | 0.341672924323152 | 0.503133363793498 | 379 | 379 |
| C17orf91 | 0.0816289977500442 | 0.112615412625954 | 0.227920628921856 | 379 | 379 |
| C17orf93 | 0.0331792309858235 | 0.51959203428492 | 0.667344918758997 | 379 | 362 |
| C17orf95 | -0.0469863155443149 | 0.361659046106604 | 0.522856402931224 | 379 | 379 |
| C17orf96 | 0.146491955128388 | 0.00426445616366131 | 0.0189714240101137 | 379 | 379 |
| C17orf97 | 0.0217106110007925 | 0.673525783964044 | 0.786864791682716 | 379 | 379 |
| C17orf98 | 0.0812686814847408 | 0.114216839611334 | 0.230128187766846 | 379 | 81 |
| C17orf99 | -0.0132942358764562 | 0.796431869828079 | 0.87565917705197 | 379 | 313 |
| C18orf10 | 0.204386108865079 | 0.0000611780353803966 | 0.000693164620298574 | 379 | 379 |
| C18orf16 | -0.0278512335200508 | 0.588838803451518 | 0.723720621452547 | 379 | 31 |
| C18orf18 | -0.00648795194420555 | 0.899818162394851 | 0.942205034007452 | 379 | 379 |
| C18orf19 | 0.292583160784381 | 6.44893233536565E-09 | 6.2989867165335E-07 | 379 | 379 |
| C18orf1 | -0.252047290146702 | 6.65697858630512E-07 | 0.0000216739854530801 | 379 | 379 |
| C18orf20 | 0.0777135722223734 | 0.13099008413606 | 0.254659416437865 | 379 | 27 |
| C18orf21 | 0.195792738164129 | 0.000124911523690096 | 0.00118209456176522 | 379 | 379 |
| C18orf22 | 0.18521275459745 | 0.00028872595584458 | 0.00229177672237243 | 379 | 379 |
| C18orf25 | 0.334130229182289 | 2.4526117428076E-11 | 0.0000000069553469566 | 379 | 379 |
| C18orf26 | 0.0456966993882776 | 0.375000976508433 | 0.535777443594842 | 379 | 26 |
| C18orf2 | 0.0895894072147315 | 0.0815324806680202 | 0.180366621297055 | 379 | 132 |
| C18orf32 | 0.194779566156782 | 0.000135610970791258 | 0.00126417222794973 | 379 | 379 |
| C18orf34 | 0.0297619690009745 | 0.563522184687018 | 0.704016420120338 | 379 | 293 |
| C18orf45 | 0.197507738255596 | 0.000108586305278051 | 0.00106819677220142 | 379 | 379 |
| C18orf54 | 0.259688339726346 | 2.94211730632552E-07 | 0.000011232735952679 | 379 | 379 |
| C18orf55 | 0.249529790875133 | 8.66238138668527E-07 | 0.0000262626449748005 | 379 | 379 |
| C18orf56 | 0.228202307227731 | 7.21326325196946E-06 | 0.000135439946742472 | 379 | 378 |
| C18orf62 | -0.0646949735597786 | 0.208886470689472 | 0.355345004396129 | 379 | 10 |
| C18orf8 | 0.346148747697129 | 4.1495077805301E-12 | 2.05691100680877E-09 | 379 | 379 |
| C19orf10 | 0.148769866115711 | 0.00369791133482352 | 0.0170239577308755 | 379 | 379 |
| C19orf12 | -0.096192601418419 | 0.0613693778560103 | 0.145328081228828 | 379 | 379 |
| C19orf18 | -0.254768401727366 | 4.99219824298554E-07 | 0.0000173946683534745 | 379 | 322 |
| C19orf20 | 0.0439874556430536 | 0.39314426943264 | 0.55293407393321 | 379 | 379 |
| C19orf21 | 0.00160421546621853 | 0.975167777382964 | 0.987115922500991 | 379 | 379 |
| C19orf22 | 0.0482530892168409 | 0.348845883524982 | 0.510473518710948 | 379 | 379 |
| C19orf23 | 0.203571573588112 | 0.0000655452442392936 | 0.000729152341965951 | 379 | 379 |
| C19orf24 | 0.0303427812428638 | 0.555931508875126 | 0.698277585073863 | 379 | 379 |
| C19orf25 | 0.0479992888141843 | 0.351389743888915 | 0.512703901131713 | 379 | 379 |
| C19orf26 | 0.106560851594627 | 0.0381189585051712 | 0.101917841051852 | 379 | 377 |
| C19orf28 | 0.200642598385035 | 0.0000838008952621347 | 0.000878690719861241 | 379 | 379 |
| C19orf29 | 0.0380869623525442 | 0.459729044038321 | 0.613756227120376 | 379 | 379 |
| C19orf2 | -0.11120957785648 | 0.0304193692975326 | 0.0858217493499541 | 379 | 379 |
| C19orf30 | 0.159421889474304 | 0.00185010589109424 | 0.00985596443004206 | 379 | 90 |
| C19orf33 | 0.118542695635378 | 0.0209823692461787 | 0.0645680265491263 | 379 | 379 |
| C19orf34 | -0.0334276679784586 | 0.516468309391885 | 0.664933271227964 | 379 | 236 |
| C19orf35 | 0.151874579074593 | 0.0030354112142194 | 0.0145511544466181 | 379 | 377 |
| C19orf36 | -0.0729152299707509 | 0.156569040251174 | 0.289810579733035 | 379 | 378 |
| C19orf38 | 0.256068056922242 | 4.34600355733038E-07 | 0.0000156320736722554 | 379 | 379 |
| C19orf39 | -0.143009486691665 | 0.0052829995417463 | 0.0224019065256086 | 379 | 379 |
| C19orf40 | 0.0410670390545758 | 0.425343347291709 | 0.582921474294996 | 379 | 379 |
| C19orf41 | 0.0125487906183603 | 0.807616916252929 | 0.883611648295603 | 379 | 225 |
| C19orf42 | -0.0012101906044159 | 0.981265730287655 | 0.98960057474918 | 379 | 379 |
| C19orf43 | 0.0113658577981251 | 0.825446052545219 | 0.895004338047061 | 379 | 379 |
| C19orf44 | 0.00337135303220732 | 0.947842285894979 | 0.970604598705037 | 379 | 379 |
| C19orf45 | -0.240081770840926 | 2.26989743405456E-06 | 0.0000550887715084868 | 379 | 379 |
| C19orf46 | -0.275001473793527 | 5.28967972437369E-08 | 3.06382265473884E-06 | 379 | 375 |
| C19orf47 | 0.102809169897925 | 0.0454816315513572 | 0.116437674680479 | 379 | 379 |
| C19orf48 | 0.102512407597856 | 0.0461118141450956 | 0.117580382056193 | 379 | 379 |
| C19orf50 | 0.0273106881222456 | 0.596094601615735 | 0.728915433909145 | 379 | 379 |
| C19orf51 | 0.287379914761071 | 0.0000000122060342433 | 1.01642297782152E-06 | 379 | 378 |
| C19orf52 | 0.0522908400498323 | 0.30995037224107 | 0.469567235696511 | 379 | 379 |
| C19orf53 | -0.0168474028680172 | 0.743725558194972 | 0.838112552878085 | 379 | 379 |
| C19orf54 | 0.150824228448339 | 0.00324638467566147 | 0.0153078038879942 | 379 | 379 |
| C19orf55 | 0.122147216988328 | 0.0173590952542738 | 0.0557041820200259 | 379 | 379 |
| C19orf56 | -0.0507922337978898 | 0.324040032758537 | 0.48461802455395 | 379 | 379 |
| C19orf57 | -0.0390125598071946 | 0.448886276376607 | 0.604071984552906 | 379 | 379 |
| C19orf59 | 0.171805143999887 | 0.000783248327287076 | 0.00506861874459796 | 379 | 363 |
| C19orf60 | -0.0400775623683479 | 0.436591263697535 | 0.593048679632439 | 379 | 379 |
| C19orf61 | 0.232685780333161 | 4.69593286525837E-06 | 0.0000968896533323028 | 379 | 379 |
| C19orf62 | -0.014501396439195 | 0.778407964539475 | 0.863842453735306 | 379 | 379 |
| C19orf63 | -0.00576384547283797 | 0.910949972889398 | 0.948601232206869 | 379 | 379 |
| C19orf66 | 0.0761562784341357 | 0.138911400981224 | 0.265580489697783 | 379 | 379 |
| C19orf69 | 0.14793918955808 | 0.00389609621569995 | 0.0177101778461482 | 379 | 316 |
| C19orf6 | 0.00646116891925748 | 0.900229566616191 | 0.942486501233742 | 379 | 379 |
| C19orf70 | -0.0750905535326648 | 0.144540626804782 | 0.273390398577241 | 379 | 379 |
| C19orf71 | 0.000335908336363448 | 0.994799555539684 | 0.996898367513637 | 379 | 378 |
| C19orf73 | -0.171916663347484 | 0.000776999440905071 | 0.00504629705675262 | 379 | 379 |
| C19orf75 | -0.0868040213877541 | 0.0915075463315879 | 0.196854901666781 | 379 | 15 |
| C19orf76 | 0.169130831249191 | 0.000947750739191512 | 0.0058946052875437 | 379 | 379 |
| C19orf77 | -0.0527941981031062 | 0.305309675253116 | 0.464559914121616 | 379 | 379 |
| C1D | -0.00900128294985252 | 0.861346029190389 | 0.918446548902297 | 379 | 379 |
| C1GALT1C1 | -0.0726778344727947 | 0.157926172425141 | 0.291641999333678 | 379 | 379 |
| C1GALT1 | 0.116957063318442 | 0.0227736588116416 | 0.0687090850452267 | 379 | 379 |
| C1QA | 0.265633701598317 | 1.53053476976944E-07 | 6.97642377356056E-06 | 379 | 379 |
| C1QBP | 0.145846778844248 | 0.00443858510840092 | 0.0195270169801139 | 379 | 379 |
| C1QB | 0.265474580529755 | 0.0000001557865558841 | 7.03630029628666E-06 | 379 | 379 |
| C1QC | 0.257255199316421 | 3.82660049145584E-07 | 0.0000140507101008493 | 379 | 379 |
| C1QL1 | 0.140448401352626 | 0.00616648757852937 | 0.0253145167095404 | 379 | 376 |
| C1QL2 | -0.0569041386917658 | 0.269141528931838 | 0.424883617488892 | 379 | 29 |
| C1QL3 | -0.011638506027789 | 0.821328387647162 | 0.892834389817321 | 379 | 367 |
| C1QL4 | 0.174751100229333 | 0.000632842507132027 | 0.00427967299843582 | 379 | 273 |
| C1QTNF1 | 0.100319855022012 | 0.0509983609850845 | 0.126719057620699 | 379 | 379 |
| C1QTNF2 | -0.000200316238804438 | 0.996898742616638 | 0.997804556718965 | 379 | 379 |
| C1QTNF3 | -0.0256430078247398 | 0.618731508598401 | 0.745878426099775 | 379 | 379 |
| C1QTNF4 | -0.0191882669521229 | 0.709627765860169 | 0.813229646368942 | 379 | 342 |
| C1QTNF6 | 0.116288142313012 | 0.02356815370686 | 0.0704734356355935 | 379 | 379 |
| C1QTNF7 | -0.0288962771018484 | 0.574927477329694 | 0.712597780073995 | 379 | 378 |
| C1QTNF8 | 0.113019551251141 | 0.0278039540832911 | 0.0802820447886262 | 379 | 220 |
| C1QTNF9B | -0.0910374144493307 | 0.0767050833554321 | 0.171716740103521 | 379 | 326 |
| C1QTNF9 | -0.044984166963394 | 0.382500846064926 | 0.542430396903874 | 379 | 355 |
| C1RL | 0.0327339659090887 | 0.525214775675291 | 0.672152628586988 | 379 | 379 |
| C1R | 0.086970459694365 | 0.0908852525199564 | 0.195728552945117 | 379 | 379 |
| C1S | 0.0733671953683297 | 0.154009642068055 | 0.286678856827394 | 379 | 379 |
| C1orf100 | 0.042111987768961 | 0.41364985307862 | 0.572035799054462 | 379 | 10 |
| C1orf101 | -0.165367109246779 | 0.00123361131809231 | 0.00721961192890623 | 379 | 337 |
| C1orf103 | 0.00514628350852967 | 0.920457717542897 | 0.954741348788479 | 379 | 379 |
| C1orf104 | -0.0579297182761157 | 0.260592849800038 | 0.414674581677395 | 379 | 379 |
| C1orf105 | -0.191874645641081 | 0.000171251188802821 | 0.00152960470431685 | 379 | 320 |
| C1orf106 | -0.0182750209800868 | 0.722865961338487 | 0.823308994279943 | 379 | 379 |
| C1orf107 | -0.225073400716059 | 9.68312332322986E-06 | 0.000170361108476488 | 379 | 379 |
| C1orf109 | 0.0113174904871237 | 0.826177022183141 | 0.895264675809092 | 379 | 379 |
| C1orf110 | 0.12549717232817 | 0.0144943843476601 | 0.0486648382526562 | 379 | 142 |
| C1orf111 | -0.00451736092971405 | 0.930152010410975 | 0.96033560015232 | 379 | 263 |
| C1orf112 | -0.0583159896213016 | 0.257422137438088 | 0.411792347004631 | 379 | 379 |
| C1orf113 | 0.0176221973303932 | 0.732380306387197 | 0.830414066686545 | 379 | 379 |
| C1orf114 | 0.0236281837739407 | 0.646567232768132 | 0.767775759275987 | 379 | 335 |
| C1orf115 | -0.0729082476907119 | 0.156608830103287 | 0.289857171967514 | 379 | 379 |
| C1orf116 | -0.077772255001134 | 0.130698556918287 | 0.254279638990557 | 379 | 379 |
| C1orf122 | 0.142539025301626 | 0.00543621130397284 | 0.0228997658243411 | 379 | 379 |
| C1orf123 | 0.109521717083513 | 0.033045235529793 | 0.0914857484061345 | 379 | 379 |
| C1orf124 | -0.0853915407293548 | 0.0969269689544073 | 0.205240061985048 | 379 | 379 |
| C1orf125 | -0.0401669498588628 | 0.435568187799886 | 0.592267592079011 | 379 | 360 |
| C1orf126 | -0.0767520339416592 | 0.135838715130824 | 0.261242487256449 | 379 | 379 |
| C1orf127 | 0.00839829224789194 | 0.870549837724915 | 0.924396839415713 | 379 | 373 |
| C1orf128 | 0.211956252742495 | 0.0000318137439975835 | 0.00041775027548615 | 379 | 379 |
| C1orf129 | 0.0521470029729573 | 0.311284953770872 | 0.470845456314243 | 379 | 44 |
| C1orf130 | -0.104665519971728 | 0.0417015643215794 | 0.109098643273292 | 379 | 379 |
| C1orf131 | -0.0413727672189505 | 0.421902354180135 | 0.579808696886867 | 379 | 379 |
| C1orf133 | -0.0356097530106691 | 0.489454170024264 | 0.640968052522364 | 379 | 379 |
| C1orf135 | 0.194626412330238 | 0.000137301255499431 | 0.00127692743623017 | 379 | 379 |
| C1orf141 | -0.0561407717564479 | 0.275627696140707 | 0.431890782288442 | 379 | 90 |
| C1orf144 | 0.319343355446488 | 1.96813449567146E-10 | 3.82589909609547E-08 | 379 | 379 |
| C1orf146 | 0.00618224181838842 | 0.904515630078447 | 0.945043022993739 | 379 | 24 |
| C1orf14 | 0.0743457318241814 | 0.14857705097553 | 0.279160974769526 | 379 | 28 |
| C1orf150 | 0.0718607201926926 | 0.162665292577909 | 0.297974004526201 | 379 | 291 |
| C1orf151 | 0.0731263165695166 | 0.155369724875318 | 0.288344337778717 | 379 | 379 |
| C1orf152 | -0.0587311528784327 | 0.254044102754677 | 0.408266045503301 | 379 | 376 |
| C1orf156 | -0.114157960103429 | 0.0262598624034428 | 0.0767521589399177 | 379 | 379 |
| C1orf157 | -0.0873276435889944 | 0.0895612259989922 | 0.193592062477708 | 379 | 62 |
| C1orf158 | 0.0540221927858841 | 0.294181765684362 | 0.452619974623135 | 379 | 22 |
| C1orf159 | 0.148602701934652 | 0.00373704425703896 | 0.0171444038705619 | 379 | 379 |
| C1orf161 | -0.244914235001485 | 1.39362094136155E-06 | 0.0000383041262136998 | 379 | 377 |
| C1orf162 | 0.249289251479841 | 8.88178464855614E-07 | 0.0000267641376917281 | 379 | 379 |
| C1orf163 | 0.0829071507778561 | 0.107076472080041 | 0.219707773387933 | 379 | 379 |
| C1orf168 | 0.0406152557436738 | 0.430457968272352 | 0.587383650295481 | 379 | 290 |
| C1orf170 | 0.222234901905587 | 0.000012602862814331 | 0.000207033607193501 | 379 | 379 |
| C1orf172 | 0.0913445624396035 | 0.0757115477215912 | 0.170147179896148 | 379 | 379 |
| C1orf173 | 0.161784502908643 | 0.00157743631525856 | 0.00871992265691665 | 379 | 185 |
| C1orf174 | 0.149292649002659 | 0.00357791157829517 | 0.0165676858417648 | 379 | 379 |
| C1orf175 | -0.139021196896255 | 0.00671436107928051 | 0.0270235780742927 | 379 | 379 |
| C1orf177 | 0.0977051311220496 | 0.0573830085007126 | 0.138358557159371 | 379 | 321 |
| C1orf180 | -0.246016526110406 | 1.24507838448364E-06 | 0.0000351050636811481 | 379 | 356 |
| C1orf182 | 0.0917253598609661 | 0.0744943270012893 | 0.168154999519759 | 379 | 370 |
| C1orf183 | 0.151263558225046 | 0.00315657274342136 | 0.0149661703387276 | 379 | 379 |
| C1orf185 | 0.0965111212582165 | 0.0605114360417924 | 0.14394651650406 | 379 | 6 |
| C1orf186 | 0.0479575736879592 | 0.351808975075955 | 0.513067693277878 | 379 | 326 |
| C1orf187 | -0.0490964921275689 | 0.340476354050363 | 0.50181856449198 | 379 | 345 |
| C1orf189 | -0.0324799067505729 | 0.528436836020872 | 0.675075419407374 | 379 | 67 |
| C1orf190 | 0.0900513927193021 | 0.0799663255706088 | 0.17753580824253 | 379 | 373 |
| C1orf192 | -0.0436711446471496 | 0.396559014920595 | 0.556158731634288 | 379 | 376 |
| C1orf194 | 0.038365815855009 | 0.456447175908504 | 0.610773019564976 | 379 | 229 |
| C1orf198 | 0.102997551166463 | 0.0450853621579994 | 0.1156043246191 | 379 | 379 |
| C1orf200 | 0.1426262557941 | 0.00540750538446771 | 0.0227885264108875 | 379 | 194 |
| C1orf201 | 0.153461837580069 | 0.00274015484829882 | 0.0134218849634558 | 379 | 379 |
| C1orf203 | -0.108654474038065 | 0.034467998994685 | 0.0943705446101371 | 379 | 379 |
| C1orf204 | -0.148827892724174 | 0.00368441438224157 | 0.0169657613495323 | 379 | 378 |
| C1orf210 | -0.0350793648548143 | 0.495949874245017 | 0.646761454128007 | 379 | 379 |
| C1orf212 | 0.133301412663042 | 0.00937318590842503 | 0.0348100557383481 | 379 | 379 |
| C1orf213 | 0.0303980360291099 | 0.555211977295468 | 0.697742931322176 | 379 | 379 |
| C1orf216 | 0.20661559403587 | 0.000050585481941385 | 0.000597384714671699 | 379 | 379 |
| C1orf21 | 0.0313865690159472 | 0.542416370682099 | 0.687310314281996 | 379 | 379 |
| C1orf220 | -0.0766641795704136 | 0.13628852054381 | 0.261926466285809 | 379 | 377 |
| C1orf223 | 0.0438850221788928 | 0.394248145560591 | 0.55401504111803 | 379 | 186 |
| C1orf226 | 0.0882542206880036 | 0.0861985768710911 | 0.188273340185062 | 379 | 379 |
| C1orf227 | 0.0576678755092186 | 0.26275743178374 | 0.417397400849863 | 379 | 160 |
| C1orf228 | 0.226661773399325 | 8.34299500148554E-06 | 0.000151349409779922 | 379 | 368 |
| C1orf229 | -0.236484127951726 | 3.24237687638031E-06 | 0.0000730566462555326 | 379 | 361 |
| C1orf230 | -0.151909729877277 | 0.00302857127880637 | 0.0145260066076857 | 379 | 315 |
| C1orf25 | -0.0984309578331265 | 0.0555475998088682 | 0.135240398945265 | 379 | 379 |
| C1orf26 | -0.0946189683826594 | 0.0657564436100159 | 0.152958559819263 | 379 | 379 |
| C1orf27 | -0.168052371204555 | 0.00102268429220715 | 0.00626106798675438 | 379 | 379 |
| C1orf31 | -0.00152035015717184 | 0.976465572984868 | 0.987522155520961 | 379 | 379 |
| C1orf35 | 0.0235355984249821 | 0.647858655929673 | 0.768587911058312 | 379 | 379 |
| C1orf38 | 0.119181996921015 | 0.0202955078782264 | 0.0628780203452301 | 379 | 379 |
| C1orf43 | -0.148361703393601 | 0.00379412175976533 | 0.0173300728524826 | 379 | 379 |
| C1orf49 | 0.0550585950486418 | 0.285003425039901 | 0.44266394420266 | 379 | 105 |
| C1orf50 | -0.0424394921227815 | 0.410024300730368 | 0.568813414550588 | 379 | 379 |
| C1orf51 | 0.0106699230318845 | 0.835977847754315 | 0.901690081339964 | 379 | 379 |
| C1orf52 | 0.0810518787911521 | 0.115188989938746 | 0.231616194352038 | 379 | 379 |
| C1orf53 | -0.0791135542602538 | 0.124170966570702 | 0.24471344052916 | 379 | 379 |
| C1orf54 | 0.185577458180138 | 0.000280715146655396 | 0.00225021491610066 | 379 | 379 |
| C1orf55 | -0.185424835357059 | 0.000284041969411475 | 0.00226730441605907 | 379 | 379 |
| C1orf56 | -0.120804130818438 | 0.0186397079990932 | 0.0590002620004581 | 379 | 379 |
| C1orf57 | -0.0709901331465413 | 0.167831272125532 | 0.304241720600474 | 379 | 379 |
| C1orf58 | -0.0913748946920461 | 0.0756140016795792 | 0.169985762505975 | 379 | 379 |
| C1orf59 | -0.110928114570817 | 0.0308444312444485 | 0.0866756494777389 | 379 | 379 |
| C1orf61 | 0.163947845361181 | 0.00136060862798167 | 0.00777468238490506 | 379 | 275 |
| C1orf63 | -0.0402590185238022 | 0.434515866220332 | 0.5913233078529 | 379 | 379 |
| C1orf64 | 0.0842925697414127 | 0.101317877321726 | 0.211611925891286 | 379 | 75 |
| C1orf65 | 0.0268071403511771 | 0.602890053830592 | 0.734371789369271 | 379 | 20 |
| C1orf66 | -0.0740116287633657 | 0.150415276830297 | 0.281467922705845 | 379 | 379 |
| C1orf68 | 0.127653555325165 | 0.0128780630630888 | 0.0443925998635127 | 379 | 3 |
| C1orf69 | 0.0479978320875216 | 0.351404378473875 | 0.512703901131713 | 379 | 379 |
| C1orf70 | 0.0217981249811988 | 0.672285977315806 | 0.78592573304745 | 379 | 366 |
| C1orf74 | -0.0620668806161264 | 0.228016324943931 | 0.377703232329846 | 379 | 379 |
| C1orf77 | -0.0350944818767858 | 0.495764102728499 | 0.646668681593361 | 379 | 379 |
| C1orf83 | -0.0771564456382141 | 0.13378288029952 | 0.258592995767097 | 379 | 379 |
| C1orf84 | 0.0589638407615291 | 0.252164300759852 | 0.406217353237089 | 379 | 379 |
| C1orf85 | 0.0419596001348931 | 0.415343244287144 | 0.573697377062033 | 379 | 379 |
| C1orf86 | 0.117224100642853 | 0.0224630255311937 | 0.0679907347098666 | 379 | 379 |
| C1orf87 | 0.00358274038758637 | 0.944577031960354 | 0.96856148263484 | 379 | 135 |
| C1orf88 | -0.0321845973057286 | 0.532194605390122 | 0.67860801515597 | 379 | 374 |
| C1orf89 | 0.0614253214513523 | 0.232869299672287 | 0.383818160756618 | 379 | 379 |
| C1orf91 | -0.0384660567336852 | 0.455270639440793 | 0.609610091763374 | 379 | 379 |
| C1orf92 | 0.0988229697755061 | 0.0545767481491287 | 0.133384415419811 | 379 | 115 |
| C1orf93 | 0.0985772765787443 | 0.0551835653749898 | 0.134635134029199 | 379 | 379 |
| C1orf94 | -0.00244617268543403 | 0.962142889735556 | 0.979783740828751 | 379 | 86 |
| C1orf95 | -0.0889276581337603 | 0.0838189342480564 | 0.184212129047934 | 379 | 345 |
| C1orf96 | 0.0738584014932201 | 0.151264083675816 | 0.282682775789264 | 379 | 379 |
| C1orf97 | 0.0282989121568008 | 0.58286048895941 | 0.719118771394884 | 379 | 379 |
| C1orf9 | -0.10211074309011 | 0.0469764100393657 | 0.11933119545492 | 379 | 379 |
| C20orf103 | 0.111957963900638 | 0.0293134706277492 | 0.0833779221929438 | 379 | 375 |
| C20orf106 | 0.0476128580996036 | 0.355285385098815 | 0.516276923102918 | 379 | 270 |
| C20orf107 | -0.00242936058831669 | 0.962402882172146 | 0.979789324642834 | 379 | 340 |
| C20orf108 | -0.37940015831668 | 2.01572171165807E-14 | 3.63343000897783E-11 | 379 | 379 |
| C20orf111 | -0.254856790979838 | 4.94547382544469E-07 | 0.0000172943306897561 | 379 | 379 |
| C20orf112 | -0.274587873074536 | 5.54845381961659E-08 | 3.15228488066928E-06 | 379 | 379 |
| C20orf114 | 0.0562080985903938 | 0.275051399421538 | 0.431430990248418 | 379 | 82 |
| C20orf117 | -0.130048650661244 | 0.0112709272359515 | 0.0399928320033012 | 379 | 379 |
| C20orf118 | -0.113635848758865 | 0.026958621873599 | 0.0784017656470362 | 379 | 379 |
| C20orf11 | -0.263281946836924 | 1.98582896629454E-07 | 8.37766313695494E-06 | 379 | 379 |
| C20orf123 | -0.0881511756684857 | 0.086567443057883 | 0.188850177241908 | 379 | 212 |
| C20orf12 | -0.175106212443209 | 0.00061664268206324 | 0.00418722788038547 | 379 | 379 |
| C20orf132 | -0.113075160698293 | 0.0277267552976904 | 0.0801057998022155 | 379 | 379 |
| C20orf134 | -0.27817898398076 | 3.65529678532345E-08 | 2.37630244784896E-06 | 379 | 379 |
| C20orf135 | -0.102418286188411 | 0.0463132068536473 | 0.118018026666768 | 379 | 379 |
| C20orf141 | 0.111751245425365 | 0.0296154420118361 | 0.084092078506471 | 379 | 91 |
| C20orf144 | -0.0665825979084648 | 0.195880066885074 | 0.339740199982264 | 379 | 130 |
| C20orf151 | -0.120133738429799 | 0.0193091745692359 | 0.0606851027672864 | 379 | 379 |
| C20orf152 | -0.12836380609486 | 0.0123816242483562 | 0.0430933553793939 | 379 | 307 |
| C20orf160 | 0.127531447066933 | 0.01296515691301 | 0.0446540092532852 | 379 | 379 |
| C20orf165 | -0.181535564666741 | 0.000382309662752295 | 0.0028519322772959 | 379 | 378 |
| C20orf166 | 0.00277827795105921 | 0.95700787021082 | 0.976750747999632 | 379 | 59 |
| C20orf173 | -0.093250429820347 | 0.069778079182937 | 0.160189852268065 | 379 | 49 |
| C20orf177 | -0.197269468810954 | 0.000110727862622313 | 0.00108037151927688 | 379 | 379 |
| C20orf186 | -0.0135820738948427 | 0.792123985311349 | 0.872811024215251 | 379 | 39 |
| C20orf191 | 0.0385941764288501 | 0.453769365583504 | 0.608380484197019 | 379 | 48 |
| C20orf194 | 0.11989671392223 | 0.0195508297222655 | 0.0612020605830566 | 379 | 379 |
| C20orf195 | 0.0550495504026539 | 0.285082680380443 | 0.442717683786295 | 379 | 373 |
| C20orf196 | -0.166723377022143 | 0.00112252877696805 | 0.00673523381573647 | 379 | 379 |
| C20orf197 | 0.0404875986449223 | 0.431909585519325 | 0.5887156040822 | 379 | 285 |
| C20orf199 | -0.171495911423994 | 0.000800819761227028 | 0.00515540721610698 | 379 | 379 |
| C20orf200 | -0.038805577521691 | 0.451298313061 | 0.606297374576428 | 379 | 321 |
| C20orf201 | -0.0198492769112303 | 0.700099830396208 | 0.806131210052033 | 379 | 92 |
| C20orf202 | 0.0123453181558458 | 0.810676844390171 | 0.885137691110589 | 379 | 378 |
| C20orf203 | -0.0098675902171711 | 0.848156130332577 | 0.909974555069225 | 379 | 233 |
| C20orf20 | -0.101270076220267 | 0.0488299914745782 | 0.122665788794873 | 379 | 379 |
| C20orf24 | -0.234698549443412 | 3.86205473606998E-06 | 0.0000835991498982485 | 379 | 379 |
| C20orf26 | 0.191872458117262 | 0.00017128106376616 | 0.00152960470431685 | 379 | 319 |
| C20orf27 | 0.184362009823254 | 0.000308249347320645 | 0.00240657886923184 | 379 | 379 |
| C20orf29 | 0.0940186825909961 | 0.0674964012106345 | 0.156053946269177 | 379 | 379 |
| C20orf30 | 0.0422201535087211 | 0.412450346903589 | 0.570830540794262 | 379 | 379 |
| C20orf3 | -0.0638091654763556 | 0.215200386721069 | 0.36243021322628 | 379 | 379 |
| C20orf43 | -0.270508426607438 | 8.84956797848575E-08 | 4.59343544181716E-06 | 379 | 379 |
| C20orf46 | 0.109955372690918 | 0.0323527789727805 | 0.0900085451764125 | 379 | 379 |
| C20orf4 | -0.30076457296644 | 0.0000000023034536489 | 0.0000002890688541166 | 379 | 379 |
| C20orf54 | 0.0831371847774339 | 0.106102829332851 | 0.218056270730904 | 379 | 379 |
| C20orf56 | -0.0774999874545099 | 0.132055383442429 | 0.256102713507089 | 379 | 379 |
| C20orf70 | 0.147840788292983 | 0.00392019870578487 | 0.0178034127206373 | 379 | 46 |
| C20orf72 | -0.00706632341899556 | 0.890940732838139 | 0.936867461323431 | 379 | 379 |
| C20orf7 | 0.0579367232987042 | 0.260535110461665 | 0.414631203967726 | 379 | 379 |
| C20orf85 | -0.0639777313803564 | 0.213988439558202 | 0.36101104224964 | 379 | 133 |
| C20orf94 | 0.0397842031408042 | 0.439958550923726 | 0.596315411013441 | 379 | 379 |
| C20orf96 | -0.000412312332257399 | 0.99361671179 | 0.996418049072748 | 379 | 379 |
| C21orf119 | -0.0330244091470869 | 0.521543587413158 | 0.669201206964868 | 379 | 379 |
| C21orf121 | -0.00688296729599332 | 0.893753647859417 | 0.938779855366664 | 379 | 185 |
| C21orf122 | 0.0327856881508949 | 0.524560046449881 | 0.67168076209288 | 379 | 379 |
| C21orf125 | 0.0434564694364111 | 0.398886672163736 | 0.558238631822597 | 379 | 369 |
| C21orf128 | 0.0873540212052243 | 0.0894640647951632 | 0.19342421510833 | 379 | 149 |
| C21orf129 | 0.184547845822808 | 0.000303882423634635 | 0.00238157339756029 | 379 | 263 |
| C21orf130 | -0.0644474303277464 | 0.210637308243945 | 0.357242027872803 | 379 | 337 |
| C21orf15 | 0.0841339629192808 | 0.101964416073994 | 0.21236874389865 | 379 | 305 |
| C21orf29 | -0.148453093027978 | 0.00377238503341305 | 0.0172665859747262 | 379 | 358 |
| C21orf2 | -0.0723301629761765 | 0.159929715829432 | 0.294464333314698 | 379 | 379 |
| C21orf33 | 0.00992891148569823 | 0.847224045796774 | 0.909417409054701 | 379 | 379 |
| C21orf34 | -0.0606186910010305 | 0.239073883505425 | 0.390925033988583 | 379 | 310 |
| C21orf45 | 0.117594740475243 | 0.0220379590858968 | 0.0670194460070442 | 379 | 379 |
| C21orf49 | -0.214514564999661 | 0.0000253696904874432 | 0.000349084124208899 | 379 | 369 |
| C21orf54 | -0.0336345756176828 | 0.513874173879297 | 0.66278676793275 | 379 | 54 |
| C21orf56 | -0.115387135021396 | 0.0246760953094395 | 0.0730919656103325 | 379 | 378 |
| C21orf57 | 0.015253782354666 | 0.767233315316066 | 0.855608724907869 | 379 | 379 |
| C21orf58 | 0.0477717564164935 | 0.353680243123996 | 0.514887801810763 | 379 | 379 |
| C21orf59 | -0.0226763613406628 | 0.659893643771772 | 0.776198087957922 | 379 | 379 |
| C21orf62 | -0.0718162448799724 | 0.162926274977154 | 0.298374635655954 | 379 | 326 |
| C21orf63 | 0.0660578534816322 | 0.199434917937583 | 0.344364324083693 | 379 | 379 |
| C21orf67 | 0.112830727439479 | 0.0280674643211184 | 0.0808075624450612 | 379 | 379 |
| C21orf70 | 0.0718541744019675 | 0.162703683739373 | 0.297994517013143 | 379 | 379 |
| C21orf71 | 0.0575089932298123 | 0.264076878975603 | 0.418922822332047 | 379 | 250 |
| C21orf7 | 0.068555510024259 | 0.18292850515618 | 0.323357974524092 | 379 | 379 |
| C21orf81 | -0.153557410814886 | 0.00272323998972393 | 0.0133720660020421 | 379 | 294 |
| C21orf82 | 0.156638664479828 | 0.00222629391257425 | 0.0113594842250443 | 379 | 360 |
| C21orf84 | -0.0640819781679172 | 0.213241389312834 | 0.360149085800246 | 379 | 101 |
| C21orf88 | 0.0481740982965032 | 0.349636363562094 | 0.511064490726811 | 379 | 328 |
| C21orf90 | -0.00558551752095664 | 0.913694197740319 | 0.950510417250527 | 379 | 319 |
| C21orf91 | -0.00264306357625803 | 0.959098355684171 | 0.977838451074956 | 379 | 379 |
| C21orf96 | 0.00149135113871118 | 0.976914340472679 | 0.98768126452455 | 379 | 156 |
| C21orf99 | 0.0564751142682973 | 0.272773899965292 | 0.428944475256706 | 379 | 155 |
| C22orf13 | 0.145157796948028 | 0.00463160009609473 | 0.020173745086596 | 379 | 379 |
| C22orf15 | 0.172127942559244 | 0.000765286815697911 | 0.00499093007929626 | 379 | 339 |
| C22orf23 | -0.0834386923186366 | 0.104837271786187 | 0.216284822076424 | 379 | 379 |
| C22orf24 | 0.101608900962867 | 0.0480756902652465 | 0.121355160608442 | 379 | 344 |
| C22orf25 | 0.149650047168637 | 0.00349791659396888 | 0.0162694558351431 | 379 | 379 |
| C22orf26 | 0.0561793268481086 | 0.275297576243456 | 0.431671036551643 | 379 | 376 |
| C22orf27 | 0.0575691622886455 | 0.263576667116717 | 0.418430596924762 | 379 | 379 |
| C22orf28 | 0.0553970426979658 | 0.282048383923001 | 0.439371385584374 | 379 | 379 |
| C22orf29 | -0.165805340686961 | 0.00119665915201837 | 0.00705751269072581 | 379 | 379 |
| C22orf30 | 0.13939174260955 | 0.00656807937613874 | 0.0265833594345946 | 379 | 379 |
| C22orf31 | -0.0574348682508707 | 0.264694008457789 | 0.419633229367638 | 379 | 255 |
| C22orf32 | -0.128815682836283 | 0.012074638058035 | 0.042247383697674 | 379 | 379 |
| C22orf33 | 0.0605073252483292 | 0.23993954727814 | 0.39204955446485 | 379 | 16 |
| C22orf34 | 0.09071902936084 | 0.077746116083303 | 0.173500280213813 | 379 | 290 |
| C22orf36 | -0.00779192913240041 | 0.879822762173555 | 0.929678475176698 | 379 | 379 |
| C22orf39 | -0.0356949668088245 | 0.48841482053372 | 0.639969258446314 | 379 | 379 |
| C22orf40 | 0.0966858030048821 | 0.0600451454276429 | 0.143166804177405 | 379 | 379 |
| C22orf41 | 0.0564221824063961 | 0.273224355924326 | 0.429537911027563 | 379 | 331 |
| C22orf42 | 0.143122028677318 | 0.00524692951664455 | 0.0222775414252737 | 379 | 8 |
| C22orf43 | 0.0768775714896737 | 0.135197958283373 | 0.260313178951517 | 379 | 290 |
| C22orf45 | -0.0717560096017041 | 0.163280238598507 | 0.298769818976651 | 379 | 347 |
| C22orf46 | 0.0697461801757536 | 0.175424352035988 | 0.314182463388092 | 379 | 379 |
| C22orf9 | 0.185346265272575 | 0.000285768926032378 | 0.00227742213238343 | 379 | 379 |
| C2CD2L | 0.0574619636775147 | 0.264468310059209 | 0.419377664871317 | 379 | 379 |
| C2CD2 | 0.102610977326431 | 0.045901691292369 | 0.117165130657195 | 379 | 379 |
| C2CD3 | 0.159495690522171 | 0.00184097375986141 | 0.00981258809422904 | 379 | 379 |
| C2CD4A | 0.010001883510944 | 0.846115146925726 | 0.908768275458713 | 379 | 379 |
| C2CD4B | 0.105190443427705 | 0.0406819973373586 | 0.107052772820855 | 379 | 378 |
| C2CD4C | 0.200011084294269 | 0.0000883194945472536 | 0.000914464197327908 | 379 | 377 |
| C2CD4D | -0.204842915156791 | 0.0000588504466072837 | 0.000672945014607394 | 379 | 379 |
| C2orf14 | 0.131342166588978 | 0.0104789581416425 | 0.0378671007895913 | 379 | 56 |
| C2orf15 | -0.16590920279101 | 0.0011880520004463 | 0.00701718649533789 | 379 | 379 |
| C2orf16 | 0.149421928529552 | 0.00354878608749975 | 0.0164558771148141 | 379 | 377 |
| C2orf18 | 0.192461140808315 | 0.000163415195385641 | 0.00147501692439297 | 379 | 379 |
| C2orf24 | -0.148392326283111 | 0.00378682560900008 | 0.0173206870069789 | 379 | 379 |
| C2orf27A | -0.0215980169858629 | 0.675122198363374 | 0.787919696529416 | 379 | 379 |
| C2orf27B | -0.0844260616791418 | 0.100776240099516 | 0.210735212897406 | 379 | 5 |
| C2orf28 | -0.0405228998837085 | 0.431507884964183 | 0.588393976521646 | 379 | 379 |
| C2orf29 | 0.0190128297877164 | 0.712164240232617 | 0.815570784066786 | 379 | 379 |
| C2orf34 | -0.140675453765106 | 0.00608312429781358 | 0.0250189148676722 | 379 | 379 |
| C2orf39 | 0.0639632956410618 | 0.21409203656007 | 0.361124364178058 | 379 | 246 |
| C2orf3 | -0.0155874077633474 | 0.762293449034589 | 0.852158685833113 | 379 | 379 |
| C2orf40 | -0.0141670416140173 | 0.783388734287996 | 0.866701920737774 | 379 | 303 |
| C2orf42 | -0.0106979285456625 | 0.835553452228499 | 0.901379184099462 | 379 | 379 |
| C2orf43 | 0.0353140473717764 | 0.493070060948917 | 0.644256551465907 | 379 | 379 |
| C2orf44 | 0.136053568984632 | 0.00799514627849551 | 0.0308419522642986 | 379 | 379 |
| C2orf47 | 0.0374614057712373 | 0.467138959384169 | 0.620182878250372 | 379 | 379 |
| C2orf48 | 0.0121214947256853 | 0.814046150876873 | 0.887399366627448 | 379 | 376 |
| C2orf49 | -0.0267199174096262 | 0.604070660337864 | 0.735042224803876 | 379 | 379 |
| C2orf50 | 0.0934939160880241 | 0.069048206456267 | 0.158771638364242 | 379 | 344 |
| C2orf51 | 0.11689169406639 | 0.0228502640934029 | 0.0688982719653273 | 379 | 8 |
| C2orf52 | -0.0200913932468161 | 0.696621550823447 | 0.803385802927197 | 379 | 362 |
| C2orf53 | -0.0975000444790897 | 0.0579106227587426 | 0.139266443670145 | 379 | 8 |
| C2orf54 | -0.134267500801103 | 0.00886711049132583 | 0.033342891489097 | 379 | 378 |
| C2orf55 | -0.0627364581259679 | 0.223028406432826 | 0.37180151696234 | 379 | 379 |
| C2orf56 | -0.00658491268202848 | 0.898329009068027 | 0.941567552827765 | 379 | 379 |
| C2orf57 | 0.0221041660350978 | 0.667957301339903 | 0.782572522510493 | 379 | 37 |
| C2orf58 | 0.0223382100581441 | 0.664654356241865 | 0.779762533315407 | 379 | 342 |
| C2orf60 | -0.081374790495342 | 0.113743393828232 | 0.229594218958179 | 379 | 379 |
| C2orf61 | 0.0825001455522074 | 0.108816422839647 | 0.222388376836171 | 379 | 325 |
| C2orf62 | 0.100328155795631 | 0.0509790767940611 | 0.126719057620699 | 379 | 255 |
| C2orf63 | -0.176650552541316 | 0.000550562851944514 | 0.00381697910082371 | 379 | 379 |
| C2orf64 | -0.102201050595923 | 0.0467808456115776 | 0.118934556582429 | 379 | 379 |
| C2orf65 | 0.133904959292297 | 0.00905410178527343 | 0.0338505017833379 | 379 | 347 |
| C2orf66 | -0.105947347737121 | 0.0392489565192889 | 0.104208397143072 | 379 | 295 |
| C2orf67 | -0.0960232569880574 | 0.061829581086569 | 0.146237148422148 | 379 | 379 |
| C2orf68 | -0.0660870447642301 | 0.199235938354537 | 0.344085897194823 | 379 | 379 |
| C2orf69 | 0.0849754372863863 | 0.0985713247448063 | 0.207613365948589 | 379 | 379 |
| C2orf70 | -0.0581948868019584 | 0.258413329088044 | 0.412577510986049 | 379 | 378 |
| C2orf71 | 0.0315484066980414 | 0.540335572754887 | 0.685374471378192 | 379 | 43 |
| C2orf72 | 0.0450417255102481 | 0.381891626132681 | 0.541796448408614 | 379 | 379 |
| C2orf73 | 0.00968547712278638 | 0.850925495199315 | 0.911629254249415 | 379 | 158 |
| C2orf74 | 0.0944069394131456 | 0.0663667653665636 | 0.153962820134342 | 379 | 379 |
| C2orf76 | -0.0936075735855173 | 0.0687096524132981 | 0.158142631584012 | 379 | 379 |
| C2orf77 | 0.00886916886217763 | 0.863361006926802 | 0.919866848218411 | 379 | 379 |
| C2orf78 | 0.152053680743083 | 0.00300070597131584 | 0.0144237570907274 | 379 | 89 |
| C2orf79 | 0.0339893386041011 | 0.509442045489912 | 0.658579483593298 | 379 | 379 |
| C2orf7 | 0.0188165951821274 | 0.715005166158265 | 0.817926639046102 | 379 | 379 |
| C2orf80 | -0.0186677732266502 | 0.717162317650803 | 0.819329909719828 | 379 | 43 |
| C2orf81 | -0.101515855273608 | 0.0482818535287025 | 0.121720609252017 | 379 | 379 |
| C2orf82 | -0.164507474292955 | 0.00130916271306936 | 0.00753718881380349 | 379 | 374 |
| C2orf83 | -0.061366889763307 | 0.23331489588779 | 0.384360896947749 | 379 | 44 |
| C2orf84 | 0.00863612726005163 | 0.866917462014175 | 0.922074934718473 | 379 | 307 |
| C2orf85 | 0.215983727857332 | 0.0000222499686526551 | 0.000316934179917274 | 379 | 184 |
| C2orf86 | -0.0421723065033624 | 0.412980693752514 | 0.571269791804441 | 379 | 379 |
| C2orf88 | 0.104490597363041 | 0.0420460700036264 | 0.109673669063629 | 379 | 379 |
| C2orf89 | -0.195712283793197 | 0.000125731286788199 | 0.00118884117998875 | 379 | 379 |
| C2 | -0.0499733045312656 | 0.331912334658897 | 0.492749159300435 | 379 | 379 |
| C3AR1 | 0.206974206621198 | 0.0000490525661615207 | 0.00058395527395298 | 379 | 379 |
| C3P1 | 0.0239328717941088 | 0.642324802499423 | 0.764879958198219 | 379 | 166 |
| C3orf10 | -0.0622763296763928 | 0.226447629952288 | 0.375963095674954 | 379 | 379 |
| C3orf14 | -0.00680584814249205 | 0.89493715312635 | 0.939440304595047 | 379 | 379 |
| C3orf15 | -0.0831459938965777 | 0.106065683301726 | 0.218025126322478 | 379 | 350 |
| C3orf16 | 0.0316335978387603 | 0.53924184329395 | 0.684469888867476 | 379 | 34 |
| C3orf17 | -0.212858516969639 | 0.0000293820063775613 | 0.000393108247270099 | 379 | 379 |
| C3orf18 | -0.12614393334704 | 0.0139918545433468 | 0.0473269348149914 | 379 | 379 |
| C3orf19 | -0.202016385530653 | 0.0000747117266262297 | 0.000804225904204605 | 379 | 379 |
| C3orf1 | -0.00496290091060979 | 0.923283249079446 | 0.956109212962622 | 379 | 379 |
| C3orf20 | 0.100929191362859 | 0.0495988397447094 | 0.124172448795214 | 379 | 132 |
| C3orf21 | 0.218485994474902 | 0.0000177569992648495 | 0.000268767772078959 | 379 | 379 |
| C3orf22 | -0.069028795205657 | 0.179917753007389 | 0.319606358107645 | 379 | 10 |
| C3orf23 | -0.312456079278533 | 4.99795293418342E-10 | 0.000000083856945326 | 379 | 379 |
| C3orf24 | -0.00977572129285948 | 0.849552935332831 | 0.910603499640586 | 379 | 61 |
| C3orf26 | -0.0457073079759457 | 0.374890002483074 | 0.535722192873601 | 379 | 379 |
| C3orf27 | -0.0448473144841453 | 0.383951721728999 | 0.543901888864942 | 379 | 17 |
| C3orf30 | 0.107390239595295 | 0.0366352293069581 | 0.0988455896086411 | 379 | 24 |
| C3orf31 | -0.235762008752512 | 3.48059117687997E-06 | 0.0000771959304867742 | 379 | 379 |
| C3orf32 | -0.068250564662976 | 0.184887986395786 | 0.325863021711614 | 379 | 379 |
| C3orf33 | -0.0257948587483965 | 0.616654878599648 | 0.744548504236058 | 379 | 379 |
| C3orf34 | 0.0257721706587768 | 0.616964954993783 | 0.74466372502931 | 379 | 379 |
| C3orf35 | -0.100510301819153 | 0.0505574475088077 | 0.125952138359673 | 379 | 379 |
| C3orf36 | 0.0106386691605675 | 0.836451525041012 | 0.90190662018126 | 379 | 376 |
| C3orf37 | -0.0943116813003168 | 0.0666424733417839 | 0.154530108925376 | 379 | 379 |
| C3orf38 | -0.184448376637972 | 0.000306212601677907 | 0.00239510195900179 | 379 | 379 |
| C3orf39 | 0.034918937935604 | 0.497923621654661 | 0.648632124707221 | 379 | 379 |
| C3orf42 | -0.167658977064667 | 0.00105135111834091 | 0.00641421229983497 | 379 | 378 |
| C3orf43 | -0.00450263841601647 | 0.930379074468125 | 0.96033560015232 | 379 | 122 |
| C3orf45 | -0.0129920003541317 | 0.800961889340743 | 0.878302861511351 | 379 | 303 |
| C3orf47 | -0.0971414302020685 | 0.0588428565774731 | 0.140978269721863 | 379 | 379 |
| C3orf48 | -0.0230448618635406 | 0.654721233813019 | 0.772669536747556 | 379 | 221 |
| C3orf49 | -0.075119097744363 | 0.144387618786726 | 0.273220798355879 | 379 | 199 |
| C3orf50 | -0.0322265085799448 | 0.531660470997687 | 0.677970533085224 | 379 | 41 |
| C3orf51 | 0.0793669980969687 | 0.122966510344635 | 0.242894995727577 | 379 | 33 |
| C3orf52 | -0.0066038601374871 | 0.898038049602797 | 0.94154566757517 | 379 | 379 |
| C3orf54 | 0.193641059942567 | 0.000148657816317217 | 0.00136147213946317 | 379 | 379 |
| C3orf55 | -0.144285186545489 | 0.00488690940646953 | 0.0210326980055302 | 379 | 337 |
| C3orf57 | 0.0501766147272684 | 0.329946535869291 | 0.490712564747697 | 379 | 341 |
| C3orf58 | -0.054227684004782 | 0.292346430493297 | 0.450714954033209 | 379 | 379 |
| C3orf59 | 0.136348497944597 | 0.00785876097755315 | 0.0304425819078916 | 379 | 379 |
| C3orf62 | -0.0433802082599904 | 0.399715516570776 | 0.55900403883237 | 379 | 379 |
| C3orf63 | -0.095943575759364 | 0.0620471015685824 | 0.146600325298123 | 379 | 379 |
| C3orf64 | -0.0348699056136416 | 0.498527700556392 | 0.649163147476991 | 379 | 379 |
| C3orf65 | -0.144773343830382 | 0.00474256329742528 | 0.0205582972763956 | 379 | 268 |
| C3orf66 | 0.000303951322819063 | 0.995294299599184 | 0.997016569980589 | 379 | 190 |
| C3orf67 | -0.171685662185063 | 0.000789994917596306 | 0.00510394891694349 | 379 | 376 |
| C3orf70 | -0.0357096533049214 | 0.488235809705381 | 0.63992954252186 | 379 | 379 |
| C3orf71 | -0.245416816761719 | 1.32390230013963E-06 | 0.0000368527087863068 | 379 | 379 |
| C3orf72 | 0.216517475029122 | 0.0000212094556185883 | 0.000306964296354284 | 379 | 155 |
| C3orf74 | 0.0421894494939045 | 0.412790630791527 | 0.571086563447837 | 379 | 99 |
| C3orf75 | -0.0840576629621275 | 0.102276605366476 | 0.212817770091981 | 379 | 379 |
| C3orf77 | 0.0881764447644178 | 0.086476870990274 | 0.188756428665252 | 379 | 4 |
| C3orf79 | -0.0242392302096688 | 0.638070801378173 | 0.761414771890131 | 379 | 4 |
| C3 | 0.100321319019244 | 0.0509949594141759 | 0.126719057620699 | 379 | 379 |
| C4A | 0.0267584149856195 | 0.603549448732881 | 0.734813856654523 | 379 | 379 |
| C4BPA | -0.0965083714303484 | 0.0605188001862887 | 0.14394651650406 | 379 | 377 |
| C4BPB | 0.0755384619983758 | 0.142153892883724 | 0.270160028364662 | 379 | 379 |
| C4orf10 | 0.120363141242441 | 0.0190777700310649 | 0.0601389545589753 | 379 | 379 |
| C4orf11 | -0.0296159384799772 | 0.565438466029345 | 0.705525032756968 | 379 | 9 |
| C4orf12 | 0.0229697732944081 | 0.655773876870361 | 0.773521477648082 | 379 | 375 |
| C4orf14 | 0.00700590989773109 | 0.891867403488723 | 0.937724379802243 | 379 | 379 |
| C4orf17 | 0.00309636337449127 | 0.952091288565158 | 0.973447433077397 | 379 | 4 |
| C4orf19 | -0.0589149833498887 | 0.252558198984205 | 0.406504096879521 | 379 | 379 |
| C4orf21 | 0.137494763756042 | 0.00734818949624809 | 0.0289719429969392 | 379 | 379 |
| C4orf22 | 0.0646665543161947 | 0.209086940556091 | 0.355494414109602 | 379 | 24 |
| C4orf23 | 0.143659801057775 | 0.00507761277240006 | 0.0217167614433021 | 379 | 379 |
| C4orf26 | 0.0658733642588896 | 0.200695816779702 | 0.345691307044374 | 379 | 188 |
| C4orf27 | 0.204202566405802 | 0.0000621374754624593 | 0.000702429796732978 | 379 | 379 |
| C4orf29 | -0.0222751876933214 | 0.665543124566589 | 0.780574297521964 | 379 | 378 |
| C4orf31 | -0.0176820933181588 | 0.731505638891153 | 0.829887240664301 | 379 | 378 |
| C4orf32 | 0.141217186096042 | 0.00588829475933991 | 0.0244560344550046 | 379 | 379 |
| C4orf33 | -0.149844553322146 | 0.00345506554477887 | 0.0161295952455281 | 379 | 379 |
| C4orf34 | -0.0294237454472817 | 0.567965256744422 | 0.707540431406564 | 379 | 379 |
| C4orf35 | 0.107593190624223 | 0.0362797174183768 | 0.0980982185969693 | 379 | 38 |
| C4orf36 | 0.129262953344251 | 0.0117774178218288 | 0.0414194112400179 | 379 | 376 |
| C4orf37 | -0.0859987358854111 | 0.0945667440939306 | 0.201577015899211 | 379 | 184 |
| C4orf38 | -0.00836190801292843 | 0.871105764071406 | 0.924739537959516 | 379 | 361 |
| C4orf39 | -0.0381417485801556 | 0.459083220130964 | 0.613265653086084 | 379 | 318 |
| C4orf3 | 0.221591705318957 | 0.0000133720683436505 | 0.000216973298787154 | 379 | 379 |
| C4orf40 | -0.0251348636797002 | 0.625702614080695 | 0.751586080571395 | 379 | 88 |
| C4orf41 | 0.0422343085809439 | 0.412293526045839 | 0.570757245998526 | 379 | 379 |
| C4orf42 | -0.0505414377432599 | 0.326437942553097 | 0.48687289939022 | 379 | 379 |
| C4orf43 | 0.013054611997383 | 0.800022889421568 | 0.877773131037653 | 379 | 379 |
| C4orf44 | -0.0239639462913372 | 0.641892775308445 | 0.764521354548154 | 379 | 359 |
| C4orf45 | -0.0845442518919998 | 0.100298609301743 | 0.209980025893248 | 379 | 20 |
| C4orf46 | 0.0821715182406627 | 0.110237497971386 | 0.224275508903822 | 379 | 379 |
| C4orf47 | 0.111293697838907 | 0.0302933093143198 | 0.0856000765404493 | 379 | 313 |
| C4orf48 | -0.111323455650577 | 0.0302488222183457 | 0.0855029520916618 | 379 | 379 |
| C4orf49 | 0.100043175487841 | 0.05164462717392 | 0.12784116730389 | 379 | 361 |
| C4orf50 | 0.115682382695641 | 0.0243081878365571 | 0.0722504494713319 | 379 | 156 |
| C4orf51 | -0.0397961783407427 | 0.439820805175291 | 0.596210222534742 | 379 | 11 |
| C4orf52 | -0.0237081388623445 | 0.645452834814641 | 0.766992617086462 | 379 | 379 |
| C4orf6 | 0.0767996179745176 | 0.135595566896539 | 0.260850771361654 | 379 | 37 |
| C4orf7 | 0.118509155080104 | 0.0210189554803426 | 0.0646446175374955 | 379 | 289 |
| C5AR1 | 0.215656299674607 | 0.0000229120286511641 | 0.000323115010025094 | 379 | 379 |
| C5orf13 | -0.0877077691242634 | 0.0881691967277502 | 0.191334008177502 | 379 | 379 |
| C5orf15 | 0.0575218340861443 | 0.263970072597078 | 0.418853921211176 | 379 | 379 |
| C5orf20 | 0.112347294053107 | 0.0287519030340332 | 0.0820986079145753 | 379 | 370 |
| C5orf22 | 0.00739431051338897 | 0.88591242677003 | 0.933709222239737 | 379 | 379 |
| C5orf23 | 0.0505304902055941 | 0.326542875020543 | 0.48687289939022 | 379 | 377 |
| C5orf24 | -0.10007732787394 | 0.0515644868070565 | 0.127722753830146 | 379 | 379 |
| C5orf25 | -0.15248673298242 | 0.0029182766151502 | 0.0141268527161128 | 379 | 379 |
| C5orf27 | 0.0155816512575469 | 0.762378602380148 | 0.852158685833113 | 379 | 315 |
| C5orf28 | -0.10059334584956 | 0.0503661862888685 | 0.125633506319749 | 379 | 379 |
| C5orf30 | -0.18518442969371 | 0.00028935696340722 | 0.00229494794817535 | 379 | 379 |
| C5orf32 | 0.013623314615261 | 0.791507276319273 | 0.872515578309588 | 379 | 379 |
| C5orf33 | 0.0612927471426126 | 0.233881169012234 | 0.38500587954957 | 379 | 379 |
| C5orf34 | -0.0480615780376437 | 0.350764332994454 | 0.512075027163709 | 379 | 379 |
| C5orf35 | -0.0440678007555289 | 0.392279735448808 | 0.552208022540047 | 379 | 379 |
| C5orf36 | -0.147385003439446 | 0.00403360773111963 | 0.0181893050017375 | 379 | 379 |
| C5orf38 | -0.0300214174517505 | 0.560125290545701 | 0.701545338951434 | 379 | 267 |
| C5orf39 | 0.22045030131399 | 0.0000148481023731427 | 0.000234214935445245 | 379 | 379 |
| C5orf40 | 0.0774605096180881 | 0.132253017566157 | 0.256459522016012 | 379 | 294 |
| C5orf41 | -0.169021309472119 | 0.000955122667060835 | 0.00593545014748542 | 379 | 379 |
| C5orf42 | -0.0941811883258596 | 0.0670216867930146 | 0.15519163911385 | 379 | 379 |
| C5orf43 | -0.0698194474262938 | 0.174970171933051 | 0.31365234328619 | 379 | 379 |
| C5orf44 | -0.179234531151353 | 0.000454498342078491 | 0.00326869536696856 | 379 | 379 |
| C5orf45 | -0.0965723402686899 | 0.0603476806413874 | 0.143703233543228 | 379 | 379 |
| C5orf46 | 0.110660391898337 | 0.0312534452019439 | 0.0875767822871881 | 379 | 332 |
| C5orf47 | -0.0231102966650209 | 0.653804480924312 | 0.771966607977566 | 379 | 97 |
| C5orf48 | -0.350878398893841 | 2.01901872101101E-12 | 1.25103447500645E-09 | 379 | 222 |
| C5orf49 | 0.00243409208625866 | 0.962329711022861 | 0.979789324642834 | 379 | 261 |
| C5orf4 | -0.187211142261876 | 0.000247311584138795 | 0.00204405756161069 | 379 | 379 |
| C5orf51 | -0.00574208282164099 | 0.911284814508993 | 0.948900078882697 | 379 | 379 |
| C5orf52 | -0.0393918333787975 | 0.444485435162053 | 0.599908725323698 | 379 | 355 |
| C5orf53 | -0.116716961642527 | 0.0230561259879464 | 0.0693502527440839 | 379 | 379 |
| C5orf54 | -0.167480286923837 | 0.00106461537430054 | 0.00648117704686248 | 379 | 379 |
| C5orf55 | 0.0145964967769488 | 0.776992931965394 | 0.862725583506123 | 379 | 379 |
| C5orf56 | 0.06854824034556 | 0.182975038698922 | 0.323403036520728 | 379 | 379 |
| C5orf58 | 0.120413647218032 | 0.0190271489226249 | 0.0600080020419605 | 379 | 330 |
| C5orf60 | 0.0630347661266234 | 0.22083142914362 | 0.369318958928787 | 379 | 112 |
| C5orf62 | 0.058735497490606 | 0.254008915435059 | 0.408242585332444 | 379 | 379 |
| C5 | 0.0931143623839292 | 0.070188693856395 | 0.160871739889562 | 379 | 379 |
| C6orf103 | -0.159214330626398 | 0.00187601187673709 | 0.00995736973698364 | 379 | 281 |
| C6orf105 | 0.0577904874166431 | 0.261742298394846 | 0.41611820819219 | 379 | 379 |
| C6orf106 | 0.209152767850677 | 0.0000406424825624294 | 0.00050651109003636 | 379 | 379 |
| C6orf108 | -0.00237997129547376 | 0.96316669099342 | 0.979900544371555 | 379 | 379 |
| C6orf10 | -0.0519795065459681 | 0.312843807228421 | 0.472578623322043 | 379 | 8 |
| C6orf114 | 0.0552404101938845 | 0.283413384984182 | 0.440988825038559 | 379 | 378 |
| C6orf115 | -0.0900737949430467 | 0.0798910042335891 | 0.17738844702616 | 379 | 379 |
| C6orf118 | 0.0830792148816552 | 0.106347531408849 | 0.218445960092682 | 379 | 48 |
| C6orf120 | -0.0457494725391416 | 0.374449128462102 | 0.535297571676031 | 379 | 379 |
| C6orf122 | 0.229098793830576 | 6.62456130614677E-06 | 0.000126057391149979 | 379 | 378 |
| C6orf123 | -0.0893503055772538 | 0.0823527303277728 | 0.181795806829112 | 379 | 378 |
| C6orf124 | -0.15829107071078 | 0.00199531613756003 | 0.0104232657129422 | 379 | 379 |
| C6orf125 | 0.023341022578138 | 0.650576128250496 | 0.770240866879987 | 379 | 379 |
| C6orf126 | -0.0237995056040772 | 0.644180353679491 | 0.766124121607549 | 379 | 152 |
| C6orf127 | 0.0232613904046333 | 0.651689620103565 | 0.770846613816947 | 379 | 22 |
| C6orf129 | -0.0198899763984071 | 0.699514694817607 | 0.805785009518592 | 379 | 379 |
| C6orf130 | 0.145184785284663 | 0.0046238992269549 | 0.0201456106069131 | 379 | 379 |
| C6orf132 | -0.00198783434424446 | 0.969232253643524 | 0.983517764853828 | 379 | 379 |
| C6orf134 | -0.134148804234246 | 0.00892794984559015 | 0.0335029987123389 | 379 | 379 |
| C6orf136 | 0.00450782250453224 | 0.930299120073893 | 0.96033560015232 | 379 | 379 |
| C6orf138 | -0.0656918581760502 | 0.201941972254471 | 0.347277140144116 | 379 | 358 |
| C6orf141 | 0.120405644697342 | 0.0190351618789688 | 0.0600237261030841 | 379 | 378 |
| C6orf142 | 0.144861141386294 | 0.00471701356424188 | 0.0204703315718512 | 379 | 207 |
| C6orf145 | -0.03634281755822 | 0.480551910665516 | 0.632430977932999 | 379 | 379 |
| C6orf146 | 0.0395676003561121 | 0.442454295071628 | 0.598203773946496 | 379 | 26 |
| C6orf147 | 0.0344993848261464 | 0.50310504170554 | 0.65276578765459 | 379 | 330 |
| C6orf150 | 0.106355923420511 | 0.0384933065576344 | 0.102738630020834 | 379 | 379 |
| C6orf153 | 0.16700269645525 | 0.00110082762774939 | 0.00664903856944095 | 379 | 379 |
| C6orf154 | 0.216848593313205 | 0.0000205873284211794 | 0.000300151138187607 | 379 | 379 |
| C6orf155 | -0.0423456481566456 | 0.411061248161744 | 0.569647919244553 | 379 | 306 |
| C6orf15 | -0.0858370133152504 | 0.0951908465436844 | 0.20257004744275 | 379 | 337 |
| C6orf162 | -0.0203790183862014 | 0.692497732006476 | 0.800891480977675 | 379 | 379 |
| C6orf163 | -0.100936375602324 | 0.049582532345434 | 0.124147297808469 | 379 | 374 |
| C6orf164 | -0.101499926370671 | 0.0483172215784642 | 0.121778806337586 | 379 | 371 |
| C6orf165 | 0.120991645673408 | 0.0184561103169056 | 0.0585516408581765 | 379 | 351 |
| C6orf167 | 0.169706344482848 | 0.000909868223905242 | 0.00569831558546846 | 379 | 379 |
| C6orf168 | -0.0759823715277341 | 0.139818326969756 | 0.266902646303679 | 379 | 378 |
| C6orf170 | -0.124736849655702 | 0.0151053646409761 | 0.0501690402179689 | 379 | 378 |
| C6orf174 | 0.101588864310143 | 0.0481200235410543 | 0.121436149519158 | 379 | 377 |
| C6orf176 | -0.00389745324464435 | 0.939717466014061 | 0.965467642312201 | 379 | 298 |
| C6orf182 | 0.126626598540725 | 0.0136268715298382 | 0.0462983772374381 | 379 | 379 |
| C6orf186 | 0.0278996224477178 | 0.588191258918511 | 0.72329032788311 | 379 | 309 |
| C6orf191 | -0.00119069406402801 | 0.981567491175146 | 0.989628904408961 | 379 | 34 |
| C6orf192 | 0.225512353992628 | 9.29359371178741E-06 | 0.000164971688556241 | 379 | 379 |
| C6orf195 | -0.0695140420082072 | 0.176869149738824 | 0.315999414400919 | 379 | 226 |
| C6orf1 | -0.134141599215716 | 0.00893165479087268 | 0.0335029987123389 | 379 | 379 |
| C6orf201 | 0.0280727715798172 | 0.585876852808444 | 0.72158309444598 | 379 | 360 |
| C6orf203 | -0.143148725204146 | 0.00523840578738471 | 0.0222550484882158 | 379 | 379 |
| C6orf204 | 0.1490981019171 | 0.00362214956418723 | 0.0167312611669622 | 379 | 378 |
| C6orf208 | 0.185920190938905 | 0.000273376445154033 | 0.00221064769759958 | 379 | 342 |
| C6orf211 | 0.0667420084630047 | 0.19480936440516 | 0.33835669914379 | 379 | 379 |
| C6orf217 | -0.0352214312517554 | 0.494205495897571 | 0.645272393827014 | 379 | 271 |
| C6orf218 | 0.0492243954389344 | 0.339218385097426 | 0.500377659301482 | 379 | 145 |
| C6orf221 | 0.0302229558146292 | 0.557493439193451 | 0.699471108762366 | 379 | 11 |
| C6orf222 | -0.223088813843032 | 0.0000116464484808008 | 0.000194545729130007 | 379 | 379 |
| C6orf223 | 0.038857854879257 | 0.450688418313557 | 0.605765317131318 | 379 | 379 |
| C6orf225 | -0.0380563880529604 | 0.460089676162585 | 0.614113638434987 | 379 | 379 |
| C6orf226 | -0.0496032152138272 | 0.335510023833068 | 0.496491734649009 | 379 | 379 |
| C6orf227 | -0.0495575402613781 | 0.335955763746535 | 0.497019840921058 | 379 | 321 |
| C6orf25 | -0.154273492851539 | 0.00259949778889028 | 0.0128792709040771 | 379 | 294 |
| C6orf26 | 0.0175284785236853 | 0.73374958672529 | 0.831246320009106 | 379 | 379 |
| C6orf27 | -0.00707035858972743 | 0.890878843450564 | 0.93685206618604 | 379 | 378 |
| C6orf35 | 0.135479974973295 | 0.0082664547109827 | 0.0316362215806534 | 379 | 379 |
| C6orf41 | 0.0476202130978596 | 0.355210986259827 | 0.516206642887705 | 379 | 343 |
| C6orf47 | 0.0182142670956509 | 0.723749624652646 | 0.824040059209972 | 379 | 379 |
| C6orf48 | -0.114966141813845 | 0.0252089985752771 | 0.0742735017377767 | 379 | 379 |
| C6orf52 | 0.120906339362243 | 0.0185394382182406 | 0.0587595877543599 | 379 | 354 |
| C6orf57 | -0.15713253418479 | 0.00215482414888095 | 0.0110746120331808 | 379 | 379 |
| C6orf58 | 0.0031500888511681 | 0.951261038950155 | 0.972899565704011 | 379 | 182 |
| C6orf59 | -0.010018348312467 | 0.845864986085008 | 0.908548805205501 | 379 | 308 |
| C6orf62 | 0.0234851731891753 | 0.64856245490388 | 0.76898262009413 | 379 | 379 |
| C6orf64 | -0.199756154522634 | 0.0000902077190484143 | 0.000929645869694366 | 379 | 379 |
| C6orf70 | -0.065192228815092 | 0.205401245028523 | 0.351427723395077 | 379 | 379 |
| C6orf72 | 0.0664871131456308 | 0.19652345676594 | 0.340498698073668 | 379 | 379 |
| C6orf81 | 0.0697195231913277 | 0.175589814537887 | 0.314336840254355 | 379 | 325 |
| C6orf89 | -0.0281736123402963 | 0.584530902479708 | 0.720641592636178 | 379 | 379 |
| C6orf94 | -0.0306122791086428 | 0.552426382232861 | 0.695700540636421 | 379 | 104 |
| C6orf97 | -0.109468232416566 | 0.0331315082524332 | 0.0916733945896239 | 379 | 379 |
| C6 | 0.0496100184658204 | 0.335443663657214 | 0.496467639247237 | 379 | 324 |
| C7orf10 | -0.0317493678148866 | 0.537757299243466 | 0.683328103652875 | 379 | 379 |
| C7orf11 | -0.112233370267444 | 0.0289152631418378 | 0.082458196113384 | 379 | 379 |
| C7orf13 | -0.150447609655324 | 0.0033252158830826 | 0.0156200854133527 | 379 | 379 |
| C7orf16 | 0.106607765936142 | 0.0380336938440698 | 0.101772210734172 | 379 | 71 |
| C7orf23 | -0.115363787893016 | 0.0247053920247493 | 0.0731459628291368 | 379 | 379 |
| C7orf25 | -0.0901198855466784 | 0.0797362178372289 | 0.17714394703379 | 379 | 379 |
| C7orf26 | 0.0597236511692348 | 0.246093417108972 | 0.399602020672892 | 379 | 379 |
| C7orf27 | -0.0262257555782258 | 0.610778821904851 | 0.739709411234388 | 379 | 379 |
| C7orf28A | 0.0401922068798621 | 0.43527936078053 | 0.592077873743318 | 379 | 379 |
| C7orf28B | -0.0287824531631136 | 0.576435131977777 | 0.71363360369976 | 379 | 379 |
| C7orf29 | -0.131268106218366 | 0.0105229349483011 | 0.0379695439059324 | 379 | 379 |
| C7orf30 | 0.109576450416965 | 0.0329571470203065 | 0.0912928626877112 | 379 | 379 |
| C7orf31 | -0.118369642113259 | 0.0211717317789464 | 0.0649433322962098 | 379 | 379 |
| C7orf33 | 0.16992176334023 | 0.000896051680107616 | 0.0056397137525056 | 379 | 48 |
| C7orf34 | 0.10748579846337 | 0.0364674710578959 | 0.0985252781218094 | 379 | 63 |
| C7orf36 | -0.186026600458209 | 0.000271134659773816 | 0.00219699960522895 | 379 | 379 |
| C7orf40 | -0.00848453085064156 | 0.869232425494481 | 0.923443020397802 | 379 | 379 |
| C7orf41 | -0.170174504836995 | 0.000880088379700938 | 0.00555467748933856 | 379 | 379 |
| C7orf42 | -0.113915642312156 | 0.0265822018051658 | 0.0775272833920306 | 379 | 379 |
| C7orf43 | 0.00444799590162092 | 0.931221870457075 | 0.960629896853592 | 379 | 379 |
| C7orf44 | 0.181049930300194 | 0.000396592330492904 | 0.00293638264712969 | 379 | 379 |
| C7orf45 | 0.00511859388614745 | 0.920884291869763 | 0.954924547393454 | 379 | 55 |
| C7orf46 | 0.00888544880968946 | 0.863112660309121 | 0.919651664711121 | 379 | 379 |
| C7orf47 | -0.0365587874653586 | 0.477946052740301 | 0.629975027171089 | 379 | 379 |
| C7orf49 | 0.106499517914273 | 0.0382306737801936 | 0.102161428532841 | 379 | 379 |
| C7orf4 | -0.182175152966666 | 0.000364229158337926 | 0.00274077258122368 | 379 | 296 |
| C7orf50 | -0.0259846348041191 | 0.614063894706644 | 0.742832666732269 | 379 | 379 |
| C7orf51 | 0.0682582158237548 | 0.184838634045493 | 0.325804999186953 | 379 | 376 |
| C7orf52 | -0.108170492998034 | 0.0352844248916244 | 0.0960883912582239 | 379 | 306 |
| C7orf53 | -0.0507575550344946 | 0.32437092063005 | 0.484840288319044 | 379 | 379 |
| C7orf54 | 0.0626301670110864 | 0.223814970483847 | 0.372768016358985 | 379 | 290 |
| C7orf55 | -0.0130465789442875 | 0.800143346732622 | 0.877798476444669 | 379 | 379 |
| C7orf57 | 0.138591789610633 | 0.00688752323667087 | 0.0275686480517125 | 379 | 271 |
| C7orf58 | -0.00686573769167881 | 0.894018040784148 | 0.938858625743769 | 379 | 379 |
| C7orf59 | -0.0460669094439935 | 0.371140258630983 | 0.531602185085251 | 379 | 379 |
| C7orf60 | -0.117942585066576 | 0.0216453924610237 | 0.0660793522914031 | 379 | 379 |
| C7orf61 | 0.228428448996708 | 7.06022994830372E-06 | 0.000133197183077989 | 379 | 368 |
| C7orf63 | 0.0124788878614535 | 0.808667823429277 | 0.884044456273426 | 379 | 379 |
| C7orf64 | -0.0918381047479986 | 0.0741370086211116 | 0.167596466416532 | 379 | 379 |
| C7orf65 | 0.0735044194224627 | 0.153238869172407 | 0.285646356862883 | 379 | 83 |
| C7orf68 | -0.165316432672637 | 0.00123795145970468 | 0.0072364686152784 | 379 | 379 |
| C7orf69 | 0.0253635229953075 | 0.622561517555463 | 0.749174593074572 | 379 | 254 |
| C7orf70 | 0.0080992446862939 | 0.875120946324865 | 0.927165275112446 | 379 | 379 |
| C7orf71 | 0.0963713680419999 | 0.0608866382205258 | 0.144599384673205 | 379 | 120 |
| C7orf72 | 0.0780897689751227 | 0.129129881263071 | 0.25200662260671 | 379 | 18 |
| C7 | -0.00971068200887196 | 0.850542098488785 | 0.911350917526918 | 379 | 377 |
| C8A | 0.0290162395618192 | 0.57334052813891 | 0.711502038793837 | 379 | 44 |
| C8B | 0.0384733960129654 | 0.455184564505568 | 0.609535999528358 | 379 | 46 |
| C8G | 0.119967391414855 | 0.0194784975963992 | 0.0610623953108939 | 379 | 377 |
| C8ORFK29 | -0.134955066850077 | 0.00852189314995237 | 0.0323577359971765 | 379 | 374 |
| C8orf12 | 0.00238267469334119 | 0.963124881881517 | 0.979900544371555 | 379 | 25 |
| C8orf22 | 0.0131958429335689 | 0.797905878332497 | 0.87654146756432 | 379 | 10 |
| C8orf30A | 0.0428341527750215 | 0.405680402525943 | 0.564558606210303 | 379 | 379 |
| C8orf31 | 0.161318568575646 | 0.00162809999618629 | 0.008932475573985 | 379 | 358 |
| C8orf33 | -0.130032009536007 | 0.011281451816129 | 0.0400086973010563 | 379 | 379 |
| C8orf34 | 0.0412288265514588 | 0.423520387798116 | 0.581117700340489 | 379 | 332 |
| C8orf37 | 0.0190664690284029 | 0.711388384682652 | 0.81487053099293 | 379 | 379 |
| C8orf38 | -0.10719432532864 | 0.0369812145857755 | 0.0995605597836736 | 379 | 379 |
| C8orf39 | -0.0554580288752374 | 0.281518112505266 | 0.438728376542829 | 379 | 379 |
| C8orf40 | -0.0775450865889304 | 0.131829886938422 | 0.255840559676522 | 379 | 379 |
| C8orf41 | 0.157111251246032 | 0.00215786036483098 | 0.011084470288567 | 379 | 379 |
| C8orf42 | -0.119432004046333 | 0.0200322642886511 | 0.0623449593965427 | 379 | 379 |
| C8orf44 | -0.205452763286018 | 0.0000558730373670033 | 0.000645977017441949 | 379 | 379 |
| C8orf45 | -0.172227137203283 | 0.000759844304227346 | 0.00496414921391098 | 379 | 379 |
| C8orf46 | 0.0833742292782349 | 0.105106842979973 | 0.216638095905083 | 379 | 290 |
| C8orf47 | -0.322412980659717 | 1.28917497310153E-10 | 2.63523313058322E-08 | 379 | 367 |
| C8orf48 | 0.0486457872689359 | 0.3449328676512 | 0.506555659355227 | 379 | 370 |
| C8orf4 | -0.104249800451414 | 0.0425242348413333 | 0.110579741433961 | 379 | 379 |
| C8orf51 | -0.145560872522759 | 0.00451778145879702 | 0.0197926769312664 | 379 | 379 |
| C8orf55 | 0.0386797392216734 | 0.452768313573126 | 0.607572422951269 | 379 | 379 |
| C8orf56 | -0.0235173175095179 | 0.648113771549645 | 0.768774861742323 | 379 | 144 |
| C8orf58 | 0.169715847922351 | 0.00090925456893283 | 0.00569831558546846 | 379 | 379 |
| C8orf59 | -0.140857700474063 | 0.00601694461676272 | 0.0248188013025112 | 379 | 379 |
| C8orf73 | 0.147009114253638 | 0.00412935990349129 | 0.0185116319616607 | 379 | 379 |
| C8orf74 | 0.0646942552756467 | 0.208891535769683 | 0.355345004396129 | 379 | 89 |
| C8orf75 | 0.0690589817087283 | 0.179726976560946 | 0.31942359488729 | 379 | 320 |
| C8orf76 | -0.0193804183576531 | 0.706853310675942 | 0.811044816223157 | 379 | 379 |
| C8orf77 | -0.172007150657239 | 0.000771962975991426 | 0.00502181164303084 | 379 | 379 |
| C8orf79 | -0.230469768495691 | 5.81198292456733E-06 | 0.000113645657778595 | 379 | 379 |
| C8orf80 | 0.116674664522976 | 0.0231061990606999 | 0.0694376651978719 | 379 | 362 |
| C8orf83 | -0.126879584153473 | 0.0134389350209457 | 0.0458241106784715 | 379 | 379 |
| C8orf84 | -0.172422321566694 | 0.000749239494950815 | 0.00490730946301639 | 379 | 379 |
| C8orf85 | 0.00342320886440745 | 0.947041197390476 | 0.970134989763296 | 379 | 335 |
| C8orf86 | 0.129973118752072 | 0.0113187667783273 | 0.0401195044119904 | 379 | 21 |
| C9orf100 | 0.0828415493348974 | 0.107355425165314 | 0.220105818444612 | 379 | 379 |
| C9orf102 | -0.0140385294028894 | 0.785305503771446 | 0.86838645523285 | 379 | 379 |
| C9orf103 | -0.0962882759464622 | 0.0611106281440119 | 0.144992405748411 | 379 | 379 |
| C9orf106 | 0.111287066090669 | 0.0303032312182074 | 0.085603714004077 | 379 | 89 |
| C9orf109 | 0.050142188601859 | 0.330278871654353 | 0.491022678800519 | 379 | 377 |
| C9orf110 | 0.0324530429684408 | 0.528778116398546 | 0.675337358579734 | 379 | 379 |
| C9orf114 | 0.0169032546874087 | 0.742905834824654 | 0.837474381312368 | 379 | 379 |
| C9orf116 | 0.190251461897462 | 0.000194812592350162 | 0.00168311288937648 | 379 | 379 |
| C9orf117 | -0.0927130609083824 | 0.0714112262413628 | 0.162864250507677 | 379 | 379 |
| C9orf119 | 0.0844627471330275 | 0.100627793868363 | 0.210548595847814 | 379 | 379 |
| C9orf11 | -0.0234672961056913 | 0.648812045438269 | 0.769140573774363 | 379 | 246 |
| C9orf122 | -0.161649478910873 | 0.00159196740797245 | 0.00877513876076294 | 379 | 379 |
| C9orf123 | -0.196341967759333 | 0.00011944796819612 | 0.00114526804322663 | 379 | 379 |
| C9orf125 | -0.0333442690180382 | 0.517515844890565 | 0.665670072818042 | 379 | 379 |
| C9orf128 | -0.292329515894709 | 6.65465212547373E-09 | 6.44924245879138E-07 | 379 | 331 |
| C9orf129 | -0.0490886711705378 | 0.340553372062909 | 0.501894771908976 | 379 | 379 |
| C9orf130 | -0.232661917938662 | 4.70678014858841E-06 | 0.0000970125122517786 | 379 | 379 |
| C9orf131 | 0.0564872047334696 | 0.272671079811991 | 0.428848742536972 | 379 | 288 |
| C9orf135 | -0.0445071930078982 | 0.387572107190415 | 0.546998344463774 | 379 | 51 |
| C9orf139 | 0.157199432148389 | 0.00214530589597598 | 0.0110371368202937 | 379 | 351 |
| C9orf140 | 0.0789430575944577 | 0.124986372572004 | 0.245807359190408 | 379 | 379 |
| C9orf142 | 0.0490581984261751 | 0.340853562877126 | 0.502150564286177 | 379 | 379 |
| C9orf144B | 0.0721212379127341 | 0.1611428722726 | 0.296077049268179 | 379 | 129 |
| C9orf144 | 0.0560205162906757 | 0.276659085963479 | 0.433006591121112 | 379 | 20 |
| C9orf150 | -0.168372464943843 | 0.000999892472330138 | 0.00614946276096836 | 379 | 379 |
| C9orf152 | -0.1306442991281 | 0.0108998859580827 | 0.0389691559280317 | 379 | 379 |
| C9orf153 | 0.00658718369189457 | 0.898294134441292 | 0.941567552827765 | 379 | 248 |
| C9orf156 | -0.0495159298197293 | 0.336362168575001 | 0.497381540644725 | 379 | 379 |
| C9orf163 | 0.0567183805321424 | 0.2707101862051 | 0.426477162885326 | 379 | 379 |
| C9orf167 | 0.0408605333124639 | 0.427676791946033 | 0.584866227374712 | 379 | 379 |
| C9orf169 | 0.158493980734246 | 0.00196851654648392 | 0.0103231277661156 | 379 | 375 |
| C9orf16 | -0.108187351606893 | 0.0352557122690632 | 0.0960411027747606 | 379 | 379 |
| C9orf170 | 0.0118313883131117 | 0.818418337973917 | 0.890647574387861 | 379 | 150 |
| C9orf171 | 0.0581466523105354 | 0.258808847314755 | 0.412944542090365 | 379 | 109 |
| C9orf172 | -0.072623705561159 | 0.158236853043544 | 0.291998168650293 | 379 | 379 |
| C9orf173 | -0.0534941461636887 | 0.298933213525366 | 0.457773228126425 | 379 | 326 |
| C9orf21 | 0.105078667902725 | 0.0408973190675119 | 0.10744826321328 | 379 | 379 |
| C9orf23 | -0.0401572068769234 | 0.435679633230416 | 0.592297275810263 | 379 | 379 |
| C9orf24 | 0.0213938362513678 | 0.678020888010605 | 0.790160936139313 | 379 | 376 |
| C9orf25 | 0.0447963730663427 | 0.384492645545723 | 0.544395899448772 | 379 | 379 |
| C9orf30 | 0.054370644138707 | 0.291074116556208 | 0.449522360236505 | 379 | 379 |
| C9orf37 | 0.0214191210890212 | 0.67766166843725 | 0.789990244200125 | 379 | 379 |
| C9orf3 | -0.0869424116547843 | 0.0909898827969116 | 0.195932601661291 | 379 | 379 |
| C9orf40 | -0.0444385901378796 | 0.388304846949148 | 0.547915492834309 | 379 | 379 |
| C9orf41 | -0.0162395578800626 | 0.752665278076361 | 0.844777942584517 | 379 | 379 |
| C9orf43 | -0.11839012094497 | 0.0211492458887819 | 0.0649144345948555 | 379 | 378 |
| C9orf44 | -0.00518444226562773 | 0.919869897638889 | 0.954481151833372 | 379 | 223 |
| C9orf45 | -0.0701873474181662 | 0.172702785515793 | 0.310655069509856 | 379 | 379 |
| C9orf46 | -0.114255899757761 | 0.0261305376636704 | 0.0764685767241501 | 379 | 379 |
| C9orf47 | 0.107643885192764 | 0.0361913736492961 | 0.097952846944887 | 379 | 346 |
| C9orf4 | -0.0527296822190615 | 0.305901899289474 | 0.465175462774115 | 379 | 100 |
| C9orf50 | -0.0113096270669543 | 0.826295875352219 | 0.895264675809092 | 379 | 243 |
| C9orf53 | 0.265577610631738 | 1.54011587815132E-07 | 6.98796742150672E-06 | 379 | 100 |
| C9orf57 | -0.029684024335885 | 0.564544623338007 | 0.704943056335159 | 379 | 219 |
| C9orf5 | -0.0633380123174903 | 0.218613968493188 | 0.366506955887624 | 379 | 379 |
| C9orf64 | 0.123738279841 | 0.0159420467324726 | 0.0523255922217293 | 379 | 379 |
| C9orf66 | 0.071617195126529 | 0.164098159540761 | 0.299745583360129 | 379 | 364 |
| C9orf68 | -0.131684028467302 | 0.010278065554907 | 0.0373384909898674 | 379 | 379 |
| C9orf69 | -0.045348815376649 | 0.378651308398058 | 0.538841567899402 | 379 | 379 |
| C9orf6 | -0.0732321063429362 | 0.154771285936308 | 0.287610595833657 | 379 | 379 |
| C9orf70 | -0.0751196748872496 | 0.144384526353372 | 0.273220798355879 | 379 | 249 |
| C9orf71 | -0.233719707585967 | 4.24818018535922E-06 | 0.000089594733017521 | 379 | 303 |
| C9orf72 | -0.119677034908867 | 0.0197771457035258 | 0.0617448031820988 | 379 | 379 |
| C9orf78 | -0.0403387153272874 | 0.433606134013789 | 0.590328373058597 | 379 | 379 |
| C9orf79 | -0.0277048167144177 | 0.590800158591546 | 0.725120355512756 | 379 | 79 |
| C9orf7 | -0.21710155382444 | 0.0000201237517103705 | 0.000295925432454133 | 379 | 379 |
| C9orf80 | -0.0446215659274338 | 0.386352370660287 | 0.54577432586823 | 379 | 379 |
| C9orf82 | -0.175379647247946 | 0.000604431719641163 | 0.00411702924666608 | 379 | 379 |
| C9orf84 | 0.0262312506799015 | 0.610704046193397 | 0.739664029559751 | 379 | 235 |
| C9orf85 | -0.08259603792879 | 0.108404489437449 | 0.221706824270521 | 379 | 379 |
| C9orf86 | -0.0116157074868871 | 0.821672516058618 | 0.892854176313355 | 379 | 379 |
| C9orf89 | 0.0529635886252562 | 0.303758361638558 | 0.462767636924267 | 379 | 379 |
| C9orf91 | 0.0125112847765379 | 0.808180730380954 | 0.883917927175758 | 379 | 379 |
| C9orf93 | -0.135501201712612 | 0.00825627013039678 | 0.0316094466394106 | 379 | 379 |
| C9orf95 | -0.0835351840017205 | 0.104434785334659 | 0.215785080273434 | 379 | 379 |
| C9orf96 | -0.0587042886366226 | 0.254261753179258 | 0.408516493156011 | 379 | 372 |
| C9orf98 | -0.149101841705882 | 0.00362129452538081 | 0.0167312611669622 | 379 | 378 |
| C9orf9 | -0.0143357966342284 | 0.780873729191694 | 0.865604347066359 | 379 | 379 |
| C9 | -0.0617772135346451 | 0.230198506477445 | 0.380523216876596 | 379 | 221 |
| CA10 | -0.0671848236380895 | 0.191857585170944 | 0.334931519525398 | 379 | 251 |
| CA11 | 0.149096486878149 | 0.0036225188717858 | 0.0167312611669622 | 379 | 379 |
| CA12 | -0.0971448825977335 | 0.0588338231106267 | 0.140978269721863 | 379 | 378 |
| CA13 | 0.000686475392758706 | 0.989372406700328 | 0.994224794775857 | 379 | 379 |
| CA14 | 0.152071358100196 | 0.00299730018321412 | 0.0144143749776303 | 379 | 370 |
| CA1 | -0.00132900853268754 | 0.979426765696479 | 0.988701451493218 | 379 | 292 |
| CA2 | 0.00219903464814752 | 0.965965171050518 | 0.981525199668057 | 379 | 379 |
| CA3 | -0.0448967653065385 | 0.383427069848816 | 0.543347059124452 | 379 | 379 |
| CA4 | 0.0154143810512932 | 0.764854200885669 | 0.854107293036778 | 379 | 336 |
| CA5A | 0.0544026253067477 | 0.290789999562423 | 0.449258540698436 | 379 | 45 |
| CA5BP | 0.0878479984868509 | 0.0876600815271285 | 0.190526205120464 | 379 | 379 |
| CA5B | -0.0112227011907821 | 0.827609996688901 | 0.895978761362137 | 379 | 379 |
| CA6 | 0.00257049542887656 | 0.960220413923112 | 0.978579891409717 | 379 | 134 |
| CA7 | 0.0470382448347478 | 0.361128085140964 | 0.522355388982713 | 379 | 367 |
| CA8 | 0.0203746507736324 | 0.692560285093969 | 0.800891480977675 | 379 | 375 |
| CA9 | 0.0862123979780715 | 0.0937472153352024 | 0.200217555543558 | 379 | 379 |
| CAB39L | -0.234901992954577 | 3.78612123259167E-06 | 0.0000822639512598465 | 379 | 379 |
| CAB39 | 0.0574783873219189 | 0.264331569384165 | 0.41925976783851 | 379 | 379 |
| CABC1 | -0.309673281934031 | 7.23435341350421E-10 | 1.12064655846064E-07 | 379 | 379 |
| CABIN1 | 0.199427654368126 | 0.0000926969009443386 | 0.000949377144589021 | 379 | 379 |
| CABLES1 | 0.212761816046516 | 0.00002963395485277 | 0.000395571547203772 | 379 | 379 |
| CABLES2 | -0.193484352478398 | 0.000150543138268716 | 0.00137515195376461 | 379 | 379 |
| CABP1 | 0.243154427049752 | 1.66651908346975E-06 | 0.0000440583205160508 | 379 | 375 |
| CABP2 | 0.0733781211001144 | 0.15394816606718 | 0.286602171555067 | 379 | 9 |
| CABP4 | -0.0464373228553835 | 0.367302104568641 | 0.528132544609093 | 379 | 368 |
| CABP5 | 0.0519257343854322 | 0.3133453371465 | 0.47308393702435 | 379 | 14 |
| CABP7 | 0.0513932755495865 | 0.318339987718817 | 0.478584068275738 | 379 | 378 |
| CABYR | 0.10168194712076 | 0.0479143570389387 | 0.12111191985954 | 379 | 379 |
| CACHD1 | -0.113233225368056 | 0.0275083295265102 | 0.0795674920279568 | 379 | 379 |
| CACNA1A | 0.15235838711009 | 0.00294248937594827 | 0.0141991677400318 | 379 | 373 |
| CACNA1B | 0.0138373940237333 | 0.788308053678856 | 0.870202209572673 | 379 | 298 |
| CACNA1C | -0.141329708959908 | 0.00584853648309799 | 0.0243112749238715 | 379 | 379 |
| CACNA1D | -0.390301630991512 | 3.0630812442216E-15 | 8.67639641577512E-12 | 379 | 379 |
| CACNA1E | 0.146954437601016 | 0.00414345781929813 | 0.0185437398491532 | 379 | 366 |
| CACNA1F | 0.0283758924223725 | 0.581835340631623 | 0.718256203090762 | 379 | 367 |
| CACNA1G | -0.0146496345107993 | 0.776202596737689 | 0.862070525296303 | 379 | 349 |
| CACNA1H | 0.0724648963927145 | 0.159151021153176 | 0.293360732051845 | 379 | 379 |
| CACNA1I | 0.0757460399078399 | 0.141058063821594 | 0.268647083891572 | 379 | 361 |
| CACNA1S | -0.0474224347993579 | 0.357215003557713 | 0.518245342104509 | 379 | 191 |
| CACNA2D1 | 0.0617822065632616 | 0.230160767092821 | 0.380492553769924 | 379 | 361 |
| CACNA2D2 | -0.140341539733682 | 0.00620607572938409 | 0.0254506865692302 | 379 | 379 |
| CACNA2D3 | 0.0560161486859567 | 0.276696594654942 | 0.433006591121112 | 379 | 353 |
| CACNA2D4 | 0.00681460242277013 | 0.894802794227342 | 0.939384222160202 | 379 | 379 |
| CACNB1 | -0.0531924715393481 | 0.301670504037196 | 0.460728834234984 | 379 | 379 |
| CACNB2 | -0.0667579990882151 | 0.194702197658464 | 0.33825945633681 | 379 | 379 |
| CACNB3 | -0.0214363292670588 | 0.677417235023017 | 0.789921720538484 | 379 | 379 |
| CACNB4 | -0.106240460167699 | 0.0387055939377776 | 0.10313862607153 | 379 | 377 |
| CACNG1 | 0.0468746249206543 | 0.362802698707835 | 0.524089458690002 | 379 | 117 |
| CACNG2 | 0.0549931725492692 | 0.285577036140842 | 0.443415933641395 | 379 | 11 |
| CACNG3 | 0.0837221557554719 | 0.10365836770438 | 0.214723998625413 | 379 | 26 |
| CACNG4 | -0.0320413463204706 | 0.534022295703874 | 0.679932837553227 | 379 | 356 |
| CACNG5 | 0.113752760310142 | 0.0268007811529405 | 0.0780505378441657 | 379 | 22 |
| CACNG6 | 0.169804576678738 | 0.000903543592325283 | 0.0056769681742287 | 379 | 151 |
| CACNG7 | 0.145801840020255 | 0.00445094961860043 | 0.0195727276641405 | 379 | 129 |
| CACNG8 | 0.113683311324711 | 0.0268944471765707 | 0.0782828976243458 | 379 | 157 |
| CACYBP | 0.0013693906049914 | 0.978801790037039 | 0.988292336739594 | 379 | 379 |
| CADM1 | 0.0584537025121741 | 0.256298193450634 | 0.410557487456711 | 379 | 379 |
| CADM2 | 0.00183519990842102 | 0.971593697218622 | 0.985157751390991 | 379 | 184 |
| CADM3 | 0.0858872211124752 | 0.0949967392434626 | 0.202269338968039 | 379 | 355 |
| CADM4 | -0.0852548415895551 | 0.0974647488492551 | 0.205960890992543 | 379 | 379 |
| CADPS2 | 0.043761194959323 | 0.395585063072003 | 0.555301227458091 | 379 | 379 |
| CADPS | -0.126700316699017 | 0.0135718709088502 | 0.0461662474490792 | 379 | 378 |
| CAD | 0.0925121100306862 | 0.0720299066935823 | 0.164011138024845 | 379 | 379 |
| CAGE1 | 0.0839914685273391 | 0.102548060145619 | 0.213203621324036 | 379 | 320 |
| CALB1 | 0.268927571301423 | 1.05827045913912E-07 | 5.26188799381533E-06 | 379 | 321 |
| CALB2 | 0.265726872275721 | 1.51474668985764E-07 | 0.0000069203680567966 | 379 | 367 |
| CALCA | -0.128858365705215 | 0.0120459913836017 | 0.0421909657615536 | 379 | 301 |
| CALCB | 0.0526419649938049 | 0.306708316281596 | 0.466187236123533 | 379 | 213 |
| CALCOCO1 | -0.159354259780137 | 0.0018585107592401 | 0.00989011039565558 | 379 | 379 |
| CALCOCO2 | 0.173312469266781 | 0.000702589340850816 | 0.00465606331898061 | 379 | 379 |
| CALCRL | 0.0765977084796714 | 0.136629606049592 | 0.262407189921669 | 379 | 379 |
| CALCR | 0.00999504879771319 | 0.846218995875668 | 0.908783524405901 | 379 | 290 |
| CALD1 | 0.0938453717985105 | 0.0680057124324669 | 0.156956962648231 | 379 | 379 |
| CALHM1 | 0.0126924777592179 | 0.80545783372172 | 0.881818669705387 | 379 | 252 |
| CALHM2 | 0.125606338438642 | 0.0144084667310827 | 0.0484961939134117 | 379 | 379 |
| CALHM3 | 0.0288865322756559 | 0.575056479665937 | 0.712680309226759 | 379 | 265 |
| CALM1 | 0.0290922038787787 | 0.5723366888552 | 0.710823167342368 | 379 | 379 |
| CALM2 | 0.0421927102083326 | 0.412754485356582 | 0.571076403297069 | 379 | 379 |
| CALM3 | 0.313964501957098 | 4.08350582069689E-10 | 7.04067420980677E-08 | 379 | 379 |
| CALML3 | -0.199744456353444 | 0.000090295270443881 | 0.000930064738888972 | 379 | 355 |
| CALML4 | -0.0268151057123225 | 0.602782290211196 | 0.734330747745613 | 379 | 379 |
| CALML5 | -0.00623894361351762 | 0.903644107611599 | 0.944565099147176 | 379 | 110 |
| CALML6 | -0.0452421837067508 | 0.379774537597773 | 0.539719719860138 | 379 | 263 |
| CALN1 | -0.144689361746203 | 0.00476711937652643 | 0.0206449861127603 | 379 | 205 |
| CALR3 | 0.0170083115373519 | 0.741364728461359 | 0.836402835614898 | 379 | 138 |
| CALR | 0.206471174607774 | 0.0000512154952405706 | 0.000602313665260993 | 379 | 379 |
| CALU | 0.14138642971585 | 0.0058285865651323 | 0.0242468735890897 | 379 | 379 |
| CALY | 0.0675051098917596 | 0.189743086461123 | 0.332197427086422 | 379 | 215 |
| CAMK1D | -0.180480736698642 | 0.000413964069039161 | 0.00303288507243772 | 379 | 379 |
| CAMK1G | 0.192250146331595 | 0.000166194705103699 | 0.00149514909836486 | 379 | 365 |
| CAMK1 | -0.0434931428159252 | 0.398488454839526 | 0.557878209599529 | 379 | 379 |
| CAMK2A | 0.122624665034972 | 0.0169227383921512 | 0.0546755836466634 | 379 | 364 |
| CAMK2B | 0.0672954778493647 | 0.191125113933204 | 0.334041117088007 | 379 | 348 |
| CAMK2D | 0.0899669932159802 | 0.0802506121154189 | 0.178067271376961 | 379 | 379 |
| CAMK2G | 0.00557137632749506 | 0.913911855790143 | 0.950637093516261 | 379 | 379 |
| CAMK2N1 | -0.100352155169921 | 0.0509233561399596 | 0.126625069669315 | 379 | 379 |
| CAMK2N2 | 0.242297847991495 | 1.81719139017891E-06 | 0.0000467166355177283 | 379 | 379 |
| CAMK4 | 0.0325325438445485 | 0.527768452818726 | 0.674395365243907 | 379 | 373 |
| CAMKK1 | 0.00453719905994214 | 0.929846057601779 | 0.960259772402503 | 379 | 379 |
| CAMKK2 | 0.0849960842698134 | 0.0984892123085721 | 0.207515754973768 | 379 | 379 |
| CAMKV | -0.067926035859378 | 0.186990248680382 | 0.328633456021504 | 379 | 347 |
| CAMLG | -0.219922192417545 | 0.0000155822316179251 | 0.000243835223980131 | 379 | 379 |
| CAMP | 0.0536059309914065 | 0.297923122907881 | 0.456755561819954 | 379 | 226 |
| CAMSAP1L1 | -0.00277579375570736 | 0.957046274568129 | 0.976750747999632 | 379 | 379 |
| CAMSAP1 | 0.0644474462608083 | 0.210637195212442 | 0.357242027872803 | 379 | 379 |
| CAMTA1 | -0.0285988970977732 | 0.578870328671973 | 0.715799243960579 | 379 | 379 |
| CAMTA2 | 0.0885739314864832 | 0.085062163218428 | 0.186407239536929 | 379 | 379 |
| CAND1 | 0.221013139379992 | 0.0000141019072410022 | 0.000226302935618461 | 379 | 379 |
| CAND2 | -0.00130816866960146 | 0.979749300081311 | 0.988842690796276 | 379 | 379 |
| CANT1 | 0.114306443296504 | 0.0260640120472284 | 0.076313826177266 | 379 | 379 |
| CANX | 0.0280389826577405 | 0.586328163360401 | 0.721780270882847 | 379 | 379 |
| CAP1 | 0.0787185868485718 | 0.126066244926942 | 0.247415768030427 | 379 | 379 |
| CAP2 | 0.143797208697673 | 0.00503514685636506 | 0.021566677819795 | 379 | 379 |
| CAPG | 0.0879618657111654 | 0.0872484163989532 | 0.189834478257264 | 379 | 379 |
| CAPN10 | -0.0851328143636268 | 0.0979468130309864 | 0.206693210810813 | 379 | 379 |
| CAPN11 | 0.142692614592112 | 0.0053857593170627 | 0.0227113644701657 | 379 | 358 |
| CAPN12 | -0.15616518300412 | 0.00229683808930876 | 0.0116624086132687 | 379 | 379 |
| CAPN13 | -0.240004818654673 | 2.28741054519678E-06 | 0.0000553782372285248 | 379 | 371 |
| CAPN14 | 0.156859477615264 | 0.00219407594139164 | 0.0112297722679178 | 379 | 333 |
| CAPN1 | 0.0431838865081586 | 0.401853980347567 | 0.561205897602762 | 379 | 379 |
| CAPN2 | 0.0924194062418559 | 0.0723167901003378 | 0.16453900112042 | 379 | 379 |
| CAPN3 | -0.273601078143204 | 6.21615803368207E-08 | 3.45249247876325E-06 | 379 | 379 |
| CAPN5 | -0.00242664619306048 | 0.962444859595949 | 0.979789324642834 | 379 | 379 |
| CAPN6 | -0.253037607848373 | 5.9973024153374E-07 | 0.0000198521723357779 | 379 | 373 |
| CAPN7 | -0.241032407315958 | 2.06385403936386E-06 | 0.0000513451667409117 | 379 | 379 |
| CAPN8 | 0.0153856902068287 | 0.765279066613387 | 0.854341159439797 | 379 | 379 |
| CAPN9 | -0.0233827383227381 | 0.649993129229592 | 0.7698761009807 | 379 | 374 |
| CAPNS1 | 0.0759278514167654 | 0.140103582215694 | 0.267267060628514 | 379 | 379 |
| CAPNS2 | 0.0781259854536994 | 0.12895188347633 | 0.251733577391815 | 379 | 256 |
| CAPRIN1 | -0.129769030597673 | 0.0114489290741891 | 0.0405156818995219 | 379 | 379 |
| CAPRIN2 | 0.076591605384069 | 0.136660955940274 | 0.26244197911707 | 379 | 379 |
| CAPS2 | -0.0903981927060748 | 0.0788067289863805 | 0.175610229528203 | 379 | 378 |
| CAPSL | -0.0407753152582001 | 0.428641886956667 | 0.585782020440885 | 379 | 117 |
| CAPS | -0.230511289742919 | 5.78892133360482E-06 | 0.000113645657778595 | 379 | 379 |
| CAPZA1 | 0.0867664444886313 | 0.0916485129797857 | 0.197094003835487 | 379 | 379 |
| CAPZA2 | -0.090314585748302 | 0.0790850318518141 | 0.17607208753175 | 379 | 379 |
| CAPZA3 | 0.0847348940615127 | 0.0995319752175791 | 0.208799421131899 | 379 | 5 |
| CAPZB | 0.278992440891678 | 3.32279794397935E-08 | 2.21088716890009E-06 | 379 | 379 |
| CARD10 | -0.00114473919939025 | 0.982278775413166 | 0.989867023729023 | 379 | 379 |
| CARD11 | -0.0590356978558671 | 0.251585749900704 | 0.405630366647517 | 379 | 379 |
| CARD14 | -0.0929329150306781 | 0.0707393232306871 | 0.161853138820455 | 379 | 379 |
| CARD16 | 0.161234764701117 | 0.00163736919092521 | 0.00895854203026077 | 379 | 379 |
| CARD17 | 0.17726524840634 | 0.000526138623506066 | 0.00367923524244609 | 379 | 128 |
| CARD18 | 0.0893335347864809 | 0.082410512533488 | 0.181886587036796 | 379 | 10 |
| CARD6 | 0.0060797763800166 | 0.906090842454574 | 0.94580423910567 | 379 | 379 |
| CARD8 | 0.0453322915619755 | 0.378825232312766 | 0.538950039915156 | 379 | 379 |
| CARD9 | 0.123631962653462 | 0.016033474809301 | 0.0525647715804927 | 379 | 379 |
| CARHSP1 | -0.0499310379672312 | 0.332321953111804 | 0.493038418280134 | 379 | 379 |
| CARKD | -0.158834033115208 | 0.00192434066889221 | 0.0101532269246394 | 379 | 379 |
| CARM1 | 0.0111218267111154 | 0.829135591747173 | 0.897091591900193 | 379 | 379 |
| CARNS1 | -0.0061374363411636 | 0.905204383900102 | 0.94521865972116 | 379 | 379 |
| CARS2 | -0.116082275006773 | 0.0238174397361284 | 0.0710153676823991 | 379 | 379 |
| CARS | 0.108735936223378 | 0.0343321735178988 | 0.0941095799588258 | 379 | 379 |
| CARTPT | -0.00429165805350306 | 0.933633617485384 | 0.961866744648248 | 379 | 226 |
| CASC1 | -0.0735447531434816 | 0.153012875555711 | 0.285412915947191 | 379 | 320 |
| CASC2 | -0.0895353591605729 | 0.0817173151587813 | 0.180663067340678 | 379 | 379 |
| CASC3 | -0.0853167879411565 | 0.0972207558286656 | 0.205554824756961 | 379 | 379 |
| CASC4 | -0.0142811014354937 | 0.781688618978649 | 0.865828268146429 | 379 | 379 |
| CASC5 | 0.0983495676024182 | 0.0557509561960471 | 0.135552416855331 | 379 | 379 |
| CASD1 | -0.0717737137524554 | 0.163176142930805 | 0.298721868897794 | 379 | 379 |
| CASKIN1 | 0.0751955182008987 | 0.143978584240285 | 0.272587354942842 | 379 | 341 |
| CASKIN2 | 0.0498633926648174 | 0.33297820136417 | 0.493807762533624 | 379 | 379 |
| CASK | -0.0914196438559106 | 0.0754702785886921 | 0.169797422427844 | 379 | 379 |
| CASP10 | 0.0911345043964512 | 0.0763898872917652 | 0.171205909937959 | 379 | 379 |
| CASP12 | -0.0283570415340789 | 0.582086300823354 | 0.718431819030529 | 379 | 258 |
| CASP14 | 0.000642564809729417 | 0.990052167279349 | 0.994667327361924 | 379 | 15 |
| CASP1 | 0.0925041993967261 | 0.0720543508468775 | 0.164047958272119 | 379 | 379 |
| CASP2 | 0.00431506310759262 | 0.933272520073497 | 0.961744583338564 | 379 | 379 |
| CASP3 | 0.0947071492559822 | 0.065503976820983 | 0.152585320693275 | 379 | 379 |
| CASP4 | 0.119318545105315 | 0.0201513601038782 | 0.0625879022775214 | 379 | 379 |
| CASP5 | 0.0446677729843036 | 0.385860255509641 | 0.545436454426831 | 379 | 379 |
| CASP6 | -0.163192333115018 | 0.00143300978292672 | 0.0080835994397006 | 379 | 379 |
| CASP7 | 0.01107436916497 | 0.829853547716895 | 0.897609520376935 | 379 | 379 |
| CASP8AP2 | -0.0133998886111094 | 0.794849908681922 | 0.874405458796335 | 379 | 379 |
| CASP8 | 0.06175267472358 | 0.230384044701714 | 0.380702961775613 | 379 | 379 |
| CASP9 | 0.148118007330616 | 0.00385263936398729 | 0.0175448170209325 | 379 | 379 |
| CASQ1 | 0.0112496263382016 | 0.827202898188522 | 0.895684745810507 | 379 | 325 |
| CASQ2 | 0.0356878554566517 | 0.488501512077742 | 0.639974098544926 | 379 | 344 |
| CASR | -0.0040400702304871 | 0.937516003795652 | 0.964313291656388 | 379 | 208 |
| CASS4 | 0.168768288793036 | 0.000972356252722166 | 0.00600806474882365 | 379 | 377 |
| CAST | -0.0692685461848276 | 0.178406671577605 | 0.317772860585766 | 379 | 379 |
| CASZ1 | 0.0378901438336593 | 0.462053328639951 | 0.615657106395601 | 379 | 379 |
| CATSPER1 | 0.161783296224633 | 0.0015775656364845 | 0.00871992265691665 | 379 | 349 |
| CATSPER2P1 | 0.0505185694214237 | 0.326657160906426 | 0.486952724340472 | 379 | 379 |
| CATSPER2 | -0.0510755967046264 | 0.321344530026612 | 0.481745809404187 | 379 | 379 |
| CATSPER3 | 0.0522115109355567 | 0.310685955317565 | 0.470142801040729 | 379 | 378 |
| CATSPER4 | 0.0854147288056073 | 0.0968359810362812 | 0.205113110991068 | 379 | 58 |
| CATSPERB | -0.08489826328385 | 0.0988787256239299 | 0.207977672990894 | 379 | 372 |
| CATSPERG | 0.15563978390409 | 0.00237750011253853 | 0.0119951837738967 | 379 | 372 |
| CAT | -0.314838672536488 | 3.63034664837213E-10 | 6.37013392424094E-08 | 379 | 379 |
| CAV1 | 0.152192919492367 | 0.00297397466044682 | 0.0143269200733411 | 379 | 379 |
| CAV2 | 0.163351119934534 | 0.00141750584638423 | 0.00803266816865005 | 379 | 379 |
| CAV3 | 0.161203582602041 | 0.00164083044117651 | 0.00897500303107524 | 379 | 72 |
| CBARA1 | 0.184591457265501 | 0.000302866000588767 | 0.0023754853875293 | 379 | 379 |
| CBFA2T2 | -0.304259717626409 | 1.46914874236483E-09 | 2.00898491473171E-07 | 379 | 379 |
| CBFA2T3 | -0.00228443846334879 | 0.964644207765548 | 0.980920321635739 | 379 | 379 |
| CBFB | -0.0384496448241441 | 0.455463151041884 | 0.609703170325309 | 379 | 379 |
| CBLB | -0.13720241877101 | 0.00747550942752445 | 0.029324045846224 | 379 | 379 |
| CBLC | -0.007285630842552 | 0.887578076942946 | 0.934719466200592 | 379 | 379 |
| CBLL1 | -0.0313717098726596 | 0.542607618601326 | 0.687333026360895 | 379 | 379 |
| CBLN1 | -0.106567228928052 | 0.0381073584953606 | 0.101900566992045 | 379 | 363 |
| CBLN2 | -0.150163586889057 | 0.00338580970469802 | 0.0158521451770371 | 379 | 348 |
| CBLN3 | 0.111895534543531 | 0.0294043871968737 | 0.0836125325311361 | 379 | 379 |
| CBLN4 | -0.0178880252186354 | 0.728501050487843 | 0.827303483910249 | 379 | 239 |
| CBL | 0.111416585069297 | 0.0301099574647371 | 0.0851790892582118 | 379 | 379 |
| CBR1 | 0.0270424608373042 | 0.59971004460746 | 0.731756970121645 | 379 | 379 |
| CBR3 | 0.201482771215365 | 0.0000781259575890764 | 0.000831944944724064 | 379 | 379 |
| CBR4 | 0.00979172913953921 | 0.849309512854266 | 0.910522250385206 | 379 | 379 |
| CBS | 0.00983171605464388 | 0.848701516496897 | 0.910271962180869 | 379 | 379 |
| CBWD1 | -0.0150565785536267 | 0.770157688444559 | 0.857586699956676 | 379 | 379 |
| CBWD2 | -0.125607041782852 | 0.0144079146277474 | 0.0484961939134117 | 379 | 379 |
| CBWD3 | -0.0352167229455944 | 0.494263254949728 | 0.645305315015686 | 379 | 379 |
| CBWD5 | -0.0271435409337717 | 0.598346422244914 | 0.730795451177404 | 379 | 250 |
| CBWD6 | -0.0462711323588354 | 0.369021082747525 | 0.529847847347132 | 379 | 379 |
| CBX1 | -0.0159798500926645 | 0.756495038272882 | 0.847828601564249 | 379 | 379 |
| CBX2 | 0.109240933728114 | 0.0335002943592276 | 0.0923844000771579 | 379 | 379 |
| CBX3 | -0.0710129451759741 | 0.167694360307761 | 0.304073504909217 | 379 | 379 |
| CBX4 | -0.0170497522333339 | 0.740757107748882 | 0.836002728239788 | 379 | 379 |
| CBX5 | 0.0153478618654629 | 0.765839351632951 | 0.854677923351052 | 379 | 379 |
| CBX6 | 0.105321034263199 | 0.040431643307746 | 0.106563687824802 | 379 | 379 |
| CBX7 | -0.0286940938651731 | 0.577606773916983 | 0.71472710392074 | 379 | 379 |
| CBX8 | 0.0232640160748839 | 0.651652893161971 | 0.770846613816947 | 379 | 379 |
| CBY1 | -0.0157973130387698 | 0.759190382046395 | 0.849873091156572 | 379 | 379 |
| CC2D1A | -0.249410864112582 | 0.0000008770200280162 | 0.0000264681173751981 | 379 | 379 |
| CC2D1B | 0.0221312534634505 | 0.667574700719571 | 0.782262937525421 | 379 | 379 |
| CC2D2A | -0.145220606873222 | 0.00461369570916219 | 0.0201099930800765 | 379 | 379 |
| CC2D2B | -0.0137074045072008 | 0.790250208735072 | 0.871614422335908 | 379 | 355 |
| CCAR1 | 0.0047118116390179 | 0.9271535522465 | 0.95869207965975 | 379 | 379 |
| CCBE1 | 0.127757124867306 | 0.0128045965038078 | 0.044239334287768 | 379 | 351 |
| CCBL1 | 0.14299605740532 | 0.00528731853065569 | 0.022406252045469 | 379 | 379 |
| CCBL2 | -0.0959230707467248 | 0.0621031797856672 | 0.146673664691571 | 379 | 379 |
| CCBP2 | 0.114197774780235 | 0.0262072226734341 | 0.0766312949666496 | 379 | 379 |
| CCDC101 | -0.0859903881884465 | 0.094598878485638 | 0.201623837752685 | 379 | 379 |
| CCDC102A | 0.0398797595357021 | 0.438860092115057 | 0.595437108694222 | 379 | 379 |
| CCDC102B | 0.166217563032198 | 0.00116283242822084 | 0.00691608619032431 | 379 | 379 |
| CCDC103 | 0.164558825678974 | 0.0013045327528118 | 0.00751489698511108 | 379 | 379 |
| CCDC104 | 0.0386549926293836 | 0.453057711496981 | 0.607837357301722 | 379 | 379 |
| CCDC105 | -0.0331510187308651 | 0.519947374265577 | 0.667628321262651 | 379 | 4 |
| CCDC106 | -0.184257379581355 | 0.000310733713445579 | 0.00242186637979518 | 379 | 379 |
| CCDC107 | 0.0000191542899057532 | 0.999703456240185 | 0.999776050024175 | 379 | 379 |
| CCDC108 | -0.0591441663843972 | 0.250714173207098 | 0.404751720106688 | 379 | 376 |
| CCDC109A | 0.17019592831299 | 0.000878747405267602 | 0.00554961939211089 | 379 | 379 |
| CCDC109B | 0.217356013271584 | 0.0000196674276708501 | 0.000291058990088346 | 379 | 379 |
| CCDC110 | 0.000451930960882675 | 0.993003363772561 | 0.996118116810804 | 379 | 376 |
| CCDC111 | 0.0425753624422927 | 0.408525722318802 | 0.567403195722695 | 379 | 379 |
| CCDC112 | -0.0473972634404883 | 0.357470563750854 | 0.518576351581642 | 379 | 379 |
| CCDC113 | -0.196775677677298 | 0.000115293008546016 | 0.00111513647485385 | 379 | 379 |
| CCDC114 | 0.0258551633464386 | 0.615831031375353 | 0.744055675468314 | 379 | 379 |
| CCDC115 | -0.184874511056924 | 0.000296345746331231 | 0.00234205557576688 | 379 | 379 |
| CCDC116 | 0.0249996546169495 | 0.627563176601654 | 0.75316869671011 | 379 | 364 |
| CCDC117 | 0.045291068839947 | 0.379259341749357 | 0.539296774828332 | 379 | 379 |
| CCDC11 | 0.0771944441569841 | 0.133590952918816 | 0.258322743756026 | 379 | 376 |
| CCDC120 | -0.082892032342732 | 0.107140708522561 | 0.219801962605829 | 379 | 379 |
| CCDC121 | -0.230892342319696 | 5.58131346813915E-06 | 0.000110225381918589 | 379 | 379 |
| CCDC122 | -0.236419659661854 | 3.26299384687759E-06 | 0.0000733544693830939 | 379 | 378 |
| CCDC123 | -0.00544191766919731 | 0.915904751046917 | 0.951861177407531 | 379 | 379 |
| CCDC124 | 0.182590584840053 | 0.000352915377997857 | 0.00267635914741261 | 379 | 379 |
| CCDC125 | -0.214748187541502 | 0.0000248473219177015 | 0.000343565340993156 | 379 | 379 |
| CCDC126 | 0.0171212063893711 | 0.739709795582529 | 0.835201060691896 | 379 | 379 |
| CCDC127 | 0.0951369292970514 | 0.0642848619694354 | 0.150524355589273 | 379 | 379 |
| CCDC129 | 0.0412715900353264 | 0.423039307248526 | 0.580646780016875 | 379 | 298 |
| CCDC12 | -0.119969289903431 | 0.0194765578779731 | 0.0610623953108939 | 379 | 379 |
| CCDC130 | -0.108910031941367 | 0.034043422547386 | 0.0935050536458747 | 379 | 379 |
| CCDC132 | -0.0335177801842465 | 0.515337683635508 | 0.663855431219383 | 379 | 379 |
| CCDC134 | 0.215798501221886 | 0.0000226222353136821 | 0.000320166796430898 | 379 | 379 |
| CCDC135 | 0.0854282430916989 | 0.0967829835871753 | 0.205066573901102 | 379 | 193 |
| CCDC136 | -0.0405339812351726 | 0.431381832326737 | 0.588350458892181 | 379 | 379 |
| CCDC137 | 0.163141310155274 | 0.00143802461768225 | 0.00810493238186574 | 379 | 379 |
| CCDC138 | 0.0156458841198777 | 0.761428597750401 | 0.851576864808785 | 379 | 378 |
| CCDC13 | 0.127834686124544 | 0.0127498209478384 | 0.0441038816737158 | 379 | 376 |
| CCDC140 | -0.032994967251022 | 0.521915131674502 | 0.669504640670378 | 379 | 27 |
| CCDC141 | 0.0391403230114042 | 0.447401051872327 | 0.602654682775382 | 379 | 355 |
| CCDC142 | -0.0639225292750806 | 0.214384787861841 | 0.361495158918665 | 379 | 379 |
| CCDC144A | -0.0658889229817875 | 0.200589256868738 | 0.345610339345963 | 379 | 316 |
| CCDC144B | -0.0644215811983534 | 0.21082074335743 | 0.357389590380297 | 379 | 353 |
| CCDC144C | -0.0228477685308126 | 0.657485675581637 | 0.774650836768544 | 379 | 208 |
| CCDC144NL | 0.0825584781874541 | 0.108565691490545 | 0.221921704213869 | 379 | 94 |
| CCDC146 | -0.0968030672298513 | 0.0597337913729213 | 0.142623196756489 | 379 | 379 |
| CCDC147 | 0.167733962305147 | 0.0010458304277595 | 0.0063844598896599 | 379 | 345 |
| CCDC148 | -0.0110403511379432 | 0.830368272603059 | 0.897886355956452 | 379 | 368 |
| CCDC149 | -0.147080999086157 | 0.00411089097117988 | 0.018447157125308 | 379 | 379 |
| CCDC14 | -0.230174351373859 | 5.97860893452535E-06 | 0.000116105639523769 | 379 | 379 |
| CCDC150 | 0.0825908372774063 | 0.108426798770985 | 0.22172922504446 | 379 | 379 |
| CCDC151 | 0.11147600943649 | 0.0300216356037193 | 0.0849777288723121 | 379 | 345 |
| CCDC152 | -0.146174344130761 | 0.00434939106143991 | 0.0192381088749947 | 379 | 379 |
| CCDC153 | -0.0536221314811013 | 0.297776923511869 | 0.456602029185163 | 379 | 378 |
| CCDC154 | -0.0216039556300824 | 0.675037960831707 | 0.787919696529416 | 379 | 340 |
| CCDC155 | -0.0286384782526177 | 0.578344807594646 | 0.715327855092424 | 379 | 118 |
| CCDC157 | 0.0540113843533645 | 0.294278513233501 | 0.452671050723937 | 379 | 379 |
| CCDC158 | -0.0115570122237846 | 0.82255863811094 | 0.893192370014442 | 379 | 275 |
| CCDC159 | -0.195706116058426 | 0.00012579433886543 | 0.0011888704247015 | 379 | 379 |
| CCDC15 | -0.0201972460330497 | 0.695102842388862 | 0.802240928922372 | 379 | 379 |
| CCDC160 | 0.0929260988246306 | 0.0707600766056489 | 0.161881942879521 | 379 | 156 |
| CCDC163P | 0.156411144391817 | 0.00225994162258909 | 0.0115015714816983 | 379 | 378 |
| CCDC17 | 0.0155311043563538 | 0.763126442385914 | 0.852898432987312 | 379 | 378 |
| CCDC18 | 0.11692700032096 | 0.0228088615574185 | 0.0687943576149216 | 379 | 379 |
| CCDC19 | 0.070961025483655 | 0.168006090066028 | 0.304388226775329 | 379 | 379 |
| CCDC21 | 0.226439908935526 | 8.51895578651512E-06 | 0.000153837755314227 | 379 | 379 |
| CCDC22 | -0.0646327017670687 | 0.209325918184464 | 0.355778699276663 | 379 | 379 |
| CCDC23 | 0.0471513711193113 | 0.359973090707567 | 0.521217061672969 | 379 | 379 |
| CCDC24 | -0.123405941791493 | 0.0162293695601421 | 0.0530578630896121 | 379 | 379 |
| CCDC25 | 0.0499337146135095 | 0.332296003255385 | 0.493038418280134 | 379 | 379 |
| CCDC27 | 0.0317009415099685 | 0.538378031718267 | 0.683897726498161 | 379 | 19 |
| CCDC28A | -0.166981971603099 | 0.0011024244330247 | 0.00665414662344406 | 379 | 379 |
| CCDC28B | 0.197351060979988 | 0.000109990086308935 | 0.00107751157674583 | 379 | 379 |
| CCDC30 | 0.00753527184604704 | 0.883752768995495 | 0.932245112930634 | 379 | 379 |
| CCDC33 | -0.0589574430556812 | 0.252215856057751 | 0.406249877653379 | 379 | 271 |
| CCDC34 | -0.0208373655378485 | 0.685944953038066 | 0.796200378646789 | 379 | 379 |
| CCDC36 | 0.00907317376790444 | 0.860249941411218 | 0.917735706354333 | 379 | 368 |
| CCDC37 | -0.00737602497818571 | 0.886192640257355 | 0.933854088677831 | 379 | 105 |
| CCDC38 | -0.0309466971733422 | 0.548091969020567 | 0.692201755524828 | 379 | 48 |
| CCDC39 | -0.101476488983612 | 0.0483693006453844 | 0.121879081610965 | 379 | 377 |
| CCDC3 | 0.0522153742517917 | 0.310650105971529 | 0.470124431476376 | 379 | 379 |
| CCDC40 | 0.18669077631993 | 0.000257525445505421 | 0.00211175125454156 | 379 | 378 |
| CCDC41 | 0.040087820955671 | 0.436473780027397 | 0.59297033986867 | 379 | 379 |
| CCDC42B | 0.0143990452851718 | 0.77993170252277 | 0.864845032731562 | 379 | 375 |
| CCDC42 | 0.146299960466393 | 0.00431561806552955 | 0.0191346321563775 | 379 | 101 |
| CCDC43 | -0.00410665114390926 | 0.936488410317703 | 0.963920454546432 | 379 | 379 |
| CCDC45 | -0.113507164779337 | 0.0271332798610151 | 0.0788276444079425 | 379 | 379 |
| CCDC46 | -0.0996520520647121 | 0.0525698435983702 | 0.129630003590161 | 379 | 379 |
| CCDC47 | -0.0113504417323867 | 0.825679017451335 | 0.89507958746493 | 379 | 379 |
| CCDC48 | -0.0955051476773227 | 0.0632552691233013 | 0.148710632698224 | 379 | 379 |
| CCDC50 | -0.0918394701887791 | 0.0741326897442086 | 0.167596466416532 | 379 | 379 |
| CCDC51 | 0.00924226980108201 | 0.857672868916897 | 0.916554612664092 | 379 | 379 |
| CCDC52 | -0.0851574205997438 | 0.0978494545630439 | 0.206553708620892 | 379 | 379 |
| CCDC53 | -0.149676918493959 | 0.00349196811865189 | 0.0162455992155396 | 379 | 379 |
| CCDC54 | -0.044579688586038 | 0.386798702877703 | 0.546153617410983 | 379 | 161 |
| CCDC55 | 0.0254446759270178 | 0.621448357841215 | 0.748282501609437 | 379 | 379 |
| CCDC56 | -0.0619074402139132 | 0.22921563208266 | 0.37937291760726 | 379 | 379 |
| CCDC57 | -0.0550837639372196 | 0.284782956244017 | 0.442425484322367 | 379 | 379 |
| CCDC58 | -0.0525724709657896 | 0.307348195653393 | 0.466909287727205 | 379 | 379 |
| CCDC59 | -0.00052796190636613 | 0.991826317468211 | 0.995541774970117 | 379 | 379 |
| CCDC60 | -0.153366239133352 | 0.0027571697522832 | 0.01348523972577 | 379 | 279 |
| CCDC61 | -0.100161425943086 | 0.0513675880937628 | 0.127330483400816 | 379 | 379 |
| CCDC62 | 0.0301915296640647 | 0.557903432498118 | 0.699823449077793 | 379 | 369 |
| CCDC63 | 0.10288013206088 | 0.0453320172256709 | 0.11611461536631 | 379 | 25 |
| CCDC64B | -0.225766508606271 | 9.07492027215281E-06 | 0.000162252046128265 | 379 | 379 |
| CCDC64 | 0.16138039423061 | 0.00162129257892068 | 0.00890498317308567 | 379 | 379 |
| CCDC65 | 0.0231895773627382 | 0.652694441438548 | 0.771270329057249 | 379 | 356 |
| CCDC66 | -0.213063383613524 | 0.0000288549218042305 | 0.000387363161499175 | 379 | 379 |
| CCDC67 | -0.0968071014837257 | 0.0597231036038981 | 0.142623196756489 | 379 | 242 |
| CCDC68 | 0.251071857309164 | 7.37449223828682E-07 | 0.0000232836675319667 | 379 | 379 |
| CCDC69 | 0.12094619088741 | 0.0185004701988463 | 0.0586642128742563 | 379 | 379 |
| CCDC6 | 0.125523544307362 | 0.0144735875437577 | 0.0486493123949192 | 379 | 379 |
| CCDC70 | 0.0472956712124174 | 0.358503175772044 | 0.51961995388948 | 379 | 59 |
| CCDC71 | -0.0562252929739577 | 0.27490435228749 | 0.431234453888951 | 379 | 379 |
| CCDC72 | -0.110634867169905 | 0.0312926815948537 | 0.0876743380899758 | 379 | 379 |
| CCDC73 | -0.0658857444800234 | 0.200611022719629 | 0.345617808539821 | 379 | 379 |
| CCDC74A | 0.0584454559319576 | 0.256365402221825 | 0.410581969224209 | 379 | 379 |
| CCDC74B | 0.0545464864455636 | 0.289514252146902 | 0.447985686871296 | 379 | 379 |
| CCDC75 | 0.0173848557814146 | 0.735849625902978 | 0.832549294288402 | 379 | 379 |
| CCDC76 | -0.110152442395055 | 0.0320422153952741 | 0.0893591732532569 | 379 | 379 |
| CCDC77 | 0.0464979807081633 | 0.366675934830158 | 0.527685472188443 | 379 | 379 |
| CCDC78 | 0.0737377398760186 | 0.151935046344453 | 0.283749467732675 | 379 | 379 |
| CCDC79 | -0.123627533248034 | 0.0160372938443147 | 0.0525686001562361 | 379 | 120 |
| CCDC7 | -0.0649479109226654 | 0.207108354785781 | 0.353403137581107 | 379 | 379 |
| CCDC80 | 0.108751266217795 | 0.034306664139778 | 0.0940717102148415 | 379 | 379 |
| CCDC81 | 0.132171346762075 | 0.00999759981419128 | 0.0365887897799033 | 379 | 375 |
| CCDC82 | -0.00535024888802599 | 0.917316225582793 | 0.952529254823546 | 379 | 379 |
| CCDC83 | 0.0729415909476053 | 0.156418886039288 | 0.289640798691352 | 379 | 115 |
| CCDC84 | 0.0485725683377417 | 0.345660330617579 | 0.507347178583563 | 379 | 379 |
| CCDC85A | -0.0735374873839442 | 0.153053567628379 | 0.285435114647808 | 379 | 378 |
| CCDC85B | -0.00138139762519649 | 0.97861596512269 | 0.988292336739594 | 379 | 379 |
| CCDC85C | 0.128138762927298 | 0.0125370620148819 | 0.0435350027374919 | 379 | 379 |
| CCDC86 | 0.150231835333784 | 0.00337115878432896 | 0.0158059438107531 | 379 | 379 |
| CCDC87 | 0.171100778594936 | 0.000823802540120997 | 0.00527425145803007 | 379 | 379 |
| CCDC88A | 0.246729157877518 | 1.15725305904476E-06 | 0.0000334489994967048 | 379 | 379 |
| CCDC88B | -0.246261434233963 | 1.21419907338459E-06 | 0.0000347404606451223 | 379 | 379 |
| CCDC88C | 0.0784781899844371 | 0.127230745736484 | 0.249158639650668 | 379 | 379 |
| CCDC89 | 0.0899267202847045 | 0.0803865531279077 | 0.178309047479601 | 379 | 377 |
| CCDC8 | 0.102382810541836 | 0.046389305675917 | 0.118151207828142 | 379 | 379 |
| CCDC90A | 0.139648884727852 | 0.00646825100766381 | 0.0263028057793187 | 379 | 379 |
| CCDC90B | 0.00100659954240101 | 0.984416967741578 | 0.991215703655292 | 379 | 379 |
| CCDC91 | -0.0384462124803428 | 0.455503418183643 | 0.609715910061789 | 379 | 379 |
| CCDC92 | -0.127389723101381 | 0.0130668920545704 | 0.0449030044467974 | 379 | 379 |
| CCDC93 | 0.0030405424945117 | 0.95295397446307 | 0.974178769109804 | 379 | 379 |
| CCDC94 | -0.0290866123105729 | 0.572410550923958 | 0.71087037477892 | 379 | 379 |
| CCDC96 | 0.0948878412442406 | 0.064989132030214 | 0.151814857433445 | 379 | 379 |
| CCDC97 | -0.0667210770561909 | 0.194949708641077 | 0.338516427963037 | 379 | 379 |
| CCDC99 | 0.15724898771277 | 0.00213827989465464 | 0.011012419156159 | 379 | 379 |
| CCDC9 | 0.0285812421350164 | 0.579104806572337 | 0.715999881818064 | 379 | 379 |
| CCHCR1 | -0.0588185031445278 | 0.253337295637015 | 0.407417745390786 | 379 | 379 |
| CCIN | 0.104747515839689 | 0.041540896668863 | 0.108821891815328 | 379 | 330 |
| CCKAR | 0.0469793239645879 | 0.361730570060433 | 0.522921678562137 | 379 | 92 |
| CCKBR | 0.00795360273610377 | 0.87734867811184 | 0.928335001312853 | 379 | 150 |
| CCK | 0.285267700001314 | 1.57564986623158E-08 | 1.25975748175967E-06 | 379 | 204 |
| CCL11 | 0.0190479242846139 | 0.711656588309719 | 0.815130658714408 | 379 | 379 |
| CCL13 | 0.16562101730298 | 0.00121207561499385 | 0.00713358126865482 | 379 | 359 |
| CCL14-CCL15 | -0.052104110391043 | 0.311683658984887 | 0.471221013370365 | 379 | 375 |
| CCL14 | 0.085704544273553 | 0.0957045012871683 | 0.203356186442131 | 379 | 377 |
| CCL15 | -0.0133778766303604 | 0.795179429758783 | 0.874670904984864 | 379 | 379 |
| CCL16 | -0.0625524740489651 | 0.224391154111912 | 0.373382662280211 | 379 | 230 |
| CCL17 | 0.0085957249060302 | 0.867534318694612 | 0.922429776441268 | 379 | 352 |
| CCL18 | 0.230406583141681 | 5.84724524180193E-06 | 0.000113979822937926 | 379 | 376 |
| CCL19 | 0.131528797688394 | 0.0103688586722347 | 0.0375788210113453 | 379 | 370 |
| CCL1 | 0.101669790121264 | 0.0479411760906417 | 0.121154427673368 | 379 | 110 |
| CCL20 | -0.0168237169998011 | 0.744073277270289 | 0.838308562027295 | 379 | 379 |
| CCL21 | 0.1669468745547 | 0.00110513345140324 | 0.00666441182312148 | 379 | 372 |
| CCL22 | 0.094688136707976 | 0.0655583433946659 | 0.152658935153193 | 379 | 379 |
| CCL23 | 0.10177862967857 | 0.0477015164090637 | 0.12070261196515 | 379 | 296 |
| CCL24 | -0.281697190106254 | 2.41446088999303E-08 | 1.76007097524933E-06 | 379 | 377 |
| CCL25 | 0.140627571624744 | 0.00610061983171379 | 0.0250856677775241 | 379 | 275 |
| CCL26 | 0.0904686967061531 | 0.0785726592594489 | 0.175147688341355 | 379 | 375 |
| CCL27 | 0.0168148478183221 | 0.744203494176638 | 0.838329607733355 | 379 | 75 |
| CCL28 | -0.0708030342786942 | 0.168957346965431 | 0.305553290371266 | 379 | 379 |
| CCL2 | 0.137341322761455 | 0.00741477131841459 | 0.0291514024575718 | 379 | 379 |
| CCL3L1 | 0.181415175102121 | 0.00038580502660509 | 0.00287260310459096 | 379 | 370 |
| CCL3L3 | 0.112603432667454 | 0.028387503230533 | 0.081362736926136 | 379 | 242 |
| CCL3 | 0.26079996389484 | 2.60688467116909E-07 | 0.0000103883014848935 | 379 | 378 |
| CCL4L2 | 0.230460779566739 | 5.81698704481179E-06 | 0.000113645657778595 | 379 | 379 |
| CCL4 | 0.293111519489088 | 6.03999965477458E-09 | 0.0000006018146389692 | 379 | 379 |
| CCL5 | 0.266208905813447 | 1.43553770990647E-07 | 0.0000066660050847835 | 379 | 379 |
| CCL7 | 0.177159129798655 | 0.000530281982520309 | 0.00370064567161943 | 379 | 311 |
| CCL8 | 0.220457704066046 | 0.0000148380490687848 | 0.000234214935445245 | 379 | 366 |
| CCM2 | 0.191914430405967 | 0.000170708700173192 | 0.00152882208989794 | 379 | 379 |
| CCNA1 | 0.11718156806057 | 0.0225122549095717 | 0.0681069561103125 | 379 | 235 |
| CCNA2 | 0.163007870006875 | 0.00145121616267313 | 0.00815622109773249 | 379 | 379 |
| CCNB1IP1 | -0.0835288648192858 | 0.10446110647363 | 0.215798378483274 | 379 | 379 |
| CCNB1 | 0.125499409301087 | 0.014492619264473 | 0.0486648382526562 | 379 | 379 |
| CCNB2 | 0.157808500319231 | 0.00206039728911712 | 0.0106839426478972 | 379 | 379 |
| CCNB3 | 0.251835356287509 | 6.80693441663413E-07 | 0.0000220176012419285 | 379 | 375 |
| CCNC | -0.00906139642045975 | 0.860429487155523 | 0.917877864707576 | 379 | 379 |
| CCND1 | 0.0826489516979884 | 0.108177709843714 | 0.221425377390437 | 379 | 379 |
| CCND2 | -0.0568963530810234 | 0.269207150432877 | 0.42491954933793 | 379 | 379 |
| CCND3 | -0.0258143019763936 | 0.616389203658276 | 0.744344948924755 | 379 | 379 |
| CCNDBP1 | -0.0319433362633666 | 0.535274588932596 | 0.680998305028828 | 379 | 379 |
| CCNE1 | 0.0624444052412879 | 0.225194364691044 | 0.37423135170211 | 379 | 379 |
| CCNE2 | -0.0220574559953565 | 0.668617266470217 | 0.783068113382838 | 379 | 379 |
| CCNF | 0.170565937070327 | 0.000855882864175373 | 0.00543924533040682 | 379 | 379 |
| CCNG1 | -0.118764955022701 | 0.0207413212265803 | 0.0640056499028369 | 379 | 379 |
| CCNG2 | 0.0127457909560398 | 0.804657109623593 | 0.881136641609135 | 379 | 379 |
| CCNH | -0.119401359049363 | 0.0200643713235726 | 0.0624252870867406 | 379 | 379 |
| CCNI2 | -0.0302615037513204 | 0.556990732885999 | 0.699165121022004 | 379 | 379 |
| CCNI | 0.0325186547061875 | 0.527944774708773 | 0.674548658258454 | 379 | 379 |
| CCNJL | 0.0299039460775529 | 0.561662089009544 | 0.702519169877997 | 379 | 379 |
| CCNJ | 0.0228273728125519 | 0.657772014486609 | 0.774768798857922 | 379 | 379 |
| CCNK | 0.117340962034201 | 0.0223282437977935 | 0.0676845158267313 | 379 | 379 |
| CCNL1 | -0.170861757183471 | 0.000837999432701701 | 0.00535261325480778 | 379 | 379 |
| CCNL2 | -0.0754382554023237 | 0.142685220632013 | 0.270812471129742 | 379 | 379 |
| CCNO | 0.109331942427093 | 0.0333522174272277 | 0.0921041458422105 | 379 | 379 |
| CCNT1 | 0.0755169423635102 | 0.142267869148365 | 0.270225817556641 | 379 | 379 |
| CCNT2 | -0.0991248464795484 | 0.0538387683879457 | 0.1319548948821 | 379 | 379 |
| CCNYL1 | 0.0447366701657248 | 0.385127194164456 | 0.54499217212412 | 379 | 379 |
| CCNY | -0.0810479574547248 | 0.115206632831238 | 0.23162818046824 | 379 | 379 |
| CCPG1 | 0.0429142719571699 | 0.404801912920997 | 0.563850532448017 | 379 | 379 |
| CCR10 | 0.100572874384163 | 0.0504132785618655 | 0.125674023855781 | 379 | 379 |
| CCR1 | 0.152016744648206 | 0.00300783357452763 | 0.0144510114164608 | 379 | 379 |
| CCR2 | 0.105752477507637 | 0.0396137597675817 | 0.104910061262403 | 379 | 375 |
| CCR3 | 0.051047219318854 | 0.321613812151371 | 0.482007457848631 | 379 | 346 |
| CCR4 | 0.0813468419469908 | 0.113867947341553 | 0.229752076919539 | 379 | 370 |
| CCR5 | 0.185213003098192 | 0.00028872042555962 | 0.00229177672237243 | 379 | 379 |
| CCR6 | 0.0421473834377301 | 0.413257105868418 | 0.571612270328497 | 379 | 379 |
| CCR7 | 0.094741265570874 | 0.065406513700727 | 0.152506813355637 | 379 | 379 |
| CCR8 | 0.0979390146209692 | 0.0567861759233354 | 0.137311743439987 | 379 | 369 |
| CCR9 | 0.146354923165191 | 0.00430091551001991 | 0.0190907886126427 | 379 | 339 |
| CCRL1 | 0.232749632657726 | 4.66702427057917E-06 | 0.000096594736155578 | 379 | 379 |
| CCRL2 | -0.20355943404166 | 0.0000656125011040802 | 0.000729240286934811 | 379 | 379 |
| CCRN4L | 0.264798678874797 | 1.67928903211552E-07 | 7.40144209761279E-06 | 379 | 379 |
| CCS | 0.0139149943678032 | 0.787149261500759 | 0.869455493122225 | 379 | 379 |
| CCT2 | 0.0595739172047057 | 0.247281644612005 | 0.400809323090562 | 379 | 379 |
| CCT3 | -0.0726693471865268 | 0.157974855971566 | 0.291676067315628 | 379 | 379 |
| CCT4 | 0.0008329386038383 | 0.987105131951079 | 0.992963601212497 | 379 | 379 |
| CCT5 | 0.00525194686892569 | 0.918830124094158 | 0.953651784994711 | 379 | 379 |
| CCT6A | 0.0528438673386327 | 0.30485425260046 | 0.46400937441943 | 379 | 379 |
| CCT6B | -0.143342379570761 | 0.00517694744634018 | 0.0220512382311564 | 379 | 379 |
| CCT6P1 | -0.049489918212525 | 0.336616381174549 | 0.497609006630057 | 379 | 379 |
| CCT7 | 0.0848954687541414 | 0.098889871128241 | 0.207977672990894 | 379 | 379 |
| CCT8L2 | -0.0528556075399051 | 0.304746671061575 | 0.463881237049662 | 379 | 11 |
| CCT8 | 0.0896843164262141 | 0.0812087261807138 | 0.179810901475287 | 379 | 379 |
| CD101 | 0.183016315872275 | 0.000341661407950502 | 0.00261158920464247 | 379 | 379 |
| CD109 | 0.2562825225275 | 4.24741016870541E-07 | 0.0000153122997863802 | 379 | 379 |
| CD14 | 0.214749427444843 | 0.0000248445769376153 | 0.000343565340993156 | 379 | 379 |
| CD151 | -0.0108181945544543 | 0.833731484819688 | 0.900295604019429 | 379 | 379 |
| CD160 | 0.073727667876112 | 0.151991155586734 | 0.283827522412296 | 379 | 378 |
| CD163L1 | 0.135913380056415 | 0.00806071157392517 | 0.0310284972020556 | 379 | 379 |
| CD163 | 0.231282371391198 | 5.37617703407231E-06 | 0.000107026946015648 | 379 | 379 |
| CD164L2 | 0.171010645321398 | 0.000829129779754317 | 0.00530151089099278 | 379 | 354 |
| CD164 | -0.182617333074406 | 0.000352198258741323 | 0.00267255532886451 | 379 | 379 |
| CD177 | 0.0656133274729178 | 0.202482873540976 | 0.347809256671304 | 379 | 377 |
| CD180 | 0.165238958961325 | 0.00124461376857759 | 0.00725829464804602 | 379 | 379 |
| CD19 | 0.131267945456556 | 0.0105230305857269 | 0.0379695439059324 | 379 | 358 |
| CD1A | -0.0548477274592775 | 0.286855052168371 | 0.444944018807276 | 379 | 369 |
| CD1B | 0.0977037441957137 | 0.0573865631469975 | 0.138358557159371 | 379 | 319 |
| CD1C | -0.0186663979135241 | 0.717182263117679 | 0.819329909719828 | 379 | 374 |
| CD1D | 0.11633850549953 | 0.0235075130528093 | 0.0703345358097334 | 379 | 379 |
| CD1E | -0.0738341051827699 | 0.151399006984949 | 0.282908256573139 | 379 | 369 |
| CD200R1L | 0.00721321733813068 | 0.888688178382047 | 0.935342067039611 | 379 | 67 |
| CD200R1 | 0.110103408526232 | 0.0321192494114502 | 0.0895095540871727 | 379 | 372 |
| CD200 | 0.0815862099568968 | 0.11280465558334 | 0.228170502024632 | 379 | 379 |
| CD207 | -0.0863297097771974 | 0.0932996692545512 | 0.199591728767091 | 379 | 366 |
| CD209 | 0.197323872278565 | 0.000110235418494406 | 0.00107831666398968 | 379 | 378 |
| CD226 | 0.177066856216283 | 0.000533909362188602 | 0.00372034283056653 | 379 | 373 |
| CD22 | 0.19763274082279 | 0.000107478413278881 | 0.00105971257011121 | 379 | 378 |
| CD244 | 0.216620382179325 | 0.0000210142226520114 | 0.000305021808672107 | 379 | 377 |
| CD247 | 0.24054948847742 | 0.0000021661792523669 | 0.0000533433960812854 | 379 | 379 |
| CD248 | 0.169325633461544 | 0.000934768158327954 | 0.00582848523375053 | 379 | 379 |
| CD24 | -0.261194103457275 | 2.49709822648768E-07 | 0.0000100635088688613 | 379 | 379 |
| CD274 | 0.269418242253219 | 1.00124666680152E-07 | 5.05158241967952E-06 | 379 | 377 |
| CD276 | 0.146412800005461 | 0.00428548233163421 | 0.0190521398366913 | 379 | 379 |
| CD27 | 0.125519742669636 | 0.0144765838775933 | 0.0486511364618507 | 379 | 379 |
| CD28 | 0.144004092734839 | 0.0049718123132959 | 0.02133562541691 | 379 | 378 |
| CD2AP | 0.0261165670903431 | 0.612265464094145 | 0.741147718074401 | 379 | 379 |
| CD2BP2 | -0.101541418443522 | 0.0482251391225168 | 0.121608553798965 | 379 | 379 |
| CD2 | 0.169017292781537 | 0.000955394034065005 | 0.00593545014748542 | 379 | 379 |
| CD300A | 0.270765040519215 | 8.59540683772255E-08 | 4.48499280995691E-06 | 379 | 379 |
| CD300C | 0.24366129412836 | 1.58307370662203E-06 | 0.0000424361184086724 | 379 | 379 |
| CD300E | 0.0937133090011032 | 0.0683959171763144 | 0.157637364381258 | 379 | 265 |
| CD300LB | 0.185579209373737 | 0.000280677186602444 | 0.00225021491610066 | 379 | 377 |
| CD300LD | -0.108084391776168 | 0.0354313777715984 | 0.0963743148389728 | 379 | 128 |
| CD300LF | 0.211089988129556 | 0.0000343267102832642 | 0.000443985656553531 | 379 | 379 |
| CD300LG | 0.0508448430583291 | 0.32353847904394 | 0.484196615781058 | 379 | 247 |
| CD302 | -0.101637603838622 | 0.0480122415865022 | 0.121262269644149 | 379 | 379 |
| CD320 | -0.0234525390602499 | 0.649018105229896 | 0.769201812261832 | 379 | 379 |
| CD33 | 0.171357418157779 | 0.000808806854085623 | 0.00519501856262058 | 379 | 378 |
| CD34 | 0.128880313625123 | 0.0120312843753331 | 0.0421728096480998 | 379 | 379 |
| CD36 | 0.168485997247049 | 0.000991921737614077 | 0.00611181610112241 | 379 | 379 |
| CD37 | 0.226371924158601 | 8.57357785710343E-06 | 0.00015468325909977 | 379 | 379 |
| CD38 | 0.165272742592312 | 0.00124170451964111 | 0.00725190603812688 | 379 | 377 |
| CD3D | 0.167827450941357 | 0.00103898486978844 | 0.00634657794151733 | 379 | 379 |
| CD3EAP | 0.0438640924697824 | 0.394473924586922 | 0.554293032152894 | 379 | 379 |
| CD3E | 0.189446882290875 | 0.000207585416388595 | 0.0017688025939635 | 379 | 379 |
| CD3G | 0.145026963072297 | 0.00466909652621217 | 0.0203023784916085 | 379 | 373 |
| CD40LG | -0.017297055162677 | 0.737134408997974 | 0.833289684242408 | 379 | 374 |
| CD40 | 0.13485352748099 | 0.00857210939341376 | 0.0324945290756942 | 379 | 379 |
| CD44 | -0.0754573883375207 | 0.142583654527541 | 0.270695969166228 | 379 | 379 |
| CD46 | -0.296858156343696 | 3.78107997191595E-09 | 4.25973032290622E-07 | 379 | 379 |
| CD47 | -0.0581873899515534 | 0.258474775139636 | 0.412577510986049 | 379 | 379 |
| CD48 | 0.202879835894526 | 0.0000694831765223643 | 0.000762430782559734 | 379 | 379 |
| CD4 | 0.166447856879328 | 0.00114432006347159 | 0.00683421030678153 | 379 | 379 |
| CD52 | 0.228170463548551 | 7.23506443206364E-06 | 0.000135533992589261 | 379 | 379 |
| CD53 | 0.234109512969204 | 4.09019304603683E-06 | 0.0000873926160741576 | 379 | 379 |
| CD55 | 0.0960007786081335 | 0.0618908806981557 | 0.146318395431386 | 379 | 379 |
| CD58 | -0.0246180423100555 | 0.632827109439098 | 0.757390953459192 | 379 | 379 |
| CD59 | -0.0267420556041521 | 0.60377091079002 | 0.734951291996297 | 379 | 379 |
| CD5L | 0.148291003888245 | 0.00381101510922016 | 0.0173872083722083 | 379 | 90 |
| CD5 | -0.0304133707483152 | 0.555012367908296 | 0.69769766251732 | 379 | 379 |
| CD63 | 0.10713459439844 | 0.0370872493316128 | 0.0997512180883368 | 379 | 379 |
| CD68 | 0.186326623604985 | 0.000264905980359812 | 0.00215976800105854 | 379 | 379 |
| CD69 | 0.154082521129128 | 0.00263198783678975 | 0.0130044990849407 | 379 | 379 |
| CD6 | 0.132544020395658 | 0.00978771854956302 | 0.0359523681735338 | 379 | 379 |
| CD70 | 0.121527846259303 | 0.0179397829698924 | 0.0572525376995053 | 379 | 379 |
| CD72 | 0.16095465404393 | 0.00166870319637234 | 0.00909047897318905 | 379 | 379 |
| CD74 | 0.189856227151879 | 0.000200992181936563 | 0.00172455782752313 | 379 | 379 |
| CD79A | 0.0971590402492604 | 0.0587967904872592 | 0.140918984864182 | 379 | 379 |
| CD79B | 0.161687944343332 | 0.00158781535997021 | 0.00876342543121009 | 379 | 379 |
| CD7 | 0.267639562285589 | 1.22324826039311E-07 | 5.83042464112369E-06 | 379 | 379 |
| CD80 | 0.189126579923562 | 0.000212884977691358 | 0.00180311120788734 | 379 | 376 |
| CD81 | 0.0691236600900519 | 0.1793187182691 | 0.318881752990108 | 379 | 379 |
| CD82 | 0.17295989891398 | 0.00072073534477817 | 0.00475249099310328 | 379 | 379 |
| CD83 | 0.165999406422322 | 0.00118062300323606 | 0.00698877915569756 | 379 | 379 |
| CD84 | 0.180861644650339 | 0.000402262257813296 | 0.00296949219952421 | 379 | 378 |
| CD86 | 0.231467473296661 | 0.0000052813572829651 | 0.000105456950862671 | 379 | 379 |
| CD8A | 0.242282872975791 | 1.81993802869497E-06 | 0.0000467166355177283 | 379 | 379 |
| CD8B | 0.0976173214236856 | 0.0576084218733872 | 0.138741623819449 | 379 | 378 |
| CD93 | 0.145689235943607 | 0.00448206826533481 | 0.0196833775337893 | 379 | 379 |
| CD96 | 0.18372343873903 | 0.000323705102388122 | 0.00249549952183191 | 379 | 378 |
| CD97 | 0.108936272000567 | 0.0340000812333385 | 0.0934118900782369 | 379 | 379 |
| CD99L2 | 0.035056044996937 | 0.49623652210565 | 0.646987399834102 | 379 | 379 |
| CD99 | 0.0252855951191593 | 0.623631249316083 | 0.750052190430626 | 379 | 379 |
| CD9 | -0.0922348912716029 | 0.0728905677832007 | 0.165457833772788 | 379 | 379 |
| CDADC1 | -0.188100822869287 | 0.000230720550694257 | 0.00192863704855216 | 379 | 379 |
| CDAN1 | 0.0446194859743206 | 0.386374531582224 | 0.54577432586823 | 379 | 379 |
| CDA | 0.241711075566395 | 0.0000019278374693294 | 0.0000487565833442135 | 379 | 379 |
| CDC123 | 0.0168278556060025 | 0.744012516941886 | 0.838292998404575 | 379 | 379 |
| CDC14A | 0.0742177767306116 | 0.149279027067851 | 0.28010831349497 | 379 | 378 |
| CDC14B | -0.079214556692165 | 0.12368987252077 | 0.244007839253987 | 379 | 379 |
| CDC14C | -0.0698080283353273 | 0.175040900761846 | 0.313750766615973 | 379 | 378 |
| CDC16 | -0.26594056596003 | 1.47912588398155E-07 | 6.83091040797507E-06 | 379 | 379 |
| CDC20B | 0.0535212107599166 | 0.298688448496743 | 0.457539752533484 | 379 | 172 |
| CDC20 | 0.259641315311748 | 2.95717579549043E-07 | 0.000011232735952679 | 379 | 379 |
| CDC23 | -0.0914027830529339 | 0.0755244051613606 | 0.169880647253484 | 379 | 379 |
| CDC25A | 0.034788728726651 | 0.499528653529133 | 0.649954337041516 | 379 | 379 |
| CDC25B | 0.25047743112446 | 7.84752913824135E-07 | 0.0000242735053990205 | 379 | 379 |
| CDC25C | -0.0484129597063595 | 0.34724948658663 | 0.508995078007394 | 379 | 379 |
| CDC26 | 0.00547305382256116 | 0.915425390454332 | 0.95156249761093 | 379 | 379 |
| CDC27 | 0.244498877237388 | 1.45388080824827E-06 | 0.0000395982811345422 | 379 | 379 |
| CDC34 | 0.00945060007715567 | 0.8544999342365 | 0.914455132558362 | 379 | 379 |
| CDC37L1 | -0.107141068856535 | 0.0370757433852999 | 0.0997413457365504 | 379 | 379 |
| CDC37 | 0.0529866878251452 | 0.303547219220179 | 0.462588137936954 | 379 | 379 |
| CDC40 | -0.0424811646562259 | 0.409564328853096 | 0.568328190391154 | 379 | 379 |
| CDC42BPA | 0.0250361949906569 | 0.627060124610302 | 0.752760679952357 | 379 | 379 |
| CDC42BPB | 0.0704299680662226 | 0.171219522921428 | 0.30871516781723 | 379 | 379 |
| CDC42BPG | -0.0236284081086449 | 0.646564104929656 | 0.767775759275987 | 379 | 379 |
| CDC42EP1 | 0.275124790779661 | 5.21480666031369E-08 | 3.04058610210371E-06 | 379 | 379 |
| CDC42EP2 | 0.204994823601255 | 0.0000580951075141656 | 0.000666570671139302 | 379 | 379 |
| CDC42EP3 | -0.0855404997972261 | 0.0963436517365692 | 0.204419681822653 | 379 | 379 |
| CDC42EP4 | 0.0654573896406147 | 0.203560052551127 | 0.349121072743166 | 379 | 379 |
| CDC42EP5 | 0.120747914248166 | 0.0186950594059276 | 0.0591299470251608 | 379 | 379 |
| CDC42SE1 | -0.00218909586524807 | 0.966118902933607 | 0.981525199668057 | 379 | 379 |
| CDC42SE2 | -0.0565660092674273 | 0.272001554668656 | 0.428200326346661 | 379 | 379 |
| CDC42 | 0.179184683213033 | 0.000456193982956404 | 0.00327851188621224 | 379 | 379 |
| CDC45 | 0.208909444256438 | 0.0000415093017968636 | 0.000513761820242329 | 379 | 379 |
| CDC5L | 0.0256202268738398 | 0.619043310672723 | 0.746059022675095 | 379 | 379 |
| CDC6 | 0.189258268946042 | 0.000210690980449722 | 0.00178911381599875 | 379 | 379 |
| CDC73 | -0.0208042848374972 | 0.686417113078949 | 0.796359913789205 | 379 | 379 |
| CDC7 | 0.252778853784348 | 6.16333733687542E-07 | 0.0000203001084245126 | 379 | 379 |
| CDCA2 | 0.191753413372294 | 0.000172914237465335 | 0.00153953457587007 | 379 | 379 |
| CDCA3 | 0.175775643234612 | 0.00058714440027666 | 0.00401306417396953 | 379 | 379 |
| CDCA4 | 0.17054969705398 | 0.000856874767818665 | 0.00544206050490342 | 379 | 379 |
| CDCA5 | 0.198242136837264 | 0.000102227844076887 | 0.00101755707447616 | 379 | 379 |
| CDCA7L | -0.0438297211841147 | 0.394844873474028 | 0.554617749450483 | 379 | 379 |
| CDCA7 | -0.260792086462587 | 2.60912554946388E-07 | 0.0000103883014848935 | 379 | 379 |
| CDCA8 | 0.20136599235399 | 0.0000788924887967451 | 0.000836513512225594 | 379 | 379 |
| CDCP1 | 0.0928862992127714 | 0.0708813542254224 | 0.16210328622626 | 379 | 379 |
| CDCP2 | 0.0603022006188388 | 0.241539768317657 | 0.393788071550938 | 379 | 228 |
| CDH10 | 0.040680368425134 | 0.42971864495097 | 0.586768217897379 | 379 | 97 |
| CDH11 | 0.0824164815400397 | 0.109176832284204 | 0.222849313416842 | 379 | 379 |
| CDH12 | 0.00219335847164699 | 0.966052969303178 | 0.981525199668057 | 379 | 331 |
| CDH13 | 0.0460597299913056 | 0.371214894825605 | 0.531670682938609 | 379 | 379 |
| CDH15 | 0.126604792021521 | 0.0136431787683945 | 0.046345202778778 | 379 | 364 |
| CDH16 | 0.218430492785582 | 0.0000178465690007834 | 0.000269506298665296 | 379 | 311 |
| CDH17 | -0.180165438434665 | 0.000423889017659361 | 0.00308435649253204 | 379 | 379 |
| CDH18 | 0.096276319978167 | 0.0611429135145435 | 0.145000303308331 | 379 | 39 |
| CDH19 | 0.0833521844886599 | 0.10519915539625 | 0.216738243266505 | 379 | 268 |
| CDH1 | -0.182177712036626 | 0.000364158442829489 | 0.00274077258122368 | 379 | 379 |
| CDH20 | 0.044341405374976 | 0.389344304263524 | 0.549108675221364 | 379 | 169 |
| CDH22 | -0.0972921150648128 | 0.0584496437276869 | 0.140289524715384 | 379 | 210 |
| CDH23 | 0.088425186715002 | 0.0855893670952451 | 0.187300986730648 | 379 | 379 |
| CDH24 | 0.0592687021237292 | 0.249716083266806 | 0.403501792764586 | 379 | 379 |
| CDH26 | -0.166721802731602 | 0.00112265219694417 | 0.00673523381573647 | 379 | 357 |
| CDH2 | 0.0992215129146975 | 0.0536042148992011 | 0.131493798468559 | 379 | 375 |
| CDH3 | 0.0886635055146957 | 0.084745945202342 | 0.185919739043155 | 379 | 379 |
| CDH4 | 0.0172488621458232 | 0.737839926996973 | 0.833823962943043 | 379 | 259 |
| CDH5 | 0.102635911615389 | 0.0458486659127356 | 0.117131707605564 | 379 | 379 |
| CDH6 | 0.102556687883233 | 0.0460173214700103 | 0.117414933741779 | 379 | 379 |
| CDH7 | 0.185867246413774 | 0.000274498290480616 | 0.00221538555576882 | 379 | 211 |
| CDH8 | -0.0027774935934947 | 0.95701999595668 | 0.976750747999632 | 379 | 308 |
| CDH9 | -0.0367235642281488 | 0.47596307002496 | 0.627987473546374 | 379 | 56 |
| CDHR1 | -0.253332957701424 | 5.81303274164228E-07 | 0.0000194041773066133 | 379 | 379 |
| CDHR2 | -0.0794790492958104 | 0.122436908771087 | 0.242186654739936 | 379 | 379 |
| CDHR3 | -0.0208813077364546 | 0.685317955917134 | 0.795765075540228 | 379 | 379 |
| CDHR4 | 0.151017273362096 | 0.00320663880521611 | 0.0151533597321696 | 379 | 146 |
| CDHR5 | -0.16428049725611 | 0.00132980896815946 | 0.00762505847908204 | 379 | 379 |
| CDIPT | -0.109310276268316 | 0.0333874189604436 | 0.0921756813071117 | 379 | 379 |
| CDK10 | -0.139356935960363 | 0.00658169761609545 | 0.0266206174829167 | 379 | 379 |
| CDK11A | -0.0169654336759098 | 0.741993590611844 | 0.836826626167547 | 379 | 379 |
| CDK11B | 0.0464934518001381 | 0.366722663738938 | 0.527714418797856 | 379 | 379 |
| CDK12 | 0.00408460180294086 | 0.936828702850089 | 0.964056441774526 | 379 | 379 |
| CDK13 | -0.0115770532984275 | 0.822256052717229 | 0.893072547661369 | 379 | 379 |
| CDK14 | 0.0916042646120334 | 0.0748796695936172 | 0.168813426799573 | 379 | 379 |
| CDK15 | 0.0661962700201357 | 0.198492696640647 | 0.34310114105054 | 379 | 178 |
| CDK16 | 0.0574017986735553 | 0.264969649218594 | 0.41996948079187 | 379 | 379 |
| CDK17 | 0.109420735680193 | 0.0332082831142387 | 0.0918345659120117 | 379 | 379 |
| CDK18 | -0.195121136532574 | 0.000131911301657009 | 0.00124075101427411 | 379 | 379 |
| CDK19 | -0.117224570836765 | 0.0224624818261233 | 0.0679907347098666 | 379 | 379 |
| CDK1 | 0.0903003179603855 | 0.0791326045376014 | 0.176138453962906 | 379 | 379 |
| CDK20 | -0.184539603214555 | 0.000304074885779038 | 0.00238214019566447 | 379 | 379 |
| CDK2AP1 | 0.0864311948751099 | 0.0929138812587452 | 0.199038076663613 | 379 | 379 |
| CDK2AP2 | 0.147510469526264 | 0.00400209682458883 | 0.0180759853844983 | 379 | 379 |
| CDK2 | 0.116427184608143 | 0.0234010652386248 | 0.0701218560603676 | 379 | 379 |
| CDK3 | -0.0655061925838946 | 0.203222488434736 | 0.348692930138797 | 379 | 379 |
| CDK4 | 0.0750651836585007 | 0.144676723000622 | 0.273517359234966 | 379 | 379 |
| CDK5R1 | 0.134879185928937 | 0.00855939520478864 | 0.0324696970242793 | 379 | 379 |
| CDK5R2 | 0.174596017528443 | 0.000640040168691494 | 0.00432097938876913 | 379 | 340 |
| CDK5RAP1 | -0.21799374760487 | 0.0000185665094704498 | 0.000278040385678275 | 379 | 379 |
| CDK5RAP2 | 0.0576175953673101 | 0.263174494904564 | 0.417951766593676 | 379 | 379 |
| CDK5RAP3 | -0.015667092485494 | 0.761115004387204 | 0.851466277758378 | 379 | 379 |
| CDK5 | -0.0278501446664491 | 0.588853378348494 | 0.723720621452547 | 379 | 379 |
| CDK6 | 0.0480338013866624 | 0.351043135768548 | 0.512327638452728 | 379 | 379 |
| CDK7 | -0.0693929572848732 | 0.177626260775654 | 0.316816448950133 | 379 | 379 |
| CDK8 | -0.0803511236745705 | 0.118375579873176 | 0.236298298371624 | 379 | 379 |
| CDK9 | 0.157863226007456 | 0.002052920493192 | 0.0106586298871461 | 379 | 379 |
| CDKAL1 | -0.0932149592232931 | 0.0698849301066838 | 0.160379443767977 | 379 | 379 |
| CDKL1 | 0.106892689419834 | 0.0375193210563445 | 0.100749336119339 | 379 | 378 |
| CDKL2 | -0.0964274706741543 | 0.0607357867857597 | 0.144293528454287 | 379 | 375 |
| CDKL3 | -0.0712450897499505 | 0.166305837590438 | 0.302496298297699 | 379 | 379 |
| CDKL4 | -0.110388424858764 | 0.0316736807020236 | 0.0885291430729805 | 379 | 137 |
| CDKL5 | -0.0485224466931525 | 0.34615887276147 | 0.507890937480718 | 379 | 375 |
| CDKN1A | 0.0892691101511457 | 0.0826327861521851 | 0.182226645494911 | 379 | 379 |
| CDKN1B | -0.194351278951876 | 0.000140387526525085 | 0.00130074947473803 | 379 | 379 |
| CDKN1C | 0.0307069047889593 | 0.551198234939125 | 0.694841286945958 | 379 | 379 |
| CDKN2AIPNL | 0.0363591435261403 | 0.480354655154335 | 0.632225164447404 | 379 | 379 |
| CDKN2AIP | 0.00998888335809987 | 0.846312677938015 | 0.908783524405901 | 379 | 379 |
| CDKN2A | 0.34455330980751 | 5.27655683779234E-12 | 2.2260333825478E-09 | 379 | 379 |
| CDKN2BAS | 0.0706555443894806 | 0.169849002953415 | 0.306718217719519 | 379 | 358 |
| CDKN2B | 0.203800134651386 | 0.0000642910112448842 | 0.000719477334148263 | 379 | 379 |
| CDKN2C | 0.054468387904547 | 0.290206356277926 | 0.448649993283647 | 379 | 379 |
| CDKN2D | -0.0189003850018145 | 0.713791641097862 | 0.816861547927629 | 379 | 379 |
| CDKN3 | 0.153139823467492 | 0.0027978520000843 | 0.0136442806555473 | 379 | 379 |
| CDNF | -0.130167525690474 | 0.0111959976797176 | 0.0398080453357665 | 379 | 379 |
| CDO1 | 0.0345156668741305 | 0.502903432948737 | 0.652589611813321 | 379 | 359 |
| CDON | 0.00676515335642111 | 0.895561767713677 | 0.939733209686007 | 379 | 379 |
| CDR1 | 0.0545154590330499 | 0.289789082363905 | 0.448201086202145 | 379 | 251 |
| CDR2L | 0.287314796852545 | 1.23028805062117E-08 | 1.01642297782152E-06 | 379 | 379 |
| CDR2 | -0.0587684547034673 | 0.253742102671073 | 0.407912957010056 | 379 | 379 |
| CDRT15P | 0.112319865280695 | 0.0287911617873942 | 0.0821863089388531 | 379 | 371 |
| CDRT15 | 0.0101004988379422 | 0.844617049429479 | 0.907583699548367 | 379 | 54 |
| CDRT1 | 0.197554986859202 | 0.000108166287448088 | 0.00106543524466999 | 379 | 353 |
| CDRT4 | 0.0159920864514475 | 0.756314461163115 | 0.847674135771988 | 379 | 379 |
| CDS1 | 0.072464590872843 | 0.159152783673679 | 0.293360732051845 | 379 | 379 |
| CDS2 | 0.0323259305281894 | 0.530394480419472 | 0.676922100782524 | 379 | 379 |
| CDSN | -0.0839135671029788 | 0.102868254694093 | 0.213700026758806 | 379 | 379 |
| CDT1 | 0.122700693405569 | 0.016854145687887 | 0.0544983693247592 | 379 | 379 |
| CDV3 | 0.0565289355633195 | 0.272316393956735 | 0.428428902592569 | 379 | 379 |
| CDX1 | -0.198160517466998 | 0.000102916818986929 | 0.00102133868211853 | 379 | 379 |
| CDX2 | -0.23160281235042 | 5.21304049857906E-06 | 0.00010451381901499 | 379 | 379 |
| CDYL2 | 0.0455452271097954 | 0.376587698371107 | 0.537270174363384 | 379 | 379 |
| CDYL | -0.0899453212359302 | 0.0803237426042234 | 0.178209596996368 | 379 | 379 |
| CEACAM16 | 0.0134668865821093 | 0.793847168366237 | 0.873826105390064 | 379 | 204 |
| CEACAM18 | 0.208501216806493 | 0.00004300294162636 | 0.000525987095115282 | 379 | 226 |
| CEACAM19 | -0.021086248375022 | 0.682396580115435 | 0.793488118140326 | 379 | 379 |
| CEACAM1 | -0.130161476304512 | 0.0111998000733756 | 0.0398116952052512 | 379 | 379 |
| CEACAM20 | 0.12306043665749 | 0.0165328715996678 | 0.0537047473915814 | 379 | 128 |
| CEACAM21 | 0.0690285003570404 | 0.179919617167518 | 0.319606358107645 | 379 | 378 |
| CEACAM22P | 0.0157311408507661 | 0.760168203145781 | 0.850551048584987 | 379 | 272 |
| CEACAM3 | 0.189731211035023 | 0.000202984711236909 | 0.00173706553923411 | 379 | 366 |
| CEACAM4 | 0.162939894566018 | 0.00145797866064785 | 0.00818019266647582 | 379 | 340 |
| CEACAM5 | -0.234466214863474 | 3.95054977041293E-06 | 0.0000847743515668264 | 379 | 379 |
| CEACAM6 | -0.0534744215111557 | 0.299111682029629 | 0.458011153856165 | 379 | 379 |
| CEACAM7 | -0.0233200030618569 | 0.6508699675501 | 0.770382623960326 | 379 | 379 |
| CEACAM8 | -0.0705114628155462 | 0.170723441936705 | 0.308016779501454 | 379 | 323 |
| CEBPA | -0.136838606776261 | 0.00763670393160148 | 0.0298189376832994 | 379 | 379 |
| CEBPB | -0.138968101400758 | 0.00673555899797009 | 0.0270952858210085 | 379 | 379 |
| CEBPD | 0.0131741529212828 | 0.79823090959848 | 0.876762822707659 | 379 | 379 |
| CEBPE | 0.126581922630036 | 0.0136602992813637 | 0.0463954118107022 | 379 | 288 |
| CEBPG | 0.0266081573392617 | 0.6055848986997 | 0.735939520132573 | 379 | 379 |
| CEBPZ | -0.0797886481497257 | 0.120982840170199 | 0.239956762518227 | 379 | 379 |
| CECR1 | 0.153922363063191 | 0.00265952013610014 | 0.0131068144348414 | 379 | 379 |
| CECR2 | -0.101781043909245 | 0.047696211776813 | 0.12070261196515 | 379 | 371 |
| CECR4 | -0.0569453359325211 | 0.268794477482781 | 0.424504731145247 | 379 | 337 |
| CECR5 | 0.0401413216075309 | 0.435861372546512 | 0.592381883258088 | 379 | 379 |
| CECR6 | 0.170858378170419 | 0.000838201742796962 | 0.00535261325480778 | 379 | 379 |
| CECR7 | 0.140882756965486 | 0.00600789649269527 | 0.0247969549661036 | 379 | 316 |
| CELA1 | 0.0733821505169319 | 0.153925498368774 | 0.286602171555067 | 379 | 62 |
| CELA2A | 0.00867747794302685 | 0.866286209979215 | 0.921646347130326 | 379 | 24 |
| CELA2B | -0.00772857690775971 | 0.880792558616733 | 0.930233972436044 | 379 | 16 |
| CELA3A | 0.144254555881862 | 0.00489609739265691 | 0.0210676690758683 | 379 | 119 |
| CELA3B | 0.062103551048392 | 0.227741121737138 | 0.377373471653349 | 379 | 140 |
| CELF1 | 0.111846462587497 | 0.0294760207320518 | 0.0837681724344451 | 379 | 379 |
| CELF2 | -0.312609327477006 | 4.89664740251895E-10 | 8.36989006009877E-08 | 379 | 379 |
| CELF3 | -0.0513477183151953 | 0.318769729506151 | 0.479048521801422 | 379 | 368 |
| CELF4 | 0.052281573738535 | 0.310036235424646 | 0.469631531219633 | 379 | 340 |
| CELF5 | -0.136201225079035 | 0.00792660336068861 | 0.0306482359200986 | 379 | 373 |
| CELF6 | 0.112547615193457 | 0.0284665733796353 | 0.0815018691732535 | 379 | 378 |
| CELP | -0.221001984249848 | 0.0000141163440615191 | 0.000226302935618461 | 379 | 341 |
| CELSR1 | -0.0597770445406563 | 0.245670674938992 | 0.399078989242203 | 379 | 379 |
| CELSR2 | -0.0445916605559856 | 0.386671072644008 | 0.546047132196118 | 379 | 379 |
| CELSR3 | -0.131514212876222 | 0.0103774256243425 | 0.0375961255763682 | 379 | 379 |
| CEL | -0.237126914033133 | 3.04350601276523E-06 | 0.000069363950828861 | 379 | 378 |
| CEMP1 | -0.0311638739699455 | 0.545286120235932 | 0.689624517925632 | 379 | 379 |
| CEND1 | 0.0801404941365565 | 0.119346729127267 | 0.237734272165507 | 379 | 374 |
| CENPA | 0.155383574734111 | 0.00241776428304211 | 0.0121488672590368 | 379 | 379 |
| CENPBD1 | -0.145243906432387 | 0.00460706990524484 | 0.0200899454764008 | 379 | 379 |
| CENPB | 0.18694874721538 | 0.000252413673159795 | 0.00207842953131745 | 379 | 379 |
| CENPC1 | -0.0134662174556393 | 0.793857181324502 | 0.873826105390064 | 379 | 379 |
| CENPE | 0.202205265004743 | 0.0000735372646928481 | 0.000795036469100213 | 379 | 379 |
| CENPF | 0.066630290980221 | 0.195559280583643 | 0.339334722403649 | 379 | 379 |
| CENPH | 0.0805128821767103 | 0.117633959495691 | 0.235339133173298 | 379 | 379 |
| CENPI | 0.128818806166759 | 0.0120725397923318 | 0.042247383697674 | 379 | 379 |
| CENPJ | -0.123389263706379 | 0.0162439072854512 | 0.0530726237146931 | 379 | 379 |
| CENPK | 0.017045652092132 | 0.74081721855192 | 0.836022982893993 | 379 | 379 |
| CENPL | -0.0112753200784686 | 0.826814461237988 | 0.895387965028683 | 379 | 379 |
| CENPM | 0.265819415239656 | 1.49922033054173E-07 | 6.86525189699342E-06 | 379 | 379 |
| CENPN | 0.0967466866287415 | 0.0598833234141887 | 0.142884059766129 | 379 | 379 |
| CENPO | 0.182875124849015 | 0.000345356116817932 | 0.00263154185094364 | 379 | 379 |
| CENPP | 0.0299024031431767 | 0.561682287677824 | 0.702519169877997 | 379 | 379 |
| CENPQ | -0.0270099417347028 | 0.600149041718156 | 0.732037818908418 | 379 | 379 |
| CENPT | -0.161779950292334 | 0.00157792427340211 | 0.00871992265691665 | 379 | 379 |
| CENPV | 0.0549248866469461 | 0.286176581488636 | 0.44417293602792 | 379 | 379 |
| CENPW | 0.0427459883575653 | 0.40664841617787 | 0.565350217078587 | 379 | 379 |
| CEP110 | -0.035297309359808 | 0.493275158879562 | 0.644439602705669 | 379 | 379 |
| CEP120 | 0.0323136420631657 | 0.530550873120098 | 0.677034541911784 | 379 | 379 |
| CEP135 | 0.209052971035841 | 0.0000409959099954613 | 0.000509954142653706 | 379 | 379 |
| CEP152 | 0.0391259267234923 | 0.447568266910782 | 0.602687873072594 | 379 | 379 |
| CEP164 | -0.00434763529411552 | 0.932770013258192 | 0.961570832696919 | 379 | 379 |
| CEP170L | 0.0585289575116571 | 0.255685436144403 | 0.409964978013243 | 379 | 331 |
| CEP170 | 0.125868188367488 | 0.0142042026767855 | 0.0479144148818139 | 379 | 379 |
| CEP192 | 0.220179361916794 | 0.0000152205346632933 | 0.000239328121573179 | 379 | 379 |
| CEP250 | -0.173587808462514 | 0.000688713326026018 | 0.00458710373814037 | 379 | 379 |
| CEP290 | -0.0327458099184285 | 0.525064810782669 | 0.67206668828344 | 379 | 379 |
| CEP350 | -0.0805404586277217 | 0.11750789181094 | 0.235134370655699 | 379 | 379 |
| CEP55 | 0.157120534856854 | 0.00215653548839907 | 0.0110805352847828 | 379 | 379 |
| CEP57 | -0.0462526504203935 | 0.369212556658194 | 0.53002798822898 | 379 | 379 |
| CEP63 | -0.257028689800602 | 3.92085943019075E-07 | 0.0000142973380375617 | 379 | 379 |
| CEP68 | -0.384594436346261 | 8.28597840203502E-15 | 1.82549310839501E-11 | 379 | 379 |
| CEP70 | -0.125131216422454 | 0.0147856959570172 | 0.0494219115704211 | 379 | 379 |
| CEP72 | -0.0220789322788973 | 0.66831379681422 | 0.782851418634864 | 379 | 379 |
| CEP76 | 0.353683949827653 | 0.0000000000013093875 | 8.776921583374E-10 | 379 | 379 |
| CEP78 | 0.154356372926477 | 0.00258551137869068 | 0.0128163799041697 | 379 | 379 |
| CEP97 | 0.0915484757761601 | 0.0750577425162365 | 0.169137960974194 | 379 | 379 |
| CEPT1 | 0.0446834694546382 | 0.385693171510391 | 0.545316899936397 | 379 | 379 |
| CER1 | -0.0488455658189451 | 0.342952929365427 | 0.504456282155614 | 379 | 169 |
| CERCAM | 0.179155396635606 | 0.000457192943103063 | 0.00328212225772901 | 379 | 379 |
| CERKL | 0.107316417185603 | 0.0367652760818489 | 0.0991003118747826 | 379 | 379 |
| CERK | -0.105787540950571 | 0.0395479092109753 | 0.10479165359284 | 379 | 379 |
| CES1 | -0.140051042526577 | 0.00631484829991087 | 0.0258166622867284 | 379 | 379 |
| CES2 | -0.0714081503270116 | 0.165335686568507 | 0.301146058541279 | 379 | 379 |
| CES3 | -0.046557721029191 | 0.36605988514384 | 0.527104967511406 | 379 | 379 |
| CES4 | -0.130814457660197 | 0.0107958957983788 | 0.0387160466432003 | 379 | 334 |
| CES7 | 0.0183562959968872 | 0.72168439438346 | 0.822293884141779 | 379 | 75 |
| CES8 | -0.205360631753745 | 0.0000563135422962118 | 0.00064993301318352 | 379 | 378 |
| CETN2 | 0.0184013332427292 | 0.721029932521568 | 0.822067822554059 | 379 | 379 |
| CETN3 | -0.0545122014769966 | 0.289817946827355 | 0.448210767466875 | 379 | 379 |
| CETN4P | 0.0618060014884475 | 0.229980975364236 | 0.380353889358752 | 379 | 233 |
| CETP | 0.138663046550503 | 0.00685851456473175 | 0.0274728538968689 | 379 | 378 |
| CFB | 0.0582084715028707 | 0.258302011195966 | 0.412577510986049 | 379 | 379 |
| CFC1B | -0.00205823022230807 | 0.968143231593659 | 0.982664141082113 | 379 | 135 |
| CFDP1 | -0.0860325059428747 | 0.0944368354483831 | 0.201408365415784 | 379 | 379 |
| CFD | 0.302990426140998 | 1.73099504322364E-09 | 2.30816883357166E-07 | 379 | 379 |
| CFHR1 | -0.00026641070773154 | 0.99587548874538 | 0.997374998531814 | 379 | 120 |
| CFHR2 | -0.0323009788811953 | 0.530712059155409 | 0.677142554633042 | 379 | 14 |
| CFHR3 | 0.0232298899840668 | 0.652130302379252 | 0.771183851349425 | 379 | 263 |
| CFHR4 | 0.110617389941145 | 0.0313195717136184 | 0.0877060867957939 | 379 | 34 |
| CFHR5 | -0.0375827209895295 | 0.465696806935455 | 0.618682498352844 | 379 | 24 |
| CFH | 0.14651738028589 | 0.00425772221637026 | 0.0189542245411292 | 379 | 379 |
| CFI | 0.0249412057802361 | 0.628368201290252 | 0.753783332033585 | 379 | 379 |
| CFL1 | 0.103655809054394 | 0.0437233788308406 | 0.112976754122639 | 379 | 379 |
| CFL2 | 0.0716424149458421 | 0.16394933152628 | 0.299584125472591 | 379 | 379 |
| CFLAR | 0.0833127804399252 | 0.105364319244463 | 0.216988338385876 | 379 | 379 |
| CFLP1 | -0.0906245421250265 | 0.0780572595689079 | 0.174116249604264 | 379 | 285 |
| CFP | 0.126707583383923 | 0.0135664598756288 | 0.0461636805241063 | 379 | 379 |
| CFTR | -0.216944201336925 | 0.000020410938674045 | 0.000298763102954531 | 379 | 379 |
| CG030 | -0.163950973918127 | 0.00136031593252488 | 0.00777468238490506 | 379 | 379 |
| CGA | 0.0871764926748452 | 0.0901196191289438 | 0.19437526466754 | 379 | 128 |
| CGB1 | 0.112297401027368 | 0.0288233490678536 | 0.0822433969373148 | 379 | 32 |
| CGB2 | 0.105184435663862 | 0.0406935461643914 | 0.107054747691064 | 379 | 66 |
| CGB5 | 0.0930230564357607 | 0.070465336091185 | 0.161374238535652 | 379 | 170 |
| CGB7 | 0.184869074523294 | 0.000296469735953198 | 0.00234205557576688 | 379 | 215 |
| CGB8 | 0.112814319692215 | 0.0280904628182973 | 0.0808385626649056 | 379 | 96 |
| CGB | 0.0921970875544326 | 0.0730085810509962 | 0.165574075841148 | 379 | 188 |
| CGGBP1 | -0.266209303361119 | 1.43547406110292E-07 | 0.0000066660050847835 | 379 | 379 |
| CGNL1 | -0.173866568704384 | 0.000674923716558035 | 0.00451345276624375 | 379 | 379 |
| CGN | -0.104325089070874 | 0.0423742399409761 | 0.110257451600836 | 379 | 379 |
| CGREF1 | -0.0667781862534345 | 0.194566967863242 | 0.338155400536703 | 379 | 379 |
| CGRRF1 | 0.0913833016106706 | 0.0755869838058963 | 0.169980544913477 | 379 | 379 |
| CH25H | -0.0947622992966883 | 0.0653464841513405 | 0.152398269554549 | 379 | 379 |
| CHAC1 | 0.207699855196598 | 0.0000460838923700448 | 0.000556486856220005 | 379 | 379 |
| CHAC2 | 0.23466405569914 | 3.87507252852169E-06 | 0.0000836400023337833 | 379 | 379 |
| CHADL | -0.211238216949481 | 0.0000338837972273879 | 0.000439403486870273 | 379 | 379 |
| CHAD | -0.152055968681634 | 0.0030002649701958 | 0.0144237570907274 | 379 | 375 |
| CHAF1A | 0.165274529610114 | 0.00124155080641829 | 0.00725190603812688 | 379 | 379 |
| CHAF1B | 0.193198379364854 | 0.000154041551240874 | 0.00140300224070007 | 379 | 379 |
| CHAT | 0.0382517109660899 | 0.457788504997754 | 0.612113458567365 | 379 | 70 |
| CHCHD10 | -0.0432976269277775 | 0.400614214074564 | 0.560063360126239 | 379 | 379 |
| CHCHD1 | 0.0494737247090282 | 0.336774703155708 | 0.497749600016695 | 379 | 379 |
| CHCHD2 | 0.0464267532052948 | 0.367411282698835 | 0.528206997777878 | 379 | 379 |
| CHCHD3 | -0.0175706844402074 | 0.733132831707417 | 0.830776308781131 | 379 | 379 |
| CHCHD4 | -0.0395688658781594 | 0.442439690065735 | 0.598203773946496 | 379 | 379 |
| CHCHD5 | 0.0484272586810545 | 0.347106929180995 | 0.508844939831424 | 379 | 379 |
| CHCHD6 | -0.0890786362556548 | 0.0832927786146265 | 0.183380992046504 | 379 | 379 |
| CHCHD7 | -0.159135701102678 | 0.0018859122137516 | 0.00999555265722836 | 379 | 379 |
| CHCHD8 | 0.122824313301052 | 0.0167431348362504 | 0.0542367060175091 | 379 | 379 |
| CHD1L | 0.0176374084737874 | 0.732158143583765 | 0.830315240847568 | 379 | 379 |
| CHD1 | -0.0530866029027799 | 0.302635046399366 | 0.46160554905166 | 379 | 379 |
| CHD2 | 0.0214782866439325 | 0.676821393658865 | 0.789459061913523 | 379 | 379 |
| CHD3 | 0.0885838953221759 | 0.0850269415554297 | 0.186371235591539 | 379 | 379 |
| CHD4 | 0.0770966734576685 | 0.134085214516111 | 0.258975417243858 | 379 | 379 |
| CHD5 | 0.0968859154824928 | 0.0595146219219342 | 0.142261111931056 | 379 | 366 |
| CHD6 | -0.219738352680412 | 0.0000158457800963685 | 0.000246230507641688 | 379 | 379 |
| CHD7 | -0.0797520017546378 | 0.121154248960805 | 0.240176609517581 | 379 | 379 |
| CHD8 | 0.0845079135883083 | 0.100445268063132 | 0.210219652797231 | 379 | 379 |
| CHD9 | -0.212643216572575 | 0.0000299457514101574 | 0.000398499569772215 | 379 | 379 |
| CHDH | -0.329027790075039 | 5.09621490207377E-11 | 1.26309686347898E-08 | 379 | 379 |
| CHEK1 | 0.160463167598823 | 0.00172501329148753 | 0.00933758218498899 | 379 | 379 |
| CHEK2 | 0.0404768623272538 | 0.432031799220385 | 0.5887156040822 | 379 | 379 |
| CHERP | 0.0458385199235102 | 0.373519096028974 | 0.534276196512949 | 379 | 379 |
| CHFR | -0.109314372005851 | 0.0333807620906325 | 0.0921701365733269 | 379 | 379 |
| CHGA | 0.0166832690965313 | 0.746136180618385 | 0.83977908777325 | 379 | 347 |
| CHGB | 0.0596623614805604 | 0.246579304201645 | 0.400227115562395 | 379 | 367 |
| CHI3L1 | 0.103627753302165 | 0.0437807139373997 | 0.113090671697598 | 379 | 379 |
| CHI3L2 | 0.117371144562793 | 0.0222935468819748 | 0.0676188610055371 | 379 | 361 |
| CHIA | 0.0466680832278342 | 0.364923509149447 | 0.5259652060344 | 379 | 13 |
| CHIC1 | 0.0268818856934097 | 0.601879161706147 | 0.733636197105151 | 379 | 379 |
| CHIC2 | -0.0018042468135602 | 0.972072610815735 | 0.985323420255863 | 379 | 379 |
| CHID1 | -0.00166062284358133 | 0.974294923326402 | 0.986476519129857 | 379 | 379 |
| CHIT1 | 0.150529567205465 | 0.00330791475560422 | 0.0155535531833342 | 379 | 362 |
| CHKA | -0.0271731331517423 | 0.597947473427177 | 0.730685474122647 | 379 | 379 |
| CHKB-CPT1B | -0.044107363356464 | 0.39185445400755 | 0.551746208923569 | 379 | 379 |
| CHKB | 0.0721858106643047 | 0.16076718341907 | 0.295594557940775 | 379 | 379 |
| CHL1 | 0.0435772939403454 | 0.397575603027947 | 0.557037589179635 | 379 | 376 |
| CHML | -0.125206852477351 | 0.0147250695935091 | 0.0492653125850742 | 379 | 379 |
| CHMP1A | -0.0494146445306149 | 0.337352727490097 | 0.498363126260888 | 379 | 379 |
| CHMP1B | 0.0686090523301782 | 0.182586047528631 | 0.323002904753267 | 379 | 379 |
| CHMP2A | -0.0799843142619209 | 0.120070833573489 | 0.238697061168553 | 379 | 379 |
| CHMP2B | -0.0630024037453895 | 0.221069021318122 | 0.369552776388072 | 379 | 379 |
| CHMP4A | 0.0233392479025444 | 0.650600934982381 | 0.770240866879987 | 379 | 379 |
| CHMP4B | -0.264999303633086 | 1.64232729096262E-07 | 7.31675865790598E-06 | 379 | 379 |
| CHMP4C | -0.179856825243445 | 0.000433817464832687 | 0.00314390814791759 | 379 | 379 |
| CHMP5 | -0.0595589491564765 | 0.247400644538106 | 0.40096942781605 | 379 | 379 |
| CHMP6 | 0.0417215936871906 | 0.417996210849601 | 0.576037591654565 | 379 | 379 |
| CHMP7 | 0.126396237439895 | 0.0138000083484317 | 0.0467577863179603 | 379 | 379 |
| CHM | -0.0306073578046376 | 0.552490292441592 | 0.695711223140842 | 379 | 379 |
| CHN1 | 0.150915705129192 | 0.0032274952133737 | 0.0152325380933603 | 379 | 379 |
| CHN2 | -0.269586485722331 | 9.82385070518887E-08 | 0.0000049690640760838 | 379 | 379 |
| CHODL | -0.056030519660781 | 0.276573190711275 | 0.432967481521511 | 379 | 346 |
| CHORDC1 | 0.257333868374919 | 3.79437594745693E-07 | 0.0000139582349325002 | 379 | 379 |
| CHP2 | -0.145658164479312 | 0.00449068949959142 | 0.0197081432930276 | 379 | 373 |
| CHPF2 | 0.0177291500273336 | 0.730818708390296 | 0.829317996335076 | 379 | 379 |
| CHPF | 0.176275882961241 | 0.000565961619817827 | 0.0038991963161042 | 379 | 379 |
| CHPT1 | -0.143371471012064 | 0.00516777123684714 | 0.0220293559095805 | 379 | 379 |
| CHP | 0.0709661928561813 | 0.167975045364916 | 0.304359791601532 | 379 | 379 |
| CHRAC1 | 0.0749142395700307 | 0.145488482408399 | 0.274800133073943 | 379 | 379 |
| CHRDL1 | 0.0303346833943562 | 0.556036997676012 | 0.698365844677264 | 379 | 371 |
| CHRDL2 | 0.0388499341979514 | 0.450780795196092 | 0.605807347644579 | 379 | 364 |
| CHRD | 0.0821873726565549 | 0.110168605494441 | 0.224227377308948 | 379 | 379 |
| CHRFAM7A | 0.176307458726323 | 0.000564648614213307 | 0.00389257372402173 | 379 | 376 |
| CHRM1 | 0.0582667525660627 | 0.257824811122713 | 0.41217047125221 | 379 | 373 |
| CHRM2 | 0.0039540018439501 | 0.938844515519104 | 0.964891558737841 | 379 | 226 |
| CHRM3 | -0.105064154528025 | 0.0409253477192435 | 0.10750765693921 | 379 | 379 |
| CHRM4 | 0.120454398737731 | 0.0189863898106404 | 0.0598889814135185 | 379 | 349 |
| CHRM5 | 0.0336312985894373 | 0.513915207421756 | 0.66278676793275 | 379 | 310 |
| CHRNA10 | 0.0063597628451396 | 0.901787469122972 | 0.94346992073503 | 379 | 379 |
| CHRNA1 | -0.0768381701774892 | 0.135398815779276 | 0.26062150759464 | 379 | 374 |
| CHRNA2 | -0.084175338121105 | 0.101795441278994 | 0.21217281716387 | 379 | 108 |
| CHRNA3 | 0.0624593675163606 | 0.225083037261499 | 0.374167939772429 | 379 | 379 |
| CHRNA4 | 0.0981350033877622 | 0.0562900148879557 | 0.136473926515456 | 379 | 88 |
| CHRNA5 | 0.161552354556742 | 0.00160249557803568 | 0.00882373849522116 | 379 | 379 |
| CHRNA6 | 0.0624529110319749 | 0.225131072198411 | 0.374199410233375 | 379 | 250 |
| CHRNA7 | 0.168167075402666 | 0.00101446244670379 | 0.00621785514474274 | 379 | 324 |
| CHRNA9 | 0.0017942318004098 | 0.972227567621389 | 0.985323420255863 | 379 | 140 |
| CHRNB1 | 0.132678241633697 | 0.00971309166156207 | 0.0357490995617186 | 379 | 379 |
| CHRNB2 | 0.0947961216132172 | 0.0652500512822252 | 0.152209367035758 | 379 | 368 |
| CHRNB3 | 0.0228411154444844 | 0.657579073875409 | 0.774650836768544 | 379 | 61 |
| CHRNB4 | 0.0478325631766155 | 0.353067201644224 | 0.514259639624012 | 379 | 343 |
| CHRND | 0.0452741168015553 | 0.37943794917559 | 0.539473372741546 | 379 | 59 |
| CHRNE | 0.091227117971298 | 0.0760902046594306 | 0.170745480937632 | 379 | 378 |
| CHRNG | -0.057062138729888 | 0.267812178469786 | 0.423358038324079 | 379 | 252 |
| CHST10 | -0.0879136661759032 | 0.0874224825195817 | 0.190150612483355 | 379 | 379 |
| CHST11 | 0.19829360413908 | 0.000101795623971111 | 0.00101529357751468 | 379 | 379 |
| CHST12 | 0.164407071552683 | 0.00131825892156608 | 0.00757196926327123 | 379 | 379 |
| CHST13 | -0.00650454477668601 | 0.899563299424004 | 0.942137180486962 | 379 | 377 |
| CHST14 | 0.06214696851528 | 0.227415588488682 | 0.37696006424959 | 379 | 379 |
| CHST15 | 0.149762506035184 | 0.00347308275967117 | 0.0161767171620296 | 379 | 379 |
| CHST1 | 0.186061500243212 | 0.000270403153017872 | 0.00219196799592738 | 379 | 379 |
| CHST2 | 0.145528984572244 | 0.00452669302777241 | 0.0198179000562312 | 379 | 379 |
| CHST3 | 0.122453110806631 | 0.0170784115980998 | 0.0550529580827706 | 379 | 379 |
| CHST4 | 0.156579835510826 | 0.00223494995654228 | 0.0113954622492863 | 379 | 342 |
| CHST5 | 0.114917290388097 | 0.0252714736638701 | 0.0743998188280945 | 379 | 376 |
| CHST6 | 0.279942080787335 | 2.97153861041454E-08 | 2.04452899824765E-06 | 379 | 379 |
| CHST7 | 0.115569420005031 | 0.0244483872626653 | 0.0725583928519874 | 379 | 379 |
| CHST8 | 0.0663005824126966 | 0.197784772348806 | 0.342203993635427 | 379 | 223 |
| CHST9 | 0.0751149917142137 | 0.144409621079767 | 0.273220798355879 | 379 | 171 |
| CHSY1 | 0.202946181212979 | 0.0000690960322930382 | 0.000759022785765297 | 379 | 379 |
| CHSY3 | 0.155469139608594 | 0.00240424884562072 | 0.0120993518048141 | 379 | 379 |
| CHTF18 | 0.0187290103719315 | 0.71627442135283 | 0.818814391391043 | 379 | 379 |
| CHTF8 | 0.105866385289045 | 0.0394001743220319 | 0.104525910684673 | 379 | 379 |
| CHUK | 0.0982621137502133 | 0.0559701508185781 | 0.135918695704932 | 379 | 379 |
| CHURC1 | 0.0144514588335745 | 0.779151297810647 | 0.864425466259484 | 379 | 379 |
| CIAO1 | -0.0319153807115577 | 0.535632052402619 | 0.681275348173442 | 379 | 379 |
| CIAPIN1 | 0.01371301464667 | 0.790166361540302 | 0.871614422335908 | 379 | 379 |
| CIB1 | 0.13306685835894 | 0.00949986181355727 | 0.0351227410104817 | 379 | 379 |
| CIB2 | 0.0462251343984365 | 0.369497738154076 | 0.530322197040827 | 379 | 379 |
| CIB3 | 0.0870600947033881 | 0.0905515244903888 | 0.195200655315876 | 379 | 49 |
| CIB4 | 0.110326195327668 | 0.0317705115795441 | 0.0887622521627731 | 379 | 78 |
| CIC | 0.0769337941879451 | 0.134911746939361 | 0.260090434449553 | 379 | 379 |
| CIDEA | 0.0567916693051646 | 0.27009054640846 | 0.425787115415409 | 379 | 77 |
| CIDEB | 0.151393637872292 | 0.00313041686699114 | 0.0148741686169903 | 379 | 379 |
| CIDECP | 0.00808084761596567 | 0.875402292957443 | 0.927278266592542 | 379 | 379 |
| CIDEC | 0.21106787276538 | 0.000034393259437948 | 0.000444556419905888 | 379 | 378 |
| CIITA | 0.196235299654803 | 0.000120491185282706 | 0.00115304016495439 | 379 | 379 |
| CILP2 | -0.000958203759337749 | 0.985166088202555 | 0.99191962610472 | 379 | 379 |
| CILP | 0.117071080493128 | 0.0226405755616172 | 0.0684217204195283 | 379 | 377 |
| CINP | 0.0108264829084505 | 0.833605953202604 | 0.900209075763915 | 379 | 379 |
| CIR1 | -0.210855222258044 | 0.0000350394225502757 | 0.000451437082733507 | 379 | 379 |
| CIRBP | -0.0684015376625589 | 0.183915957488234 | 0.324660047691769 | 379 | 379 |
| CIRH1A | -0.0756677973392375 | 0.141470353925009 | 0.269118008238208 | 379 | 379 |
| CISD1 | -0.0265989846159117 | 0.605709255173803 | 0.735939520132573 | 379 | 379 |
| CISD2 | 0.163342693293853 | 0.00141832473732589 | 0.0080350122547708 | 379 | 379 |
| CISD3 | 0.0635663099376616 | 0.216955107118211 | 0.364514413127648 | 379 | 379 |
| CISH | -0.274644830890173 | 5.51210539626101E-08 | 3.14968374054938E-06 | 379 | 379 |
| CITED1 | 0.291772633226123 | 7.12885671329297E-09 | 6.79571975534486E-07 | 379 | 377 |
| CITED2 | 0.119319887715767 | 0.0201499471995543 | 0.0625879022775214 | 379 | 379 |
| CITED4 | -0.0600192902860865 | 0.243759065627835 | 0.396623564194052 | 379 | 379 |
| CIT | 0.148224367498219 | 0.00382699988339239 | 0.0174440813075642 | 379 | 379 |
| CIZ1 | -0.183841726456315 | 0.000320788670853587 | 0.0024789640387452 | 379 | 379 |
| CKAP2L | 0.117651340881343 | 0.0219736644535599 | 0.0668344560185897 | 379 | 379 |
| CKAP2 | -0.10862955568207 | 0.0345096376816302 | 0.0944584616166985 | 379 | 379 |
| CKAP4 | 0.152958616239605 | 0.00283080375700065 | 0.0137841790014265 | 379 | 379 |
| CKAP5 | 0.0925258472879034 | 0.071987474131899 | 0.163952175176579 | 379 | 379 |
| CKB | -0.0881448834527647 | 0.0865900081189225 | 0.188878622770296 | 379 | 379 |
| CKLF | 0.00349482480148181 | 0.945934935158744 | 0.96950263074163 | 379 | 379 |
| CKMT1A | 0.0274201315662052 | 0.594622257586459 | 0.727833207199476 | 379 | 379 |
| CKMT1B | -0.0305623575112725 | 0.55307485349517 | 0.696100558277404 | 379 | 379 |
| CKMT2 | -0.0470443103408001 | 0.361066098989649 | 0.522303830945266 | 379 | 362 |
| CKM | 0.0182778973752518 | 0.722824133377018 | 0.823308646403925 | 379 | 350 |
| CKS1B | 0.104153173757874 | 0.0427173959626526 | 0.110980152928128 | 379 | 379 |
| CKS2 | 0.0645950861051971 | 0.209591692061751 | 0.356056790096222 | 379 | 379 |
| CLASP1 | 0.0668878495313811 | 0.19383355447914 | 0.337252695525832 | 379 | 379 |
| CLASP2 | -0.219872532427161 | 0.0000156530098035464 | 0.000244191879138252 | 379 | 379 |
| CLCA1 | -0.0272996613004171 | 0.59624303730088 | 0.72905198221521 | 379 | 364 |
| CLCA2 | -0.0132229189744055 | 0.797500184799725 | 0.876348573720292 | 379 | 299 |
| CLCA3P | 0.139526389868406 | 0.0065156354576584 | 0.026446677554647 | 379 | 239 |
| CLCA4 | 0.0495249967475245 | 0.336273586006915 | 0.497287638972636 | 379 | 356 |
| CLCC1 | -0.0838682390030914 | 0.103054928807934 | 0.213920972404075 | 379 | 379 |
| CLCF1 | 0.160547978080075 | 0.00171517387874164 | 0.0092919310567457 | 379 | 379 |
| CLCN1 | 0.130804555933618 | 0.0108019229505102 | 0.0387306561053736 | 379 | 361 |
| CLCN2 | -0.234564389537291 | 0.0000039129219159343 | 0.0000843319736403754 | 379 | 379 |
| CLCN3 | -0.00416702963498867 | 0.935556634553104 | 0.963336119823517 | 379 | 379 |
| CLCN4 | 0.00157298071238971 | 0.975651120260163 | 0.987250607251374 | 379 | 379 |
| CLCN5 | 0.0236690230938413 | 0.645997923670964 | 0.767410390662505 | 379 | 379 |
| CLCN6 | 0.060782792853767 | 0.237802296639827 | 0.389456012040513 | 379 | 379 |
| CLCN7 | -0.124521233074478 | 0.0152826914101605 | 0.0506477027044398 | 379 | 379 |
| CLCNKA | -0.0318288450370774 | 0.536739329883213 | 0.682340670188135 | 379 | 251 |
| CLCNKB | 0.0462096763225321 | 0.369658008527333 | 0.530454158066099 | 379 | 247 |
| CLC | 0.107726414679984 | 0.0360479429261815 | 0.0976579604235997 | 379 | 284 |
| CLDN10 | -0.171654814714336 | 0.000791745436944925 | 0.00511359235301106 | 379 | 253 |
| CLDN11 | 0.0529811410920801 | 0.303597911273568 | 0.46262983282603 | 379 | 377 |
| CLDN12 | 0.0234996467532372 | 0.648360411334846 | 0.768926983428873 | 379 | 379 |
| CLDN14 | 0.124132077600892 | 0.0156073638298563 | 0.051499885175302 | 379 | 377 |
| CLDN15 | -0.087444604207293 | 0.0891310483941857 | 0.192893519707478 | 379 | 379 |
| CLDN16 | -0.0911069408588119 | 0.0764792633605438 | 0.171367480383418 | 379 | 356 |
| CLDN17 | -0.0169387505009347 | 0.742385022317282 | 0.837091901248576 | 379 | 4 |
| CLDN18 | 0.203919430530734 | 0.0000636453938809004 | 0.000714621455168845 | 379 | 334 |
| CLDN19 | 0.145615639229712 | 0.00450251301088083 | 0.0197425537328052 | 379 | 123 |
| CLDN1 | -0.11252143429243 | 0.028503725884022 | 0.0815779267939359 | 379 | 379 |
| CLDN20 | -0.089333045210065 | 0.0824121998153409 | 0.181886587036796 | 379 | 329 |
| CLDN22 | -0.0217367747867034 | 0.673155029999606 | 0.786709768645065 | 379 | 3 |
| CLDN23 | -0.123355634295581 | 0.0162732555770816 | 0.053113763223436 | 379 | 379 |
| CLDN2 | -0.127379135959374 | 0.0130745200811093 | 0.0449214320166756 | 379 | 379 |
| CLDN3 | -0.107261425093003 | 0.0368624054156186 | 0.0993081215463159 | 379 | 379 |
| CLDN4 | -0.0992121364044268 | 0.053626928980065 | 0.131533244410778 | 379 | 379 |
| CLDN5 | 0.0714631259207501 | 0.165009559903113 | 0.300756033340472 | 379 | 379 |
| CLDN6 | 0.178903931342723 | 0.00046585443640294 | 0.00333343982858083 | 379 | 248 |
| CLDN7 | 0.0579831596950329 | 0.260152578473669 | 0.414321712929792 | 379 | 379 |
| CLDN8 | -0.0380819257438327 | 0.459788441352653 | 0.613794197477977 | 379 | 209 |
| CLDN9 | 0.0474875456886549 | 0.356554476406657 | 0.517703731560574 | 379 | 379 |
| CLDND1 | -0.0342102690642727 | 0.506692022781723 | 0.656088906662052 | 379 | 379 |
| CLDND2 | -0.00868302509397554 | 0.866201534630767 | 0.921605710917517 | 379 | 362 |
| CLEC10A | 0.0792268955749768 | 0.12363119950787 | 0.243940633281127 | 379 | 376 |
| CLEC11A | 0.1083138789646 | 0.0350408513377603 | 0.095648403128457 | 379 | 379 |
| CLEC12A | 0.151620258323697 | 0.00308531862869308 | 0.0147233929650364 | 379 | 368 |
| CLEC12B | 0.0278377873100114 | 0.589018799899712 | 0.723834196740718 | 379 | 203 |
| CLEC14A | 0.103512897236546 | 0.0440160950886158 | 0.113498996924854 | 379 | 379 |
| CLEC16A | 0.00463843618817199 | 0.92828489361475 | 0.95944708458055 | 379 | 379 |
| CLEC17A | 0.0712107853942171 | 0.166510477747595 | 0.30278519376186 | 379 | 287 |
| CLEC18A | -0.0285506588192847 | 0.579511092727963 | 0.716257094584462 | 379 | 377 |
| CLEC18B | -0.00403131856872755 | 0.937651082540431 | 0.964402202749853 | 379 | 362 |
| CLEC18C | 0.0479926278122783 | 0.351456664868015 | 0.51273748591255 | 379 | 219 |
| CLEC1A | 0.172832755925832 | 0.000727384810893812 | 0.00478837517609645 | 379 | 379 |
| CLEC1B | 0.0535786001990364 | 0.298169874644137 | 0.456897960061969 | 379 | 11 |
| CLEC2A | -0.102285023303391 | 0.0465996121199443 | 0.11856500822716 | 379 | 47 |
| CLEC2B | 0.205563716490863 | 0.0000553468561958763 | 0.000641389517622346 | 379 | 379 |
| CLEC2D | 0.0748078017328383 | 0.146062977571185 | 0.275717509451776 | 379 | 379 |
| CLEC2L | 0.0519920827775008 | 0.312726585439409 | 0.472509531153806 | 379 | 292 |
| CLEC3A | -0.0116420506638425 | 0.821274886860094 | 0.892825179925541 | 379 | 133 |
| CLEC3B | -0.044575582510566 | 0.386842482635908 | 0.54616296964574 | 379 | 379 |
| CLEC4A | 0.123746428793069 | 0.0159350578709099 | 0.0523113124941062 | 379 | 379 |
| CLEC4C | 0.0819943698411224 | 0.111009571518995 | 0.225567186941829 | 379 | 114 |
| CLEC4D | 0.123653895705886 | 0.0160145758552896 | 0.0525114949658811 | 379 | 289 |
| CLEC4E | 0.187964300087803 | 0.000233197026292295 | 0.00194605666554025 | 379 | 374 |
| CLEC4F | -0.0572317161655806 | 0.266390428578401 | 0.421659740097526 | 379 | 302 |
| CLEC4GP1 | 0.0516887573574503 | 0.315561885556609 | 0.475428589932448 | 379 | 189 |
| CLEC4G | 0.171809289303334 | 0.000783015219931148 | 0.00506861874459796 | 379 | 280 |
| CLEC4M | 0.0931652802782453 | 0.0700348068109907 | 0.160630439496625 | 379 | 103 |
| CLEC5A | 0.191066286127992 | 0.000182629620761073 | 0.00160229208869494 | 379 | 378 |
| CLEC6A | 0.146787823909081 | 0.00418668639348559 | 0.0186967607680253 | 379 | 254 |
| CLEC7A | 0.184682028542344 | 0.000300765238636046 | 0.00236568921645833 | 379 | 379 |
| CLEC9A | 0.118300988695162 | 0.0212472650774907 | 0.0651278780197353 | 379 | 292 |
| CLECL1 | 0.125240610091633 | 0.0146980815600745 | 0.0491870989321783 | 379 | 348 |
| CLGN | -0.139966246706897 | 0.00634691953556476 | 0.0259090235019154 | 379 | 359 |
| CLIC1 | -0.0357064184924307 | 0.488275235175628 | 0.639931347945161 | 379 | 379 |
| CLIC2 | 0.126660077601373 | 0.0136018687672399 | 0.046252418781827 | 379 | 379 |
| CLIC3 | 0.139626922228538 | 0.00647672393446243 | 0.0263180867138358 | 379 | 379 |
| CLIC4 | 0.0631427574878991 | 0.220039917706565 | 0.368305882853771 | 379 | 379 |
| CLIC5 | -0.0331928568940495 | 0.51942045747603 | 0.667254216445399 | 379 | 379 |
| CLIC6 | -0.0210547587029065 | 0.682845150494479 | 0.793730428186454 | 379 | 379 |
| CLINT1 | 0.0834215579916078 | 0.104908870621862 | 0.21638877483285 | 379 | 379 |
| CLIP1 | 0.0958483509854884 | 0.0623078809543927 | 0.146977204451123 | 379 | 379 |
| CLIP2 | 0.0223828725813405 | 0.664024790334801 | 0.779392857559844 | 379 | 379 |
| CLIP3 | 0.070472705845664 | 0.170959232395403 | 0.308301924505325 | 379 | 379 |
| CLIP4 | 0.147306412869694 | 0.00405345990588328 | 0.0182622138181899 | 379 | 379 |
| CLK1 | -0.144565070362728 | 0.0048036721207171 | 0.0207610415838118 | 379 | 379 |
| CLK2P | -0.0721105372498201 | 0.161205193233183 | 0.296125307710539 | 379 | 379 |
| CLK2 | -0.0755677655505535 | 0.141998802320946 | 0.269999257040632 | 379 | 379 |
| CLK3 | 0.0868342961555446 | 0.0913940993329128 | 0.196681628043066 | 379 | 379 |
| CLK4 | -0.224201233907354 | 0.00001050365747796 | 0.000179695013350294 | 379 | 379 |
| CLLU1OS | -0.0186054930619883 | 0.718065727630617 | 0.819866822956344 | 379 | 293 |
| CLLU1 | -0.0686638100766856 | 0.182236305826135 | 0.322565744681361 | 379 | 299 |
| CLMN | -0.132274061146807 | 0.00993935849835414 | 0.0364082025319353 | 379 | 379 |
| CLN3 | -0.131783110868705 | 0.0102204822967661 | 0.0371769809173138 | 379 | 379 |
| CLN5 | -0.232111445039661 | 4.96375083957872E-06 | 0.000100944873484274 | 379 | 379 |
| CLN6 | 0.20884443649899 | 0.0000417438267585046 | 0.000516020322299021 | 379 | 379 |
| CLN8 | -0.0011163420216517 | 0.982718310793878 | 0.990209303100976 | 379 | 379 |
| CLNK | 0.115827760307447 | 0.0241287796689645 | 0.0717817619319173 | 379 | 229 |
| CLNS1A | -0.0584015874380771 | 0.256723130798296 | 0.410839890029751 | 379 | 379 |
| CLOCK | 0.0659332557429038 | 0.200285852440159 | 0.345207569730829 | 379 | 379 |
| CLP1 | -0.014239839839047 | 0.782303523014982 | 0.866189457354439 | 379 | 379 |
| CLPB | 0.112317149167401 | 0.0287950518558711 | 0.0821863089388531 | 379 | 379 |
| CLPP | 0.0769708524592348 | 0.134723350592677 | 0.259853559878561 | 379 | 379 |
| CLPS | 0.0939394119926824 | 0.0677289657542115 | 0.156441761976942 | 379 | 74 |
| CLPTM1L | 0.0763416481274009 | 0.137949672062005 | 0.264318291122272 | 379 | 379 |
| CLPTM1 | 0.171860106471709 | 0.000780162770842326 | 0.00505922880416034 | 379 | 379 |
| CLPX | 0.0988853166540341 | 0.0544236477454091 | 0.133109425901463 | 379 | 379 |
| CLRN1OS | -0.0616918375074596 | 0.230844492198769 | 0.381217888291459 | 379 | 60 |
| CLRN1 | 0.0181376935580802 | 0.724863906817086 | 0.824745307677373 | 379 | 33 |
| CLRN2 | -0.075351464413977 | 0.143146640346271 | 0.271452905966513 | 379 | 10 |
| CLRN3 | -0.216765309416454 | 0.0000207421582237822 | 0.000301742856391162 | 379 | 379 |
| CLSPN | 0.260496152241966 | 2.69466490163911E-07 | 0.0000106646338662076 | 379 | 379 |
| CLSTN1 | 0.089325149435829 | 0.0824394157565298 | 0.181906157981357 | 379 | 379 |
| CLSTN2 | -0.15369769592519 | 0.00269858393549336 | 0.0132615716836813 | 379 | 375 |
| CLSTN3 | -0.0206073345061954 | 0.689230698664779 | 0.7986713981138 | 379 | 379 |
| CLTA | -0.10610077726746 | 0.0389637344422587 | 0.103645415417374 | 379 | 379 |
| CLTB | 0.0255296878349004 | 0.620283189964493 | 0.747158440593886 | 379 | 379 |
| CLTCL1 | -0.0473056357375807 | 0.358401810965233 | 0.519511010148304 | 379 | 379 |
| CLTC | 0.0827452097532127 | 0.107766123039205 | 0.220832997882503 | 379 | 379 |
| CLUAP1 | 0.0192507438317318 | 0.708725245440189 | 0.812665057054596 | 379 | 379 |
| CLUL1 | -0.127169418750694 | 0.0132264316822936 | 0.0452629767684705 | 379 | 317 |
| CLU | 0.011007475231954 | 0.830865785017877 | 0.898228383694153 | 379 | 379 |
| CLVS1 | 0.0831388920650406 | 0.106095629290747 | 0.218056270730904 | 379 | 359 |
| CLVS2 | 0.0717582747624663 | 0.16326691727825 | 0.298769818976651 | 379 | 106 |
| CLYBL | -0.185232926263683 | 0.000288277364936426 | 0.00229096737152684 | 379 | 379 |
| CMA1 | 0.0850826185160682 | 0.0981456598559325 | 0.207002674569028 | 379 | 177 |
| CMAH | 0.0841483293787182 | 0.101905718800919 | 0.212318461097848 | 379 | 378 |
| CMAS | -0.00141116935714701 | 0.978155212219604 | 0.988271851564948 | 379 | 379 |
| CMBL | -0.0647599734286046 | 0.208428481952378 | 0.354796374879182 | 379 | 379 |
| CMC1 | -0.208181888983992 | 0.000044206559481414 | 0.000537417327650201 | 379 | 379 |
| CMIP | -0.0454897298224573 | 0.377170081835494 | 0.537853144470528 | 379 | 379 |
| CMKLR1 | 0.183471666147464 | 0.000329995141960792 | 0.0025370855660328 | 379 | 379 |
| CMPK1 | -0.0985544838964683 | 0.0552401419555951 | 0.134706866891593 | 379 | 379 |
| CMPK2 | 0.118619560394835 | 0.0208987331319306 | 0.0643747212272673 | 379 | 379 |
| CMTM1 | 0.0451756979707641 | 0.380475911973321 | 0.540358532674475 | 379 | 379 |
| CMTM2 | 0.144338526296862 | 0.00487094666770536 | 0.0209776565002741 | 379 | 374 |
| CMTM3 | 0.0617043446502333 | 0.230749778527377 | 0.381180255656155 | 379 | 379 |
| CMTM4 | -0.115108307414563 | 0.0250279429280348 | 0.0738913121466757 | 379 | 379 |
| CMTM5 | 0.0741783482816201 | 0.149495843319301 | 0.280409004004834 | 379 | 166 |
| CMTM6 | 0.0217234444352229 | 0.673343917884804 | 0.786837765430215 | 379 | 379 |
| CMTM7 | 0.0139811522146071 | 0.786161708552161 | 0.86884869055082 | 379 | 379 |
| CMTM8 | -0.28160491645177 | 2.44104599064908E-08 | 1.77293259716447E-06 | 379 | 379 |
| CMYA5 | 0.0591599598178465 | 0.25058744360659 | 0.404625923487913 | 379 | 379 |
| CN5H6.4 | 0.153139311637929 | 0.00279794458066649 | 0.0136442806555473 | 379 | 379 |
| CNBD1 | 0.0331702969289142 | 0.51970454732603 | 0.66744619239462 | 379 | 8 |
| CNBP | -0.064054679118359 | 0.213436837499519 | 0.36029504630857 | 379 | 379 |
| CNDP1 | 0.224237338992589 | 0.0000104684160920661 | 0.000179246765348434 | 379 | 234 |
| CNDP2 | 0.260539031319567 | 2.68210574016386E-07 | 0.0000106361585231938 | 379 | 379 |
| CNFN | 0.0140298721893416 | 0.785434673544538 | 0.868402071223073 | 379 | 379 |
| CNGA1 | -0.141178223610782 | 0.00590211802266391 | 0.024490894291188 | 379 | 379 |
| CNGA2 | 0.00265918060784788 | 0.958849162896464 | 0.977684932732237 | 379 | 3 |
| CNGA3 | 0.13179480498571 | 0.0102137049889247 | 0.0371591454165869 | 379 | 321 |
| CNGA4 | 0.0085640566336301 | 0.868017881002024 | 0.922764404474715 | 379 | 348 |
| CNGB1 | 0.100399970227013 | 0.0508124927519097 | 0.126487001042557 | 379 | 226 |
| CNGB3 | 0.105728363381796 | 0.0396591008394588 | 0.105016112639528 | 379 | 154 |
| CNIH2 | 0.127985858871388 | 0.0126436525860707 | 0.0438046139986575 | 379 | 378 |
| CNIH3 | -0.0233756215956162 | 0.650092574073021 | 0.7698761009807 | 379 | 379 |
| CNIH4 | -0.0512353584481383 | 0.319831237503027 | 0.480207010238529 | 379 | 379 |
| CNIH | 0.113880379791733 | 0.0266293917914101 | 0.0776139321534733 | 379 | 379 |
| CNKSR1 | -0.064235607047272 | 0.212143879071072 | 0.358845660656989 | 379 | 379 |
| CNKSR2 | -0.0395448626958805 | 0.442716750307326 | 0.598336018341876 | 379 | 248 |
| CNKSR3 | 0.0105866341548645 | 0.837240287918653 | 0.902511711908831 | 379 | 379 |
| CNN1 | 0.068140572917075 | 0.185598540739347 | 0.32694099731519 | 379 | 379 |
| CNN2 | -0.0292892157843379 | 0.569737134096752 | 0.708859723410572 | 379 | 379 |
| CNN3 | -0.0343160848063538 | 0.505377643882183 | 0.655199942650446 | 379 | 379 |
| CNNM1 | -0.00599356615043977 | 0.907416447077528 | 0.946587177514435 | 379 | 374 |
| CNNM2 | 0.0761984721476169 | 0.138692042121363 | 0.265332593871169 | 379 | 379 |
| CNNM3 | 0.154953536785077 | 0.00248674582537574 | 0.0124168210087006 | 379 | 379 |
| CNNM4 | 0.132133349865024 | 0.0100192214848517 | 0.0366465824758606 | 379 | 379 |
| CNOT10 | -0.204502446925188 | 0.0000605771482978291 | 0.000689392457358651 | 379 | 379 |
| CNOT1 | -0.0688393280748196 | 0.181118595045037 | 0.321103317467184 | 379 | 379 |
| CNOT2 | 0.0049633130311532 | 0.923276898093405 | 0.956109212962622 | 379 | 379 |
| CNOT3 | 0.216347964761275 | 0.0000215348010190992 | 0.000309863595505587 | 379 | 379 |
| CNOT4 | -0.0590548453718674 | 0.251431741147369 | 0.405534412016772 | 379 | 379 |
| CNOT6L | 0.0589489987987042 | 0.252283914495638 | 0.406326493105314 | 379 | 379 |
| CNOT6 | 0.0629792899007386 | 0.221238825978471 | 0.3696255039153 | 379 | 379 |
| CNOT7 | 0.0501567259681201 | 0.330138507528277 | 0.490887613593602 | 379 | 379 |
| CNOT8 | -0.27418955406521 | 5.80919090910189E-08 | 0.0000032538033148495 | 379 | 379 |
| CNO | -0.0403393362995371 | 0.433599049977951 | 0.590328373058597 | 379 | 379 |
| CNPY1 | 0.108192557602943 | 0.0352468497276828 | 0.0960388259448254 | 379 | 112 |
| CNPY2 | 0.00637678272274133 | 0.901525966100541 | 0.94346992073503 | 379 | 379 |
| CNPY3 | 0.0418822623904429 | 0.41620420983646 | 0.574286504706842 | 379 | 379 |
| CNPY4 | 0.070754375554499 | 0.169251127760042 | 0.305945059825519 | 379 | 379 |
| CNP | 0.155232052277028 | 0.00244186812162271 | 0.01223288511508 | 379 | 379 |
| CNR1 | 0.0696015269267925 | 0.176323621581389 | 0.315337311149616 | 379 | 355 |
| CNR2 | 0.0977730692363093 | 0.0572091084960545 | 0.138065019870955 | 379 | 315 |
| CNRIP1 | 0.126252223114473 | 0.0139092281412749 | 0.0470635111920133 | 379 | 379 |
| CNST | -0.208059534620314 | 0.000044676118566014 | 0.000541796990169373 | 379 | 379 |
| CNTD1 | -0.0257087420043602 | 0.617832190464147 | 0.745156731905299 | 379 | 379 |
| CNTD2 | 0.0748673388679777 | 0.145741414861477 | 0.275189103311434 | 379 | 379 |
| CNTFR | 0.0848948933077879 | 0.0988921663217214 | 0.207977672990894 | 379 | 333 |
| CNTF | 0.193599002304967 | 0.000149161619387252 | 0.00136482537573162 | 379 | 378 |
| CNTLN | -0.11608960910582 | 0.0238085198497807 | 0.070999448275147 | 379 | 379 |
| CNTN1 | 0.0199120417278783 | 0.69919753634409 | 0.805606877252055 | 379 | 326 |
| CNTN2 | 0.0338548115944935 | 0.511120369759375 | 0.660227667204487 | 379 | 304 |
| CNTN3 | -0.264963491867369 | 1.64886705054114E-07 | 7.31675865790598E-06 | 379 | 362 |
| CNTN4 | 0.00613654847723637 | 0.90521803294358 | 0.94521865972116 | 379 | 379 |
| CNTN5 | -0.0116177908587421 | 0.821641067562678 | 0.892854176313355 | 379 | 118 |
| CNTN6 | 0.0573538351877767 | 0.265369783288529 | 0.420502842087824 | 379 | 126 |
| CNTNAP1 | 0.0790368927151807 | 0.124537090010656 | 0.245209376621846 | 379 | 379 |
| CNTNAP2 | 0.0631912683658626 | 0.219685022967672 | 0.367856365390014 | 379 | 379 |
| CNTNAP3 | 0.101841713200986 | 0.0475630691484031 | 0.12043348287541 | 379 | 367 |
| CNTNAP4 | 0.0745824443057566 | 0.147285042360391 | 0.277337874636451 | 379 | 70 |
| CNTNAP5 | 0.0580064497844151 | 0.259960866117802 | 0.414149449894246 | 379 | 107 |
| CNTROB | 0.101243870492738 | 0.0488887414172132 | 0.122766712869871 | 379 | 379 |
| COASY | 0.211911408924269 | 0.0000319394408047416 | 0.000419123251010203 | 379 | 379 |
| COBLL1 | -0.166490888467918 | 0.00114089116631268 | 0.00681989449672832 | 379 | 379 |
| COBL | 0.184696979406647 | 0.00030041976954422 | 0.00236568921645833 | 379 | 379 |
| COBRA1 | 0.0539235739974157 | 0.295065301460849 | 0.453565325166621 | 379 | 379 |
| COCH | -0.083776301338797 | 0.103434378385354 | 0.214394402532386 | 379 | 377 |
| COG1 | -0.0641289463095275 | 0.212905420990367 | 0.359765526452787 | 379 | 379 |
| COG2 | -0.183739202196131 | 0.00032331502555151 | 0.00249346181510515 | 379 | 379 |
| COG3 | -0.217250193880776 | 0.0000198559893060685 | 0.000293153057305083 | 379 | 379 |
| COG4 | -0.0377351102298034 | 0.463888753883225 | 0.617017468261734 | 379 | 379 |
| COG5 | -0.180667459587995 | 0.000408188775426297 | 0.00299983952526042 | 379 | 379 |
| COG6 | -0.230405353591463 | 5.84793344475814E-06 | 0.000113979822937926 | 379 | 379 |
| COG7 | -0.0579035464824635 | 0.260808650459827 | 0.414933316321707 | 379 | 379 |
| COG8 | 0.113643490738686 | 0.0269482801771836 | 0.0783935591774056 | 379 | 379 |
| COIL | -0.0875872604944932 | 0.0886086087503866 | 0.191951436064969 | 379 | 379 |
| COL10A1 | 0.0997716888032155 | 0.0522853847234978 | 0.129049829704265 | 379 | 378 |
| COL11A1 | 0.0833904147119451 | 0.10503910735599 | 0.216594066838743 | 379 | 379 |
| COL11A2 | 0.0411759891029477 | 0.424115238583877 | 0.581761117304815 | 379 | 377 |
| COL12A1 | 0.0942877312376616 | 0.0667119402170081 | 0.154673099932511 | 379 | 379 |
| COL13A1 | 0.210440211996828 | 0.0000363336919348207 | 0.000463892107974002 | 379 | 379 |
| COL14A1 | 0.0720149523393772 | 0.16176268702219 | 0.296956814950096 | 379 | 379 |
| COL15A1 | 0.16682971138612 | 0.00111422132510064 | 0.00671105116467058 | 379 | 379 |
| COL16A1 | 0.109655532039886 | 0.0328302257450639 | 0.0910303057017377 | 379 | 379 |
| COL17A1 | -0.0543514971274681 | 0.291244305338696 | 0.449680118848752 | 379 | 379 |
| COL18A1 | 0.183609026288872 | 0.000326549487009389 | 0.00251547133971335 | 379 | 379 |
| COL19A1 | 0.0317068270747124 | 0.538302570943665 | 0.683880463149615 | 379 | 271 |
| COL1A1 | 0.174437739611041 | 0.000647464266644072 | 0.00436367147485338 | 379 | 379 |
| COL1A2 | 0.151674655121756 | 0.00307458167570723 | 0.0146862937764209 | 379 | 379 |
| COL20A1 | -0.000848418726536032 | 0.98686550283256 | 0.992924807944588 | 379 | 88 |
| COL21A1 | 0.0418953145195518 | 0.41605883293909 | 0.574205786839025 | 379 | 376 |
| COL22A1 | 0.123130596986609 | 0.0164708419926096 | 0.0535910493976801 | 379 | 374 |
| COL23A1 | 0.18814067884682 | 0.000230002226402416 | 0.0019242549135473 | 379 | 379 |
| COL24A1 | 0.096427384153473 | 0.0607360191889992 | 0.144293528454287 | 379 | 376 |
| COL25A1 | -0.0803710980721746 | 0.118283805329309 | 0.23618643424668 | 379 | 261 |
| COL27A1 | -0.0790237309555941 | 0.124600032761844 | 0.245266499513733 | 379 | 379 |
| COL28A1 | 0.0751805428774888 | 0.144058668702898 | 0.27271293517673 | 379 | 378 |
| COL29A1 | 0.125390951976519 | 0.01457841513278 | 0.0488691150046935 | 379 | 265 |
| COL2A1 | -0.191881539006675 | 0.000171157078190506 | 0.00152960470431685 | 379 | 342 |
| COL3A1 | 0.138319144434024 | 0.00699953332831435 | 0.0279530205103357 | 379 | 379 |
| COL4A1 | 0.156967912340153 | 0.00217841100213386 | 0.0111697784717637 | 379 | 379 |
| COL4A2 | 0.154022042178716 | 0.00264235395304101 | 0.0130427169979829 | 379 | 379 |
| COL4A3BP | 0.0310706196908695 | 0.546490062829094 | 0.690611425471427 | 379 | 379 |
| COL4A3 | -0.0648402785820339 | 0.207863653955176 | 0.354295584167732 | 379 | 332 |
| COL4A4 | -0.0164751562182633 | 0.749196280564673 | 0.842171543230134 | 379 | 379 |
| COL4A5 | -0.0914631892544795 | 0.0753306349806166 | 0.169637232299337 | 379 | 379 |
| COL4A6 | -0.07478164911165 | 0.146204399883618 | 0.275879410058277 | 379 | 361 |
| COL5A1 | 0.144907226267874 | 0.00470365225145401 | 0.0204302337002914 | 379 | 379 |
| COL5A2 | 0.146362019061358 | 0.004299020654235 | 0.0190866505893801 | 379 | 379 |
| COL5A3 | 0.224608990280993 | 0.0000101121326319809 | 0.000174795082006428 | 379 | 379 |
| COL6A1 | 0.1639799993349 | 0.00135760318556197 | 0.00776422150658283 | 379 | 379 |
| COL6A2 | 0.165529559237231 | 0.00121979263143082 | 0.0071641138317566 | 379 | 379 |
| COL6A3 | 0.15102327923954 | 0.00320540937464409 | 0.0151533597321696 | 379 | 379 |
| COL6A4P2 | -0.0370900993240003 | 0.471568210534068 | 0.624511591088903 | 379 | 345 |
| COL6A6 | 0.0960106707035463 | 0.0618638983140422 | 0.1462764194966 | 379 | 323 |
| COL7A1 | 0.121077543856708 | 0.0183725343458517 | 0.0583442610718501 | 379 | 379 |
| COL8A1 | 0.104446319814914 | 0.0421336529782207 | 0.109808894749232 | 379 | 379 |
| COL8A2 | 0.0388198264555555 | 0.451132031518451 | 0.606156123937646 | 379 | 379 |
| COL9A1 | -0.194029474760474 | 0.000144079873582153 | 0.00132566855377584 | 379 | 368 |
| COL9A2 | -0.0502347873965269 | 0.329385450940598 | 0.490061883488421 | 379 | 379 |
| COL9A3 | -0.266396170125008 | 1.40585507571576E-07 | 6.57436189653113E-06 | 379 | 379 |
| COLEC10 | -0.0253921425117209 | 0.622168850671917 | 0.748869323607701 | 379 | 350 |
| COLEC11 | -0.0222925167485565 | 0.665298696103559 | 0.780379942400696 | 379 | 377 |
| COLEC12 | 0.145399093766001 | 0.00456315708955319 | 0.0199555092129821 | 379 | 379 |
| COLQ | -0.0881889107631115 | 0.0864322171731965 | 0.188700506728709 | 379 | 379 |
| COMMD10 | -0.0907417835303034 | 0.0776713378275254 | 0.173391948485045 | 379 | 379 |
| COMMD1 | 0.0769729334158897 | 0.134712777462127 | 0.259853559878561 | 379 | 379 |
| COMMD2 | -0.129162089400328 | 0.0118438736315115 | 0.0416088459187829 | 379 | 379 |
| COMMD3 | -0.104709361015423 | 0.041615594473999 | 0.108945604334625 | 379 | 379 |
| COMMD4 | 0.0870283117541663 | 0.0906697458651165 | 0.195370541296841 | 379 | 379 |
| COMMD5 | -0.0521148636058965 | 0.311583671690218 | 0.471141694675028 | 379 | 379 |
| COMMD6 | -0.255037379247322 | 4.85131349274854E-07 | 0.0000170251051211005 | 379 | 379 |
| COMMD7 | -0.332918809402479 | 2.9212640738294E-11 | 7.93463343231361E-09 | 379 | 379 |
| COMMD8 | -0.0134192610782209 | 0.794559931487224 | 0.874231969453897 | 379 | 379 |
| COMMD9 | 0.065795810333036 | 0.20122758849804 | 0.346265863911635 | 379 | 379 |
| COMP | 0.0366376171156737 | 0.476996827522988 | 0.629100245851124 | 379 | 369 |
| COMTD1 | 0.0225426049539604 | 0.661775126872467 | 0.777673040693847 | 379 | 379 |
| COMT | 0.0393289341504733 | 0.445213575841536 | 0.60053504215988 | 379 | 379 |
| COPA | -0.111146546219882 | 0.030514120756962 | 0.0860156363902533 | 379 | 379 |
| COPB1 | 0.027496504233242 | 0.593595796585292 | 0.727025601006435 | 379 | 379 |
| COPB2 | 0.0356455276193522 | 0.489017684081867 | 0.640568089863962 | 379 | 379 |
| COPE | 0.103353883982403 | 0.0443437257492315 | 0.11408426030307 | 379 | 379 |
| COPG2 | -0.163625519584907 | 0.00139107609241083 | 0.00791082138666244 | 379 | 379 |
| COPG | 0.0249775435182279 | 0.627867664268356 | 0.753319620423149 | 379 | 379 |
| COPS2 | 0.0348260786374137 | 0.499067977854234 | 0.649610704713041 | 379 | 379 |
| COPS3 | 0.175850440739542 | 0.00058393101842079 | 0.00399937279214074 | 379 | 379 |
| COPS4 | 0.0708417669900834 | 0.16872376774672 | 0.305269148135948 | 379 | 379 |
| COPS5 | -0.104140257280809 | 0.0427432724857889 | 0.111030385921192 | 379 | 379 |
| COPS6 | -0.040768058963413 | 0.428724122790051 | 0.585854025270925 | 379 | 379 |
| COPS7A | 0.113452369282556 | 0.027207946530919 | 0.0789520216325279 | 379 | 379 |
| COPS7B | 0.0587239813488766 | 0.254102192868788 | 0.408326305227517 | 379 | 379 |
| COPS8 | 0.032820399049955 | 0.524120889982746 | 0.671335207143274 | 379 | 379 |
| COPZ1 | 0.0982628960162009 | 0.0559681869804595 | 0.135918695704932 | 379 | 379 |
| COPZ2 | 0.122515211795009 | 0.0170219154695373 | 0.0549306219210111 | 379 | 379 |
| COQ10A | 0.252069400948767 | 6.64151780884782E-07 | 0.0000216592130121438 | 379 | 379 |
| COQ10B | 0.0738588507686914 | 0.151261589599556 | 0.282682775789264 | 379 | 379 |
| COQ2 | 0.0953025796142664 | 0.0638199867182721 | 0.149630211262847 | 379 | 379 |
| COQ3 | 0.048318164232377 | 0.348195513281524 | 0.509860470965665 | 379 | 379 |
| COQ4 | 0.0815750165359542 | 0.112854203309051 | 0.228240834680932 | 379 | 379 |
| COQ5 | 0.0558521369907694 | 0.278107615232403 | 0.434848812777233 | 379 | 379 |
| COQ6 | 0.0575298747266545 | 0.263903208079378 | 0.418814855914672 | 379 | 379 |
| COQ7 | -0.0732470634445526 | 0.154686816456434 | 0.28751870801958 | 379 | 379 |
| COQ9 | -0.1424149490653 | 0.00547727853691449 | 0.0230482764919229 | 379 | 379 |
| CORIN | -0.0263185033973424 | 0.609517279517922 | 0.738497318562869 | 379 | 377 |
| CORO1A | 0.168978756424291 | 0.000958001161598743 | 0.00594530423542406 | 379 | 379 |
| CORO1B | 0.0363934570424057 | 0.479940211703235 | 0.631805505089082 | 379 | 379 |
| CORO1C | 0.225997021934684 | 8.88082988718862E-06 | 0.000159615849311092 | 379 | 379 |
| CORO2A | -0.0651601973522257 | 0.20562447482455 | 0.351536651734883 | 379 | 379 |
| CORO2B | 0.00724764726412844 | 0.888160339217545 | 0.935130947056256 | 379 | 379 |
| CORO6 | 0.185962115487888 | 0.000272491140879098 | 0.00220528748626562 | 379 | 374 |
| CORO7 | -0.0659416469426284 | 0.200228462348909 | 0.345207569730829 | 379 | 379 |
| CORT | 0.186884962240538 | 0.000253668696793584 | 0.00208616462879435 | 379 | 358 |
| COTL1 | 0.294938273010207 | 4.81084707488185E-09 | 4.9942133927098E-07 | 379 | 379 |
| COX10 | 0.0238216483338878 | 0.643872123987644 | 0.765986468736246 | 379 | 379 |
| COX11 | -0.027702337859128 | 0.590833390340129 | 0.725120355512756 | 379 | 379 |
| COX15 | 0.0642137456214784 | 0.212299806493247 | 0.359071593524869 | 379 | 379 |
| COX16 | -0.00582208003021066 | 0.910054049686257 | 0.948180773111815 | 379 | 379 |
| COX17 | -0.0113505613715086 | 0.825677209426827 | 0.89507958746493 | 379 | 379 |
| COX18 | -0.0863785613096111 | 0.0931138043137111 | 0.199358655861383 | 379 | 379 |
| COX19 | -0.0532087626606539 | 0.30152226091574 | 0.460635132863649 | 379 | 379 |
| COX4I1 | -0.159993088732018 | 0.00178049386356824 | 0.0095570201209613 | 379 | 379 |
| COX4I2 | 0.0738276591448624 | 0.151434818720164 | 0.282929485488367 | 379 | 377 |
| COX4NB | 0.0249131165391303 | 0.628755235421979 | 0.75398463498897 | 379 | 379 |
| COX5A | -0.0579700128255299 | 0.260260839945796 | 0.414388335718607 | 379 | 379 |
| COX5B | -0.0897046266368293 | 0.0811395791322673 | 0.179677861853317 | 379 | 379 |
| COX6A1 | 0.0470072943514089 | 0.361444485629438 | 0.522625331057277 | 379 | 379 |
| COX6A2 | 0.0747444044830203 | 0.146405983196219 | 0.276064899323943 | 379 | 35 |
| COX6B1 | -0.0195731602756657 | 0.704074220292705 | 0.809156879381195 | 379 | 379 |
| COX6B2 | 0.116001104482462 | 0.0239163540113483 | 0.0712567193594312 | 379 | 379 |
| COX6C | -0.0686799795676988 | 0.182133124812572 | 0.322469470379826 | 379 | 379 |
| COX7A1 | 0.102653731651756 | 0.0458108012359407 | 0.117129151116213 | 379 | 379 |
| COX7A2L | 0.0068536471463563 | 0.894203580467789 | 0.939003738667266 | 379 | 379 |
| COX7A2 | -0.00374490531620657 | 0.942072731651236 | 0.967043804264895 | 379 | 379 |
| COX7B2 | 0.0931229703570303 | 0.0701626589419927 | 0.160854225689725 | 379 | 24 |
| COX7B | -0.0135173540965733 | 0.793092058827423 | 0.873446339194812 | 379 | 379 |
| COX7C | -0.0823339972197891 | 0.109533079563392 | 0.223452570410826 | 379 | 379 |
| COX8A | 0.106291891209784 | 0.0386109122530075 | 0.102972560805236 | 379 | 379 |
| COX8C | -0.0239196119990611 | 0.642509189480239 | 0.76500763880467 | 379 | 64 |
| CP110 | 0.0275318408608308 | 0.593121140120254 | 0.726650682992315 | 379 | 379 |
| CPA1 | 0.0265206253426272 | 0.606772051575839 | 0.736476263384289 | 379 | 77 |
| CPA2 | 0.0657952156817763 | 0.201231669853428 | 0.346265863911635 | 379 | 296 |
| CPA3 | 0.0987068652197605 | 0.0548628107277976 | 0.133967957033346 | 379 | 377 |
| CPA4 | -0.0460447317385025 | 0.37137084367475 | 0.531817209907766 | 379 | 347 |
| CPA5 | 0.148614753343389 | 0.00373421054700641 | 0.0171390388783157 | 379 | 155 |
| CPA6 | -0.120830529607808 | 0.0186137647973965 | 0.0589574646010827 | 379 | 353 |
| CPAMD8 | -0.00116689310855387 | 0.981935877906679 | 0.989672372649501 | 379 | 379 |
| CPB1 | 0.00698243576135808 | 0.892227508218154 | 0.937724379802243 | 379 | 151 |
| CPB2 | 0.0438151495165989 | 0.395002200229684 | 0.554674381238692 | 379 | 181 |
| CPD | 0.0958046647384078 | 0.0624278204785309 | 0.14721917512468 | 379 | 379 |
| CPEB1 | 0.066292453123234 | 0.197839876218569 | 0.342241237625353 | 379 | 300 |
| CPEB2 | -0.153165897753483 | 0.00279313932507438 | 0.0136309048824944 | 379 | 379 |
| CPEB3 | -0.0414992272769728 | 0.420483803116414 | 0.578460615291213 | 379 | 379 |
| CPEB4 | -0.0245475341915324 | 0.633801734538153 | 0.758191299693665 | 379 | 379 |
| CPE | -0.0700287509077832 | 0.173677525697298 | 0.311814376994388 | 379 | 379 |
| CPLX1 | 0.107720335758683 | 0.0360584911912644 | 0.0976598502035775 | 379 | 379 |
| CPLX2 | -0.129191253902298 | 0.0118246241810269 | 0.0415694305456883 | 379 | 374 |
| CPLX3 | 0.099553749532463 | 0.0528045397051078 | 0.130046546093621 | 379 | 204 |
| CPLX4 | 0.0637207039969093 | 0.215838370259729 | 0.363204888866156 | 379 | 81 |
| CPM | 0.0484108222568919 | 0.347270799641115 | 0.508995078007394 | 379 | 379 |
| CPN1 | -0.158284250594792 | 0.00199622267907659 | 0.0104243095287676 | 379 | 252 |
| CPN2 | -0.085038724121843 | 0.0983198070866704 | 0.207259742176749 | 379 | 276 |
| CPNE1 | -0.250116508319803 | 8.14880757143052E-07 | 0.0000249343451429513 | 379 | 379 |
| CPNE2 | -0.23665327553369 | 3.18887335192082E-06 | 0.0000720147845351777 | 379 | 379 |
| CPNE3 | -0.151547368710594 | 0.0030997591193834 | 0.0147714515632311 | 379 | 379 |
| CPNE4 | 0.21140749557449 | 0.000033384600190056 | 0.000434921059506196 | 379 | 229 |
| CPNE5 | 0.153919777364462 | 0.00265996677858592 | 0.0131068144348414 | 379 | 379 |
| CPNE6 | -0.101582820895615 | 0.0481334019673684 | 0.121438999135893 | 379 | 318 |
| CPNE7 | -0.182860854184111 | 0.000345731618000211 | 0.00263154185094364 | 379 | 379 |
| CPNE8 | 0.0768339091973469 | 0.135420550882305 | 0.26062150759464 | 379 | 379 |
| CPNE9 | -0.147742617688774 | 0.00394437902575532 | 0.0178682082071456 | 379 | 355 |
| CPOX | 0.00743893175631293 | 0.885228696042667 | 0.933244012814559 | 379 | 379 |
| CPO | -0.00181104425316287 | 0.971967438358704 | 0.985323420255863 | 379 | 229 |
| CPPED1 | 0.0831660555857941 | 0.105981126101198 | 0.21789649194676 | 379 | 379 |
| CPS1 | 0.144576375292475 | 0.00480033705122138 | 0.020754706291238 | 379 | 376 |
| CPSF1 | -0.0579233545909514 | 0.260645310517294 | 0.414706725801389 | 379 | 379 |
| CPSF2 | 0.0537939507425074 | 0.296229299887735 | 0.454649065211295 | 379 | 379 |
| CPSF3L | 0.143217397750336 | 0.00521653707584111 | 0.0221912673545972 | 379 | 379 |
| CPSF3 | -0.00897673225915493 | 0.861720403848434 | 0.918612482124018 | 379 | 379 |
| CPSF4L | -0.0121319638395477 | 0.813888478049237 | 0.887325053211649 | 379 | 205 |
| CPSF4 | 0.111269803195712 | 0.0303290716559108 | 0.0856483876634667 | 379 | 379 |
| CPSF6 | 0.0488892361252163 | 0.342521093004003 | 0.504008032065556 | 379 | 379 |
| CPSF7 | 0.164538872114373 | 0.00130633002165007 | 0.00752306467304024 | 379 | 379 |
| CPT1A | -0.00449968643909743 | 0.930424603233141 | 0.96033560015232 | 379 | 379 |
| CPT1B | -0.0203755807559502 | 0.692546965682339 | 0.800891480977675 | 379 | 379 |
| CPT1C | 0.134959155241452 | 0.00851987673595555 | 0.0323577359971765 | 379 | 379 |
| CPT2 | 0.0171753338591603 | 0.738916762503053 | 0.834590804153263 | 379 | 379 |
| CPVL | 0.142898811821587 | 0.00531868855813763 | 0.0224955112480275 | 379 | 379 |
| CPXCR1 | 0.0150555859606979 | 0.770172416071319 | 0.857586699956676 | 379 | 30 |
| CPXM1 | 0.0848172563877068 | 0.0992022131623649 | 0.208366682477052 | 379 | 379 |
| CPXM2 | 0.0988724269272588 | 0.0544552707527084 | 0.133169598974433 | 379 | 379 |
| CPZ | 0.0799774097695964 | 0.120102924126204 | 0.238706378871348 | 379 | 379 |
| CP | 0.13697323891069 | 0.00757669415727173 | 0.0296505708647402 | 379 | 369 |
| CR1L | 0.141988266983953 | 0.00562064073793066 | 0.0235665181965932 | 379 | 206 |
| CR1 | 0.171036777641553 | 0.000827581987098615 | 0.00529332117425527 | 379 | 376 |
| CR2 | 0.0940249016611965 | 0.0674781833860912 | 0.156048217888899 | 379 | 362 |
| CRABP1 | -0.0745059768165622 | 0.147701473403493 | 0.277910876318511 | 379 | 315 |
| CRABP2 | 0.142806014796856 | 0.00534877955177226 | 0.0225938647108096 | 379 | 379 |
| CRADD | 0.0807312735580847 | 0.116638459627579 | 0.233775295707338 | 379 | 379 |
| CRAMP1L | -0.216183621090987 | 0.0000218547434731994 | 0.000313103940452744 | 379 | 379 |
| CRAT | -0.101838158624441 | 0.0475708613012009 | 0.12043348287541 | 379 | 379 |
| CRB1 | -0.00194392552377798 | 0.969911550802465 | 0.983908101325076 | 379 | 185 |
| CRB2 | -0.309998553580915 | 6.92968051273997E-10 | 1.08190319060321E-07 | 379 | 378 |
| CRB3 | -0.0511937590082501 | 0.320224827259168 | 0.480652375086661 | 379 | 379 |
| CRBN | -0.353593145143048 | 1.32795868217279E-12 | 8.776921583374E-10 | 379 | 379 |
| CRCP | -0.163376554720324 | 0.00141503675629133 | 0.00802096878323172 | 379 | 379 |
| CRCT1 | 0.0797164172856083 | 0.121320871753027 | 0.240482879648008 | 379 | 67 |
| CREB1 | 0.0202505954537648 | 0.694337879975815 | 0.801762357075998 | 379 | 379 |
| CREB3L1 | 0.120099812640557 | 0.0193436029296277 | 0.0607458013778116 | 379 | 379 |
| CREB3L2 | 0.172788403250366 | 0.000729717739099223 | 0.00480054523253463 | 379 | 379 |
| CREB3L3 | 0.010756169640259 | 0.834671019576097 | 0.900819561079624 | 379 | 371 |
| CREB3L4 | -0.191760993708956 | 0.000172809809564409 | 0.00153929600361326 | 379 | 379 |
| CREB3 | -0.0152537110547496 | 0.767234372042662 | 0.855608724907869 | 379 | 379 |
| CREB5 | 0.0235429809789493 | 0.647755641862148 | 0.768577515818483 | 379 | 379 |
| CREBBP | -0.0397377726035031 | 0.44049285514569 | 0.59668204521452 | 379 | 379 |
| CREBL2 | -0.0461439071952611 | 0.370340388234962 | 0.53091672459857 | 379 | 379 |
| CREBZF | -0.0596513757921654 | 0.246666466300827 | 0.40023753631856 | 379 | 379 |
| CREG1 | -0.15907947694379 | 0.00189302074837179 | 0.0100226476365062 | 379 | 379 |
| CREG2 | 0.282621726633215 | 2.16308529055465E-08 | 1.62297535016937E-06 | 379 | 310 |
| CRELD1 | -0.122306454082665 | 0.0172124828420645 | 0.0553950835566392 | 379 | 379 |
| CRELD2 | 0.212193529884841 | 0.0000311564126595364 | 0.000411024185105315 | 379 | 379 |
| CREM | 0.131862950344209 | 0.0101742906591349 | 0.0370906113604203 | 379 | 379 |
| CRHBP | 0.0900264714192204 | 0.0800501841381912 | 0.177682195353415 | 379 | 309 |
| CRHR1 | 0.0847494093853528 | 0.0994737956625324 | 0.208738112011503 | 379 | 113 |
| CRHR2 | -0.15830561081254 | 0.00199338469860008 | 0.0104208597372805 | 379 | 223 |
| CRH | 0.0303629769617382 | 0.555668466302599 | 0.698079854897544 | 379 | 13 |
| CRIM1 | -0.107440097832027 | 0.0365476192230592 | 0.0986880285925122 | 379 | 379 |
| CRIP1 | 0.232058952727173 | 4.98894224410603E-06 | 0.000101171398258674 | 379 | 379 |
| CRIP2 | 0.179438229382966 | 0.000447629985740796 | 0.00322514802226326 | 379 | 379 |
| CRIP3 | 0.0744900974254836 | 0.147788062575142 | 0.27804741481544 | 379 | 318 |
| CRIPAK | -0.022138783528305 | 0.667468356420797 | 0.782230780253654 | 379 | 379 |
| CRIPT | -0.121810865488053 | 0.0176723695856337 | 0.0565264952643885 | 379 | 379 |
| CRISP1 | 0.0510157893508107 | 0.321912232430873 | 0.482381782394146 | 379 | 4 |
| CRISP2 | 0.0379536161734924 | 0.461303053268434 | 0.614988027984032 | 379 | 28 |
| CRISP3 | -0.00501455446016477 | 0.922487279840375 | 0.955818948490246 | 379 | 79 |
| CRISPLD1 | 0.00195643199769559 | 0.969718065523426 | 0.983864814678605 | 379 | 378 |
| CRISPLD2 | 0.121390094365162 | 0.0180712117639566 | 0.0575978117434063 | 379 | 379 |
| CRKL | 0.0848978408584863 | 0.0988804103312463 | 0.207977672990894 | 379 | 379 |
| CRK | 0.105223185115229 | 0.040619106054265 | 0.106958251639305 | 379 | 379 |
| CRLF1 | 0.0839039647653173 | 0.102907777496191 | 0.213727392080703 | 379 | 358 |
| CRLF2 | 0.0403104732171563 | 0.43392838974098 | 0.590604895097758 | 379 | 174 |
| CRLF3 | 0.218903233661502 | 0.0000170971793037478 | 0.000261576289532956 | 379 | 379 |
| CRLS1 | -0.0381738269241673 | 0.458705313491237 | 0.613110087513113 | 379 | 379 |
| CRMP1 | 0.0451342449044186 | 0.380913611440979 | 0.540685006629795 | 379 | 379 |
| CRNKL1 | 0.0174013744990178 | 0.735607990236945 | 0.83232339822062 | 379 | 379 |
| CRNN | 0.0232255986061852 | 0.652190346951564 | 0.771202380962247 | 379 | 18 |
| CROCCL1 | -0.0050627828626015 | 0.921744161102225 | 0.955282283493542 | 379 | 379 |
| CROCCL2 | -0.0183742255719656 | 0.721423825174617 | 0.82209052829106 | 379 | 379 |
| CROCC | 0.0602004741952865 | 0.242336127116895 | 0.394729378828045 | 379 | 379 |
| CROT | -0.144025104509211 | 0.0049654201726513 | 0.0213221908114127 | 379 | 379 |
| CRP | 0.0241074153521001 | 0.639899699016492 | 0.763000254501113 | 379 | 137 |
| CRTAC1 | 0.0656112819084334 | 0.202496976952468 | 0.347809256671304 | 379 | 331 |
| CRTAM | 0.246027491106576 | 1.24367991516105E-06 | 0.0000351050636811481 | 379 | 359 |
| CRTAP | -0.20742078103145 | 0.0000472049362711179 | 0.000567948711397891 | 379 | 379 |
| CRTC1 | -0.0830229841262704 | 0.106585317807226 | 0.218889040029174 | 379 | 379 |
| CRTC2 | -0.0466475653006499 | 0.365134611600765 | 0.526192970333597 | 379 | 379 |
| CRTC3 | 0.00495450138657564 | 0.923412691054455 | 0.956109212962622 | 379 | 379 |
| CRX | -0.0142212853948881 | 0.782580076206902 | 0.86631296067627 | 379 | 25 |
| CRY1 | 0.15838489913436 | 0.00198288244798667 | 0.0103819892206706 | 379 | 379 |
| CRY2 | -0.0862683450634105 | 0.093533563000022 | 0.199955092955734 | 379 | 379 |
| CRYAA | -0.0994579210033859 | 0.0530341680604233 | 0.130515264279766 | 379 | 132 |
| CRYAB | 0.0554609310155878 | 0.281492895362117 | 0.438723559915112 | 379 | 379 |
| CRYBA1 | 0.0104306648085685 | 0.839605493319712 | 0.904225610859988 | 379 | 78 |
| CRYBA2 | 0.180654479006599 | 0.000408587824502631 | 0.00300165964588298 | 379 | 301 |
| CRYBA4 | -0.0518227189936577 | 0.314307627340053 | 0.474067521291539 | 379 | 313 |
| CRYBB1 | 0.000466630386603106 | 0.99277579852861 | 0.995990615929229 | 379 | 341 |
| CRYBB2 | -0.0128673780512052 | 0.802831727553065 | 0.879623556054715 | 379 | 327 |
| CRYBB3 | 0.0809839597032312 | 0.115494871052346 | 0.232066508231244 | 379 | 348 |
| CRYBG3 | 0.0419961827097321 | 0.414936352690463 | 0.573335052344704 | 379 | 379 |
| CRYGA | -0.0483196224732846 | 0.348180948185296 | 0.509860470965665 | 379 | 7 |
| CRYGB | -0.0863723541195512 | 0.0931374043212604 | 0.199387654165618 | 379 | 7 |
| CRYGC | -0.104215381537445 | 0.0425929552717548 | 0.110714881637173 | 379 | 22 |
| CRYGD | 0.0412518807348324 | 0.423260993291563 | 0.580899803218364 | 379 | 11 |
| CRYGN | 0.0866751135219469 | 0.091991858295775 | 0.19753244166002 | 379 | 174 |
| CRYGS | -0.0851921446824748 | 0.0977121942780899 | 0.206387916245929 | 379 | 379 |
| CRYL1 | -0.208136653019411 | 0.0000443796155858003 | 0.000538860390591089 | 379 | 379 |
| CRYM | -0.0492398033694807 | 0.339067043975089 | 0.50027443860294 | 379 | 379 |
| CRYZL1 | -0.0432400955219712 | 0.401241017908453 | 0.560715799448167 | 379 | 379 |
| CRYZ | 0.148371901914446 | 0.00379169046789321 | 0.0173269995168153 | 379 | 379 |
| CSAD | -0.0853268655743656 | 0.0971811082923569 | 0.2054929098028 | 379 | 379 |
| CSAG1 | 0.234866930511664 | 3.79910551136885E-06 | 0.0000824164814873322 | 379 | 136 |
| CSAG2 | 0.202742472543983 | 0.0000702912380485343 | 0.000768622245799451 | 379 | 42 |
| CSAG3 | 0.240926681059649 | 2.08585297781969E-06 | 0.000051697866055261 | 379 | 145 |
| CSDAP1 | 0.0686293288433504 | 0.182456482224304 | 0.322862315430982 | 379 | 379 |
| CSDA | 0.118478745817052 | 0.0210521739236149 | 0.0646865805915753 | 379 | 379 |
| CSDC2 | 0.0752253370514371 | 0.143819221385088 | 0.272408846612867 | 379 | 377 |
| CSDE1 | -0.0160161153744299 | 0.755959894785 | 0.847516272407384 | 379 | 379 |
| CSE1L | -0.13942668486639 | 0.00655443347151046 | 0.026544384573756 | 379 | 379 |
| CSF1R | 0.166498765987746 | 0.00114026448516046 | 0.00681820392393295 | 379 | 379 |
| CSF1 | 0.19453902201197 | 0.000138274577435883 | 0.00128537661575185 | 379 | 379 |
| CSF2RA | 0.0581291373605464 | 0.258952571584422 | 0.413074142347218 | 379 | 379 |
| CSF2RB | 0.148363643644146 | 0.00379365910212753 | 0.0173300728524826 | 379 | 379 |
| CSF2 | 0.0883644115858965 | 0.0858055311353298 | 0.187725043732905 | 379 | 351 |
| CSF3R | 0.207713310262332 | 0.0000460304837577433 | 0.000556180641041155 | 379 | 379 |
| CSF3 | 0.00368842446150434 | 0.942944897605828 | 0.967738686838942 | 379 | 366 |
| CSGALNACT1 | 0.0216281635614105 | 0.674694621995896 | 0.787812553143785 | 379 | 379 |
| CSGALNACT2 | 0.0833033549977314 | 0.105403856769279 | 0.217024680376039 | 379 | 379 |
| CSH2 | 0.154924652192323 | 0.00249144280842033 | 0.0124277554730461 | 379 | 12 |
| CSK | 0.184100863875389 | 0.0003144849383766 | 0.00244150640490651 | 379 | 379 |
| CSMD1 | 0.0905719275404146 | 0.0782309549593172 | 0.174444823991604 | 379 | 227 |
| CSMD2 | 0.00895908871842352 | 0.86198947016036 | 0.918800516844405 | 379 | 379 |
| CSMD3 | 0.0422381462349192 | 0.412251015609465 | 0.570738244484323 | 379 | 191 |
| CSN1S1 | 0.0789161537337983 | 0.125115420151576 | 0.245987957438319 | 379 | 82 |
| CSN2 | -0.0521913912118379 | 0.310872698703192 | 0.470389489460233 | 379 | 55 |
| CSN3 | 0.0830896141473108 | 0.10630360133851 | 0.218378347217154 | 379 | 15 |
| CSNK1A1L | -0.0380047471923575 | 0.460699151972426 | 0.614434002246216 | 379 | 369 |
| CSNK1A1P | -0.0290588078859175 | 0.572777901648886 | 0.71105936851328 | 379 | 185 |
| CSNK1A1 | -0.0573113876826194 | 0.265724246049293 | 0.420829101490845 | 379 | 379 |
| CSNK1D | -0.045157660390424 | 0.380666331443416 | 0.540522201364943 | 379 | 379 |
| CSNK1E | -0.0364637536853244 | 0.479091765859963 | 0.630981835501252 | 379 | 379 |
| CSNK1G1 | 0.216238721530037 | 0.0000217469756907171 | 0.000311785274038712 | 379 | 379 |
| CSNK1G2 | 0.0672328988007785 | 0.191539101284837 | 0.334522795761099 | 379 | 379 |
| CSNK1G3 | -0.0482628153943886 | 0.348748629708895 | 0.510368870755625 | 379 | 379 |
| CSNK2A1P | 0.117395166237016 | 0.0222659656776416 | 0.0675680390964611 | 379 | 379 |
| CSNK2A1 | 0.149485102255997 | 0.00353463191563867 | 0.0164171191434255 | 379 | 379 |
| CSNK2A2 | -0.109557402004911 | 0.0329877810304003 | 0.0913649563166331 | 379 | 379 |
| CSNK2B | -0.00243552405932379 | 0.962307566065471 | 0.979789324642834 | 379 | 379 |
| CSPG4 | 0.0984871501643804 | 0.0554075607929949 | 0.134965738993059 | 379 | 379 |
| CSPG5 | 0.109300735522191 | 0.0334029300795835 | 0.0921928309601867 | 379 | 379 |
| CSPP1 | -0.0891748884963361 | 0.0829587347673424 | 0.182807934315055 | 379 | 379 |
| CSRNP1 | -0.0540238869567942 | 0.294166602887426 | 0.452619974623135 | 379 | 379 |
| CSRNP2 | 0.0136520931708929 | 0.791077002061652 | 0.872239047816184 | 379 | 379 |
| CSRNP3 | -0.0143019678840154 | 0.781377706878461 | 0.86574667691519 | 379 | 328 |
| CSRP1 | 0.00627191979872236 | 0.903137307515926 | 0.944400866374789 | 379 | 379 |
| CSRP2BP | -0.0533397109975394 | 0.300332434927775 | 0.459242039002693 | 379 | 379 |
| CSRP2 | 0.161240668197054 | 0.00163671464974212 | 0.00895838826394691 | 379 | 379 |
| CSRP3 | 0.0251721273234419 | 0.625190259069817 | 0.751152666596154 | 379 | 28 |
| CST11 | -0.0337178850398788 | 0.512831579539365 | 0.661791380351873 | 379 | 24 |
| CST1 | -0.0703266227825797 | 0.171850159154556 | 0.309491775110096 | 379 | 379 |
| CST2 | 0.0716800590010497 | 0.163727373693001 | 0.299233695786232 | 379 | 354 |
| CST3 | -0.0466377447666989 | 0.365235678837806 | 0.526272558914525 | 379 | 379 |
| CST4 | -0.0581647896686722 | 0.258660073720819 | 0.412806820809433 | 379 | 366 |
| CST5 | -0.0369508520087264 | 0.473235195111702 | 0.625899552970123 | 379 | 230 |
| CST6 | 0.128365604431937 | 0.0123803890115081 | 0.0430933553793939 | 379 | 354 |
| CST7 | 0.0948383907697203 | 0.0651296994737862 | 0.152017855346231 | 379 | 379 |
| CST8 | -0.0571993262493328 | 0.266661587591324 | 0.421976533021609 | 379 | 7 |
| CST9L | -0.00773698738324369 | 0.880663800696896 | 0.930155107879298 | 379 | 8 |
| CST9 | -0.0298455548332584 | 0.562426736876107 | 0.703213103571006 | 379 | 55 |
| CSTA | 0.166216572852723 | 0.00116291261642835 | 0.00691608619032431 | 379 | 379 |
| CSTB | 0.164349368942376 | 0.00132351286602833 | 0.00759554648555997 | 379 | 379 |
| CSTF1 | -0.25147885242162 | 7.06652320921656E-07 | 0.0000226723336880819 | 379 | 379 |
| CSTF2T | -0.0782620119402902 | 0.128285031947976 | 0.250752722147522 | 379 | 379 |
| CSTF2 | 0.196959331857023 | 0.000113574839927627 | 0.00110282170719148 | 379 | 379 |
| CSTF3 | -0.0220431172441701 | 0.668819909578736 | 0.783212920335884 | 379 | 379 |
| CSTL1 | -0.00173793222573453 | 0.973098679329154 | 0.985877094360966 | 379 | 267 |
| CSTT | 0.0412307361355322 | 0.423498898564059 | 0.581117700340489 | 379 | 5 |
| CS | 0.0703772882501735 | 0.171540771047821 | 0.309125730104171 | 379 | 379 |
| CT45A1 | 0.129962140214948 | 0.0113257351655733 | 0.0401370289299351 | 379 | 41 |
| CT45A2 | 0.147827440923077 | 0.00392347838993124 | 0.0178101486986164 | 379 | 23 |
| CT45A3 | 0.0970511943767431 | 0.0590793728445796 | 0.14140823331269 | 379 | 11 |
| CT45A4 | 0.0508015874285968 | 0.323950822479339 | 0.484594259382899 | 379 | 9 |
| CT45A5 | 0.15570012798072 | 0.00236810623241527 | 0.0119599618890295 | 379 | 34 |
| CT45A6 | 0.153469027269929 | 0.00273887907633845 | 0.0134218849634558 | 379 | 8 |
| CT47A1 | -0.00354216399195066 | 0.945203736377602 | 0.968938371154328 | 379 | 3 |
| CT47A2 | 0.041341796256032 | 0.422250192915095 | 0.580125888658571 | 379 | 4 |
| CT47A6 | 0.0118545196995767 | 0.818069517021973 | 0.890365703343489 | 379 | 3 |
| CT47B1 | 0.0318955075596062 | 0.535886240252835 | 0.681387224043428 | 379 | 11 |
| CT62 | -0.0679537943301277 | 0.186809747546762 | 0.328432671959319 | 379 | 286 |
| CTAG1B | 0.189139498811225 | 0.000212668801705446 | 0.0018022098649536 | 379 | 83 |
| CTAG2 | 0.185362850972994 | 0.000285403561447859 | 0.00227542493622362 | 379 | 63 |
| CTAGE1 | -0.0017642659501589 | 0.972691219000407 | 0.985565000272884 | 379 | 378 |
| CTAGE4 | -0.0167286618851531 | 0.745469250820153 | 0.839193556009697 | 379 | 379 |
| CTAGE5 | 0.0638168671216303 | 0.21514490662387 | 0.362407034962033 | 379 | 379 |
| CTAGE6 | -0.0115972539323119 | 0.821951084900883 | 0.892972774719999 | 379 | 376 |
| CTAGE9 | -0.0393591377721859 | 0.444863844992203 | 0.600255891017721 | 379 | 379 |
| CTBP1 | 0.0390071070167429 | 0.448949726043397 | 0.604071984552906 | 379 | 379 |
| CTBP2 | -0.00977352944505098 | 0.849586266663755 | 0.910603499640586 | 379 | 379 |
| CTBS | 0.0998111029003358 | 0.0521919514976981 | 0.128938701008766 | 379 | 379 |
| CTCFL | -0.0496266276569588 | 0.335281689908001 | 0.496339058346711 | 379 | 379 |
| CTCF | -0.03735622814521 | 0.4683912675811 | 0.621282912956034 | 379 | 379 |
| CTDP1 | 0.165494908274745 | 0.00122272818576453 | 0.00717497912617314 | 379 | 379 |
| CTDSP1 | 0.000361329617034721 | 0.994405996159584 | 0.996667951870405 | 379 | 379 |
| CTDSP2 | -0.150938334532367 | 0.00322283777511228 | 0.0152184871171532 | 379 | 379 |
| CTDSPL2 | 0.00724272455061474 | 0.888235805455289 | 0.935130947056256 | 379 | 379 |
| CTDSPL | -0.113650724308792 | 0.0269384943141412 | 0.0783765906472182 | 379 | 379 |
| CTF1 | 0.0845931003394661 | 0.100101728471772 | 0.209656392958519 | 379 | 379 |
| CTGF | 0.112826717513911 | 0.0280730834815616 | 0.0808120062822885 | 379 | 379 |
| CTHRC1 | 0.116689676592516 | 0.0230884163970258 | 0.0694005754775912 | 379 | 379 |
| CTH | -0.0457051103960692 | 0.374912989206516 | 0.535722192873601 | 379 | 379 |
| CTLA4 | 0.160246681501373 | 0.00175036412178805 | 0.00944130027388834 | 379 | 379 |
| CTNNA1 | -0.103548631654849 | 0.0439427487208782 | 0.113396496253728 | 379 | 379 |
| CTNNA2 | -0.218491857122643 | 0.0000177475629571084 | 0.000268767772078959 | 379 | 332 |
| CTNNA3 | 0.139019575498227 | 0.0067150075233024 | 0.0270235780742927 | 379 | 217 |
| CTNNAL1 | 0.110068814043162 | 0.0321736936856221 | 0.0896360823940585 | 379 | 379 |
| CTNNB1 | -0.251606345713121 | 6.97261607449176E-07 | 0.0000224072984643473 | 379 | 379 |
| CTNNBIP1 | -0.00220631106261204 | 0.965852621156616 | 0.981525199668057 | 379 | 379 |
| CTNNBL1 | -0.142816791560296 | 0.0053452771637483 | 0.0225838814410401 | 379 | 379 |
| CTNND1 | 0.00551265533532191 | 0.914815744629371 | 0.951227992265518 | 379 | 379 |
| CTNND2 | 0.0708854279887946 | 0.168460757745859 | 0.304933348967035 | 379 | 284 |
| CTNS | 0.230768408080147 | 5.64804456467776E-06 | 0.000111210950971629 | 379 | 379 |
| CTPS2 | -0.255608170740404 | 4.56488786971107E-07 | 0.0000162593353119288 | 379 | 379 |
| CTPS | 0.224509346058185 | 0.0000102065080007668 | 0.000175977948381917 | 379 | 379 |
| CTR9 | 0.0652559199175309 | 0.20495789802054 | 0.350972035750174 | 379 | 379 |
| CTRB1 | 0.0171011155444029 | 0.740004221034498 | 0.835390781978594 | 379 | 15 |
| CTRB2 | 0.0396102313078729 | 0.441962455441843 | 0.597927918019983 | 379 | 65 |
| CTRC | 0.0515350898957858 | 0.317004672466593 | 0.476974400187252 | 379 | 136 |
| CTRL | 0.189755634476241 | 0.000202593999267714 | 0.00173521979156813 | 379 | 379 |
| CTSA | -0.101749433786471 | 0.0477657057607164 | 0.12081877966877 | 379 | 379 |
| CTSB | 0.156755125954068 | 0.00220924805797374 | 0.0112928513775466 | 379 | 379 |
| CTSC | 0.221943638604703 | 0.0000129458276012098 | 0.000211267382450031 | 379 | 379 |
| CTSD | 0.224600361670809 | 0.0000101202720467712 | 0.000174795082006428 | 379 | 379 |
| CTSE | 0.204454740086639 | 0.0000608228781691512 | 0.00069111520248592 | 379 | 368 |
| CTSF | -0.0295517905443355 | 0.566281230210415 | 0.706177624692585 | 379 | 379 |
| CTSG | 0.149468341595921 | 0.00353838217318194 | 0.0164268418941352 | 379 | 333 |
| CTSH | -0.046325359913522 | 0.368459637377644 | 0.529330364434425 | 379 | 379 |
| CTSK | 0.0482677227354769 | 0.348699566826394 | 0.510334736568773 | 379 | 379 |
| CTSL1 | 0.198136017479866 | 0.000103124481406495 | 0.00102211091954522 | 379 | 379 |
| CTSL2 | -0.19192807558668 | 0.000170523011195973 | 0.00152853990325215 | 379 | 379 |
| CTSO | 0.00405815614368095 | 0.937236859923912 | 0.964291232650912 | 379 | 379 |
| CTSS | -0.149806223350241 | 0.00346347206161611 | 0.0161406440207073 | 379 | 379 |
| CTSW | 0.232393125243015 | 0.0000048306298676848 | 0.0000987701853637539 | 379 | 376 |
| CTSZ | -0.0474882305670214 | 0.356547532632541 | 0.517703731560574 | 379 | 379 |
| CTTNBP2NL | -0.123872575071052 | 0.0158272116316577 | 0.0520517139200074 | 379 | 379 |
| CTTNBP2 | -0.302319221642626 | 1.8872259699209E-09 | 2.44650348167908E-07 | 379 | 379 |
| CTTN | 0.195361267893936 | 0.000129367291193218 | 0.00122089226548269 | 379 | 379 |
| CTU1 | 0.0289625439131016 | 0.574050596781961 | 0.712015215375499 | 379 | 379 |
| CTU2 | -0.028044215123613 | 0.586258264059047 | 0.721780270882847 | 379 | 379 |
| CTXN1 | 0.218193799780126 | 0.0000182333842163188 | 0.00027368019851716 | 379 | 379 |
| CTXN2 | 0.0561698607228526 | 0.275378603053345 | 0.431671036551643 | 379 | 126 |
| CTXN3 | 0.00870822968869216 | 0.865816814452179 | 0.921365494819573 | 379 | 32 |
| CUBN | 0.122618399166088 | 0.0169284023206667 | 0.0546849725014955 | 379 | 379 |
| CUEDC1 | -0.183488982648914 | 0.000329558901437622 | 0.00253569805886891 | 379 | 379 |
| CUEDC2 | -0.0878214418390618 | 0.0877563160787283 | 0.190688464132496 | 379 | 379 |
| CUL1 | 0.0100911507181326 | 0.84475903583328 | 0.907606727851654 | 379 | 379 |
| CUL2 | 0.0570862934256368 | 0.267609347076351 | 0.423239860718664 | 379 | 379 |
| CUL3 | -0.0584136003218345 | 0.25662513663012 | 0.410782588695223 | 379 | 379 |
| CUL4A | -0.164258001435386 | 0.00133187144399086 | 0.00763467678272644 | 379 | 379 |
| CUL4B | -0.0297311044414921 | 0.563926944021488 | 0.704393564700647 | 379 | 379 |
| CUL5 | 0.100366508823225 | 0.0508900547667669 | 0.126605772385879 | 379 | 379 |
| CUL7 | -0.0271486142960846 | 0.598278016937458 | 0.730795451177404 | 379 | 379 |
| CUL9 | -0.0397465607520563 | 0.440391696191159 | 0.596663132629351 | 379 | 379 |
| CUTA | -0.112749691600725 | 0.0281812084823522 | 0.0810056540719164 | 379 | 379 |
| CUTC | 0.0170045447626542 | 0.741419966448999 | 0.836417563424599 | 379 | 379 |
| CUX1 | -0.201561305479898 | 0.0000776144122563005 | 0.000827386325923616 | 379 | 379 |
| CUX2 | 0.112273402244196 | 0.0288577691435873 | 0.0823179177929864 | 379 | 274 |
| CUZD1 | -0.0118675268750705 | 0.81787338476862 | 0.89024997107994 | 379 | 373 |
| CWC15 | 0.0299236401273307 | 0.561404303475156 | 0.702304386706964 | 379 | 379 |
| CWC22 | 0.076938360089156 | 0.134888523923643 | 0.260070950248736 | 379 | 379 |
| CWC25 | 0.014748433827161 | 0.774733735474354 | 0.861114440662901 | 379 | 379 |
| CWC27 | -0.0476814353170734 | 0.354592078333287 | 0.515760836941932 | 379 | 379 |
| CWF19L1 | -0.00280006435754424 | 0.956671068060575 | 0.976719733149945 | 379 | 379 |
| CWF19L2 | 0.0279275650640412 | 0.587817477937519 | 0.722959375465951 | 379 | 379 |
| CWH43 | -0.0705510915992174 | 0.170482598490852 | 0.307666238543426 | 379 | 313 |
| CX3CL1 | 0.173536073903263 | 0.000691301031083018 | 0.00459816063210804 | 379 | 379 |
| CX3CR1 | -0.104973133633957 | 0.0411014998362843 | 0.107884635789495 | 379 | 377 |
| CXADRP2 | -0.128383425107712 | 0.0123681542698499 | 0.0430767192802713 | 379 | 288 |
| CXADRP3 | -0.0341292800423121 | 0.507699227065655 | 0.65687832132188 | 379 | 337 |
| CXADR | -0.132281551587941 | 0.00993512305424734 | 0.0364025350878752 | 379 | 379 |
| CXCL10 | 0.254380664105462 | 5.20222171046287E-07 | 0.0000178329995492093 | 379 | 379 |
| CXCL11 | 0.246462867390764 | 1.18935262909877E-06 | 0.0000340787340025585 | 379 | 379 |
| CXCL12 | 0.0151257986554432 | 0.76913083705691 | 0.85690432304121 | 379 | 379 |
| CXCL13 | 0.227738409148085 | 0.0000075371355470245 | 0.000139669461333086 | 379 | 375 |
| CXCL14 | -0.279196881581089 | 3.24394399143786E-08 | 2.19298726537792E-06 | 379 | 379 |
| CXCL16 | 0.219103114253829 | 0.0000167893973265626 | 0.000257264428277498 | 379 | 379 |
| CXCL17 | 0.0500880567299166 | 0.330801875005788 | 0.491652767979518 | 379 | 321 |
| CXCL1 | 0.0367632550656011 | 0.475486088489999 | 0.627650500138453 | 379 | 379 |
| CXCL2 | -0.0443533852538444 | 0.389216080248391 | 0.548966882854253 | 379 | 379 |
| CXCL3 | 0.0245225541982616 | 0.634147181900806 | 0.758467265214692 | 379 | 379 |
| CXCL5 | 0.167907344170396 | 0.00103316756598246 | 0.00631688143641695 | 379 | 375 |
| CXCL6 | 0.0665033486250725 | 0.196413951153402 | 0.34038060029697 | 379 | 378 |
| CXCL9 | 0.213384630925615 | 0.0000280463987872646 | 0.000378817435390928 | 379 | 379 |
| CXCR1 | 0.125286274116358 | 0.0146616438720317 | 0.0490734427236063 | 379 | 365 |
| CXCR2P1 | 0.160839403264866 | 0.00168175429801121 | 0.0091383459087329 | 379 | 355 |
| CXCR2 | 0.0437454359201526 | 0.395755403185025 | 0.555385245548353 | 379 | 376 |
| CXCR3 | 0.0251287111281095 | 0.625787225676326 | 0.7515966509607 | 379 | 379 |
| CXCR4 | 0.205577195666461 | 0.0000552832524122152 | 0.000641027092882692 | 379 | 379 |
| CXCR5 | 0.138842571726692 | 0.00678591409037485 | 0.0272480973236032 | 379 | 371 |
| CXCR6 | 0.146812687503431 | 0.0041802096751583 | 0.0186720426760619 | 379 | 379 |
| CXCR7 | 0.00797744118508909 | 0.876983979709247 | 0.928075924588486 | 379 | 379 |
| CXXC1 | 0.216608321610538 | 0.0000210370152045968 | 0.000305021808672107 | 379 | 379 |
| CXXC4 | -0.0974185784025577 | 0.0581213171464165 | 0.139654756485488 | 379 | 367 |
| CXXC5 | -0.180477593745299 | 0.000414061927127743 | 0.00303288507243772 | 379 | 379 |
| CXorf1 | 0.103149835671055 | 0.0447671471209196 | 0.114994557988547 | 379 | 26 |
| CXorf21 | 0.134038119266977 | 0.00898501705979124 | 0.0336649505407295 | 379 | 378 |
| CXorf22 | 0.0476638055048683 | 0.354770232135082 | 0.515868595099326 | 379 | 116 |
| CXorf23 | -0.203496965243177 | 0.0000659596276162028 | 0.000732277433580106 | 379 | 379 |
| CXorf26 | 0.0480588859747058 | 0.350791347936134 | 0.512075027163709 | 379 | 379 |
| CXorf27 | -0.0157391818747669 | 0.760049361169307 | 0.85046606846868 | 379 | 132 |
| CXorf30 | 0.13627987749169 | 0.00789030679467764 | 0.0305266347560718 | 379 | 85 |
| CXorf36 | 0.135454485761293 | 0.0082786992176053 | 0.0316708562775763 | 379 | 379 |
| CXorf38 | -0.0270663988121415 | 0.599386982431119 | 0.731407784334065 | 379 | 379 |
| CXorf40A | -0.0274883842458114 | 0.593704892142018 | 0.727069396664315 | 379 | 379 |
| CXorf40B | 0.0357027834226812 | 0.48831954096916 | 0.639947115554297 | 379 | 379 |
| CXorf41 | 0.0613791567795438 | 0.23322129852303 | 0.384238629589916 | 379 | 37 |
| CXorf42 | 0.0350996591380359 | 0.495700488406923 | 0.646628242377136 | 379 | 379 |
| CXorf48 | 0.166002250371532 | 0.00118038947776946 | 0.00698877915569756 | 379 | 120 |
| CXorf49B | 0.106356671497428 | 0.0384919343853198 | 0.102738630020834 | 379 | 51 |
| CXorf50B | -0.156379194805616 | 0.00226470359415208 | 0.0115228490800224 | 379 | 366 |
| CXorf51 | 0.0729332260703498 | 0.1564665212552 | 0.289701950083865 | 379 | 4 |
| CXorf56 | 0.0106609669869655 | 0.836113577692756 | 0.901741217205166 | 379 | 379 |
| CXorf57 | 0.0866662184374848 | 0.0920253529859565 | 0.197582966865787 | 379 | 379 |
| CXorf58 | -0.0000319949767973607 | 0.99950465872805 | 0.999756766042464 | 379 | 186 |
| CXorf59 | 0.0917790212891398 | 0.0743240853439201 | 0.167890921959287 | 379 | 60 |
| CXorf61 | 0.0710436009679729 | 0.167510503888408 | 0.30385482609858 | 379 | 52 |
| CXorf64 | 0.000202165851496406 | 0.996870107415216 | 0.997804556718965 | 379 | 11 |
| CXorf65 | -0.00483837357570397 | 0.925202499573154 | 0.957409068500418 | 379 | 312 |
| CYB561D1 | -0.0596555579311865 | 0.246633282048437 | 0.40023753631856 | 379 | 379 |
| CYB561D2 | -0.0113176221934853 | 0.826175031515902 | 0.895264675809092 | 379 | 379 |
| CYB561 | 0.00485187790379695 | 0.924994345886962 | 0.957343558317501 | 379 | 379 |
| CYB5A | 0.0597292117616269 | 0.246049367424792 | 0.399563215175985 | 379 | 379 |
| CYB5B | -0.0401102883392771 | 0.436216541843061 | 0.592673093321243 | 379 | 379 |
| CYB5D1 | 0.124043373888537 | 0.0156822097527232 | 0.0516694674272176 | 379 | 379 |
| CYB5D2 | 0.146498692595939 | 0.00426267078416752 | 0.0189677370530686 | 379 | 379 |
| CYB5R1 | -0.119900723472145 | 0.0195467200606891 | 0.0612020605830566 | 379 | 379 |
| CYB5R2 | 0.110248797544744 | 0.0318912973347891 | 0.0890270505382157 | 379 | 379 |
| CYB5R3 | 0.198959241836858 | 0.0000963582044722973 | 0.000980296936310063 | 379 | 379 |
| CYB5R4 | -0.0802996486147003 | 0.118612344005931 | 0.236651796835339 | 379 | 379 |
| CYB5RL | -0.0683879226243528 | 0.18400346199856 | 0.324737040009564 | 379 | 379 |
| CYBASC3 | 0.124044684166707 | 0.0156811018890849 | 0.0516694674272176 | 379 | 379 |
| CYBA | 0.059618859527089 | 0.246924581218403 | 0.400525245124222 | 379 | 379 |
| CYBB | 0.174506545478732 | 0.000644227148405975 | 0.00434480812877336 | 379 | 379 |
| CYBRD1 | 0.0336944666396886 | 0.513124543360937 | 0.662068590854608 | 379 | 379 |
| CYC1 | 0.0131404020683083 | 0.798736745398056 | 0.877026923676633 | 379 | 379 |
| CYCSP52 | -0.165478980963707 | 0.00122407969196152 | 0.00718078465450092 | 379 | 119 |
| CYCS | 0.0135382439118481 | 0.792779555865927 | 0.873242210638831 | 379 | 379 |
| CYFIP1 | 0.0563199602363787 | 0.274095711153209 | 0.430408629187126 | 379 | 379 |
| CYFIP2 | -0.284246226888001 | 1.78137461232502E-08 | 1.36374887309577E-06 | 379 | 379 |
| CYGB | 0.139999035436287 | 0.00633450100271031 | 0.0258650094484638 | 379 | 379 |
| CYHR1 | -0.108270701535756 | 0.0351140470907475 | 0.0957954493279226 | 379 | 379 |
| CYLC1 | 0.0319409100877099 | 0.535305607332021 | 0.680998305028828 | 379 | 3 |
| CYLD | 0.0236327525850081 | 0.646503532290423 | 0.767775759275987 | 379 | 379 |
| CYMP | 0.203835975524219 | 0.0000640963980696036 | 0.000718024508996667 | 379 | 62 |
| CYP11A1 | 0.0125076425908991 | 0.80823548756166 | 0.883917927175758 | 379 | 304 |
| CYP11B1 | 0.0648990172994984 | 0.207451214633569 | 0.353835929785325 | 379 | 18 |
| CYP11B2 | -0.0247464110114437 | 0.631054314931216 | 0.755907989878339 | 379 | 9 |
| CYP17A1 | 0.0406321350618401 | 0.430266241128777 | 0.587202861615952 | 379 | 192 |
| CYP19A1 | 0.0544411909887121 | 0.29044763370792 | 0.448869499700751 | 379 | 373 |
| CYP1A1 | 0.110201301071227 | 0.0319656135520794 | 0.0891942281889431 | 379 | 193 |
| CYP1A2 | -0.00786785819572691 | 0.878660676002627 | 0.929227365927787 | 379 | 33 |
| CYP1B1 | 0.159213057932141 | 0.00187617174386579 | 0.00995736973698364 | 379 | 379 |
| CYP20A1 | -0.144029385890248 | 0.00496411861160596 | 0.0213221908114127 | 379 | 379 |
| CYP21A2 | 0.00237803924341291 | 0.963196571000687 | 0.979900544371555 | 379 | 375 |
| CYP24A1 | -0.0964014589801965 | 0.0608056898732061 | 0.144441741800159 | 379 | 331 |
| CYP26A1 | -0.0352035749881083 | 0.494424566297548 | 0.645473419841176 | 379 | 312 |
| CYP26B1 | 0.154942748026294 | 0.00248849926272512 | 0.0124224479811968 | 379 | 379 |
| CYP26C1 | -0.0319543887165247 | 0.535133295906179 | 0.680925599749082 | 379 | 139 |
| CYP27A1 | -0.0129861537205711 | 0.801049587175109 | 0.878350451501856 | 379 | 379 |
| CYP27B1 | 0.0216465764891315 | 0.674433518306826 | 0.787646825361512 | 379 | 379 |
| CYP27C1 | 0.109156012355135 | 0.0336389716648017 | 0.0926910395995709 | 379 | 364 |
| CYP2A13 | 0.0330927258858539 | 0.520681981565286 | 0.668336316162312 | 379 | 7 |
| CYP2A6 | -0.0274085292185007 | 0.594778265668608 | 0.727950738167641 | 379 | 139 |
| CYP2A7 | -0.0307390687739314 | 0.550781083107464 | 0.694683901618791 | 379 | 117 |
| CYP2B6 | -0.21100848922991 | 0.0000345725596650523 | 0.000446582874943751 | 379 | 375 |
| CYP2B7P1 | -0.141069691092335 | 0.00594077733885129 | 0.0246172900887656 | 379 | 372 |
| CYP2C18 | 0.159308849921707 | 0.00186417378556227 | 0.00991226543848988 | 379 | 372 |
| CYP2C19 | 0.180352683881523 | 0.00041796855199541 | 0.00305472924768337 | 379 | 289 |
| CYP2C8 | -0.0157532272134318 | 0.759841792211061 | 0.850281791069525 | 379 | 346 |
| CYP2C9 | 0.180026777839012 | 0.000428323454562237 | 0.00311091481943591 | 379 | 325 |
| CYP2D6 | -0.0858084099348481 | 0.0953015708671731 | 0.202729272304936 | 379 | 379 |
| CYP2D7P1 | -0.0890254781650966 | 0.0834777286816613 | 0.183665823823788 | 379 | 378 |
| CYP2E1 | 0.0649879962195507 | 0.206827567818402 | 0.353075937555168 | 379 | 375 |
| CYP2F1 | 0.0439964242257748 | 0.393047708216474 | 0.552876699667724 | 379 | 237 |
| CYP2J2 | -0.0481817279165666 | 0.349559962857132 | 0.51101425288295 | 379 | 379 |
| CYP2R1 | -0.130426156915672 | 0.0110344971452281 | 0.039372190080916 | 379 | 379 |
| CYP2S1 | 0.00820110189137274 | 0.87356352821091 | 0.926208097821823 | 379 | 379 |
| CYP2U1 | -0.102126229455569 | 0.0469428251277209 | 0.119285189879848 | 379 | 379 |
| CYP2W1 | -0.149955366111056 | 0.00343086605644285 | 0.0160290320846251 | 379 | 376 |
| CYP39A1 | -0.27290800510697 | 6.73079006686555E-08 | 3.69690042786178E-06 | 379 | 379 |
| CYP3A43 | 0.0218271390415402 | 0.671875132064242 | 0.78576553499641 | 379 | 141 |
| CYP3A4 | -0.0249829854772421 | 0.627792718246413 | 0.753319620423149 | 379 | 365 |
| CYP3A5 | -0.032066338565835 | 0.533703200976672 | 0.679701141304223 | 379 | 379 |
| CYP3A7 | -0.215372042112712 | 0.0000235018987548389 | 0.000329325546650844 | 379 | 368 |
| CYP46A1 | 0.0695612281897226 | 0.176574756307197 | 0.315644091963496 | 379 | 348 |
| CYP4A11 | -0.0278020568107246 | 0.58949722577905 | 0.724186759878873 | 379 | 185 |
| CYP4A22 | -0.0661664777090345 | 0.198695222890643 | 0.343356623176677 | 379 | 31 |
| CYP4B1 | -0.0296328226809565 | 0.56521674422623 | 0.705453175916651 | 379 | 321 |
| CYP4F11 | -0.0846528249579669 | 0.0998614290361777 | 0.209285743042948 | 379 | 375 |
| CYP4F12 | -0.116999416189487 | 0.0227241448267873 | 0.0686118994404658 | 379 | 378 |
| CYP4F22 | 0.156925788861202 | 0.00218448416884474 | 0.0111866660898635 | 379 | 313 |
| CYP4F2 | -0.0861313686403464 | 0.0940573435313835 | 0.200706953028441 | 379 | 367 |
| CYP4F3 | -0.0742341840671772 | 0.149188873913691 | 0.280024411270774 | 379 | 379 |
| CYP4F8 | -0.158821100657835 | 0.00192600398803021 | 0.0101565976262402 | 379 | 282 |
| CYP4V2 | -0.167721055645579 | 0.0010467787561833 | 0.0063882822953532 | 379 | 379 |
| CYP4X1 | -0.141384184923386 | 0.00582937494448207 | 0.0242468735890897 | 379 | 379 |
| CYP4Z1 | -0.142052768847974 | 0.00559875446284291 | 0.0234846844699067 | 379 | 176 |
| CYP4Z2P | -0.131266635519812 | 0.0105238098976333 | 0.0379695439059324 | 379 | 61 |
| CYP51A1 | 0.0541405887603555 | 0.293123380193455 | 0.451526599011484 | 379 | 379 |
| CYP7A1 | 0.0579602940939489 | 0.2603408914444 | 0.414421900735353 | 379 | 134 |
| CYP7B1 | 0.109408109863489 | 0.0332287172813505 | 0.0918654498402981 | 379 | 377 |
| CYP8B1 | 0.0700522751306666 | 0.173532686891911 | 0.311667220624349 | 379 | 252 |
| CYR61 | 0.104946389066727 | 0.041153379865676 | 0.107992220219246 | 379 | 379 |
| CYS1 | 0.0283923045584351 | 0.581616888351446 | 0.718031234806829 | 379 | 379 |
| CYSLTR1 | 0.0852945819461747 | 0.0973081643598719 | 0.205673838922027 | 379 | 377 |
| CYSLTR2 | 0.146226169953688 | 0.00433542844477653 | 0.0191924258100087 | 379 | 356 |
| CYTH1 | 0.180270683348196 | 0.000420551775683556 | 0.00306475623909492 | 379 | 379 |
| CYTH2 | -0.05829485922268 | 0.257594894170024 | 0.411902545290583 | 379 | 379 |
| CYTH3 | 0.0946958303734066 | 0.0655363388188356 | 0.152625619696955 | 379 | 379 |
| CYTH4 | 0.240330731920306 | 0.000002214112109104 | 0.0000540657818957071 | 379 | 379 |
| CYTIP | 0.202357032170177 | 0.0000726061934777361 | 0.00078711624071982 | 379 | 379 |
| CYTL1 | 0.144550832287511 | 0.00480787546937167 | 0.0207737099164745 | 379 | 371 |
| CYTSA | 0.101444424904624 | 0.0484406247233068 | 0.122012284935687 | 379 | 379 |
| CYTSB | 0.0932392152827091 | 0.0698118471845239 | 0.160248819862785 | 379 | 379 |
| CYYR1 | 0.0932953926824091 | 0.0696428259654796 | 0.159955269005858 | 379 | 379 |
| CYorf15A | 0.0554828070782312 | 0.281302860021794 | 0.438599756901166 | 379 | 246 |
| CYorf15B | 0.0546446471283745 | 0.288645930177754 | 0.447095656867785 | 379 | 243 |
| D2HGDH | -0.0802605272745676 | 0.118792533317216 | 0.236939779761972 | 379 | 379 |
| D4S234E | -0.138221241792198 | 0.00704015028149565 | 0.0280756435602365 | 379 | 379 |
| DAAM1 | 0.0187146242287861 | 0.716482976206212 | 0.8189556956371 | 379 | 379 |
| DAAM2 | 0.0594092211739432 | 0.248593219495703 | 0.402179043420431 | 379 | 379 |
| DAB1 | 0.0177920626790447 | 0.72990064983692 | 0.828465858661999 | 379 | 322 |
| DAB2IP | -0.112712387813831 | 0.0282337019629677 | 0.081106965900565 | 379 | 379 |
| DAB2 | -0.198062756639364 | 0.000103747794242645 | 0.00102701510945739 | 379 | 379 |
| DACH1 | -0.343992644325959 | 0.0000000000057396023 | 2.32254763956932E-09 | 379 | 379 |
| DACH2 | -0.0684092560320915 | 0.183866364787571 | 0.324639561977557 | 379 | 197 |
| DACT1 | 0.0120342344630076 | 0.815360648261763 | 0.888463695784313 | 379 | 379 |
| DACT2 | -0.0810732511352601 | 0.115092868603929 | 0.231446389318327 | 379 | 373 |
| DACT3 | 0.0809390649915404 | 0.115697408297656 | 0.232426363903335 | 379 | 379 |
| DAD1L | 0.0233264844409192 | 0.65077935628018 | 0.770321358505367 | 379 | 38 |
| DAD1 | 0.127527254768547 | 0.0129681562670377 | 0.0446565825743006 | 379 | 379 |
| DAG1 | -0.0566150305072725 | 0.271585635083007 | 0.427652475573845 | 379 | 379 |
| DAGLA | -0.144592519961123 | 0.00479557782772689 | 0.0207431756475063 | 379 | 379 |
| DAGLB | 0.211216792899668 | 0.0000339474771211083 | 0.000439941553174729 | 379 | 379 |
| DAK | 0.0612197440939308 | 0.23443968576318 | 0.385381370362489 | 379 | 379 |
| DALRD3 | -0.191933651980377 | 0.000170447179922262 | 0.00152853990325215 | 379 | 379 |
| DAND5 | 0.0359691985180661 | 0.485078086341276 | 0.637004324523135 | 379 | 334 |
| DAO | 0.123600364653056 | 0.0160607360476802 | 0.0526287269959715 | 379 | 198 |
| DAP3 | -0.102240780638868 | 0.0466950250354315 | 0.118762051873081 | 379 | 379 |
| DAPK1 | 0.208601280019333 | 0.0000426321978326193 | 0.00052347664645704 | 379 | 379 |
| DAPK2 | -0.34500399278392 | 4.9309721120217E-12 | 2.22207534175378E-09 | 379 | 379 |
| DAPK3 | 0.0275485584246797 | 0.592896643128411 | 0.726512040487853 | 379 | 379 |
| DAPL1 | -0.121627525996922 | 0.0178451995782994 | 0.0569873759443584 | 379 | 320 |
| DAPP1 | 0.219118400563095 | 0.0000167660767873761 | 0.000257264428277498 | 379 | 379 |
| DAP | -0.0566306378717306 | 0.271453305852583 | 0.427545964607596 | 379 | 379 |
| DARC | 0.0338022847252415 | 0.511776461451076 | 0.660840856277561 | 379 | 376 |
| DARS2 | -0.0362322838248547 | 0.481888569847573 | 0.633854308307013 | 379 | 379 |
| DARS | -0.127734969497921 | 0.0128202811766159 | 0.0442703823005817 | 379 | 379 |
| DAXX | 0.106766385651027 | 0.0377466052927257 | 0.101222570968916 | 379 | 379 |
| DAZ1 | 0.0275468647929955 | 0.59291938482095 | 0.726512040487853 | 379 | 116 |
| DAZ2 | 0.0065585120911612 | 0.898734442492743 | 0.941676909919738 | 379 | 18 |
| DAZ3 | 0.00686862577077773 | 0.893973721612454 | 0.938858625743769 | 379 | 12 |
| DAZAP1 | 0.0676682986310473 | 0.188672329976996 | 0.330754013230036 | 379 | 379 |
| DAZAP2 | -0.0263583335166347 | 0.608975870358467 | 0.737976626174531 | 379 | 379 |
| DAZL | 0.0266395254830546 | 0.60515972048953 | 0.73574317025653 | 379 | 263 |
| DBC1 | -0.0747825012899894 | 0.146199790019286 | 0.275879410058277 | 379 | 363 |
| DBF4B | 0.152734846475014 | 0.00287198221168363 | 0.013939917528862 | 379 | 379 |
| DBF4 | 0.120099612465455 | 0.0193438062285406 | 0.0607458013778116 | 379 | 379 |
| DBH | 0.0539393354159774 | 0.29492397455315 | 0.453454758641428 | 379 | 348 |
| DBI | -0.0813049169693651 | 0.114054987769595 | 0.229989046831641 | 379 | 379 |
| DBN1 | 0.0302285706611538 | 0.557420201906476 | 0.699445485330993 | 379 | 379 |
| DBNDD1 | -0.107523095222182 | 0.036402171905752 | 0.0983944810186998 | 379 | 379 |
| DBNDD2 | -0.257741492451886 | 3.63153803988548E-07 | 0.0000134842951788107 | 379 | 379 |
| DBNL | 0.0857331090926744 | 0.0955935533989416 | 0.203176007803003 | 379 | 379 |
| DBP | -0.0450355895688674 | 0.381956542852669 | 0.541849776896525 | 379 | 379 |
| DBR1 | -0.00357753157438536 | 0.944657480432009 | 0.968593883649078 | 379 | 379 |
| DBT | 0.0150605217951155 | 0.770099181298551 | 0.857586699956676 | 379 | 379 |
| DBX1 | -0.0596742382913652 | 0.246485095987178 | 0.400106957284794 | 379 | 160 |
| DBX2 | 0.103163766278205 | 0.044738132032569 | 0.11493499564872 | 379 | 82 |
| DCAF10 | -0.157803088174263 | 0.00206113806380798 | 0.0106845086350809 | 379 | 379 |
| DCAF11 | -0.0652343109714575 | 0.205108237862105 | 0.351138502877726 | 379 | 379 |
| DCAF12L1 | -0.00607804407164637 | 0.906117476601337 | 0.94580423910567 | 379 | 31 |
| DCAF12L2 | 0.0437132041730226 | 0.396103934902275 | 0.555795684752834 | 379 | 168 |
| DCAF12 | -0.107892715598873 | 0.0357603889873543 | 0.0970513266960391 | 379 | 379 |
| DCAF13 | -0.084362617688801 | 0.101033373835232 | 0.211163288465003 | 379 | 379 |
| DCAF15 | -0.00404554677651499 | 0.937431476192514 | 0.964308939208039 | 379 | 379 |
| DCAF16 | -0.126088747047892 | 0.0140341281149575 | 0.0474375540851307 | 379 | 379 |
| DCAF17 | -0.0400100427066159 | 0.437364966802636 | 0.593855547610948 | 379 | 379 |
| DCAF4L1 | 0.0113798042223622 | 0.825235309794692 | 0.894951386226961 | 379 | 371 |
| DCAF4L2 | 0.0860507133129318 | 0.0943668534031629 | 0.20130241735104 | 379 | 17 |
| DCAF4 | -0.187641501079701 | 0.000239151654226039 | 0.00198821761006033 | 379 | 379 |
| DCAF5 | -0.0334254032161457 | 0.516496741549298 | 0.664933271227964 | 379 | 379 |
| DCAF6 | -0.142033139535336 | 0.00560540680899398 | 0.0235076155263817 | 379 | 379 |
| DCAF7 | 0.0282109717331546 | 0.584032619970128 | 0.720161616216897 | 379 | 379 |
| DCAF8L1 | 0.0257879361787045 | 0.616749481616767 | 0.744548504236058 | 379 | 55 |
| DCAF8L2 | 0.0594465292753023 | 0.248295688628969 | 0.40189444197022 | 379 | 58 |
| DCAF8 | -0.171471624564271 | 0.000802215124542357 | 0.00516099849116706 | 379 | 379 |
| DCAKD | 0.0222031586019633 | 0.66655948293001 | 0.781304175191312 | 379 | 379 |
| DCBLD1 | 0.218128201009923 | 0.0000183419887621332 | 0.00027489414450157 | 379 | 379 |
| DCBLD2 | 0.104813830377669 | 0.0414113380933069 | 0.108584045528236 | 379 | 379 |
| DCC | 0.0664247392045392 | 0.196944572893227 | 0.341079307478986 | 379 | 265 |
| DCDC1 | 0.151711647157865 | 0.00306729950949408 | 0.0146656413489869 | 379 | 165 |
| DCDC2B | -0.0454956842360755 | 0.377107570288693 | 0.537853144470528 | 379 | 170 |
| DCDC2 | 0.109185233688624 | 0.0335911979002031 | 0.0925964509891878 | 379 | 368 |
| DCHS1 | -0.00157944909269612 | 0.975551024431499 | 0.987250607251374 | 379 | 379 |
| DCHS2 | 0.0143243131147776 | 0.781044799367776 | 0.86560596287878 | 379 | 348 |
| DCI | 0.0763132776042456 | 0.138096530871641 | 0.264481600900503 | 379 | 379 |
| DCK | 0.0341373034351806 | 0.507599399002559 | 0.656792017973293 | 379 | 379 |
| DCLK1 | 0.0846806753705017 | 0.099749530865476 | 0.209095432709658 | 379 | 374 |
| DCLK2 | 0.0450576703714335 | 0.3817229650233 | 0.541635347638262 | 379 | 379 |
| DCLK3 | 0.147074651302401 | 0.00411251885804716 | 0.0184486479451039 | 379 | 379 |
| DCLRE1A | 0.0302314784065288 | 0.557382276544511 | 0.699445485330993 | 379 | 379 |
| DCLRE1B | 0.172054474814592 | 0.000769340982501094 | 0.00500639744044362 | 379 | 379 |
| DCLRE1C | 0.125292913024775 | 0.0146563529523364 | 0.0490723009690859 | 379 | 379 |
| DCN | 0.0462547867873995 | 0.369190420632367 | 0.53002798822898 | 379 | 379 |
| DCP1A | -0.136709348689658 | 0.00769471722752923 | 0.0299982015704777 | 379 | 379 |
| DCP1B | -0.139759142596602 | 0.00642586445598921 | 0.0261733854628912 | 379 | 379 |
| DCP2 | 0.0659786202225027 | 0.199975732720208 | 0.344932056034514 | 379 | 379 |
| DCPS | 0.11278573542931 | 0.028130567599183 | 0.0809187428342666 | 379 | 379 |
| DCST1 | -0.0629681626975689 | 0.221320604591757 | 0.369675720759803 | 379 | 191 |
| DCST2 | -0.00443420859098816 | 0.931434535548334 | 0.960799290961002 | 379 | 372 |
| DCTD | 0.0422986768230228 | 0.411580849020901 | 0.570208571435608 | 379 | 379 |
| DCTN1 | 0.019806985131537 | 0.700708046228132 | 0.806503694236455 | 379 | 379 |
| DCTN2 | 0.077770963652861 | 0.130704966743583 | 0.254279638990557 | 379 | 379 |
| DCTN3 | -0.165710190726403 | 0.00120459464296159 | 0.00709797996452968 | 379 | 379 |
| DCTN4 | -0.1802821949908 | 0.000420188236065737 | 0.00306474447280188 | 379 | 379 |
| DCTN5 | -0.0966537120126319 | 0.0601305850208409 | 0.14331881714067 | 379 | 379 |
| DCTN6 | 0.0410076302741843 | 0.426013886490365 | 0.583413577414386 | 379 | 379 |
| DCTPP1 | -0.112545953576361 | 0.0284689300935018 | 0.0815018691732535 | 379 | 379 |
| DCT | 0.0207091118543281 | 0.687776194192348 | 0.797358731125878 | 379 | 170 |
| DCUN1D1 | -0.0800559719884755 | 0.119738179050008 | 0.238250739006879 | 379 | 379 |
| DCUN1D2 | -0.0932363102500188 | 0.069820596682883 | 0.160250352011599 | 379 | 379 |
| DCUN1D3 | 0.0300000102398281 | 0.560405195286565 | 0.701748072042227 | 379 | 379 |
| DCUN1D4 | 0.0135568198702866 | 0.792501695083049 | 0.873081654078603 | 379 | 379 |
| DCUN1D5 | 0.162111163303295 | 0.00154278325054998 | 0.00856655594520935 | 379 | 379 |
| DCXR | -0.108602478055536 | 0.0345549330367212 | 0.0945563362202745 | 379 | 379 |
| DCX | -0.0361091231623477 | 0.483380292867297 | 0.635321784898101 | 379 | 255 |
| DDA1 | 0.185879482597693 | 0.000274238635936408 | 0.00221490984657723 | 379 | 379 |
| DDAH1 | -0.00769406271738212 | 0.881320977195232 | 0.930551804453223 | 379 | 379 |
| DDAH2 | -0.199682906899334 | 0.0000907572370349745 | 0.000933368514486242 | 379 | 379 |
| DDB1 | 0.142708747875386 | 0.00538048426881431 | 0.0226939464118379 | 379 | 379 |
| DDB2 | 0.0701798455007676 | 0.172748800582662 | 0.310709653297625 | 379 | 379 |
| DDC | -0.0233778118127391 | 0.650061968641434 | 0.7698761009807 | 379 | 379 |
| DDHD1 | 0.0478455598095348 | 0.352936259092266 | 0.51410668125782 | 379 | 379 |
| DDHD2 | -0.0189911367808073 | 0.712478099343764 | 0.815883092912974 | 379 | 379 |
| DDI1 | -0.0792625376915655 | 0.123461838074239 | 0.243679208175992 | 379 | 13 |
| DDI2 | 0.113099794208489 | 0.0276926171655705 | 0.080042159352614 | 379 | 379 |
| DDIT3 | 0.0931810860825254 | 0.0699870937137548 | 0.160555264419178 | 379 | 379 |
| DDIT4L | -0.153425685426954 | 0.00274657805336475 | 0.013450024609068 | 379 | 368 |
| DDIT4 | 0.200443799114029 | 0.0000851993240763047 | 0.000888892667634882 | 379 | 379 |
| DDN | 0.0311257079583033 | 0.54577869712947 | 0.689983423022388 | 379 | 379 |
| DDOST | 0.194269820748934 | 0.000141313685418515 | 0.00130810819536803 | 379 | 379 |
| DDO | -0.110478729400213 | 0.0315336127073873 | 0.0881873727450036 | 379 | 378 |
| DDR1 | -0.0394652824434724 | 0.443636023593715 | 0.599281592108146 | 379 | 379 |
| DDR2 | 0.0813700531959808 | 0.113764498214791 | 0.229613443668859 | 379 | 379 |
| DDRGK1 | 0.170831123744575 | 0.000839835185025149 | 0.00535945763129753 | 379 | 379 |
| DDTL | -0.00230756104249673 | 0.964286581222676 | 0.980657246370376 | 379 | 379 |
| DDT | 0.0156493566154406 | 0.761377249698194 | 0.851567469935457 | 379 | 379 |
| DDX10 | 0.0787485290550239 | 0.125921783544341 | 0.247181182468784 | 379 | 379 |
| DDX11L2 | -0.0233927506388879 | 0.649853233687652 | 0.769793290365364 | 379 | 372 |
| DDX11 | 0.143466525466741 | 0.00513789052261297 | 0.0219319899423832 | 379 | 379 |
| DDX12 | 0.0581968556104222 | 0.258397193916149 | 0.412577510986049 | 379 | 379 |
| DDX17 | -0.0872105656570628 | 0.0899935011251637 | 0.194252633952726 | 379 | 379 |
[truncated: 1,285,539 more chars]
