# Supplementary material for: Abnormal Expression of N6-Methyladenosine RNA Methylation Regulator IGF2BP3 in Colon Cancer Predicts a Poor Prognosis
Source: Dis Markers. 2022 May 30;2022:5883101. doi: 10.1155/2022/5883101 (PMC9170420; doi:10.1155/2022/5883101)
Supplement: Supplementary Materials — See Figures S1 in the Supplementary Material for correlations between expression of IGF2BP3 and clinicopathological features in TCGA database, Figure S2 and Table S4 for the DEGs in groups with different expression of IGF2BP3, Table S1 and Table S2 for the clinical information of the samples, Table S3 for gene coexpressed with IGF2BP3, Table S5 for the genes related to IGF2BP3, and Table S6 for the results of KEGG enrichment analysis. [file 5883101.f1.zip › Table S2 (2).docx]

|  | baseMean | log2FoldChange | lfcSE | stat | pvalue | padj |
| --- | --- | --- | --- | --- | --- | --- |
| TMEM176A | 7108.51717115095 | 0.631858558645759 | 0.109046226118 | 5.79441014274091 | 6.85617200187786E-09 | 5.8640435827826E-07 |
| CYP26B1 | 83.5278669641475 | -0.506139035792598 | 0.126962285217036 | -3.98653060574152 | 0.0000670464826274473 | 0.000958023962144719 |
| TFPI | 534.878564448604 | -0.571998354877952 | 0.106254092710788 | -5.38330656528091 | 7.3129829635213E-08 | 4.07734133920457E-06 |
| SLC7A2 | 155.532650493839 | -0.862508278412877 | 0.156268640547066 | -5.51939452082904 | 0.0000000340169705975 | 2.24534900599703E-06 |
| HSPB6 | 252.451586162076 | 0.58130114733655 | 0.180616062363198 | 3.21843550197446 | 0.00128891954850915 | 0.00943608746524583 |
| ARX | 21.0978882619903 | -1.36433210703087 | 0.250850855661716 | -5.43881783234073 | 5.363526017515E-08 | 3.19158428963944E-06 |
| CALCR | 5.11111946445911 | -0.828502560379742 | 0.216521071190849 | -3.8264292515414 | 0.000130015493775622 | 0.00161574810213465 |
| PON1 | 8.85176778159469 | -0.926298530932762 | 0.248296549049255 | -3.73061379418935 | 0.000191013843509042 | 0.00218165469761932 |
| ASB4 | 19.7415026308438 | -0.726964575267673 | 0.227544150844066 | -3.19482866323317 | 0.00139913876301777 | 0.0100493983060224 |
| TAC1 | 71.1136010162884 | 1.02208321104351 | 0.232819808107773 | 4.39001826928054 | 0.0000113341138122379 | 0.000236601394574819 |
| CX3CL1 | 927.046144492463 | -0.552514610548444 | 0.124618569069233 | -4.43364592191305 | 9.26527463250998E-06 | 0.000199967883273743 |
| DLX6 | 30.0887435440631 | 0.995423697565933 | 0.264981195827728 | 3.75658240373059 | 0.000172249570349051 | 0.00200989885959977 |
| ETV1 | 276.03734134982 | -0.555456081241291 | 0.115037145067962 | -4.828493274178 | 1.37569979708823E-06 | 0.0000438520440169442 |
| CCL26 | 32.3384232255967 | -0.803776969832594 | 0.167537004363479 | -4.79760858137801 | 1.60571169201904E-06 | 0.0000494464511984051 |
| PRSS21 | 106.703622795055 | -0.930772818929258 | 0.283538311971771 | -3.28270565080434 | 0.00102815960923773 | 0.00796098019647721 |
| SLC13A2 | 281.898142297428 | 0.592262870263367 | 0.225128845175858 | 2.63077292383713 | 0.0085190936113756 | 0.0393718000502013 |
| TENM1 | 8.04990242301656 | -1.06465898521388 | 0.192859004661518 | -5.52040070455842 | 3.38227478683938E-08 | 2.24424669487069E-06 |
| DPF1 | 23.083825707422 | -0.866214434658617 | 0.119393177323939 | -7.25514182697719 | 4.0124118136649E-13 | 1.86494226713184E-10 |
| GABRA3 | 26.4678253206659 | -1.5761403540631 | 0.345944772532213 | -4.55604616461241 | 5.21254849688758E-06 | 0.000126961432507585 |
| CD22 | 78.9130613345498 | -0.628990555835215 | 0.159256040292174 | -3.94955541203498 | 0.0000782964843971223 | 0.00108153285056116 |
| LTF | 163.733675656669 | -0.540591215507965 | 0.15055135317333 | -3.59074298645182 | 0.000329736664846094 | 0.00335016003885136 |
| NR1H4 | 37.2218439964184 | -0.609999890840776 | 0.230273913465502 | -2.64901864766441 | 0.00807258772540452 | 0.0378654318735041 |
| MAMLD1 | 87.9308739492653 | -0.648653530035996 | 0.119194541175984 | -5.44197346318316 | 5.26935266298107E-08 | 3.14674449563237E-06 |
| DPEP1 | 9401.57200531032 | 1.03545709048719 | 0.177653237040202 | 5.82852926148976 | 5.59179930594963E-09 | 4.86981646847832E-07 |
| NPC1L1 | 203.16599855844 | -0.889939091808027 | 0.233679621568195 | -3.80837270206001 | 0.000139884327287282 | 0.00171292994256363 |
| ISL1 | 38.7257542854166 | -0.687739862452993 | 0.21346872598249 | -3.22173592074281 | 0.00127416514554559 | 0.00935674808900652 |
| IGF1 | 22.7081906807311 | -0.517394502160379 | 0.170927324998155 | -3.02698531183334 | 0.00247005960447654 | 0.0154804597625383 |
| SLC11A1 | 321.30799481488 | -0.632668533200432 | 0.129200752206931 | -4.8967867631849 | 9.74164484776469E-07 | 0.0000333791072744823 |
| VSIG2 | 857.095025975363 | 0.532987941470595 | 0.197460261784598 | 2.69921622028443 | 0.00695030033582105 | 0.0338658380606548 |
| MARCO | 173.53653206221 | -0.857353366482636 | 0.216904997827811 | -3.95266764283247 | 0.0000772847476159689 | 0.00107020974317732 |
| SYT13 | 1254.89122423974 | -0.662479600953607 | 0.1401246028533 | -4.72778932081736 | 2.26977512375794E-06 | 0.0000652111852995818 |
| SLC7A9 | 32.284688555684 | -0.628438110554771 | 0.231681615061286 | -2.71250746585365 | 0.00667762771311693 | 0.0328643452512224 |
| NRXN3 | 211.90464152499 | 0.605219724336552 | 0.171148440062027 | 3.53622693912496 | 0.000405885859382689 | 0.00392869317206249 |
| GRAMD1B | 217.177089029894 | -1.0313923179958 | 0.143605398962504 | -7.18212772950896 | 6.86347515161901E-13 | 3.02010968447951E-10 |
| BIRC3 | 1360.96395205211 | -0.801710009634995 | 0.116790439910199 | -6.86451742327059 | 0.0000000000066716398 | 1.906948530511E-09 |
| KCNG1 | 66.2612403553557 | 0.855045121041097 | 0.186773928925098 | 4.57796827406248 | 4.69513879814997E-06 | 0.000116135230538263 |
| POU2F2 | 136.286154644633 | -0.514282504827972 | 0.104873568624238 | -4.9038333640638 | 9.39842269739076E-07 | 0.0000325364442904909 |
| ARNTL2 | 903.479694907373 | -0.677968594736559 | 0.0839675912014768 | -8.07416986763145 | 6.79372441131815E-16 | 7.5717536910403E-13 |
| ANK1 | 60.4484064995783 | -0.618498771166903 | 0.134707266285228 | -4.59142842270512 | 4.40222621293781E-06 | 0.000110029334090483 |
| FUT8 | 1550.00378018582 | -0.51655769310904 | 0.0817456302212348 | -6.31908631337281 | 2.63114295634453E-10 | 3.94612406427635E-08 |
| DAPK2 | 809.898447693371 | 0.564094782055025 | 0.090907525790772 | 6.20514943232881 | 5.46449539810627E-10 | 7.22306937167865E-08 |
| STAP1 | 12.8138674357 | -0.604418081426344 | 0.155351475968592 | -3.89064910814586 | 0.0000999764191776113 | 0.00131673902675496 |
| OTC | 101.418684470663 | -0.60892242373105 | 0.227367560548833 | -2.67814116605376 | 0.00740320095505622 | 0.0355613108789127 |
| SLC18A1 | 97.1750170491852 | -0.987806470484076 | 0.227883286641245 | -4.33470345738506 | 0.0000145956741856018 | 0.000290476675828294 |
| HOXC8 | 11.002164394814 | -1.22357682003691 | 0.2322702923502 | -5.26790063273392 | 1.37992763413652E-07 | 6.86719344357047E-06 |
| TLL1 | 19.0944005026961 | -0.578391179856754 | 0.159559755380154 | -3.62491894324309 | 0.000289052096506642 | 0.00302739749808178 |
| RAB27B | 483.229354903143 | -0.913551135639004 | 0.146490793967059 | -6.23623581318314 | 4.4822527507009E-10 | 6.10454918572386E-08 |
| BARX2 | 221.812743035677 | -0.675209923823344 | 0.161401508022782 | -4.18341768980274 | 0.0000287159088888066 | 0.000497294406956446 |
| ZIC2 | 233.554441948622 | -1.0895136719839 | 0.245949272748363 | -4.42983083385091 | 9.43070357173649E-06 | 0.000203209786627585 |
| ADRB1 | 34.2543417351452 | -0.756229858212163 | 0.167083739799555 | -4.52605297870032 | 6.00955204845382E-06 | 0.000142129730979061 |
| MAGEC2 | 2.08914404638247 | -2.77524859271893 | 0.588045478496459 | -4.71944550923987 | 2.36488382125229E-06 | 0.0000675952519233497 |
| CP | 81.2610093101418 | -0.555240586903338 | 0.174950463535153 | -3.17370171923994 | 0.00150508276597064 | 0.0106682869562505 |
| GUCA1A | 4.33499616853595 | -0.642708184449744 | 0.190359190855031 | -3.37629184891419 | 0.000734699406406475 | 0.00622493477300363 |
| UTS2 | 28.3403764832737 | -0.664394074690998 | 0.195545705826956 | -3.39764083226118 | 0.000679695929453035 | 0.00585382211505754 |
| FSTL4 | 35.9930495646547 | 1.12129423782958 | 0.20722998291475 | 5.41086874620287 | 6.27197309498569E-08 | 3.62257900246134E-06 |
| LAMA3 | 2889.22162659477 | -0.55252349726763 | 0.113034229407773 | -4.88810778967131 | 1.01809785951633E-06 | 0.0000345657143329393 |
| PTPRN | 55.2254514386589 | -0.573525130092534 | 0.168356459495763 | -3.40661196968785 | 0.000657745650817087 | 0.00568821568518878 |
| FOXC1 | 200.670031522164 | -0.740185231486273 | 0.142521022973125 | -5.19351612867563 | 2.06359055539773E-07 | 9.37145429924803E-06 |
| ATP9A | 6964.10868005524 | 0.581639247371701 | 0.0850701222529709 | 6.83717422718748 | 8.07704888898407E-12 | 2.25093890787838E-09 |
| MCOLN3 | 87.7080646651646 | -0.527399076765477 | 0.170992005980371 | -3.08434931645881 | 0.00203997918880568 | 0.0133845367926309 |
| CCDC85A | 7.88879541624566 | 0.514343428527419 | 0.168010884816062 | 3.06136967905693 | 0.00220326880617662 | 0.0141668362653641 |
| GYG2 | 608.511656992428 | 0.581618550739802 | 0.117503653025564 | 4.94979122575248 | 0.0000007429313257573 | 0.0000268349810983674 |
| DCBLD2 | 1041.23391031331 | -0.849412212917864 | 0.126749527107898 | -6.7015020276548 | 2.06287993638818E-11 | 4.82425390438417E-09 |
| SERPINB3 | 5.20355955789118 | -2.11599519161141 | 0.444148380908858 | -4.7641627946081 | 1.89639358336238E-06 | 0.0000570316494737452 |
| MSH4 | 10.8416681056875 | -1.47985946457616 | 0.200214948256059 | -7.39135353012474 | 1.4534160891864E-13 | 7.7151017229479E-11 |
| F7 | 69.5838147811512 | 1.1552707229451 | 0.228000577275858 | 5.0669640259171 | 4.0421063581022E-07 | 0.0000161887569853478 |
| RASGRF1 | 65.055309495383 | 0.795496976389545 | 0.162029189477127 | 4.90959054326346 | 9.12667536726791E-07 | 0.0000317600705132335 |
| PTPRU | 647.526324635353 | -0.563174902450143 | 0.159644216791605 | -3.52768746509181 | 0.000419206801201878 | 0.00402848099017046 |
| RIMBP2 | 100.631697888924 | -0.706359747191471 | 0.187509773886209 | -3.76705561823209 | 0.000165184210891967 | 0.00194578738310995 |
| BCAT1 | 353.432832541637 | -0.567741497639687 | 0.124687736929793 | -4.55330661714843 | 5.28092160661322E-06 | 0.000128268576199506 |
| LTK | 217.99491573355 | -0.520471629461962 | 0.166756990176919 | -3.12113830376631 | 0.00180153403670398 | 0.0122031398127313 |
| SEZ6 | 4.50627230001326 | -0.62474624537644 | 0.206326131188819 | -3.0279550233252 | 0.00246214729462579 | 0.0154540408834226 |
| DLX3 | 68.2788746338388 | 0.99917485671411 | 0.269324891428318 | 3.70992391908212 | 0.000207321540564952 | 0.00232114059577272 |
| CCN5 | 30.3871281817278 | -0.718627959715745 | 0.208434876989975 | -3.44773374827433 | 0.000565311008321313 | 0.00506975884695129 |
| DMRT3 | 8.25857515762567 | 0.983898255957518 | 0.235958809198153 | 4.1697881901551 | 0.0000304882860545945 | 0.000521144618654916 |
| EYA2 | 103.6457169144 | 0.717419175343843 | 0.169692905253986 | 4.22774997145612 | 0.0000236039874914923 | 0.000424389542844346 |
| SNCAIP | 194.098072208462 | 0.638758664546185 | 0.155943939125087 | 4.09607880966645 | 0.0000420206932811914 | 0.000665367435942047 |
| CHI3L2 | 38.6263815907544 | -0.735391837593451 | 0.156421637644651 | -4.70134342452076 | 2.58455407284803E-06 | 0.0000728774513526002 |
| ROPN1 | 1.18354301428354 | 0.844896074797943 | 0.286953746768615 | 2.94436327914277 | 0.00323619894522534 | 0.0190402823937765 |
| TLE2 | 1801.1234098433 | 0.794956984184774 | 0.125702817436851 | 6.32409838056439 | 0.0000000002547154119 | 3.87190582031683E-08 |
| SLC9A7 | 431.47633004208 | -0.560557912097113 | 0.11031843891857 | -5.0812712506826 | 0.0000003749173014577 | 0.0000152530223787695 |
| CTNNA2 | 54.2164645099767 | 1.75781178493675 | 0.250012323744904 | 7.03090055164764 | 2.05204729322154E-12 | 7.22363848209629E-10 |
| CLDN18 | 440.3128228631 | -3.63186478994771 | 0.318354109063815 | -11.4082547909557 | 3.80282530882205E-30 | 4.23913613258757E-26 |
| NAV3 | 36.4686911303201 | -0.711499952640194 | 0.155156306870981 | -4.58569791321364 | 4.52472426348513E-06 | 0.000112418892139279 |
| FGFR3 | 1211.6337933388 | 0.627869313855444 | 0.122291824368207 | 5.13418879061775 | 2.83363548575493E-07 | 0.0000122274113438215 |
| REEP1 | 248.948164001223 | 0.97741150384208 | 0.17244686414379 | 5.66789955094278 | 1.44558666762386E-08 | 1.09374003028681E-06 |
| MAOB | 271.687633283343 | -0.653671077629567 | 0.16822850271794 | -3.88561431070656 | 0.00010207144952184 | 0.00133809228338274 |
| SCT | 24.0810442665243 | 0.522208464944943 | 0.18872575103751 | 2.76702284703666 | 0.00565707904103999 | 0.0291052365062245 |
| FSTL3 | 839.332319118875 | -0.528058807866273 | 0.113128167562325 | -4.66779246269814 | 3.04453198224987E-06 | 0.0000821090633470969 |
| FRMPD1 | 19.9050029875927 | -0.716050967345838 | 0.1903172128219 | -3.76240780709585 | 0.000168285350733033 | 0.00197605291404989 |
| ST6GALNAC2 | 195.162132440958 | -0.554832554705671 | 0.137584121186371 | -4.03267869810424 | 0.0000551446526369049 | 0.000819985537342541 |
| EPHA8 | 5.39608909152777 | 1.34228675531901 | 0.332352955921049 | 4.03873873063394 | 0.0000537393817698554 | 0.000805175808757843 |
| TRIB2 | 773.438140518138 | -0.715793928419678 | 0.108910374886721 | -6.57232085707336 | 4.95369705140137E-11 | 1.01632844658261E-08 |
| SLC6A15 | 4.39306826198615 | -1.46524218329036 | 0.267422165836242 | -5.47913512968709 | 4.27409856840235E-08 | 2.65184423607628E-06 |
| RPS6KA6 | 211.023531679356 | 0.571233257700986 | 0.167120811591266 | 3.41808570854762 | 0.000630632446376581 | 0.00550769587305707 |
| LIMS2 | 622.230576658126 | 0.568235870884973 | 0.124051864534266 | 4.58063144007006 | 4.63574159383827E-06 | 0.000114951277144243 |
| CYP2W1 | 1914.66961526563 | 0.844661148485007 | 0.233329533081253 | 3.62003530942167 | 0.000294562816504279 | 0.00307452238125346 |
| TP63 | 12.5560873133356 | -0.728223684863804 | 0.173381681665276 | -4.20011893914885 | 0.000026677480256769 | 0.000470046519887707 |
| ABCB11 | 4.69740522525044 | -0.748324004517193 | 0.193041285041104 | -3.87649721849839 | 0.000105971020815352 | 0.00137609120262808 |
| PTGS2 | 491.881413381817 | -0.981592626050779 | 0.162547425393916 | -6.038807589059 | 1.5525724283836E-09 | 1.74231970302028E-07 |
| ST6GAL1 | 4464.82317401408 | 0.596070552927142 | 0.101740974339994 | 5.85870694470858 | 4.66485049786698E-09 | 4.23918288993662E-07 |
| TBX21 | 20.8788961406229 | -0.612434803339499 | 0.143523802410062 | -4.26713055991723 | 0.0000198003290730699 | 0.000369716697298494 |
| SNCB | 1.64417989931803 | -0.743806476496066 | 0.220461012284649 | -3.37386855293805 | 0.000741197519715764 | 0.0062657046143414 |
| TACR2 | 125.229142638436 | 0.584657371794298 | 0.173878207315726 | 3.36245341391565 | 0.000772531600351707 | 0.0064660536750394 |
| ADD2 | 22.1303218043245 | -0.78036120023502 | 0.178188794043963 | -4.37940670973106 | 0.0000119002852007273 | 0.000246420642528001 |
| FSCN1 | 2395.85577019077 | -0.848166231069254 | 0.133547726071153 | -6.35103461527574 | 2.13871571865723E-10 | 3.43860245496803E-08 |
| ATP12A | 24.4039643333952 | 1.29513663179729 | 0.309347237774708 | 4.18667592157559 | 0.0000283069548502024 | 0.0004909964647824 |
| RAP1GAP | 1589.69387494065 | -0.547303312796496 | 0.130454414078933 | -4.19536062969356 | 0.0000272437766959942 | 0.000477758982835574 |
| ACTL6B | 1.10889881181439 | -0.727426420650664 | 0.276866899522227 | -2.62735062192679 | 0.00860526147586861 | 0.0396496492526864 |
| CAPN6 | 453.879941546144 | 1.27700715758264 | 0.22177603696741 | 5.75809350299779 | 8.50692330396838E-09 | 6.93874461295879E-07 |
| SPAG6 | 1.0074004126988 | -0.753713896039296 | 0.243471151624605 | -3.09570103484542 | 0.00196348221650832 | 0.0129742683826262 |
| TYR | 3.41694565164062 | -1.44993875620112 | 0.460601705653546 | -3.14792311536018 | 0.00164434937157979 | 0.0114151707404066 |
| SMC1B | 10.7340085331226 | -0.776250551619499 | 0.167975036872536 | -4.62122566586211 | 3.81479539157451E-06 | 0.0000982853524538019 |
| RBFOX1 | 14.8194881800048 | 1.21497035246605 | 0.263589927146478 | 4.60932011180719 | 4.03987906063947E-06 | 0.000103010102826041 |
| SLC1A3 | 133.897651530849 | -0.549180057845781 | 0.138935428994379 | -3.95277188706128 | 0.0000772510743690247 | 0.00107018659032681 |
| SP140 | 86.1119902157943 | -0.837226710971669 | 0.130674160190135 | -6.40697984784043 | 1.48430428932255E-10 | 2.54554379710384E-08 |
| RIMS1 | 7.74744991505593 | 0.606085292235708 | 0.232225707556938 | 2.60989749417431 | 0.00905693569195784 | 0.041204561426812 |
| SESN1 | 1889.49237260265 | 0.732948600517793 | 0.113934765962124 | 6.4330548654609 | 1.25064480979904E-10 | 2.18974155650782E-08 |
| KCNN2 | 29.5200262311771 | -0.569170590616359 | 0.137206474546573 | -4.14827793292774 | 0.0000334985567134905 | 0.000556235716788753 |
| KCNK2 | 7.01921977324089 | -0.563208143342704 | 0.216462117521525 | -2.60187856328579 | 0.00927146756923167 | 0.0418938546750771 |
| EPYC | 47.6297815640687 | -0.695249593073794 | 0.261804431281174 | -2.65560666666909 | 0.00791658923644885 | 0.0373513794082001 |
| FAM234B | 105.264530254427 | -0.54984867342755 | 0.0983980337679214 | -5.58800468233345 | 2.29693452655762E-08 | 1.63434222206681E-06 |
| WNT11 | 837.856616764486 | 0.654632151923154 | 0.194691554372777 | 3.36240652057112 | 0.000772662819923779 | 0.0064660536750394 |
| HSD17B2 | 541.847542467488 | -0.677579695550862 | 0.158198249848126 | -4.28310487759096 | 0.000018430324411094 | 0.00034802197004845 |
| TMPRSS11E | 8.16580664276282 | -1.36734931488076 | 0.328614883144029 | -4.16094761685356 | 0.0000316929781502161 | 0.000533405422898604 |
| MT3 | 48.775161529089 | 0.951068967543332 | 0.222952843993263 | 4.26578531365166 | 0.0000199200263583162 | 0.000371744152608712 |
| DOCK3 | 28.0642074746424 | -0.638156718585193 | 0.129756515875056 | -4.91810923159866 | 8.73841494639616E-07 | 0.0000306000076060084 |
| SLC4A11 | 306.979068795569 | -0.694179717327888 | 0.147033235772419 | -4.72124355885326 | 2.34407039354255E-06 | 0.0000672301904810035 |
| LAG3 | 138.280211147107 | -0.543812919957684 | 0.131543047272198 | -4.13410614422204 | 0.0000356338768720497 | 0.000584151034487788 |
| LYZ | 19498.4574009336 | -0.683812300632308 | 0.180419966508252 | -3.7901143308384 | 0.00015057796058786 | 0.00181419902030259 |
| ABCC6 | 345.639067785161 | 0.507652685164936 | 0.10713934816344 | 4.73824690804091 | 0.0000021557505194342 | 0.0000629318899019938 |
| SMPX | 23.3364630393419 | 0.934653534731809 | 0.263407504323135 | 3.54831779426156 | 0.00038770013526375 | 0.00379440091410311 |
| APOH | 20.8563732911763 | -0.518657107252012 | 0.19613663431275 | -2.64436630652581 | 0.00818440313368865 | 0.0382053056388632 |
| CPA1 | 1.05751078993299 | -1.19207365521322 | 0.37265372544578 | -3.19887760088061 | 0.00137963729418113 | 0.00995101124476563 |
| COL9A3 | 875.007182124891 | 0.782717518447082 | 0.18090244553643 | 4.32673818270445 | 0.0000151333663223075 | 0.000297875242231081 |
| GABRP | 226.605876181996 | -0.825534130954709 | 0.21421678165078 | -3.85373230142402 | 0.000116330784903341 | 0.00148656251766815 |
| FMO2 | 23.8098594466416 | -0.556325162488257 | 0.193792353188126 | -2.87072814451145 | 0.00409527555964745 | 0.0227083742771895 |
| CRISP3 | 0.813166752344294 | -1.5119538688026 | 0.522379104346226 | -2.89436131005826 | 0.00379930706713644 | 0.0214731159268509 |
| MACROH2A2 | 795.128104395491 | 0.539901712332386 | 0.115679785416075 | 4.66720879875839 | 3.05319019675441E-06 | 0.0000822099730755725 |
| MAGEB2 | 5.70256985306389 | -3.42499118337729 | 0.605542007101888 | -5.65607528991958 | 1.54873550611272E-08 | 1.16388343360498E-06 |
| HCN2 | 33.3666652379079 | -0.807130164186599 | 0.164822855535768 | -4.89695534980854 | 9.73329431217607E-07 | 0.0000333791072744823 |
| IGF2-AS | 8.13414808008718 | 1.74425184654722 | 0.268321429423524 | 6.50060582300366 | 7.99971919604797E-11 | 1.46992642502328E-08 |
| P2RX6 | 10.0686434702019 | -0.905742072337566 | 0.15861329167851 | -5.71037939350882 | 1.12724597481496E-08 | 8.78726337756687E-07 |
| OSM | 216.257441088979 | -0.576832368514562 | 0.159931614899283 | -3.60674385035019 | 0.000310063470212446 | 0.00319345320937623 |
| PRODH | 140.119958611489 | -0.800001372357729 | 0.178632133841055 | -4.47848522634547 | 0.0000075174572033629 | 0.000169634820374401 |
| PLA2G3 | 80.8066039644416 | -0.830468329037086 | 0.2624851312621 | -3.16386808290424 | 0.00155687344895406 | 0.010935719781542 |
| CYP2D6 | 59.8172654606298 | 0.607338143496745 | 0.122529824367673 | 4.95665562756637 | 7.17169258381274E-07 | 0.0000260691025421593 |
| MIOX | 14.2006387362533 | -0.64051428254431 | 0.207761087645767 | -3.0829367029325 | 0.00204968746833931 | 0.0134324217746822 |
| APOL1 | 4017.98783700999 | -0.721388796164957 | 0.121476839714579 | -5.93848833950508 | 0.0000000028766210786 | 2.85459828221385E-07 |
| PVALB | 1.86588194064378 | -0.630423432525508 | 0.221396108938812 | -2.84749102207456 | 0.00440653370970211 | 0.0240396901011188 |
| GZMH | 60.3951983187533 | -0.770818454443874 | 0.152273992024521 | -5.06204929808202 | 4.14773732961551E-07 | 0.000016552342694153 |
| LRRC74A | 4.26084235041857 | 0.736473768089032 | 0.184441318533065 | 3.99299774012948 | 0.000065243190510033 | 0.000936421792719538 |
| ISM2 | 94.5140922248089 | 1.2014944249303 | 0.244061318127998 | 4.92292033062028 | 8.5262170370935E-07 | 0.0000299706227188904 |
| SIX4 | 51.8455442876166 | -0.726784106931078 | 0.148050449402785 | -4.90903006281185 | 9.15279480717646E-07 | 0.0000318178548795837 |
| SLC10A1 | 3.39314476985285 | 0.511497222090128 | 0.145964628199898 | 3.50425461564314 | 0.000457887337716696 | 0.00430010343946131 |
| CPNE6 | 25.1957653984246 | 0.73061465149464 | 0.216781258879731 | 3.37028512183325 | 0.000750904440627631 | 0.00632378400993937 |
| PYGB | 21162.9121901624 | 0.525817648355509 | 0.107496276055028 | 4.89149640947879 | 1.00072219594021E-06 | 0.0000341454454167198 |
| NINL | 255.343503429462 | 0.606115949778633 | 0.161270619977726 | 3.75837799756923 | 0.000171018390722078 | 0.00199971923864606 |
| SGK2 | 734.767460976891 | 0.537461011923812 | 0.115651885598113 | 4.64723086134085 | 0.0000033642059316326 | 0.0000891487914149425 |
| R3HDML | 55.5759322133442 | 0.99902095358758 | 0.157666728583383 | 6.33628262959259 | 2.35374854863091E-10 | 0.0000000364593101158 |
| SALL4 | 120.406855757993 | 0.620536770451995 | 0.132134910275786 | 4.69623636294782 | 2.64998879804672E-06 | 0.0000740977636992294 |
| NTSR1 | 117.403941650384 | -1.67300593510254 | 0.223065971852835 | -7.5000499682053 | 6.37935102905738E-14 | 4.02525013422145E-11 |
| RSPO4 | 12.6118641236443 | 1.26998505232749 | 0.278394235808692 | 4.56182236905295 | 5.07115416321798E-06 | 0.000124150466710348 |
| PLCB4 | 4644.85392947204 | 0.696785134813626 | 0.144778038747085 | 4.81278197193188 | 1.48843646371614E-06 | 0.0000466218466642063 |
| POFUT1 | 5488.90461832545 | 0.609254873830055 | 0.0783164170743312 | 7.77940177283394 | 7.28682982640679E-15 | 0.0000000000058020515 |
| PXMP4 | 886.245408536937 | 0.54203434201515 | 0.0632523076981867 | 8.56940025969501 | 0.0000000000000000104 | 2.31921105513262E-14 |
| SLC32A1 | 1.2305087662254 | 1.39481130960241 | 0.428243826788496 | 3.25704942453563 | 0.00112576829195875 | 0.00856608491915462 |
| MAP1LC3A | 982.65884820696 | 0.545777093168875 | 0.0860848270770177 | 6.33999174651978 | 2.29777471372397E-10 | 3.60761417729376E-08 |
| TNNC2 | 486.066430587331 | 0.789153800980001 | 0.182307311775799 | 4.32870077065532 | 0.0000149991560050028 | 0.000296608162904661 |
| SMCHD1 | 1228.67217483057 | -0.522282673938838 | 0.0742732291559932 | -7.03191014950901 | 2.03724986624057E-12 | 7.22363848209629E-10 |
| RNF125 | 285.430460025796 | -0.562368860377494 | 0.105069370630464 | -5.35235775186456 | 0.0000000868155441443 | 4.69028340431952E-06 |
| NOL4 | 9.80067610870236 | -0.716769188492369 | 0.248056506744816 | -2.8895399596582 | 0.00385805969465637 | 0.0217353828013306 |
| GPR143 | 269.396430775952 | 0.748774388006111 | 0.17060788005 | 4.3888616855603 | 0.0000113945506706177 | 0.000237714637259387 |
| PCYT1B | 9.80396681527949 | -0.505242136577339 | 0.188442637444519 | -2.68114553812744 | 0.00733705983849304 | 0.0353095344824988 |
| RUBCNL | 2999.19635115964 | 0.73791759711389 | 0.140144809910254 | 5.26539368519204 | 1.39889255582291E-07 | 6.93063182990069E-06 |
| CAB39L | 815.911640470642 | 0.578122407130811 | 0.133243945209555 | 4.33882685041777 | 0.0000143245323136275 | 0.000286912958034256 |
| FGF9 | 29.9282393552726 | -0.671684992309235 | 0.18380132896402 | -3.65440770257282 | 0.00025777637223498 | 0.00277277498883313 |
| CBLN1 | 61.3842605103183 | 1.18449281870055 | 0.205665404255185 | 5.75931972122479 | 8.44536034353795E-09 | 6.91695816353615E-07 |
| PLLP | 565.7228478509 | -0.58955704823752 | 0.115858677878308 | -5.08858774356774 | 3.60739864563523E-07 | 0.0000148022853383231 |
| HAS3 | 623.606764573618 | -0.525989725716138 | 0.132133790408274 | -3.98073591994075 | 0.0000687022407760764 | 0.000975600991946304 |
| NECAB2 | 3.78538755744133 | -0.658345821834002 | 0.164724622263514 | -3.99664490218609 | 0.0000642465619445084 | 0.000926092036443211 |
| ZP2 | 15.707151916486 | 0.816415644095663 | 0.227369276598191 | 3.59070344204173 | 0.000329786700792377 | 0.00335016003885136 |
| PRSS33 | 241.925003435673 | 0.867543134776991 | 0.216388068623147 | 4.00920041616468 | 0.0000609246927826507 | 0.00088603167883913 |
| QPRT | 2659.83684472379 | 0.719383871417572 | 0.147283955243065 | 4.88433292160278 | 1.03779598618919E-06 | 0.0000350359065415654 |
| AQP9 | 184.116273514179 | -0.742141706702869 | 0.175592443309177 | -4.22650139559897 | 0.0000237352800515991 | 0.000425641179451951 |
| AP3B2 | 13.550621166155 | 0.694476763723862 | 0.178247105439049 | 3.89614609456498 | 0.0000977354480085665 | 0.00129444310982276 |
| HOMER2 | 61.1260187711231 | -0.750410423085491 | 0.172015256130664 | -4.36246435325174 | 0.0000128605545418618 | 0.000262726124000576 |
| FAM189A1 | 121.154945238304 | 0.748281862423251 | 0.203122412946966 | 3.68389608791534 | 0.000229695909319409 | 0.00251769603391009 |
| RHOV | 243.687654434005 | -0.569166975319839 | 0.12932808471208 | -4.40095418243423 | 0.000010777588267567 | 0.00022783535914325 |
| MYEF2 | 294.008807189872 | 0.72043806605689 | 0.16021673083667 | 4.49664690007518 | 6.90334898483583E-06 | 0.000158232897019109 |
| PDGFRL | 72.3744131268565 | -0.628426874621169 | 0.147982934946127 | -4.24661718494736 | 0.0000217022295106977 | 0.000397582883210706 |
| EYA1 | 54.4802392742297 | 1.16937392303936 | 0.222056616029005 | 5.26610710345426 | 1.3934700727073E-07 | 6.91400981772661E-06 |
| CALB1 | 98.0668688746661 | -0.863528564398574 | 0.281787028958038 | -3.06447236975966 | 0.00218054315280182 | 0.0140612657377552 |
| LAPTM4B | 6017.07949325022 | 0.517727047173443 | 0.10559875193864 | 4.90277619449783 | 9.44916204050682E-07 | 0.000032644512082503 |
| DKK4 | 96.4481037265078 | -0.658080406678618 | 0.236072321902337 | -2.78762203622865 | 0.0053096451654931 | 0.0277272257377296 |
| NEFM | 4.73050460235422 | -0.701090407107627 | 0.204609314585781 | -3.42648333741277 | 0.000611451275553542 | 0.00537684816120472 |
| ADAM2 | 0.813156198384087 | -1.13387158493627 | 0.426960656102203 | -2.65568166230485 | 0.0079148290572761 | 0.0373503584669537 |
| FGL1 | 4.54450810318341 | -1.336969700465 | 0.328312150822127 | -4.0722516578113 | 0.0000465608328471824 | 0.000723217543927299 |
| KLC3 | 38.7667867762134 | -0.85708539081702 | 0.176395190974137 | -4.85889318231292 | 1.18043794991753E-06 | 0.0000386179981941529 |
| AMH | 149.337756576594 | -0.540165127772122 | 0.20294410842655 | -2.66164478466553 | 0.00777598907276309 | 0.0368990105334965 |
| RETN | 7.17696117240409 | -0.591528367911542 | 0.201596661326268 | -2.93421708484647 | 0.00334390243608071 | 0.0194932130588841 |
| FCER2 | 12.658944237443 | -0.555213951138288 | 0.205509633596901 | -2.70164440187421 | 0.00689975118230067 | 0.0336884273025117 |
| TLE6 | 46.8173375014331 | -0.607058273115837 | 0.125933171067537 | -4.82047952870398 | 1.43213553130042E-06 | 0.00004522519021506 |
| KIR3DX1 | 1.4632039737888 | -1.21123817705922 | 0.229794821364843 | -5.2709550627172 | 1.35715697624494E-07 | 6.76394092393192E-06 |
| TNNT1 | 147.194608079388 | -1.97481428480488 | 0.218063851730372 | -9.05612860239974 | 1.35162695725258E-19 | 3.76675905870341E-16 |
| APLP1 | 44.950707371603 | -0.578467762092731 | 0.116632320738924 | -4.95975522417672 | 7.05820593550573E-07 | 0.000025768616036592 |
| NKG7 | 183.38117689373 | -0.584890232444413 | 0.141003034713141 | -4.14806839891301 | 0.0000335292234858972 | 0.000556341487002453 |
| ICAM5 | 26.1831548465748 | -0.511438077475594 | 0.14746320193067 | -3.468242048047 | 0.000523875199144204 | 0.00477759323964562 |
| ATP1A3 | 119.740628476535 | -0.639184540454548 | 0.160824556485444 | -3.97442128505045 | 0.0000705505896111447 | 0.000995507518048904 |
| PTPRS | 426.312054875259 | 0.51312272229848 | 0.12626546824975 | 4.06384048949579 | 0.0000482718203945486 | 0.000740847277482558 |
| SYNGR4 | 15.3098928960198 | -0.759316444966044 | 0.185020490806136 | -4.103958656999 | 0.0000406140073250668 | 0.000646847466891464 |
| SIGLEC5 | 9.66371145662301 | -0.642751272505043 | 0.144684205478406 | -4.44244256226692 | 8.89433808831697E-06 | 0.000193901208832788 |
| HAS1 | 21.4998475452208 | -0.636257826323211 | 0.202530709179267 | -3.1415375421415 | 0.00168063260952619 | 0.0115860061281746 |
| CAPS | 573.14669000232 | 0.816507842662933 | 0.124839326399024 | 6.54046978796675 | 6.13258854746862E-11 | 1.18679830757762E-08 |
| CACNG7 | 1.55050899163012 | -1.0340705393746 | 0.250887436474004 | -4.12165134256034 | 0.0000376166220872323 | 0.000609188898712457 |
| COMP | 742.01100537028 | -0.556773173608164 | 0.211763211491482 | -2.62922520718647 | 0.00855796652463338 | 0.0394752436574882 |
| UPK1A | 8.71606566796978 | -0.944890567775669 | 0.235390618439537 | -4.01413860093314 | 0.0000596632817679996 | 0.000870912033559774 |
| ZNF85 | 67.5234708358017 | 0.519400469466089 | 0.136647049422395 | 3.80103684390982 | 0.000144091872263024 | 0.00175160792008407 |
| PON3 | 112.279049590606 | -0.654604749350944 | 0.152376509552344 | -4.29596892115498 | 0.0000173931951274191 | 0.000332378989400658 |
| ATP6V0A4 | 7.34146419967926 | -0.801432489030006 | 0.246544969690281 | -3.2506543939501 | 0.0011513973133556 | 0.00871351639584479 |
| VIPR2 | 20.3013402794005 | 0.584508874247238 | 0.143991805347993 | 4.05932040948179 | 0.000049215743039993 | 0.000749828190771501 |
| CRHR2 | 6.60925998642584 | 0.540789511478732 | 0.153366917666957 | 3.52611579932172 | 0.000421702537010474 | 0.00404665028485058 |
| EPHB6 | 87.5436328732711 | -0.591195670844968 | 0.141302926435733 | -4.18388837200659 | 0.000028656486511543 | 0.000496544156434726 |
| NPTX2 | 823.923809200409 | 0.974613013077455 | 0.229542957877416 | 4.24588504953366 | 0.0000217732242315168 | 0.00039832612951334 |
| SERPINE1 | 1344.93144103155 | -0.505143439776567 | 0.128453011875366 | -3.93251534083677 | 0.0000840616132857373 | 0.00115163720703401 |
| AGR2 | 30748.2902382807 | -0.841541639371468 | 0.126185826564271 | -6.66906626746104 | 2.57436001939937E-11 | 5.93736191508647E-09 |
| TMEM176B | 8031.18341483316 | 0.58554534560438 | 0.0922313701464717 | 6.34865712907096 | 2.17202517233169E-10 | 3.45889837205316E-08 |
| RLN2 | 16.1639735435724 | 0.593216929633281 | 0.157864702404133 | 3.75775534745347 | 0.000171444380917212 | 0.00200259971590408 |
| ELAVL2 | 32.8182295452557 | 0.670833751299167 | 0.23079190900514 | 2.90666061124451 | 0.00365309308498489 | 0.020826242575531 |
| KCNT1 | 31.1976246233434 | 0.687568551392546 | 0.219052191207767 | 3.13883439193903 | 0.00169621280372257 | 0.0116717589675083 |
| SH3GL2 | 8.37467161189454 | 0.645789628752951 | 0.236916773793277 | 2.7258079637554 | 0.00641443061188396 | 0.0318739061697806 |
| DNTT | 0.738469797385758 | -1.25706567403132 | 0.460614901390523 | -2.72910335778639 | 0.0063506795784602 | 0.0316558990107119 |
| FGF8 | 2.69198574157976 | -0.520186357761052 | 0.186212289430345 | -2.79351249776472 | 0.00521390142457699 | 0.0273511045397182 |
| PITX3 | 0.796193955725051 | -0.670261820081805 | 0.252548141846073 | -2.65399624476479 | 0.0079544712109557 | 0.0374930833314701 |
| NEURL1 | 385.44829207473 | -0.55122321349523 | 0.16901273490305 | -3.26143005621101 | 0.00110851776467998 | 0.00846564308893077 |
| DKK1 | 70.7120901186573 | -1.3357791858258 | 0.244723428881679 | -5.45832163242381 | 4.80656386716787E-08 | 2.91197660952587E-06 |
| CYP2C18 | 89.8868996409289 | -0.587389447005836 | 0.146188526483278 | -4.01802700345997 | 0.0000586874730212021 | 0.000861178794548065 |
| KRT23 | 2244.90130712533 | 0.58659325775563 | 0.212151449717247 | 2.7649740717654 | 0.00569273204966753 | 0.0292505978798062 |
| RNF43 | 9002.5653381292 | 0.558721176032269 | 0.0954024927567503 | 5.85646307436486 | 4.72828656662852E-09 | 0.0000004250627939817 |
| CCL7 | 9.33251227233695 | -0.619965802588397 | 0.194492758845974 | -3.1876035193648 | 0.00143457096183461 | 0.0102532425957839 |
| CCL1 | 0.85611003598266 | -0.986869875780608 | 0.268184619820541 | -3.67981533184485 | 0.000233402914000288 | 0.0025466428221852 |
| GABRA4 | 32.2603890766263 | 0.852470260766508 | 0.253353477141218 | 3.36474663930246 | 0.000766139755670336 | 0.00641975587800736 |
| SULT1E1 | 21.1959342949237 | -0.932610325034857 | 0.246740723844889 | -3.77971787754474 | 0.000157006155044791 | 0.00187320721976736 |
| ANXA10 | 27.1584776205784 | -1.76473975738738 | 0.307461722331554 | -5.73970556076037 | 9.48412999129353E-09 | 7.58775777915881E-07 |
| SOD3 | 3606.80181251865 | 0.67659188659507 | 0.14934049106811 | 4.53053208648214 | 5.88353178048843E-06 | 0.000140040619076935 |
| HGFAC | 4.27587348546102 | -0.601666368530257 | 0.21020218303448 | -2.86232216927815 | 0.00420549185174379 | 0.0231696966237258 |
| CRTAM | 19.8522595548412 | -0.710745643067705 | 0.161022988430111 | -4.41393896608863 | 0.000010150658716598 | 0.000216767770626098 |
| P2RX3 | 3.06898756894758 | 1.08384315219738 | 0.249514989557665 | 4.34379976176499 | 0.0000140039168613878 | 0.000281610936667787 |
| FOLR1 | 209.841088210105 | 1.01722994685976 | 0.233027058891559 | 4.36528681131889 | 0.0000126956068662857 | 0.00025967369102283 |
| KIAA1549L | 154.897179154464 | -1.51293302506236 | 0.180368720256633 | -8.38800110634326 | 4.94477562934583E-17 | 8.70332561034649E-14 |
| IL23A | 95.7234002404117 | -0.590551826972677 | 0.122971557159486 | -4.80234487237376 | 1.56818299646027E-06 | 0.0000485434123506144 |
| LIN7A | 148.209724543367 | 0.9694957215525 | 0.17347898232306 | 5.58854858709664 | 2.28975315222658E-08 | 1.63434222206681E-06 |
| OAS3 | 3526.14076981257 | -0.59878510529046 | 0.0890115717481612 | -6.72704788299427 | 1.7313977825318E-11 | 4.13581461738775E-09 |
| OAS2 | 1477.17658582414 | -0.958880842288093 | 0.134079371130952 | -7.15159113739858 | 0.0000000000008577774 | 3.54145578319955E-10 |
| FZD10 | 254.394018059818 | 0.872485063831647 | 0.252686302251749 | 3.45283878095774 | 0.000554720427892161 | 0.00500160705030187 |
| IFNG | 12.130404409951 | -0.791168121171793 | 0.186982777182009 | -4.23123526720148 | 0.0000232411421279496 | 0.000419444293061462 |
| ENO2 | 927.542629983663 | -0.61482556168596 | 0.109746873080564 | -5.60221484610884 | 2.11630011093826E-08 | 1.53188979025968E-06 |
| SLCO1B3 | 142.417550893828 | -0.866653008061359 | 0.240512227412405 | -3.60336361018068 | 0.000314125611322523 | 0.00322041345611521 |
| CEP85L | 71.4211180003137 | -0.562994893799421 | 0.105741073415539 | -5.32427821672448 | 0.000000101354678659 | 5.31982414659606E-06 |
| UST | 98.1582407102414 | -0.709695832481857 | 0.152783841974108 | -4.64509743512096 | 3.39915988945567E-06 | 0.0000897195777609918 |
| ULBP1 | 29.82083360087 | -0.577426616881571 | 0.13362438978081 | -4.32126663275131 | 0.0000155136071469805 | 0.000304106711728794 |
| IL17A | 12.4638505385084 | 0.701559010196158 | 0.221366466561544 | 3.1692198962805 | 0.00152848706100961 | 0.0107806234089117 |
| IL17F | 4.04901951792955 | 1.06401399091981 | 0.253968905489078 | 4.18954434154369 | 0.0000279515160855342 | 0.000486776845751114 |
| BMP5 | 61.1912553134538 | 0.512859192578314 | 0.179173671076535 | 2.86235800995138 | 0.00420501626375741 | 0.0231696966237258 |
| BAG2 | 413.19640458063 | -0.57667378715179 | 0.123771741413547 | -4.65917163777313 | 3.17484401358498E-06 | 0.0000850746262037733 |
| KHDRBS2 | 1.28083659765576 | -0.804591501955198 | 0.272875880589628 | -2.94856218225166 | 0.00319255905766994 | 0.0188565100682794 |
| VNN1 | 391.562511387151 | -1.45398585693016 | 0.196394062910525 | -7.40341044623421 | 1.32730684711409E-13 | 0.000000000072766878 |
| VNN2 | 94.2980786512718 | -0.743144255930278 | 0.174815989530394 | -4.25100849142334 | 0.0000212810098634106 | 0.000392109934904781 |
| EYA4 | 12.3982334243153 | -0.936289712383457 | 0.179758267782767 | -5.20860444380192 | 1.90266241104657E-07 | 8.77639122071993E-06 |
| ADGRG6 | 851.855451696737 | -0.813887425319833 | 0.12749419634523 | -6.38372136654737 | 1.72835447941159E-10 | 2.87560350748669E-08 |
| UNC93A | 149.006126472966 | -0.822015545698669 | 0.201228895555344 | -4.08497767395732 | 0.000044081054833142 | 0.000695357847042422 |
| PACRG | 10.3889869043918 | 0.679754484105139 | 0.153079715493047 | 4.44052617889804 | 8.97391866977319E-06 | 0.000194873888412049 |
| PDE10A | 134.833342347189 | -0.911538497882684 | 0.146274040668384 | -6.23171749216406 | 4.61348908809547E-10 | 6.22114121306809E-08 |
| PTK7 | 3472.68745214348 | 0.584491286776531 | 0.115753193171707 | 5.04946145122322 | 4.4305732821593E-07 | 0.0000174314390237613 |
| C7 | 264.556460520215 | -0.522230366065991 | 0.190194181692634 | -2.74577466785997 | 0.00603682108361914 | 0.0304683626137023 |
| GHR | 84.2916467875471 | 0.74043107099785 | 0.144377318626415 | 5.12844453714894 | 0.0000002921459390694 | 0.0000125395909455649 |
| NME5 | 18.222654359515 | 0.546130437550503 | 0.189619445182815 | 2.88013941304369 | 0.00397499358292514 | 0.0222127917197856 |
| GZMK | 40.9638750470789 | -0.680709789025398 | 0.16585828607494 | -4.10416509861818 | 0.0000405777616646081 | 0.000646807199994196 |
| CDH9 | 1.70043926282224 | 1.85465637205465 | 0.599105820193257 | 3.09570748529263 | 0.00196343950702632 | 0.0129742683826262 |
| PCDHB6 | 9.54525079121004 | -0.947538785422244 | 0.17559940134786 | -5.39602514672122 | 6.81334016397067E-08 | 0.0000038750292816923 |
| GABRG2 | 1.98484650517212 | -1.27515681590941 | 0.332104709111685 | -3.83962280848173 | 0.000123223479454817 | 0.00155152093370782 |
| POLR3G | 183.555915095724 | -0.500992876720316 | 0.0946230671670508 | -5.29461675381803 | 1.19266270138971E-07 | 0.0000060764620527226 |
| UNC5A | 33.9494795742439 | -0.830416690006415 | 0.200098762850133 | -4.15003410405075 | 0.0000332425741330144 | 0.000553083663759337 |
| CNTN3 | 41.4522442435226 | 1.1506704419352 | 0.190641667794228 | 6.03577620385275 | 1.5820037955057E-09 | 1.76351236431005E-07 |
| RBP2 | 172.9946533339 | 1.0000426152369 | 0.214062283437161 | 4.67173665149877 | 2.98663730278281E-06 | 0.0000808544754514181 |
| RBP1 | 758.748183748555 | 0.706221351378731 | 0.173472616255044 | 4.07108261018226 | 0.0000467951493769153 | 0.000725302973632602 |
| C3orf14 | 109.551518165702 | 0.528121651235303 | 0.156983919615974 | 3.36417674196972 | 0.000767723613887048 | 0.00642819556725355 |
| PEX5L | 2.95544231908751 | -0.815115452854254 | 0.174739536302522 | -4.66474542683383 | 3.08999338067591E-06 | 0.000082933835181833 |
| VIPR1 | 1109.63473238199 | 0.558908562739538 | 0.0920933032259382 | 6.06893816555079 | 0.000000001287587049 | 1.51617908777531E-07 |
| SLC4A3 | 90.8654814357406 | -0.537051528567068 | 0.149858945220815 | -3.583713523245 | 0.000338743605201071 | 0.00341521364037812 |
| IL1A | 88.0886120448721 | -0.514264775814987 | 0.165716611746391 | -3.10327836416307 | 0.00191389529484979 | 0.0127783992278116 |
| TFCP2L1 | 1928.73990969901 | 0.576195737401531 | 0.115481780283586 | 4.98949475827772 | 6.05374168757738E-07 | 0.000022696101963673 |
| TLX2 | 9.68500663475483 | -0.5665161242897 | 0.164394969756684 | -3.446067267923 | 0.000568808749761498 | 0.00508748387524044 |
| CCDC88A | 283.130407200479 | -0.505894503410316 | 0.109036710107985 | -4.63967138140268 | 3.48963637913033E-06 | 0.0000913148824654747 |
| REG1A | 14543.0398998089 | -1.14552793803107 | 0.285728333639726 | -4.00915066223521 | 0.0000609375294937503 | 0.00088603167883913 |
| GNLY | 236.755343835253 | -1.01430145514042 | 0.155326848719866 | -6.53011030288602 | 6.57212722546243E-11 | 0.0000000125591473528 |
| CHST10 | 75.4232124965465 | 0.536626999686002 | 0.114209250178977 | 4.69862991697306 | 2.61912559967057E-06 | 0.0000734912228827144 |
| IL1R2 | 352.435248873443 | -0.983793489012246 | 0.145471450511869 | -6.76279424966603 | 1.35355398708547E-11 | 3.4292085178873E-09 |
| SMYD1 | 11.1814296220317 | 0.74815315209142 | 0.255353402423189 | 2.9298734420289 | 0.00339100081299511 | 0.0196809873634472 |
| MLPH | 2030.18072646905 | -0.551275316246081 | 0.139894820105109 | -3.94064137494071 | 0.0000812640403802547 | 0.00111882751683675 |
| KYNU | 159.315499575086 | -0.856213709796313 | 0.126648478093628 | -6.76055269423244 | 1.3746632084609E-11 | 3.45650278325936E-09 |
| KISS1R | 13.2172108790782 | -0.76226755777017 | 0.200091944425818 | -3.80958643766277 | 0.00013919943592434 | 0.00170759419527936 |
| VAX2 | 7.21969115545122 | -0.597267202311385 | 0.189208218879995 | -3.15666626876394 | 0.00159583876800578 | 0.0111571098467237 |
| ATP6V1B1 | 17.8557681807811 | -0.531707859213763 | 0.170522656174009 | -3.11810683192259 | 0.00182016813316437 | 0.0122969823655117 |
| PAPPA2 | 15.0871737338946 | -0.543474392827104 | 0.203064473971531 | -2.67636372920306 | 0.00744258247853586 | 0.0357094466638732 |
| PRG4 | 16.9092277525841 | -0.720354543455359 | 0.147628667729742 | -4.87950311096815 | 1.06353445979811E-06 | 0.0000356023217262948 |
| PDC | 1.07825316650448 | -0.667002477858215 | 0.227019835872703 | -2.93808016948891 | 0.00330251612072532 | 0.0193115481918685 |
| RPE65 | 5.2074547073393 | -1.71415964246987 | 0.280767071332742 | -6.10527308039762 | 1.02625064016751E-09 | 1.2434736923363E-07 |
| ACTL8 | 59.5944144668723 | -1.35864354486038 | 0.204250284707142 | -6.65185630858938 | 2.89419195792201E-11 | 6.40977267926012E-09 |
| GBP1 | 1539.35273532529 | -0.556445631327699 | 0.113968851047502 | -4.88243608857453 | 1.04783206204126E-06 | 0.0000352886201599031 |
| CR2 | 98.3971998677874 | -0.892221304784197 | 0.208861353414624 | -4.2718353117869 | 0.0000193870736502382 | 0.000363420693391965 |
| F3 | 1603.094668074 | -0.550886379515971 | 0.10847801032591 | -5.07832304317615 | 3.80780856149028E-07 | 0.0000154727501717324 |
| FASLG | 27.618350224312 | -0.669377582151733 | 0.146493949709283 | -4.56931896150053 | 4.89311625412415E-06 | 0.000120497491730795 |
| MMP8 | 15.4924881469372 | -1.32388311964069 | 0.228503789751234 | -5.7937031201188 | 6.88511234051028E-09 | 5.87377364518736E-07 |
| ZNF541 | 27.4873162789405 | 0.95281037389278 | 0.196369619184425 | 4.85212721728572 | 1.22144227365585E-06 | 0.000039812351379726 |
| PLAGL1 | 798.313855669798 | 0.5177582829259 | 0.0991271652788999 | 5.22317249231488 | 1.75883408144848E-07 | 8.26979013758262E-06 |
| ARMC2 | 121.39053126266 | 0.709224031042054 | 0.102942500011569 | 6.88951629271047 | 5.59824130862247E-12 | 1.68663410669327E-09 |
| SPP1 | 5555.28983218895 | -0.596785667239162 | 0.192220371866887 | -3.10469520708469 | 0.00190475192721587 | 0.0127270157742164 |
| RARRES1 | 965.025745061732 | -0.619844056073255 | 0.159639080993552 | -3.88278391616582 | 0.000103267330779554 | 0.00134795709443008 |
| FGF23 | 3.02099991034748 | -1.12386222014945 | 0.363759503391169 | -3.08957486930838 | 0.00200443179830097 | 0.0132002095923816 |
| GDA | 1056.26257076448 | -0.659398054299112 | 0.106656388164496 | -6.18245250609951 | 6.31133025134642E-10 | 8.14917012608212E-08 |
| CSF3R | 309.29370708552 | -0.560771200778673 | 0.144456886203626 | -3.88192778839364 | 0.000103631653658967 | 0.00135181360418807 |
| ONECUT2 | 171.164457277449 | -0.621475557887168 | 0.154392605412106 | -4.02529354452125 | 0.0000569042863541788 | 0.000840544675024932 |
| TGFB3 | 342.559161270712 | -0.605145583422656 | 0.136998002952044 | -4.41718543615915 | 9.99944399467141E-06 | 0.000214085407214982 |
| ESRRB | 3.37065335733133 | -0.549808208440338 | 0.193493386737425 | -2.8414832036944 | 0.00449042172912361 | 0.0244216431070665 |
| TECTB | 1.6989739678943 | 0.531668135298033 | 0.195771929984673 | 2.71575263797857 | 0.00661253034354409 | 0.0326304027960457 |
| ELOVL3 | 9.26436258938932 | -0.696972579680371 | 0.167251838507412 | -4.1672042944358 | 0.0000308358127657923 | 0.000525337009984067 |
| IFIT3 | 880.74434299972 | -0.564217467012268 | 0.119165709624377 | -4.73473005607688 | 2.19346842543962E-06 | 0.0000636992594131662 |
| IFIT2 | 307.356117705998 | -0.588967410532403 | 0.115055604063537 | -5.11898064701967 | 3.07191478988583E-07 | 0.000013020402332492 |
| PRLHR | 0.855585567615591 | -0.879444908873003 | 0.308350514086644 | -2.85209483589798 | 0.00434321407927827 | 0.023771811004783 |
| CPN1 | 27.5230433888629 | 1.2008099101445 | 0.292761726781115 | 4.10166288929664 | 0.0000410191591448932 | 0.000651978479146159 |
| INSL4 | 2.26004425531891 | -1.83808655766721 | 0.45052286568606 | -4.07989626645955 | 0.0000450558024099135 | 0.00070771401129486 |
| CD274 | 145.734928781219 | -0.903264688849974 | 0.128616289383926 | -7.02294159765161 | 2.17245037578107E-12 | 7.567821402799E-10 |
| PCDHB8 | 19.4856399891343 | -1.16971748900326 | 0.224974770136173 | -5.19932741033693 | 2.00010944255228E-07 | 9.15015868369815E-06 |
| TP53AIP1 | 5.47131216262623 | -0.774055654482461 | 0.186157820579499 | -4.15806143450149 | 0.000032095971700338 | 0.000538347666271318 |
| SOHLH2 | 1.83348682893891 | -0.944331616750339 | 0.34028946960174 | -2.77508327793846 | 0.00551875743040909 | 0.028573817307283 |
| TGFBI | 32980.7967032547 | 0.512691840450885 | 0.106602679176755 | 4.80937106281172 | 1.51405920203857E-06 | 0.0000471006212414639 |
| DUSP4 | 1825.94919934212 | -0.950355119675226 | 0.164856395977514 | -5.76474521379717 | 8.17812930631961E-09 | 6.73628079462907E-07 |
| PRB2 | 1.78965488476215 | -1.04772613433602 | 0.341969648116763 | -3.06379861518668 | 0.00218545972488919 | 0.0140875374170671 |
| ZSCAN18 | 236.246069700769 | 0.609560403259156 | 0.130051201639213 | 4.68708013133315 | 2.77130480220935E-06 | 0.000076413917443364 |
| RGSL1 | 0.740196830249009 | 1.19816985208902 | 0.367075936060146 | 3.26409261513862 | 0.00109815262033548 | 0.00839991306707666 |
| DPPA4 | 3.39346906808143 | -0.731102387268663 | 0.255371789742459 | -2.86289408867743 | 0.0041979085843113 | 0.0231431682948464 |
| GJB6 | 8.11950804836033 | -1.2356733957808 | 0.234778560895442 | -5.26314409232238 | 1.4161250313807E-07 | 6.98496361348577E-06 |
| GJA3 | 25.1374099173379 | -1.13708361763786 | 0.200119115281031 | -5.68203400280393 | 1.33102189203935E-08 | 1.02320181353713E-06 |
| FABP3 | 249.835980303462 | 0.521019183583576 | 0.169699553755971 | 3.07024486542142 | 0.00213883337140345 | 0.0138617956601694 |
| CXCR4 | 1185.97026524622 | -0.578227845909252 | 0.113529064411274 | -5.09321422587035 | 3.52043528220488E-07 | 0.0000145525830293567 |
| SV2C | 28.811514523766 | 1.01397144485345 | 0.205437221082134 | 4.93567543170801 | 7.9873845694017E-07 | 0.0000284163951882906 |
| PAEP | 13.3980322000591 | -1.48903966413739 | 0.207763527614756 | -7.16699259601729 | 7.66631765809538E-13 | 0.0000000003204712439 |
| PLG | 0.884658901806665 | 0.929133902892222 | 0.335934044906428 | 2.76582238978197 | 0.00567794501524428 | 0.0291946244157133 |
| SPINK4 | 4293.79439053018 | -0.716282309616966 | 0.246405827737461 | -2.90692113978793 | 0.00365005202997402 | 0.0208160027261922 |
| CNTFR | 39.9679974806764 | -0.739588723594128 | 0.239339462501583 | -3.09012444443523 | 0.00200072648140544 | 0.013181303189748 |
| SFTPA1 | 1.96655690865664 | -0.952067225476128 | 0.358555550693454 | -2.65528513959639 | 0.00792413958666774 | 0.0373659159697324 |
| HOXC13 | 2.70269684229542 | -2.09185970764985 | 0.395981849541455 | -5.28271613982361 | 1.27282529921483E-07 | 6.40087573779587E-06 |
| HOXC12 | 5.31256563896469 | -1.65260607940355 | 0.428471304449274 | -3.85698193144506 | 0.000114795644370784 | 0.00147144344156679 |
| SERPINA7 | 29.621755931147 | 1.60314013576606 | 0.274749675430374 | 5.83491184568234 | 5.38190139664937E-09 | 0.0000004723925105164 |
| TNFAIP6 | 145.887872135484 | -0.654852117382107 | 0.145586964341157 | -4.49801340625234 | 6.85913731866667E-06 | 0.000157327345823629 |
| GPR83 | 7.52840950406458 | 0.69714551318251 | 0.171403541627831 | 4.06727601169539 | 0.0000475658919811517 | 0.000732463739401182 |
| CDH26 | 21.9028602572778 | 0.780651454012874 | 0.161477165884081 | 4.83443866344099 | 1.33521864789159E-06 | 0.000042885595485563 |
| MOCS3 | 743.616682253426 | 0.613523728615074 | 0.0659415149006208 | 9.30405874872157 | 1.35184356064163E-20 | 4.52083523549775E-17 |
| PMEPA1 | 6609.30688951645 | 0.536654440977497 | 0.114100498497529 | 4.70334878501095 | 2.55928661950161E-06 | 0.0000723479823578807 |
| SEMG1 | 50.7571892583177 | -1.33218344673244 | 0.298971715334401 | -4.45588454828371 | 8.35479879039833E-06 | 0.000184423221880199 |
| C20orf85 | 1.58361844443335 | 0.837816182046333 | 0.321344123424693 | 2.60722422155224 | 0.00912795659218795 | 0.0414244978091938 |
| NEURL2 | 31.6431723257616 | 0.592294405290894 | 0.0939317401293914 | 6.30558322964108 | 2.87110664763932E-10 | 4.24847559780329E-08 |
| LYPD3 | 142.122534733905 | -0.539079110460425 | 0.14384161081872 | -3.74772715205346 | 0.000178444211650549 | 0.00206918561928491 |
| GRM4 | 24.1114660964203 | 1.65947861314065 | 0.22829551064755 | 7.2689936321289 | 3.62175473053321E-13 | 1.75534379273176E-10 |
| BTN1A1 | 20.1719035495565 | -1.1190015935752 | 0.180624583566733 | -6.19517881496887 | 5.82188967465642E-10 | 7.63512292156313E-08 |
| OR2B6 | 2.8285126312707 | -0.826568430975819 | 0.182812304321225 | -4.52140480393175 | 6.14305850042219E-06 | 0.000144266967957246 |
| SPDEF | 807.606962101788 | -0.656081320604192 | 0.176678106647064 | -3.71342739094886 | 0.000204471147747802 | 0.00229834124332229 |
| TCP11 | 24.9171022302837 | 0.84130586011762 | 0.298465023934026 | 2.81877537618473 | 0.0048207240343439 | 0.0257398706175406 |
| TREM1 | 149.011303315364 | -0.681624244038766 | 0.158916473438107 | -4.28919815102891 | 0.0000179319338025756 | 0.000340533634426878 |
| KCNK17 | 22.9394920930638 | 1.18884483509199 | 0.164799847336592 | 7.21387097321671 | 5.43832891774992E-13 | 2.45768372523504E-10 |
| EREG | 1789.91146920764 | 0.547578509812325 | 0.187721982048848 | 2.91696531133917 | 0.00353455079610138 | 0.0203201732376178 |
| MYRF-AS1 | 7.48169210995661 | -0.808742040899703 | 0.187263708665587 | -4.31873344099974 | 0.0000156927178089414 | 0.000306846965111608 |
| MYRF | 1506.3854043283 | -0.69046171444436 | 0.133541480903921 | -5.17039132538247 | 2.33604292683783E-07 | 0.0000103885568562913 |
| MT2A | 2035.22294130172 | -0.583545539419185 | 0.122336501668017 | -4.7700034859812 | 1.84222731132716E-06 | 0.0000557032240012685 |
| PIWIL1 | 295.304033298265 | -1.19914953934245 | 0.228490537375334 | -5.24813654480864 | 1.53645404561322E-07 | 7.44668060773874E-06 |
| KIF25 | 18.9354267984501 | 0.922030877144717 | 0.175581447103272 | 5.25130013652527 | 1.51029350879078E-07 | 7.34116795363098E-06 |
| AMELX | 11.0752076772733 | 0.714792895915473 | 0.206848163488581 | 3.45564052327172 | 0.000548986944927164 | 0.00496329316362645 |
| KIR2DL1 | 1.01002495133905 | -1.14897808122114 | 0.309953263158286 | -3.7069397802545 | 0.000209778799492281 | 0.00234551073641621 |
| IL1B | 763.280507630599 | -0.763498150607987 | 0.150944083582757 | -5.05815221428925 | 4.23338470362379E-07 | 0.0000168338705420436 |
| TNFSF9 | 357.604580075562 | -0.801385570678216 | 0.165871017177804 | -4.83137792432526 | 1.35591346052493E-06 | 0.0000433918257864828 |
| GRIA3 | 7.14933635291717 | -0.524472695864435 | 0.155562971763586 | -3.37144945174674 | 0.000747737591526707 | 0.00630346370956293 |
| BANF2 | 0.950422236691772 | 0.864077088023191 | 0.31842388933044 | 2.71360634982543 | 0.0066555201350778 | 0.0327700094754523 |
| CITED1 | 59.3169573836908 | -1.02446249115131 | 0.162645527034999 | -6.29874371479555 | 3.00067643383453E-10 | 4.36298353479541E-08 |
| MMP24 | 98.7801337432826 | 0.535918024936128 | 0.12506495251414 | 4.28511756621454 | 0.0000182642577790779 | 0.000345666841340081 |
| ROMO1 | 3475.81593881105 | 0.698032847963442 | 0.104864120070269 | 6.65654608550277 | 0.0000000000280337259 | 6.28674387812247E-09 |
| PLAGL2 | 3070.06897892003 | 0.51843487170871 | 0.0883293683034699 | 5.86933747706135 | 4.37540016737578E-09 | 4.01983880212584E-07 |
| GRPR | 25.5109542318541 | 0.982174647611032 | 0.218729147444198 | 4.49036929502779 | 7.10997939771502E-06 | 0.000162301659398215 |
| F10 | 228.359017623902 | 0.656647764526984 | 0.169686714222739 | 3.86976533510476 | 0.000108940160894495 | 0.00140574900037145 |
| FFAR2 | 70.5370953636957 | -0.625698046062535 | 0.1551486900924 | -4.0328928699939 | 0.0000550944000372084 | 0.000819967479325467 |
| PRDX5 | 14602.0136916746 | 0.561442549064637 | 0.0892192172344472 | 6.2928432513513 | 3.11703060416561E-10 | 4.49309213209079E-08 |
| FLRT1 | 34.4753962655507 | 0.756300427271727 | 0.136685971603039 | 5.53312398047813 | 3.14576945892314E-08 | 2.12275989168522E-06 |
| PRKCG | 80.7788632095435 | -1.05672382488347 | 0.205493532563977 | -5.14237023276867 | 2.71293822779782E-07 | 0.0000118441357981742 |
| SIX1 | 27.7218477716623 | -0.936464098168142 | 0.163453133936815 | -5.72925141056118 | 1.00874796945718E-08 | 8.03203561775885E-07 |
| HSPA2 | 748.648766839391 | -0.50286778680659 | 0.135758905158616 | -3.70412376425 | 0.000212122681182386 | 0.00236460223470046 |
| CTAG2 | 1.47284257720131 | -1.67992972452266 | 0.608303802940467 | -2.76166237396853 | 0.0057507913057619 | 0.0294740172946038 |
| AVPR2 | 22.961069766069 | 0.731633057921764 | 0.165932664428354 | 4.4092165966374 | 0.0000103745225041563 | 0.000220983937314646 |
| MASP1 | 88.8825720898283 | 0.506976363370507 | 0.188218441966231 | 2.6935530762786 | 0.00706948885517832 | 0.0342883025808373 |
| PLAAT1 | 7.51630320956301 | -0.998052719969083 | 0.26256776906979 | -3.8011242716687 | 0.000144041032943235 | 0.00175160792008407 |
| IL22 | 2.59191267814331 | 0.837835310637699 | 0.283962914058823 | 2.95050962346492 | 0.00317250161210871 | 0.0187611910485879 |
| BEST3 | 16.7591981417602 | 0.990326517550789 | 0.244911871313857 | 4.04360357151359 | 0.0000526358884244882 | 0.000792001440349195 |
| TRPV5 | 1.3562412139235 | -0.796036728754604 | 0.28139137753824 | -2.8289307786143 | 0.00467037994188929 | 0.0251280536008388 |
| FGFRL1 | 3483.54051559877 | 0.902985410693757 | 0.127778400878381 | 7.06680788369873 | 1.58538195022801E-12 | 5.70089711607796E-10 |
| FBXL16 | 273.967136081595 | -0.534891176172925 | 0.150523972186755 | -3.55352817496262 | 0.000380100479431133 | 0.00374192529677243 |
| GNG13 | 8.34298975543905 | 0.83958904912061 | 0.214471857769086 | 3.91468166431683 | 0.0000905235603347238 | 0.0012201889982724 |
| STEAP4 | 142.56701731796 | -0.716987042970117 | 0.142240069118572 | -5.04068261083615 | 4.63874248913974E-07 | 0.0000181225264394639 |
| APOBEC3A | 24.3544099636593 | -0.592137240885997 | 0.156780206387773 | -3.77686223617689 | 0.000158816517406861 | 0.00188874181192043 |
| STRIP2 | 248.829569007966 | -0.625133571727431 | 0.0939247576822772 | -6.65568469010156 | 0.0000000000281984206 | 6.28674387812247E-09 |
| FEZF1 | 79.0288233993315 | -0.650077628132107 | 0.245649439307698 | -2.64636316681279 | 0.00813624145203797 | 0.0380335737544106 |
| HOXD1 | 18.9910174223197 | -0.675214870222531 | 0.16644964786448 | -4.05657133484762 | 0.0000497983577443483 | 0.000756636383319626 |
| HOXD10 | 133.063571751868 | 0.523396222920832 | 0.193058359043751 | 2.7110777565566 | 0.00670648971797199 | 0.0329724241617788 |
| SNRPN | 393.030444416273 | 0.514602471855441 | 0.125729430612528 | 4.09293567423637 | 0.0000425945913292331 | 0.000673179736877227 |
| ALDH1A2 | 66.9631517411958 | -1.26593610948846 | 0.208940743735693 | -6.05882838767842 | 0.0000000013711658306 | 0.0000001581190610583 |
| ACKR4 | 23.9549624024437 | -0.810406120774028 | 0.162629500042203 | -4.98314340610851 | 6.25595500023843E-07 | 0.0000233234835137094 |
| AIPL1 | 0.756526888968624 | -1.10538817942215 | 0.396084446535211 | -2.79078915895751 | 0.00525797108319784 | 0.0275520321159985 |
| CD68 | 31.6341071493309 | -0.573942479616943 | 0.132528506982036 | -4.33070961626951 | 0.0000148629581160612 | 0.000294808449179903 |
| KLK10 | 1910.73980232206 | -1.13082585784639 | 0.189699716959236 | -5.9611362419133 | 2.50490004984195E-09 | 2.55722264845166E-07 |
| KLK8 | 103.163497449347 | -1.25938333861499 | 0.250184273944097 | -5.03382294482824 | 4.80793768705233E-07 | 0.0000186744543705463 |
| TNNI3 | 57.6739748311161 | -0.572754570953736 | 0.189898837806824 | -3.01610361373762 | 0.00256045748939557 | 0.0159092586143384 |
| GALNT8 | 288.803359149942 | -0.605240732273046 | 0.177567227481771 | -3.40851597930805 | 0.000653172596153922 | 0.0056568107790112 |
| FAM155B | 105.414393974336 | 0.928694228523741 | 0.232563922006702 | 3.99328589108064 | 0.0000651639206741036 | 0.000935685631250912 |
| CNN1 | 1366.6736679167 | 0.514305121158314 | 0.170004482904393 | 3.02524446633298 | 0.00248432233974117 | 0.0155669304263864 |
| ATP8B3 | 40.7088226495499 | -0.900859908044482 | 0.135825630547861 | -6.63247359434891 | 3.30107409691196E-11 | 7.12222709347934E-09 |
| BST2 | 2862.03136842117 | -0.687559084226395 | 0.14479953934015 | -4.74835132321272 | 2.05081564794252E-06 | 0.0000605477556478915 |
| UNC13A | 38.8072988209805 | -1.01420804878792 | 0.147416929803591 | -6.87986142527312 | 5.99108107657644E-12 | 1.77249263380917E-09 |
| SULT4A1 | 23.5109114977147 | 1.08895951990321 | 0.272625515033989 | 3.99434190804716 | 0.0000648741906842593 | 0.000933128036500214 |
| PNCK | 57.7621977017298 | 0.561445647291772 | 0.186508007526351 | 3.01030317538751 | 0.00260987038310598 | 0.0161299732677565 |
| SLC7A10 | 6.02729560885416 | 0.865195202794654 | 0.242206475221218 | 3.57213902726768 | 0.000354077345383896 | 0.00354310430410779 |
| LRP3 | 1286.14493910534 | 0.53549577973305 | 0.102904285135704 | 5.2038239129388 | 1.95229004028767E-07 | 8.96819828671705E-06 |
| RGN | 39.2447385619612 | 0.773020747236346 | 0.195373364115512 | 3.95663324289848 | 0.0000760134976515143 | 0.00105566585899582 |
| ULBP2 | 75.087070753467 | -1.0691628009095 | 0.154144080071242 | -6.93612625548357 | 4.02997467921062E-12 | 0.000000001321278561 |
| AKAP12 | 876.074897156056 | -0.743263793180609 | 0.152001756931073 | -4.88983685575196 | 1.00919583880609E-06 | 0.0000343331914967988 |
| GGT7 | 913.436039349885 | 0.758021061261041 | 0.111206190149363 | 6.81635671757955 | 9.3378267112732E-12 | 2.51835161998708E-09 |
| EDA2R | 60.8379715910991 | 0.567236890552039 | 0.16115797437414 | 3.51975688919468 | 0.000431942519483737 | 0.00411304719150773 |
| NFATC1 | 228.395797765637 | -0.632347118570134 | 0.118386708887877 | -5.34136918333478 | 9.22471728959769E-08 | 4.93588792957961E-06 |
| IDO1 | 624.691919722101 | -0.956242938349277 | 0.188469856618741 | -5.07371818233872 | 3.90116697536649E-07 | 0.000015756379950508 |
| C3orf20 | 3.13226832699946 | -0.547362871598798 | 0.169119821395378 | -3.23653884614237 | 0.00120988757786187 | 0.00902141814467261 |
| GALNT15 | 74.2565028016574 | -0.623314855389246 | 0.140823981129741 | -4.42619822553508 | 9.59083966443453E-06 | 0.000205996698816968 |
| SLC6A6 | 3902.40217154658 | 0.647812900303672 | 0.0903665326896329 | 7.16872586589782 | 7.56989628266493E-13 | 3.20446166436558E-10 |
| GFPT2 | 223.91527645764 | -0.614493751050144 | 0.147301960136149 | -4.17166038036546 | 0.0000302388107205166 | 0.000518322044139168 |
| AOC2 | 17.0667600928866 | -0.533965215636655 | 0.100471932204831 | -5.31457098434281 | 1.06908901710481E-07 | 5.56026048367326E-06 |
| ANO1 | 1239.69946811931 | -0.860346628640505 | 0.134810684139195 | -6.38188756428371 | 1.74918377160999E-10 | 2.88158638867889E-08 |
| KRT34 | 1.64039928049499 | -1.03588857035717 | 0.347810753194675 | -2.97831093731991 | 0.00289841783095509 | 0.0175025079636692 |
| RAI2 | 228.050092014662 | 0.656553817824056 | 0.129422452632981 | 5.07295144286848 | 3.91692474627891E-07 | 0.0000158009405747961 |
| RTBDN | 8.1257890379837 | -1.23449807634417 | 0.221734317731733 | -5.56746510406087 | 2.58471818821644E-08 | 1.78650176558209E-06 |
| FCRLA | 27.0129372081314 | -0.77549958244088 | 0.179944369115253 | -4.30966296002392 | 0.0000163503539596211 | 0.000316978862097187 |
| LINC00470 | 3.68132677707674 | -1.00237842559058 | 0.381141535464783 | -2.62993752273217 | 0.00854005614002555 | 0.0394252564100959 |
| RAMP1 | 628.02879692816 | -0.972069205118758 | 0.182742911034594 | -5.31932647682701 | 1.04152094336206E-07 | 5.42531828472182E-06 |
| POPDC3 | 11.3538545749984 | -0.533681655031842 | 0.195505583970171 | -2.72975146895686 | 0.00633820885219727 | 0.0316069740911792 |
| XAF1 | 268.267033781024 | -0.55293179866612 | 0.116764059637461 | -4.73546226795223 | 2.18556365450961E-06 | 0.0000635561910731396 |
| RHBG | 5.34033234344203 | -0.777003955475376 | 0.207076765801057 | -3.75225077748153 | 0.000175254022518742 | 0.00203997734117621 |
| FCRL2 | 25.2218380360877 | -0.577263775812893 | 0.178759984960912 | -3.2292673102379 | 0.00124107831388261 | 0.00918639685100978 |
| ZSWIM3 | 303.683533227653 | 0.548250269992236 | 0.0802308863922963 | 6.83340661739066 | 8.29214824035725E-12 | 2.29178530127295E-09 |
| VSTM2L | 137.294965870178 | 1.01870312281391 | 0.207442696587128 | 4.91076880301759 | 9.07200007078787E-07 | 0.0000316236816778653 |
| AL139352.1 | 11.1912546950615 | -0.707492596134901 | 0.187347999248585 | -3.77635522649033 | 0.000159139987746085 | 0.00189181647263477 |
| KANK4 | 38.8249330440541 | -0.75627262281501 | 0.16094894157701 | -4.69883563945744 | 2.61648911306304E-06 | 0.0000734912228827144 |
| NMUR2 | 21.1045687967286 | -1.97931947655216 | 0.302634258125133 | -6.5403021086058 | 6.13946854885856E-11 | 1.18679830757762E-08 |
| PDE6A | 31.2575139020819 | 0.817489385308504 | 0.167957610916425 | 4.86723632735692 | 1.13169669772595E-06 | 0.000037434422319833 |
| MTUS2 | 11.4677627319565 | -0.717666118720597 | 0.167256400873226 | -4.29081407332542 | 0.0000178019316832623 | 0.000338641751622103 |
| POSTN | 5542.87388408678 | -0.576762394188113 | 0.150157893288024 | -3.84103946558308 | 0.000122514413408635 | 0.00154688300856199 |
| STOML3 | 14.2788114496485 | 0.56943702836663 | 0.174887916477334 | 3.25601127760266 | 0.00112989263491013 | 0.00857794994248909 |
| MORC4 | 1289.6138453588 | -0.540618601851551 | 0.078261544507172 | -6.90784478195428 | 0.0000000000049207194 | 1.53793175990547E-09 |
| BEX2 | 145.350823066912 | 1.2493983622033 | 0.200189337142883 | 6.24108346645631 | 4.3455027586306E-10 | 5.95583210057887E-08 |
| PLAAT4 | 1307.48991717448 | -0.745466770358881 | 0.148028143136546 | -5.03598001409259 | 4.75410165178075E-07 | 0.0000184868217952153 |
| PLAAT2 | 42.3758994526274 | -0.563166891870154 | 0.156637730817062 | -3.59534633790042 | 0.000323960302780967 | 0.0033040196540412 |
| MYO18B | 12.7172802554003 | 0.564041371339943 | 0.21494703439882 | 2.6240946888032 | 0.00868796280776158 | 0.0399426522157221 |
| GGT2 | 0.789807274564339 | -1.03736039645037 | 0.333391480503839 | -3.11153840788809 | 0.00186115261584392 | 0.0125142622946703 |
| SEC14L4 | 22.0899676625569 | 1.27330030741182 | 0.26100029223988 | 4.87853977665879 | 1.06874112820073E-06 | 0.0000357051356736153 |
| TEX15 | 0.792760404325333 | -1.5370109599663 | 0.423455417008593 | -3.62968779765336 | 0.000283764220330699 | 0.00298698239102904 |
| VRTN | 13.5182243963118 | -1.16079144588381 | 0.23879580494782 | -4.86102109765896 | 1.16781805461068E-06 | 0.0000383882090114355 |
| CNTN6 | 1.00640564418082 | -0.669232363338713 | 0.243092409053594 | -2.75299572678622 | 0.00590526670350067 | 0.0300127551821382 |
| MEIS2 | 150.243336062692 | -0.639179186258993 | 0.132472799798295 | -4.82498435325754 | 1.40014259030626E-06 | 0.0000444246380503054 |
| TRPM1 | 1.49922771790251 | -1.35512434349977 | 0.332723902891482 | -4.07281933075227 | 0.0000464474537660835 | 0.000722463138997844 |
| REG4 | 21656.8066895156 | -0.907409894003929 | 0.253937623451429 | -3.57335743191867 | 0.000352433164959391 | 0.0035319358413461 |
| VAV3 | 2795.39233233555 | 0.813566904029135 | 0.132227652266953 | 6.15277432580163 | 7.61391126208772E-10 | 9.60846869534859E-08 |
| HMGCS2 | 6302.1718345241 | 0.669847897134971 | 0.187983514285711 | 3.56333319802123 | 0.000366175407119239 | 0.00363479903973927 |
| SPIRE1 | 296.143983354526 | -0.603717977697181 | 0.104307030588874 | -5.78789343622227 | 7.12746018073952E-09 | 6.00394265401237E-07 |
| GRHL1 | 200.85165169991 | -0.583920054917162 | 0.0947476528374585 | -6.16289731122826 | 7.14258297729739E-10 | 9.08221520634142E-08 |
| FST | 77.4512925521104 | -0.610037477529263 | 0.137369907287965 | -4.44083780482183 | 8.96093179477496E-06 | 0.000194718311293609 |
| ECHDC3 | 198.838580614864 | 0.588237245095133 | 0.209415466950693 | 2.80894842038403 | 0.00497036091620539 | 0.0263546551069828 |
| KCTD1 | 164.463876392143 | -0.848115722756016 | 0.103602430550638 | -8.18625314337085 | 2.69484591649653E-16 | 0.0000000000003918306 |
| SLCO1B1 | 4.70358364742583 | -0.820299618356488 | 0.308885730476273 | -2.65567340094236 | 0.00791502293775375 | 0.0373503584669537 |
| KLRD1 | 90.4017893110679 | -0.784486799648181 | 0.145428164031314 | -5.39432512865434 | 6.87815757600226E-08 | 0.0000039052520485003 |
| KLRC1 | 9.77844499742035 | -0.872140231687116 | 0.167317905405016 | -5.21247400017337 | 1.86338690814244E-07 | 8.65491458084717E-06 |
| LRP4 | 3221.82594577327 | 0.748053926798961 | 0.154567721069066 | 4.83965165317216 | 1.30066907564161E-06 | 0.0000419046004119525 |
| SPOCD1 | 92.4256797233252 | -0.518249246680056 | 0.112751038974344 | -4.59640329166263 | 4.29846341391512E-06 | 0.000108244889674811 |
| TCN1 | 781.863737565213 | -0.70252721478376 | 0.247480170077893 | -2.83872123799916 | 0.00452947077702865 | 0.0245787506339753 |
| CLDN10 | 20.6806780981901 | 1.11260602614766 | 0.31117864865963 | 3.57545747736901 | 0.000349616000827587 | 0.00350790828073093 |
| ADAMTS8 | 58.4669196486621 | 0.541031819329242 | 0.143753675250296 | 3.7636033888332 | 0.000167482436981483 | 0.0019686986493971 |
| ANXA1 | 2740.58874733856 | -0.880582764574279 | 0.113145642508929 | -7.78273687830965 | 7.09721457629677E-15 | 0.0000000000058020515 |
| MSI1 | 152.163080028478 | 0.596807283484021 | 0.179899997134505 | 3.31743909388619 | 0.000908467201782894 | 0.00728734952315269 |
| OASL | 502.088236949487 | -0.772628051033668 | 0.11184363787204 | -6.90810908634453 | 4.91156254343438E-12 | 1.53793175990547E-09 |
| HRK | 4.0122076916737 | -1.21337144722149 | 0.223635834708095 | -5.42565751506356 | 5.77415828115093E-08 | 3.37586365801135E-06 |
| UGT2A3 | 403.493096450825 | 0.572986963805439 | 0.217450031409951 | 2.63502819516823 | 0.00841302944528428 | 0.0390219876157 |
| ELF5 | 101.813628842205 | 1.60487626336326 | 0.327520259012088 | 4.900082419952 | 9.57964596298051E-07 | 0.0000329930504937172 |
| KRT7 | 299.421913892566 | -0.684764393959999 | 0.157519825239674 | -4.3471632406784 | 0.0000137909580601706 | 0.000277662383773765 |
| MIP | 6.41234840238714 | 0.508485501666871 | 0.108650126340407 | 4.68002678684198 | 2.86837437829617E-06 | 0.000078433504463598 |
| EMX1 | 43.0908092495699 | -1.38135686515319 | 0.251084239067226 | -5.50156740337392 | 0.0000000376429649573 | 2.41623039175041E-06 |
| CHST5 | 126.993653596666 | -0.599950275646516 | 0.18985588600542 | -3.16002989567249 | 0.00157752951278052 | 0.0110599039761858 |
| SLC19A3 | 317.766499116366 | 0.632619562774258 | 0.164445205900005 | 3.8469930413109 | 0.000119576364290912 | 0.00151759877594561 |
| ALDH1L2 | 256.700413524455 | -0.50085786191479 | 0.118647374470193 | -4.22139861207479 | 0.0000242791155670584 | 0.000433961615603189 |
| PCDH8 | 6.13275493744129 | 1.25604558483698 | 0.27074688746533 | 4.63918753266489 | 3.49781549136374E-06 | 0.0000914573461002237 |
| CNMD | 0.966505692916826 | 0.726999485764829 | 0.251982237206011 | 2.88512195869767 | 0.00391262057679309 | 0.0219356005581081 |
| SCEL | 87.2713683184881 | -1.76220020207511 | 0.218359179256471 | -8.07019062846604 | 7.01884948335175E-16 | 7.5717536910403E-13 |
| IGF2BP3 | 140.244590515879 | -5.27843801812471 | 0.15010763310563 | -35.1643544629758 | 7.01475966396025E-271 | 2.34587592682159E-266 |
| IL6 | 144.646707032848 | -0.612026940025422 | 0.171832569065976 | -3.56176331036772 | 0.000368372410107919 | 0.00365010670780119 |
| NKX2-8 | 2.47204518714411 | -1.5311047293588 | 0.455558336985288 | -3.36094108054541 | 0.000776773938016738 | 0.00649421850878894 |
| ALPK3 | 712.946711033072 | 0.691281142332167 | 0.173529236106141 | 3.98365807309459 | 0.0000678624953698442 | 0.000966961043953272 |
| TBR1 | 0.782431845556291 | -0.837371784732015 | 0.302852742879882 | -2.76494700615651 | 0.00569320440164042 | 0.0292505978798062 |
| ERMN | 22.4942078069714 | -0.874127194619437 | 0.146563803605967 | -5.96414103013801 | 2.45924268977033E-09 | 2.5461917656749E-07 |
| BLK | 28.1090558058454 | -0.629850006176501 | 0.185969187553288 | -3.38685141588858 | 0.000706996666397062 | 0.00604226489078726 |
| IL1RN | 682.618633338752 | -0.886942573317494 | 0.140047387085896 | -6.33316045213684 | 2.40189549084142E-10 | 0.0000000366777118743 |
| TMOD1 | 47.4382331177026 | -0.704843294932752 | 0.145980862491887 | -4.82832669228767 | 1.37685085275377E-06 | 0.0000438520440169442 |
| HEMGN | 1.51390787758534 | -1.25268766656437 | 0.365903899273827 | -3.4235428183478 | 0.000618105132104291 | 0.00542394957434576 |
| SIT1 | 90.4847074814868 | 0.525844453267601 | 0.127496520000005 | 4.1243827930957 | 0.0000371730225963019 | 0.000603173324437422 |
| DMRT1 | 2.87208656236993 | 1.32448425403828 | 0.338937022923372 | 3.90775915423594 | 0.0000931560982343993 | 0.00124812749886009 |
| KLC4 | 2054.10674399156 | 0.503486986340418 | 0.0739233426633649 | 6.810933707817 | 9.69673103744114E-12 | 2.59422463483285E-09 |
| TFAP2A | 236.577423635416 | -0.806852271704928 | 0.168394058488416 | -4.79145332648677 | 1.65577540991056E-06 | 0.000050753841666571 |
| GCM1 | 4.79623696565618 | 0.786234629867819 | 0.235395634649136 | 3.34005611888141 | 0.000837614620867252 | 0.00687456113941974 |
| TTC29 | 9.11658584027212 | -2.78309888841327 | 0.421276934652883 | -6.60634053157088 | 3.93936175715513E-11 | 0.0000000083910914575 |
| PI15 | 89.1326390831981 | -0.673192387276954 | 0.174758799156359 | -3.85212298623452 | 0.000117098181486609 | 0.00149237705231524 |
| TTPA | 166.488035875937 | 0.572763215561896 | 0.144650475816135 | 3.95963589010199 | 0.0000750641221585802 | 0.00104595598884468 |
| NXPE4 | 807.323979337783 | 0.67719014665472 | 0.211830943832041 | 3.19684241784647 | 0.00138940808060743 | 0.0100009868772436 |
| MMP7 | 2509.15277101336 | -0.556719850009925 | 0.189474986367119 | -2.93822345990963 | 0.00330099002280978 | 0.0193060000599519 |
| SLC28A2 | 294.553309881655 | -0.978278855329995 | 0.244566196654981 | -4.00005752516196 | 0.000063327088226742 | 0.000914414716959718 |
| STRA6 | 449.688477211704 | 0.667510542703424 | 0.176124479625341 | 3.78999298747897 | 0.000150651537756809 | 0.00181421992281714 |
| IFI44L | 415.474140809509 | -0.809697286468806 | 0.169253767601 | -4.78392474179714 | 1.71905070061569E-06 | 0.0000525969748673285 |
| IFI44 | 417.929967914906 | -0.557640619283783 | 0.127297203471011 | -4.3806195586282 | 0.0000118342336938602 | 0.000245204735557667 |
| CLCA2 | 13.1551078478517 | -0.659255807046312 | 0.250301525500259 | -2.6338465406022 | 0.00844236351942327 | 0.039103811747445 |
| CYP1B1 | 186.415557617418 | -0.956803144776497 | 0.180613347181518 | -5.29752180393901 | 1.17384892196117E-07 | 6.02083675586278E-06 |
| DUSP5 | 836.096066524541 | -0.691409168592391 | 0.112031237774542 | -6.17157484222234 | 6.76131121885117E-10 | 8.63022022064202E-08 |
| PLA2G12B | 151.91395249101 | 0.767185623653405 | 0.246422160224949 | 3.11329802057198 | 0.00185009095451057 | 0.0124488413884794 |
| OIT3 | 53.8495157491126 | 0.533649803392296 | 0.166026616424225 | 3.21424248042696 | 0.00130789173667237 | 0.00953739979454804 |
| CILP | 158.191772916407 | -0.750455643097899 | 0.210352806960205 | -3.5676046064832 | 0.000360259574762166 | 0.00358992869463539 |
| NDST4 | 0.933541345730708 | -1.04949921029498 | 0.363966432925318 | -2.88350549763562 | 0.00393275782232794 | 0.0220189665317748 |
| FGF5 | 6.15330709102171 | -0.685465896167706 | 0.217759508463492 | -3.14781155139605 | 0.00164497704980342 | 0.0114151707404066 |
| GPAT3 | 239.656533427274 | -0.604406210351264 | 0.101073969631374 | -5.97984043325486 | 2.23356304516034E-09 | 2.33421297988288E-07 |
| FRAS1 | 288.717249067372 | -0.519915968035311 | 0.159870522701634 | -3.25210651250342 | 0.00114553085618704 | 0.00867500971299979 |
| SLC38A4 | 123.326155735265 | 0.632130797290102 | 0.188256433099745 | 3.35781777483894 | 0.00078560384958436 | 0.00655656699221367 |
| COL2A1 | 23.2637959256581 | 1.20581745248594 | 0.19615040469847 | 6.14741251408359 | 7.87571132010031E-10 | 9.82759470025353E-08 |
| PHLDA1 | 5388.14541317816 | -0.540149789257134 | 0.104293518669419 | -5.17913093880027 | 2.22921964401352E-07 | 9.99323905296249E-06 |
| RHOF | 191.906806665255 | -0.666773177231253 | 0.120004504764984 | -5.55623456416956 | 2.75656384857472E-08 | 1.88517399231157E-06 |
| SLAIN1 | 83.7476871047135 | -0.930071476557072 | 0.172300387470737 | -5.39796508998002 | 6.74009777242828E-08 | 3.84259939477864E-06 |
| ZIC5 | 99.7082829205199 | -1.25953833524456 | 0.288271062268828 | -4.3692846771764 | 0.0000124654181361207 | 0.000255747554176779 |
| SERPINA10 | 77.0093127914096 | 0.94463154338532 | 0.246177503551371 | 3.83719685900624 | 0.000124446709884164 | 0.00156397853135896 |
| WARS1 | 7060.58884404864 | -0.654517388254 | 0.103555450978581 | -6.32045326507607 | 2.60797197385062E-10 | 3.94612406427635E-08 |
| GCNT3 | 3388.83653062378 | -0.712248092315173 | 0.148036335863521 | -4.8113058740647 | 0.0000014994733160772 | 0.0000468648473236015 |
| BCL2A1 | 161.630041532041 | -0.631319847030584 | 0.139423217033606 | -4.52808262829292 | 5.95213080864674E-06 | 0.000141171034399124 |
| NKD1 | 3227.23349442606 | 0.96625153462728 | 0.17624109923663 | 5.4825550839872 | 4.19226404596744E-08 | 2.63035073593327E-06 |
| C17orf64 | 1.01081556517595 | -0.82004377553444 | 0.239001058935233 | -3.43113030204884 | 0.00060107180633629 | 0.0053149242061074 |
| ADCYAP1 | 30.2709682195478 | -0.517206192715178 | 0.189403947642494 | -2.73070439741532 | 0.00631991296229733 | 0.0315354415525436 |
| OSBPL1A | 603.337256437926 | -0.502658293614485 | 0.0905726151333381 | -5.54978226999947 | 2.86025592035356E-08 | 0.0000019520954793564 |
| SLC14A1 | 168.008557194234 | 1.18130053273011 | 0.229167978907943 | 5.15473644424221 | 2.53987898953945E-07 | 0.0000111761359431813 |
| ASGR1 | 164.965787538827 | 0.734372070930298 | 0.143015356831294 | 5.13491758648402 | 2.82267688032545E-07 | 0.0000122052146127885 |
| ANAPC11 | 3491.6948791015 | 0.552946436042567 | 0.0988746112585985 | 5.59240060723355 | 2.23951359513521E-08 | 1.60571766397651E-06 |
| RNF165 | 10.4480182414506 | -0.501445183584867 | 0.180775600582201 | -2.77385433637021 | 0.0055396476074865 | 0.0286420679173722 |
| CBLN2 | 10.5030836685532 | 0.555910352203855 | 0.154073960117105 | 3.60807466609757 | 0.000308477722351171 | 0.00318103977516739 |
| PMAIP1 | 492.188597484143 | -0.76503244638864 | 0.0983080795862037 | -7.78198953340151 | 7.13927761516878E-15 | 0.0000000000058020515 |
| STAC2 | 5.34512445882734 | -0.582352200524377 | 0.188962495546641 | -3.08184012303455 | 0.00205725299029203 | 0.0134714420406004 |
| CACNA1A | 24.9956143385228 | -0.529555874042738 | 0.124823049913586 | -4.24245261119118 | 0.0000221090236195808 | 0.000403653307332269 |
| PGGHG | 2935.54083455552 | 0.549730678728062 | 0.140557770512078 | 3.911065725682 | 0.0000918897599996205 | 0.0012341274513684 |
| HUNK | 1185.52443845427 | 0.548876081157325 | 0.139745198278507 | 3.92769188436397 | 0.0000857649881693329 | 0.00116829031949443 |
| KLK3 | 32.6585597104058 | -0.640752053794616 | 0.212725705860846 | -3.01210449015392 | 0.00259443276013899 | 0.0160539068510981 |
| IGLON5 | 13.1875858051509 | -0.627178187024653 | 0.153595871406168 | -4.08330107627793 | 0.0000444004376299154 | 0.000700065740320429 |
| CFAP74 | 16.2566418697883 | 0.54304613459375 | 0.161969840805073 | 3.35276080963304 | 0.000800098190345961 | 0.00664107314012152 |
| PADI3 | 80.522798156652 | -1.41645801485881 | 0.253478591341553 | -5.58807750730392 | 2.29597172564226E-08 | 1.63434222206681E-06 |
| FHAD1 | 46.6373992536354 | -0.68775250017774 | 0.108411309109855 | -6.34391841427567 | 2.23993481538546E-10 | 3.55013744531378E-08 |
| TMEM61 | 46.0066852696476 | -0.502674057912567 | 0.156794302879501 | -3.20594593477595 | 0.00134619279172832 | 0.00974656404870717 |
| C1orf162 | 151.969029020868 | -0.502406480741585 | 0.0976145584610823 | -5.14683965857294 | 2.64911575181787E-07 | 0.0000116262111512196 |
| PROK1 | 1.00506895637833 | -1.20055405608426 | 0.377242498665421 | -3.18244646436043 | 0.00146036510974667 | 0.0104044820002117 |
| ITGA10 | 34.0045605193071 | -0.837265375804847 | 0.122640036955573 | -6.82701503187049 | 8.66996143444645E-12 | 2.37656434664556E-09 |
| MAEL | 9.15614066141492 | 0.734515334157163 | 0.212989611236685 | 3.44859699913218 | 0.00056350703416946 | 0.00506172501657134 |
| FCGR2A | 750.669361695161 | -0.527738428932297 | 0.110650326996591 | -4.76942493761048 | 1.84752568307381E-06 | 0.000055812966479995 |
| LHX9 | 1.49901780255802 | -1.21867154869456 | 0.344934903833068 | -3.53304793209428 | 0.000410797984975476 | 0.00396590826026266 |
| SELENBP1 | 9940.55315148334 | 0.824621238699712 | 0.134797380746272 | 6.11748710645861 | 9.50624494881906E-10 | 1.16024760430076E-07 |
| HORMAD1 | 9.10365315073117 | -0.608227151306011 | 0.231587554273701 | -2.6263378151452 | 0.00863091123991394 | 0.0397404562419389 |
| S100A8 | 186.470271943716 | -0.708147903863425 | 0.158439461669159 | -4.46951723013375 | 7.83963440123152E-06 | 0.000175483971650592 |
| PKLR | 109.615676697061 | 1.0926862405565 | 0.227009300410002 | 4.81339856377246 | 1.48384934270464E-06 | 0.0000466218466642063 |
| ETNK2 | 99.8507363971168 | 0.675773774949864 | 0.123102512185671 | 5.48952058696103 | 4.03026143293202E-08 | 2.53822980866502E-06 |
| REG3G | 5.70519986635429 | -1.20938034006059 | 0.363545640630177 | -3.32662588929475 | 0.000879043031605771 | 0.00711102009263672 |
| NAT8 | 10.155111018187 | -0.912388454280168 | 0.283772079579735 | -3.21521573098879 | 0.00130346523771739 | 0.00951549541142652 |
| LYG1 | 26.2765393747309 | -0.743109967195814 | 0.120040079677434 | -6.19051544444708 | 5.99677913352419E-10 | 7.80327968028467E-08 |
| GPR17 | 15.7646102977053 | 0.751122167447796 | 0.16293969721287 | 4.6098168849946 | 4.03023794509421E-06 | 0.000102963496837159 |
| DLX1 | 10.6825702068353 | -0.845606267607616 | 0.242417865044882 | -3.48821761734045 | 0.000486252143701864 | 0.00450949644749799 |
| CPO | 3.44902034651805 | 0.626352825957922 | 0.170164969687838 | 3.68085644834507 | 0.000232451853826155 | 0.00253875078238219 |
| ABCA12 | 77.0634986817016 | -1.08644418445965 | 0.247289836365559 | -4.39340411408416 | 0.0000111589419095647 | 0.000233820385551168 |
| ZNF660 | 27.5100039330446 | 0.540605753555085 | 0.126841732190519 | 4.2620495969188 | 0.0000202560470900061 | 0.000376544039346295 |
| MYH15 | 38.8907401953726 | -0.570451858979282 | 0.112854952864422 | -5.0547348122558 | 4.30989201996667E-07 | 0.000017077181153048 |
| PHLDB2 | 340.973155556417 | -0.51373509457975 | 0.124027844468905 | -4.14209483990951 | 0.0000344147968871563 | 0.000568064974087009 |
| TAGLN3 | 6.10143408539592 | -0.774366849108046 | 0.216443494430192 | -3.57768595053708 | 0.000346649596881154 | 0.00348279825929049 |
| IGSF11 | 3.60742726304763 | -0.541444492269979 | 0.200084188328447 | -2.70608335817708 | 0.00680819539821224 | 0.0333743287169472 |
| AMT | 480.118589718407 | 0.703393284873017 | 0.123022539860347 | 5.71759683771361 | 1.08041106343813E-08 | 8.5014368902348E-07 |
| STXBP5L | 5.10172780388926 | 0.586713859958107 | 0.202656969119822 | 2.89510823391032 | 0.00379027819587908 | 0.0214365776131555 |
| VWA5B2 | 28.9306020010265 | -0.70034262103991 | 0.176879853574386 | -3.95942560380618 | 0.0000751302435291316 | 0.00104641964418979 |
| GC | 1.05652708704137 | -1.34618211870943 | 0.456871165208098 | -2.94652458116123 | 0.00321366875328203 | 0.0189310393600947 |
| DDIT4L | 33.9578032330586 | 1.24771294008549 | 0.199242672411104 | 6.2622776787045 | 0.0000000003793945645 | 5.36884851538711E-08 |
| SFRP2 | 2011.30667723701 | -0.910904503803579 | 0.214207872881809 | -4.25243242252901 | 0.0000211461038071194 | 0.000389839031707655 |
| GLRA3 | 1.38996382608378 | -0.769969285958327 | 0.241540511311837 | -3.187743876903 | 0.00143387483968842 | 0.0102504579711116 |
| NKD2 | 857.715215360799 | 0.674415877115679 | 0.139834934719375 | 4.8229426964665 | 1.41455619685155E-06 | 0.0000448394202228526 |
| ADAMTS16 | 53.6574690805681 | -0.564357921331754 | 0.173940228854692 | -3.24455087272084 | 0.00117636002477972 | 0.00883445586092147 |
| PLK2 | 922.776694176258 | -0.643355452154034 | 0.105565785546177 | -6.09435575007031 | 1.09879061633507E-09 | 1.31897218206563E-07 |
| SHISAL2B | 2.3551465069585 | 0.753620813675499 | 0.232242860454903 | 3.24496870301783 | 0.00117463532837417 | 0.00882745048348071 |
| GZMA | 196.327468988174 | -0.728813528562026 | 0.140355636521627 | -5.19262030812509 | 2.07354790808338E-07 | 9.39615029026076E-06 |
| CXCL14 | 5476.80137926665 | 0.864026152148804 | 0.157288392601282 | 5.49326074136357 | 3.94579556133715E-08 | 2.50865580156344E-06 |
| GABRB2 | 47.1730622990942 | 1.01854430015165 | 0.235987962433775 | 4.31608582762982 | 0.0000158820248024166 | 0.000309875538764536 |
| TENM2 | 14.8035530640348 | 0.821520710437466 | 0.181311638953653 | 4.53098717312607 | 5.87087034864179E-06 | 0.000139838779344216 |
| KLHL3 | 209.033684268278 | 0.582255109135593 | 0.120526111929877 | 4.83094575783172 | 0.0000013588602393455 | 0.0000434030602905369 |
| TRIM7 | 275.447910728229 | -1.06656043901608 | 0.187737256972807 | -5.68113360242912 | 1.33804901549779E-08 | 1.02395961501778E-06 |
| PRSS35 | 12.678099935183 | -0.674407154764412 | 0.148907314997935 | -4.52903979078371 | 5.92523408381759E-06 | 0.000140832749275784 |
| RSPO3 | 99.1421641430988 | -0.587770471999282 | 0.162470379923819 | -3.61770848492434 | 0.000297222891223569 | 0.00309314177820612 |
| SLC2A12 | 244.442708212889 | 0.588078637624496 | 0.146373631171607 | 4.01765422444865 | 0.0000587803641542975 | 0.000862163569319305 |
| IGFBP1 | 27.915659923549 | -0.914813370826806 | 0.274213557686015 | -3.33613472122448 | 0.000849519853777361 | 0.00694952126957498 |
| SYTL5 | 245.079093223389 | -0.858793899044189 | 0.159976084533788 | -5.36826427241884 | 7.94980085387637E-08 | 4.33439903879986E-06 |
| CCNB3 | 13.0892916312886 | -1.02333552019109 | 0.132205232747901 | -7.74050692942281 | 9.90212056484939E-15 | 7.70108641696961E-12 |
| GPR174 | 11.0062865898931 | -0.560252790433988 | 0.166042647361893 | -3.3741499508431 | 0.000740440215953138 | 0.00626088538606949 |
| NXF3 | 102.901687084616 | -0.955391038709326 | 0.283307537702056 | -3.3722753953482 | 0.000745498640795593 | 0.00629093251210856 |
| HTR2C | 2.61911343118835 | -2.5302338024433 | 0.605091184688393 | -4.18157439154613 | 0.0000289497499065049 | 0.000500329476161932 |
| ST18 | 24.0608937164516 | -0.67893244731646 | 0.187496420576475 | -3.62104217898678 | 0.000293418668663772 | 0.0030644931659756 |
| GSDMC | 12.3991945503371 | -0.598671933204253 | 0.12537538043775 | -4.77503582532694 | 1.79675265319077E-06 | 0.0000545254103702413 |
| CDKN2B | 466.223150185587 | -0.550013175832229 | 0.104519642096885 | -5.26229486436999 | 1.42268357987971E-07 | 6.99667415857901E-06 |
| CDKN2A | 345.332399813951 | -0.812661990171542 | 0.166221943637583 | -4.88901749304176 | 0.0000010134049115629 | 0.0000344413486305758 |
| NTRK2 | 341.032344139429 | 1.04111175404825 | 0.214181113425218 | 4.86089430295059 | 0.0000011685663768208 | 0.0000383882090114355 |
| SHC3 | 137.019997925176 | 0.671480531831147 | 0.135635442883594 | 4.95062734013726 | 7.39746351947109E-07 | 0.0000267733739197134 |
| CRB2 | 207.38957110267 | 0.52866107288101 | 0.127317509306355 | 4.15230454759313 | 0.0000329143835414395 | 0.00054908450120982 |
| GBGT1 | 172.10796005121 | 0.557205052396877 | 0.103175793967663 | 5.40054048502415 | 6.64404144367384E-08 | 3.79812023862121E-06 |
| CACNA1B | 15.0370944297804 | 1.00560154723049 | 0.230611811328196 | 4.36058127915819 | 0.0000129717385797795 | 0.000264351542708706 |
| TMEM236 | 254.774012551441 | 0.546487909916249 | 0.154285488725323 | 3.54205644634001 | 0.000397020506398703 | 0.00386525757641497 |
| CDHR1 | 2914.02096499203 | 0.581498621413532 | 0.17469867717796 | 3.3285805640141 | 0.000872897546343466 | 0.00707842864811304 |
| RGR | 13.343287560592 | 0.872975070922385 | 0.312734948046337 | 2.79142154203066 | 0.00524770783312667 | 0.0275068723127621 |
| LRMDA | 132.711874471292 | 0.709869781029817 | 0.120867068660417 | 5.87314467784632 | 4.27604976476031E-09 | 3.94518074089238E-07 |
| ANKRD1 | 5.24158195298828 | -1.18896258685676 | 0.208181571185718 | -5.71118077399893 | 1.12194989849767E-08 | 8.76641320223345E-07 |
| HABP2 | 59.798866291275 | -0.737662006221884 | 0.281754181063437 | -2.61810491485058 | 0.00884196152764768 | 0.0405003256276666 |
| NPFFR1 | 124.479960730655 | 0.792211422131043 | 0.136386767881981 | 5.80856511547045 | 0.0000000063010523156 | 5.43092246233065E-07 |
| INA | 11.2062074279184 | -0.524511714383425 | 0.187134880062411 | -2.80285382505119 | 0.00506526211860904 | 0.026751815503873 |
| ADAM12 | 693.750533941497 | -0.568497508821938 | 0.152680108179265 | -3.72345497787082 | 0.000196514918048276 | 0.00223228664720464 |
| LRRC4C | 10.6273116715399 | -0.582512632588915 | 0.196860111698542 | -2.9590180944372 | 0.00308620979572217 | 0.0183547977927335 |
| DRD2 | 151.134271770417 | 0.98440368540767 | 0.239530413014765 | 4.10972315798157 | 0.000039613373760174 | 0.000634155311291401 |
| GRIK4 | 9.93381923605627 | 0.776227471913216 | 0.158924599349009 | 4.88424998453869 | 1.03823286234948E-06 | 0.0000350359065415654 |
| PLCH2 | 207.974867936732 | -0.5826943775139 | 0.152192085606556 | -3.82867726131482 | 0.000128833822094555 | 0.00160344647505996 |
| DUSP15 | 99.8849526803887 | 0.932262702347429 | 0.156171710797228 | 5.96947230448073 | 2.38022159346009E-09 | 2.47203014063641E-07 |
| COMMD7 | 2283.88846501144 | 0.50059097173863 | 0.0664195058343879 | 7.53680662705946 | 4.81619685036609E-14 | 0.0000000000309737029 |
| SPATA25 | 39.431851125751 | 0.697889462783091 | 0.118250906651152 | 5.90176838848186 | 3.59625798829078E-09 | 3.38777632801184E-07 |
| CDH22 | 3.58057944182414 | 0.800812352428744 | 0.273309221715529 | 2.93005975942612 | 0.00338896822841844 | 0.0196760200511753 |
| CNDP1 | 13.2002043002106 | -0.779091060999911 | 0.236943468197331 | -3.28808836524276 | 0.00100870173586196 | 0.00786133848769415 |
| FREM2 | 186.293991940823 | 0.62687887006197 | 0.22345608309578 | 2.80537840535436 | 0.00502575396830295 | 0.0265943123245384 |
| SLC7A11 | 826.142061699422 | -0.862377073159235 | 0.112990233212954 | -7.63231518899429 | 2.30574863088965E-14 | 1.57364991253493E-11 |
| GPR158 | 36.1057577812744 | -0.99488130398075 | 0.174760172661544 | -5.69283772629092 | 1.249451257836E-08 | 9.69469813562677E-07 |
| PTPRO | 2005.783642415 | 0.547445766057916 | 0.165660738309284 | 3.30461986132074 | 0.000951052991780518 | 0.00752961982744415 |
| VENTX | 145.107760828453 | 1.84379509843345 | 0.210111629545421 | 8.77531197308082 | 0.0000000000000000017 | 0.0000000000000040711 |
| ITIH2 | 11.6666568269835 | -0.719531169091774 | 0.202775630010451 | -3.54841047247487 | 0.000387563726861954 | 0.00379417627450745 |
| SERP2 | 99.0858213251567 | 0.625029528558806 | 0.149967359746054 | 4.16777043762852 | 0.0000307593475678244 | 0.000524555890547264 |
| TDO2 | 179.38617329637 | -0.601070719432546 | 0.144599716858841 | -4.15679043147308 | 0.0000322749798765888 | 0.000539939908470678 |
| TMEM132D | 5.50847031248569 | 0.914967011449042 | 0.198747271559655 | 4.60367080397584 | 4.15108445834133E-06 | 0.00010532737463765 |
| AP1S3 | 303.850387736773 | -0.535615813003506 | 0.0733829851040274 | -7.29891012534064 | 2.90108013161097E-13 | 1.42673414354903E-10 |
| ASTN1 | 4.22070038479599 | -0.742932184846498 | 0.258898023390806 | -2.86959388533084 | 0.00410999298105828 | 0.0227502922347349 |
| ARL11 | 155.875973762042 | 0.702912012588955 | 0.141792992276883 | 4.95731136850796 | 7.14753803916449E-07 | 0.0000260095720463263 |
| GUCY1A2 | 86.1454338415129 | -0.838605537100968 | 0.138547506256336 | -6.05283746897188 | 1.42316440887223E-09 | 1.62435031267935E-07 |
| ZNF256 | 60.1456370577723 | 0.731136473379144 | 0.13998460933111 | 5.22297756069572 | 1.7606873326831E-07 | 8.26979013758262E-06 |
| PRDM8 | 122.010913436874 | -1.36461502763246 | 0.145658873713676 | -9.36856775588493 | 7.35234048955413E-21 | 2.7319663405741E-17 |
| PART1 | 56.8311443154237 | 0.772583707313194 | 0.175731735224587 | 4.39638125877391 | 0.0000110070529400839 | 0.000231362579775164 |
| JAKMIP1 | 23.5115509560229 | -0.53033095872121 | 0.138085745452092 | -3.84059163373386 | 0.000122738145613663 | 0.00154832480785066 |
| TEKT5 | 14.2201252257543 | 1.06047398648718 | 0.173294261555611 | 6.11949857408792 | 9.38702703572014E-10 | 1.14989361951851E-07 |
| ACOXL | 16.9532333974391 | -0.533031323063998 | 0.146604872445098 | -3.63583634141229 | 0.000277080209183557 | 0.00293231530237865 |
| CLGN | 38.1054263867746 | 0.542631696333515 | 0.204641882520929 | 2.65161603113194 | 0.0080107583117929 | 0.0376946361985336 |
| PTPRR | 351.171522036607 | -0.646425658623575 | 0.136582053275257 | -4.73287407182858 | 2.21362834463783E-06 | 0.0000640936442436177 |
| FEZF2 | 2.84536032418995 | 1.55313322051985 | 0.347154842072651 | 4.47389185542403 | 7.68085997978398E-06 | 0.000172993891697165 |
| ADGRF1 | 91.5705077773817 | -0.541518726394351 | 0.189728372023961 | -2.85417895393084 | 0.004314821838154 | 0.0236434985927488 |
| FRMD1 | 306.387333150835 | 0.621790129885511 | 0.155905975534764 | 3.98823796042932 | 0.000066565875808782 | 0.000952952062841304 |
| CD8A | 242.293095097602 | -0.611118604103787 | 0.138624702706453 | -4.408439420771 | 0.0000104118133863007 | 0.000221637086737536 |
| CLCA3P | 3.14099731881453 | -0.568975243357698 | 0.190783148004469 | -2.9823139481081 | 0.00286078469882669 | 0.0173221730758939 |
| SEMA3D | 34.5000151172212 | -0.735853867879229 | 0.207522202262837 | -3.54590429291625 | 0.000391268283975433 | 0.00382260997741935 |
| C16orf74 | 61.4777770877858 | -0.718909784684892 | 0.153287043711547 | -4.6899579199774 | 0.0000027326123988162 | 0.0000755239866456293 |
| UBASH3B | 238.650228575813 | -0.81303698226102 | 0.119256523717764 | -6.81754722437808 | 9.26079674960041E-12 | 2.51788264146453E-09 |
| CERS3 | 1.1316033505733 | -0.732388572204901 | 0.235544430545377 | -3.10934361941455 | 0.0018750351247027 | 0.0125841112069625 |
| ENPP3 | 600.22580152237 | 0.943233634488269 | 0.194696229864015 | 4.84464252413653 | 1.26839840706876E-06 | 0.0000411823102225179 |
| UCHL1 | 148.758659823088 | -0.730079749573295 | 0.15429489730124 | -4.73171674723574 | 2.22628928172647E-06 | 0.0000643488039407923 |
| TDH | 1.05289232351066 | -0.583733419580872 | 0.21922148296058 | -2.6627564584345 | 0.0077503485263021 | 0.0368111284500206 |
| FAM167A | 138.391063262205 | -0.707711619519713 | 0.137613270293415 | -5.14275707575839 | 2.70735600187179E-07 | 0.0000118352156097512 |
| GBP5 | 379.097592544693 | -0.670851575189152 | 0.148513217699384 | -4.51711696494967 | 6.26872825698621E-06 | 0.000146600566692401 |
| WNT7A | 6.10111005497311 | 1.55660362425912 | 0.296697032954928 | 5.24644149203738 | 1.55065061477777E-07 | 7.49371522736537E-06 |
| APCDD1 | 3427.55217987308 | 1.14187311082188 | 0.170296478557551 | 6.70520682807889 | 2.01121295630788E-11 | 4.7365481468203E-09 |
| MAGEC1 | 2.4102584559839 | -3.00279005851134 | 0.558049376413737 | -5.38086804757055 | 7.41275169201561E-08 | 4.11789438678382E-06 |
| PIK3AP1 | 893.677148514599 | -0.607934377151648 | 0.101981761750042 | -5.96120685423831 | 2.50381769313027E-09 | 2.55722264845166E-07 |
| SLC26A2 | 3059.21890576759 | 0.518037058393825 | 0.162650472540848 | 3.18497112428448 | 0.00144768452733774 | 0.0103337173880957 |
| RAET1L | 50.9313990464369 | -0.822109585205207 | 0.155860175052036 | -5.27466098976685 | 1.33001718837191E-07 | 6.65844832537926E-06 |
| GRIP1 | 73.6667868666909 | 0.576206442249052 | 0.129254575630788 | 4.45791910605137 | 8.27591198416536E-06 | 0.000182923363234936 |
| WIF1 | 147.386376325654 | 2.56368605515708 | 0.331714886976914 | 7.72858305673663 | 1.08750220849466E-14 | 8.08183307921743E-12 |
| CFAP161 | 4.71119214541705 | -0.730575882315948 | 0.155055423942697 | -4.7117080056867 | 2.45649187581917E-06 | 0.0000697960928726801 |
| SLC28A1 | 3.62929614069182 | 0.741843111271876 | 0.198575526300646 | 3.73582346773548 | 0.000187101897003687 | 0.00214503312978995 |
| CXCL13 | 203.395551712164 | -0.964516669691653 | 0.185997735158379 | -5.18563663622224 | 2.15278184266875E-07 | 9.72882843007141E-06 |
| TIAM1 | 122.569550496469 | -0.702502636064691 | 0.124901587881231 | -5.62444920021917 | 1.86100807632262E-08 | 1.36482087913117E-06 |
| GDF6 | 10.1857024033458 | -0.6319617337134 | 0.195148939279109 | -3.23835597594279 | 0.00120220703227451 | 0.00897415347618846 |
| CD109 | 530.572950037357 | -1.16751031072181 | 0.166905096849667 | -6.99505487105284 | 2.65155133285163E-12 | 9.14156491476537E-10 |
| LRFN2 | 15.9823942442841 | 0.925222280015899 | 0.260651442238015 | 3.54965340713914 | 0.000385738642765762 | 0.00378184452986591 |
| NODAL | 14.6418890175988 | 0.901704628587146 | 0.190509624655326 | 4.73311849844084 | 2.21096321641108E-06 | 0.0000640719513719404 |
| GLYATL2 | 5.35324297410234 | 0.652802130155934 | 0.177972382956042 | 3.66799679429573 | 0.00024445824833776 | 0.00265341536543699 |
| MS4A1 | 73.1063874803367 | -0.659085210698972 | 0.211496041660403 | -3.11630045425275 | 0.00183135577081936 | 0.0123488824535493 |
| MPV17L | 502.60186736589 | 0.886569634792666 | 0.180495995508153 | 4.91185210118758 | 9.0220096951793E-07 | 0.0000314941595225664 |
| NMNAT2 | 93.9408345601744 | 0.626382159765819 | 0.1910039787012 | 3.27941943421875 | 0.00104020906562175 | 0.0080283110021977 |
| SUSD3 | 262.449611158038 | 0.674327107603193 | 0.134995295357927 | 4.99518969024275 | 0.0000005877796443186 | 0.0000221357284519174 |
| KCNJ15 | 52.0617699835133 | -0.567356773597248 | 0.157148612949157 | -3.61031995733114 | 0.000305819522693339 | 0.0031584979857661 |
| DPYSL5 | 4.09727358402272 | -0.637020587604639 | 0.22532679155879 | -2.82709651700887 | 0.00469721680192127 | 0.0252330484648988 |
| MEGF11 | 26.0755094813135 | 0.744060494086905 | 0.165922730056924 | 4.48437952914369 | 7.31264059013412E-06 | 0.000165683825620098 |
| RADIL | 22.915866523052 | -0.59581003743698 | 0.157382610317466 | -3.78574250506543 | 0.000153250300407693 | 0.00183889362979336 |
| SLC30A2 | 126.931660797439 | 0.730542379242657 | 0.223743052948038 | 3.26509524929169 | 0.00109427272472214 | 0.00837981874517007 |
| LRRC43 | 24.4527365086483 | 0.57810985969228 | 0.135441056532532 | 4.2683501922729 | 0.0000196924009175762 | 0.000367906855578538 |
| CLSTN2 | 101.420430703161 | 0.624985246018391 | 0.173099438408482 | 3.61055617374993 | 0.000305541116588819 | 0.00315757293602079 |
| SLC13A3 | 306.75783130529 | 1.13723092986346 | 0.208485154512868 | 5.45473337188269 | 4.90463824939928E-08 | 0.0000029553317538092 |
| CPA2 | 23.514831583 | -1.08215250692409 | 0.246611984083246 | -4.38807753381036 | 0.0000114357010932165 | 0.000238127469464102 |
| GDPD5 | 1253.58253915701 | 0.544039698265689 | 0.117635590593347 | 4.62478825941693 | 3.74981262554128E-06 | 0.0000969847129337598 |
| HTR6 | 1.93079966533497 | -0.543730771051948 | 0.207592359473513 | -2.61922342629052 | 0.00881302045044676 | 0.0404065025916974 |
| PLA2G2F | 39.7467224433109 | -1.01459865260423 | 0.261134232254077 | -3.88535292307847 | 0.0001021813392035 | 0.00133900797243081 |
| CDA | 516.17200454795 | -0.574608547030444 | 0.13049634889462 | -4.40325382202424 | 0.0000106639277780834 | 0.00022642734778074 |
| STC1 | 458.628492665909 | -0.544274031098708 | 0.105447515875412 | -5.16156332920899 | 2.44896013636139E-07 | 0.0000108187747529984 |
| CLIC6 | 155.646922313659 | 0.548736989539782 | 0.150818422440643 | 3.63839496965795 | 0.000274342467467216 | 0.00290886518612512 |
| IGF2BP1 | 159.610133522288 | -1.38311455267937 | 0.284004853701479 | -4.87003843298108 | 1.11576544426495E-06 | 0.0000369806025640323 |
| M1AP | 10.0930531372049 | -0.88404323930245 | 0.187477102159125 | -4.71547313843213 | 2.41149708083406E-06 | 0.0000687513089320143 |
| CELF3 | 71.1687904859762 | 0.994259492880237 | 0.205172471110439 | 4.84596928378882 | 1.25995010214713E-06 | 0.0000409477660991297 |
| LRRC36 | 119.926003118157 | 0.789936487840198 | 0.146905934329075 | 5.37715846162287 | 7.56705711079794E-08 | 4.17586673101163E-06 |
| TCAF2P1 | 1.28210471758198 | -0.663877256818334 | 0.193133213387721 | -3.43740594987969 | 0.000587314644097862 | 0.00522126055184881 |
| TNFRSF13C | 64.8647358799094 | -0.631335552689874 | 0.128737070149727 | -4.90406960447059 | 9.3871201407415E-07 | 0.0000325309918908474 |
| CILP2 | 60.4899657035551 | -0.607182076795189 | 0.174531505352989 | -3.47892534111343 | 0.000503428819484071 | 0.00463058126453697 |
| TFF2 | 247.904359559594 | -2.19886100121155 | 0.225291824679534 | -9.76005678119618 | 1.67061659565642E-22 | 0.0000000000000000008 |
| TFF1 | 5047.64566486432 | -0.970976096056963 | 0.203384050887672 | -4.77410146871954 | 1.80511358606451E-06 | 0.0000547294728424019 |
| AIRE | 10.4196279295983 | 0.73225994296552 | 0.207706124512683 | 3.52546148883928 | 0.000422745638057538 | 0.00405171298561695 |
| ZNF66 | 30.6139065282074 | 0.616569418898623 | 0.126661794013839 | 4.86784056470302 | 1.12824292876352E-06 | 0.0000373571287363461 |
| LCN1 | 1.53931926084776 | -0.630243850744608 | 0.221651413300438 | -2.84340100232224 | 0.00446348732489513 | 0.0243067811625375 |
| SPTBN4 | 20.794289505115 | -0.678822792531941 | 0.152842430518939 | -4.44132424633112 | 8.94069544583318E-06 | 0.000194404900584885 |
| NLRP4 | 1.69286043680448 | -1.28880415969835 | 0.332138942958675 | -3.88031631647212 | 0.000104320704137869 | 0.00135852530676737 |
| FCRL3 | 33.0749314482305 | -0.674056722204852 | 0.170635381083609 | -3.95027524728047 | 0.0000780613702694375 | 0.00107962297127813 |
| PTGER1 | 21.6223943614465 | 0.549283316646997 | 0.161566470922965 | 3.39973580848279 | 0.000674509907555407 | 0.0058166478412759 |
| SCGB3A1 | 3.37943642365338 | 0.641830269085167 | 0.213654440148936 | 3.0040577141189 | 0.00266404783946431 | 0.0163800492457006 |
| PGAP3 | 1861.35659787358 | 0.605054471468626 | 0.09043487979844 | 6.69049898465241 | 2.2241090262516E-11 | 5.16518430943792E-09 |
| IKZF3 | 296.149217940477 | -0.549309959958444 | 0.120353562510645 | -4.56413543977858 | 5.01556927647723E-06 | 0.000122969697759495 |
| KASH5 | 2.60684160861015 | 1.02042138492105 | 0.294462820339902 | 3.46536579301645 | 0.000529510734363167 | 0.00481978714713474 |
| ZNF385A | 473.891725622738 | -0.556634266120637 | 0.107657371578647 | -5.17042407740743 | 2.33563351925641E-07 | 0.0000103885568562913 |
| CD300LG | 3.94170932553075 | 0.737947486654474 | 0.274645795148124 | 2.68690618859276 | 0.00721172041021805 | 0.0348417153941797 |
| AQP5 | 102.004924611418 | -1.5247428749003 | 0.275573674302655 | -5.53297726554868 | 3.14840292529116E-08 | 2.12275989168522E-06 |
| KRT84 | 1.50757629250385 | -1.02117189239515 | 0.338688806132708 | -3.01507423305577 | 0.00256916367139419 | 0.0159461713991768 |
| ELAVL4 | 9.64380681206909 | -0.528058318541595 | 0.157639086802834 | -3.34979305736566 | 0.000808719560239366 | 0.00669269971133998 |
| SLC1A7 | 243.533227345159 | 0.567632678621449 | 0.199890569949306 | 2.83971714506295 | 0.0045153551840103 | 0.0245169837118082 |
| BSND | 0.7576046205996 | -0.821615217898901 | 0.2777465594042 | -2.95814723920024 | 0.00309494253374044 | 0.0183824129200616 |
| PRKAA2 | 65.2441882671558 | 0.615323900231692 | 0.215170786288328 | 2.85970001246899 | 0.00424041922803056 | 0.0233275373949331 |
| GBP4 | 1426.64775067685 | -0.822592129512746 | 0.150899573292912 | -5.45125550432144 | 0.0000000500154498608 | 3.00830337094423E-06 |
| GFI1 | 133.635077934202 | -0.745029376118209 | 0.129118695767461 | -5.77011231169795 | 7.9218716841315E-09 | 6.57377749034058E-07 |
| CADM3 | 31.9205907981863 | -0.832388394668274 | 0.204493851467649 | -4.07048128192723 | 0.0000469161108755203 | 0.000726374342545902 |
| SLAMF9 | 18.8506454453591 | -0.927416660659872 | 0.17265274095008 | -5.37157218331113 | 7.80531118125081E-08 | 4.28612835013776E-06 |
| VANGL2 | 594.764890347069 | 0.7845291132119 | 0.143740577173117 | 5.45795160031279 | 4.81658901183296E-08 | 2.91277341290629E-06 |
| FCGR3B | 119.827375864108 | -0.675521175595363 | 0.194902777250781 | -3.46593919863014 | 0.000528382754240827 | 0.00481149411374784 |
| LMX1A | 3.45550351201063 | 1.28277098082778 | 0.285637950433183 | 4.49089828183685 | 7.09234181979849E-06 | 0.0001620353650859 |
| CAPN13 | 236.827578169664 | 0.566128053755997 | 0.150052766842707 | 3.77285981237148 | 0.000161386977796339 | 0.00190980301184189 |
| KCNJ3 | 109.948800745218 | -0.608642554583058 | 0.214227452387847 | -2.84110438601092 | 0.00449575939533531 | 0.0244427224351818 |
| INHBB | 308.52721862281 | 0.618591698237807 | 0.166663947062422 | 3.71161075410101 | 0.000205944519757456 | 0.00231191561924433 |
| SPRR3 | 17.7223889618141 | -2.33462416139606 | 0.338365666893688 | -6.89970759394328 | 5.21096874111991E-12 | 1.61356682074567E-09 |
| SPRR2D | 27.1450754944754 | -1.7193437817844 | 0.306131423691134 | -5.61635836352137 | 1.95023971200777E-08 | 1.42713165096201E-06 |
| PGLYRP4 | 6.7635389975112 | 0.699922471325753 | 0.217602258591811 | 3.21652208876517 | 0.00129754544692344 | 0.00948055819008379 |
| ALPP | 20.3962366536015 | -1.32130084770233 | 0.270327344816223 | -4.88778095534726 | 1.01978902397772E-06 | 0.0000345880167747089 |
| ANTXR2 | 2120.90015892669 | -0.509896934495107 | 0.0814293754032309 | -6.26183035262328 | 3.80484749161756E-10 | 5.36884851538711E-08 |
| LMOD3 | 13.1352113335064 | 0.589282565450341 | 0.155537599163961 | 3.78868240616948 | 0.000151448375327873 | 0.00182119258098336 |
| PROK2 | 24.8629947798453 | -0.953402354524653 | 0.220869565410262 | -4.31658545962964 | 0.0000158461347831286 | 0.000309355773156676 |
| CRYBA2 | 16.466688799944 | -1.16959244720024 | 0.216961498708417 | -5.39078340702328 | 7.01511858053907E-08 | 3.95614832327803E-06 |
| IHH | 2760.21923483344 | 0.602640594751268 | 0.117510091491757 | 5.12841567137697 | 2.92190728763538E-07 | 0.0000125395909455649 |
| SPTA1 | 2.44986580024536 | -0.846002017178739 | 0.226792637921749 | -3.73028871188773 | 0.000191260478406119 | 0.00218298051838137 |
| IFI16 | 1446.10080256892 | -0.575584812140502 | 0.107416223751798 | -5.35845323952629 | 8.39374543284676E-08 | 4.54949164935594E-06 |
| AIM2 | 95.3609888394691 | -1.46260275158268 | 0.181175998329902 | -8.07282843790069 | 6.86880910089998E-16 | 7.5717536910403E-13 |
| PTX3 | 21.2465851812641 | -0.526095562083623 | 0.129877359546488 | -4.0507103310436 | 0.0000510623875797538 | 0.00077338241188502 |
| DNASE1L3 | 95.6441226145231 | 0.590682287285772 | 0.18964298032755 | 3.11470683631711 | 0.00184127812518428 | 0.0124020187436883 |
| PCOLCE2 | 37.6373804988606 | -0.72072905628568 | 0.208588039366683 | -3.45527508899342 | 0.000549731626056257 | 0.00496733991855535 |
| CXCL5 | 850.601710468789 | -1.31365756915062 | 0.226344023967567 | -5.80380937885443 | 6.48249716262122E-09 | 0.0000005572947817799 |
| PPBP | 167.6748287625 | -0.742131377688579 | 0.264714917611905 | -2.80351173399532 | 0.00505493928329258 | 0.0267057313604851 |
| DNAJC5G | 1.34844632369725 | -0.857240087268246 | 0.237462989015262 | -3.60999451250548 | 0.000306203483042503 | 0.00316135903598694 |
| RTP3 | 0.718568388576462 | 1.09282992213396 | 0.280057716364439 | 3.902159655947 | 0.0000953382371854492 | 0.00127023957289075 |
| LRRC2 | 166.29082010951 | 0.645480964887487 | 0.132196231355097 | 4.88274860993306 | 1.04617211979737E-06 | 0.0000352682339014756 |
| CAMK2N2 | 18.1722980234502 | -1.5009085271589 | 0.178568924094021 | -8.40520563571651 | 4.27112551419266E-17 | 7.93527663586838E-14 |
| HSPA4L | 456.10076504934 | -0.793179756442562 | 0.130071318018434 | -6.09803735770671 | 1.07378655636112E-09 | 1.29637436887467E-07 |
| CAMKV | 39.6747404392859 | 0.810791931552867 | 0.232169703947067 | 3.49223829711098 | 0.000478990713210243 | 0.00445574615609929 |
| HTR4 | 22.1053882314256 | 0.753551940288348 | 0.207831038467864 | 3.62579115152171 | 0.000288078114384409 | 0.00302003395023304 |
| SPZ1 | 1.11414692728698 | 0.908769485548635 | 0.280878971433542 | 3.23544863793285 | 0.00121451733930242 | 0.00904585498016738 |
| SLC6A18 | 4.20188504892378 | 0.987850145782378 | 0.220334044709136 | 4.48342037693922 | 7.34560191954128E-06 | 0.000166205425841204 |
| ACSL6 | 824.745806271215 | 0.614250825410863 | 0.203245653231519 | 3.02220891637552 | 0.00250937294812245 | 0.0156853464290457 |
| GJB7 | 6.34383667155385 | -1.4252556898634 | 0.306998111305873 | -4.64255523853489 | 3.44126595162274E-06 | 0.0000904739119136537 |
| GRIK2 | 13.0403546444888 | -0.680584044585334 | 0.168653978796791 | -4.03538682834965 | 0.0000545124106853452 | 0.000814568381652956 |
| SAMD3 | 39.7885351233406 | -0.758619053232838 | 0.151396528431428 | -5.01080877542342 | 5.42017538499789E-07 | 0.000020739302657334 |
| FAM183BP | 0.961203200186257 | -0.63087563684252 | 0.241550900233334 | -2.61177100243926 | 0.00900745661998827 | 0.0410559308008243 |
| KIF6 | 10.1214262392298 | 0.646932992244192 | 0.179209540516629 | 3.60992495365593 | 0.000306285607218398 | 0.00316135903598694 |
| SLC29A4 | 167.300198229473 | -0.570528057558151 | 0.144436414715712 | -3.95002921306997 | 0.0000781416553040997 | 0.00108015044596652 |
| IQUB | 5.70016236005001 | -0.656132637535102 | 0.127459990047346 | -5.14775371700074 | 2.63624300012371E-07 | 0.0000115849196333952 |
| TNFRSF11B | 563.088882970862 | -0.58755399781297 | 0.148031180539861 | -3.96912323248517 | 0.000072137569995379 | 0.00101362378814515 |
| CSMD3 | 2.76662171887514 | -0.677032315404237 | 0.260939667323316 | -2.59459331097163 | 0.00947029031942557 | 0.0425572877198479 |
| GPER1 | 85.5649112243628 | 0.542346286888209 | 0.141527395751205 | 3.83209401974453 | 0.000127057161071262 | 0.00158822033553625 |
| OSR2 | 175.056261436643 | -0.524870970722688 | 0.127226874665891 | -4.12547248449705 | 0.0000369974415878262 | 0.000601784261468913 |
| FREM1 | 659.466448580533 | 0.788421311530349 | 0.186761031391161 | 4.22155149635603 | 0.000024262650854968 | 0.000433899235236278 |
| NIPSNAP3B | 21.3950973029148 | 0.510649973328302 | 0.0975294648412558 | 5.23585333067769 | 1.64224329609365E-07 | 7.82334762221706E-06 |
| LETM2 | 55.251139691249 | -0.822515164180086 | 0.101034171242234 | -8.14096017285149 | 3.92155385896956E-16 | 5.2457841660664E-13 |
| PRKACG | 1.2125327106797 | 0.728988052287442 | 0.268000846709987 | 2.7200960789363 | 0.00652629505970315 | 0.0322852586504612 |
| NKX6-3 | 15.413700895123 | -1.30469503391386 | 0.396667291103644 | -3.28914196651762 | 0.00100493321112983 | 0.00784110509720107 |
| TRPV6 | 58.4966238752227 | -2.07664671962289 | 0.274591313321292 | -7.56268177061035 | 3.94842775793679E-14 | 2.59178081268362E-11 |
| CLDN3 | 15120.6646755502 | 0.510868204302688 | 0.114786271738869 | 4.45060368773785 | 8.56292372909145E-06 | 0.0001880244880816 |
| HDX | 45.9948902967294 | 0.564278033104272 | 0.120275452866391 | 4.69154777351871 | 0.0000027114593894476 | 0.0000751256212940402 |
| AQP3 | 1079.79069169613 | -0.968726046637334 | 0.165827914801982 | -5.84175497710448 | 5.16537424175774E-09 | 4.58197467885577E-07 |
| SLITRK5 | 10.5248404875353 | -0.557397905116013 | 0.213831398121383 | -2.60671683397778 | 0.00914149236747286 | 0.0414543314563611 |
| DEUP1 | 4.0982049294386 | 0.660392659340326 | 0.21322495659073 | 3.09716399946514 | 0.00195381749339454 | 0.012946657272547 |
| LRRC18 | 2.64536614885314 | 0.626947634863185 | 0.155489725923142 | 4.03208399230881 | 0.0000552844202750801 | 0.000821333444175579 |
| PHYHIPL | 86.1270644121933 | 1.23062738441073 | 0.257347514966049 | 4.78196723435654 | 0.000001735879783026 | 0.0000530633379378022 |
| HEPACAM | 1.59509544097416 | -0.884651141559064 | 0.322351516132903 | -2.74436786329346 | 0.00606275566343799 | 0.0305715734162686 |
| RPL10L | 8.6586970958934 | 0.576937518996238 | 0.1737265780097 | 3.32095138007049 | 0.000897111639139793 | 0.00722224541071569 |
| EML5 | 7.49938886600332 | -0.711127784364223 | 0.181672475548699 | -3.91433970510078 | 0.0000906519357013837 | 0.00122142708852767 |
| OTX2 | 1.15173767119582 | -1.40609703966161 | 0.541585493692705 | -2.59626052772276 | 0.00942445752988273 | 0.04238471069316 |
| OXGR1 | 202.451338383644 | 0.851561731638559 | 0.168275320155424 | 5.06052658733336 | 4.18100150457475E-07 | 0.0000166453633709511 |
| ZNF503 | 887.91548930012 | 0.517454423999979 | 0.0987880094493258 | 5.23802865230736 | 1.62300869949555E-07 | 7.79837024835202E-06 |
| CLEC1B | 1.17628489201419 | -0.670894219112324 | 0.233876432848495 | -2.8685841105971 | 0.00412313553902762 | 0.0228089168197952 |
| SLC39A2 | 168.373974330114 | 0.739928531156723 | 0.278053161760782 | 2.66110454012139 | 0.00778847713380305 | 0.0369397606451059 |
| RNASE7 | 12.2277738723568 | 0.504836966343255 | 0.195505918924314 | 2.58220809436819 | 0.00981703711185085 | 0.0436687091107364 |
| LARGE2 | 1443.49653565879 | 0.62796988165705 | 0.121536813360575 | 5.16691086670169 | 0.0000002379946876742 | 0.0000105697454783541 |
| SMCO2 | 7.71740196773928 | -0.793645994787084 | 0.115079273952292 | -6.8965154847614 | 5.32935587148366E-12 | 1.63508549590969E-09 |
| PASD1 | 0.8598616973553 | -1.68233847174684 | 0.511020782966409 | -3.29211360442345 | 0.000994374352210543 | 0.00777890724371569 |
| AC126323.1 | 0.847858945229717 | -1.34215484921325 | 0.381926990674446 | -3.51416601074236 | 0.000441137026433638 | 0.00418036396656099 |
| AVPR1A | 44.7272001068015 | -0.536807420660962 | 0.138069862977281 | -3.8879405620131 | 0.000101098374622603 | 0.0013289826431325 |
| CKB | 19351.195294517 | 0.781720634784685 | 0.17122772500302 | 4.56538586126106 | 4.98576412401872E-06 | 0.000122508393707152 |
| ASPG | 47.3511480378821 | 0.519466945490371 | 0.169011165394067 | 3.07356584565985 | 0.00211517004268264 | 0.0137346628697124 |
| MYRFL | 28.5741250744454 | -1.34473783106871 | 0.204469991740618 | -6.57670017796345 | 0.0000000000481003297 | 9.92945201215652E-09 |
| SYNPO2L | 2.94266580856909 | -0.636375275114531 | 0.165714709373547 | -3.8401858080084 | 0.000122941224615878 | 0.00154901283221518 |
| SERPINB7 | 20.8863772583054 | -1.19527362700293 | 0.257193879221341 | -4.64736420097414 | 0.0000033620327847855 | 0.0000891487914149425 |
| WDR72 | 543.839037213986 | -0.555894391798416 | 0.186369156220221 | -2.98275961040221 | 0.0028566226503722 | 0.0173001040698564 |
| HDGFL3 | 424.547094201526 | 0.528068489194222 | 0.129474979514454 | 4.0785369588359 | 0.0000453199985528145 | 0.00071021152371285 |
| CCDC68 | 370.416660498788 | -0.688468620864349 | 0.101610310407621 | -6.77557836505448 | 1.2390955620039E-11 | 3.16319341866676E-09 |
| CLEC4D | 5.76964150560136 | -0.644964674293 | 0.20038966906656 | -3.21855251968489 | 0.00128839373985172 | 0.00943430336065712 |
| A2ML1 | 8.51507111676689 | -1.05117308463055 | 0.242995827321195 | -4.32588944517593 | 0.0000151917606545564 | 0.000298673051034495 |
| BEAN1 | 45.1961406698727 | -0.762209222926597 | 0.168987841371861 | -4.51043824655609 | 6.46938329156287E-06 | 0.000150138179067623 |
| CDH16 | 53.2287098503336 | -1.17264346631668 | 0.247572992149604 | -4.73655650454824 | 2.17380152780138E-06 | 0.0000632691650937631 |
| CHRFAM7A | 5.2360194592298 | -1.21288982825717 | 0.186499221213121 | -6.50345787166128 | 0.0000000000784944116 | 1.45833895153622E-08 |
| TMPRSS5 | 67.6707270005036 | 0.72119537474211 | 0.187590321361288 | 3.84452337150768 | 0.000120786973605395 | 0.00153063962535491 |
| ZNF606 | 167.366562125194 | 0.569576625348042 | 0.127682399670333 | 4.46088597033459 | 8.16215138517565E-06 | 0.000181127184222325 |
| HTR3A | 8.92318430395842 | -1.04595175216577 | 0.218229860100202 | -4.79289017408302 | 1.64395630127899E-06 | 0.0000504377858966714 |
| ANPEP | 3270.53567387313 | -0.656280182484173 | 0.189874674243564 | -3.45638608781665 | 0.000547470548684853 | 0.00495628318600944 |
| ELFN2 | 43.4891226342048 | -1.38546050741657 | 0.20153521200369 | -6.87453320758264 | 0.000000000006219317 | 1.79306680547501E-09 |
| MS4A15 | 23.3363247473313 | 0.715556021260176 | 0.231377499757834 | 3.0925912070495 | 0.00198417250894554 | 0.013085130554951 |
| NKX3-1 | 43.9853957626335 | -0.703170305228609 | 0.149138489172519 | -4.71488151133946 | 2.41851447648159E-06 | 0.0000688926415012755 |
| PIP5KL1 | 20.4442682750802 | -0.594137950417243 | 0.128356227194209 | -4.62882061435385 | 3.67754155347057E-06 | 0.000095484739620468 |
| IGF2 | 25440.0403466833 | 1.8089506106049 | 0.284545511517108 | 6.357333141402 | 2.0528646580651E-10 | 3.31651690314072E-08 |
| ENGASE | 2858.12073984568 | 0.565772954162612 | 0.0995148937857488 | 5.68530933048772 | 1.30576094780121E-08 | 1.00615800959374E-06 |
| RBFOX3 | 22.2314073733753 | 0.909473485207762 | 0.187026776173542 | 4.86279827848747 | 1.15737781449753E-06 | 0.0000381330333718487 |
| OR51B5 | 2.3526418389796 | -1.78196597879941 | 0.405853013770934 | -4.39066834133494 | 0.0000113002789976909 | 0.000236042429881811 |
| JSRP1 | 167.187879698492 | -0.65887171912694 | 0.148161475052502 | -4.44698406852028 | 8.70842936352656E-06 | 0.000190593779303047 |
| NIBAN3 | 16.3709659649112 | -0.681966788159379 | 0.177964688462806 | -3.83203428753174 | 0.000127088021586478 | 0.00158822033553625 |
| ZNF610 | 35.7929521284864 | 0.615477447103889 | 0.140925568277517 | 4.36739375704954 | 0.0000125737925136709 | 0.000257654883114081 |
| AQP2 | 2.89182672163538 | -1.0845035906322 | 0.254843289458348 | -4.25557052311339 | 0.0000208516625070959 | 0.00038589999865097 |
| ANKRD33 | 3.26561522618773 | -0.671284277368597 | 0.223531996134363 | -3.00307915187719 | 0.00267262910752911 | 0.0164207353690958 |
| LAIR2 | 24.8292113430186 | -0.823941391620909 | 0.170212124363313 | -4.8406739220423 | 1.29399549318213E-06 | 0.0000417698693055011 |
| KIR3DL1 | 1.87532674003899 | -1.16609186646899 | 0.305542135062427 | -3.81646827934463 | 0.000135375469834666 | 0.00166942921556041 |
| DNAAF3 | 37.4505777162017 | -0.911511490416954 | 0.138176404546697 | -6.59672317721155 | 0.0000000000420344558 | 8.7311569620625E-09 |
| SERPINF2 | 231.675613672379 | 0.529243139612926 | 0.127933575853206 | 4.13685880413591 | 0.0000352092612557277 | 0.000579177626617828 |
| KLK1 | 1487.31739402808 | -0.568110498306523 | 0.137004261918785 | -4.14666296033401 | 0.0000337356096317107 | 0.000558784674246494 |
| KLK6 | 940.802534958836 | -1.13717659250012 | 0.217170344926214 | -5.2363346058436 | 1.63796887605772E-07 | 7.82334762221706E-06 |
| KLK11 | 434.726683122206 | -0.607680743132551 | 0.206612070590432 | -2.94116767426023 | 0.00326977486886109 | 0.0191636542524452 |
| ANGPTL4 | 309.842719392462 | -0.782459860090056 | 0.126610239774587 | -6.18006775347021 | 0.0000000006407409696 | 8.24140750205417E-08 |
| TBX10 | 116.421667955243 | 0.811449775365566 | 0.186240202168567 | 4.35700652124033 | 0.0000131853340959897 | 0.000268214077152121 |
| TEKT1 | 1.21375020771691 | -0.999354363638325 | 0.268152473723476 | -3.7268138897308 | 0.000193915535917039 | 0.00220650675472529 |
| RAB26 | 173.682848178637 | -0.771152569673615 | 0.171299326247051 | -4.50178402080487 | 6.73854393851461E-06 | 0.000154879990647289 |
| CASKIN1 | 10.9570392253842 | -0.768719325398656 | 0.178816850643613 | -4.29891994312513 | 0.000017163244928065 | 0.000328359975334297 |
| ABCA3 | 428.791247081575 | -0.846615772800988 | 0.164124547255373 | -5.15837385058361 | 2.49103854283416E-07 | 0.0000109901465632533 |
| PLAAT5 | 23.5027712461026 | -1.43774419331016 | 0.216278540193794 | -6.64765071940047 | 2.97807974370517E-11 | 6.55216728874922E-09 |
| ENTPD3 | 30.0170509442713 | -0.599499953021325 | 0.16156296866648 | -3.71062724317039 | 0.000206746345320582 | 0.00231775787771463 |
| MAJIN | 1.64899935833777 | -1.01939404875351 | 0.234037308635718 | -4.35569035849841 | 0.0000132648180470226 | 0.000269503065084161 |
| CCDC88B | 1986.23556736954 | 0.810910542960178 | 0.136761601085034 | 5.92937298574018 | 3.04093628739496E-09 | 2.97353775798428E-07 |
| GNG4 | 1356.46370321809 | 0.718423628970103 | 0.202616583575178 | 3.5457296549645 | 0.000391527657685687 | 0.00382402684822569 |
| PTF1A | 9.61419678038852 | 1.12873850435134 | 0.42004413730579 | 2.68719023574808 | 0.00720559016418382 | 0.0348221598656987 |
| ACOX2 | 599.983481720882 | 0.531772742292647 | 0.114451707696336 | 4.6462630658474 | 0.0000033800193185729 | 0.0000894969169055541 |
| MTCL1 | 475.049664278312 | -0.576056078602007 | 0.129685841505626 | -4.44193500164793 | 8.91534937365039E-06 | 0.000194106193850011 |
| CHRM1 | 46.9447480870107 | -0.511187471706566 | 0.176830713080018 | -2.89082966868571 | 0.0038422630597177 | 0.0216646368644545 |
| DYNLRB2 | 2.30166104664277 | 0.501651308498245 | 0.187562603245961 | 2.67458064569728 | 0.00748227779475762 | 0.0358536085416656 |
| MUCL3 | 6.73652869640176 | -1.28312934824633 | 0.26199804707848 | -4.89747676577899 | 9.70751080960161E-07 | 0.000033364704675714 |
| AXIN2 | 5004.15888262131 | 0.58339382194467 | 0.117421961855509 | 4.96835355776592 | 6.75237650113452E-07 | 0.0000249241694206336 |
| SHOX2 | 19.2031195585447 | -0.871429411158769 | 0.186700893518193 | -4.66751601846969 | 3.04862988382964E-06 | 0.0000821533284246825 |
| OR13J1 | 1.22011370329074 | 0.909451958805045 | 0.271062358674527 | 3.35513925006848 | 0.000793250434845628 | 0.00659978523184293 |
| FSTL5 | 2.6075176349722 | -0.779678596859153 | 0.281390968749728 | -2.7708017791879 | 0.00559184563734102 | 0.0288406079278159 |
| NTSR2 | 0.607452153977475 | -0.957208054457212 | 0.367834133952537 | -2.60228175175478 | 0.00926057367760428 | 0.0418785807878894 |
| KLK7 | 213.494733083709 | -0.86188429878158 | 0.24741535918614 | -3.48355211906288 | 0.000494806768283177 | 0.00456942588157029 |
| ACTBL2 | 2.70086085568027 | -1.03847926034856 | 0.270590495078713 | -3.83782608493498 | 0.000124128342063401 | 0.00156136972478087 |
| RAB3B | 159.703932589091 | -0.896512183732117 | 0.143010486609839 | -6.26885625652039 | 3.63709682135115E-10 | 0.0000000519793982477 |
| CXCL10 | 969.563979853355 | -0.710397900244426 | 0.168163427447211 | -4.22444945984125 | 0.0000239525595393338 | 0.000429042043982004 |
| CXCL11 | 574.849535086619 | -0.898907940013154 | 0.174875492676362 | -5.14027395294743 | 0.0000002743381858012 | 0.0000119458562624529 |
| AL669983.1 | 24.9848184627117 | 0.557745965993639 | 0.200974674417627 | 2.775205222299 | 0.00551668844007591 | 0.0285675278434529 |
| HSPB3 | 15.1825085787009 | 1.20415170888489 | 0.303365615519072 | 3.96930847559812 | 0.0000720815171010865 | 0.0010135011273833 |
| GP2 | 77.4835674895558 | -1.90411355017607 | 0.292059226949443 | -6.51961442911605 | 7.04883479907028E-11 | 1.3243097379242E-08 |
| PTAFR | 460.19570554922 | -0.501177301091093 | 0.115247295124206 | -4.34871205047334 | 0.000013693937116269 | 0.000276374559470288 |
| CXCL8 | 3907.60016252435 | -0.90152018471299 | 0.182075773966284 | -4.95134616250446 | 7.37018692766132E-07 | 0.000026703552679832 |
| SPRR1B | 29.5228662131198 | -2.00688948979509 | 0.338336315942694 | -5.9316407823481 | 2.99922238105259E-09 | 2.94134882308389E-07 |
| SPRR1A | 29.1850205833056 | -2.96145732273974 | 0.348027791208353 | -8.50925528808362 | 1.75054031172118E-17 | 3.65884806903623E-14 |
| SLC38A11 | 100.103794975507 | 0.695747319789428 | 0.202035915411783 | 3.44368137898343 | 0.000573851535864563 | 0.00512573265555094 |
| CRCT1 | 1.06469412109715 | -2.06164480036207 | 0.587198273726051 | -3.51098579919174 | 0.000446448247050398 | 0.00422112589139367 |
| CT55 | 0.445626753706552 | -1.17620379269126 | 0.39668893213478 | -2.96505321275672 | 0.00302630577798249 | 0.0180886001478624 |
| VPREB1 | 1.44144664524137 | 2.01214518659915 | 0.432225507758524 | 4.65531337341454 | 3.23488308825381E-06 | 0.0000864065177614888 |
| MT1E | 1347.21725425303 | -0.50344149191611 | 0.164647542578146 | -3.05769211026738 | 0.00223048618969 | 0.01431431954627 |
| MUC17 | 2657.81530370187 | -0.746450746865201 | 0.244416245862811 | -3.05401445075863 | 0.00225801203894578 | 0.0144549078496219 |
| WNT10B | 26.2237241103959 | -1.01598214425935 | 0.149807065978916 | -6.78193740475723 | 1.18574851887492E-11 | 3.05029245909347E-09 |
| TM4SF4 | 140.375895046715 | -2.33880161494057 | 0.230571636268641 | -10.1434922906806 | 3.54205505565441E-24 | 2.3690681034239E-20 |
| MYRIP | 345.925449896297 | 0.622985146625263 | 0.134503833523116 | 4.63172781256227 | 3.62626689928023E-06 | 0.0000943732433040695 |
| ZPLD1 | 1.3164972540546 | -1.1130835796471 | 0.254272270431727 | -4.37752641197249 | 0.0000120033818938879 | 0.000248094621319778 |
| SERPINA6 | 95.3282648002536 | 1.0507160211032 | 0.223074536719808 | 4.71015668822365 | 2.47526420789434E-06 | 0.000070269767097116 |
| FOXD4 | 11.1682982356773 | -0.563641997495108 | 0.140009105185173 | -4.02575244481169 | 0.000056793414028103 | 0.000839523692990781 |
| ANKK1 | 12.0155769719603 | 0.538769839847399 | 0.159920075378065 | 3.36899440907403 | 0.000754429591242372 | 0.00634374948898734 |
| FABP6 | 356.06857158147 | 0.546360869668635 | 0.158187607179784 | 3.45387909589962 | 0.000552585049345386 | 0.0049877325830522 |
| C7orf33 | 0.441544948411216 | -1.46284271832367 | 0.529895991791891 | -2.7606223503917 | 0.0057691344159184 | 0.0295273022860642 |
| SLN | 10.561291435979 | -0.740263022232954 | 0.21599489134867 | -3.42722467930033 | 0.000609784307229716 | 0.00536913044517503 |
| LGALS9B | 90.6550550098491 | 1.09342039803597 | 0.180387127615284 | 6.06152119882929 | 0.0000000013484011167 | 1.56031938216494E-07 |
| FABP4 | 37.8254095677745 | -0.891352233112881 | 0.192099946792709 | -4.64004414365988 | 3.48334761321748E-06 | 0.0000912931903457829 |
| TCAF2 | 67.4447023099262 | -0.551026198447337 | 0.10521901150124 | -5.236945211568 | 1.63256129364929E-07 | 7.82334762221706E-06 |
| KRT86 | 27.2918754481594 | -0.670168680142618 | 0.139281179183713 | -4.81162411224752 | 1.49708720128177E-06 | 0.0000468340413332695 |
| KRT6C | 4.8529614234996 | -0.926366606365996 | 0.30303118652477 | -3.05700088822466 | 0.00223563613509059 | 0.0143418652656243 |
| COX7B2 | 0.477822399981809 | -1.83105299503012 | 0.70028531854036 | -2.61472423675349 | 0.00892995216399246 | 0.0407971940257153 |
| GTSF1 | 11.5768440813291 | -1.43515191914312 | 0.185614000595903 | -7.73191631307794 | 1.05939582388037E-14 | 8.05188980504709E-12 |
| SDR16C5 | 542.67489491976 | -0.712936365285723 | 0.180194339346112 | -3.95648591333569 | 0.0000760603712269523 | 0.00105587834560886 |
| CELP | 184.146839420997 | 0.870596295292297 | 0.20238421529115 | 4.30170057501697 | 0.0000169492250540877 | 0.000325569204054451 |
| CEL | 1422.96387522522 | 0.845123986772705 | 0.239681021367258 | 3.52603632090562 | 0.000421829113049653 | 0.00404670372880278 |
| LILRP2 | 2.8600313266238 | -0.657571925754843 | 0.177991976626267 | -3.6943908271526 | 0.000220414567073253 | 0.00243753437568244 |
| CYTL1 | 17.4398678351469 | -0.535181191924341 | 0.149519530756208 | -3.57933969707914 | 0.00034446346962539 | 0.00346596829814269 |
| ZNF415 | 68.9778422773363 | 0.657164019421797 | 0.141233045787888 | 4.65304713748626 | 3.27065442686849E-06 | 0.0000870837781395987 |
| CEACAM3 | 28.7894730487903 | -0.505108423804045 | 0.135912502176708 | -3.7164235498168 | 0.000202062744006654 | 0.00227751340919128 |
| DCDC1 | 2.77630323471658 | -0.955482321735604 | 0.207367959786691 | -4.60766611543298 | 4.07213852689732E-06 | 0.000103638094837519 |
| FPR2 | 58.7624209031352 | -0.738592557212884 | 0.190728131328519 | -3.87248882515762 | 0.00010772961673278 | 0.00139217173690371 |
| MUC7 | 1.06228732846285 | -1.05640214847506 | 0.409229521450597 | -2.58144169250162 | 0.00983886100876194 | 0.0437265278858975 |
| UGT2B7 | 158.36183503382 | -0.602319873285836 | 0.186230484448878 | -3.23427109728202 | 0.00121953635260403 | 0.00906708197060562 |
| ZNF439 | 97.5445261792236 | 0.552181775889469 | 0.123710188715941 | 4.46351090092804 | 8.06274944322118E-06 | 0.000179516955313051 |
| KRT9 | 1.62527126991785 | -0.824433600888591 | 0.276297318988594 | -2.98386391842849 | 0.00284633330732338 | 0.0172659311560871 |
| CDK5R2 | 10.0160031828862 | -0.583810127259155 | 0.184319796360348 | -3.16737615159795 | 0.0015382121367659 | 0.010831941519841 |
| MROH2B | 0.751589137176499 | -1.26620334695373 | 0.413421018633986 | -3.06274545773576 | 0.00219316526278122 | 0.0141235957477238 |
| FGG | 6.70463703184956 | -0.963822854741612 | 0.356229407099372 | -2.70562406003935 | 0.00681761776296219 | 0.0334009336696428 |
| DNAI2 | 2.08724499600044 | 0.714224108555892 | 0.216213343250132 | 3.30333039496839 | 0.000955437455160601 | 0.00755537584022485 |
| UTF1 | 0.836050928426747 | 1.73772466042836 | 0.346305069653766 | 5.01790130351176 | 0.0000005223900035055 | 0.000020103298615916 |
| CFAP46 | 13.9431259951257 | -0.680623417277157 | 0.21541321678562 | -3.15961772185277 | 0.00157976265712584 | 0.0110709184366308 |
| PCDHB1 | 0.655678661472857 | -0.902771778549106 | 0.328263720840724 | -2.75014179525229 | 0.00595694812486821 | 0.0301974017268217 |
| ZNF570 | 100.586634223654 | 0.759742672993442 | 0.117832684320012 | 6.44763952699336 | 1.13605570846441E-10 | 2.03165641724421E-08 |
| RPS21 | 31350.2406685114 | 0.764263725465953 | 0.10470911099349 | 7.29892287513996 | 2.9008052778028E-13 | 1.42673414354903E-10 |
| ADRA1D | 10.1258604152455 | 0.516678208595826 | 0.156060025472333 | 3.31076588660064 | 0.000930410140493007 | 0.0074082799805636 |
| MIR31HG | 7.40333487985458 | -0.846610712069642 | 0.268763099796383 | -3.15002585068799 | 0.00163256016453402 | 0.0113505357634817 |
| LGALS9C | 107.596596194879 | 1.06630986615584 | 0.177567821241498 | 6.00508503568123 | 1.91231652013171E-09 | 2.06295771181434E-07 |
| FBXW10 | 1.56330145572089 | -1.04606163298714 | 0.245354492580537 | -4.26347046669128 | 0.0000201276114008833 | 0.000374781503601524 |
| SRGAP2C | 52.7731761765487 | -0.518702297919941 | 0.111851954564992 | -4.63740039177005 | 3.52818543954135E-06 | 0.0000920355518480045 |
| SCG2 | 105.031156912484 | -0.973676231128881 | 0.151032731485595 | -6.44678952404266 | 1.14244266437398E-10 | 2.03221104159547E-08 |
| FOXB1 | 2.12581587845936 | -0.848194326383204 | 0.236780421016476 | -3.58219789770617 | 0.000340715524828361 | 0.00343199053653917 |
| REG1B | 1165.16247074111 | -1.37138579576125 | 0.296328186044526 | -4.62792896641695 | 3.69340645737285E-06 | 0.0000958222643502428 |
| CALB2 | 114.305466953665 | -1.26486323555272 | 0.200720489110862 | -6.30161495299123 | 0.0000000002945601956 | 4.32047458822141E-08 |
| TP53RK | 1325.39785724322 | 0.503497749836683 | 0.0727736041647212 | 6.91868646078088 | 4.55850511087446E-12 | 1.48005366910547E-09 |
| CLEC12A | 33.8496029241313 | -0.577825425916691 | 0.158788040844812 | -3.63897320505023 | 0.000273727275603964 | 0.00290418386762302 |
| ARNT2 | 215.247528406215 | -0.617427372008748 | 0.144753718802092 | -4.26536449024082 | 0.0000199576116473656 | 0.000372237840887452 |
| FGGY | 1163.01908261861 | 0.843317811336803 | 0.146387442383236 | 5.76086170785767 | 0.0000000083685586448 | 0.0000006876199955763 |
| PRSS30P | 37.6645100897901 | 0.629922511465856 | 0.147320729911068 | 4.27585793150847 | 0.0000190402620353064 | 0.000357721597182425 |
| AGXT | 37.1597997634228 | 0.605911219137167 | 0.162948560963639 | 3.71842019072739 | 0.000200472607924626 | 0.00226323068695019 |
| CTSW | 94.7201659440319 | -0.617160012599407 | 0.148362309547661 | -4.15981669792721 | 0.0000318503105460255 | 0.000535245268985017 |
| MUCL1 | 10.7419121186804 | 0.961885088287508 | 0.290028010326051 | 3.31652479774678 | 0.000911444984306762 | 0.00730597870689999 |
| COL6A5 | 5.2398398077496 | -0.89852229723625 | 0.21520671351543 | -4.17515923438804 | 0.0000297777690815261 | 0.000511467978235437 |
| HOXC5 | 1.16369971690411 | -1.87591959062984 | 0.29086355702525 | -6.44948308346168 | 0.0000000001122322863 | 2.01788823578497E-08 |
| CES4A | 97.8511898999055 | 0.672253335571379 | 0.116122301375115 | 5.7891837107135 | 7.07293065505816E-09 | 5.98817587256848E-07 |
| LVRN | 4.95606317658198 | -0.726831218606544 | 0.152660992260834 | -4.76108014131529 | 1.92559557319102E-06 | 0.000057622536945052 |
| HPSE2 | 16.59301286148 | 0.509890624287383 | 0.138483722112613 | 3.68195349250325 | 0.000231453639518164 | 0.00253115520365155 |
| DELEC1 | 15.6790541437232 | 0.787551734256318 | 0.183172892228231 | 4.29949936737931 | 0.0000171184364534829 | 0.000327690184245779 |
| RXFP4 | 86.8746187449137 | 0.632763216957635 | 0.129772764662323 | 4.87593231603045 | 1.08295739336444E-06 | 0.00003607197325587 |
| HSPA6 | 162.54079943427 | -0.71224692876017 | 0.140983450923284 | -5.05198960655134 | 4.37231498288819E-07 | 0.0000172835647349582 |
| ADAMTS20 | 1.46275201823424 | -2.06888356996599 | 0.537568908177937 | -3.84859231717543 | 0.000118798526997805 | 0.00151231836309882 |
| ABLIM3 | 281.973260197904 | -0.99511468203222 | 0.126354533006854 | -7.87557563904922 | 3.39177605743029E-15 | 3.23334781717419E-12 |
| SYT12 | 33.523402445246 | -0.993303332355848 | 0.164236318583142 | -6.04801264985127 | 1.46643445274333E-09 | 1.66238986334381E-07 |
| SLC2A14 | 11.2948721088491 | 0.639580398015455 | 0.204902507608857 | 3.12138882768758 | 0.00180000196640565 | 0.0122006636074998 |
| IQCF1 | 0.882326418788036 | -0.899472322871709 | 0.273982371663689 | -3.28295691949043 | 0.00102724362774704 | 0.00795832199377359 |
| INSM1 | 52.0433764508812 | -1.18001843267665 | 0.232568598984138 | -5.0738510608525 | 3.89844232769492E-07 | 0.000015756379950508 |
| AGR3 | 1237.10385218103 | -0.557779041902925 | 0.158572156478163 | -3.51750934269308 | 0.000435617015544524 | 0.00414331747265073 |
| XCR1 | 30.2872966346779 | -0.812517849000336 | 0.179494604210346 | -4.52669790590564 | 0.0000059912490592279 | 0.000141797134493064 |
| TAS1R1 | 3.31400145328273 | 0.662545536692411 | 0.210147026937529 | 3.15277140175468 | 0.00161728402828374 | 0.0112701005363336 |
| GPR160 | 2968.24815601574 | 0.571561602382059 | 0.0898649752417256 | 6.36022656039942 | 2.01456382741953E-10 | 3.27043900565845E-08 |
| C9orf131 | 1.48679962326541 | 0.667514936085338 | 0.216353871125591 | 3.08529231583682 | 0.00203352186569947 | 0.0133474069151563 |
| TLR6 | 49.9300899398919 | -0.505116298048259 | 0.111580516121738 | -4.52692204342523 | 5.98490055339073E-06 | 0.000141747198517346 |
| FAM53A | 60.3983614078238 | 0.506045671529849 | 0.0956431450461984 | 5.29097690467429 | 1.21664730982229E-07 | 6.15586422414571E-06 |
| NWD2 | 9.21030724178992 | -0.932805381460521 | 0.17478940179825 | -5.33673879459355 | 9.46332147254791E-08 | 5.03936937396413E-06 |
| AC020659.1 | 76.0954054796618 | 0.609387279695893 | 0.193918756268231 | 3.14248756243559 | 0.00167518830027969 | 0.0115675505137216 |
| ZNF80 | 2.53517295540704 | -0.699941899048508 | 0.206878576080176 | -3.38334646491982 | 0.000716082335906937 | 0.00609809663290038 |
| EVX2 | 2.29454227920941 | 0.672736809829908 | 0.235563022023559 | 2.85586763173138 | 0.00429194027163514 | 0.0235489854904056 |
| CHRNA9 | 1.25307434409554 | -0.88364452496796 | 0.239275853425303 | -3.69299497763076 | 0.000221628401783464 | 0.00244933807417138 |
| SNHG11 | 443.158052545099 | 0.532661801324205 | 0.0713704245465333 | 7.46334079849716 | 8.4355786195007E-14 | 5.03754678916683E-11 |
| GOLGA6L2 | 6.11883812669852 | 1.19408786808174 | 0.337656622103589 | 3.53639700783186 | 0.000405624625719801 | 0.00392820282843636 |
| SLC26A9 | 31.0242138206439 | -2.25593856438579 | 0.2539818720914 | -8.88228181723912 | 6.55026348655267E-19 | 1.68503008859457E-15 |
| IL20RB | 41.5062700299502 | -0.512332228567791 | 0.128027560387515 | -4.00173390024038 | 0.0000628799930562073 | 0.000909669943972599 |
| WDR49 | 1.223363124854 | -0.758607264198512 | 0.277092323298915 | -2.73774190192978 | 0.0061862605076089 | 0.0310557430206593 |
| SLC66A1L | 14.5354395792087 | 0.88983531151703 | 0.209469131998813 | 4.24804983448384 | 0.0000215639433392941 | 0.000396122001572885 |
| AMZ1 | 13.4055808902629 | -0.586604169508 | 0.139489663719247 | -4.20535940705018 | 0.0000260667589173547 | 0.000461493759641258 |
| CD164L2 | 13.2832665015014 | -0.687109579792932 | 0.161200041324215 | -4.26246528318796 | 0.0000202183917181684 | 0.000376053090010561 |
| DES | 4880.738746101 | 0.720140324512764 | 0.22727396414408 | 3.16860018359265 | 0.00153174948128397 | 0.0107960646256688 |
| ISX | 636.495922650033 | 0.509754216743997 | 0.149660191142327 | 3.40607754709615 | 0.000659034569147705 | 0.00569494420192185 |
| CHRNA7 | 26.1903419231482 | -1.15032600538773 | 0.209366222537813 | -5.49432468831009 | 3.92208318551881E-08 | 2.50309744065115E-06 |
| PCSK1 | 863.954262588862 | -1.27774969475022 | 0.255813239177763 | -4.99485366299716 | 5.88803971008459E-07 | 0.000022149361528082 |
| DPP10 | 59.7762906694486 | 1.10331694600105 | 0.274584136770191 | 4.01813797030982 | 0.0000586598485503457 | 0.000861151297287384 |
| ACRP1 | 22.4202383093282 | 0.663199526346371 | 0.148690768608991 | 4.46026026061088 | 8.18601833236022E-06 | 0.000181511228000762 |
| FOSL1 | 587.678217212581 | -0.995531391843645 | 0.130101761138559 | -7.65194401006914 | 1.97963436660381E-14 | 1.40857303165882E-11 |
| LINC02873 | 0.683764934515274 | -0.982167916262917 | 0.315174531669896 | -3.11626675879893 | 0.00183156505961503 | 0.0123488824535493 |
| FAM172BP | 1.49539406407595 | -0.783898989144442 | 0.212272236733922 | -3.69289456410185 | 0.000221715963190298 | 0.002449496280479 |
| CREG2 | 47.7271835344674 | -0.928160623678534 | 0.192840990508694 | -4.8130878255196 | 1.48615937133203E-06 | 0.0000466218466642063 |
| TSPEAR | 61.2051978140725 | 0.779112488555567 | 0.190966784652518 | 4.07983246915548 | 0.0000450681693678385 | 0.00070771401129486 |
| PLEKHD1 | 4.04967463672036 | -0.688818263269548 | 0.157005464391094 | -4.38722477552585 | 0.0000114806128358044 | 0.000238765332372493 |
| AC007160.1 | 14.4319798066296 | 0.608403196592904 | 0.234604146928946 | 2.59331816831511 | 0.00950547881449816 | 0.0426630281189703 |
| JAKMIP2 | 20.6362138298343 | -0.603069406911582 | 0.138327618934364 | -4.35971797647828 | 0.0000130230173821006 | 0.00026523492526931 |
| ZNF683 | 45.8772078118452 | -0.864339703072967 | 0.192904115274628 | -4.48067010826829 | 7.44090470594571E-06 | 0.000168020752988681 |
| SPHK1 | 493.650011079964 | -0.610724621639787 | 0.130031274106209 | -4.69675180711489 | 2.64331315989237E-06 | 0.0000739729528812724 |
| CYCSP34 | 16.9048950623807 | 0.508160514111663 | 0.119307743173201 | 4.25924169375961 | 0.0000205121584067142 | 0.000380248116096084 |
| AL033381.1 | 1.04538761365287 | 0.724799930455442 | 0.232298384615498 | 3.12012471225375 | 0.00180774486192179 | 0.0122402518065172 |
| NANOGP1 | 0.858528458132851 | 0.792502156852812 | 0.285263437687592 | 2.77814136742166 | 0.00546708269864864 | 0.0283545563908511 |
| MIR7-3HG | 0.823620775568087 | -0.899199919036573 | 0.320014480726247 | -2.80987259387735 | 0.00495611151320369 | 0.0262916055242002 |
| GRIN1 | 94.1996162773773 | -0.743330955471834 | 0.165520683916848 | -4.49086445199355 | 7.09346852717414E-06 | 0.0001620353650859 |
| HCG4 | 2.83129263454064 | -0.518522834190526 | 0.192110748368011 | -2.69908289148525 | 0.00695308554433577 | 0.0338701106858884 |
| C19orf18 | 10.3996915285286 | 0.5716780704554 | 0.171716144255115 | 3.3292039775018 | 0.000870945931922464 | 0.00706774420173527 |
| FBXO39 | 6.44845248285185 | -1.07191929543222 | 0.178243688918317 | -6.01378540770352 | 0.0000000018124039831 | 1.96150207129138E-07 |
| KCNA2 | 7.58845314641956 | 0.920260427484594 | 0.199775191698449 | 4.60648001216128 | 4.09542406896177E-06 | 0.000104151461379635 |
| RPL13AP3 | 20.1043015048191 | 0.512725699540154 | 0.12931741877189 | 3.96486184467213 | 0.0000734384683415601 | 0.00102640217876078 |
| AC024940.1 | 17.5712863239177 | -0.512442238106013 | 0.143346356527888 | -3.57485359599158 | 0.00035042392914078 | 0.00351440724366852 |
| UMODL1 | 47.9339049718514 | 1.30135547128736 | 0.183209520252881 | 7.10309960689336 | 1.21989415385006E-12 | 4.74368608058766E-10 |
| SAMD9L | 596.037618456029 | -0.724164455751841 | 0.115682309708587 | -6.25994119218459 | 3.85122675493778E-10 | 5.38881695140707E-08 |
| AL161742.1 | 5.52498864166019 | 0.796999086085117 | 0.234156880837536 | 3.40369705658189 | 0.000664804408413881 | 0.00574035347951898 |
| ERICH5 | 53.4540319957094 | 0.901633875965066 | 0.179977080125988 | 5.00971498889693 | 5.45107021912767E-07 | 0.0000208098961493228 |
| ZBED2 | 39.8185630951271 | -1.28957727998706 | 0.177482989403763 | -7.2659204373291 | 3.70506626786559E-13 | 1.77006894471373E-10 |
| CD163 | 735.300831866876 | -0.593268179546157 | 0.140444033517708 | -4.22423199253497 | 0.0000239756977454883 | 0.000429226597432879 |
| NAALADL2 | 487.822521228815 | 0.579708818307536 | 0.111572581392146 | 5.19579999919536 | 2.03841319119034E-07 | 9.27464135235204E-06 |
| AC112247.1 | 4.49817257672185 | 0.732511819543991 | 0.257678110558286 | 2.84273979639532 | 0.00447275701045046 | 0.0243493309365919 |
| TENM3-AS1 | 9.6989349734111 | -0.794580276392286 | 0.219979739098401 | -3.61206118185664 | 0.000303772870465941 | 0.00314318450931992 |
| LCN15 | 1077.2285712286 | 1.54106936764619 | 0.311818145874963 | 4.94220553881475 | 7.72437178290535E-07 | 0.000027657220681362 |
| ZNF518B | 318.832099198542 | 0.589539586637143 | 0.129817650546142 | 4.54128991055495 | 5.59110842180155E-06 | 0.000134226739297837 |
| AMER3 | 5.43303452666638 | 1.21045275358068 | 0.292276154552131 | 4.14146941078898 | 0.0000345087901031776 | 0.000568774252651783 |
| CALML3 | 23.4955068101021 | 0.685866295861528 | 0.209529679503664 | 3.27336106983133 | 0.00106276621660902 | 0.00816659646503647 |
| NEUROG2 | 13.4483159599559 | 1.41709898926631 | 0.320421188100669 | 4.42261324123514 | 9.75142093937953E-06 | 0.00020904296093252 |
| GLDC | 85.6847232884853 | 0.964420631208851 | 0.211801767368802 | 4.55341163196973 | 5.27828491284365E-06 | 0.000128268576199506 |
| H3P16 | 18.0182905892615 | -1.26422757287887 | 0.174254880664168 | -7.25504828364234 | 4.01518579132506E-13 | 1.86494226713184E-10 |
| MCMDC2 | 69.9505626566027 | 0.507181912586238 | 0.12664717075543 | 4.00468411225439 | 0.0000621004089989331 | 0.000900709069827666 |
| CA8 | 338.967284526186 | -0.66284273726256 | 0.194372388407156 | -3.410169225652 | 0.000649225826316074 | 0.00562909258067466 |
| PSAPL1 | 5.00114573591099 | -2.0908117556128 | 0.365867361229721 | -5.71467142787853 | 1.09916194108513E-08 | 8.62867925675329E-07 |
| GLULP4 | 2.28971446049446 | 0.558393669414412 | 0.210535333641166 | 2.65225632086123 | 0.00799558183293816 | 0.0376444104824888 |
| CPNE7 | 1077.04093786929 | 0.804698372159785 | 0.137472540146385 | 5.85352079261007 | 4.81274053270969E-09 | 4.31495090870985E-07 |
| C5orf46 | 17.8219563682498 | -0.74176575512884 | 0.207497587364236 | -3.57481628847454 | 0.000350473899815637 | 0.00351440724366852 |
| RIIAD1 | 28.6643215580693 | 0.762838358093951 | 0.192216363490954 | 3.96864421030342 | 0.0000722827090057253 | 0.00101474101106071 |
| APOLD1 | 1598.04295099519 | 0.687117730790217 | 0.156215744732681 | 4.3985177804326 | 0.0000108992698623451 | 0.000229819282936031 |
| SMIM10L2A | 36.0881232865488 | 0.558684884720146 | 0.14144062747445 | 3.94996045122232 | 0.0000781641073870874 | 0.00108015044596652 |
| ERICH3 | 1.19745359118793 | -0.937984806448328 | 0.261405708064121 | -3.58823383542278 | 0.000332925634838604 | 0.0033769181317175 |
| IGSF22 | 39.9975324745371 | 0.651148686450234 | 0.116257519924033 | 5.60091671382394 | 2.13221171181531E-08 | 1.54007395392068E-06 |
| C9orf50 | 8.73272872106704 | 0.545988963664866 | 0.128788175811195 | 4.23943394046741 | 0.0000224084121901584 | 0.000407716061187855 |
| AC020907.1 | 7.86859889752761 | -0.698977146190391 | 0.269446652243651 | -2.59412072991106 | 0.00948331796672979 | 0.0425921460439669 |
| TAAR3P | 2.37587050514358 | -1.35462862763993 | 0.291165173324285 | -4.65244044187665 | 3.28029498548993E-06 | 0.0000872709824222388 |
| FAM133A | 1.429240440917 | -1.20492996034461 | 0.290926249758495 | -4.14170244639268 | 0.0000344737396617221 | 0.000568587292949104 |
| HES7 | 5.99313288047153 | -1.18042378674616 | 0.215948728909006 | -5.46622243487968 | 4.59727386966383E-08 | 2.80040132512382E-06 |
| ALOXE3 | 11.1676231680846 | -1.46616167670214 | 0.180472188822578 | -8.12403111120641 | 4.50950092481853E-16 | 5.69215309817734E-13 |
| LINC00311 | 0.842947169488006 | -0.6924670150991 | 0.242366275658989 | -2.85710960906709 | 0.00427518177314123 | 0.0234858547298611 |
| MEIS3P1 | 39.7586561945789 | 0.510277100771062 | 0.133165408378747 | 3.83190429844767 | 0.000127155204575975 | 0.00158846632477764 |
| RAB39A | 12.9801724803345 | 0.874872804214229 | 0.170998284092884 | 5.11626656872774 | 0.0000003116425451276 | 0.0000131923417647561 |
| GPC5 | 5.70185293692677 | 0.617033209732278 | 0.233932515126632 | 2.63765475012427 | 0.0083481526157877 | 0.0388125844261327 |
| ALOX12B | 17.9446349869479 | -0.649465702034432 | 0.17723550199702 | -3.6644221655171 | 0.000247897610057168 | 0.00268464762808672 |
| SLITRK4 | 8.82965155920403 | -0.770933860865682 | 0.201193119797733 | -3.83181026091116 | 0.000127203827390366 | 0.00158848035682921 |
| HTR1D | 316.923693887894 | -0.506015244545426 | 0.139253541314461 | -3.63376930862209 | 0.00027931061702261 | 0.00294938606077365 |
| CIITA | 736.042742923114 | -0.564854574817965 | 0.145706423691698 | -3.8766621299631 | 0.000105899252886041 | 0.00137586744950077 |
| GRM8 | 521.406936173707 | 0.982045734881659 | 0.181174771696737 | 5.42043313031174 | 5.94548090258289E-08 | 3.46391589449786E-06 |
| TPPP2 | 1.60167085341285 | 0.602686768820584 | 0.19942849981116 | 3.02206941029628 | 0.00251052974430424 | 0.0156853464290457 |
| NKPD1 | 29.9127878783871 | 0.605205401666801 | 0.144346469065377 | 4.19272743964865 | 0.000027562052199792 | 0.000481824437880524 |
| TH | 46.5894833201141 | 0.580924523832626 | 0.187601790595994 | 3.09658304426135 | 0.00195765019377499 | 0.0129554601064257 |
| TDRP | 69.930335936812 | 0.620641523023979 | 0.129871155631504 | 4.77890198178404 | 1.76255087311879E-06 | 0.0000537312910654864 |
| MYLPF | 13.5719619533658 | 0.71219439273399 | 0.1346006563475 | 5.29116582385238 | 1.21539103573948E-07 | 6.15586422414571E-06 |
| SLC9A4 | 5.12996229037458 | -0.562857911734787 | 0.216453573046276 | -2.60036322715012 | 0.00931251329912099 | 0.0420395612512425 |
| ADGRD2 | 4.47448817966117 | 0.882408980678358 | 0.22527124282536 | 3.91709554051886 | 0.0000896222363737022 | 0.00121096841568054 |
| KCTD4 | 2.84945171493291 | 0.766031532857632 | 0.213829132744339 | 3.58244698945453 | 0.000340390705794631 | 0.00342975172738296 |
| TPRXL | 40.7870968071207 | -0.688105031463866 | 0.228755388537906 | -3.00803856845471 | 0.0026293976576653 | 0.0162117102632085 |
| GAS1 | 205.183808988921 | -0.759111414721792 | 0.1745119075044 | -4.34991185173225 | 0.0000136192267405161 | 0.000275396641094898 |
| AC022148.1 | 1.29281359124268 | 0.64759922348858 | 0.234140662154648 | 2.76585543719376 | 0.00567736966744276 | 0.0291946244157133 |
| KCNE1 | 4.47207108715744 | -0.584120443925698 | 0.169067361483566 | -3.45495688109189 | 0.000550380835696522 | 0.00497052009380586 |
| ZSCAN4 | 2.45918275436792 | -0.750524324566556 | 0.270624712199724 | -2.77330299389902 | 0.00554904277616864 | 0.0286729123177737 |
| PRF1 | 277.313503276248 | -0.610741191655138 | 0.131551950873967 | -4.64258559145396 | 3.44076028284272E-06 | 0.0000904739119136537 |
| CHST13 | 86.5351663969382 | 0.51070771737034 | 0.173507148058547 | 2.94343906337513 | 0.00324587715967202 | 0.019077046203678 |
| ARSJ | 318.353782314391 | -0.661376807962057 | 0.122196760851219 | -5.41239230364968 | 6.21882577479668E-08 | 3.60238573698998E-06 |
| HOXC9 | 14.0584998019794 | -1.21252704747824 | 0.209214906933247 | -5.79560541479537 | 6.80751514868538E-09 | 5.83735696416247E-07 |
| MAP3K15 | 4.62769073881524 | -0.80310183206441 | 0.150189857976107 | -5.34724410081121 | 8.93035411325076E-08 | 4.79815328340607E-06 |
| HOXC10 | 16.6938828727998 | -2.6045242927926 | 0.261903966687062 | -9.94457749433265 | 2.66301656883648E-23 | 1.48427666825049E-19 |
| LINC01555 | 29.4270067815511 | 0.632845617336354 | 0.159116489103086 | 3.97724724133624 | 0.000069717666025688 | 0.000987086446753199 |
| GPR62 | 2.0757695287331 | 0.53169715072383 | 0.200631688325981 | 2.65011551844164 | 0.00804642533909587 | 0.0377933365435455 |
| C1orf105 | 12.9842901741453 | 0.577917716815774 | 0.17287846395858 | 3.34291330211169 | 0.000829037969070621 | 0.00682201962639264 |
| FCRL6 | 14.5079014656011 | -0.673898649921807 | 0.154497382720582 | -4.36187745096366 | 0.000012895109638066 | 0.00026295015641232 |
| ADIPOQ | 11.6469525716316 | -1.65839994569691 | 0.509850299251282 | -3.25271937298512 | 0.00114306324356599 | 0.00865828335024551 |
| LINC02878 | 21.8010605313623 | 0.53756744166425 | 0.106183682436393 | 5.06261818510831 | 4.13537542219254E-07 | 0.0000165227269855392 |
| MUC16 | 45.3517188945694 | -1.14090923836701 | 0.261542774068678 | -4.36222809989552 | 0.0000128744538135259 | 0.000262811453701452 |
| TMEM132E | 7.74691351084933 | 0.548197626065027 | 0.172199667658744 | 3.18349990751095 | 0.00145506159803799 | 0.0103753027636645 |
| CCL13 | 75.6775364064786 | -0.606059782637547 | 0.187530109039576 | -3.23179987331872 | 0.00123013176025309 | 0.00912703139316901 |
| SYNE4 | 298.678931820762 | 0.625979618462785 | 0.16581005738728 | 3.7752813570331 | 0.000159827160963632 | 0.00189592078378203 |
| UTS2R | 5.62524928384644 | 0.815925936429923 | 0.26076725986055 | 3.12894316896322 | 0.00175436235937605 | 0.0119831262300355 |
| SAGE1 | 1.35304636772075 | -2.20262657258903 | 0.462197737660447 | -4.76555031129812 | 1.88338892593907E-06 | 0.0000567937713807524 |
| SOX2 | 103.009530411716 | -0.961190441119111 | 0.313889771597449 | -3.06219102402546 | 0.00219723183379875 | 0.0141420434431428 |
| AC011890.1 | 1.32868710374833 | 0.913028388992009 | 0.271202023414968 | 3.3665987351244 | 0.000761013338753845 | 0.00638319741023479 |
| FDCSP | 43.8994544078678 | -1.25976858404052 | 0.240214149634844 | -5.24435627940954 | 1.56828895927092E-07 | 7.55716417520721E-06 |
| TMEM252 | 84.2595913378739 | 1.56280518471856 | 0.229749156115143 | 6.80222383030356 | 1.03016372949061E-11 | 2.7166957044776E-09 |
| TIGIT | 115.999642151582 | -0.548729360904147 | 0.129518734043325 | -4.23667946538602 | 0.0000226849615025369 | 0.000411404817010759 |
| USH1G | 4.10189658205664 | -1.47118369819735 | 0.256873220573099 | -5.72727548210378 | 1.02056370193396E-08 | 0.0000008106815040398 |
| FAM181B | 5.44377011207975 | 0.568020294388413 | 0.189676192229802 | 2.99468419157333 | 0.002747291261218 | 0.0168145890112834 |
| ZNF716 | 1.02572398019843 | -1.90232197124956 | 0.612240710501255 | -3.10714713775255 | 0.001889023516612 | 0.0126547925565983 |
| KCNIP1 | 2.91421136128551 | -0.561168925168166 | 0.20043617519236 | -2.79973874291708 | 0.00511439810951835 | 0.026955981336251 |
| ZAR1 | 2.0941134812471 | 0.898678690586326 | 0.275592414172598 | 3.26089777646601 | 0.00111060070998486 | 0.00847380993459128 |
| GABRG3 | 1.18165848025499 | -1.39045330805706 | 0.355273401970786 | -3.91375571698834 | 0.0000908715691501611 | 0.00122389328051538 |
| FAM87A | 6.93267965016742 | 0.667724260110619 | 0.160739125281481 | 4.15408668512611 | 0.0000326589357763522 | 0.000545544520595789 |
| NXPH4 | 255.637716132705 | -0.967248481364613 | 0.232621386362276 | -4.15803764430436 | 0.0000320993136339037 | 0.000538347666271318 |
| ACTL10 | 304.318961532217 | 0.640676878295243 | 0.101398900621587 | 6.31838091308506 | 2.6431786089286E-10 | 3.94612406427635E-08 |
| HS3ST4 | 8.71054142400622 | 1.74979592034667 | 0.314252252524209 | 5.56812530790649 | 0.0000000257494628183 | 1.78650176558209E-06 |
| PAPPA | 92.7432023216854 | -0.52032992595776 | 0.121013558875416 | -4.29976550390889 | 0.0000170978927625478 | 0.000327484381308776 |
| HCAR2 | 73.5938190683435 | -0.764095344154506 | 0.205191564636865 | -3.72381459981922 | 0.000196235058094866 | 0.00222986504003008 |
| MAGEB17 | 79.3568962262918 | 1.33411944913425 | 0.290454196938872 | 4.59321801232236 | 4.36462666796586E-06 | 0.000109360180164726 |
| PLCXD3 | 6.63686204659286 | -1.18255864227562 | 0.251670981063155 | -4.69882795894878 | 2.61658749870154E-06 | 0.0000734912228827144 |
| LCK | 493.062259183474 | -0.799500449100599 | 0.126042168776092 | -6.34311879003663 | 2.25159678552049E-10 | 3.55178772176303E-08 |
| ZNF662 | 75.6542397667658 | 1.00275943727551 | 0.158704794007413 | 6.31839412002052 | 2.64295277694698E-10 | 3.94612406427635E-08 |
| MCEMP1 | 52.7393468013964 | -0.507633978918264 | 0.188590275931936 | -2.69172933975384 | 0.00710826068265964 | 0.0344413870978707 |
| SLC25A21 | 19.7261853404957 | -1.15016073803423 | 0.209848170253151 | -5.48091859293664 | 4.2312315315769E-08 | 2.63502504430158E-06 |
| PCP4 | 106.217929315541 | 1.06905903174364 | 0.244042107226761 | 4.38063350580022 | 0.0000118334761726305 | 0.000245204735557667 |
| ABAT | 905.7049411084 | 0.745257548140565 | 0.120509441566398 | 6.18422538892891 | 6.24081485157434E-10 | 8.0893538862926E-08 |
| CAMK1D | 1152.14732189835 | 0.521118872181132 | 0.103934137411107 | 5.01393368109526 | 5.33283739393044E-07 | 0.0000204519206568603 |
| BEGAIN | 43.1380671834724 | -0.525336316181554 | 0.128008096189727 | -4.10393039048819 | 0.0000406189725636049 | 0.000646847466891464 |
| RIPPLY3 | 41.5337921496546 | 0.971521657643014 | 0.186435813105839 | 5.21102486404521 | 1.87800278101448E-07 | 8.69856647042824E-06 |
| C2CD4C | 35.5832894503243 | -0.94926968540643 | 0.162649989762532 | -5.83627264159291 | 5.33815197581773E-09 | 4.71277470077318E-07 |
| CHST6 | 112.986620781566 | -1.24710651294386 | 0.14987561882132 | -8.32094321112127 | 8.72660556806608E-17 | 1.45917571703633E-13 |
| CCBE1 | 28.8225387348807 | -0.723357446485077 | 0.177319188946333 | -4.0794087249294 | 0.0000451503929351809 | 0.000708549714002026 |
| GBP6 | 3.27352379961769 | -0.706473966732745 | 0.19625970915144 | -3.59968925760309 | 0.000318597680841904 | 0.00325926694485009 |
| TMEM89 | 7.90294203715317 | 0.568147639503247 | 0.152647604313066 | 3.72195582144893 | 0.000197685615810064 | 0.00223949944577241 |
| URAD | 245.18547998629 | 0.723719671699473 | 0.236239450744706 | 3.06350048401343 | 0.00218763851116031 | 0.0140961478015844 |
| KLRC4 | 1.4266470501743 | -0.845311597512203 | 0.222331674980681 | -3.80202954700744 | 0.000143515606594425 | 0.00174652435070261 |
| IZUMO1R | 0.861081827662729 | -0.86184748618583 | 0.289442223757645 | -2.97761492776351 | 0.0029050070878727 | 0.017526474297788 |
| OPCML | 14.6731104282564 | 0.565814693286755 | 0.169617047660724 | 3.33583623279733 | 0.000850432451866471 | 0.00695358485460111 |
| ASCL2 | 4061.46466709591 | 0.710405806269624 | 0.139367819211685 | 5.09734464016109 | 3.4445089538411E-07 | 0.0000142563451032616 |
| ACP7 | 2.74646611212033 | -0.948718070397576 | 0.305432740040005 | -3.10614399187629 | 0.00189544392607359 | 0.0126901773324831 |
| FOXL2 | 1.73227793101594 | -0.932809554952798 | 0.277793389172753 | -3.35792567897542 | 0.000785297245934141 | 0.00655644372017821 |
| AIFM3 | 1125.62958622796 | 0.600118710438404 | 0.135574052957068 | 4.42650121722364 | 0.0000095773842249876 | 0.000205839899262233 |
| KCTD16 | 22.5544102981435 | 0.661171983262469 | 0.219084335032081 | 3.01788798895938 | 0.00254542969409076 | 0.015845915828329 |
| SLC35F3 | 2.93854830665544 | -0.844371426919753 | 0.212366217796013 | -3.97601575091764 | 0.0000700794858162242 | 0.000991369782007685 |
| ANKRD45 | 7.31338257820477 | -0.68951718305478 | 0.197487586844796 | -3.4914456856302 | 0.000480414132403121 | 0.00446526109389249 |
| ZNF730 | 7.7198035160236 | 0.671690240297128 | 0.236965505039579 | 2.83454859889813 | 0.00458904773699208 | 0.0247967255486329 |
| SCN5A | 24.4521988196479 | 0.672825887461819 | 0.164236359093377 | 4.09669266401164 | 0.0000419094706589227 | 0.000663920662139125 |
| DNAH2 | 191.904085710972 | -0.611124768779107 | 0.137456126408072 | -4.44596239359194 | 8.74992554007499E-06 | 0.000191376723290509 |
| TREML3P | 2.5421530624571 | -0.748555205865324 | 0.19670255682872 | -3.80551843317996 | 0.000141507474446214 | 0.00172459655992358 |
| KCNJ12 | 65.3520557565707 | 0.807991336834149 | 0.200677462985428 | 4.02631827617245 | 0.0000566569883679669 | 0.000838002213622975 |
| EFNA5 | 137.220586647873 | -0.591565589474416 | 0.153772493252034 | -3.84701826031289 | 0.000119564061385517 | 0.00151759877594561 |
| MAP7D2 | 342.837005409425 | 0.789231315571183 | 0.224220568116583 | 3.51988812712679 | 0.000431728856348272 | 0.00411304719150773 |
| IRS3P | 1.82860643819046 | 0.610933291423398 | 0.226136790172454 | 2.70160945929008 | 0.00690047625806486 | 0.0336884273025117 |
| LRRC19 | 649.245160060955 | 0.620269145547327 | 0.132205443609455 | 4.69170654863236 | 2.70935553721536E-06 | 0.0000751256212940402 |
| PTP4A3 | 2160.8212542711 | 0.690340746544133 | 0.116790130677142 | 5.9109510584634 | 3.40138091799174E-09 | 3.24071169970028E-07 |
| FOXD4L1 | 3.11880508436221 | -0.692396769457973 | 0.16639392666128 | -4.16119015489941 | 0.000031659332781418 | 0.000533405422898604 |
| GAST | 2.72309526408998 | -0.850805178346175 | 0.257277518528795 | -3.30695500800608 | 0.00094316039179408 | 0.00747598241819806 |
| NUTM1 | 2.75909944546606 | -0.596364322124818 | 0.199543516088992 | -2.98864294773103 | 0.00280219384900492 | 0.0170569651799094 |
| BEX5 | 65.7806685488861 | -0.532886457973611 | 0.186668766189274 | -2.85471677373861 | 0.00430752241004331 | 0.0236077745047627 |
| DDX53 | 1.25799924677554 | -2.51787172455429 | 0.726469383959336 | -3.46590204645876 | 0.000528455770581777 | 0.00481149411374784 |
| TRARG1 | 3.99461802211815 | -1.05707066364227 | 0.340073427931628 | -3.1083600682109 | 0.00188128710838392 | 0.0126156495941195 |
| SDR42E1 | 351.866562521509 | 0.518383301304816 | 0.123336889323831 | 4.20298666641219 | 0.0000263416110709848 | 0.000465600506044331 |
| LCN12 | 146.072809844579 | 0.728596117969835 | 0.13458001612056 | 5.41385072592903 | 6.16835952189837E-08 | 3.58128956825218E-06 |
| MUC6 | 221.673103222637 | -1.81750752577796 | 0.269037634616401 | -6.75558840817716 | 1.42256731558132E-11 | 0.0000000034980511888 |
| USP18 | 201.96276929352 | -0.570103131878236 | 0.0991346706960549 | -5.75079462992481 | 8.8824961584723E-09 | 7.20344418500572E-07 |
| IFNE | 3.84610189914134 | -1.77393432599517 | 0.292413281768317 | -6.06653129867294 | 1.30702265202003E-09 | 1.53366496592469E-07 |
| SLC2A3P2 | 1.77019358617656 | 0.517946127703358 | 0.197970294971614 | 2.61628204260454 | 0.00888930965633385 | 0.0406726355899735 |
| ANO9 | 4747.30842302736 | 0.584316675207436 | 0.106891774932268 | 5.46643252558663 | 4.59183100518891E-08 | 2.80040132512382E-06 |
| MYADML2 | 87.1483448794776 | 0.628072350333856 | 0.166965227539979 | 3.76169553138523 | 0.000168765411596062 | 0.00198029926126158 |
| PRSS57 | 1.36554836667777 | -0.720817130750953 | 0.262099424236199 | -2.75016678442364 | 0.00595649383648467 | 0.0301974017268217 |
| NOTUM | 2852.28081703427 | 0.895306193268295 | 0.255202178327671 | 3.5082231630435 | 0.000451110463968154 | 0.00425438131303525 |
| GALNT17 | 44.8364328388571 | 0.568948868891055 | 0.135751687872531 | 4.19109977789218 | 0.0000277605535024595 | 0.00048428191456925 |
| CD24P4 | 15.8826707531015 | 0.524330766205511 | 0.130275282495919 | 4.02479085947817 | 0.0000570259725468219 | 0.000841598664567881 |
| RGPD2 | 3.68597865881168 | -0.556656512179551 | 0.213352964708919 | -2.60908730721885 | 0.00907840766578469 | 0.0412680766691921 |
| MAPK11 | 148.532128866438 | -0.562122385187979 | 0.0995171149851309 | -5.64849961006171 | 1.61854219156916E-08 | 0.0000012109012968782 |
| LINC00158 | 0.872846410682692 | -0.935845835881147 | 0.25965603941142 | -3.60417511567415 | 0.00031314587977687 | 0.00321431077700985 |
| KRT6B | 444.166259628325 | -0.981505091602575 | 0.219701991604836 | -4.46743829872943 | 7.91618331017514E-06 | 0.000176960563007271 |
| ROR1 | 87.4020904836775 | -0.718925230463644 | 0.137756321754538 | -5.21881842740159 | 1.80068183496137E-07 | 8.42215411535359E-06 |
| L3MBTL1 | 236.091555300411 | 0.699895245318678 | 0.121059982796682 | 5.78139224168021 | 7.40849292774951E-09 | 6.19813316382815E-07 |
| FAM131C | 19.2326479652861 | -0.623909762901503 | 0.149992897948957 | -4.15959536373396 | 0.0000318811890591572 | 0.000535495090163905 |
| AHNAK2 | 1071.04717885156 | -0.848162510485434 | 0.15106656001479 | -5.61449542772665 | 1.97136641582665E-08 | 0.0000014394418270322 |
| PDIA2 | 68.3060847683138 | 0.607771084640608 | 0.171725485940999 | 3.53920142552065 | 0.000401339491086619 | 0.00389596379126813 |
| KRT79 | 1.61704136860888 | -1.21717222577852 | 0.256283879121956 | -4.74931248094348 | 2.04109355356479E-06 | 0.0000603521225626116 |
| MORN5 | 3.85885481315347 | 0.820772743006059 | 0.310368020501652 | 2.64451454012379 | 0.00818081918395159 | 0.0381947733283769 |
| ADARB2 | 15.9614290212189 | 0.867347145751848 | 0.157628599218202 | 5.50247321903303 | 3.74500127992941E-08 | 0.0000024131085318574 |
| SRL | 20.8579940081961 | -0.704432303654811 | 0.135187765266721 | -5.21076964520412 | 1.88058834941679E-07 | 8.69856647042824E-06 |
| KCNQ5 | 6.48742788822218 | -0.629755482314206 | 0.188715338829044 | -3.33706568963479 | 0.00084667934298172 | 0.00693640179449192 |
| CCDC190 | 3.27245852419046 | -1.19005422885607 | 0.359476632562037 | -3.31051901864774 | 0.000931231237136657 | 0.0074130528522552 |
| ZNF829 | 25.2027396963476 | 0.596421169735555 | 0.126341795112634 | 4.72069570646708 | 2.35039338822882E-06 | 0.0000672542954242623 |
| C16orf54 | 78.4987826171893 | -0.73246400564859 | 0.137425750221073 | -5.32988908170628 | 9.82727675080095E-08 | 5.17549274173678E-06 |
| KLHL34 | 18.4104755323329 | 0.89711897785315 | 0.241011988208331 | 3.72230022465803 | 0.000197416091505463 | 0.00223790960906729 |
| KRTAP5-5 | 10.1845413852997 | 0.593716983722341 | 0.193465996802679 | 3.06884410456835 | 0.00214888694846483 | 0.0139053942203098 |
| NKAIN3 | 1.90993909969797 | 0.76142316752502 | 0.233599360794047 | 3.25952590339632 | 0.00111598589178781 | 0.00850907437144729 |
| C7orf61 | 5.99904224047541 | -0.563435751699734 | 0.129904818855378 | -4.33729677362471 | 0.0000144245801791528 | 0.000288336407860865 |
| SHOX | 3.50393385656151 | 0.88510428635964 | 0.190739720547347 | 4.64037738872503 | 3.47773473188204E-06 | 0.0000912891718238611 |
| GRK1 | 15.6848601744093 | 1.04993903963821 | 0.212762398412112 | 4.93479603291799 | 8.02345640509033E-07 | 0.0000284841219850351 |
| SLITRK2 | 2.6918008454637 | -1.01456555132608 | 0.219494079496042 | -4.62229119644373 | 3.79524749781872E-06 | 0.0000979326132886216 |
| ATP4B | 1.39653045666847 | 0.633791166573512 | 0.245351345488785 | 2.58319825110754 | 0.0097889054895349 | 0.0435783516215423 |
| CYP4F2 | 297.320882838906 | 0.600822433250831 | 0.186061144721721 | 3.2291665954729 | 0.00124151548962847 | 0.00918759924854064 |
| LILRP1 | 0.637561497377366 | -0.762511325126656 | 0.247434875042499 | -3.08166472085104 | 0.00205846549859518 | 0.0134741051485653 |
| FFAR4 | 571.685960560826 | -0.518050885131022 | 0.162483595605578 | -3.18832730898305 | 0.00143098456556389 | 0.0102319832887722 |
| CD300E | 62.7861832229596 | -0.545628679920418 | 0.157644325750459 | -3.46113745181106 | 0.000537898095804155 | 0.00488284150919722 |
| FCAR | 16.5039128314831 | -0.540365117564049 | 0.191785181525741 | -2.81755406369351 | 0.00483909670474897 | 0.0258224145524518 |
| KLK12 | 216.340857080617 | -0.739296624016732 | 0.231592042402303 | -3.19223672950077 | 0.00141175576378573 | 0.0101161209026189 |
| ANKRD20A5P | 47.9441125218504 | -0.548178347253282 | 0.134950115394476 | -4.06208135243819 | 0.0000486371203686308 | 0.000744519462606814 |
| PRG2 | 1.40933255396873 | -0.659125275843238 | 0.213795960423156 | -3.0829641240118 | 0.00204949861243072 | 0.0134324217746822 |
| KRT16 | 66.8841407064084 | -1.19643827976188 | 0.225663010816547 | -5.3018803366695 | 1.14615963760324E-07 | 5.90598930673767E-06 |
| KRT14 | 13.1984090946123 | -1.10883317502688 | 0.326587936056318 | -3.39520555601805 | 0.000685770926298174 | 0.00589551447744564 |
| MAPT | 55.1198837943993 | -0.82519354763717 | 0.167019757336543 | -4.94069420765839 | 7.78449126348772E-07 | 0.0000278426691800595 |
| C1QL4 | 2.8115732264432 | -0.851550289435137 | 0.207782811848843 | -4.09827108343599 | 0.0000416247621236685 | 0.000660348811641234 |
| SERPINA11 | 5.5973000783666 | -0.89665073655298 | 0.303259639370073 | -2.9567097633417 | 0.00310940651665926 | 0.0184489672244172 |
| SAMD7 | 0.611434638873061 | -1.04262138078791 | 0.313221894558354 | -3.32869891569367 | 0.000872526731375076 | 0.00707815136000215 |
| TMPRSS6 | 41.0396730065621 | 0.516858407476145 | 0.150571621494276 | 3.43264157181034 | 0.00059773172683637 | 0.00529098581494491 |
| CCK | 11.7638021077495 | -2.55217389825667 | 0.324195053534698 | -7.87234064934157 | 3.4806686627077E-15 | 3.23334781717419E-12 |
| AKR1C1 | 121.175161689569 | -0.621213470210894 | 0.134551203048258 | -4.61692988347409 | 3.89458783441999E-06 | 0.0000999561061847071 |
| VSTM2B | 5.04445150220409 | 1.78211739326389 | 0.420821253592357 | 4.23485595855908 | 0.0000228698252350778 | 0.000413859683718328 |
| AC092118.1 | 1.41138192471721 | -0.899405586301308 | 0.235853323785333 | -3.81341069045065 | 0.000137062069098222 | 0.00168376493160235 |
| MT1X | 568.673750352324 | -0.5651157250427 | 0.102589810848187 | -5.50849758246421 | 3.61909210861094E-08 | 2.36693558416047E-06 |
| CIDEC | 78.4633851168692 | -0.665586130351824 | 0.162123814120167 | -4.1054186515652 | 0.0000403583293219254 | 0.000643923305908315 |
| AGMO | 62.0964050613365 | -0.531621578602643 | 0.175141948084229 | -3.0353755020869 | 0.00240236382682214 | 0.015158462471054 |
| CYP26C1 | 1.63830740978315 | 0.64402201945708 | 0.223396579104442 | 2.88286428574176 | 0.00394077183170856 | 0.0220464192069062 |
| NANOS3 | 73.300706513567 | 0.651887441090482 | 0.180931135749718 | 3.60295887376863 | 0.000314615321901309 | 0.00322345759651458 |
| NHLRC1 | 80.4979947998146 | 0.527468862559891 | 0.133500974836421 | 3.95104877103857 | 0.0000778094643909161 | 0.00107702984609314 |
| PERM1 | 52.8671629491752 | -0.505069943800212 | 0.104637309425705 | -4.8268628711141 | 1.38700554376733E-06 | 0.0000441334342480182 |
| LIN28B | 2.19789179719731 | -2.82448377934102 | 0.585777924064493 | -4.82176549048312 | 1.42293173061842E-06 | 0.0000450508540322151 |
| TMEM72 | 34.5175561896676 | 0.730865121862135 | 0.173453375664175 | 4.21361140458385 | 0.0000251319536388445 | 0.000447292599036849 |
| H1-2 | 501.104113217984 | -0.746123745898527 | 0.113590516551429 | -6.56853906955089 | 5.0811301660721E-11 | 1.01754003252517E-08 |
| TTC24 | 5.06823503331226 | -1.02690678828371 | 0.201795214838542 | -5.08885599247406 | 3.60230028383065E-07 | 0.0000148022853383231 |
| FYB2 | 8.1575873858282 | -0.665602763246476 | 0.217590423198818 | -3.05897085662772 | 0.00222098754659623 | 0.0142615717229783 |
| LRRC74B | 1.61261602188474 | -0.744138730342628 | 0.285986656753271 | -2.60200506831554 | 0.009268048254661 | 0.0418895322742079 |
| DNER | 16.3710074612188 | -0.594344849405951 | 0.183840963039948 | -3.23292937318221 | 0.00122527850902911 | 0.00909763852108159 |
| RTP5 | 1.68219382273623 | -1.16038635846738 | 0.252301374125399 | -4.59920744581695 | 4.24101308993152E-06 | 0.000107039969625275 |
| ARL4C | 1184.59008690447 | -0.590388413844955 | 0.110651215598245 | -5.335580008344 | 9.52396212315499E-08 | 5.06066658052275E-06 |
| WNT7B | 77.1803540201876 | -2.39195983063851 | 0.207451483601116 | -11.5302131810141 | 9.29133557425923E-31 | 1.55360422137189E-26 |
| FAM25A | 1.11406223539595 | -2.02093835010496 | 0.380389413168162 | -5.31281439531428 | 1.07944968471123E-07 | 5.59673741955239E-06 |
| MAPK12 | 203.121100410474 | -0.771591004542639 | 0.124084709930936 | -6.21826013029404 | 5.02697683257709E-10 | 6.6976955870535E-08 |
| TMEM215 | 1.73476452891743 | 0.629926843496959 | 0.231505837226654 | 2.72099766918721 | 0.00650852204344136 | 0.032214012095076 |
| SMTNL2 | 62.6705391321133 | 0.781809024688914 | 0.182580147120909 | 4.28200457178503 | 0.0000185217175804032 | 0.000349550383365601 |
| ZNF793 | 61.1867960247933 | 0.838352236132565 | 0.164744913741103 | 5.08878979687371 | 3.60355775638584E-07 | 0.0000148022853383231 |
| PLA2G2A | 8256.4300335854 | -0.640488947784372 | 0.217414690820036 | -2.94593224298046 | 0.00321982923856823 | 0.0189606496559604 |
| IGFL1 | 14.834565629139 | -1.16788985915723 | 0.281492047857548 | -4.1489266501349 | 0.0000334037812445408 | 0.000555213346113287 |
| SLC38A3 | 40.9855254063295 | 1.07712165165233 | 0.236818718393746 | 4.54829609313847 | 5.40820090074285E-06 | 0.000130585598933316 |
| C10orf99 | 2322.7141273576 | 0.595439257200759 | 0.166136552389655 | 3.5840352326815 | 0.000338326418032767 | 0.00341306548170492 |
| PDCD1 | 64.885958823565 | -0.586826588821595 | 0.135209685909881 | -4.34012241706355 | 0.000014240336216507 | 0.000285678058639729 |
| SERPINA5 | 43.6303882819359 | -0.679601271982053 | 0.153229022972555 | -4.43519940803759 | 9.19871019828038E-06 | 0.00019898012060213 |
| LCTL | 8.58488790864122 | -0.538798835947623 | 0.111528420268406 | -4.83104516903354 | 1.35818184725029E-06 | 0.0000434030602905369 |
| HBA2 | 118.312647751552 | -0.602725472054912 | 0.160450980715601 | -3.75644617045527 | 0.000172343320457152 | 0.00201029135777051 |
| FBLL1 | 12.5617484191449 | 0.787469493329651 | 0.200844269875399 | 3.92079641514387 | 0.0000882568021255148 | 0.00119493278408157 |
| FAM72B | 26.222302310198 | -0.68677427485069 | 0.159211931132172 | -4.31358548299094 | 0.0000160627990507999 | 0.000312309375498168 |
| HMX3 | 8.25310659654269 | -1.8233268079039 | 0.308060130736809 | -5.91873672046727 | 3.24423810352192E-09 | 3.11763823729828E-07 |
| IGFL3 | 8.01706571303425 | -1.28898506520387 | 0.261048683783302 | -4.93771907417031 | 7.90415920032132E-07 | 0.0000282404799120882 |
| VWC2 | 5.46997296436861 | 0.657544705858781 | 0.186423851573818 | 3.52714902255098 | 0.000420060266702021 | 0.00403552296439213 |
| SPRED3 | 44.7896151590326 | -0.623319987707208 | 0.101683082736826 | -6.13002645996162 | 8.78644587817806E-10 | 1.08028059947805E-07 |
| HMX2 | 2.69953332184182 | -0.748973884152273 | 0.286805108166506 | -2.61143843964401 | 0.00901622189386791 | 0.0410902824440898 |
| BEND4 | 5.18455735492304 | -0.607373905346792 | 0.171909465736032 | -3.53310332706995 | 0.000410711916679235 | 0.00396590826026266 |
| KLRG2 | 39.9510082860493 | 0.985586435976345 | 0.268950202305039 | 3.66456848713767 | 0.000247755938979162 | 0.00268462949025879 |
| SBSN | 7.29919617513568 | -0.794374994621426 | 0.206012910873408 | -3.85594762606678 | 0.000115282170008288 | 0.00147711353617516 |
| KIR2DL4 | 11.7655288970213 | -0.871974827344856 | 0.187589453245056 | -4.64831477602186 | 0.0000033465794339644 | 0.0000888930178162332 |
| CGB5 | 2.34375678981666 | -1.41822397376473 | 0.428653926707648 | -3.3085523901709 | 0.000937796370910674 | 0.00744758637758128 |
| IL1RAPL2 | 1.23391127668682 | -1.78082597772353 | 0.412770682297895 | -4.31432282886388 | 0.0000160092862348287 | 0.000311994493161505 |
| DPY19L2P1 | 1.66525932976998 | -1.12883981270142 | 0.228059539350402 | -4.94975924233107 | 7.43053420760967E-07 | 0.0000268349810983674 |
| LINC00943 | 4.21040632582312 | -0.902781474728 | 0.196813330430908 | -4.58699353723361 | 4.49674595539793E-06 | 0.000112127636656059 |
| GJB5 | 162.010399259624 | -1.04706967029338 | 0.240517437530555 | -4.35340439780114 | 0.0000134039562985908 | 0.000271669761537862 |
| AC073349.1 | 2.459557477621 | 0.618123044055403 | 0.195032223180632 | 3.16933804052943 | 0.00152786582834793 | 0.0107806234089117 |
| S100A14 | 5740.55521087461 | -0.615967296140422 | 0.102310042392588 | -6.02059467219072 | 1.73777427793055E-09 | 1.89298525741217E-07 |
| NCR1 | 4.89936296125163 | -0.981892568010872 | 0.184568860129692 | -5.31992540518981 | 0.0000001038098066629 | 5.42438680378219E-06 |
| RASSF10 | 258.910304037979 | 0.765265604563407 | 0.152884700289052 | 5.00550809280821 | 5.57148719096318E-07 | 0.00002119700507852 |
| GJB4 | 145.550541309186 | -0.627261608107658 | 0.153112212303751 | -4.09674446387899 | 0.0000419000979700562 | 0.000663920662139125 |
| PAX5 | 85.7340839914882 | -0.972329028102024 | 0.204410479714472 | -4.75674744983824 | 1.96737043713012E-06 | 0.0000585345214933323 |
| IFITM3P3 | 3.38266738748522 | 0.537621492347411 | 0.165644884005149 | 3.24562690587352 | 0.00117192317458668 | 0.00881748535400644 |
| SERPINA3 | 3.56309033455407 | -0.908149679374387 | 0.231766669848966 | -3.91837911795598 | 0.0000891464169824069 | 0.00120600100191167 |
| COLCA1 | 304.243622613079 | -0.754662775555131 | 0.18749772302469 | -4.02491701435625 | 0.0000569954107831963 | 0.000841518996649735 |
| ZNF681 | 170.135829318395 | 0.508857604881772 | 0.145893181516135 | 3.48787790898571 | 0.00048687034420916 | 0.00451397783505482 |
| SULT1C3 | 13.299424514011 | -1.36894614445646 | 0.248911217154804 | -5.49973665351162 | 3.80358896000883E-08 | 0.0000024321151434152 |
| SFTA2 | 108.162979259862 | -0.760157844307539 | 0.203173157862633 | -3.74142850514479 | 0.000182977233212308 | 0.0021122280404163 |
| ZNF471 | 38.8665242903512 | 0.5515814691718 | 0.141588119556182 | 3.89567621139945 | 0.0000979251370828602 | 0.00129599295140187 |
| CPNE4 | 8.74231337460243 | -0.737891483726589 | 0.256152076624014 | -2.88067734391115 | 0.00396821639283752 | 0.0221803597876103 |
| ELAVL3 | 2.30637962230509 | -0.881936757451021 | 0.222935702826645 | -3.95601398191843 | 0.0000762107024345918 | 0.0010575262700488 |
| NUDT11 | 21.1686210050732 | -0.503967408554866 | 0.13485736868683 | -3.73704020375182 | 0.000186199160734302 | 0.00213908359095724 |
| NOXO1 | 2.25826644780674 | 0.836333576622667 | 0.270818064021576 | 3.0881750065093 | 0.00201389834954475 | 0.0132445995291004 |
| PRTN3 | 0.649184585611825 | -1.53854940723769 | 0.344856611864034 | -4.46141774380214 | 0.000008141919824619 | 0.000180798195733671 |
| C20orf204 | 43.0655810812204 | 0.734567971971381 | 0.159978717896472 | 4.59166057604453 | 4.39733117696111E-06 | 0.000109989191637946 |
| ZNF569 | 48.1840636055484 | 0.509826880905084 | 0.110477556747679 | 4.61475521285724 | 0.0000039355889470816 | 0.000100853613462301 |
| ESRRG | 41.0085530447114 | 0.815001123125379 | 0.179125191825138 | 4.54989672207014 | 5.36722521422032E-06 | 0.000129783619388255 |
| SPTSSB | 44.9664415712785 | -0.594730889620245 | 0.227091061747268 | -2.61890928266533 | 0.00882114024354435 | 0.0404221046728403 |
| MME | 667.867285049353 | 0.875075754065598 | 0.170742051159641 | 5.12513319432609 | 2.97327480128308E-07 | 0.0000127151222384282 |
| LAMA2 | 402.383562244527 | -0.555171536222232 | 0.132355785902376 | -4.19453922952577 | 0.0000273426829371894 | 0.00047896613994909 |
| MMP1 | 4014.91807303235 | -0.600520937963075 | 0.178006150591255 | -3.37359656376154 | 0.000741930186160944 | 0.00627031318817141 |
| LINC00173 | 16.2655343229901 | -0.782613552446495 | 0.158913444351798 | -4.92477874127481 | 8.44558612081994E-07 | 0.0000297929631911878 |
| ALKAL1 | 59.9585335207594 | 0.796129315703716 | 0.151940184704472 | 5.23975482359858 | 1.6079007646823E-07 | 7.73689458597199E-06 |
| DAPK1 | 681.284239444819 | -0.605547622897204 | 0.131367586792216 | -4.60956646676474 | 4.03509517232011E-06 | 0.000103008895231091 |
| HLA-DQA1 | 2040.53588499221 | -0.502128396482363 | 0.138104411410702 | -3.63586066044704 | 0.000277054067630159 | 0.00293231530237865 |
| CHRNG | 4.06063370124361 | 0.5078281896688 | 0.162635872200897 | 3.12248572714329 | 0.00179330800587867 | 0.0121671345775196 |
| ZFP28 | 77.798003677749 | 0.665036160311948 | 0.142093621825888 | 4.68026750086529 | 2.86500851347001E-06 | 0.0000784055766836858 |
| TEX43 | 13.9811263797225 | 1.10535586120098 | 0.175456147349376 | 6.29989816771676 | 2.97841254268027E-10 | 4.34952280577788E-08 |
| FAM163B | 6.71819861994822 | 0.606905990371529 | 0.195039629031681 | 3.11170603320286 | 0.00186009624061219 | 0.0125111300238441 |
| DTHD1 | 7.57323938354785 | -0.954375890374165 | 0.192373141008995 | -4.96106621417352 | 0.0000007010728654032 | 0.0000257075425052784 |
| NPSR1-AS1 | 25.5346052594847 | -0.939416570646799 | 0.206696905080432 | -4.5448990650404 | 5.49615849820733E-06 | 0.000132518047943078 |
| ZNF682 | 50.6529751284892 | 0.552386830522277 | 0.141682654253649 | 3.89876116756933 | 0.0000966860805300888 | 0.00128512555846074 |
| MAGEA6 | 109.488769835399 | -4.04273369218442 | 0.540198016981148 | -7.48379957923005 | 7.22040421260257E-14 | 4.47156958662694E-11 |
| PIWIL2 | 196.444275313182 | 0.515335435628801 | 0.129487056173846 | 3.97982200581443 | 0.0000689668861571153 | 0.000978943381522177 |
| SERPINA1 | 12950.0451474059 | -0.564841765036249 | 0.165777481748023 | -3.40722852754388 | 0.000656261551319623 | 0.00567978747392102 |
| RAMP2-AS1 | 28.3759620986977 | 0.676093385128136 | 0.149843485677068 | 4.51199718208108 | 6.42200416497228E-06 | 0.000149453488716077 |
| FITM2 | 1219.13237990754 | 0.700668324401668 | 0.084507538484672 | 8.29119315229795 | 1.12118045205633E-16 | 1.78545317512704E-13 |
| OGDHL | 99.460340858056 | 0.573533857804169 | 0.21290596260166 | 2.69383652198239 | 0.00706348000497302 | 0.0342690988432189 |
| PRAMENP | 5.75772101950244 | -0.983942097077646 | 0.269298596816833 | -3.65372158900214 | 0.000258466525102219 | 0.00277751848729705 |
| COL4A6 | 32.1470108298617 | 0.513502199572155 | 0.166833258264082 | 3.07793664713618 | 0.00208439222601959 | 0.0135858651693693 |
| DMBX1 | 29.5745334204006 | -1.18233744446715 | 0.196271375794767 | -6.02399325769985 | 1.70165457602009E-09 | 1.85969713500862E-07 |
| SERPINB2 | 39.1531990705237 | -1.59696440534356 | 0.269204338367249 | -5.93216444812631 | 2.98966953686731E-09 | 2.9406037838799E-07 |
| DPP4 | 1413.04236952213 | -0.798603769403041 | 0.139010389865753 | -5.74492144201795 | 9.19636716708293E-09 | 7.42746029117511E-07 |
| KLHL14 | 12.7383079901032 | -1.02628857837491 | 0.209986195600894 | -4.88740974347429 | 1.02171309428484E-06 | 0.0000346181654499226 |
| HOXC6 | 70.6333619910476 | -1.6042909326555 | 0.225418659674546 | -7.11693936505407 | 1.10350002136464E-12 | 4.44617442343088E-10 |
| ZNF461 | 86.2842368379457 | 0.760305701302545 | 0.102526326292876 | 7.41571193266656 | 1.20973186355047E-13 | 6.8529862092083E-11 |
| ADAMTSL2 | 326.460407822289 | 0.579956918351168 | 0.121418959149647 | 4.77649390517656 | 1.78377957227682E-06 | 0.00005421380598518 |
| ZNF790 | 122.554781058264 | 0.611804307039664 | 0.130821659628668 | 4.67662853977122 | 0.0000029162982681294 | 0.0000796137523941088 |
| SLC22A12 | 1.37485183958485 | 1.39736269761658 | 0.33554266081907 | 4.16448595300988 | 0.0000312054837461154 | 0.000528391791107642 |
| HRNR | 4.188094546405 | 0.714903191994974 | 0.205914694819134 | 3.47184154400885 | 0.000516901340501446 | 0.0047281768679019 |
| TUBA3C | 0.676520372008908 | -1.35981797565959 | 0.47121678030454 | -2.88575881101 | 0.00390471266743018 | 0.0219133077738211 |
| UCKL1 | 2249.97530662233 | 0.556064436242524 | 0.0784846700420641 | 7.08500699492652 | 1.39037278958074E-12 | 5.1095436075999E-10 |
| CARD11 | 575.764002544784 | 0.572376635559689 | 0.164153775726384 | 3.48683198438118 | 0.000488778321090542 | 0.00452456266986838 |
| ZNF813 | 141.637924443654 | 0.569764606915994 | 0.161486307798822 | 3.5282533527598 | 0.000418311578901285 | 0.00402566210694008 |
| HOXC4 | 32.8817914493138 | -1.20640853568999 | 0.184593116836132 | -6.53550119510113 | 6.33968758904648E-11 | 1.21845880662582E-08 |
| ZNF43 | 189.398344568012 | 0.844281082195405 | 0.157220716210579 | 5.37003712070989 | 7.87204367407149E-08 | 4.30862331502944E-06 |
| SH2D1B | 12.1287470323338 | -0.542399397892178 | 0.141558345472144 | -3.83163137491538 | 0.000127296370116237 | 0.00158904263136514 |
| ARC | 31.2976160001757 | -1.17106776034219 | 0.183055140644255 | -6.39734976150171 | 1.58096907608888E-10 | 2.68379532195758E-08 |
| MMP17 | 117.413958804759 | 0.543296148449153 | 0.154871389589454 | 3.50804722479321 | 0.000451408910488171 | 0.00425599571033139 |
| TAT | 4.91027526440641 | -0.519193959885725 | 0.132549467147446 | -3.91698262587642 | 0.0000896642081962275 | 0.00121104622394921 |
| MAGEA1 | 5.21262917047036 | -2.51230869290749 | 0.602954592734672 | -4.16666316697752 | 0.0000309090680669907 | 0.000525447871910951 |
| F5 | 269.714116564852 | -1.53724287791992 | 0.188804260549259 | -8.14199252415096 | 3.88825265727528E-16 | 5.2457841660664E-13 |
| COLGALT2 | 82.508757392131 | -0.825330351684057 | 0.210333785028686 | -3.92390766690903 | 0.0000871241384391818 | 0.00118246973931945 |
| APCDD1L | 17.6542356835282 | -1.00654481807182 | 0.18386081152462 | -5.4744935025865 | 4.3876473872623E-08 | 2.69232484265735E-06 |
| PAX9 | 80.5259375469025 | -0.512371769861419 | 0.155852001096434 | -3.28755335996223 | 0.00101062034855479 | 0.00786163426293773 |
| GJC2 | 139.115393674983 | 0.596788156578797 | 0.172797530513013 | 3.45368452203577 | 0.000552983853878649 | 0.00498863216997464 |
| CES1 | 651.72624039158 | 1.05828710918309 | 0.207872244787134 | 5.09104575392833 | 3.56094079207563E-07 | 0.0000146837215744258 |
| PNMA5 | 8.30749736794202 | -1.05128933014731 | 0.322538802077343 | -3.25941971439213 | 0.00111640373363281 | 0.00851026142202622 |
| POU3F3 | 4.56523028427304 | -0.648098882156239 | 0.237796278682819 | -2.72543744479995 | 0.00642163437630987 | 0.0318971428880137 |
| CSAG1 | 18.1649886430586 | -3.63191801136903 | 0.447184997473704 | -8.12173492377191 | 4.59566215091167E-16 | 5.69215309817734E-13 |
| MAGEE1 | 45.3614458124692 | 0.796461611547126 | 0.141117764621978 | 5.64395002769967 | 1.66192330234806E-08 | 0.0000012378182422522 |
| SOWAHA | 540.628956126817 | 0.575527097888948 | 0.103359253478246 | 5.56822034332975 | 2.57354258476803E-08 | 1.78650176558209E-06 |
| DMD | 361.000916387698 | 0.57285584312844 | 0.127668608795562 | 4.48705322735807 | 7.22150377860371E-06 | 0.000164175070947699 |
| SNORA60 | 7.91637572141548 | 0.517504329040693 | 0.133518204185356 | 3.87590839914435 | 0.000106227644168654 | 0.00137799258195815 |
| RNA5SP183 | 1.73166600007925 | 1.5576744670425 | 0.471992129844857 | 3.30021279709581 | 0.000966115426785674 | 0.00761564561833915 |
| RNA5SP191 | 1.44480903248405 | 3.75325899174486 | 1.16263083548448 | 3.22824655702584 | 0.00124551571763629 | 0.00920905076922239 |
| AL163636.1 | 0.639610393663845 | 0.586673551622744 | 0.225672807282032 | 2.59966434896853 | 0.00933149830241732 | 0.0420973811060945 |
| RNA5SP226 | 1.46217013317784 | 2.32165653629829 | 0.650802389522217 | 3.56737555620028 | 0.000360574524406645 | 0.00359092711292645 |
| RNA5SP452 | 0.588607436910154 | 1.80945421886677 | 0.49938494834069 | 3.62336555172328 | 0.000290794389833282 | 0.00304183484041433 |
| RNA5SP145 | 1.33585237960303 | 2.47918581287181 | 0.570171032711212 | 4.34814410174973 | 0.0000137294388644493 | 0.000276632673421779 |
| RNU6-403P | 2.00458390418506 | -0.967029003804951 | 0.219662261051641 | -4.40234475951975 | 0.0000107087210351237 | 0.000226946165308369 |
| SNORA73B | 68.836761853814 | 1.71243195051146 | 0.184976338271548 | 9.25757297669927 | 2.09131674291642E-20 | 6.35798313787371E-17 |
| RNU5D-1 | 0.520950896094396 | 1.25241206665093 | 0.364280046583928 | 3.43804739896008 | 0.000585925126974023 | 0.00521252407447541 |
| RNU1-22P | 1.11791273081693 | 0.966525898742249 | 0.256598213472615 | 3.7666898988188 | 0.000165426265951601 | 0.00194658169808355 |
| RNA5SP215 | 0.811058099680251 | 2.37716965111768 | 0.87024982464628 | 2.73159451894622 | 0.00630286589497956 | 0.0314685098232839 |
| RNA5-8SP2 | 1.72941124393136 | 1.64878242825826 | 0.529581603736041 | 3.11336801850099 | 0.00184965217003916 | 0.0124488413884794 |
| RNA5SP429 | 0.628677900622978 | 1.22127442286767 | 0.434607315858976 | 2.81006411604898 | 0.00495316314707979 | 0.0262884751570612 |
| RNA5SP161 | 0.71271966408095 | 2.36842908391496 | 0.76367661245995 | 3.10135081430057 | 0.00192639915053197 | 0.0128306393929675 |
| RNU4-1 | 14.122022380415 | 1.39398844267312 | 0.281804758583772 | 4.94664621590741 | 7.55030190799433E-07 | 0.000027208749612839 |
| SNORA74A | 1.41172082338636 | 0.953077189433624 | 0.226097411178496 | 4.21533879784763 | 0.0000249403489019361 | 0.000444118822139801 |
| RNA5SP242 | 0.75773885494421 | 2.48537328382775 | 0.711286826670768 | 3.49419276533031 | 0.000475497554220495 | 0.00443188105023461 |
| RNY1 | 3.77595867404655 | 1.59947426033633 | 0.425401239351374 | 3.75991913604932 | 0.0001699682883378 | 0.0019895273008725 |
| RNVU1-3 | 2.31168170047362 | 0.527116912104364 | 0.162683908963925 | 3.24012937395825 | 0.00119475478148961 | 0.00894247748490948 |
| RNA5SP202 | 3.26546881993306 | 2.17521924741775 | 0.572250069909646 | 3.80116903744757 | 0.000144015008142294 | 0.00175160792008407 |
| RNA5S9 | 5.23123375402241 | 1.54096228154531 | 0.521784409209146 | 2.95325474343111 | 0.00314442359633417 | 0.0186149431596047 |
| RNU4-38P | 0.482752137347703 | 0.857168996439654 | 0.304602082496375 | 2.81406151072475 | 0.00489198667269725 | 0.0260216030393417 |
| AL117382.1 | 12.901623495779 | 0.929900668645337 | 0.178097992521796 | 5.22128663820585 | 1.77684265948137E-07 | 8.33396524801909E-06 |
| RNA5SP141 | 5.37658601912509 | 3.48186245155364 | 0.648293836990996 | 5.37080911907851 | 7.83841446532789E-08 | 4.29725010736877E-06 |
| AL732366.1 | 1.3496823876751 | 0.925779549466566 | 0.227884574004089 | 4.06249327543319 | 0.0000485513467613277 | 0.000743771936963958 |
| SNORA14A | 0.633640706641681 | 1.01520452986869 | 0.337876738554078 | 3.00465943353541 | 0.00265878371520362 | 0.0163627245130364 |
| RNU12-2P | 0.552800129528045 | 0.573811363119419 | 0.197475331934576 | 2.90573692166029 | 0.00366389355166568 | 0.020880696686231 |
| RN7SKP9 | 1.30596780171012 | 1.82000097717098 | 0.384878642781185 | 4.72876583647097 | 2.25888723416504E-06 | 0.0000651036616938508 |
| SNORD48 | 3.59128970413018 | 0.566195986021588 | 0.191587130313813 | 2.95529237842949 | 0.00312372866209394 | 0.0185153729028262 |
| AL732366.2 | 0.775161417590499 | 1.16498714414721 | 0.258478836944604 | 4.50708908287485 | 6.57230452937061E-06 | 0.00015192555106272 |
| SNORA23 | 2.07398085146175 | 0.544618271721359 | 0.192048947599778 | 2.83583054491047 | 0.00457066901283987 | 0.0247294078004939 |
| RNA5SP428 | 0.832986554657475 | 2.25386234694447 | 0.757155592619174 | 2.97674925591957 | 0.00291322164789785 | 0.0175633600773391 |
| RNA5SP355 | 2.34777807375971 | 4.25687671293935 | 0.774866233450273 | 5.49369236801637 | 3.9361591092964E-08 | 2.50729586539219E-06 |
| AL162581.1 | 244.698070351272 | 1.03076215790889 | 0.203212614519198 | 5.07233352785546 | 3.92966860452372E-07 | 0.0000158245737966223 |
| RN7SKP230 | 0.725465064707194 | 1.20812656100339 | 0.364554839222315 | 3.31397757215518 | 0.000919788846327919 | 0.00734703552411833 |
| RNU4-2 | 23.9256179472568 | 0.916360934948367 | 0.251624057300063 | 3.64178586412189 | 0.000270753249242804 | 0.00288085592146925 |
| TDRKH-AS1 | 23.7551868002617 | 0.525223861153027 | 0.0974080981474815 | 5.39199379868609 | 6.96801732928187E-08 | 3.93852372032203E-06 |
| AL139400.1 | 1.89522287504813 | 0.968315196644624 | 0.216833866428645 | 4.46570091929471 | 7.98070345246304E-06 | 0.000178164676139699 |
| AC144450.1 | 6.90958102465352 | -1.14156868837823 | 0.222428182841889 | -5.1323023629146 | 2.86219208104533E-07 | 0.00001231884524766 |
| STUM | 74.1652417106871 | 0.744316714986107 | 0.21400707623072 | 3.47800048529079 | 0.000505169010317572 | 0.00464244628827706 |
| SAMD5 | 1207.06592236994 | -0.583996763353087 | 0.145311260562507 | -4.01893673685307 | 0.0000584613638908721 | 0.000859369200544415 |
| FCGR3A | 978.74772359381 | -0.641534986165766 | 0.135108337191868 | -4.74830050831517 | 2.05133087581667E-06 | 0.0000605477556478915 |
| PRR9 | 15.6968969998027 | 1.1018488394691 | 0.407256997376539 | 2.70553691297379 | 0.00681940688114842 | 0.0334048051734826 |
| SPRR2E | 4.72190569035681 | -1.99752455652652 | 0.469697742990984 | -4.2527872154686 | 0.0000211126169435332 | 0.000389436368353908 |
| HSD3B1 | 20.0077619259239 | 0.611022283044899 | 0.224420617053918 | 2.72266555125859 | 0.00647575787106633 | 0.0320987817008022 |
| SPATA42 | 0.707582256877071 | 0.977145435943288 | 0.256028047352673 | 3.81655621736354 | 0.000135327252755613 | 0.00166942921556041 |
| CCDC160 | 2.29658694807578 | -0.845356338932872 | 0.315035458758025 | -2.68336885716151 | 0.00728845541696022 | 0.0351261746727171 |
| ZYG11A | 3.85350754099249 | -0.729448405897749 | 0.196419591541524 | -3.71372529681461 | 0.000204230480108275 | 0.00229652848546769 |
| LIPN | 1.78069779486923 | -0.683313538677957 | 0.259275929724507 | -2.63546847331339 | 0.00840212305939486 | 0.0389822141165764 |
| LRIT2 | 27.474261285501 | 0.628470494106641 | 0.190460809593406 | 3.29973654658034 | 0.000967756312773121 | 0.00761857500276806 |
| AL391421.1 | 2.68616487344376 | 0.656136656133503 | 0.236653467654833 | 2.77256303334841 | 0.0055616746959403 | 0.0287204331657868 |
| NEU4 | 302.341721445413 | 0.503021677478059 | 0.145032874743805 | 3.46832866939049 | 0.000523706349521747 | 0.00477735617586096 |
| HLA-DOA | 466.374674262034 | -0.524869280214656 | 0.145624246815237 | -3.60427120959185 | 0.000313030055098961 | 0.00321410841345393 |
| BTBD17 | 1.62886402500836 | -0.916630923116133 | 0.273428023642553 | -3.35236641403818 | 0.000801238981231913 | 0.00664889181398453 |
| C9orf129 | 0.790217690951857 | 0.962208696222158 | 0.259753811540461 | 3.70431021017868 | 0.000211966737727791 | 0.00236457432242527 |
| LINC02783 | 1.3453721200226 | -1.06244217666416 | 0.338309872934152 | -3.14044094382948 | 0.00168693715327238 | 0.0116222810629862 |
| AL662899.1 | 2.53833734874394 | 0.767617601873139 | 0.206718039793879 | 3.71335565410034 | 0.000204529141203491 | 0.00229834124332229 |
| LY6G6F | 7.44565050913821 | 0.998484325830517 | 0.199287085995576 | 5.0102811270605 | 5.4350580784899E-07 | 0.0000207724814012411 |
| PSORS1C2 | 13.6905765944961 | 0.613987223323139 | 0.194475653470768 | 3.15714184457248 | 0.00159323824282113 | 0.0111472343796048 |
| KRTAP5-10 | 2.86274541273502 | 0.708590254810961 | 0.181272445287902 | 3.90897940216758 | 0.0000926868654701582 | 0.00124283646954813 |
| LILRB3 | 52.0724684935704 | -0.60484025213606 | 0.113655969834425 | -5.32167604585307 | 1.02815545804014E-07 | 0.0000053808411311077 |
| AC010624.1 | 2.4063255102144 | -0.93367314202828 | 0.231587102293463 | -4.03162841445784 | 0.0000553917170348372 | 0.00082219698228097 |
| C9orf135 | 0.553637072046378 | 1.20246621546145 | 0.401690412593089 | 2.99351485065077 | 0.00275784086902967 | 0.0168606424757021 |
| MALRD1 | 102.012970874184 | -0.68690094874517 | 0.248106605884901 | -2.76857178508109 | 0.00563025809464097 | 0.0290114975185119 |
| IGFL2 | 115.248551052157 | -1.24863235924735 | 0.21374091792305 | -5.84180311088997 | 5.16388165254155E-09 | 4.58197467885577E-07 |
| KRT40 | 85.3232663620362 | 1.13487923932197 | 0.280285437041477 | 4.04901250418526 | 0.0000514341970026338 | 0.000777253690086796 |
| FAM221B | 11.621393889022 | 0.506656572941878 | 0.156814899147625 | 3.23092114139557 | 0.00123391978026653 | 0.00914352875951104 |
| FAM83A-AS1 | 2.72692510433924 | -0.714562425720499 | 0.2311894684614 | -3.09080872271569 | 0.00199612176243978 | 0.0131535574343865 |
| PCDHA5 | 4.51316549788588 | 0.775137937926033 | 0.254574880052444 | 3.04483277284212 | 0.00232809718413534 | 0.0147987504337301 |
| PCDHA4 | 40.1734549070809 | 0.954713863856536 | 0.256962710816997 | 3.71537901674949 | 0.000202899329378863 | 0.00228463278555149 |
| PRSS1 | 32.9329640989783 | -1.11924930383268 | 0.251833665311311 | -4.44439905383218 | 8.81378787098711E-06 | 0.000192521681242032 |
| AC134312.1 | 2.4954749278885 | -0.557034910411064 | 0.205922970098125 | -2.70506447214524 | 0.00682911337660457 | 0.0334425552116576 |
| LINC02397 | 3.40867826312911 | -0.921634756668339 | 0.211962128759517 | -4.34811049531392 | 0.0000137315423084789 | 0.000276632673421779 |
| SYCE1L | 59.9013297336795 | 0.734219978440078 | 0.140870703795865 | 5.21201327640156 | 1.86802176839591E-07 | 8.66440831882051E-06 |
| C2orf91 | 1.70781463262996 | -0.847584425727441 | 0.261197353976769 | -3.24499621769838 | 0.00117452183686299 | 0.00882745048348071 |
| LINC00654 | 197.029301518949 | 0.568541553135305 | 0.15411614827284 | 3.68904595337269 | 0.000225096563246097 | 0.00247784044373797 |
| MT1H | 105.399760382519 | -0.594885700377424 | 0.17067609202721 | -3.48546590979236 | 0.00049128081779778 | 0.00454102075975494 |
| MT1A | 55.5616395966864 | -0.709173078417853 | 0.196822868273736 | -3.60310305727053 | 0.000314440785264173 | 0.00322265667815031 |
| SAMD9 | 645.585071511895 | -0.839714332048096 | 0.113266774711828 | -7.4135979786172 | 1.22918155103001E-13 | 6.8529862092083E-11 |
| AC007608.1 | 20.7215220588022 | 0.931716666195765 | 0.215398337319681 | 4.32555180225447 | 0.0000152150506396742 | 0.00029880093255981 |
| KRT6A | 150.611957068561 | -1.51433895120466 | 0.254844207842118 | -5.9422145161833 | 2.81197099063886E-09 | 2.80710250355059E-07 |
| LINC01597 | 8.79112146015609 | 0.729672627942496 | 0.219334487864821 | 3.32675738797723 | 0.000878628345386249 | 0.00711102009263672 |
| AP001042.1 | 120.464326284896 | 0.884555446582794 | 0.160738470060663 | 5.50307245209541 | 0.0000000373228947964 | 0.0000024131085318574 |
| LINC01446 | 1.79810373957203 | -3.23724672495524 | 0.622799673570138 | -5.19789406182255 | 2.01558935262609E-07 | 9.19581707101249E-06 |
| ADARB2-AS1 | 0.596519204339143 | 1.12966182732216 | 0.320883138450939 | 3.52047736997213 | 0.000430770749600681 | 0.00410539624056596 |
| CYP2D7 | 55.5450031418396 | 0.5703631483457 | 0.133066827967803 | 4.28629100923414 | 0.0000181680960203699 | 0.000344236525276606 |
| SLCO1B7 | 1.4538018623781 | -1.03319469955351 | 0.298562082070537 | -3.46056904610349 | 0.000539034980305142 | 0.0048918338701125 |
| OR7E91P | 6.57425902265952 | 0.70804588235347 | 0.230055715399439 | 3.07771480975428 | 0.0020859443862488 | 0.0135874858131929 |
| KRTAP5-1 | 33.5973213392103 | 0.599460912871957 | 0.119684886908104 | 5.00866006024831 | 5.48102828861674E-07 | 0.0000209004045641871 |
| OR7E136P | 0.690360129629945 | 1.01644366878889 | 0.353214316925538 | 2.87769668465385 | 0.00400590101822319 | 0.0223461787908958 |
| BHLHA9 | 5.04955996991904 | 0.83358819422447 | 0.314023363880802 | 2.65454195484922 | 0.00794161632011518 | 0.0374377689564832 |
| ONECUT3 | 141.439523042553 | -2.63593132379395 | 0.320334582908472 | -8.22868171104431 | 1.89284997124418E-16 | 0.0000000000002877304 |
| C21orf62 | 5.38758769074399 | 0.765014002122211 | 0.172148675967594 | 4.44391452808047 | 8.83367096650906E-06 | 0.000192830042077021 |
| TMEM211 | 136.498733918298 | 1.07397950152771 | 0.183373728351164 | 5.85677954625548 | 4.71928906283542E-09 | 0.0000004250627939817 |
| SERPINB4 | 2.77904899872736 | -1.55546353980927 | 0.404844979738239 | -3.84212134930014 | 0.000121975500721596 | 0.00154161175175042 |
| SERPINB5 | 2476.59674028079 | -0.723415356129018 | 0.165522521635306 | -4.37049501772882 | 0.0000123965183248578 | 0.000254646416351286 |
| FOXL2NB | 2.15876333130199 | -1.49589135571809 | 0.326055520675366 | -4.58784244051327 | 4.47850429733599E-06 | 0.000111768761725008 |
| COL6A6 | 8.46106181500276 | -0.566965460595039 | 0.193383485234765 | -2.93181943590865 | 0.00336982610313952 | 0.0195818808933435 |
| RNVU1-7 | 0.888391602191164 | 1.29022915826272 | 0.312969591791943 | 4.12253839382722 | 0.0000374720129961726 | 0.000607435316828891 |
| RNVU1-30 | 4.39942484227165 | 0.647954076194134 | 0.148112057198508 | 4.3747557656681 | 0.0000121568511424848 | 0.000250646988845239 |
| SNORA5A | 9.20697552400715 | 0.736582384637125 | 0.142449662819076 | 5.17082575037506 | 2.3306181530727E-07 | 0.0000103885568562913 |
| SNORA70J | 1.09564552051858 | 1.07024446121829 | 0.332222709417513 | 3.22146689819836 | 0.00127536193921585 | 0.00936348056449101 |
| SNORA54 | 1.74758049323627 | 1.63865869971569 | 0.311109905265709 | 5.26713766415173 | 1.38567296517823E-07 | 6.88553867778459E-06 |
| SNORD116-1 | 0.349306632995515 | 1.45027127881275 | 0.539444408487425 | 2.68845363117071 | 0.00717838045557599 | NA |
| SNORA37 | 0.69503558383811 | 1.03134712504512 | 0.347898922684718 | 2.96450220968282 | 0.00303173065752667 | 0.0181145500534227 |
| SNORD116-24 | 0.613058381906877 | 1.21776501400657 | 0.354051759485947 | 3.43951126178461 | 0.000582765537949998 | 0.00518734232635716 |
| MIR23B | 0.623835438178973 | 1.08508027142694 | 0.353041969899247 | 3.07351636332814 | 0.00211552085526848 | 0.0137346628697124 |
| MIR200A | 12.1399618710574 | 0.699748353572721 | 0.16472462693442 | 4.24798869844339 | 0.0000215698273088997 | 0.000396122001572885 |
| MIR200C | 0.498922643752752 | 0.934017194358954 | 0.297304685609854 | 3.14161612503021 | 0.00168018165549733 | 0.0115852855511632 |
| MIR200B | 8.25924635146774 | 0.657364792279562 | 0.168260643975964 | 3.9068244168462 | 0.0000935170576465541 | 0.00125196054516255 |
| MIR483 | 1.6522987267267 | 2.13051732519641 | 0.654073086608333 | 3.25730773642455 | 0.00112474423694646 | 0.00856024050340541 |
| MIR199A2 | 0.617379008189202 | 1.00422410105005 | 0.308388413722027 | 3.25636131698267 | 0.00112850044252857 | 0.00857550235785112 |
| SNORD41 | 0.804462198943738 | 1.56863088711948 | 0.421822195379197 | 3.71870163377571 | 0.000200249412785604 | 0.00226164838310577 |
| MT-TV | 9.39085885838733 | 0.729660464802026 | 0.252123876563224 | 2.89405539351626 | 0.00380301064747386 | 0.0214831557555441 |
| MT-TM | 39.2273038012449 | 0.824225233445789 | 0.240506745883238 | 3.4270358214648 | 0.000610208567709107 | 0.00536913044517503 |
| MT-TS2 | 2.58069738384123 | 0.805554318588801 | 0.26502626147506 | 3.039526400536 | 0.00236950433998877 | 0.0149878880533203 |
| MT-TL2 | 1.57877362937511 | 1.02584668168475 | 0.26417869216061 | 3.88315451672035 | 0.000103109997894272 | 0.00134695490217978 |
| IGLV3-1 | 451.945095062186 | -0.751119015202884 | 0.211988017926011 | -3.54321448236307 | 0.000395281077149434 | 0.00385056503991593 |
| TRBV27 | 1.25534915198385 | -0.749014895116335 | 0.219423194211491 | -3.4135629909498 | 0.000641193341877262 | 0.00558115245680879 |
| TRAV8-1 | 1.93369782792072 | -0.560647067066166 | 0.208881067909635 | -2.68404921842275 | 0.00727363974419843 | 0.0350851089464134 |
| TRAV19 | 6.2256227391896 | -0.542122201700801 | 0.16017825615048 | -3.38449309369122 | 0.000713098128768083 | 0.00608197593018675 |
| TRAV23DV6 | 2.89390795480519 | -0.567883327097688 | 0.184506659600389 | -3.07784731633877 | 0.00208501713168798 | 0.0135867386823674 |
| TRDV1 | 4.20761496170979 | -0.732624485716656 | 0.2470300646489 | -2.96573004892309 | 0.00301965413241272 | 0.0180671445799108 |
| TRAV24 | 1.44081920752893 | -0.653363864827532 | 0.216192462326493 | -3.02213989237434 | 0.00250994524000764 | 0.0156853464290457 |
| TRDC | 28.656609153014 | -0.787461821637989 | 0.172779314300456 | -4.55761631435014 | 5.17374397620683E-06 | 0.000126292223395846 |
| TRAJ49 | 0.692940376187969 | 0.704127066630259 | 0.23404316830384 | 3.00853501400282 | 0.00262510550743369 | 0.0161912169641456 |
| IGHG1 | 45843.1924984474 | -0.635572927652642 | 0.194218604475072 | -3.27246161288435 | 0.00106615349186702 | 0.00819074318286628 |
| IGHG3 | 5601.97984482745 | -0.749836688629166 | 0.197898209972986 | -3.78900187491095 | 0.00015125377243538 | 0.0018201614457661 |
| IGHD | 70.1421023178161 | -0.658841177014703 | 0.233039856555167 | -2.82716092755034 | 0.00469627205901986 | 0.0252330484648988 |
| IGHV2-26 | 134.766328566712 | -0.766343702641267 | 0.234106968005406 | -3.27347668961127 | 0.00106233152562413 | 0.00816513235576238 |
| MIR374B | 1.06616480105074 | 0.796512794008544 | 0.258751965764175 | 3.07828692878214 | 0.00208194351900842 | 0.0135825897703238 |
| RNA5SP18 | 2.09736990358157 | -0.858277815078508 | 0.237267049735292 | -3.61734937925873 | 0.000297635426979218 | 0.00309560029136714 |
| SNORA74B | 1.30967545039192 | 1.13929458766601 | 0.29016646467831 | 3.92634823920497 | 0.0000862452652534756 | 0.00117339876346897 |
| SNORA12 | 2.16814394837635 | 1.38855569943157 | 0.231304880005038 | 6.00314052778015 | 1.93536970186865E-09 | 2.08111361961065E-07 |
| RNA5SP354 | 0.647135293622538 | 2.04049925598467 | 0.726701191840745 | 2.80789309126638 | 0.00498667784839358 | 0.0264327740184138 |
| EWSAT1 | 4.40288055343086 | 1.04233520973088 | 0.15927751876647 | 6.54414519891619 | 5.98366404558728E-11 | 1.17709231183841E-08 |
| CGB8 | 0.929356182139576 | -1.41232038326314 | 0.481231049891285 | -2.93480726894534 | 0.00333754921195827 | 0.0194823391074024 |
| AL365357.1 | 715.983890405795 | 0.845859191414507 | 0.293492757151755 | 2.88204451661184 | 0.00395103908887947 | 0.0220917320197805 |
| RPL29P7 | 1.42706764889731 | 0.627020571568487 | 0.205959686667719 | 3.04438495568348 | 0.00233156585737562 | 0.014803915967791 |
| PPP1R14BP2 | 13.3158036411128 | 0.596611674723793 | 0.200068206624496 | 2.9820413987294 | 0.00286333276991711 | 0.0173313256998313 |
| AL356535.1 | 22.6415300164961 | 0.672097669695349 | 0.172423989055912 | 3.89793597384762 | 0.0000970160571201497 | 0.00128848728443687 |
| RPL13P6 | 3.70423485854847 | 0.511197283115681 | 0.181886059573579 | 2.81053580639524 | 0.00494590852713635 | 0.0262708184505231 |
| ASIC3 | 36.8128034207598 | -0.540490653446367 | 0.132972190231863 | -4.06468940989779 | 0.0000480964667030476 | 0.000739053839513481 |
| NOC2LP1 | 53.0525099661058 | 0.635367986173524 | 0.190324521500551 | 3.33834012120023 | 0.000842805161080222 | 0.00690980392175651 |
| IFITM9P | 5.64495881765632 | 0.594398010451247 | 0.172805778898953 | 3.4396882687518 | 0.000582384564471563 | 0.00518671227831106 |
| AC012618.1 | 75.4985704945176 | 0.694285038181266 | 0.225188079341811 | 3.0831340638037 | 0.00204832855011479 | 0.0134314124260664 |
| RPL7AP34 | 9.40779244905735 | 0.686371994515595 | 0.178165347569518 | 3.85244383309601 | 0.000116944806748762 | 0.00149147556636683 |
| AL122020.1 | 176.15531868572 | 0.649082055485973 | 0.189579391463275 | 3.42380071207114 | 0.000617518881498487 | 0.00542022741078016 |
| AC004884.1 | 13.9748862418069 | 0.790762092795163 | 0.240953099603502 | 3.28180917405251 | 0.00103143381221733 | 0.00797899827623687 |
| FTH1P12 | 23.7346879004046 | 0.510771334885253 | 0.167851018729806 | 3.04300408034731 | 0.00234229157123683 | 0.0148582667331958 |
| AC091980.1 | 0.770043030007542 | 0.675803325767854 | 0.239186453761636 | 2.82542474767962 | 0.00472179777528711 | 0.0253190898771688 |
| MAGEA12 | 34.4024796016884 | -3.68051619716175 | 0.533942031270823 | -6.89310071432631 | 5.45891965255416E-12 | 1.65961082746106E-09 |
| HNRNPA1P33 | 0.824385079810428 | -0.553625924055388 | 0.19241622323957 | -2.87723100856256 | 0.00401181784287269 | 0.022358236027197 |
| YBX1P6 | 33.356157708183 | 0.564838149591468 | 0.191541504189718 | 2.9489073502942 | 0.00318899563430233 | 0.0188421187283284 |
| HMGN2P32 | 1.11325717761097 | 0.853487345463698 | 0.294889140021521 | 2.89426509705108 | 0.0038004715169918 | 0.0214733458830948 |
| KRT18P19 | 2.73547369235903 | -0.625530579557253 | 0.156955546066316 | -3.98539965763909 | 0.0000673666414819258 | 0.00096112424250792 |
| RPL11P3 | 49.6432440056009 | 0.811637785636123 | 0.220338955484686 | 3.68358733411775 | 0.000229974440961013 | 0.00251992308473729 |
| BOLA3P3 | 9.10080079218428 | 0.847565581391342 | 0.164830698067543 | 5.14203720137153 | 2.71775284419712E-07 | 0.0000118496858690535 |
| RPL7AP4 | 11.087924701399 | 0.824205594913062 | 0.236136870689041 | 3.49037231038106 | 0.000482348053830656 | 0.00447825752809684 |
| SAPCD2P1 | 0.772513139995191 | 1.28197553304153 | 0.389069220678526 | 3.29498059704082 | 0.000984284757577568 | 0.00771265914677329 |
| AP002982.1 | 4.11529097398107 | 0.590384272904359 | 0.18268588387563 | 3.23169070526699 | 0.00123060177476823 | 0.00912703139316901 |
| PSMC1P2 | 0.742919118786871 | -0.720878171427629 | 0.26209198590789 | -2.75047773372581 | 0.00595084357235444 | 0.0301847581900011 |
| AC004129.1 | 1.58418664574388 | 0.52521640141836 | 0.198574832820673 | 2.64492933952343 | 0.00817079774543534 | 0.0381638109348175 |
| KLRK1 | 7.8817471031853 | -0.612495746588863 | 0.157188525322321 | -3.89656780183489 | 0.0000975655026954636 | 0.00129372939775642 |
| RPL13P2 | 51.4401472924398 | 0.972985358054277 | 0.224449784800067 | 4.33498013340037 | 0.0000145773286823631 | 0.000290476675828294 |
| CEACAM18 | 22.5297532241646 | -1.50740355873526 | 0.274989061677059 | -5.48168552429742 | 4.21292613333769E-08 | 2.63311778269386E-06 |
| CNN2P6 | 1.19135366280873 | 0.727122327221527 | 0.237080588622013 | 3.06698381106522 | 0.00216230567703584 | 0.0139732997973782 |
| AL049830.1 | 20.6646130388227 | 1.08510827534782 | 0.258199059293285 | 4.20260351961724 | 0.0000263862514551422 | 0.000466143170186405 |
| UBD | 572.400233074623 | -0.690175709584209 | 0.159222345730669 | -4.33466613255196 | 0.0000145981507608647 | 0.000290476675828294 |
| SEPHS1P6 | 3.8159555668291 | 0.634051168578329 | 0.150317033896998 | 4.21809260161959 | 0.0000246377639692779 | 0.000439667077193486 |
| ATP5PDP3 | 1.31179206529119 | 1.160932886916 | 0.363640876621697 | 3.19252581750798 | 0.00141034336327509 | 0.0101112870633931 |
| PGAM1P7 | 1.58733163460544 | -0.563057796517963 | 0.208157438788298 | -2.70496120530484 | 0.0068312366898981 | 0.0334431587444843 |
| AL034370.1 | 8.3040879987839 | 0.662027336818677 | 0.183671950579889 | 3.60440086103799 | 0.000312873846158491 | 0.00321406680246935 |
| UCA1 | 954.640135381382 | -0.610031158136595 | 0.200168407444925 | -3.04758960678869 | 0.00230684737013966 | 0.0146926655188733 |
| CPNE1 | 7581.13052837021 | 0.608995626051268 | 0.092973067658882 | 6.55023698137693 | 5.74458492543656E-11 | 1.13674798270088E-08 |
| LINC00887 | 4.15376412230477 | -0.832586459237658 | 0.178364884149274 | -4.66788327314957 | 3.04318699687984E-06 | 0.0000821090633470969 |
| AC018695.1 | 36.0212778758719 | 1.00213303311241 | 0.289371532115572 | 3.46313621725639 | 0.000533918024490033 | 0.00485360251272419 |
| ATP5MC1P6 | 1.88322550321535 | -0.871990295953337 | 0.19537245496057 | -4.46322024324934 | 8.07369894647986E-06 | 0.000179641144489807 |
| CBX1P2 | 1.0042448218166 | 0.584997840602724 | 0.207821477020442 | 2.81490560547398 | 0.00487915630607215 | 0.0259657455741032 |
| VSTM5 | 82.1067897572142 | -0.565262486022788 | 0.148493338489791 | -3.80665214865278 | 0.000140860648284547 | 0.0017187331187771 |
| AS3MT | 2.84770597816742 | 0.814465059585933 | 0.180715189901688 | 4.5068987284855 | 6.57820100289513E-06 | 0.00015192555106272 |
| PLIN5 | 34.9779975248448 | 0.58169277782637 | 0.137764848122092 | 4.22235995433938 | 0.0000241757612833943 | 0.000432576676746535 |
| HMGN2P15 | 7.03236697829194 | 0.561256165479966 | 0.145136205114441 | 3.86709963263414 | 0.000110137438998822 | 0.00141935115028848 |
| AL354710.1 | 7.14209907770828 | 0.882655864612374 | 0.203355659983033 | 4.34045388599471 | 0.0000142188707062685 | 0.0002854186519562 |
| LINC01913 | 4.09352708231324 | -2.03196410032826 | 0.349262948008409 | -5.81786333739397 | 5.96046007551717E-09 | 5.17739495702455E-07 |
| CAPN14 | 16.977803452808 | -1.33894435400364 | 0.208944648896617 | -6.40812943080503 | 1.47315826588233E-10 | 2.53945148080603E-08 |
| TUBAP5 | 271.191517036119 | 0.632219089884616 | 0.0981512826961836 | 6.44127180529652 | 1.18476478459459E-10 | 2.09634412309061E-08 |
| FER1L6 | 139.440332455295 | -0.638948677999625 | 0.206471766194534 | -3.09460557138655 | 0.00197074784566471 | 0.0130171340025122 |
| SMTNL1 | 9.78205620749394 | -0.670057632510523 | 0.161033406729511 | -4.16098526460429 | 0.0000316877533514789 | 0.000533405422898604 |
| TMEM14DP | 7.83574125210604 | 0.533981255268493 | 0.188503768060938 | 2.83273517957408 | 0.00461516028339017 | 0.0248975948051515 |
| LINC00243 | 2.86733221832185 | -0.579685437903857 | 0.15994308212299 | -3.62432329182016 | 0.000289719023522255 | 0.00303343255624022 |
| AC129492.1 | 24.1501564224921 | -0.594215485793232 | 0.168014182125452 | -3.53669838031618 | 0.000405162088900636 | 0.00392725761090801 |
| RPL15P20 | 6.90321973189184 | 0.513124143974536 | 0.175155441386155 | 2.92953584492578 | 0.00339468657472874 | 0.0196955427536569 |
| RPL13P12 | 1869.52263587535 | 0.607569350676968 | 0.150887191381607 | 4.02664629856069 | 0.0000565780422118305 | 0.000837714096519282 |
| FDPSP5 | 2.98690505084403 | 0.618453054580107 | 0.234035536573345 | 2.64256045742134 | 0.00822817740856346 | 0.0383668026906273 |
| PRSS41 | 9.16293635671717 | 0.664167225557133 | 0.200131335615093 | 3.31865683859976 | 0.000904515114188535 | 0.00726261571397191 |
| MUC5AC | 1490.81535420175 | -1.26018777056424 | 0.268464764996836 | -4.69405275801869 | 2.67844907467727E-06 | 0.0000745817601626621 |
| AL353795.1 | 0.609081022901223 | 1.00743003528443 | 0.388751269992837 | 2.59145143192198 | 0.00955720307582848 | 0.0428606658524683 |
| C5orf49 | 2.88107774590299 | 0.57509348974088 | 0.190664514430729 | 3.01625864392201 | 0.0025591486324568 | 0.0159092586143384 |
| RPL37P3 | 0.794216433694385 | 1.56251558252949 | 0.410578524539241 | 3.80564371768586 | 0.000141435857804542 | 0.00172435215337203 |
| AL138807.1 | 0.657736511881153 | 0.886515647403784 | 0.258624137268137 | 3.42781480788338 | 0.000608460378723809 | 0.00536182661008738 |
| ZNF663P | 19.6327662832516 | 1.02869578766081 | 0.286227590104384 | 3.5939784396244 | 0.00032566679762502 | 0.00331737710818639 |
| KRTAP10-4 | 0.888623057294644 | 0.880153941644755 | 0.2798388098067 | 3.14521757097496 | 0.00165963353653754 | 0.0114941595194721 |
| EFCAB8 | 6.11482667200833 | 0.541008963690208 | 0.166852147672691 | 3.24244531003275 | 0.00118508690979418 | 0.00888602610702626 |
| CELA2B | 1.97462714469959 | -0.526787040867668 | 0.158912768396763 | -3.31494470949256 | 0.000916612526565865 | 0.00732807939598749 |
| KRT18P65 | 2.87980647310158 | -0.562822571416759 | 0.15283076101663 | -3.68265241678353 | 0.00023081977891105 | 0.00252670214282924 |
| LINC01139 | 4.34607094521471 | -1.8346586149241 | 0.391510442591255 | -4.6861039076792 | 2.78454932501675E-06 | 0.0000766427148372101 |
| NBPF7 | 8.832159448528 | -1.22110539898098 | 0.268246312313627 | -4.55217963091063 | 5.30929728252602E-06 | 0.000128755271734761 |
| H1-12P | 4.43602793146987 | -0.53968445254391 | 0.15516217503019 | -3.4781959742373 | 0.000504800715359247 | 0.00464033686724682 |
| AL031229.1 | 7.99933350327007 | 0.64809194620087 | 0.20384148030281 | 3.17939187469655 | 0.0014758441885424 | 0.0104944038599266 |
| NUDT19P3 | 0.651699626567402 | 0.62879421994669 | 0.209401436742702 | 3.00281712354872 | 0.00267493118631167 | 0.0164318605313436 |
| AL589993.1 | 1.03325352569729 | -0.695299949248421 | 0.21133717975733 | -3.29000297082986 | 0.00100186324984653 | 0.00782262685065789 |
| AL139100.1 | 67.2038829687681 | 0.543013226555969 | 0.17123208194663 | 3.17121196205053 | 0.00151804331762736 | 0.0107305864781429 |
| PIMREGP3 | 1.07463570699252 | 0.701124465053654 | 0.239172303919927 | 2.93146176861843 | 0.00337370889161502 | 0.0196010376569474 |
| KRT18P48 | 1.94427092719422 | -0.515900961155952 | 0.162096791058822 | -3.18267226504651 | 0.00145922682866438 | 0.0104027848228937 |
| KRT18P22 | 1.63814953977431 | -0.624843150506851 | 0.208901340069681 | -2.99109211218285 | 0.00277981628357648 | 0.0169608859980596 |
| RPS4XP7 | 15.1721642662817 | 0.77816387854552 | 0.143898212695539 | 5.40773831702808 | 6.3825585636043E-08 | 3.66744885711435E-06 |
| H2AC9P | 0.665846524675464 | -0.724065634848816 | 0.262309532998866 | -2.76034815269922 | 0.00577397927717106 | 0.0295430561485854 |
| RAET1K | 8.85757200525725 | -0.728763074192855 | 0.136232080089812 | -5.34942337893112 | 8.82349068564866E-08 | 4.75927702434617E-06 |
| AL512378.1 | 0.536955529838123 | -0.733415279376192 | 0.246472565514058 | -2.97564671283612 | 0.00292371462838708 | 0.0176075751130057 |
| DBIP1 | 2.88684279813633 | 0.731651021868228 | 0.268733319036503 | 2.72259139466381 | 0.00647721145872935 | 0.0320987817008022 |
| RPS4XP8 | 36.0865225566064 | 0.668280791058337 | 0.230882944821091 | 2.89445715263282 | 0.00379814742292004 | 0.0214731159268509 |
| ZNF602P | 1.11655138765612 | -0.59293553854708 | 0.179411752441754 | -3.3048868342088 | 0.000950147558382938 | 0.00752601483833307 |
| PPP1R3G | 101.984149909083 | 0.505985732357088 | 0.12619447598827 | 4.0095711669988 | 0.0000608291181887428 | 0.000885225139455152 |
| AL356432.2 | 0.595045329947399 | 1.17934942273399 | 0.381563103912371 | 3.09083716596675 | 0.00199593056978158 | 0.0131535574343865 |
| RPL31P28 | 1.58837191701365 | 0.686180961953854 | 0.246285164299686 | 2.78612381669442 | 0.00533424922945487 | 0.0278383212751919 |
| ZSCAN12P1 | 32.2059248463751 | -0.556942679137537 | 0.0780931114852303 | -7.13177729181493 | 9.90811013293043E-13 | 4.04081730567633E-10 |
| RPL12P8 | 9.29914546809384 | 0.561074048025929 | 0.166869686755313 | 3.3623485423608 | 0.000772825086392336 | 0.0064660536750394 |
| AL139095.2 | 99.1967143566216 | 0.510335148908382 | 0.175067086770378 | 2.91508334503647 | 0.00355593565681289 | 0.0204045298962143 |
| RPL35P2 | 72.4075999400771 | 1.0305697463751 | 0.195100755323857 | 5.28224375484556 | 1.27611253280526E-07 | 6.40777106938042E-06 |
| SNORA2C | 2.44347482912775 | 0.540096513399415 | 0.197977625252827 | 2.72806845071349 | 0.00637063868153185 | 0.0317222898731072 |
| SNORD93 | 0.474288225976686 | -0.846355518943722 | 0.283151416208727 | -2.98905628047372 | 0.0027984058018001 | 0.0170432137723182 |
| EBF2 | 20.0918018026475 | -0.600372591308464 | 0.15253841277235 | -3.93587805456234 | 0.0000828930591256081 | 0.00113797606045919 |
| MAGEA3 | 107.061564671859 | -3.63014517628577 | 0.525058991900732 | -6.91378536942015 | 4.71888975048607E-12 | 1.51739529842072E-09 |
| HMSD | 11.7583021836203 | -1.41688878928863 | 0.191484191107446 | -7.39950792331252 | 1.36690067320327E-13 | 7.37288585697803E-11 |
| XKR9 | 93.8489061795764 | -0.728440015469911 | 0.179241198026494 | -4.06402112622701 | 0.0000482344572362554 | 0.000740613736866323 |
| MYBPHL | 16.0618121066397 | 1.08976242856885 | 0.285845552609088 | 3.81241694552154 | 0.0001376144783418 | 0.00168884527879136 |
| RNU4-62P | 0.782147011728752 | -0.813062811919713 | 0.235601055730675 | -3.45101514676216 | 0.000558482225302645 | 0.00502436175556395 |
| RNU2-33P | 0.750389303047878 | -0.641835915567557 | 0.208971264003613 | -3.07140753838987 | 0.00213052137968482 | 0.0138213183277245 |
| RN7SKP271 | 1.86112854879217 | 1.14032713509088 | 0.19684709402171 | 5.79295894998131 | 0.0000000069157015527 | 5.8848572856329E-07 |
| SNORA79B | 4.51943843003941 | 1.72647529634356 | 0.224690431849098 | 7.68379535405878 | 1.54443310519312E-14 | 1.1228028674754E-11 |
| RNA5SP221 | 1.34526983947087 | 2.48296809509444 | 0.446143515514996 | 5.56540218281169 | 0.0000000261548467528 | 1.79973330268963E-06 |
| AL590113.1 | 2.04236791877296 | -0.524281516576731 | 0.151360952645162 | -3.46378314495558 | 0.000532635709669419 | 0.00484427614978644 |
| RNU2-6P | 3.25635722442175 | 1.0465782797485 | 0.208815820084368 | 5.01196834284707 | 5.38760690241113E-07 | 0.0000206382989725582 |
| KIF28P | 5.77790025609202 | 0.594783319589482 | 0.139814943161058 | 4.25407546677132 | 0.000020991450275468 | 0.00038778336703052 |
| TNRC18P2 | 2.41963988193255 | 0.607527885862914 | 0.194967877343365 | 3.11604093013217 | 0.00183296829123243 | 0.012353511808826 |
| HBD | 4.77092388295753 | 0.807174832444949 | 0.179006492953422 | 4.50919304170143 | 6.50746758460243E-06 | 0.000150603966065242 |
| AL357033.2 | 1.87045655679636 | -1.53881687908384 | 0.332641743722739 | -4.62604861874 | 3.72707844496469E-06 | 0.0000964713292233042 |
| BX842568.2 | 8.03773814298721 | 0.81498761503198 | 0.182518363251793 | 4.46523626725529 | 7.99804400856144E-06 | 0.000178432680276392 |
| LINP1 | 0.779132744995451 | -2.58494025215801 | 0.629798733161621 | -4.10439099993339 | 0.0000405381345715418 | 0.000646483689242489 |
| LINC00114 | 42.7885925416159 | 0.517574577996301 | 0.155688949144256 | 3.32441435850874 | 0.000886044422777313 | 0.00715899917528845 |
| AL603910.1 | 4.34662379783891 | 0.605872951962955 | 0.144641033556955 | 4.18880408320909 | 0.0000280428363976484 | 0.000487703462151569 |
| AC004870.2 | 3.03570232151685 | -1.31699973114346 | 0.306025004083053 | -4.30356903381017 | 0.0000168068446270437 | 0.000323391540861678 |
| OSER1-DT | 287.592303758986 | 0.521209625014846 | 0.106571193737249 | 4.89071771401837 | 1.00468963333438E-06 | 0.0000342146952321471 |
| SEC13P1 | 2.16916788909618 | 0.618394836520682 | 0.200701938843811 | 3.081160252278 | 0.00206195640991692 | 0.0134890348709784 |
| ROR1-AS1 | 1.41800344688247 | -0.716405673853327 | 0.2554512835399 | -2.80447083266046 | 0.00503992473969052 | 0.0266474566236728 |
| AC002456.1 | 0.925782085556294 | -0.589948909276648 | 0.20695837462765 | -2.85056794796566 | 0.00436412247731997 | 0.0238589151359383 |
| RPL21P32 | 1.21472529574067 | 0.556021820379844 | 0.215697055023222 | 2.57779050492823 | 0.00994342611839163 | 0.0440843240423244 |
| IGKV3D-15 | 30.0580761691361 | -0.567530267323161 | 0.206523229299889 | -2.74802146590038 | 0.00599560836480978 | 0.0303094669820127 |
| EGFR-AS1 | 12.9730470207817 | 0.925919059600747 | 0.244697610351005 | 3.78393176080701 | 0.000154370164843563 | 0.00184835197017488 |
| AC007319.1 | 2.02585701194992 | -1.03449081558557 | 0.238825633792516 | -4.33157362196851 | 0.0000148047427127672 | 0.000294002497506153 |
| SNHG14 | 306.788062989068 | 0.608318239584776 | 0.147062329322942 | 4.1364654183393 | 0.0000352696479097162 | 0.000579600769236721 |
| LINC01857 | 5.26480754920221 | -0.623191571459254 | 0.172606657305064 | -3.61047239538292 | 0.000305639831109431 | 0.00315761730984294 |
| MIR3681HG | 1.43367256160588 | -0.770695042295106 | 0.246095394132969 | -3.13169226514937 | 0.00173801944568011 | 0.0119031018436278 |
| HAGLR | 349.870803017156 | -0.668048947177742 | 0.144287085011803 | -4.6299982228007 | 3.65668871148167E-06 | 0.000095017081499122 |
| SOX1-OT | 1.73567987296058 | 1.6744618134399 | 0.397111432595188 | 4.21660439866216 | 0.0000248008496794304 | 0.000441938337372922 |
| AC004009.1 | 4.9302479750214 | -2.11558913243923 | 0.352621615874509 | -5.99960137778997 | 0.000000001978025051 | 2.09997186525678E-07 |
| FAM135A-AS1 | 0.877989106520781 | 0.571695777464104 | 0.201396323328709 | 2.83866044828938 | 0.00453033367943975 | 0.0245787506339753 |
| PIGUP1 | 1.6550052143382 | 0.659038745664367 | 0.206472062695256 | 3.19190275459726 | 0.00141338909222808 | 0.0101234864044317 |
| C4BPAP1 | 0.739303484616193 | -1.33690873277224 | 0.389451849893425 | -3.43279594932748 | 0.000597391509068924 | 0.00529077511818934 |
| FAM96AP2 | 3.97428921895531 | -0.751932292016568 | 0.188058031491797 | -3.99840563070748 | 0.0000637705967796701 | 0.000919627553905015 |
| RPS5P2 | 2.55700752403141 | 0.646886182915968 | 0.221706622650262 | 2.9177575986822 | 0.00352558305292948 | 0.0202799119824782 |
| RPS28P5 | 5.83441852310329 | 0.659382099139114 | 0.169491628671692 | 3.89035201506234 | 0.000100098907067938 | 0.0013173977371767 |
| AF124730.1 | 1.98991088503323 | -1.64240383572749 | 0.223274990787896 | -7.35596866416501 | 1.89547481431709E-13 | 9.75207211390648E-11 |
| LINC00885 | 2.91128001958097 | 0.811158981675485 | 0.228279350337506 | 3.5533611799587 | 0.00038034187653161 | 0.00374319983371692 |
| AC007182.1 | 5.643928991949 | -0.703475426740386 | 0.202887824916047 | -3.4673121811596 | 0.000525690972431106 | 0.00478892876601499 |
| MUC20P1 | 246.433680373474 | 0.58632553841444 | 0.129715030554709 | 4.52010484758086 | 6.18090124089849E-06 | 0.000144749089144347 |
| RPL32P1 | 1.78299602815211 | -0.574295956668949 | 0.222614040487422 | -2.57978317724932 | 0.00988623659706235 | 0.0438831330341066 |
| TOMM20P1 | 1.65823508764889 | 0.647848254335035 | 0.224515821974214 | 2.88553496425496 | 0.00390749055666446 | 0.0219168736755439 |
| NQO2-AS1 | 0.799827254201196 | -1.02023886800209 | 0.204543473519712 | -4.9878827735062 | 6.10445889653619E-07 | 0.0000228606175160093 |
| CDKN2A-DT | 0.499992473050432 | -1.14844576447396 | 0.297300334946083 | -3.86291446554285 | 0.000112042246555456 | 0.00144001414654403 |
| POT1-AS1 | 6.23546970879148 | -0.593376713266715 | 0.151650948019031 | -3.9127794518782 | 0.0000912398578274672 | 0.0012278645172902 |
| PRRT4 | 7.10230943705143 | -0.522882715911699 | 0.201923738980236 | -2.58950591224382 | 0.00961137723787613 | 0.0430182305969608 |
| FTH1P22 | 4.46870094927619 | -0.654945823327605 | 0.149718150987422 | -4.37452519289146 | 0.0000121697068882343 | 0.000250757447785787 |
| RPL3P7 | 122.682149349931 | 0.513389194759977 | 0.180989631499036 | 2.83656688235597 | 0.00456014261971444 | 0.0247033332306927 |
| ZBTB45P1 | 3.52138535285615 | -0.625237134805004 | 0.207049085740215 | -3.01975317867131 | 0.00252980755604298 | 0.0157780351154773 |
| ZNF33BP1 | 1.53075084858211 | 0.618981345235763 | 0.175498065044376 | 3.52699811863594 | 0.000420299749900279 | 0.00403666405403939 |
| RPS12P26 | 19.5762053323178 | 0.565611352345196 | 0.162263883528303 | 3.48575012532926 | 0.000490759184304621 | 0.00453890539025437 |
| HSPA7 | 46.9894300080121 | -0.547446376606264 | 0.134849733787367 | -4.05967710302999 | 0.0000491406238586299 | 0.000749024951267229 |
| LINC00705 | 1.83957257739034 | -0.657918582754246 | 0.221384800825695 | -2.97183266556882 | 0.00296027978240445 | 0.0177925371105625 |
| LHFPL3-AS2 | 147.920944276605 | 0.640836200867441 | 0.183011081664348 | 3.50162512039991 | 0.000462429781833319 | 0.00433181421962741 |
| LINC01678 | 1.24885098921081 | -0.525478161613528 | 0.185373479575084 | -2.83469977915954 | 0.0045868768530818 | 0.0247957079600033 |
| TDGF1P3 | 4.48672698035629 | 0.607440115646133 | 0.191481953022016 | 3.17231000655341 | 0.00151231477370396 | 0.0107059336710855 |
| SFTA1P | 4.54195291621428 | -0.610025497012477 | 0.189738858071611 | -3.21507941605847 | 0.00130408438603142 | 0.00951586079809355 |
| AC019330.1 | 4.93732071500731 | 0.668029209254506 | 0.223456668485998 | 2.98952460797277 | 0.00279411939898995 | 0.0170270482578717 |
| GBP1P1 | 42.6556110580079 | -0.688272673219884 | 0.153442842752825 | -4.48553129538011 | 7.27324695104686E-06 | 0.000165014874177008 |
| AC005237.1 | 0.661022081064503 | 0.741252285911208 | 0.256558021419546 | 2.88921890576576 | 0.00386200119293224 | 0.0217502600023644 |
| BX248409.1 | 9.917218113981 | 0.550826862360034 | 0.185371239563912 | 2.97147963004327 | 0.00296368533511089 | 0.017801711271077 |
| AL138785.1 | 128.112814117214 | 0.721250960381146 | 0.234532478463966 | 3.07527113133698 | 0.00210311270900821 | 0.0136700282244223 |
| AL031846.1 | 2.96322356541761 | -0.710209581763504 | 0.172395138171381 | -4.11966131583985 | 0.0000379429705179438 | 0.000613030019796406 |
| MEG8 | 1.58673214609926 | -0.580986846084888 | 0.18358754560154 | -3.16463104390461 | 0.00155279719616378 | 0.0109185542123863 |
| LSM3P3 | 2.45276058892397 | 0.706814138181894 | 0.26506388822875 | 2.66658028336139 | 0.00766273020784078 | 0.0364986502792496 |
| AL121917.1 | 14.2682356877265 | 0.509098375059505 | 0.165131560599787 | 3.08298651820626 | 0.00204934438979257 | 0.0134324217746822 |
| DNAJC19P5 | 4.68227777291838 | -0.567255683344443 | 0.122789504694874 | -4.61974078936181 | 3.84219750247103E-06 | 0.0000989151415532226 |
| RPS18P1 | 6.18254009360508 | 1.21322219176927 | 0.269285257983822 | 4.50534203339922 | 6.62661193946357E-06 | 0.000152832521710028 |
| TRMT2B-AS1 | 0.885839349905499 | 0.696941029291514 | 0.214171117551566 | 3.25413173008126 | 0.00113739524640283 | 0.00862707458158388 |
| AC010970.1 | 24.4457960191185 | 0.856376013097595 | 0.30272274106686 | 2.82891206018928 | 0.00467065310635769 | 0.0251280536008388 |
| C3orf86 | 3.75826751716559 | -0.524884047484736 | 0.185697658823144 | -2.82655177674926 | 0.00470521367378598 | 0.0252498739408086 |
| NTF4 | 1.91280554010119 | -0.914313929499902 | 0.265455761891679 | -3.44431751258423 | 0.000572502957000565 | 0.00511642006627817 |
| SATB2-AS1 | 371.641234038912 | 0.666718737441135 | 0.157739766611022 | 4.22670041781682 | 0.0000237143056482131 | 0.000425641179451951 |
| MTND1P23 | 83.6334331232798 | -1.39735148615629 | 0.223288058899806 | -6.25806634282806 | 3.89780014738968E-10 | 5.43125968870857E-08 |
| AC010745.1 | 2.08071662345559 | -0.594448215087283 | 0.223694995275828 | -2.65740507226948 | 0.00787447636116574 | 0.0372565980090876 |
| HHLA3-AS1 | 2.50350083373923 | -0.851191343226414 | 0.188450276193585 | -4.51679541372512 | 6.27825091737702E-06 | 0.000146720661900016 |
| AC099342.1 | 0.688946812007085 | -1.12325191812158 | 0.300287964415282 | -3.74058254485413 | 0.000183594237248517 | 0.00211788840360984 |
| DDC-AS1 | 3.25201937058822 | 0.660824682366838 | 0.239435837484994 | 2.75992386648575 | 0.0057814833173088 | 0.0295769259748265 |
| KRT16P6 | 1.10653793476419 | -2.013631243368 | 0.445897304319901 | -4.51590808883508 | 0.0000063046006779078 | 0.000147130780099126 |
| SLC25A39P1 | 2.10137915352964 | 0.84923193293828 | 0.256259326657686 | 3.31395521877997 | 0.00091986238082302 | 0.00734703552411833 |
| PRELID3BP1 | 1.06384736600646 | 1.1192746311136 | 0.334703510534404 | 3.34407795522227 | 0.000825565359754465 | 0.00680013713322877 |
| RPL37AP1 | 47.6650631494053 | 0.570493726634307 | 0.168920330986668 | 3.37729462937965 | 0.000732025943290005 | 0.00620700091163903 |
| UBE2V2P1 | 0.861883560290293 | -1.07000492442363 | 0.388197955982111 | -2.75633837822923 | 0.00584524921194384 | 0.0298164771424383 |
| AC093382.1 | 1.05945903930959 | -0.573957923840197 | 0.182516305346395 | -3.14469396447014 | 0.00166260654923721 | 0.0115044254540846 |
| CROCC2 | 6.56583692859693 | 0.809950329082567 | 0.229933965069105 | 3.52253451915702 | 0.000427441373226746 | 0.00408297469392997 |
| AC005682.1 | 6.08556042931823 | -0.637092741655093 | 0.148928674469637 | -4.27783799140024 | 0.0000188717284785318 | 0.000355353797172895 |
| TMEM252-DT | 2.83166480997854 | 0.74731085592016 | 0.220030488940485 | 3.39639683354199 | 0.000682792908375349 | 0.00587595482292548 |
| ST7-AS2 | 0.876809752449693 | 0.797715621815967 | 0.307844184442892 | 2.59129670829936 | 0.00956150146057982 | 0.0428684450790602 |
| MLXP1 | 10.9412592929121 | -1.42778008619948 | 0.259184432391583 | -5.50874168261134 | 3.61407793994902E-08 | 2.36693558416047E-06 |
| SCDP1 | 6.32985240008163 | 0.739469963403392 | 0.19957489233822 | 3.70522541558088 | 0.000211202819600545 | 0.00235906636375465 |
| RPL39P40 | 15.0691718687265 | 0.672529087602556 | 0.161285775414461 | 4.16979789987267 | 0.0000304869871723241 | 0.000521144618654916 |
| TEX41 | 6.79206191076091 | -0.761484202464141 | 0.180726684387625 | -4.21345749270152 | 0.0000251490935188771 | 0.000447359566733133 |
| FSIP2-AS2 | 8.09948828251681 | 0.524471598884439 | 0.187703620705711 | 2.79414748054715 | 0.00520367409362645 | 0.027318380822811 |
| TLK1P1 | 0.780939704872273 | 0.833811285734316 | 0.257359827883878 | 3.23986572648213 | 0.00119585998501649 | 0.00894874683797752 |
| AL117382.2 | 274.976865799801 | 0.856856919130436 | 0.136142625639989 | 6.29381808307619 | 0.0000000003097508151 | 4.48427998200485E-08 |
| LINC02785 | 0.910283769092306 | -0.849387849758408 | 0.269287964144 | -3.15419908371472 | 0.00160939255993574 | 0.0112297175903635 |
| H3P44 | 4.76044670941643 | 0.58460378009934 | 0.182800132350438 | 3.19804899800959 | 0.00138360769148807 | 0.00996996518395689 |
| AL035416.1 | 2.71855466815872 | 1.11106123685172 | 0.200425011271437 | 5.54352587934748 | 2.96440836811741E-08 | 2.01086703137084E-06 |
| RPS4XP2 | 29.2867303858653 | 0.611288246133452 | 0.168488807986193 | 3.62806440047664 | 0.000285554040888195 | 0.00299921426990672 |
| AC073114.1 | 0.723868760304234 | -0.827811156311053 | 0.287844594202982 | -2.87589613625781 | 0.00402882256552909 | 0.0224254134880865 |
| LINC00658 | 4.61941466181213 | 0.76349786125714 | 0.266310901889991 | 2.86694181814806 | 0.00414459199609426 | 0.0229058743238117 |
| GTF2IP7 | 20.401572972304 | -0.619458317641612 | 0.132997759401113 | -4.65765980142089 | 3.19824147915983E-06 | 0.0000855644732368503 |
| Z98257.1 | 3.66359242275551 | -1.16433080849546 | 0.236696536181901 | -4.91908680742448 | 8.69489050880356E-07 | 0.0000305114930110607 |
| LINC01630 | 4.00806712778801 | -1.76996759787866 | 0.311523410867835 | -5.68165195979307 | 1.33399913492671E-08 | 1.02320181353713E-06 |
| BTF3P13 | 7.7163315730992 | 0.671333410592262 | 0.227018075479772 | 2.95718043232235 | 0.00310466384030147 | 0.0184252294848911 |
| IL21-AS1 | 1.30815825060235 | -0.640491574940649 | 0.243116991822086 | -2.63449942408535 | 0.00842614467531842 | 0.0390527365388816 |
| MGAT3-AS1 | 5.36959344193802 | -0.643539051407621 | 0.155956356634843 | -4.12640475382742 | 0.0000368478510945253 | 0.000599934681744457 |
| TRGC2 | 20.9730961897694 | -0.647450478375554 | 0.147116162505463 | -4.40094730143273 | 0.000010777930092836 | 0.00022783535914325 |
| AC079145.1 | 4.76696652801191 | 0.53338839626527 | 0.149630934984407 | 3.56469333243726 | 0.000364281857673967 | 0.00361815084179768 |
| SPANXB1 | 0.520939107806316 | -2.15485867867037 | 0.786887273446312 | -2.7384591813676 | 0.00617278226138163 | 0.0310048339418931 |
| HCG4B | 22.8558490655644 | -0.564687898823558 | 0.128883492700051 | -4.3813826502805 | 0.0000117928553696394 | 0.000244802401782421 |
| KRT16P2 | 0.778320809482751 | -1.37074325086141 | 0.336847434528538 | -4.06932964408633 | 0.0000471485991903897 | 0.000729271791139372 |
| AC067942.1 | 21.3518166079594 | 0.621247957705608 | 0.185088139286371 | 3.35649793714984 | 0.000789363097830951 | 0.00658046957984565 |
| LINC02832 | 1.4251835971185 | -1.07050471816744 | 0.229150666914917 | -4.67161947455697 | 0.0000029883419549654 | 0.0000808544754514181 |
| AC254562.1 | 6.9751544384593 | 0.538262320551162 | 0.141112190615002 | 3.81442820925167 | 0.000136498609225287 | 0.00168070194761122 |
| RPS15AP30 | 3.52950981802213 | -0.694060042743684 | 0.173006407500221 | -4.01175917569872 | 0.0000602679635494572 | 0.000878205332035271 |
| AL136528.1 | 1.02310912758142 | -0.724180044903962 | 0.234027524322628 | -3.09442253427257 | 0.00197196423752925 | 0.0130200252776808 |
| PHBP11 | 3.12207711948484 | 0.543367548302836 | 0.210037835902479 | 2.58699841372924 | 0.00968160378742535 | 0.0432214916378426 |
| SOX21-AS1 | 1.27920918625872 | 1.02919274434351 | 0.332273175174138 | 3.09742952859235 | 0.00195206802915536 | 0.0129448857884223 |
| LINC00355 | 5.14294731948798 | -1.95770796261701 | 0.473682528120715 | -4.13295371138979 | 0.00003581308726184 | 0.00058651384143509 |
| AL033504.1 | 3.49889164657881 | -0.716962429056698 | 0.194712153931079 | -3.68216577435878 | 0.00023126094670549 | 0.00252987523052829 |
| LINC01940 | 2.12385837516623 | -2.01251133866499 | 0.42906712180302 | -4.69043475111318 | 2.72625158894522E-06 | 0.0000754105092121638 |
| DNAJB3 | 0.883717350349618 | 0.873525696191528 | 0.247805350587551 | 3.52504776075409 | 0.000423406445762383 | 0.00405369549361169 |
| CARM1P1 | 1.01864440634816 | 1.27899919976103 | 0.449504855286189 | 2.84535124530907 | 0.00443624746721538 | 0.024182068100834 |
| MRLN | 8.77748265805926 | 0.999953109553398 | 0.325675595632961 | 3.07039619474698 | 0.00213774983189767 | 0.0138574587862613 |
| RPS26P13 | 5.85823995960401 | 0.588182357050351 | 0.179223336609672 | 3.28184023451893 | 0.00103132020885929 | 0.00797899827623687 |
| RPL3P2 | 94.0535475078555 | 0.554518329632517 | 0.150267417789412 | 3.6902100121906 | 0.000224068989752239 | 0.00246896710223867 |
| FRMD8P1 | 1.51775331899226 | 0.758769388190173 | 0.27211591741213 | 2.78840501285703 | 0.00529682778679851 | 0.0276978031435297 |
| TARID | 6.27108792540034 | -0.562934863259181 | 0.138236717821663 | -4.0722528148087 | 0.00004656060149798 | 0.000723217543927299 |
| MTND5P1 | 2.81935929910602 | 0.583386600468077 | 0.186684762344677 | 3.12498242031649 | 0.0017781568618152 | 0.0121060915661286 |
| AC011933.1 | 7.71526490576916 | 0.528425190475697 | 0.169274537897645 | 3.1217051131176 | 0.00179806944417488 | 0.0121944916552619 |
| FAM245A | 0.875594656227293 | 0.720052798335543 | 0.276783454388572 | 2.60150231857672 | 0.00928164377126045 | 0.0419285061459532 |
| HLA-U | 22.4711276418523 | 0.69377897301765 | 0.182872316052428 | 3.79378895610831 | 0.000148365790485677 | 0.00179574692921535 |
| PHBP3 | 3.44811363035077 | 0.688946197188245 | 0.188296162243349 | 3.65884354189794 | 0.000253355948998758 | 0.0027357861305833 |
| AC131235.1 | 166.990115581674 | 0.552029358191905 | 0.154299416566727 | 3.57765032736323 | 0.00034669683048795 | 0.00348279825929049 |
| ORM2 | 11.7060145450761 | 0.661183562799311 | 0.202927821448163 | 3.25822037649089 | 0.00112113304741475 | 0.00853664193343442 |
| AL031668.1 | 0.847431626407307 | 0.894584325701648 | 0.310021369365333 | 2.88555697800127 | 0.00390721729133079 | 0.0219168736755439 |
| AC005013.1 | 1.96412436727987 | 0.715291954234988 | 0.269132107410523 | 2.65777264971181 | 0.00786589361929055 | 0.0372456795461032 |
| LINC02474 | 28.4167584824944 | -0.907414880428148 | 0.339371927289914 | -2.6738065451506 | 0.00749956998344291 | 0.035910741607431 |
| AL137244.1 | 9.69039909331805 | 0.514700108899343 | 0.130565898898456 | 3.94207149984573 | 0.0000807808856536686 | 0.0011130920387433 |
| AC006460.1 | 1.97880672262738 | -0.615648810623151 | 0.214007532653184 | -2.87676234098198 | 0.00401778068666936 | 0.0223863081845379 |
| AL353689.1 | 0.598995540915819 | 0.83303247575813 | 0.252489749009139 | 3.2992724616633 | 0.000969357765699322 | 0.00762579684792207 |
| MACC1-AS1 | 0.928386165517126 | 0.707225990788891 | 0.247295481161176 | 2.8598419488626 | 0.00423852190749319 | 0.0233247736762197 |
| AC141930.1 | 5.66454325337588 | -1.24521579056161 | 0.231094837791021 | -5.38833235075402 | 7.11144555701316E-08 | 3.99699096332158E-06 |
| HDAC2-AS2 | 31.6871041875319 | 0.568003464718813 | 0.08404351494535 | 6.75844489712456 | 1.3948067624581E-11 | 3.48097968284507E-09 |
| HOTAIR | 20.6992738798221 | -1.0815677857627 | 0.331301640154292 | -3.26460136224799 | 0.00109618233341884 | 0.00838676037387159 |
| AC079779.2 | 1.47394207059436 | -0.547994071568112 | 0.185704243124168 | -2.95089687962436 | 0.00316852682876886 | 0.0187410460218762 |
| SNHG26 | 19.0293667971326 | -0.877525010242256 | 0.132036683719061 | -6.64606975520074 | 3.01022640424608E-11 | 6.57960728175147E-09 |
| PSMD10P1 | 3.6681621257792 | 0.610553818189529 | 0.190098854893004 | 3.21177009999969 | 0.00131919897423277 | 0.00960101242574371 |
| CES1P1 | 13.8565599717718 | 1.86186769594559 | 0.306274466316077 | 6.07908232880286 | 1.20872290876182E-09 | 1.44364683981474E-07 |
| AL022313.2 | 9.69190926087244 | 0.638276606716176 | 0.16086419010305 | 3.9677979686299 | 0.0000725397871350424 | 0.00101671230568738 |
| LINC00954 | 27.4064741449178 | 0.668293898597673 | 0.156353968576227 | 4.2742368785597 | 0.0000191793048417499 | 0.000360054283647373 |
| LAMA5-AS1 | 1.38300697595221 | 0.832263908909174 | 0.222419911147345 | 3.74185883186433 | 0.000182664121483256 | 0.00211007031110295 |
| AL121985.1 | 5.76482247406367 | -0.963581292888357 | 0.188138396686213 | -5.12166208419149 | 3.02854256231284E-07 | 0.0000128528579148307 |
| EEF1DP1 | 57.7659735628652 | 0.543548331430808 | 0.14397835046568 | 3.77520877043505 | 0.000159873710013316 | 0.00189592078378203 |
| AC107419.1 | 1.74014023155642 | -1.43568685742848 | 0.419071872165635 | -3.4258726313682 | 0.000612827682940531 | 0.00538607011851453 |
| LINC02041 | 8.07937130340779 | -0.875589696036954 | 0.192673793341152 | -4.54441510105435 | 5.50880044773808E-06 | 0.000132727164678139 |
| SATB1-AS1 | 88.7131635762777 | 0.511812339905159 | 0.18780614313869 | 2.72521617957512 | 0.00642593976593119 | 0.0319026540457647 |
| HNF4A-AS1 | 3.89788301613614 | 0.562868041401821 | 0.170643393139048 | 3.29850474165835 | 0.000972012384261814 | 0.00764308444732744 |
| RPS3AP54 | 0.775898409195144 | 0.918765088493667 | 0.321663745053206 | 2.85629046674718 | 0.00428622811296916 | 0.023526116394516 |
| LINC02587 | 1.28317111892914 | -0.874972124977536 | 0.297244461942792 | -2.9436111921437 | 0.00324407266350833 | 0.0190747953833349 |
| VIM-AS1 | 18.8869984034769 | -0.6530544409681 | 0.123515227459516 | -5.28723829765971 | 1.24176829313677E-07 | 6.27299324155286E-06 |
| ACTBP1 | 10.0679945875612 | -0.732796405610191 | 0.17311758058833 | -4.23294042765515 | 0.0000230655617918245 | 0.000417176050536611 |
| MRPS16P1 | 0.720448638669413 | 1.07079560007917 | 0.39291916367794 | 2.72523129199384 | 0.00642564562513287 | 0.0319026540457647 |
| AC087071.1 | 4.75793801798523 | 0.613771498742324 | 0.185129423859148 | 3.31536438642677 | 0.000915237367933789 | 0.00732320706025383 |
| ORM1 | 21.544412425175 | 1.38972506118698 | 0.297358417522108 | 4.67356892993842 | 2.96010294774887E-06 | 0.0000804157293083816 |
| LINC00452 | 0.665889038284527 | -0.893261545017379 | 0.305574700968373 | -2.92321825788134 | 0.00346433586565545 | 0.0200059264408996 |
| SFTA3 | 1.9432414947582 | 1.77578908184463 | 0.684677044722538 | 2.59361562583752 | 0.00949725984506322 | 0.0426375840701576 |
| AL512625.2 | 2.48861027850282 | 0.819850433476468 | 0.195813843008066 | 4.18688699880476 | 0.0000282806535456463 | 0.000490794818823821 |
| LINC00404 | 1.12824474037228 | 1.76726519502198 | 0.417753914265432 | 4.23039769269308 | 0.0000233278522479948 | 0.000420555274866546 |
| LINC02572 | 0.722374783388005 | 0.75713248932686 | 0.240681713680892 | 3.14578319120124 | 0.00165642746836856 | 0.0114806730356853 |
| AC087857.1 | 1.41941204467774 | -1.07554626920731 | 0.323185138464014 | -3.32795707846903 | 0.000874853441009964 | 0.00709085040578168 |
| MYOSLID | 3.55730431989055 | -0.554166846904451 | 0.138713950982743 | -3.995033253529 | 0.0000646851732108464 | 0.000931119301018103 |
| ITGB1-DT | 7.60492437608026 | -0.580224161953899 | 0.124343556262249 | -4.66629859556347 | 0.0000030667395318316 | 0.0000824989540460063 |
| AL121972.1 | 2.40311030082816 | 0.524958912924024 | 0.141194702645381 | 3.71797881286305 | 0.000200823108240151 | 0.0022657997287957 |
| CASC20 | 0.448903201070266 | -1.32630584566453 | 0.497131517603228 | -2.66791743975301 | 0.00763230104658617 | 0.03638473012914 |
| LINC01315 | 165.072926095291 | 0.56828417642337 | 0.102962851407682 | 5.51931272933813 | 3.40328061917884E-08 | 2.24534900599703E-06 |
| TFAP2A-AS1 | 30.1550377439796 | -0.60215930237879 | 0.178646152480023 | -3.37068161849233 | 0.00074982461750115 | 0.00631628082077417 |
| AL590483.1 | 8.00196590505262 | 0.663375595102239 | 0.141375715108689 | 4.69228816697578 | 2.70166216593796E-06 | 0.0000750406861738349 |
| GTF3AP6 | 0.498010469794837 | 1.35252207543302 | 0.446043813112093 | 3.03226283085586 | 0.00242727755901461 | 0.0152666947768604 |
| AP001042.2 | 0.809373067591597 | 1.21867281604907 | 0.318936847625742 | 3.82104741148985 | 0.000132886100137541 | 0.00164469909726116 |
| AC093585.1 | 6.82220067407353 | 0.8817779251881 | 0.181818640356559 | 4.84976635761257 | 1.23606984054691E-06 | 0.0000402499002994838 |
| ALMS1-IT1 | 18.8185904035339 | -0.529748944276346 | 0.112966829329702 | -4.68942031408384 | 2.73980100949801E-06 | 0.0000756601365480035 |
| AL022316.1 | 15.9937650923817 | -0.871302160070299 | 0.184033422884772 | -4.73447782697518 | 2.19619777834402E-06 | 0.0000636992594131662 |
| SEPHS1P4 | 5.09449528827761 | 0.721233666907364 | 0.20846667370788 | 3.45970727157097 | 0.000540762911101221 | 0.00490219389347981 |
| CT45A11P | 1.19521302093732 | -0.51115389747292 | 0.176855024159651 | -2.89024244519901 | 0.00384944819881743 | 0.0216941770584517 |
| ATP6V0CP1 | 2.02893431220552 | 0.664850836275195 | 0.222036674852017 | 2.99432891759122 | 0.0027504925811673 | 0.0168280228502373 |
| HSPB1P2 | 23.3942489551159 | 0.572612897105423 | 0.18695540917088 | 3.06283139730954 | 0.00219253554492497 | 0.0141222599563522 |
| H3P1 | 2.11492637549309 | -0.548168393210957 | 0.210183145838804 | -2.60805114046283 | 0.00910593489258966 | 0.0413694708161912 |
| NFE4 | 1.17136339797695 | -0.773438692370309 | 0.231009852602094 | -3.34807664546901 | 0.000813745029657163 | 0.00672596670336007 |
| XXYLT1-AS2 | 5.93199210058806 | -0.917305516125829 | 0.202909270231729 | -4.52076691754021 | 6.16160008034827E-06 | 0.000144398198939738 |
| ARMC2-AS1 | 0.62566417063116 | 0.813225003942827 | 0.289888238190643 | 2.80530527564218 | 0.00502689447424346 | 0.0265953812699968 |
| AC078817.1 | 49.6757539249387 | 0.600601397494494 | 0.187794290229557 | 3.1981877444747 | 0.00138294212958482 | 0.00996731696068439 |
| FEZF1-AS1 | 151.647466368587 | -0.694747115713758 | 0.217456735111777 | -3.19487513392788 | 0.00139891350499954 | 0.0100493983060224 |
| HMGN2P3 | 13.4474917817557 | -0.652766542713109 | 0.185846495201783 | -3.51239630321985 | 0.000444085263697159 | 0.00420306714183721 |
| NCAPD2P1 | 15.8924968930839 | -0.515256139800769 | 0.195838651613779 | -2.6310237307849 | 0.00851280915608985 | 0.0393537964885204 |
| FAM41C | 1.21071588344706 | 1.24734172085843 | 0.299359002464932 | 4.16670856926894 | 0.0000309029153674114 | 0.000525447871910951 |
| AP000695.1 | 7.87762877108481 | -0.559699545272323 | 0.134281798304138 | -4.16809688536228 | 0.000030715338383304 | 0.000524072625619619 |
| HMGB1P27 | 0.672249830778936 | 0.54973572281413 | 0.202788262426344 | 2.71088531573075 | 0.0067103831339695 | 0.0329867165612536 |
| AC104461.1 | 0.767760002526177 | -0.637938536315004 | 0.246421216399849 | -2.58881335639489 | 0.00963072784584247 | 0.0430750183239196 |
| AL445933.1 | 16.5759332942455 | -0.527008713566867 | 0.156125594409462 | -3.37554336020466 | 0.0007367008228933 | 0.00623557299903764 |
| AL645608.2 | 6.83172265920285 | -0.890377487192252 | 0.228339830586303 | -3.89935249100452 | 0.0000964502755307147 | 0.00128250103948237 |
| AL035252.2 | 3.87324893104902 | 1.04084999856398 | 0.315406897636691 | 3.30002294294435 | 0.00096676924615306 | 0.00761702983476846 |
| FOXD3-AS1 | 3.21300909113204 | -0.707542870730941 | 0.253595725543645 | -2.79004257352583 | 0.00527011111621209 | 0.0275853898807896 |
| AC099535.1 | 3.38530925293108 | 0.5539508823116 | 0.214688165048452 | 2.58025812548438 | 0.00987264894778094 | 0.0438402770032785 |
| AC005532.1 | 6.20725196427625 | -1.06397396505485 | 0.166509507896416 | -6.38986913418024 | 1.66027781529462E-10 | 2.7901010401549E-08 |
| AC025181.1 | 2.94737340763898 | 0.808028029725976 | 0.215808287386436 | 3.74419369854452 | 0.000180974002613122 | 0.0020934391544061 |
| LINC01293 | 3.65906312271494 | -0.615404845637467 | 0.179927642790308 | -3.4202907129433 | 0.000625542478169986 | 0.00547198314280949 |
| STMND1 | 21.483896792645 | 0.948118550316826 | 0.193837070326 | 4.8913169638927 | 1.00163512809647E-06 | 0.0000341454454167198 |
| AC005077.4 | 113.055761423733 | -0.528701713708194 | 0.143921504736223 | -3.67354214838978 | 0.000239211197723804 | 0.00260068298903753 |
| NPM1P51 | 0.476153319017559 | 0.607678980711412 | 0.234753694539023 | 2.58858111649611 | 0.00963722460104758 | 0.043086639720352 |
| DPP4-DT | 0.928056524976545 | -0.841366159649672 | 0.233908448418628 | -3.59698918674315 | 0.000321921860731419 | 0.00328623652826011 |
| AC099513.1 | 11.0960673305516 | 0.646644169780241 | 0.114553370554162 | 5.64491613517825 | 1.65261787331779E-08 | 1.23363497588155E-06 |
| AL359976.1 | 2.33601619259821 | 0.79633175336674 | 0.26651798683161 | 2.98790998248788 | 0.00280892273089991 | 0.0170823775171404 |
| FAM201B | 1.35674521320954 | 0.589267036609093 | 0.192380087429917 | 3.06303549645574 | 0.00219104068291968 | 0.0141153501287228 |
| AL031666.1 | 3.34963513565355 | 0.551869509892406 | 0.183007102796978 | 3.01556333856961 | 0.00256502359898054 | 0.0159234303317444 |
| SPATA20P1 | 3.27952367813595 | -0.639127301724267 | 0.232580345512642 | -2.74798500413065 | 0.00599627514960217 | 0.0303094669820127 |
| LNCAROD | 1.24152013114675 | -2.3222377837091 | 0.40253636368688 | -5.7690136673354 | 7.97368382933819E-09 | 6.60039442130514E-07 |
| LINC01098 | 0.604852314006236 | -1.24685659345613 | 0.387170772812455 | -3.22043057227385 | 0.00127998192053509 | 0.00939442833784634 |
| AL356056.2 | 1.09685564841437 | -0.650622950482069 | 0.223760879817614 | -2.90767068404623 | 0.00364131570665461 | 0.0207768093946329 |
| CFAP58-DT | 3.75747536527297 | -0.611426595436748 | 0.155650272977529 | -3.92820766543095 | 0.0000855812977649044 | 0.00116721442082134 |
| AL139811.2 | 0.866589132216416 | 1.28188109546415 | 0.32261721058066 | 3.97338100207663 | 0.0000708595668116027 | 0.000998182659357042 |
| SBK3 | 2.46093678037357 | -0.646895045292381 | 0.177841446742928 | -3.6374819095319 | 0.000275316522730082 | 0.00291686030418676 |
| MANCR | 15.1015627797019 | -0.593229348698298 | 0.178171246093606 | -3.32954593799402 | 0.000869877133441631 | 0.00706421347657965 |
| Z97353.1 | 58.763087528593 | 0.727018328571561 | 0.205838339987791 | 3.53198693991937 | 0.000412449724898786 | 0.00397726173588962 |
| RNF2P1 | 1.47540060368256 | 0.723209802140927 | 0.267763903205625 | 2.70092343845745 | 0.00691472539804259 | 0.0337432141779279 |
| AC005392.2 | 23.6605096058876 | -0.947295144170296 | 0.216968935287789 | -4.36604043299469 | 0.0000126519069839903 | 0.000258938233389598 |
| LINC00689 | 3.24928285784366 | 0.906680931735682 | 0.198878006164934 | 4.55898039818316 | 0.000005140256825474 | 0.000125566449055881 |
| AC105450.1 | 0.942521388360242 | -1.76287217399565 | 0.281366909862524 | -6.26538555957766 | 3.7190404841696E-10 | 5.29243199453616E-08 |
| PTMAP4 | 120.624637922039 | -0.683143719152042 | 0.148211571919657 | -4.60924683750311 | 4.04130299945473E-06 | 0.000103010102826041 |
| FAM27C | 2.11217096516543 | -0.542276133267234 | 0.160634605394887 | -3.37583630833568 | 0.000735916893778857 | 0.00623051462322849 |
| LINC01249 | 0.529952739884727 | -1.73546589498686 | 0.672006772036559 | -2.58251250910377 | 0.00980838063907696 | 0.0436418128435353 |
| PTCHD3P2 | 7.15922855309175 | 0.767396808424486 | 0.257872137535506 | 2.97588105391504 | 0.00292148150555981 | 0.0175975893091879 |
| RPL7P21 | 1.31104215613343 | -0.705803600004187 | 0.213239183029658 | -3.30991513837315 | 0.000933242607379435 | 0.00742341794080344 |
| AL590428.1 | 1.64654334544512 | -1.01401672418368 | 0.20497688615777 | -4.94698081911143 | 7.53734003107456E-07 | 0.0000271913403796327 |
| EIF1P3 | 21.4036840108154 | 0.547675807347128 | 0.205661590009842 | 2.66299510434067 | 0.00774485409088654 | 0.0367954838055729 |
| AL354993.1 | 9.84806810652442 | 0.604040702737205 | 0.187590896386338 | 3.21998942578322 | 0.00128195325262696 | 0.00939783967583895 |
| AF064860.2 | 2.33012048667686 | -0.872421987675254 | 0.227323958265076 | -3.83779164472382 | 0.000124145747807916 | 0.00156136972478087 |
| ABCA9-AS1 | 6.83048519672962 | 0.746942913600747 | 0.267903543411752 | 2.78810389772538 | 0.00530175375038714 | 0.0277072183532014 |
| DLX6-AS1 | 49.3668102399665 | 1.10876283324945 | 0.282451142725204 | 3.9255030889649 | 0.0000865486593490249 | 0.001175613430524 |
| LINC01611 | 0.585420309221558 | -1.60296450599499 | 0.481682807814005 | -3.32784247224774 | 0.000875213406283465 | 0.00709204912355988 |
| PCAT7 | 60.1018886693659 | 0.50223808064461 | 0.0972391396951201 | 5.16497865179914 | 2.40466366326674E-07 | 0.0000106653530804995 |
| AKAIN1 | 1.39732471919901 | 0.878554448691292 | 0.27862992246207 | 3.15312311372764 | 0.0016153366518066 | 0.0112588762629671 |
| AL513523.2 | 1.53851060561298 | 0.592952805005 | 0.203339210170711 | 2.91607705423462 | 0.00354462947530368 | 0.0203606147222785 |
| AL353751.1 | 1.10210682413064 | 0.533893674005732 | 0.195523623674542 | 2.73058397738384 | 0.00632222235757466 | 0.0315381803111589 |
| AL161909.1 | 46.2446445330914 | 0.891542698275926 | 0.182651944776229 | 4.88110159116112 | 1.05494874790273E-06 | 0.0000354568804295106 |
| SLC25A6P2 | 38.6577908172946 | 0.858086111765321 | 0.24011669390278 | 3.5736212164938 | 0.000352078141541891 | 0.0035294356143417 |
| LINC00865 | 33.0754758523276 | 0.543770725791762 | 0.139256418224178 | 3.90481625713215 | 0.0000942970031779997 | 0.00125837205916946 |
| RPS29P8 | 3.40334652398871 | 0.82022426716791 | 0.285783245033065 | 2.8700922164734 | 0.00410352105370598 | 0.0227314810465522 |
| GRHL3-AS1 | 1.42985639549441 | -0.516222054408347 | 0.197969625049067 | -2.60758211912763 | 0.00911841958356131 | 0.0413980705557232 |
| AC233982.1 | 0.829074150497366 | 0.942480307722041 | 0.343946198793469 | 2.7401969000622 | 0.00614023872267648 | 0.0308738330121406 |
| AL021391.1 | 1.27879541277852 | -0.65662866773846 | 0.210614783962857 | -3.1176760500073 | 0.00182283042451231 | 0.0123050252435488 |
| RAD17P1 | 4.74962461845105 | -0.760847996957791 | 0.182893057400726 | -4.16007041366663 | 0.0000318149494129754 | 0.000535050892160108 |
| AL121895.1 | 7.72768632088567 | 0.807793056829805 | 0.147443427143649 | 5.47866441033551 | 4.28548275321667E-08 | 2.65398359690874E-06 |
| AL355994.2 | 2.33051937233127 | -0.828318505148891 | 0.212126334822972 | -3.90483579438713 | 0.0000942893856345507 | 0.00125837205916946 |
| KRT8P52 | 1.96375669544207 | -0.549971593151328 | 0.157476341626982 | -3.49240773229328 | 0.000478686942137192 | 0.00445574615609929 |
| MFAP1P1 | 1.92851076928344 | 0.696027710464418 | 0.183132992416308 | 3.80066803518489 | 0.000144306520915734 | 0.00175232341048075 |
| LINC01549 | 5.35574343523734 | 1.22789566794915 | 0.242083031559394 | 5.07220873780199 | 3.9322471218806E-07 | 0.0000158245737966223 |
| LINC00665 | 71.7367408854508 | -0.822401733928123 | 0.156350161921048 | -5.2599992467127 | 1.4405599176409E-07 | 7.06381301550542E-06 |
| AC002511.1 | 4.8376301414189 | 0.54009687695922 | 0.176758739587011 | 3.05555967541483 | 0.00224640894173779 | 0.0143999248283679 |
| ATP11A-AS1 | 1.2195113907852 | 1.05432146265906 | 0.318621100082039 | 3.30901331514952 | 0.000936253843810005 | 0.00743710238591311 |
| IGKV1D-35 | 1.4828878232341 | 1.0971813305288 | 0.353073903285965 | 3.10751182774378 | 0.00188669435567064 | 0.0126442550385446 |
| AC002480.1 | 2.67729166211937 | -0.830308481362919 | 0.169590251678116 | -4.89596821248225 | 0.0000009782287934347 | 0.0000334840607062877 |
| LINC01700 | 2.25430353755897 | 0.9368344146404 | 0.217668388013142 | 4.30395255457966 | 0.0000167777608850412 | 0.000323126109751138 |
| AC080129.2 | 14.0676181688932 | 0.546136105075858 | 0.122331246475297 | 4.46440399171558 | 8.02919414552174E-06 | 0.000179008207076359 |
| RPS11P5 | 236.873853404444 | 0.523749930192849 | 0.170764422841755 | 3.06709044821474 | 0.00216153440781342 | 0.0139710153973901 |
| AL121832.1 | 41.4366516569688 | 0.708658826945627 | 0.171551893937002 | 4.13087148548687 | 0.000036139058358042 | 0.000590984053598847 |
| AL136307.1 | 4.08311118711973 | 0.818264236986173 | 0.192613110080956 | 4.24822711518574 | 0.0000215468897949625 | 0.000396122001572885 |
| BX322234.2 | 0.892679309149853 | -1.3620921164914 | 0.398378997584487 | -3.41908615853308 | 0.000628318280375853 | 0.00549195502674576 |
| AL138889.1 | 1.80343856965819 | 0.928112020641821 | 0.241019838781227 | 3.85077023258764 | 0.000117746927974602 | 0.00150007343441015 |
| RPSAP63 | 2.86223518709981 | -0.7974557928996 | 0.247754834994859 | -3.21872948681767 | 0.00128759892991765 | 0.00943054827295357 |
| AC009362.1 | 27.6416489098834 | 0.693089490446508 | 0.236553521576856 | 2.92994788590083 | 0.00339018855247746 | 0.0196796885214288 |
| AK4P3 | 2.34482532905942 | -0.692260013834418 | 0.25204307763766 | -2.74659403591961 | 0.00602176205127055 | 0.0304199043079441 |
| BTBD18 | 1.80677407851234 | 0.570598500354572 | 0.181304578294141 | 3.14718197258569 | 0.00164852330404036 | 0.0114353694946521 |
| U40455.1 | 2.93002274312093 | 0.581254205593472 | 0.196717999787257 | 2.95475862006566 | 0.00312913766149899 | 0.0185408613883503 |
| AC017074.2 | 0.889641154563388 | 0.779049407688298 | 0.236669127749087 | 3.29172383021681 | 0.000995753425719003 | 0.00778764875184633 |
| TMEM238 | 845.570800867498 | 0.723970666831267 | 0.139646111721772 | 5.18432384478911 | 2.16799943037568E-07 | 0.0000097757716502117 |
| HNRNPLP1 | 5.80304288680594 | 0.53054334654382 | 0.192166671256939 | 2.76084995943156 | 0.0057651155248023 | 0.0295229167141651 |
| AP000943.1 | 0.929406031867185 | -0.99802196686137 | 0.318755720204962 | -3.1309931198086 | 0.00174216241991929 | 0.0119143958378202 |
| AC011294.1 | 0.838946731885596 | -1.82296921376664 | 0.396008930728016 | -4.60335379410591 | 4.15741087619456E-06 | 0.00010532737463765 |
| AC007690.1 | 2.26357391865636 | 0.66302039083967 | 0.2392070818197 | 2.77174231546964 | 0.00557571557529917 | 0.0287751667082029 |
| AL138789.1 | 6.91392466625362 | -1.22724961741975 | 0.191418144862393 | -6.41135467226475 | 0.0000000001442322101 | 0.0000000249917801561 |
| LINC02640 | 1.18958639353738 | 0.61930398874439 | 0.229836687317938 | 2.69453930950411 | 0.00704860117917959 | 0.0342167688538429 |
| AL353662.2 | 28.0706627220692 | 0.614184760264593 | 0.202292847454005 | 3.03611703525129 | 0.00239646324465536 | 0.0151297949457739 |
| CEACAMP1 | 0.965956123843574 | 0.700050782585847 | 0.247747501444536 | 2.82566233162424 | 0.00471829736030218 | 0.0253110844278514 |
| GAS6-AS1 | 494.602315319511 | 0.590167709287305 | 0.148030975518859 | 3.98678524693041 | 0.0000669745953926702 | 0.000957573501120853 |
| AP000695.2 | 7.0963255407206 | -0.775180328166995 | 0.128645402828416 | -6.02571340385096 | 0.0000000016836530008 | 1.8521290675242E-07 |
| AC139769.1 | 60.3261828802135 | 0.625959779842814 | 0.176801032242388 | 3.54047582134387 | 0.000399406230174761 | 0.00388057616197105 |
| AC093732.1 | 7.04930870535027 | -0.701097425408637 | 0.182533187526681 | -3.84093125698666 | 0.00012256843806128 | 0.00154688300856199 |
| LINC01694 | 51.464260206366 | 0.561685200581135 | 0.204234177230581 | 2.75020179383097 | 0.00595585743863288 | 0.0301974017268217 |
| AL591501.1 | 1.12925150337652 | -1.64066188596463 | 0.509583010499851 | -3.21961653383087 | 0.00128362176392897 | 0.00940553878819298 |
| AC138951.1 | 1.90308707348034 | 0.715619684289115 | 0.242798056519101 | 2.94738637758749 | 0.00320472500596092 | 0.0188983272173065 |
| LINC02574 | 0.687300621630573 | -0.86687641085688 | 0.259009519846519 | -3.34689015048776 | 0.000817235880643437 | 0.00674481794681091 |
| KDM3AP1 | 4.427737630149 | 0.743538566700299 | 0.212575464887435 | 3.49776286315086 | 0.000469178102551372 | 0.00438275254344217 |
| AL035446.1 | 14.6966393970456 | -1.14172344076119 | 0.228479947152082 | -4.99704002470393 | 5.82169888449126E-07 | 0.000021998785773464 |
| AC016683.1 | 0.875647106677792 | 0.592266990797934 | 0.221045435917706 | 2.67939027258827 | 0.0073756372475588 | 0.0354492757736219 |
| AL138720.1 | 1.05569397977469 | -0.830483593762829 | 0.241319230072984 | -3.44143147444843 | 0.000578645015695443 | 0.0051589033897326 |
| LINC01641 | 3.25802102307053 | 0.881972324653365 | 0.276800461212729 | 3.18631089265253 | 0.00144099657979348 | 0.0102947677037927 |
| LINC02765 | 6.57418051120063 | -0.600214847256468 | 0.230508324026076 | -2.60387493506989 | 0.00921763855473222 | 0.0417182661452639 |
| AC004231.1 | 9.70171014031553 | 0.779867482922069 | 0.226267490869915 | 3.44666164778585 | 0.000567558912397563 | 0.00508446427763175 |
| AC016894.1 | 2.07668820778458 | 0.644084448124825 | 0.213544893480493 | 3.01615476552551 | 0.0025600255679518 | 0.0159092586143384 |
| AC053503.3 | 9.64035023583781 | 1.11465895769552 | 0.279625494858629 | 3.98625654023092 | 0.0000671239350837587 | 0.000958070267635962 |
| LINC01934 | 3.87226083321637 | -0.558932351022613 | 0.171901578497628 | -3.25146724019363 | 0.00114811005903953 | 0.00869060583847897 |
| NUS1P2 | 1.28090854760493 | 0.8179046475369 | 0.28313368873078 | 2.88875778507097 | 0.00386766865665251 | 0.0217785107283673 |
| LINC01239 | 3.43218564314037 | -2.10978601185641 | 0.316961692106947 | -6.65628075693306 | 2.80843548646384E-11 | 6.28674387812247E-09 |
| AC008758.2 | 2.81993255067801 | 0.55707165943357 | 0.158939571918154 | 3.50492739291152 | 0.000456731825032388 | 0.00429166217834591 |
| LINC02539 | 0.754711986747868 | -0.609361375467512 | 0.232937940911429 | -2.61598163477891 | 0.00889713428860226 | 0.0407028679725632 |
| AC074085.2 | 87.1951994741537 | 0.865736743729809 | 0.237801362553517 | 3.64058781847803 | 0.000272016306847606 | 0.00288881612727808 |
| AC098934.1 | 21.3668586299107 | 0.523278819003792 | 0.104220466967616 | 5.02088346204003 | 5.14343596950482E-07 | 0.0000198393063082099 |
| AC016745.1 | 1.06487645485344 | 0.695197487993395 | 0.23582109065238 | 2.94798690850843 | 0.00319850609613486 | 0.0188716374147745 |
| AL023775.1 | 0.547380608845905 | -1.13572213896172 | 0.315354808925303 | -3.60141055984574 | 0.000316495307850024 | 0.00324073364516856 |
| AL137077.1 | 0.614414174458674 | 0.719824882094791 | 0.269239383648617 | 2.67354973236097 | 0.0075053146889191 | 0.0359279607539125 |
| LINC00940 | 9.13787337437375 | 0.622831092072271 | 0.200268471726629 | 3.10998075085154 | 0.00187099536458677 | 0.0125642222856447 |
| AL357632.1 | 3.33418931649675 | 0.640399991038242 | 0.239185881117063 | 2.67741552322153 | 0.00741925592215684 | 0.0356281959432466 |
| LINC00330 | 2.94560288724949 | -1.23250873712609 | 0.272155590450322 | -4.52869160279499 | 5.93500484434855E-06 | 0.000140894081626431 |
| KRT8P17 | 4.79721564961998 | -0.597705821822186 | 0.159068360260519 | -3.75754059979794 | 0.000171591533267749 | 0.00200361873447628 |
| HSP90AB6P | 0.908758019082786 | 0.86813394546781 | 0.2881165413172 | 3.01313469021566 | 0.00258564131677056 | 0.016021681844625 |
| TSPY26P | 144.010683357728 | 0.649931818081899 | 0.110796483015347 | 5.86599682944694 | 4.46442325992329E-09 | 4.07921428028291E-07 |
| AC145207.1 | 3.33740355864974 | 0.507593817288447 | 0.1772820762887 | 2.86319873906397 | 0.00419387419018181 | 0.0231285522209862 |
| FAUP1 | 99.4845252936052 | 0.672377614380082 | 0.195670785604786 | 3.43626981565937 | 0.000589783287220475 | 0.00523448319830868 |
| B3GALT1-AS1 | 0.515684507260932 | -1.06549100112161 | 0.317617039297101 | -3.35464055542985 | 0.00079468170115813 | 0.00660923786374787 |
| RPEL1 | 3.08883756606432 | 0.566063073506105 | 0.164182487495423 | 3.44776767693867 | 0.000565240004645234 | 0.00506975884695129 |
| LINC02154 | 6.22933739818678 | -0.719984196308343 | 0.189945078901424 | -3.79048617880748 | 0.000150352699284201 | 0.00181323294967986 |
| AL135924.2 | 2.18347378499294 | -0.877176135512854 | 0.224704844529229 | -3.90368145978603 | 0.0000947404573573884 | 0.00126328164870247 |
| AGAP1-IT1 | 15.4361795293632 | -0.536829409930209 | 0.135068993602902 | -3.97448293357741 | 0.0000705323192802832 | 0.000995507518048904 |
| AC073150.1 | 1.20068487743956 | -0.612411725021292 | 0.202544780264462 | -3.0235868049607 | 0.00249797354326144 | 0.015634892613466 |
| GNAS-AS1 | 4.23821078443411 | 0.596376232140339 | 0.223042222538591 | 2.67382662059491 | 0.00749912107733198 | 0.035910741607431 |
| RNF148 | 3.25513895120039 | -0.690480053484223 | 0.154425304179947 | -4.47128828497969 | 7.77498061902562E-06 | 0.00017433823845196 |
| CHN2-AS1 | 12.7992109275767 | 0.822403165068509 | 0.193362061963646 | 4.25317746778647 | 0.000021075841580636 | 0.000388972568509729 |
| SEPTIN14P12 | 0.991929806284572 | -0.707462549787136 | 0.212460834077226 | -3.32984925367461 | 0.000868930137281473 | 0.00705823698104615 |
| AL390860.1 | 0.516012103460103 | 1.05688859956131 | 0.330396805341229 | 3.19884630382479 | 0.00137978706841631 | 0.00995101124476563 |
| MYL6P2 | 7.13360451266463 | 0.904036882826329 | 0.277583900615919 | 3.25680589119325 | 0.00112673454807389 | 0.00856953758396339 |
| LINC00941 | 42.9484889100116 | -1.68677727828871 | 0.199392114825501 | -8.45959871464776 | 2.68299151414684E-17 | 5.27791777741756E-14 |
| AF064858.1 | 58.8446565832935 | 0.659832089117751 | 0.161840865574411 | 4.07704251195057 | 0.0000456121558244106 | 0.000713317400020835 |
| TSPEAR-AS1 | 20.0769883003514 | 0.656134229148911 | 0.175447351720496 | 3.73977847322651 | 0.000184182502238978 | 0.00212247802890279 |
| THRAP3P3 | 0.971848894397901 | 1.06697983634016 | 0.319513127932503 | 3.33939279191546 | 0.000839617522898458 | 0.00688704665213888 |
| LINC01553 | 1.33221149332847 | 0.590992111378681 | 0.190701404185192 | 3.0990443615442 | 0.00194145947901811 | 0.0128975542108311 |
| AC019117.1 | 6.11510092414486 | -1.32269754879127 | 0.202601353280496 | -6.52857213130279 | 6.63996334823317E-11 | 1.25692894648605E-08 |
| RPL17P36 | 17.1487297042733 | -0.537951710714298 | 0.111489088029599 | -4.82515123427575 | 0.0000013989707148287 | 0.0000444246380503054 |
| VEZF1P1 | 1.05768553142684 | 0.7964463441006 | 0.252279961249882 | 3.15699407973083 | 0.0015940458267432 | 0.0111485859195832 |
| MYO16-AS1 | 2.94118207761623 | -1.42290999208346 | 0.2871028529947 | -4.95609840599434 | 7.19227991126294E-07 | 0.0000261155510089528 |
| MTND4P14 | 0.345159271086305 | -0.890928096927983 | 0.345728465362812 | -2.57695904788468 | 0.00996737587297514 | NA |
| DIAPH2-AS1 | 7.57810220437628 | -1.15858986101977 | 0.174646056286345 | -6.63393085223852 | 0.0000000000326862998 | 7.09802102612377E-09 |
| RPL26P30 | 7.07122755679625 | 0.504253265586037 | 0.140404770514146 | 3.59142544615486 | 0.000328874260090036 | 0.00334495529377463 |
| GUSBP5 | 20.5698316825149 | 0.750859700316491 | 0.138801601163302 | 5.40958961585101 | 6.3169334839742E-08 | 3.63598777230749E-06 |
| TRHDE-AS1 | 1.51111418282295 | -0.759415037623493 | 0.292952869334032 | -2.59227717874847 | 0.00953429208504628 | 0.0427693891224839 |
| RRAS2P1 | 3.3684575023175 | -0.644247259154669 | 0.238625598453277 | -2.69982459271154 | 0.0069376042877338 | 0.0338153858898694 |
| LINC00479 | 3.58642871723064 | -0.530948986966042 | 0.172502011884293 | -3.07792924364372 | 0.00208444400996474 | 0.0135858651693693 |
| AC092573.1 | 7.32967054508331 | 0.754617320238924 | 0.142161105231851 | 5.30818411272351 | 1.10722771233756E-07 | 5.73187448235183E-06 |
| VLDLR-AS1 | 8.26842826196634 | 0.532065304214442 | 0.164753798804741 | 3.22945697200598 | 0.00124025542898076 | 0.00918233829000984 |
| AL589986.2 | 0.832741845460959 | 0.97560024879339 | 0.372766079782403 | 2.61719158932831 | 0.00886565643210777 | 0.0405824021530214 |
| UBE2L5 | 2.74080257100972 | -0.501036057789609 | 0.125972346565119 | -3.97734956481585 | 0.00006968768238858 | 0.000987079828224859 |
| AC123023.1 | 29.6900538315539 | 0.729569965855916 | 0.165321258673766 | 4.41304386204561 | 0.0000101927336127922 | 0.000217527375544988 |
| LINC02195 | 5.02271591934168 | -0.7072151017534 | 0.173204450458373 | -4.08312315233129 | 0.0000444344597041012 | 0.000700272008211381 |
| RPS12P4 | 3.33909462306109 | 0.527156741122884 | 0.187947462826585 | 2.80480903117739 | 0.00503463991653329 | 0.0266237236066898 |
| ZNF853 | 157.620796323995 | 0.609767155870678 | 0.128935542156755 | 4.72924025191862 | 2.25361574635111E-06 | 0.000065026244857182 |
| LINC01923 | 2.04987439170673 | 1.55087654407779 | 0.334419883680776 | 4.63751295828509 | 3.52626509230393E-06 | 0.0000920355518480045 |
| FRGCA | 6.21469197121769 | 0.626883756437602 | 0.188811943312562 | 3.32014884990538 | 0.000899694639946854 | 0.00723764147366055 |
| SPA17P1 | 0.565119564036987 | 0.926820432698348 | 0.267849957882702 | 3.4602224320835 | 0.000539729351355425 | 0.0048956050327224 |
| BCYRN1 | 107.564823601293 | 0.660647259936267 | 0.106198762854049 | 6.22085645992134 | 4.94448360218834E-10 | 6.64069962346918E-08 |
| AC024560.1 | 7.6245701401509 | 0.766448115804295 | 0.186317375399031 | 4.11366956067738 | 0.0000389418761174913 | 0.000625992963348886 |
| AC007750.1 | 5.85261795254725 | -0.505900540675877 | 0.15774397160895 | -3.20709904483712 | 0.00134080824090355 | 0.00971804487705168 |
| LINC01474 | 0.941949980780772 | -0.626294649034317 | 0.232656086128822 | -2.691933228377 | 0.00710391664302281 | 0.0344253268187174 |
| LINC02827 | 4.7236182386804 | -0.570929479000624 | 0.184566558927234 | -3.09335278459471 | 0.00197908716345512 | 0.0130593198343067 |
| AP003774.2 | 281.502426730994 | 1.06902125966476 | 0.181778556121491 | 5.88089861903339 | 4.08045049386206E-09 | 3.8010703458422E-07 |
| ZDHHC20-IT1 | 7.03568835571448 | 0.774174960907581 | 0.148322911910661 | 5.21952374676875 | 1.79383814640849E-07 | 8.40189569918667E-06 |
| LAPTM4BP1 | 1.41341701219983 | 1.4599445759222 | 0.315031769140531 | 4.6342772981443 | 3.58186598704517E-06 | 0.0000932903133479475 |
| AC005550.2 | 66.1043343301093 | -0.769084332321805 | 0.165907905360998 | -4.63560992255528 | 3.55886539803661E-06 | 0.0000927635047865475 |
| RPL38P2 | 18.7133678295901 | 0.782942710598864 | 0.251744631511686 | 3.11006715772812 | 0.00187044811406292 | 0.0125630700603519 |
| DDX10P1 | 1.41470852204883 | -0.571539531363814 | 0.201311206433321 | -2.83908452733415 | 0.00452431703282462 | 0.0245580604141732 |
| TRBV30 | 5.92814275967392 | -0.763630239747137 | 0.194973522743025 | -3.91658430849403 | 0.0000898124162102948 | 0.00121255826520173 |
| BX276092.7 | 0.781795373398385 | -1.61302507268689 | 0.535482277307824 | -3.01228470304656 | 0.00259289290337264 | 0.0160517446269137 |
| RSL24D1P8 | 0.496683875777692 | 0.671121425104782 | 0.256153636136855 | 2.6199956995583 | 0.00879308758054076 | 0.0403261704427378 |
| AL513366.1 | 2.91303756616715 | 0.876069536203853 | 0.184079037507161 | 4.75920315570843 | 1.94358735733412E-06 | 0.0000579816667296768 |
| TUSC8 | 129.484657016489 | 0.558704207307403 | 0.166837071815377 | 3.34880132591675 | 0.000811619719291135 | 0.0067150387561935 |
| LINC01522 | 0.544500528622793 | -0.969956026730584 | 0.324135223439838 | -2.99244252579854 | 0.00276754772701092 | 0.0169138031956687 |
| UNC5B-AS1 | 16.4560997162944 | -0.812335811910852 | 0.197295813774685 | -4.11734945799992 | 0.000038325471381128 | 0.000617975127255391 |
| AC007384.1 | 9.10606415854051 | -0.667347862399803 | 0.140342719805297 | -4.75512989434466 | 1.98318863895042E-06 | 0.0000589003503230728 |
| SHISA9 | 76.4589083095192 | 1.685201103392 | 0.303169802772797 | 5.55860474222404 | 0.0000000271939745827 | 1.86579559559714E-06 |
| AP001056.1 | 0.637759741426476 | -0.801422481635279 | 0.266285701020113 | -3.00963393289655 | 0.00261562728757435 | 0.0161583590902984 |
| RPS15AP38 | 7.90783033632898 | 0.735685546569089 | 0.152665181254564 | 4.81894784733107 | 1.44317268763892E-06 | 0.0000455307368113403 |
| RPL30P4 | 19.5144132492829 | 0.762516317121462 | 0.21089427976037 | 3.61563299861844 | 0.000299614599708691 | 0.00311074555835394 |
| AC078991.1 | 30.1280789726828 | 0.848195695452798 | 0.261373607738067 | 3.24514668023717 | 0.00117390139387332 | 0.00882590162183264 |
| UQCRBP1 | 7.96998504719585 | 0.550829167967664 | 0.149656678657077 | 3.68061868611847 | 0.000232668728698668 | 0.00254028978946812 |
| AL162615.1 | 3.375926740913 | 0.589854380458428 | 0.143366360797547 | 4.11431508184393 | 0.0000388330712802005 | 0.000625152661273322 |
| AC002066.1 | 0.848945895592898 | -0.782980371250184 | 0.26935898525721 | -2.90682848579386 | 0.00365113328250757 | 0.0208186187951608 |
| CDK6-AS1 | 1.87034340855069 | -0.633305481417919 | 0.192030757424846 | -3.29793773617631 | 0.000973977293551884 | 0.00765673452067751 |
| Z94160.1 | 1.38039469167127 | -1.25598900916272 | 0.374311713694495 | -3.35546274191097 | 0.000792323284737481 | 0.00659618503564621 |
| LINC00398 | 1.99911748482999 | 0.576958830254903 | 0.170896239462145 | 3.37607680584864 | 0.000735273901727672 | 0.00622822437223323 |
| AC087650.1 | 4.73035072147318 | 0.568843070408158 | 0.190024460342916 | 2.99352551446077 | 0.00275774449486631 | 0.0168606424757021 |
| IDI1P1 | 1.06697825735501 | 0.76503354390862 | 0.29691977151251 | 2.57656652506344 | 0.00997870016475582 | 0.0442172639339823 |
| AC000111.2 | 1.87342103626996 | 0.536842912270842 | 0.201136214695147 | 2.66905148376443 | 0.00760657897066791 | 0.0363087659059486 |
| OR2I1P | 509.174411986936 | -0.65319827003088 | 0.169521720813285 | -3.8531833377879 | 0.000116592021819217 | 0.00148819480674742 |
| ACTG1P19 | 5.15251500514351 | 0.605047253406303 | 0.227832803357622 | 2.65566347114897 | 0.00791525597916695 | 0.0373503584669537 |
| LINC02257 | 7.06975729062531 | -0.673088221783707 | 0.187633625148278 | -3.58724733507546 | 0.00033418729892459 | 0.00338355787182444 |
| LINC02806 | 1.55808327347092 | -0.752141021523531 | 0.257450703342124 | -2.92149530671127 | 0.00348355524084267 | 0.0201010825768408 |
| LINC01589 | 3.21434493136274 | -0.502987934063527 | 0.15533034635082 | -3.23818201581492 | 0.00120294036316262 | 0.00897762366098735 |
| AC104978.1 | 0.943620781377945 | 0.75687565618797 | 0.284936080412974 | 2.65629980973623 | 0.0079003342228432 | 0.0373220761520444 |
| MLLT10P1 | 2.12241718892899 | 0.501096714781854 | 0.160166750424366 | 3.12859387765678 | 0.00175644892342784 | 0.0119900316181412 |
| RPS2P35 | 17.5630704028759 | 0.816662788290511 | 0.17904290639715 | 4.56126860719632 | 5.08454874515272E-06 | 0.00012438732928705 |
| AC078993.1 | 112.225867204133 | 1.24210381553496 | 0.259024551371478 | 4.79531306572404 | 1.62420970509631E-06 | 0.0000499235486744769 |
| AHCYP2 | 3.54440275211718 | 0.647060852989376 | 0.217748561069033 | 2.97159645883602 | 0.00296255795209659 | 0.0177998316625968 |
| CD81-AS1 | 2.22887577669993 | 0.536742019166621 | 0.140945162850861 | 3.80816204196073 | 0.000140003522154524 | 0.00171376200142445 |
| MPC1L | 0.779065845846003 | 1.12285327881899 | 0.246437545368239 | 4.55634013535221 | 5.20526219431674E-06 | 0.00012687636902503 |
| TARDBPP2 | 4.59494518770253 | 0.80957239077738 | 0.217085155691258 | 3.7292848891463 | 0.00019202395343047 | 0.00218796083496483 |
| HMGN2P17 | 20.8580614380401 | -0.552972424114122 | 0.186959025556417 | -2.95771986652367 | 0.00309923636613484 | 0.0183973757600316 |
| AL121748.1 | 0.783978448378197 | -0.56027031048551 | 0.214711089618906 | -2.60941487223572 | 0.0090697209094204 | 0.0412441673446882 |
| LINC00707 | 1.28902228004536 | -1.0034294038175 | 0.2365231423953 | -4.24241532416509 | 0.0000221126983742814 | 0.000403653307332269 |
| IFNWP19 | 5.19492437613485 | -1.09067141304563 | 0.272694041953554 | -3.99961585237494 | 0.0000634453838279472 | 0.000915727460498149 |
| RNU1-88P | 0.581201682744292 | 2.09722402609602 | 0.622438627783148 | 3.36936676562862 | 0.000753411049777697 | 0.00634005690237755 |
| SCARNA7 | 5.25967175129108 | 0.790950859533979 | 0.203507850236206 | 3.88658648114038 | 0.000101663718592234 | 0.0013353645236298 |
| SCARNA10 | 4.50151874521944 | 1.68001724096848 | 0.236891407514381 | 7.09192983652854 | 1.32254566499446E-12 | 5.02597410553917E-10 |
| AC068299.1 | 1.96051643233069 | -0.653838794925971 | 0.239864195200141 | -2.72587075524304 | 0.00641321051769643 | 0.0318725793034335 |
| RPS20P22 | 1.88922821384774 | -0.952017865055756 | 0.200363115729313 | -4.75146267111315 | 2.01950445291521E-06 | 0.0000598195464255009 |
| RPL34P31 | 34.0579679281548 | 0.658756211785193 | 0.248067316445682 | 2.65555423110097 | 0.00791782012958119 | 0.0373519171636979 |
| RN7SL546P | 1.63232216356674 | 0.766519684955068 | 0.268824460964112 | 2.85137625573961 | 0.0043530425981838 | 0.0238072393581042 |
| ATP6V1B1-AS1 | 0.676980694400492 | -1.56853152282167 | 0.378883638900441 | -4.13987663171129 | 0.0000347492647620873 | 0.00057217376276402 |
| RN7SL477P | 0.765807989039583 | 0.959884761522342 | 0.265175691044119 | 3.61980676940195 | 0.000294823097323707 | 0.00307627894561604 |
| AC146507.1 | 1.48034499268026 | 0.801694927051617 | 0.223464266457287 | 3.58757549813832 | 0.000333767105865016 | 0.00338093261016175 |
| SIDT1-AS1 | 0.983474489388977 | -0.649597645978286 | 0.228848138538893 | -2.8385533311554 | 0.00453185455631373 | 0.0245830137992285 |
| IGKV2D-30 | 4.27136358472236 | -0.803567823241023 | 0.290753375726344 | -2.76374374410475 | 0.00571423961574858 | 0.0293361377386957 |
| PRR20G | 0.547208658258961 | -1.30725069443754 | 0.447805900728094 | -2.91923508000243 | 0.00350891501488354 | 0.0202214606113623 |
| AC005165.2 | 10.044778826218 | 0.634658744452065 | 0.238616328819672 | 2.65974565777388 | 0.00781996795456536 | 0.0370575837234766 |
| AC092104.1 | 0.456765673468043 | 0.97657971242151 | 0.358702312034178 | 2.72253531593758 | 0.00647831088620259 | 0.0320987817008022 |
| RN7SL825P | 0.657080155551963 | 0.869580680251549 | 0.292167379675627 | 2.97630995361968 | 0.00291739839297216 | 0.0175822016683682 |
| AC092902.1 | 1.06332397656823 | 0.552683094685647 | 0.203833372829848 | 2.71144556464266 | 0.00669905396325139 | 0.0329529308098408 |
| AC112206.1 | 1.88665952988281 | 1.12982740850362 | 0.332798444870271 | 3.39492995210371 | 0.000686461612897492 | 0.00589993555860137 |
| RPS27P25 | 1.14498494129937 | 0.676850253114952 | 0.225204477658119 | 3.00549198734171 | 0.00265151582009483 | 0.0163209998261755 |
| RN7SL75P | 1.64680362536551 | 0.507352205254642 | 0.152893280653859 | 3.3183420689576 | 0.000905535142571035 | 0.00726906054677402 |
| RPL23AP75 | 3.44543574921764 | 0.514896744033077 | 0.186479176245244 | 2.76114874808286 | 0.00575984364800406 | 0.0295068460901581 |
| AC080013.1 | 17.2706126449948 | -0.500849363863065 | 0.103051374511197 | -4.86019100898695 | 1.17272549010095E-06 | 0.0000384492998430942 |
| AL512306.2 | 8.15693950698537 | -0.628756059348069 | 0.155495884631418 | -4.04355434125123 | 0.0000526469470096364 | 0.000792001440349195 |
| AC010343.1 | 269.469969764012 | 0.596713771907081 | 0.157322570846525 | 3.79293173698008 | 0.00014887909360937 | 0.00180092774766861 |
| KIR3DL2 | 1.70906096013501 | -0.796461956841758 | 0.281422753752788 | -2.83012637116542 | 0.00465296218343236 | 0.0250610986210895 |
| SAMMSON | 0.6250488601357 | -0.904139209733967 | 0.347126563360498 | -2.60463849548443 | 0.00919712420725607 | 0.0416523984020414 |
| RN7SL472P | 0.941494420900788 | -1.36343726959899 | 0.367116335430143 | -3.71391065451085 | 0.000204080870480844 | 0.00229639046790726 |
| PHBP8 | 5.27795393407742 | 0.797987171485567 | 0.195901586045131 | 4.07340842713611 | 0.0000463300726989447 | 0.000721308329235619 |
| LINC00973 | 2.29827694189728 | -1.72688549887905 | 0.341103645241384 | -5.06264158407633 | 4.13486772482129E-07 | 0.0000165227269855392 |
| RPL29P2 | 8.55506433905167 | 0.677408728331589 | 0.211922196184075 | 3.19649730197773 | 0.00139107127453838 | 0.0100108038655288 |
| AC034238.1 | 1.46585956267203 | -0.920548784745 | 0.221951166110701 | -4.14752848960418 | 0.0000336083659312771 | 0.000556952910542007 |
| RPS6P25 | 82.7862678143235 | 0.526990198562889 | 0.175396049838867 | 3.00457278853785 | 0.00265954113997778 | 0.0163643743888016 |
| PNMA2 | 151.350062354664 | -1.0757142038546 | 0.159337784028141 | -6.75115579406212 | 1.46671998222443E-11 | 3.58029559456565E-09 |
| KRBOX1 | 4.5384463257159 | 0.67520273628601 | 0.218678812402701 | 3.08764588972896 | 0.00201748716590476 | 0.0132655929614996 |
| AC025271.1 | 2.58315543494656 | 1.01605720109446 | 0.308217563022466 | 3.29655841520102 | 0.000978772580759064 | 0.00767998888919395 |
| LINC00886 | 40.9785330253559 | 0.509052010777988 | 0.148423599930006 | 3.42972418818873 | 0.000604195071692546 | 0.00533383487409218 |
| AC022034.2 | 2.71367565167757 | 0.527340807459137 | 0.19353529645068 | 2.72477846227663 | 0.00643446454207373 | 0.0319354946892297 |
| AC021074.1 | 135.84694578395 | 1.05464113794402 | 0.207961829653786 | 5.0713207308273 | 3.95064307440407E-07 | 0.0000158794958767093 |
| AC090311.1 | 0.54860958252896 | 1.06508740865252 | 0.396853724454004 | 2.68382868301885 | 0.0072784392010626 | 0.0350930743601407 |
| HLA-DOB | 52.5765549947536 | -0.643338498015673 | 0.123565030401117 | -5.20647707468097 | 0.0000001924594589753 | 0.0000088653295138459 |
| UGT1A9 | 1.11764862429356 | 0.758489227643861 | 0.260305021826791 | 2.9138478478858 | 0.00357003859258822 | 0.0204575512228256 |
| TDGF1 | 923.238002854671 | 0.796154068985647 | 0.146533101698914 | 5.43327111591162 | 5.53302422415378E-08 | 3.26917660961397E-06 |
| AL136126.1 | 33.2526784372492 | 0.599518227373864 | 0.175000751709167 | 3.4258037266617 | 0.000612983161027041 | 0.00538607011851453 |
| ENPP7P4 | 7.82871416165777 | 0.621212234575039 | 0.136020626923164 | 4.56704434192875 | 4.94649403143868E-06 | 0.000121632833381892 |
| AC106712.1 | 0.612407048869538 | -0.991685185076305 | 0.341559657306493 | -2.9034025648598 | 0.00369131812123134 | 0.0209797859636673 |
| RPL34P33 | 8.58712849169361 | 0.759924239699253 | 0.224458505945456 | 3.38558895996535 | 0.000710256838577583 | 0.00606392882198405 |
| CDRT1 | 5.77131398552139 | -0.829287462969869 | 0.150633458826689 | -5.50533373813055 | 3.68469563347925E-08 | 2.38805409641111E-06 |
| PTPRG-AS1 | 9.97561146492586 | 0.565703358180984 | 0.168935977697212 | 3.34862570952712 | 0.000812134285812127 | 0.00671723905565257 |
| CA15P1 | 1.32275341139272 | -0.698728833087526 | 0.203878082440096 | -3.4271895474239 | 0.000609863208593022 | 0.00536913044517503 |
| KRTAP5-4 | 4.60443407877016 | 0.606730508009331 | 0.180240304401025 | 3.36623104374807 | 0.000762028531037619 | 0.00639010986333 |
| UGT1A1 | 15.8763787756899 | -1.04841520816378 | 0.243827041955182 | -4.29983155172955 | 0.0000170927980192889 | 0.000327484381308776 |
| SPRR2A | 19.4497331549535 | -1.27494165684372 | 0.312727147110987 | -4.07684995889162 | 0.0000456499286669783 | 0.00071337612826219 |
| SCG5-AS1 | 0.730225392023979 | -1.00399467634663 | 0.235041781461961 | -4.27155831657581 | 0.0000194111749883657 | 0.000363668635272228 |
| CECR3 | 3.43618236852112 | 0.965016573097711 | 0.184414952025511 | 5.23285429136037 | 1.66912312879516E-07 | 7.91756250683231E-06 |
| HOGA1 | 24.6451714888147 | 0.506580317311653 | 0.171906329252712 | 2.94683924387071 | 0.00321040054197647 | 0.0189217862045783 |
| AC084864.1 | 1.99582394159881 | -0.52295368089411 | 0.168606277693791 | -3.10162639284318 | 0.00192460691171383 | 0.0128263264486845 |
| AMACR | 659.450862212497 | 0.512296898642558 | 0.0983520353357147 | 5.20880830675119 | 0.0000001900573410042 | 8.77639122071993E-06 |
| LASTR | 6.13145172552799 | -0.501861061816889 | 0.155662641800967 | -3.22403022337613 | 0.00126400058442179 | 0.00930048570830222 |
| ABCF2P1 | 2.92363368900858 | 0.812952767863731 | 0.247280157306738 | 3.28757784982848 | 0.00101053245033668 | 0.00786163426293773 |
| AC093789.1 | 7.58718569853038 | 0.800051726225921 | 0.17696667953328 | 4.52091731808453 | 6.15722353688916E-06 | 0.000144396822945755 |
| ARHGDIG | 84.5867258947646 | 0.864783463075549 | 0.204135837501163 | 4.23631378821773 | 0.0000227219187982669 | 0.000411851711897909 |
| PEG10 | 153.737196862194 | -1.66542097109994 | 0.213090547146862 | -7.8155553749277 | 5.4721213904991E-15 | 4.69227393695053E-12 |
| RPS3AP48 | 0.871491613785641 | 0.840834992544586 | 0.274178705284036 | 3.06674069262061 | 0.00216406501113223 | 0.0139819671758663 |
| AC073861.1 | 587.056088090733 | 0.58321389484883 | 0.138820673165607 | 4.20120347747543 | 0.0000265499827106128 | 0.000468540644753727 |
| RN7SL272P | 3.55589013436954 | 0.990445374586706 | 0.26019709955501 | 3.80651965867633 | 0.000140936094710048 | 0.00171888580572335 |
| AC011506.1 | 7.14632060098243 | 0.684426434960267 | 0.187802969510724 | 3.64438558529387 | 0.000268031336680367 | 0.00285917191746885 |
| AC127024.1 | 4.13285572020449 | 0.739222950768974 | 0.222491129591446 | 3.32248279797216 | 0.000892201723617382 | 0.00718964097378614 |
| AC012501.3 | 27.4642057364606 | 0.784664980285763 | 0.296310398905758 | 2.64811826781458 | 0.00809412032705609 | 0.037921486687785 |
| CSAG4 | 0.939695996380564 | -2.7668991786963 | 0.757805445268882 | -3.65119991677356 | 0.00026101795688358 | 0.00279953897180907 |
| RPS24P16 | 6.9619620319486 | 0.913888275764463 | 0.280621148265794 | 3.25666216324816 | 0.00112730517059949 | 0.00857192803892406 |
| RPS29P11 | 1.02156929260186 | 0.783757126283507 | 0.284158831459576 | 2.75816564369214 | 0.00581267352907907 | 0.0296820015512998 |
| CCT4P1 | 0.75166260642394 | 1.03603912865054 | 0.373431955463094 | 2.77437191299215 | 0.00553084089261827 | 0.0286221729636286 |
| CCDC169 | 4.59610648384427 | 0.731586733771814 | 0.258315684398851 | 2.83214213443661 | 0.00462372905932222 | 0.024927736127979 |
| SOX2-OT | 6.75019619348837 | -0.587292089418004 | 0.186407158478589 | -3.1505876395056 | 0.00162942363018302 | 0.0113357988434742 |
| RN7SL749P | 1.0353269838424 | -0.570279382987952 | 0.19888869242201 | -2.86732933905519 | 0.00413951994505152 | 0.0228854068445054 |
| UBA52P3 | 10.5821788202568 | 0.619419789129775 | 0.233694839275194 | 2.65054971282596 | 0.00803608999453404 | 0.0377633236806742 |
| AC092894.1 | 20.5457265531608 | 0.533085958829532 | 0.188726609971028 | 2.82464650274472 | 0.00473328042278566 | 0.0253629809163272 |
| AC079760.2 | 1.32589920813854 | -0.968009879514027 | 0.230547934259452 | -4.19873586212512 | 0.0000268409186351989 | 0.000472428421578065 |
| AC011005.4 | 1.35407173000177 | 0.652726398530023 | 0.210454436712804 | 3.10150932774472 | 0.00192536806328558 | 0.0128263264486845 |
| RPL31P61 | 19.4105492451889 | 0.848918687695433 | 0.254986503703771 | 3.32926909998993 | 0.000870742297591101 | 0.00706774420173527 |
| AC106820.1 | 18.7395430685017 | 0.510160238830412 | 0.18927281972813 | 2.69536978190108 | 0.00703105539826154 | 0.0341487088856105 |
| C4orf48 | 629.433173597982 | 0.681514818273479 | 0.151298646348512 | 4.50443434043442 | 6.65499697184179E-06 | 0.000153381398161498 |
| RPS26P28 | 11.5398058488561 | 0.537027756148774 | 0.182051437910924 | 2.94986824773962 | 0.0031790946672364 | 0.0187902587242346 |
| LINC01214 | 3.95273678377932 | 0.778505870476951 | 0.179187966427333 | 4.3446325442432 | 0.0000139508989435808 | 0.000280713575494122 |
| RN7SL838P | 1.24983300837602 | 0.767933376303085 | 0.21820930356243 | 3.51925130489854 | 0.000432766563779579 | 0.00411972087273461 |
| AC109454.1 | 3.52271295460838 | 0.506428489943658 | 0.17110825455857 | 2.95969642873254 | 0.00307942318728738 | 0.0183339986165684 |
| LEFTY1 | 2365.1523992551 | 0.976317433295092 | 0.208135895568503 | 4.69076912768159 | 2.72179956614181E-06 | 0.0000753496863335385 |
| HLA-L | 894.343969005532 | 0.677226926262215 | 0.177563509572212 | 3.81399831470891 | 0.000136736400325002 | 0.00168239098589725 |
| KIR2DL3 | 1.72963081199838 | -0.76441591502053 | 0.271322897223806 | -2.81736603450017 | 0.0048419309206703 | 0.0258292955573546 |
| CFAP61-AS1 | 1.04133648373291 | -0.904745463376066 | 0.296907156181627 | -3.04723360329721 | 0.00230958143779572 | 0.0146989892852195 |
| AC011495.1 | 65.6575011928351 | 0.528932072536129 | 0.161366328657775 | 3.27783421074098 | 0.0010460681504541 | 0.00806050946716265 |
| PARAL1 | 1.80231290242174 | -0.789816057318619 | 0.296203904388947 | -2.6664606563778 | 0.00766545780585671 | 0.0365012444743643 |
| RPL23AP65 | 50.4002071202477 | 0.806790593403079 | 0.165898270425204 | 4.86316458474968 | 1.15523710007351E-06 | 0.000038100038560807 |
| RPS3AP19 | 1.52039447650087 | 0.65846552296945 | 0.204803196118283 | 3.21511351116395 | 0.00130392949913792 | 0.00951586079809355 |
| RPS18P6 | 1.85058269061616 | 1.02385992474266 | 0.291028209306496 | 3.51807794571689 | 0.000434684663442544 | 0.00413562575102292 |
| GSTA2 | 2.98528937612025 | 0.685672946951119 | 0.212823918146247 | 3.2217851871327 | 0.00127394608777324 | 0.00935674808900652 |
| SPRR2F | 1.0111979679002 | -1.58976699323479 | 0.525164041416394 | -3.02718173343914 | 0.00246845503620132 | 0.0154804137401773 |
| UGT1A7 | 1.48708133531461 | 0.597095357046742 | 0.206436589067775 | 2.8923911199226 | 0.00382321671877117 | 0.0215790740099824 |
| LINC01322 | 1.78778324098653 | -1.83310440005396 | 0.363174018856175 | -5.04745467703709 | 0.0000004477349389463 | 0.0000175947730061601 |
| TMEM225B | 7.13501787323343 | -0.540126143854437 | 0.157325469925902 | -3.43317673933424 | 0.000596553093040596 | 0.00528474928144731 |
| LRRC77P | 2.13923332820175 | 0.997966111337677 | 0.263280370650215 | 3.79050708897527 | 0.000150340041566819 | 0.00181323294967986 |
| IFITM10 | 131.5793257081 | -0.69754397543541 | 0.105699481503768 | -6.59931312350426 | 0.0000000000413067062 | 8.6879174134839E-09 |
| AC107302.1 | 2.49780841871397 | -2.84793128171434 | 0.692627391198325 | -4.11177975041832 | 0.0000392620761683318 | 0.000630341983303577 |
| LY6G6D | 310.705945670408 | 0.903251412148167 | 0.263531328831325 | 3.4274915857397 | 0.000609185182230226 | 0.00536679949002719 |
| ETV5 | 623.477439486998 | -0.698988097930286 | 0.106487397663234 | -6.56404526046204 | 5.23672951091845E-11 | 1.04242088276271E-08 |
| LILRA6 | 37.766084667094 | -0.51769005846822 | 0.125455015361673 | -4.12649950243739 | 0.000036832680067034 | 0.000599934681744457 |
| LINC01213 | 3.82063705931879 | 0.665949982503961 | 0.241097257055065 | 2.76216324747262 | 0.00574197607395837 | 0.0294423740977178 |
| KCNK15-AS1 | 7.85132949740875 | 0.801949754298085 | 0.224937706222952 | 3.56520819814538 | 0.000363567464709089 | 0.00361427561082085 |
| LINC01391 | 0.414566477600019 | -1.07016452487292 | 0.414799568566414 | -2.57995573276875 | 0.00988129808300216 | 0.0438670344473328 |
| AC108676.1 | 21.1918394376508 | 0.641696822766514 | 0.22056598868351 | 2.90931900514945 | 0.00362217052837503 | 0.0206922833634981 |
| FCGR2C | 29.2971044589864 | -0.526880156255062 | 0.138830172299297 | -3.79514155697501 | 0.000147559240203299 | 0.00178676439642877 |
| NT5C3AP2 | 0.881834863967137 | -0.63160403713749 | 0.240924299061899 | -2.62158711095894 | 0.00875213920883531 | 0.0401769443269554 |
| ASLP1 | 7.55693851320142 | 0.55901433064454 | 0.130280468855142 | 4.29085292336569 | 0.0000177988172506599 | 0.000338641751622103 |
| IGFBP7-AS1 | 13.5941000153262 | 0.911629328888006 | 0.224476389943685 | 4.06113680426128 | 0.0000488343437581476 | 0.000746397680054831 |
| ARNTL2-AS1 | 1.00629986763531 | -0.609447069424115 | 0.230263688393069 | -2.64673546088502 | 0.00812729031018552 | 0.0380129849724789 |
| AL357153.1 | 1.29520753973078 | -0.638070362458026 | 0.229234354594976 | -2.78348489075908 | 0.0053778367216903 | 0.0280079881462119 |
| AC083805.1 | 5.3206144661453 | 0.938600468079513 | 0.145519782217824 | 6.44998538188127 | 1.11860940586822E-10 | 2.01788823578497E-08 |
| DRAIC | 58.1302266342746 | 0.804916158507978 | 0.191497777859765 | 4.20326631203744 | 0.000026309074945809 | 0.000465271329633922 |
| LINC01096 | 6.38622436356953 | 0.561989783117462 | 0.164873944291531 | 3.40860276942092 | 0.000652964849748017 | 0.0056568107790112 |
| LINC02458 | 1.30909306679168 | -0.67455642259894 | 0.199006395568037 | -3.38962182935633 | 0.000699891017067801 | 0.00599686277037699 |
| AC096746.1 | 0.732313422581467 | 0.97852647338223 | 0.321680006676167 | 3.04192505929442 | 0.00235070410909462 | 0.0148943248988902 |
| PICART1 | 5.62803860585719 | -0.617827103767172 | 0.127673025001541 | -4.83913578267385 | 1.30404934080836E-06 | 0.0000419730683881742 |
| LRP4-AS1 | 18.5885498757317 | 0.606468218824102 | 0.151535133293606 | 4.00216244010585 | 0.0000627661804567322 | 0.000908669526767982 |
| AC108174.1 | 0.483603660837795 | -1.00241892579514 | 0.283908375248027 | -3.53078321454735 | 0.000414331180176988 | 0.00399425290500975 |
| AC026774.1 | 2.19497473169384 | 1.52001796767531 | 0.342734934729988 | 4.43496654017135 | 9.20865902543146E-06 | 0.000199066564401085 |
| LINC01194 | 0.703285470682486 | -2.60619667133921 | 0.982036013204272 | -2.65387077082386 | 0.00795742954182705 | 0.0375017416485034 |
| AC091839.1 | 4.71611919093566 | 1.15187792366934 | 0.343427723497331 | 3.35406213551741 | 0.000796344783000772 | 0.00661928954307632 |
| FLJ12825 | 3.5598260733311 | -0.813585506925725 | 0.195253951325556 | -4.16680687587826 | 0.0000308895973177211 | 0.000525447871910951 |
| SUMO2P17 | 9.01731778735974 | -0.714110865202171 | 0.14199850204058 | -5.02900280594576 | 4.93037177371362E-07 | 0.0000190394333552576 |
| LUCAT1 | 13.8926079049371 | -0.517136303312774 | 0.151432334239406 | -3.41496620196852 | 0.000637899262830954 | 0.00555826658353121 |
| AC010280.1 | 8.1739282539903 | 0.670963276853753 | 0.222719496731237 | 3.01259335936551 | 0.00259025748316055 | 0.016041368657751 |
| LINC00504 | 1.06646499332006 | -0.754639353960042 | 0.226143038790873 | -3.33700014820221 | 0.000846879030168964 | 0.00693640179449192 |
| AC107464.1 | 1.11691665576665 | 0.680448418072394 | 0.243459672380783 | 2.79491223913313 | 0.00519138059624188 | 0.0272671823306928 |
| RPL23AP94 | 0.79606780763535 | 1.15943799973215 | 0.427286462438983 | 2.71349106899851 | 0.00665783628542993 | 0.0327717634762066 |
| ASNSP1 | 1.24044168953967 | -1.64624200516807 | 0.376170356949855 | -4.37632039514353 | 0.0000120699558943575 | 0.000249162632727841 |
| AC008443.3 | 2.44987355920397 | -1.00835194429352 | 0.262091961211899 | -3.84732114495596 | 0.00011941639454839 | 0.00151671973660739 |
| SBF1P1 | 2.25930393426635 | 0.915984073636519 | 0.296478007855916 | 3.0895514991509 | 0.00200458950287191 | 0.0132002095923816 |
| AC022784.1 | 10.6911688988917 | -1.07326557009049 | 0.208548692045275 | -5.14635483715952 | 2.65596814702519E-07 | 0.0000116410074407361 |
| TECRP2 | 7.4435610284583 | 0.788866666608053 | 0.276270026939386 | 2.8554189368546 | 0.00429800932426557 | 0.0235706834736125 |
| AC139491.2 | 15.9634514176761 | -0.595585566196134 | 0.181653054085923 | -3.27869833619432 | 0.00104287051367761 | 0.00804328314538899 |
| AC126768.1 | 0.753602391786343 | 1.07362083073303 | 0.405055613674599 | 2.65055166374147 | 0.00803604358275878 | 0.0377633236806742 |
| AC147055.1 | 43.4495614510627 | 0.777776671328889 | 0.202943169251144 | 3.83248509520606 | 0.000126855287235869 | 0.00158649757507177 |
| AC021127.1 | 0.785908826809708 | -1.35874277630803 | 0.27322253037463 | -4.97302610602787 | 6.59157804559882E-07 | 0.0000244385313748244 |
| LINC01948 | 1.75766489824978 | -0.604214663494658 | 0.208952020058608 | -2.89164308306369 | 0.00383233042641622 | 0.0216159207488972 |
| C8orf34-AS1 | 2.30467870785554 | 1.33576994345686 | 0.397740331895352 | 3.35839701518706 | 0.00078395927185258 | 0.00654774374857492 |
| AC024587.1 | 1.86286343846758 | 0.572382465926658 | 0.221114315252898 | 2.58862690672921 | 0.00963594334087851 | 0.043086639720352 |
| AC010280.2 | 3.64816275912698 | 0.753539280689806 | 0.199460773242178 | 3.77788207897343 | 0.000158167734558639 | 0.00188370561934116 |
| AC022126.1 | 2.94120982939381 | -0.87266324526767 | 0.169804845912556 | -5.13921284506253 | 2.75891751641223E-07 | 0.0000119978829107747 |
| AC131254.1 | 0.572345064307089 | -0.8278022432297 | 0.238718981550495 | -3.46768504897714 | 0.000524962161279083 | 0.00478489631984058 |
| AC093895.1 | 4.15283116694829 | -1.04209854953842 | 0.290136967505293 | -3.59174688595795 | 0.000328468797476068 | 0.00334184774116054 |
| AC114801.3 | 2.45939688188965 | 0.680017711203935 | 0.257325631303096 | 2.64263496706616 | 0.0082263671387688 | 0.0383637107592673 |
| C5orf66-AS1 | 8.36338746362007 | 0.578196607705314 | 0.15254762988746 | 3.79026936132585 | 0.000150484006276433 | 0.00181416227033038 |
| MTND6P4 | 9.03115018510435 | 0.565409763455487 | 0.153984425965218 | 3.6718633063788 | 0.000240788470094775 | 0.00261612996000957 |
| SLED1 | 1.81213680420501 | -0.519144050428613 | 0.186378114026795 | -2.78543461575095 | 0.00534560196502165 | 0.0278836458244233 |
| TMEM132D-AS1 | 10.9611354678117 | -1.61129226885553 | 0.519099610577162 | -3.10401363442366 | 0.00190914532756569 | 0.0127538230212648 |
| TMEM150C | 266.831660942566 | 0.903777204044313 | 0.131388740822296 | 6.87865031956335 | 6.04222714712772E-12 | 1.77249263380917E-09 |
| AC110373.1 | 6.98318933221652 | -0.57415662382646 | 0.109424656774832 | -5.24704980348191 | 1.5455412939891E-07 | 7.47988306130007E-06 |
| LINC00939 | 6.83075179185854 | -1.01958403881093 | 0.272870810143674 | -3.73650827024736 | 0.000186593314743788 | 0.0021421399353456 |
| AC104779.1 | 1.13143467803858 | -0.853527950231337 | 0.210677813053709 | -4.05134236899328 | 0.0000509246284976178 | 0.000771645412876001 |
| LINC01411 | 63.8857987183299 | 0.684084767822507 | 0.256371682751747 | 2.66833201108614 | 0.00762288882540819 | 0.0363638222636757 |
| LINC01016 | 1.69198087008349 | 0.572335529518482 | 0.198795944558189 | 2.87901008640021 | 0.00398925575194508 | 0.0222793404904053 |
| AC108673.1 | 0.917737640927604 | 0.697500468769277 | 0.240221487426182 | 2.90357235001141 | 0.00368931716982175 | 0.0209791098100968 |
| NEUROG2-AS1 | 4.1332066520597 | 1.25951835312614 | 0.354047903381808 | 3.55748005028535 | 0.000374429527539228 | 0.00369698029523674 |
| AC026369.1 | 1.94809036724851 | -0.60124399677035 | 0.180478232511495 | -3.33139342292737 | 0.000864123817155276 | 0.00703628650920544 |
| AC116345.1 | 33.117443140302 | 0.673218077200763 | 0.259790698222572 | 2.59138638067785 | 0.0095590100577464 | 0.0428630215005571 |
| AC092490.1 | 4.48540046850585 | -0.976090639349963 | 0.192038608543748 | -5.08278333587071 | 3.7194388836278E-07 | 0.0000151504841834691 |
| SNRPCP13 | 0.698488589963561 | 0.880056507018488 | 0.289531208277762 | 3.03959118000919 | 0.00236899480736098 | 0.0149878880533203 |
| LINC00964 | 2.46628317699804 | 1.07143440679408 | 0.209720522134629 | 5.10886772495385 | 0.0000003240951380695 | 0.0000136331944746166 |
| HS3ST5 | 6.41861988952524 | 0.646783942136159 | 0.228345399595692 | 2.83248072122913 | 0.00461883513191659 | 0.0249109009510519 |
| AC007663.2 | 2.13799273495948 | -1.14991900833504 | 0.236640684892453 | -4.85934618071972 | 1.17774043570073E-06 | 0.0000385759017146951 |
| SLC7A11-AS1 | 6.13786379279315 | -0.824381624594566 | 0.160917652969391 | -5.12300303529395 | 3.00707506790469E-07 | 0.0000128105228561616 |
| AC017013.1 | 1.23811597756918 | 0.747741453000376 | 0.245170792424102 | 3.04987982298853 | 0.00228932952152922 | 0.0145994961592258 |
| HOXC-AS2 | 5.96320160963506 | -1.80841223135249 | 0.269337019806267 | -6.71430994763837 | 1.88958279069002E-11 | 4.48166153803231E-09 |
| AC106791.1 | 4.76787761361058 | 0.638090555145571 | 0.225707777138707 | 2.82706499188744 | 0.00469767925997792 | 0.0252330484648988 |
| AC034206.1 | 4.02223821847487 | 0.787633820639913 | 0.199912145870293 | 3.93989978553352 | 0.0000815156538014725 | 0.00112090727566975 |
| AP002754.1 | 6.46815836153221 | 0.844619555456179 | 0.22641338507706 | 3.73043119852968 | 0.000191152339183613 | 0.00218249113246104 |
| AC093844.1 | 0.74040792705419 | 1.26637232359119 | 0.278026900488514 | 4.55485538041849 | 5.24216307010143E-06 | 0.000127589823428189 |
| LINC00989 | 1.46572237527004 | -0.568796244354778 | 0.20359721311324 | -2.79373295762362 | 0.00521034853825609 | 0.0273410443772729 |
| AC093720.1 | 21.4340143397381 | 0.820282317459643 | 0.23326997572379 | 3.51645047723982 | 0.000437358240079977 | 0.00415515177975983 |
| AC114296.1 | 6.75106686608401 | 0.795337943846744 | 0.188910071611792 | 4.2101405026258 | 0.0000255211922587457 | 0.000453014708873128 |
| UNC93B4 | 0.997183839357922 | -0.68172964573655 | 0.217321066003445 | -3.13696991402454 | 0.00170703642955721 | 0.0117317534478529 |
| AC022113.1 | 0.578797709424898 | 0.602680000781438 | 0.232442867437825 | 2.59280918113199 | 0.00951955721768511 | 0.0427147500971187 |
| AACSP1 | 1.76751727401546 | -1.15371084227988 | 0.305462283742003 | -3.7769338595475 | 0.000158770871865915 | 0.00188874181192043 |
| OR7E35P | 0.348748240950127 | 0.971657123833526 | 0.36594879552971 | 2.65517235116748 | 0.00792678970040456 | NA |
| HOXC-AS1 | 1.70477082692082 | -1.40586059492958 | 0.277740171113377 | -5.06178342619257 | 4.15352694022677E-07 | 0.0000165556910530469 |
| AP002784.1 | 1.7364404034193 | -0.649072912807372 | 0.245095742724476 | -2.64824229744793 | 0.0080911511056601 | 0.037921486687785 |
| LINC01303 | 4.38589842690537 | -0.506207476046318 | 0.153313638070367 | -3.30177720923941 | 0.000960743476327413 | 0.00758658402251272 |
| LY6G6F-LY6G6D | 313.751060034761 | 0.847103094418257 | 0.258899799901001 | 3.2719341410931 | 0.00106814454691761 | 0.00820415478594824 |
| AC083906.3 | 1.14020955748285 | 0.619533745374041 | 0.223086545150003 | 2.77710045201278 | 0.00548462255500298 | 0.0284278901866723 |
| AC068580.4 | 9.76330728296221 | -0.751130408584702 | 0.11854572850516 | -6.33620812876447 | 0.0000000002354886366 | 0.0000000364593101158 |
| AC023794.3 | 2.84180359417843 | -0.688584417394979 | 0.173479632608432 | -3.969252223108 | 0.0000720985342397245 | 0.0010135011273833 |
| LINC00491 | 1.67295136864166 | -1.80911135870988 | 0.633380188028318 | -2.85628030826407 | 0.00428636526498971 | 0.023526116394516 |
| AC111000.4 | 0.938384717273588 | 0.792508575519948 | 0.298307731874862 | 2.65668130872451 | 0.00789140038709987 | 0.0373057811835359 |
| Z99943.2 | 3.61849829744406 | 0.698465464598723 | 0.165957156931515 | 4.20870950981014 | 0.0000256833321794366 | 0.000455409329132938 |
| AC139783.1 | 1.83088979188904 | 0.73114856705772 | 0.20750205450363 | 3.52357266440911 | 0.000425770332130629 | 0.00407166469748713 |
| AC106865.1 | 4.92642230246946 | -0.930272449745327 | 0.211481775023156 | -4.39883034669757 | 0.0000108835862912579 | 0.000229633370821606 |
| AC127070.2 | 6.42833546373134 | 0.509626188918309 | 0.149385334591796 | 3.41148741481814 | 0.000646094837932821 | 0.00561504770534028 |
| PRODH2 | 1.32287956856048 | -1.97769742563971 | 0.511068551576643 | -3.86973023391583 | 0.000108955846210238 | 0.00140574900037145 |
| OR7E85P | 1.98480428348711 | 0.942549536843517 | 0.247607219089546 | 3.80663189187004 | 0.000140872181046829 | 0.0017187331187771 |
| RNPS1P1 | 26.2985463064194 | 0.640614926012289 | 0.147555901366345 | 4.34150664311148 | 0.0000141508999370091 | 0.000284224862278352 |
| AC008443.5 | 3.14389286991979 | -0.646626916982181 | 0.171084494228164 | -3.77957640111918 | 0.000157095386247792 | 0.00187360339047741 |
| AC105460.1 | 65.3374846026043 | -2.27716034276471 | 0.44404377769442 | -5.12823387502076 | 2.92472966256223E-07 | 0.0000125395909455649 |
| AC244502.1 | 8.84169501336906 | -0.707868400271206 | 0.176095724770756 | -4.01979321867534 | 0.0000582492443322258 | 0.000856627629269259 |
| ZFPM2-AS1 | 8.37167687847459 | -0.623901799822344 | 0.164705904356782 | -3.78797470715356 | 0.000151880306591191 | 0.00182573012689526 |
| SLC25A30-AS1 | 4.39186777471465 | 0.588025185344492 | 0.126584531596167 | 4.64531627940468 | 3.39555838725026E-06 | 0.0000897116367603381 |
| H3P24 | 6.93011131432805 | 0.959418935766881 | 0.221667127381476 | 4.32819672948518 | 0.0000150335158221219 | 0.000296608162904661 |
| AC091173.1 | 0.402089550044243 | -1.31876601466839 | 0.386630228709038 | -3.41092319416349 | 0.000647433262706416 | 0.00562031710017288 |
| LINC02506 | 0.622791328281839 | -1.63074910919479 | 0.529553797107321 | -3.07947770009909 | 0.00207363893596028 | 0.0135389756533354 |
| AC025171.3 | 1.43168850206351 | -0.525120308302955 | 0.1841647693887 | -2.85136136540117 | 0.00435324647673007 | 0.0238072393581042 |
| LINC02882 | 3.34914747922199 | -1.30521765965626 | 0.320778963667311 | -4.06890041894999 | 0.00004723552865607 | 0.000729271791139372 |
| AC113346.1 | 2.11466083892046 | -1.09502096498051 | 0.279974945473642 | -3.91113913113915 | 0.0000918618328000816 | 0.0012341274513684 |
| HOXC-AS3 | 1.38126549876726 | -1.70603382402992 | 0.422792012953355 | -4.03516095801494 | 0.0000545648788898014 | 0.000814988244677418 |
| LINC00589 | 9.11975828866784 | -0.802729862490893 | 0.178247900957126 | -4.50344636980592 | 6.68602460868864E-06 | 0.000153884401213879 |
| MIR3945HG | 4.10399146268498 | -0.83971116577895 | 0.208857113262069 | -4.02050546741637 | 0.0000580734014147094 | 0.000855028234958269 |
| ZNF345 | 82.9452683657312 | 0.657314545586155 | 0.108290267673395 | 6.06993185729878 | 1.27964535147808E-09 | 1.51215193795512E-07 |
| RFPL4B | 0.591376175299479 | -1.42936151222358 | 0.402037269816866 | -3.55529603729196 | 0.000377553740158261 | 0.00372013912150046 |
| AC011352.3 | 1.82422195608387 | -0.792399882983995 | 0.260010583539559 | -3.04756780357534 | 0.00230701473116452 | 0.0146926655188733 |
| AC021106.2 | 0.710478268826457 | -0.624419648669196 | 0.22800790808343 | -2.7385876828479 | 0.00617037041211969 | 0.0310031758604328 |
| RTEL1P1 | 3.38509279637335 | 0.605942589024099 | 0.181029228270131 | 3.34720859617163 | 0.000816297602776203 | 0.00673873720860078 |
| FOXD1 | 93.6455415496657 | -1.05246434532033 | 0.224760856563094 | -4.68259625547782 | 2.83264099705355E-06 | 0.000077646869035627 |
| AC096734.1 | 0.763862815440671 | -0.693806589277607 | 0.256905038570203 | -2.70063441783379 | 0.00692073647127848 | 0.0337528465907095 |
| IMPA1P1 | 2.17669847808318 | -1.00617863686429 | 0.241809161602287 | -4.16104431361121 | 0.0000316795601099059 | 0.000533405422898604 |
| AC079140.4 | 0.972412232505902 | 0.661820068081108 | 0.20090699667099 | 3.29416137340862 | 0.000987158074361517 | 0.00773226918771933 |
| AC091133.5 | 0.974722089798763 | -0.774235876277582 | 0.251210830876703 | -3.08201630310114 | 0.00205603576431463 | 0.0134661081140246 |
| LINC01267 | 2.4020291130848 | 0.888734023634374 | 0.181325512372047 | 4.90131814331153 | 0.0000009519574755348 | 0.0000328199607183863 |
| AC105460.2 | 2.97398583539841 | -2.36635233362363 | 0.519043159141375 | -4.5590666054402 | 5.13814749486558E-06 | 0.000125566449055881 |
| MSNP1 | 7.18489946016122 | 0.523959002457876 | 0.200990045950912 | 2.60689030632812 | 0.00913686256386183 | 0.0414525530521317 |
| AC107223.1 | 0.854414848401261 | 0.82001748793477 | 0.26200789624461 | 3.12974341494351 | 0.00174959051296488 | 0.0119578593775949 |
| LINC02172 | 1.9746858422385 | 0.55462889364411 | 0.171906780213118 | 3.22633518559605 | 0.00125386422852283 | 0.00925645199343498 |
| PRB1 | 1.37508617724526 | -1.87958608819531 | 0.49800084150139 | -3.77426287579889 | 0.000160481470177692 | 0.00190177934999375 |
| UGT2B27P | 0.509583388704473 | -1.2465058084929 | 0.391977738330312 | -3.18004235088089 | 0.0014725352977167 | 0.0104753298077519 |
| RNA5-8SP6 | 1.04637699723052 | 1.88835504369215 | 0.40629811405084 | 4.64770811969819 | 3.35643386765659E-06 | 0.0000890840169858504 |
| SCARNA6 | 6.22462952311501 | 0.809483796419351 | 0.161894311477575 | 5.00007559889761 | 5.73078397524954E-07 | 0.0000217042896602826 |
| SCARNA5 | 9.96520156895998 | 2.46710756700463 | 0.262018860771952 | 9.4157632764913 | 4.69657499616828E-21 | 1.96328576277325E-17 |
| SNORA74D | 1.24277707283287 | 1.24630142639238 | 0.260693909688962 | 4.78070787261338 | 1.74679034479112E-06 | 0.0000533262569391313 |
| RNA5SP243 | 1.01228140569592 | 2.3082866727044 | 0.646364020636838 | 3.57118682198636 | 0.000355367293130597 | 0.00355494855425469 |
| AC104211.1 | 0.393432845060863 | 0.806561905980994 | 0.270187411092479 | 2.98519424987172 | 0.00283398294131438 | NA |
| KRT18P41 | 1.16834918721335 | -0.509911445418839 | 0.191949755155134 | -2.65648395855847 | 0.00789602074724672 | 0.0373122404732832 |
| AC110998.1 | 2.05867028825089 | 0.798742585563451 | 0.233892638293107 | 3.41499668990219 | 0.000637827866550281 | 0.00555826658353121 |
| IGLV3-29 | 6.07891531997855 | 0.814770391411454 | 0.315627509085962 | 2.58143022378176 | 0.0098391879176299 | 0.0437265278858975 |
| AC111149.2 | 1.94869995768767 | -1.32481574574118 | 0.321154436399253 | -4.12516719555508 | 0.0000370465529676454 | 0.000602183003861416 |
| AC008514.1 | 4.17936483380376 | -0.793292473824286 | 0.225857981065196 | -3.51235085907942 | 0.000444161212823776 | 0.00420306714183721 |
| AC069120.1 | 3.56792913334537 | -1.07758792934212 | 0.296215132871623 | -3.63785576683935 | 0.000274917299432597 | 0.00291403623696511 |
| TRNP1 | 335.37640755116 | -1.19107755442604 | 0.160662013408301 | -7.41356048737592 | 1.22952925229501E-13 | 6.8529862092083E-11 |
| LINC02159 | 10.960853808528 | 0.695707615116998 | 0.202133733870807 | 3.44181845253825 | 0.000577817903066686 | 0.00515290301716163 |
| PCDHGA5 | 17.6493190722754 | -0.508160642149496 | 0.137437312880499 | -3.69739942886791 | 0.000217819470511166 | 0.00241230040437683 |
| AC104966.1 | 0.66501503491998 | -0.88815366208316 | 0.324687550887272 | -2.7354102726024 | 0.0062302569317381 | 0.0312044709167569 |
| PCDHGA7 | 26.6397377075615 | 0.745412461476691 | 0.186646606345374 | 3.99371023171654 | 0.0000650473512308878 | 0.000934413023996284 |
| EIF5AL1 | 158.424028532563 | -0.961195462526697 | 0.145029485407798 | -6.62758652024433 | 3.41219645508664E-11 | 7.31478678532098E-09 |
| AC084026.2 | 1.70432922309519 | -1.01708422244108 | 0.228610173546799 | -4.44898932825872 | 8.62753022982296E-06 | 0.000189194666193927 |
| LINCR-0001 | 19.8782726353131 | 0.569502847122927 | 0.201568560827526 | 2.82535552560812 | 0.00472281809263138 | 0.0253190898771688 |
| PRSS51 | 27.4813782947827 | 0.765029155928385 | 0.21827095924849 | 3.5049516370038 | 0.000456690236003868 | 0.00429166217834591 |
| AC138646.1 | 1.50401267889276 | -1.7351001407827 | 0.571047530779975 | -3.03845134994768 | 0.00237797497392176 | 0.0150329374438358 |
| AC008456.1 | 2.85205411672617 | 0.702378401340291 | 0.20142745133281 | 3.48700436158416 | 0.000488463391250548 | 0.00452456266986838 |
| AC011632.1 | 0.998243139299524 | -1.00555075152784 | 0.316399983613917 | -3.1780998849698 | 0.00148243670627585 | 0.0105300867313672 |
| AC064807.2 | 14.1663246467241 | 0.689727914297694 | 0.186355455043854 | 3.70114153157139 | 0.000214631712161284 | 0.00238779564806975 |
| AC090150.1 | 0.866107490197314 | 1.06308524941339 | 0.348132983803131 | 3.05367574712359 | 0.00226056269151286 | 0.014462905678885 |
| LINC01419 | 1.13571060114832 | -1.83739980098303 | 0.391594376827851 | -4.69209955430686 | 2.70415474485957E-06 | 0.0000750475875332728 |
| AC067904.2 | 2.05750841734129 | -0.988071886916253 | 0.262761710292926 | -3.76033435699118 | 0.000169686403611792 | 0.00198831559551 |
| MAPRE1P1 | 2.32652573348556 | 0.634164107344458 | 0.187209024623043 | 3.38746547406775 | 0.00070541595132136 | 0.006031838466911 |
| IGLV3-31 | 1.31881758301596 | 1.29295102458208 | 0.415974273464772 | 3.10824756976607 | 0.00188200342839728 | 0.0126178746296034 |
| AC105118.1 | 0.92682213661147 | -0.939070274799206 | 0.282844968520661 | -3.32008831449519 | 0.000899889756817193 | 0.00723764147366055 |
| PCDHGB4 | 17.1134656415497 | 0.796909246135504 | 0.195564927663081 | 4.07490880731139 | 0.0000460323820145904 | 0.0007176759530685 |
| RPL10P8 | 6.6726643789491 | 0.733373017593011 | 0.156552056453792 | 4.68453135784564 | 2.80601207994512E-06 | 0.0000770432315086411 |
| IGHEP2 | 1.40969191079205 | -0.688823438475246 | 0.247648186380672 | -2.78145965267204 | 0.00541150568269919 | 0.028144879166536 |
| AC011676.2 | 1.09691340226707 | -0.80969220925343 | 0.276800272666841 | -2.92518573573799 | 0.00344250687502327 | 0.0199148940018518 |
| AP001207.3 | 2.30151493564514 | 0.598374111109497 | 0.20631389162687 | 2.90030936061101 | 0.00372794529427507 | 0.0211555992756061 |
| AC087273.2 | 1.40832970353133 | -0.922923573798477 | 0.289273823857049 | -3.19048423218051 | 0.00142034591254177 | 0.0101667825357923 |
| AP003467.2 | 0.492156630572096 | 0.813505175349583 | 0.309744018088402 | 2.62637897051302 | 0.00862986763205185 | 0.0397404562419389 |
| AC104417.1 | 0.611985466749735 | 0.984916647932281 | 0.377227863046483 | 2.61093292520367 | 0.00902956019898569 | 0.0411342531228006 |
| PCDHGB1 | 10.0583231110105 | -1.06700778288941 | 0.206992131924097 | -5.15482290544586 | 2.53870739218271E-07 | 0.0000111761359431813 |
| RPSAP71 | 15.1605671955186 | 1.06497809711989 | 0.323739641952181 | 3.28961288366778 | 0.00100325305118812 | 0.00782982229587703 |
| AC007991.4 | 2.01351344455301 | -1.07775041208871 | 0.265847400510631 | -4.05401899743461 | 0.0000503451251683359 | 0.00076390275675113 |
| AC083967.1 | 2.07865571060539 | -1.44141163980031 | 0.258304330692991 | -5.58028444948335 | 2.40125562264825E-08 | 1.69058506384427E-06 |
| SPON1-AS1 | 0.991659117884698 | -0.533331182051359 | 0.202082227877058 | -2.63917904931168 | 0.00831070745849626 | 0.0386707498020081 |
| AL139349.1 | 16.879077107144 | 0.643019606585086 | 0.132756401643474 | 4.84360526968756 | 0.000001275041178878 | 0.0000413578342425198 |
| HSPD1P2 | 4.3378680629863 | 0.805809084630428 | 0.248981453402501 | 3.2364221254977 | 0.00121038247225447 | 0.00902215731620459 |
| AL354919.2 | 1.48784775262441 | -0.516012621998255 | 0.188190179413253 | -2.74197422844859 | 0.00610711329886075 | 0.0307488833093197 |
| OR5M6P | 1.02180890482891 | 0.784394846021106 | 0.24993832338868 | 3.13835363615403 | 0.00169899762015367 | 0.0116861123844465 |
| AP003555.1 | 7.99915304830862 | -0.659521582666222 | 0.171158074898866 | -3.85328932366129 | 0.000116541542959054 | 0.00148819480674742 |
| AC087379.1 | 10.5441686682329 | 0.650832121508879 | 0.214578860374114 | 3.03306728525898 | 0.00242081616892536 | 0.0152375182234523 |
| RASSF10-DT | 8.60068920968616 | 0.56541545278891 | 0.159435698207121 | 3.54635416752392 | 0.000390600865893154 | 0.00381943688806984 |
| AC087379.2 | 0.51311514185067 | 0.882058978995711 | 0.320859777083852 | 2.749048157461 | 0.00597686042322796 | 0.0302616451587569 |
| MEX3A | 517.171727268272 | 0.535952787290378 | 0.116849172038482 | 4.58670590420504 | 0.0000045029428870608 | 0.000112127636656059 |
| CASP1P2 | 6.63628185593685 | -0.789340628096213 | 0.207902489871013 | -3.79668674764759 | 0.000146642901803228 | 0.00177770032858007 |
| LINC02688 | 48.8664831946782 | -0.5650141322766 | 0.148389170337565 | -3.80765072674286 | 0.000140293230532007 | 0.0017141313603283 |
| AC139103.1 | 1.20395486486432 | 0.969712728967046 | 0.225080738444998 | 4.30828837539117 | 0.0000164522788869909 | 0.000318770052455823 |
| AC136475.3 | 223.121376726398 | 0.544342461937828 | 0.182296599043444 | 2.98602642503552 | 0.00282628219472184 | 0.0171723345123342 |
| AP006621.1 | 12.9389330273034 | 0.536927670710302 | 0.170095494352118 | 3.15662488742235 | 0.0015960652325437 | 0.0111571098467237 |
| AC087623.1 | 2.19797907539351 | -0.723785405599246 | 0.175069988947905 | -4.13426315925924 | 0.0000356095260854079 | 0.00058403814190692 |
| SMIM35 | 1.22925223456334 | -0.714879678080631 | 0.171788705805024 | -4.16138927603309 | 0.0000316317356659089 | 0.000533405422898604 |
| ABCC6P2 | 22.1682597297493 | 0.620067325641386 | 0.131023390847098 | 4.7324933481915 | 2.21778574620581E-06 | 0.0000641584696579712 |
| AC108136.1 | 2.03923623425584 | -1.00661527881818 | 0.273426630527511 | -3.68148222020716 | 0.000231881962227787 | 0.00253501032390378 |
| AC136475.5 | 10.5525254261959 | 0.728919400860418 | 0.158845178424401 | 4.58886702190544 | 4.45658199162625E-06 | 0.000111304716179212 |
| AL096711.2 | 2.67394834049824 | -0.519026779345331 | 0.192398222598326 | -2.69766930450763 | 0.0069826767761796 | 0.0339608314061952 |
| AC120036.4 | 2.27376608260589 | 0.52167764210076 | 0.182422820445468 | 2.85971700704357 | 0.00424019201416864 | 0.0233275373949331 |
| HCAR3 | 32.4519774882189 | -0.793600146288296 | 0.235123220107861 | -3.37525211641894 | 0.000737480960002477 | 0.00624059672682258 |
| AC091564.5 | 1.35208493366224 | 0.598944517825899 | 0.230892924947768 | 2.59403581968305 | 0.00948566038105056 | 0.0425969456778693 |
| AP004608.1 | 275.381932348682 | 0.576371727294374 | 0.149557881657925 | 3.85383719604076 | 0.000116280931254811 | 0.00148649346445849 |
| LY6G6E | 9.73682163799194 | 1.07019994239213 | 0.196094012560243 | 5.45758602427163 | 4.82651333245359E-08 | 2.91350647768796E-06 |
| PCDHB17P | 1.09363863358455 | -1.07044554123966 | 0.272393232455721 | -3.92978023568801 | 0.0000850235340914901 | 0.00116077379319641 |
| LINC02700 | 1.25770678789839 | -1.59265695773723 | 0.382223918325293 | -4.1668165736865 | 0.0000308882838065208 | 0.000525447871910951 |
| TRIL | 253.218807883176 | 0.627458175538636 | 0.138306507073695 | 4.53672201557588 | 5.71353373586276E-06 | 0.000136773081742822 |
| HNRNPABP1 | 5.83445919985733 | 0.515549783445068 | 0.198129205042744 | 2.60208879016016 | 0.00926578594906478 | 0.0418895322742079 |
| LINC02422 | 1.06449380280293 | -0.775493462985856 | 0.263642410353712 | -2.94145946376923 | 0.00326669594973721 | 0.0191562560924508 |
| KLRC4-KLRK1 | 2.44359187475529 | -0.855766832243048 | 0.193836536183906 | -4.41488921072715 | 0.0000101061734424854 | 0.000216093767431966 |
| AC026310.2 | 1.36343588874554 | -0.851894469051208 | 0.283576557704135 | -3.00410751843606 | 0.00266361176657156 | 0.0163800492457006 |
| MT1JP | 0.729382946743889 | -0.785010170310585 | 0.300312363121966 | -2.61397886570447 | 0.00894945722386392 | 0.0408583956969908 |
| MRPL40P1 | 1.62035006226095 | -0.568153720458879 | 0.190050889856192 | -2.98948203235875 | 0.0027945088275854 | 0.0170270482578717 |
| LINC02446 | 11.943162920688 | -1.33672748812648 | 0.221345599313639 | -6.03909674405764 | 1.54979309650967E-09 | 1.74231970302028E-07 |
| AC243972.2 | 0.628509843253769 | 0.980140076541583 | 0.328958056935225 | 2.97952901860246 | 0.00288691884105107 | 0.0174425796620396 |
| AC092112.1 | 5.54366980212752 | -0.78819190288759 | 0.259704197128778 | -3.0349602031913 | 0.0024056742805267 | 0.0151736249131222 |
| LINC00944 | 16.0125314256247 | -1.02254223263156 | 0.18654844303812 | -5.48137639735006 | 4.22029523211503E-08 | 2.63311778269386E-06 |
| AC023512.1 | 4.30541423225066 | 0.65481931609465 | 0.226271948109635 | 2.89394828464275 | 0.00380430813685918 | 0.0214868557191091 |
| UBE2NP1 | 0.991195953252066 | 0.702967711416812 | 0.220787050484644 | 3.18391730798408 | 0.0014529651288465 | 0.0103647738564174 |
| LINC02393 | 0.707480651466868 | -1.28527096950045 | 0.462460765809854 | -2.77920001981077 | 0.00544929591665742 | 0.0282834162407414 |
| AP002840.1 | 1.25107062144656 | 0.852444098951661 | 0.320759242928392 | 2.65758233860767 | 0.00787033623776151 | 0.0372487665529607 |
| TPT1P12 | 17.8281762778803 | 0.870719556988305 | 0.26834551583061 | 3.24477028912954 | 0.00117545403852299 | 0.00883161850287256 |
| AP000721.2 | 1.68981809594944 | 0.621473985889403 | 0.22539725533435 | 2.7572384808658 | 0.00582918206788801 | 0.02975199920573 |
| SLC5A8 | 8.46584250621139 | 1.6364884992987 | 0.387961101446669 | 4.21817675327859 | 0.0000246285726959006 | 0.000439667077193486 |
| AC026369.3 | 2.16342611532604 | -0.654404292346878 | 0.222080822498722 | -2.94669429347347 | 0.00321190567766942 | 0.0189273215282151 |
| AL513165.2 | 1.90874712955028 | -0.651559093042825 | 0.155310447941546 | -4.19520451893907 | 0.0000272625480451073 | 0.000477837595243437 |
| SLCO1B3-SLCO1B7 | 1.88598740458984 | -0.848838498766328 | 0.299687023450567 | -2.83241659579679 | 0.00461976163953781 | 0.0249109009510519 |
| AC009318.1 | 17.153888058213 | -0.600672410462857 | 0.12932676975734 | -4.64461001840469 | 0.0000034071944354646 | 0.0000898607226425924 |
| HMGN2P6 | 2.42238865317922 | -0.701303770047395 | 0.177760316560999 | -3.94522120355665 | 0.0000797263452281182 | 0.00109946739674999 |
| FAHD2P1 | 17.1978773263183 | -0.857415188986859 | 0.160352135985287 | -5.34707681764546 | 8.93860862257634E-08 | 4.79815328340607E-06 |
| AC011611.2 | 2.00764755271206 | -0.575604518053604 | 0.218212784257131 | -2.63781299529793 | 0.00834425823681554 | 0.0387998726300869 |
| AL928654.3 | 0.648110718811696 | -0.690119854761624 | 0.225984929833172 | -3.05383131198655 | 0.00225939086211467 | 0.0144609663561414 |
| LINC02413 | 6.24001008283942 | 0.754524931638139 | 0.206626907188621 | 3.65162960576748 | 0.00026058153438008 | 0.00279575478753245 |
| AC126763.1 | 0.606543472017197 | 1.29344068414235 | 0.388687900866355 | 3.3277101789363 | 0.000875629095460705 | 0.00709369869437909 |
| AC092375.1 | 5.62206417482828 | 0.502804723742861 | 0.154687839640878 | 3.2504476428798 | 0.00115223482778477 | 0.00871565394842945 |
| AC023794.4 | 0.611050725572193 | -0.778345238651131 | 0.255189796674574 | -3.05006410441912 | 0.00228792526462005 | 0.0145933238030562 |
| AC025154.2 | 20.3548484003648 | -0.749680925809762 | 0.197769026765778 | -3.79068926044534 | 0.000150229808703169 | 0.00181323294967986 |
| AC025265.1 | 23.0553437884051 | -0.514501707650491 | 0.112999597832238 | -4.55312866169962 | 5.28539256034086E-06 | 0.000128268576199506 |
| LSM6P2 | 0.813806190573736 | 1.03697929240613 | 0.366833372557385 | 2.82684011320128 | 0.00470097931578335 | 0.025234374041481 |
| AC089983.1 | 1.16484232358394 | -0.622196975720311 | 0.228066579583564 | -2.72813744502332 | 0.00636930631083209 | 0.0317203785028811 |
| AC025265.3 | 3.76817353687633 | -0.752191419901049 | 0.196106516111087 | -3.83562685635066 | 0.000125244439692576 | 0.00157106969154376 |
| AC121757.1 | 3.34570758998618 | -0.751945917421083 | 0.165147047067427 | -4.55319020699218 | 5.28384588624585E-06 | 0.000128268576199506 |
| LINC02404 | 1.95485682939303 | -1.78507096759333 | 0.435474885897166 | -4.0991364264686 | 0.0000414694550648884 | 0.000658197207536781 |
| AC004801.3 | 0.955824117213978 | 0.885058211834284 | 0.23893207409957 | 3.70422520781138 | 0.000212037820386494 | 0.00236457432242527 |
| AC078923.1 | 10.2607267409131 | -0.830930970889123 | 0.174559689220959 | -4.76015381671151 | 1.93445473722819E-06 | 0.000057760745823558 |
| AC078820.1 | 3.66617106642041 | -0.884020561055899 | 0.20738336924262 | -4.26273603464159 | 0.0000201939012730857 | 0.000375806592306361 |
| CLEC5A | 98.551298953294 | -0.703583019144644 | 0.149727211506866 | -4.69909919555526 | 2.61311516755916E-06 | 0.0000734912228827144 |
| AL352984.1 | 0.926592756182618 | -1.00593395071485 | 0.313541220265233 | -3.20829889564091 | 0.00133522652990134 | 0.0096881418123152 |
| AL512361.1 | 5.1701061345374 | 0.791092728613609 | 0.252926492304028 | 3.12775748165867 | 0.00176145458479295 | 0.012016842967084 |
| AC012651.1 | 45.133474388896 | 0.707143743374584 | 0.127824331081569 | 5.53215289601892 | 3.16323980052803E-08 | 2.12847214103136E-06 |
| LINC02251 | 13.788589141919 | 0.777647553793998 | 0.205509250193971 | 3.78400268143653 | 0.00015432615904893 | 0.00184835197017488 |
| AL157871.2 | 6.23955830832296 | -0.592279429660857 | 0.148548139674011 | -3.98712115116767 | 0.0000668798785154682 | 0.000956628270878652 |
| ASB9P1 | 1.97702918658596 | 0.575841471086945 | 0.178470554531972 | 3.22653489029074 | 0.00125298954497284 | 0.00925203717442741 |
| CRAT37 | 13.8463552391987 | 0.76930257102825 | 0.276508659723254 | 2.78220064354661 | 0.00539916492244814 | 0.0280918683417665 |
| BLZF2P | 0.657666770920506 | 0.690124940371096 | 0.264342957165133 | 2.61071809051444 | 0.00903523407558307 | 0.0411432867586668 |
| AL355922.2 | 4.10098068811625 | 0.534793195089564 | 0.190953454110237 | 2.80064687796026 | 0.00510002926786554 | 0.0269014477564605 |
| AL161668.4 | 3.02935322485446 | 0.774000911314998 | 0.186544881573364 | 4.14914043626869 | 0.000033372603627762 | 0.00055497096495257 |
| LINC00930 | 3.27546620150929 | -1.08183618732163 | 0.232314426544353 | -4.65677574748072 | 3.21199980256912E-06 | 0.0000858638668245536 |
| HSBP1P1 | 0.756564642060387 | 1.15936400862873 | 0.351732828289168 | 3.29614956405374 | 0.00098019816974289 | 0.0076875673528475 |
| LINC02308 | 0.887838175804163 | 1.23341394946189 | 0.258130012792903 | 4.77826633221243 | 1.76813081546789E-06 | 0.0000538033036677682 |
| AC091544.2 | 2.47922917139176 | -0.916161081678739 | 0.257526347973912 | -3.55754309757675 | 0.000374339698820587 | 0.00369698029523674 |
| LINC01269 | 6.86589853392045 | -0.708082440681432 | 0.178611648966725 | -3.96436875633653 | 0.0000735904204161905 | 0.00102762710428037 |
| AL355102.2 | 1.40827650548259 | -0.615699313007109 | 0.20526200999034 | -2.99957753037732 | 0.00270354307980095 | 0.0165862938313526 |
| AL049873.2 | 17.5492907764933 | 0.547669739100634 | 0.137051505579594 | 3.99608699506456 | 0.0000643980771858011 | 0.000927876129792141 |
| AL161670.2 | 1.39115936854644 | 0.594452744632664 | 0.22977657049725 | 2.58709033452033 | 0.00967902134250889 | 0.0432171594304138 |
| LINC00520 | 15.9632640566553 | -1.22238465148057 | 0.20223139273554 | -6.04448515606625 | 0.0000000014988785221 | 1.69342890324917E-07 |
| AL137230.1 | 2.87028292074073 | 0.604140037128505 | 0.196322146722946 | 3.0772892779187 | 0.00208892473123437 | 0.0136039198907194 |
| AL139317.2 | 2.98235075895317 | 0.944307450983093 | 0.32397024596153 | 2.91479684555731 | 0.00355920146581324 | 0.0204197658980488 |
| AL110505.1 | 0.947846078843494 | 1.78090696451106 | 0.47273273704363 | 3.76725964791116 | 0.000165049316646857 | 0.00194488345570972 |
| AC007040.2 | 1.66759813088922 | -1.20787994536226 | 0.228022436111136 | -5.29719779317482 | 1.17593297684233E-07 | 6.02228952703846E-06 |
| AL133467.2 | 1.64615019614267 | -1.79270755816416 | 0.47344451282543 | -3.78652093244381 | 0.000152771232062633 | 0.00183380313806123 |
| COX4I1P1 | 4.03950633117627 | 0.892107068165216 | 0.272020344177604 | 3.2795601037206 | 0.00103969061201773 | 0.00802801511131306 |
| TMEM179 | 7.7183915983915 | 0.65128653530014 | 0.22378880498931 | 2.91027308238789 | 0.00361113083735347 | 0.020636303495178 |
| AL355075.4 | 2.56345344387057 | 2.3655705710564 | 0.342443878192027 | 6.90790731475682 | 4.91855143673626E-12 | 1.53793175990547E-09 |
| LINC00524 | 3.28036793855245 | 0.778184318866652 | 0.268048003239046 | 2.90315282883366 | 0.00369426310244703 | 0.0209890327499941 |
| LINC00648 | 1.37135375672607 | -2.55233104212068 | 0.480320084845402 | -5.31381285657082 | 0.0000001073548727027 | 5.57478517534735E-06 |
| AL355075.5 | 7.01702696968166 | -0.762848955052907 | 0.186007366238473 | -4.101176047377 | 0.0000411055673575362 | 0.000652834953628377 |
| GOLGA8VP | 1.00030937636257 | -0.616358100307329 | 0.201280692301815 | -3.06218193736693 | 0.00219729853868101 | 0.0141420434431428 |
| AC116158.1 | 2.21591613356897 | -0.548279298429168 | 0.179964896674987 | -3.04659024375932 | 0.00231452989761339 | 0.0147209031639382 |
| AC025580.1 | 141.469167679618 | 0.572648552540681 | 0.147145781176915 | 3.89170894306634 | 0.000099540613729821 | 0.00131211557128604 |
| CTXND1 | 15.1086855512869 | 1.77191883679772 | 0.222819049875241 | 7.95227714053102 | 1.831140434751E-15 | 1.85566661875584E-12 |
| AC051619.2 | 1.91366430668474 | 0.767881812770726 | 0.247872727103346 | 3.09788745919826 | 0.00194905428772437 | 0.0129325939464441 |
| AC100826.1 | 0.991475567544176 | 1.02966707983612 | 0.283240948233275 | 3.63530445106437 | 0.000277652538968599 | 0.00293744264732297 |
| AHCYP7 | 1.48656135277679 | 0.886844214488595 | 0.314751960844342 | 2.81759710760682 | 0.00483844810314238 | 0.0258224145524518 |
| AC016705.2 | 13.7616062872095 | -0.637988147195503 | 0.215704029863277 | -2.95770156728128 | 0.00309942034065003 | 0.0183973757600316 |
| AC027243.1 | 1.45136372595485 | -0.784076500931816 | 0.260427774036008 | -3.01072534922257 | 0.0026062447540494 | 0.016110542895549 |
| AC010478.1 | 12.0793582963286 | -0.559841838216231 | 0.204606698869083 | -2.73618528284083 | 0.00621560180372136 | 0.0311497310834782 |
| AC124248.1 | 24.7038998875295 | 0.506393502355462 | 0.116461754352873 | 4.34815279204121 | 0.0000137288949846699 | 0.000276632673421779 |
| AC090877.2 | 6.68262810915619 | -0.535759530139958 | 0.192507024114801 | -2.7830648393404 | 0.00538480436005648 | 0.0280321649142292 |
| AC092384.2 | 5.24470885847324 | -0.653136239852528 | 0.205704051685284 | -3.1751257911623 | 0.0014977155957909 | 0.0106228218355121 |
| NDUFA5P11 | 3.38760548852593 | 0.517205572940615 | 0.170783511572901 | 3.02842802667072 | 0.00245829627007908 | 0.0154385622279783 |
| AC007938.1 | 4.7959395950287 | 0.587111406099727 | 0.120896947599537 | 4.85629635617016 | 1.19601661052954E-06 | 0.0000390597534075476 |
| AC009133.2 | 1.31657217347665 | 1.25576759094111 | 0.403351861680834 | 3.11333034563946 | 0.00184988831180896 | 0.0124488413884794 |
| IL21R-AS1 | 1.33329806240274 | -0.638455804277721 | 0.196029807126263 | -3.25693226778768 | 0.00112623303377153 | 0.00856767177329107 |
| LINC00261 | 1263.47659920947 | -1.07425666091321 | 0.189294682804588 | -5.67504931991241 | 1.38648703664529E-08 | 1.05379316998845E-06 |
| TGFBR3L | 8.04380800605964 | -0.693097284109283 | 0.178459000525065 | -3.88379001378491 | 0.000102840733650227 | 0.00134494210730811 |
| AC027601.1 | 49.7358541728842 | 0.518673655623657 | 0.108112034988423 | 4.79755704976971 | 1.60612471950574E-06 | 0.0000494464511984051 |
| LINC01992 | 1.51499396512708 | -1.53130777452201 | 0.452006314935275 | -3.38780172737473 | 0.000704551756669386 | 0.00602637326215147 |
| AC007608.2 | 3.35132637337947 | 1.39777768352985 | 0.322916979561982 | 4.32859766440852 | 0.0000150061785233235 | 0.000296608162904661 |
| RPL10P14 | 2.56616184628454 | 0.795336338305323 | 0.237157477828436 | 3.35362117015212 | 0.000797614822688105 | 0.00662374842322712 |
| AL032819.1 | 1.04800940476205 | 0.585388455599254 | 0.213784005466444 | 2.73822381764261 | 0.00617720201371743 | 0.0310223741917313 |
| AC012508.1 | 0.902448328567278 | 0.843157351706541 | 0.279584722960622 | 3.01574901081164 | 0.00256345355982843 | 0.015916682952935 |
| LINC01413 | 0.992182625023787 | -1.50067247995942 | 0.386441153641287 | -3.88331435671166 | 0.000103042210086215 | 0.0013465953848 |
| CCDC187 | 29.2022833881331 | -0.590796445027764 | 0.182419707597327 | -3.23866567274567 | 0.00120090252386879 | 0.00896841942903533 |
| FRRS1L | 5.32102364598731 | 0.613613767485288 | 0.206740342982295 | 2.96804077343452 | 0.00299704571571206 | 0.0179573730458706 |
| AC084262.1 | 2.13777831303986 | -0.685636917502206 | 0.235100810702218 | -2.91635284223091 | 0.00354149742982026 | 0.0203496146130669 |
| MRC1 | 475.211042611827 | -0.501364901122919 | 0.14776511841674 | -3.39298547921795 | 0.000691353029846339 | 0.00593739805447387 |
| AC104024.2 | 10.3170143830877 | -0.64943865387043 | 0.155872983075814 | -4.16646067237036 | 0.0000309365233358639 | 0.000525447871910951 |
| AC091544.4 | 10.0955624279113 | -0.813270438658005 | 0.212030798844243 | -3.83562408428895 | 0.000125245852450681 | 0.00157106969154376 |
| AC138811.2 | 3.56711181481367 | -0.770026010487907 | 0.154194660515805 | -4.99385651819624 | 5.91853751228024E-07 | 0.0000222390709534467 |
| AC007218.1 | 1.32708157382475 | 0.737789580407966 | 0.247987973259554 | 2.97510226286565 | 0.00292890890895468 | 0.0176325061626035 |
| AL360014.1 | 2.87906917805034 | 0.518963026408675 | 0.195217582245032 | 2.65838261308496 | 0.00785166978106355 | 0.0371948832283406 |
| AC092127.1 | 2.04857368917563 | 0.759304388122454 | 0.224687990607125 | 3.37937237353342 | 0.000726515307859866 | 0.00617122807352035 |
| SCX | 94.4332868881801 | 0.577916967840696 | 0.121514622455903 | 4.75594587845113 | 1.97519385671014E-06 | 0.0000587150515165338 |
| AL355607.2 | 0.772082288208219 | -0.802828656687115 | 0.278077140010978 | -2.88707175518067 | 0.00388845540165197 | 0.0218517492880991 |
| NBAT1 | 1.69330438266539 | 0.779274152660432 | 0.185101084385155 | 4.20999236848842 | 0.0000255379314448271 | 0.000453071354577139 |
| BMS1P8 | 1.03234788737613 | -1.14119423701572 | 0.284967070527796 | -4.00465301096747 | 0.000062108579481568 | 0.000900709069827666 |
| AL035071.2 | 3.30481838806857 | 0.533678872460346 | 0.164281797793663 | 3.24855753727899 | 0.00115991747754687 | 0.00875224735652579 |
| AC012531.1 | 3.89477986752521 | -2.10747332073937 | 0.292580810291913 | -7.20304697576273 | 5.88816753101554E-13 | 2.62549464762962E-10 |
| AL031705.1 | 3.52674983728871 | 0.581253357640457 | 0.138348024288211 | 4.2013853152653 | 0.0000265286627943426 | 0.000468411584566212 |
| AC237221.1 | 5.37299233152875 | -0.994147350751496 | 0.329258921791154 | -3.01934825438709 | 0.00253319158004678 | 0.0157932499664289 |
| HNRNPA3P11 | 3.97369979644726 | 0.531208731686343 | 0.151767897133662 | 3.50013897351761 | 0.00046501565748587 | 0.00434872864028033 |
| AC009133.3 | 2.5677425399359 | -0.521802945214611 | 0.183951002700458 | -2.8366409400024 | 0.00455908513669951 | 0.0247031376323378 |
| AC005307.1 | 19.2729208331727 | 1.06490089363322 | 0.289183750462771 | 3.68243683100828 | 0.000231015121592399 | 0.00252801298962468 |
| AC009093.3 | 22.6664665007166 | 0.858342932201605 | 0.186458841087697 | 4.60339089953852 | 4.15666990324807E-06 | 0.00010532737463765 |
| LINC00565 | 3.66619687030417 | -0.510832853293823 | 0.173650078010674 | -2.94173696404803 | 0.0032637702590542 | 0.0191544820605505 |
| LINC02555 | 1.1353654075087 | -0.845717408749124 | 0.306150911720349 | -2.76242002350017 | 0.00573746161502282 | 0.0294237373607718 |
| AC022167.3 | 2.77861454486617 | 0.529635100898079 | 0.145224490193222 | 3.64700953808407 | 0.00026531007948925 | 0.00283912362818576 |
| WFDC21P | 76.2924566029795 | -1.30076013205417 | 0.165601030855719 | -7.85478281948296 | 4.00465455219288E-15 | 3.61955831174146E-12 |
| AC009486.1 | 0.694850375887604 | -0.818291671908009 | 0.256041174486088 | -3.19593781566749 | 0.00139377147086921 | 0.0100259207418387 |
| LINC02473 | 13.3492942440092 | 0.939397665922774 | 0.176117228848678 | 5.33393394878996 | 9.61074946362232E-08 | 5.07745155706884E-06 |
| AC112484.3 | 9.64577340434132 | -0.857672498011142 | 0.182696502812693 | -4.69452061099637 | 2.67232667284165E-06 | 0.0000744732904943087 |
| AC004449.1 | 1.5972639851685 | -0.634079573647991 | 0.177608469433243 | -3.570097617931 | 0.000356848220926842 | 0.0035676287606085 |
| BCAP31P1 | 1.53728377510026 | 0.500852521823348 | 0.188496962387628 | 2.65708537410479 | 0.0078819479666508 | 0.0372720735153756 |
| AC092384.3 | 4.29969109239416 | -0.825298678471785 | 0.237816433768405 | -3.47031811634807 | 0.000519842283380382 | 0.0047498813226248 |
| AC137932.2 | 4.07850825391842 | -0.710716492473289 | 0.136779720295322 | -5.19606628043086 | 2.03549710746538E-07 | 9.27399104466721E-06 |
| AC112236.1 | 0.800365322933234 | -1.63305668524469 | 0.293216590565606 | -5.56945526886652 | 2.55536980754766E-08 | 0.0000017840642401672 |
| AC009061.1 | 7.29746134268162 | 0.719437194813815 | 0.164640568899562 | 4.36974434443738 | 0.0000124392082037354 | 0.000255366482964591 |
| AC134312.5 | 12.6924704331085 | -0.537249358998762 | 0.14550440968604 | -3.69232355334113 | 0.00022221450692721 | 0.00245392782109079 |
| AC010325.1 | 1.35395632525521 | -0.58470175266701 | 0.189976266152647 | -3.07776210422622 | 0.00208561338559897 | 0.0135874858131929 |
| AC010735.1 | 0.819820736465043 | -1.1389205941532 | 0.267937645149787 | -4.25069270694121 | 0.0000213110387123613 | 0.000392447002543385 |
| AL592164.1 | 0.936004166215065 | -0.605547428814652 | 0.217435699327711 | -2.78494943878556 | 0.0053536070351 | 0.0279001599607003 |
| AC109446.3 | 37.1132054662412 | 0.504108404470473 | 0.173110976885798 | 2.91205337488816 | 0.00359061274771595 | 0.0205432229918278 |
| AC009097.4 | 1.79487938171486 | 0.517902124636585 | 0.189424451048347 | 2.73408275315207 | 0.00625543207887387 | 0.0312985554948233 |
| DLGAP1-AS5 | 21.8187759101608 | -1.90355111950771 | 0.413832172378201 | -4.59981424007813 | 4.22867854288548E-06 | 0.000106889998360677 |
| AC026464.5 | 1.66102658974282 | 0.662378847190587 | 0.188185684663723 | 3.51981527380373 | 0.000431847453690749 | 0.00411304719150773 |
| AC027279.2 | 0.83766829766116 | 0.717110866955557 | 0.24489689912266 | 2.92821538175696 | 0.00340913796840676 | 0.0197519736554849 |
| TMEM249 | 21.992932156643 | 0.910280055655072 | 0.167574981150082 | 5.4320761333687 | 5.57021464815336E-08 | 3.28534600112071E-06 |
| LINC02177 | 21.0377699687345 | 0.58154883791241 | 0.137919898053736 | 4.21656951693676 | 0.0000248046845059797 | 0.000441938337372922 |
| AC093591.2 | 2.83088212230702 | -0.516122203537805 | 0.154274285316755 | -3.34548432668547 | 0.000821390013531408 | 0.00677110569997592 |
| LINC02582 | 3.96748229451624 | -3.0871537988113 | 0.43401183853369 | -7.11306357273861 | 1.13494967537458E-12 | 4.51845083855677E-10 |
| AC092718.6 | 1.68785364480986 | -1.31183996250146 | 0.226865949385064 | -5.78244538705474 | 7.36224740396843E-09 | 0.0000006186137630239 |
| LINC01996 | 13.2922294550953 | 0.790511764433021 | 0.212009720697018 | 3.72865810979836 | 0.000192502112987051 | 0.0021926620103927 |
| AC027796.1 | 1.69779587276231 | 0.542664007348443 | 0.185077220164087 | 2.9320950837025 | 0.00336683648422395 | 0.0195713098740514 |
| GFY | 1.48783065314952 | -1.43540870850222 | 0.306356662238983 | -4.68541698428115 | 2.79390526696273E-06 | 0.0000768032233503257 |
| AC005920.4 | 1.48922055518119 | 0.630010887667518 | 0.205035567269633 | 3.07269073389115 | 0.00212138215998282 | 0.0137673708896071 |
| AC144836.1 | 0.782860275476449 | -0.668660601501598 | 0.218114458163574 | -3.0656408893359 | 0.0021720401790036 | 0.0140172457866149 |
| AC108134.3 | 88.9771999210522 | 0.874245389760446 | 0.168026762417583 | 5.20301276523888 | 0.000000196083394976 | 8.99509039065481E-06 |
| AC099684.2 | 2.84804373942484 | 1.25057636978491 | 0.320324102645484 | 3.90409700505421 | 0.0000945778432399678 | 0.00126161636762306 |
| AC133065.2 | 0.96555054895426 | -0.911189959001498 | 0.262108889656884 | -3.47637945509708 | 0.000508232644845697 | 0.00466419212649007 |
| AC104581.1 | 0.869135471536453 | 0.71135561555404 | 0.240135734029513 | 2.96230637405516 | 0.00305343789352796 | 0.0182084646996009 |
| SPON1 | 1406.21960987093 | -0.926147426901525 | 0.156869257033889 | -5.90394475254921 | 3.54911058458981E-09 | 3.35280667146476E-07 |
| U91319.1 | 2.86562067295269 | 1.23902315345942 | 0.442095367853611 | 2.80261509971235 | 0.00506901251714851 | 0.0267631696555858 |
| C19orf84 | 2.56730297112285 | -0.539851692505516 | 0.172872771910082 | -3.12282661138975 | 0.00179123238385239 | 0.0121579852609685 |
| MTCO1P40 | 67.0807411171477 | -0.560455946864294 | 0.178302691591758 | -3.14328371524259 | 0.00167063826517419 | 0.0115408975137276 |
| ALOX12P2 | 18.1972527638237 | -0.672534868386168 | 0.255689389614363 | -2.63028070660461 | 0.0085314391307416 | 0.0394066078862855 |
| AC036164.1 | 3.36994912646021 | 0.941596961607239 | 0.298103064060794 | 3.15862892779664 | 0.00158513178236734 | 0.0111015658776814 |
| AC024361.2 | 2.77904253456004 | 0.553598396219635 | 0.143862135134976 | 3.84811747511067 | 0.000119028975773445 | 0.00151294831159846 |
| AC110285.3 | 5.80739859783013 | -1.14671811363309 | 0.244188976635543 | -4.6960273532109 | 2.65270034035217E-06 | 0.0000741116163592792 |
| LINC01975 | 1.40925326009893 | 0.56533250075639 | 0.216829512678101 | 2.60726731234077 | 0.00912680786716212 | 0.0414244978091938 |
| AC011825.2 | 2.36550738099731 | -0.55225307874673 | 0.145296904991808 | -3.80085920466005 | 0.000144195221697011 | 0.00175160792008407 |
| AC129492.2 | 0.795757385298441 | -1.53148206450436 | 0.499530083915669 | -3.06584550924246 | 0.00217055435110583 | 0.0140103606658331 |
| FAM72C | 7.14268613900365 | -0.828931244926078 | 0.167589091466131 | -4.94621241558317 | 7.56713845703264E-07 | 0.0000272400693520006 |
| LINC02563 | 7.68398123097882 | 0.899859564254275 | 0.255311833570139 | 3.52455094490192 | 0.000424201236012607 | 0.00406014245413097 |
| AP000829.1 | 0.739693444248796 | -1.25559355798364 | 0.467191023481317 | -2.68753784828199 | 0.00719809442775537 | 0.0347909631237166 |
| AC125421.1 | 0.711535600714676 | -1.30240898682565 | 0.501021939072588 | -2.59950490239303 | 0.0093358344983767 | 0.0421027121130086 |
| MIR4665 | 0.731037659135016 | 0.834433868741484 | 0.311884948608742 | 2.67545411365227 | 0.00746280883684173 | 0.035775184138628 |
| MSMB | 3.49690192098296 | -2.1640290589628 | 0.356225811054136 | -6.07488001096565 | 1.24080465101397E-09 | 1.47313037520922E-07 |
| AC005244.1 | 4.55855696535453 | 0.678947775683809 | 0.225894095264008 | 3.00560213798552 | 0.0026505556059995 | 0.0163209998261755 |
| LINC02864 | 8.19199901959391 | -2.41577963909143 | 0.405162891784898 | -5.96248987277438 | 2.48423052955875E-09 | 2.55623499598473E-07 |
| AP005899.1 | 6.95062818028282 | -0.620217811664737 | 0.130999071918646 | -4.73452065408454 | 2.19573411952598E-06 | 0.0000636992594131662 |
| RNU4ATAC | 3.30706757790262 | 0.786006665199488 | 0.208243466240135 | 3.77446015181628 | 0.000160354536417013 | 0.00190094874401196 |
| MYH4 | 10.8936956175099 | -0.989101140444862 | 0.210072743953503 | -4.70837445082255 | 2.49700087249808E-06 | 0.0000708267202528251 |
| MIR4449 | 1.08199731049821 | 0.978860236777269 | 0.334410008647787 | 2.92712601735626 | 0.00342110231183785 | 0.0198109962792176 |
| NPY4R2 | 4.10092587025243 | 0.672849960956702 | 0.168855184700499 | 3.9847752507579 | 0.0000675440234409892 | 0.000963244022137979 |
| AC005725.1 | 14.7144120578524 | 1.11607709681957 | 0.308853320819598 | 3.61361533642526 | 0.000301956942327646 | 0.00312826643907098 |
| AGAP12P | 10.942979548188 | 0.576322838144253 | 0.199810411254388 | 2.88434839068776 | 0.00392224567330085 | 0.0219785086807183 |
| FTLP12 | 1.34165350340867 | -0.685121034050473 | 0.179112371784818 | -3.82509051286287 | 0.000130724049545529 | 0.00162334707200207 |
| TSPOAP1-AS1 | 93.2161339305065 | 0.548511312702687 | 0.107077900033882 | 5.12254454494459 | 3.01439852744588E-07 | 0.0000128144968192902 |
| MIR4728 | 1.51858026590515 | 0.586022991163083 | 0.21709427108751 | 2.69939408454891 | 0.00694658635164223 | 0.0338542321147799 |
| ANXA8 | 2.59701590406857 | -1.44459835907555 | 0.274701751242197 | -5.25878831330014 | 1.45007697726656E-07 | 7.08968922130825E-06 |
| AC004687.1 | 59.5362439831799 | 0.675894853485611 | 0.164297642693922 | 4.11384388968237 | 0.0000389124638721256 | 0.000625931032617423 |
| AC129926.1 | 2.12596531470865 | -0.850978135029024 | 0.283699134006254 | -2.99957960044483 | 0.00270352470813903 | 0.0165862938313526 |
| AP001178.2 | 1.01959266633747 | -0.66678224023324 | 0.197918922543396 | -3.36896660341833 | 0.00075450570191832 | 0.00634374948898734 |
| FCGR1CP | 3.38644705002576 | -0.579262706877325 | 0.17965842957111 | -3.22424451922557 | 0.0012630550119029 | 0.00929916956606874 |
| RN7SL648P | 1.36376510725911 | 0.722370208168487 | 0.240206145353892 | 3.00729278638659 | 0.00263585757062451 | 0.016248543571765 |
| AP001099.1 | 0.926742432155771 | -1.26289538319871 | 0.285921269316303 | -4.4169340259945 | 0.0000100110769421115 | 0.000214197335315478 |
| LINC01029 | 0.942973382107757 | -2.06369608051928 | 0.693968535306709 | -2.97376030111683 | 0.00294174776658302 | 0.0176906903093094 |
| AC090912.1 | 3.52497113015707 | -0.720921257614581 | 0.126015696791553 | -5.72088458795005 | 1.05970865549172E-08 | 0.0000008377961431904 |
| AC002091.1 | 2.80434339234828 | -0.510828970836598 | 0.173691456435625 | -2.94101380297842 | 0.00327139955996691 | 0.0191698167311045 |
| ESRG | 2.41996503903156 | -1.0806963382585 | 0.395202266361467 | -2.73453982996659 | 0.00624675373211275 | 0.0312683637643039 |
| AC004805.1 | 3.2021003048267 | 0.877973840808258 | 0.186275623474298 | 4.71330507144645 | 2.43730875887061E-06 | 0.0000693689187354476 |
| RHOT1P1 | 2.83205784719028 | -0.578396138723074 | 0.188325334904952 | -3.07126037511093 | 0.00213157181559389 | 0.0138230412690246 |
| LINC01910 | 2.26600884049969 | -0.903676880712412 | 0.231224384433831 | -3.90822483072072 | 0.0000929767634248718 | 0.00124622401701586 |
| UBL5P2 | 13.4222171050951 | 0.920340858008316 | 0.209529972147435 | 4.39240672146285 | 0.000011210273182493 | 0.000234601974824113 |
| AC005224.2 | 1.75706794519574 | -0.656617885143978 | 0.209246227010139 | -3.13801541144233 | 0.00170095933328662 | 0.0116971996758731 |
| AC002091.2 | 1.69249731278145 | -0.614345902151693 | 0.187394228772399 | -3.27836084481476 | 0.00104411829935664 | 0.00804919413717954 |
| SNHG25 | 243.578537728976 | 0.672296255123642 | 0.205685799881379 | 3.26855940230857 | 0.0010809648421076 | 0.00829119868113819 |
| AP001178.3 | 2.95232662565075 | -0.518300549493193 | 0.142639155172699 | -3.6336484807812 | 0.000279441513989035 | 0.00294983684053703 |
| RN7SL220P | 0.472311846654211 | 1.10270558682562 | 0.406260031628421 | 2.71428518923119 | 0.00664189600442273 | 0.0327366670862056 |
| GDF10 | 39.016701210043 | 2.72709366034563 | 0.265113892765617 | 10.2864985003129 | 8.1071052799379E-25 | 6.77794536929208E-21 |
| MIR4754 | 1.4976704528014 | 0.737072146635921 | 0.223844420356904 | 3.29278766681211 | 0.000991993601384477 | 0.00776550796289786 |
| DSG1-AS1 | 3.26574658017634 | -0.82360321240915 | 0.205761824927976 | -4.00270172903764 | 0.0000626232319759084 | 0.000906992691094989 |
| AP005136.3 | 7.11297040622119 | -0.654086983884003 | 0.11216686345541 | -5.83137446955556 | 5.49726597953063E-09 | 4.81255415935768E-07 |
| AC090912.2 | 2.60906798552591 | -0.771769410516728 | 0.164282810700838 | -4.69780987569133 | 2.62966040008637E-06 | 0.0000737142523886744 |
| ZNF793-AS1 | 14.1260617683148 | 1.06311392873104 | 0.178347120778089 | 5.9609256605484 | 2.50813058038438E-09 | 2.55722264845166E-07 |
| AC008752.2 | 0.675596674483454 | 0.708352076485787 | 0.247368458423327 | 2.8635505148905 | 0.0041892201035763 | 0.0231143208552712 |
| AC023090.1 | 4.57651867425575 | -0.881794667946581 | 0.216225465720169 | -4.07812588128619 | 0.0000454001848251717 | 0.000711134885678404 |
| AP005264.1 | 1.24886728265584 | -0.823840981337306 | 0.266122665351867 | -3.09571896196074 | 0.00196336352020975 | 0.0129742683826262 |
| AC005256.1 | 14.328846744223 | -1.23522272519753 | 0.204032177664962 | -6.05405843006717 | 1.41241340653203E-09 | 1.61760031305631E-07 |
| AC020663.2 | 31.0605500667586 | 0.682941199224452 | 0.129251364180682 | 5.28382198171435 | 1.26516196915968E-07 | 6.37191966455391E-06 |
| AC011511.3 | 5.64820256811539 | 0.648246678943585 | 0.210143812631599 | 3.0847764244195 | 0.00203705217864861 | 0.0133679550546246 |
| AC008738.2 | 8.53525043456734 | 0.508984816705868 | 0.115638379973489 | 4.40152150888448 | 0.0000107494409580397 | 0.000227552087653558 |
| AC007993.1 | 0.928065907141801 | 0.787044183067384 | 0.275145582452147 | 2.86046454409009 | 0.00423020852089104 | 0.023286688618212 |
| AC005336.2 | 2.2832805114882 | 0.894258337951157 | 0.272622506390137 | 3.28020730860505 | 0.00103730834355952 | 0.0080133207727691 |
| SELENOKP1 | 1.46679267228948 | -0.537257972689635 | 0.164734411784835 | -3.26135849133551 | 0.00110879760579947 | 0.00846584692537574 |
| MIR924HG | 40.2204118963609 | -0.60599126373752 | 0.145007283871036 | -4.17904016653718 | 0.0000292741984702283 | 0.00050489311255357 |
| AC011498.4 | 2.54085403997649 | 0.649337203520306 | 0.182866669603596 | 3.55087783316603 | 0.000383948592234643 | 0.00376871406560344 |
| AC023043.2 | 0.690511906858651 | -0.793210751346458 | 0.266577261879732 | -2.97553792005081 | 0.00292475188448075 | 0.017610650435867 |
| AC068473.4 | 0.765143193464412 | -0.788368497688307 | 0.294051923725652 | -2.68105199823093 | 0.00733911109265672 | 0.0353143241957735 |
| AC005498.2 | 1.47139523415009 | 0.704630183329869 | 0.183453829427865 | 3.84091291812981 | 0.000122577596217011 | 0.00154688300856199 |
| AC020934.1 | 0.444874792120518 | -0.806305257906695 | 0.306923103127995 | -2.62705951324376 | 0.00861262694034483 | 0.0396781195948494 |
| LINC02594 | 2.13477994153693 | 0.761807849742515 | 0.182133172510501 | 4.1826968653862 | 0.000028807138157079 | 0.000498379883212124 |
| AC005884.2 | 1.27832767236058 | -0.627376578005222 | 0.198754188220373 | -3.15654519596641 | 0.00159650143729372 | 0.0111578267640494 |
| AC023043.3 | 0.754290218846216 | -0.7361764579355 | 0.263683895509608 | -2.79189010202057 | 0.00524011502321371 | 0.0274713789945623 |
| AC021683.1 | 7.29574383708319 | 1.29462007089856 | 0.219978728772158 | 5.88520571113699 | 3.97559196835775E-09 | 3.71373593871005E-07 |
| ZNF571-AS1 | 9.61882450731495 | 0.522855379496871 | 0.138411177319664 | 3.77755170949326 | 0.000158377628672785 | 0.00188553387613929 |
| AP005131.3 | 0.682172131439004 | 0.808069250435377 | 0.277598459972242 | 2.9109284342434 | 0.00360356545665749 | 0.0206000745301777 |
| AC021683.2 | 14.3313508557069 | 1.12390455180952 | 0.2146179130348 | 5.23676954974063 | 1.63411520025679E-07 | 7.82334762221706E-06 |
| ZNF285 | 36.4685745778068 | 0.619354641306263 | 0.178451707992098 | 3.47071287955219 | 0.000519078708504422 | 0.00474435760691136 |
| AP002414.4 | 24.5157007307943 | 0.682732632273841 | 0.169087993377859 | 4.03773572939709 | 0.0000539696020582422 | 0.000807178636865713 |
| AC015911.8 | 3.75246360172916 | -0.573895942630879 | 0.171613893410595 | -3.34411119767445 | 0.000825466440154559 | 0.00680013713322877 |
| AC016168.2 | 0.746354754048342 | -1.21776513547176 | 0.42540349044447 | -2.86261199737551 | 0.00420164737406111 | 0.0231561455971245 |
| AC010327.4 | 1.0447548218314 | -1.33577442042021 | 0.272437512588198 | -4.90304880458696 | 9.43605281616144E-07 | 0.0000326329346719825 |
| AC011511.5 | 3.70900420794233 | -0.559329546192766 | 0.132642310816822 | -4.21682600935079 | 0.0000247764994178395 | 0.000441907036550073 |
| AC007787.1 | 0.782860684443936 | 0.669231851129595 | 0.245338867707346 | 2.72778568428013 | 0.00637610189333462 | 0.0317353176837173 |
| AC125437.1 | 7.3479475411246 | -0.521302357924789 | 0.0884484369593572 | -5.89385607983476 | 3.77285805011916E-09 | 3.53422742050658E-07 |
| AP005264.5 | 3.61580084928191 | -0.795013183511193 | 0.249816653330606 | -3.18238665401972 | 0.00146066675597977 | 0.0104044820002117 |
| AC087645.2 | 3.93503165819196 | -0.714480958864587 | 0.129722742169656 | -5.50775405233236 | 3.63440689925384E-08 | 2.36693558416047E-06 |
| AC018755.1 | 0.434615440750294 | 0.78739051616596 | 0.243875021678487 | 3.22866405401705 | 0.00124369901299455 | 0.00919968643940803 |
| AC011483.1 | 0.949326299240545 | -1.11053922407015 | 0.303098827478195 | -3.66395090772841 | 0.000248354406476661 | 0.00268785374155097 |
| AL031666.2 | 6.4793889801604 | 0.55356977705075 | 0.121889646584712 | 4.54156519902638 | 0.0000055838111437756 | 0.000134147853642344 |
| AC074135.1 | 6.30623360229875 | 0.964004463898375 | 0.273051173444014 | 3.53049009729318 | 0.000414790542412595 | 0.00399637721675656 |
| AC008750.3 | 4.73053128643843 | 0.714281529838983 | 0.257694767049629 | 2.77181231895726 | 0.00557451670587693 | 0.0287751667082029 |
| ERFL | 11.2767038129287 | -0.90563584227981 | 0.127427784669684 | -7.1070516106623 | 1.18548113338851E-12 | 4.66410118385629E-10 |
| AC135012.2 | 0.876898618948545 | 0.686741353753857 | 0.194770802376995 | 3.52589477156136 | 0.000422054630106958 | 0.00404770603385056 |
| AC123912.1 | 1.72374418066416 | 0.608960855521497 | 0.236352979761663 | 2.57648901289745 | 0.00998093774689609 | 0.0442213195722972 |
| AC093063.1 | 1.92836388651507 | -1.07259926119448 | 0.29585036665096 | -3.62547889778321 | 0.000288426449652965 | 0.00302273811635677 |
| ZNF649-AS1 | 1.24157177642085 | -0.516405879650558 | 0.190462964633373 | -2.71131913043883 | 0.00670160917112362 | 0.0329581196912818 |
| SLC6A14 | 582.79589305909 | -0.958379778918802 | 0.188878728893972 | -5.0740482241216 | 0.0000003894402917319 | 0.000015756379950508 |
| AC007192.1 | 1.06934231937498 | -0.578831418213099 | 0.203102621985909 | -2.8499455721121 | 0.00437267110820463 | 0.0238978374245104 |
| SMIM17 | 4.67951728506555 | 0.545185287248995 | 0.154138007193092 | 3.53699452313556 | 0.000404708058393226 | 0.00392410753516563 |
| AC008763.1 | 9.24994250994278 | 0.505429499352833 | 0.140869533982245 | 3.58792625392327 | 0.000333318530805227 | 0.00337985688230605 |
| AIRN | 1.07403604106388 | 0.904968062249056 | 0.324552054118614 | 2.78836029772382 | 0.0052975590233488 | 0.0276978031435297 |
| AC006272.1 | 1.13117632618555 | -0.646327507745951 | 0.175416835785381 | -3.68452380783291 | 0.000229130609220519 | 0.0025123232241156 |
| LRRC2-AS1 | 3.10080375207188 | 0.697985178772361 | 0.145491726285914 | 4.79742179566081 | 1.60720927135537E-06 | 0.0000494464511984051 |
| AL132655.1 | 1.4054958505785 | 1.05694911163327 | 0.300855650187477 | 3.51314363208613 | 0.000442838017815233 | 0.00419529433194817 |
| BNIP3P27 | 1.10890988895859 | 0.809579014583716 | 0.243558445877989 | 3.32396198237065 | 0.00088748293476077 | 0.00716369884244983 |
| AC006262.1 | 7.77683207314416 | -0.606749683999033 | 0.195531149002926 | -3.10308453201977 | 0.0019151492888464 | 0.0127819025891028 |
| AC005523.1 | 1.11183644722278 | -0.562798615244597 | 0.191870122809905 | -2.93322695061903 | 0.00335458579229698 | 0.0195204555535054 |
| AL121761.1 | 40.3063888284121 | -1.45641200557559 | 0.19623496971336 | -7.4217760866117 | 1.15560063003848E-13 | 0.0000000000677992917 |
| AC008687.4 | 1.55465240702014 | -1.21995678649551 | 0.209851223468368 | -5.81343661634356 | 0.0000000061203152262 | 5.28877472337749E-07 |
| AC245128.3 | 7.3558302835554 | -0.921359520436694 | 0.204302099637497 | -4.5097897773518 | 6.48918987897266E-06 | 0.000150389111526406 |
| LINC02132 | 0.823021322056998 | -0.723700495128519 | 0.263895951953602 | -2.7423705811742 | 0.00609974815577721 | 0.0307164249097277 |
| Z69706.1 | 20.6396709377468 | 0.592923769898067 | 0.127709732581086 | 4.64274537198332 | 3.43809957087829E-06 | 0.0000904739119136537 |
| CSAG2 | 1.34427566437054 | -2.61722954841751 | 0.643181993400016 | -4.06918970878242 | 0.0000471769231190407 | 0.000729271791139372 |
| CSAG3 | 2.66180075545057 | -2.56042065505697 | 0.553869928407323 | -4.62278329935617 | 3.78625197765365E-06 | 0.0000977759371711918 |
| AL354861.3 | 4.65468134010623 | 0.60366324452794 | 0.162142929592243 | 3.72303156262214 | 0.00019684490303894 | 0.00223451705615351 |
| LINC01082 | 17.3006522070242 | 0.571505351150455 | 0.142331699789862 | 4.01530616155238 | 0.0000593686757898472 | 0.000868127352760852 |
| AC019171.1 | 3.5768166711013 | 0.646931754016296 | 0.181570287191219 | 3.56298249027378 | 0.000366665145006195 | 0.00363750097279655 |
| AC010422.3 | 117.338634985316 | 0.735193810005343 | 0.18875261634782 | 3.89501255257082 | 0.0000981936444802678 | 0.00129794144613009 |
| AC008739.2 | 1.19046366578039 | 0.667890245228904 | 0.233957879916095 | 2.8547456724622 | 0.00430713050633164 | 0.0236077745047627 |
| AC018755.3 | 12.7214444548822 | 0.750663638663152 | 0.162553609782874 | 4.61794505619301 | 3.87558830049815E-06 | 0.0000996213865835965 |
| SPIB | 72.7174605507669 | -0.654465500281227 | 0.166816703617163 | -3.92326119681155 | 0.000087358352087135 | 0.00118468694667395 |
| AC006272.2 | 2.36868276316255 | -0.652631858966008 | 0.183227985571027 | -3.56185686881887 | 0.000368241133742546 | 0.00364988737244168 |
| CYP4F23P | 15.8179047756821 | 0.70056588342558 | 0.20276748428674 | 3.45502083773395 | 0.000550250293658564 | 0.00497052009380586 |
| AC010320.4 | 1.47221730726169 | 0.614373120926178 | 0.237886444084562 | 2.5826319078013 | 0.00980498722221652 | 0.0436325193194098 |
| U62631.1 | 1.04469082594852 | -1.10260638056005 | 0.291580662281945 | -3.78147978652263 | 0.000155898882583666 | 0.0018646532301012 |
| AL589765.7 | 1.92562992498003 | 0.975562673539725 | 0.267929411592416 | 3.64111826223761 | 0.000271456398795411 | 0.00288558324491931 |
| CCDC194 | 1.96223162640096 | -0.745282756215234 | 0.238138556699149 | -3.12961817920473 | 0.00175033650196705 | 0.0119594752372358 |
| AC006262.2 | 0.841777350018155 | -0.965621347704161 | 0.291949601246016 | -3.30749329193454 | 0.000941349663480966 | 0.00746871066337615 |
| AL606834.1 | 12.1722497873896 | -0.649414116025398 | 0.104168365959455 | -6.23427381282114 | 4.53878599766082E-10 | 0.000000061451854791 |
| AC004817.3 | 1.43009429164874 | -0.534572876022009 | 0.200187255234112 | -2.67036418175994 | 0.00757690177382195 | 0.0362015260285282 |
| AC090181.2 | 17.6039016266164 | -0.570991283918591 | 0.10847735943936 | -5.26369084636303 | 1.41191796041269E-07 | 6.97449932527637E-06 |
| MEI4 | 0.758616275281141 | -1.10290179855552 | 0.346641692941105 | -3.18167670252783 | 0.0014642516993752 | 0.0104274926172286 |
| AL359881.1 | 6.33466390104833 | 0.72187143983187 | 0.187981848946201 | 3.84011245701952 | 0.000122977963923548 | 0.00154901283221518 |
| AC136475.7 | 5.97813970050846 | 0.727050086010995 | 0.169438034892912 | 4.29094970601202 | 0.0000177910608794925 | 0.000338641751622103 |
| AL359881.2 | 3.66827519834669 | 0.892291984208484 | 0.187391135154303 | 4.76165525905879 | 1.92011490060591E-06 | 0.0000575380667617051 |
| AC027243.2 | 0.681809668160314 | -0.830342571360941 | 0.258475850084425 | -3.21245706741937 | 0.00131604816644568 | 0.00958433858499055 |
| AL133467.4 | 1.42615681970834 | -2.1776062814574 | 0.61335415527825 | -3.55032449477663 | 0.000384756582080733 | 0.00377442933937925 |
| AC136475.8 | 0.440379552775837 | 0.789541562391536 | 0.267920810002411 | 2.94692137719512 | 0.00320954797141099 | 0.0189200957623703 |
| VTRNA2-1 | 1.01956745378316 | 1.3367070854992 | 0.323697703729871 | 4.12949202325728 | 0.0000363565615554189 | 0.000593669986101718 |
| AL359881.3 | 1.86249203348984 | 0.628091070096498 | 0.236526608690249 | 2.65547742630105 | 0.00791962338505484 | 0.0373551544771515 |
| AC004080.4 | 11.5217836514421 | 0.581871277803119 | 0.168750364445022 | 3.44811864387226 | 0.000564506011289627 | 0.00506797584685844 |
| STX8P1 | 1.24604938589049 | 1.01739417168838 | 0.279233613700614 | 3.6435232786093 | 0.000268931317404649 | 0.00286420417727588 |
| HEATR9 | 2.00313272071519 | -0.592211997910199 | 0.189369457039382 | -3.12728360301018 | 0.00176429646998289 | 0.012031321890124 |
| AP001554.1 | 4.98548139215776 | 0.619241542973677 | 0.201609286511481 | 3.07149315236733 | 0.00212991049459358 | 0.0138200362359718 |
| MICOS10P2 | 6.50495003045956 | 0.820131779983404 | 0.230054558456303 | 3.56494470479785 | 0.000363932906507748 | 0.00361575884118601 |
| RNVU1-31 | 3.58587554581867 | 1.26951343939516 | 0.259118442984981 | 4.89935577248257 | 9.61514009413217E-07 | 0.0000330812258259226 |
| AL355877.1 | 0.986449955057053 | -0.620685770569159 | 0.189777107055645 | -3.27060402700294 | 0.00107318063019261 | 0.00823905110994061 |
| SRGAP2D | 2.34291565204302 | -0.768994001863483 | 0.207596590144838 | -3.70427087134218 | 0.000211999631783342 | 0.00236457432242527 |
| RASL10B | 103.920981627014 | 0.847183792277556 | 0.163849158319739 | 5.1705104924881 | 2.33455364429244E-07 | 0.0000103885568562913 |
| AC087072.1 | 3.82595476731364 | 1.09501390693836 | 0.265457614584203 | 4.12500469671411 | 0.000037072719148811 | 0.000602183003861416 |
| AL645939.4 | 0.79201674913246 | -0.865569061415987 | 0.239141793616474 | -3.6194805112324 | 0.000295195041186966 | 0.00307727947860802 |
| AC108451.2 | 0.898555526436027 | -1.99298788350154 | 0.359508357098784 | -5.54364827450707 | 0.000000029623359624 | 2.01086703137084E-06 |
| AC136475.9 | 6.09815228718841 | 0.979322836675702 | 0.211070391939065 | 4.63979257194172 | 3.48759061466547E-06 | 0.0000913148824654747 |
| AC060764.1 | 0.783222819393586 | -0.683467688261268 | 0.238981057150669 | -2.85992411453083 | 0.00423742391805341 | 0.0233225692342894 |
| AC013244.1 | 0.897034641294334 | 0.632235782379824 | 0.238259631814808 | 2.65355812717465 | 0.00796480510693846 | 0.0375259245401854 |
| AC099791.2 | 2.44143898030732 | 0.653870770517372 | 0.162822722437773 | 4.01584472196297 | 0.0000592332477106881 | 0.000866905150958789 |
| LINC01050 | 4.81738052735819 | -0.54306880912488 | 0.208532082433745 | -2.60424584450896 | 0.0092076683476237 | 0.0416844246488739 |
| TMCC1-AS1 | 42.3638925182449 | 0.540694481871399 | 0.0912410256363542 | 5.92600179689304 | 3.10399190782312E-09 | 3.01754934248316E-07 |
| AC006237.1 | 1.02142909406048 | -0.658621458931288 | 0.217275817525434 | -3.03126904057876 | 0.00243528149033092 | 0.0153083991728659 |
| AC011247.2 | 0.736598807649756 | 0.832685646811841 | 0.252714140354601 | 3.29497053723801 | 0.000984319993972501 | 0.00771265914677329 |
| CCL5 | 617.747016333357 | -0.77684688736469 | 0.132397225208392 | -5.86754659051156 | 4.42290748609204E-09 | 4.05235266164082E-07 |
| BBIP1P1 | 0.804123160489997 | -0.679547572493978 | 0.221524008612233 | -3.0676023639654 | 0.00215783541039958 | 0.0139504828512215 |
| AL390208.1 | 8.75793150929273 | 1.0314437490913 | 0.176397536986714 | 5.84726842965494 | 4.99710775043157E-09 | 4.45635406373153E-07 |
| AC080013.3 | 2.7501746112338 | -0.501921546058394 | 0.136445999677969 | -3.67853617726423 | 0.00023457642139816 | 0.00255527839882647 |
| AL031055.1 | 3.85147472708763 | 0.515756894904719 | 0.119030176976845 | 4.33299275867708 | 0.000014709594994715 | 0.000292460330447835 |
| AL603832.3 | 0.984976936210207 | -0.623199725269682 | 0.186573877549954 | -3.34023033370695 | 0.000837089318139701 | 0.00687305204449493 |
| SMIM32 | 146.952186583532 | 0.703477978925569 | 0.180084114806274 | 3.90638552257836 | 0.0000936869969581023 | 0.00125273112845776 |
| AC016747.2 | 0.728905434114285 | 0.677346120485939 | 0.244702464638629 | 2.76803963330009 | 0.00563945971162277 | 0.0290413938521849 |
| AC016877.3 | 2.81474023822174 | -0.914441571965646 | 0.202894207626946 | -4.50698707795047 | 6.57546363480102E-06 | 0.00015192555106272 |
| AL136984.1 | 0.858253998259835 | 0.785201950023891 | 0.279108701115576 | 2.81324783815588 | 0.00490438349978928 | 0.0260792483701627 |
| AC016575.1 | 1.92712580108402 | 0.652326727988764 | 0.163335231165306 | 3.99379070476576 | 0.0000650252669769039 | 0.000934413023996284 |
| AC122710.2 | 0.890309332053931 | -0.768889733962824 | 0.219449587538453 | -3.50371920306341 | 0.000458808871610289 | 0.00430513083175962 |
| AC025181.2 | 110.158352271427 | 0.543085939719479 | 0.0743164161559114 | 7.3077520124237 | 2.71648530171227E-13 | 1.37643487060397E-10 |
| LINC01607 | 6.38343703176698 | 0.55885585371199 | 0.13433988247813 | 4.1600144603593 | 0.0000318227445878373 | 0.000535050892160108 |
| AL355385.1 | 5.68654310771379 | 0.621056582705901 | 0.119098288358244 | 5.21465582139837 | 0.000000184158843552 | 8.56556334640609E-06 |
| AL645939.5 | 0.916436613868269 | -0.842642138378309 | 0.285103962149532 | -2.95556095406475 | 0.00312101019582201 | 0.018503278832155 |
| LINC01749 | 3.4738443181304 | -0.863191628379345 | 0.270670011045614 | -3.18909222726516 | 0.00142720336002073 | 0.0102093122493718 |
| LINC02084 | 4.4542628986931 | -0.692135374147576 | 0.166322095074222 | -4.16141567864876 | 0.0000316280781225032 | 0.000533405422898604 |
| AC026801.2 | 26.2614209734333 | 0.570400146043677 | 0.12002542686166 | 4.75232757723175 | 2.01088232483682E-06 | 0.0000596698551084232 |
| AC011816.2 | 1.19178792752702 | 0.581176544243906 | 0.18508500780803 | 3.14005197464022 | 0.00168917863005445 | 0.0116329307550002 |
| FAM47E-STBD1 | 1.0247613526898 | -0.592747375041355 | 0.202349756620821 | -2.92932091908612 | 0.003397034956249 | 0.0197057490037952 |
| AC137630.3 | 8.27210462199783 | 0.588001363791143 | 0.161508956462341 | 3.64067341323108 | 0.000271925884424962 | 0.00288881612727808 |
| AL645608.6 | 1.85550480613404 | -0.733622505587692 | 0.256080485987148 | -2.86481222011001 | 0.00417256592574075 | 0.0230338312460585 |
| AL021807.1 | 10.9675648022871 | -0.6883232691394 | 0.132954420497202 | -5.17713714643947 | 2.25316587703273E-07 | 0.0000100870646934041 |
| SNORA28 | 1.49065231236759 | 0.757413338122847 | 0.212194717782232 | 3.56942597836084 | 0.000357764285028336 | 0.00357464990137963 |
| AC017048.3 | 1.59984789777383 | -0.622687473690484 | 0.20863921334548 | -2.98451793268312 | 0.00284025552308273 | 0.0172384437754869 |
| AC005162.2 | 20.1342922663949 | 0.667163300940351 | 0.212216949851434 | 3.143779520945 | 0.00166781046993014 | 0.0115285071797031 |
| AC010735.2 | 4.84999276861474 | -0.538015815412123 | 0.158697217172788 | -3.39020321211011 | 0.000698408317185862 | 0.0059856921946001 |
| AC005020.2 | 1.05149366228734 | -0.805841797524682 | 0.221963751140532 | -3.63051080811155 | 0.000282860858922755 | 0.00298122686545691 |
| KLHDC7B-DT | 15.4509733925011 | -0.672024731979387 | 0.132140596826066 | -5.08567955738809 | 0.0000003663120824811 | 0.000014975805210676 |
| AC009336.1 | 2.12720045956312 | -0.939309833471753 | 0.196483395761026 | -4.78060667586482 | 1.74766992420573E-06 | 0.0000533262569391313 |
| AP000864.1 | 1.21191911076246 | -1.00276429505118 | 0.231661846916473 | -4.3285690259248 | 0.0000150081296333148 | 0.000296608162904661 |
| AC092902.2 | 2.72793280215725 | 0.505614208626344 | 0.175935830418899 | 2.8738558110789 | 0.00405494040148935 | 0.0225370312292849 |
| AC009237.14 | 98.8311437819675 | 0.575363477462238 | 0.165747444408944 | 3.47132638764952 | 0.000517894098470851 | 0.0047359623847586 |
| RBAK-RBAKDN | 1.15212520655335 | -0.53611459011033 | 0.180149281779846 | -2.97594630860365 | 0.00292085994664045 | 0.0175975893091879 |
| AC012531.2 | 0.327752767343145 | -1.1372143772123 | 0.367597679287529 | -3.09363861985317 | 0.00197718162147488 | NA |
| PACERR | 1.96540962942747 | -0.866514841543228 | 0.220969315997638 | -3.92142609317075 | 0.0000880264484032577 | 0.00119229667375526 |
| AL355512.1 | 1.14906231080912 | -1.40254005638745 | 0.263659102313945 | -5.31952071473496 | 1.04040967825331E-07 | 5.42531828472182E-06 |
| LINC00653 | 27.7994383610329 | 0.543253665204639 | 0.11488971907258 | 4.72847935907517 | 2.26207617169493E-06 | 0.0000651036616938508 |
| SNURF | 3.2655582800461 | 0.612791151079805 | 0.16802729084104 | 3.64697394103394 | 0.000265346822658065 | 0.00283912362818576 |
| AC006946.2 | 2.66762529576247 | -0.729210173671965 | 0.246159643272892 | -2.96234656492236 | 0.00305303931096673 | 0.0182084646996009 |
| AC090912.3 | 1.29126273730639 | -1.31901965518455 | 0.217544761069808 | -6.06321038805108 | 1.33430938964086E-09 | 0.0000001554772634438 |
| AC010997.5 | 2.37118942141153 | 0.835168031020974 | 0.179948707755164 | 4.64114492090319 | 3.46484007813909E-06 | 0.0000910221381721348 |
| AL049839.2 | 1.15877237133611 | -0.766387941939236 | 0.26853619591411 | -2.85394652043243 | 0.00431797995452686 | 0.0236530525207678 |
| U62317.3 | 1.89517027475602 | -0.576899236067872 | 0.183878598229758 | -3.13739196198912 | 0.00170458081867585 | 0.0117172850438146 |
| AP001033.2 | 10.6965005372154 | -0.597084343774257 | 0.111678843043871 | -5.34644098649648 | 8.97005086338863E-08 | 4.80731475918978E-06 |
| AC009275.1 | 15.654404915222 | -0.594625002173455 | 0.130079180770704 | -4.57125420571049 | 4.84813723650876E-06 | 0.000119477822743792 |
| AL137796.1 | 0.974690511335946 | -0.545731265445562 | 0.19461383995467 | -2.80417500406279 | 0.00504455158548402 | 0.0266634888765223 |
| AC004832.6 | 1.91409042260249 | -0.623193958382115 | 0.18747661659675 | -3.32411566676906 | 0.000886993990732532 | 0.00716148069485209 |
| AL645608.8 | 1.83249120461107 | -1.34525901220116 | 0.22074695254699 | -6.09412268971097 | 1.10039243704417E-09 | 1.31897218206563E-07 |
| AC009093.5 | 1.73539900365657 | 0.741846616626495 | 0.216798519878946 | 3.42182509844034 | 0.000622023128032558 | 0.00545260745679288 |
| AL117379.1 | 27.3887371871424 | 0.513367787203339 | 0.115121414843075 | 4.45935960657819 | 8.22048985130661E-06 | 0.000181938862744802 |
| AC245041.1 | 2.89208257666609 | -1.51427022414567 | 0.322084459422241 | -4.70146938123616 | 2.58296000175927E-06 | 0.0000728774513526002 |
| AC083805.3 | 1.14106651125388 | 0.980361361516918 | 0.222966029485748 | 4.39690908869856 | 0.0000109803307618339 | 0.000231120017781701 |
| BX640514.2 | 5.95406378884968 | -0.541507795795385 | 0.181533536806884 | -2.98296284708782 | 0.00285472645518616 | 0.0172940686411624 |
| AL135999.3 | 15.6754089529428 | 0.704497514936734 | 0.148685569610735 | 4.73817006439251 | 2.15656796327029E-06 | 0.0000629318899019938 |
| AL136038.7 | 6.21975493908292 | 0.581050566843674 | 0.114313242518465 | 5.08296811500049 | 3.7158209702188E-07 | 0.0000151504841834691 |
| MIR8071-1 | 1.22626375521524 | -1.04674547566916 | 0.298151199786042 | -3.5107874005549 | 0.000446781559357754 | 0.00422308335444941 |
| RNVU1-27 | 3.16792779025975 | 0.834406946309021 | 0.208317043443922 | 4.00546653559644 | 0.0000618951964867429 | 0.000899174266250937 |
| AP005212.2 | 3.24641337039755 | -0.683410626750228 | 0.171749575433281 | -3.97911101105289 | 0.000069173438210473 | 0.000981042459980763 |
| AC138649.1 | 3.1258561201378 | 0.718040457726737 | 0.261178920788861 | 2.74922821320334 | 0.00597357795792188 | 0.0302496054010938 |
| AL121772.1 | 46.8245985460679 | 0.503800736698071 | 0.139345047706076 | 3.61549079060743 | 0.000299779132663217 | 0.00311148781952927 |
| AC078909.2 | 0.684732954206491 | -0.765700913808683 | 0.274088837109343 | -2.79362312556793 | 0.00521211829584777 | 0.0273460401709666 |
| AL121772.2 | 1.09838331071407 | 0.938017312726357 | 0.268442458331736 | 3.49429564367635 | 0.000475314342974875 | 0.00443140849115298 |
| MIR223HG | 30.0522427596118 | -0.533058797595891 | 0.162159440266508 | -3.28725109509389 | 0.00101170581051093 | 0.00786824784072241 |
| IGHV2-70 | 91.9613841860948 | -0.922790334980084 | 0.242277040034951 | -3.80882288658869 | 0.000139629925966462 | 0.00171106778459891 |
| AL049757.1 | 1.68304904053939 | -0.63517034750259 | 0.202213811048169 | -3.14108291718655 | 0.00168324369636621 | 0.0116016149410303 |
| AC012653.2 | 0.945441833947821 | -1.33595529265098 | 0.453384573696065 | -2.94662714648639 | 0.00321260313635641 | 0.0189280962096602 |
| AC120498.9 | 5.18512895838361 | 0.520941302828245 | 0.133396499385418 | 3.90520969611883 | 0.0000941437138992002 | 0.00125783223340673 |
| AL139384.1 | 59.0872358470273 | 0.512500009410779 | 0.118894365424687 | 4.31054918019156 | 0.0000162849604671712 | 0.000315894227345208 |
| KRT89P | 2.03034046790545 | -1.0614931498943 | 0.277825605711316 | -3.82071748634028 | 0.000133064006694768 | 0.0016458177716223 |
| AL157813.1 | 4.95304185864178 | 0.508927878937257 | 0.12390724735504 | 4.1073293919523 | 0.0000400260225283662 | 0.000640148371780785 |
| AC026356.1 | 26.4140442959851 | -0.515019750502424 | 0.0898554412317497 | -5.73164789402253 | 9.94595730528434E-09 | 7.93825069697658E-07 |
| AC020656.2 | 83.6759557058575 | -0.675071803514776 | 0.17637601891803 | -3.82745799375657 | 0.00012947347102966 | 0.00161020893200963 |
| MIR6750 | 0.625848505747515 | 0.788900746978719 | 0.273653119202601 | 2.88284946021262 | 0.00394095729976931 | 0.0220464192069062 |
| AC129492.6 | 2.6030757458664 | -1.5848819861345 | 0.31054613129349 | -5.10353157366067 | 3.3337286139316E-07 | 0.0000139159915360047 |
| AC027348.1 | 7.51893527250246 | 0.831049021267328 | 0.195914252159459 | 4.24190181218117 | 0.0000221633657801275 | 0.000404214041471014 |
| TRBV12-5 | 0.711899677276997 | 0.755911879796879 | 0.28382474326548 | 2.66330507728082 | 0.00773772267730912 | 0.0367668260549264 |
| LINC02826 | 10.1312487600116 | -1.62691886871873 | 0.305443326910143 | -5.32641811224558 | 0.0000001001684075152 | 5.26703126434474E-06 |
| AL161431.1 | 285.62648893825 | -0.66898344193639 | 0.208934130305475 | -3.20188683848969 | 0.00136530620855067 | 0.00986831326614142 |
| LINC02340 | 4.11592474524939 | -0.529288577192422 | 0.160525512513919 | -3.29722402939862 | 0.000976455812590953 | 0.00766837288436211 |
| CCL4 | 238.491102135432 | -0.670873543458349 | 0.128594353937878 | -5.21697510749528 | 1.81868689652262E-07 | 8.49448703819965E-06 |
| AL080312.2 | 1.67194944036343 | 1.12596560719963 | 0.231359718926529 | 4.86673139310475 | 1.13459065957847E-06 | 0.0000374930640687976 |
| H3C11 | 2.86720063946764 | 0.79678726732449 | 0.235899851392855 | 3.37765056917125 | 0.000731079164956306 | 0.0062021180706415 |
| CCL18 | 742.608094085818 | -0.598999964363251 | 0.190585160322546 | -3.14295175631462 | 0.00167253403574197 | 0.0115516074397528 |
| AC018695.6 | 1.88442712270623 | -0.77007670705813 | 0.217452383081914 | -3.54135786485284 | 0.000398073267071687 | 0.0038721251301371 |
| AL449403.3 | 0.800201048413758 | 0.968075019461246 | 0.309653249840881 | 3.12631958475715 | 0.00177009077251418 | 0.012060854125234 |
| AC009318.4 | 4.47389741000744 | -0.785628570975656 | 0.177573845781586 | -4.424235829988 | 9.67842514703613E-06 | 0.000207611221146365 |
| AC133552.5 | 21.6020390104175 | 0.599368107159566 | 0.107666407735066 | 5.56689983225249 | 2.5931135626123E-08 | 1.78801863424496E-06 |
| FLJ16779 | 24.983901259099 | 0.780393449986491 | 0.202056248300326 | 3.86225843818774 | 0.000112343628872424 | 0.00144262994487662 |
| AC025580.3 | 1.89227670978758 | 0.718024746424988 | 0.194170096061786 | 3.69791621360948 | 0.000217376610457992 | 0.00241111396581631 |
| RIMBP3 | 4.22377234659039 | -0.685792892814403 | 0.163460546390909 | -4.19546433654002 | 0.0000272313133861344 | 0.000477758982835574 |
| PRSS2 | 501.231678777656 | -1.58523608015762 | 0.284735051759063 | -5.56740756139505 | 2.58557160020852E-08 | 1.78650176558209E-06 |
| MIR6807 | 0.634226161331558 | 0.953117434054158 | 0.30198964406576 | 3.156126220827 | 0.00159879657477373 | 0.0111715326062648 |
| AC109809.1 | 1.92382659369051 | 0.607149272575942 | 0.169627486348212 | 3.57930949545277 | 0.000344503278004495 | 0.00346596829814269 |
| CCL4L2 | 121.501439416763 | -0.710816113973169 | 0.14307854496319 | -4.96801329756355 | 0.0000006764232529655 | 0.0000249404039974336 |
| CCL3L3 | 84.5783336183804 | -0.652362326474504 | 0.164827298971352 | -3.95785364770122 | 0.0000756262691811164 | 0.00105203564640387 |
| AC234782.3 | 1.05095473144654 | 0.640709138468063 | 0.239151550196765 | 2.67909255842544 | 0.00738219845882354 | 0.0354757121511678 |
| H2BC3 | 1.16699275740154 | 2.00269105142898 | 0.416993940358728 | 4.80268622058661 | 1.56551109772981E-06 | 0.0000485206877945136 |
| PCDHGB9P | 14.8359624498632 | 0.715863947431301 | 0.204981898623438 | 3.49232762618899 | 0.000478830537589269 | 0.00445574615609929 |
| IGKV1D-13 | 58.5491281771286 | 0.831302579015389 | 0.258443370147631 | 3.21657537022723 | 0.00129730452822403 | 0.00948055819008379 |
| AL117335.1 | 10.7635143110845 | 0.617066774844948 | 0.196296558665608 | 3.14354351925305 | 0.00166915594042979 | 0.0115354232196431 |
| AC137834.2 | 1.30530039749606 | -0.757168794692076 | 0.187792745857856 | -4.0319384608455 | 0.0000553186741255253 | 0.000821477397915549 |
| AC245884.12 | 2.87884422974105 | -0.707911348070898 | 0.218117772393484 | -3.24554638671913 | 0.00117225464637021 | 0.00881748535400644 |
| IGHV4-4 | 17.2223409320368 | -1.24969597716696 | 0.312121242953211 | -4.00387991968332 | 0.0000623120022609631 | 0.000903267438062908 |
| TRBV15 | 2.30003619850018 | -0.653285999484743 | 0.203149971952323 | -3.21578188373101 | 0.00130089664957661 | 0.00950089228109651 |
| AC023510.2 | 1.47013678703674 | -0.563168485313322 | 0.210937604477628 | -2.66983445985351 | 0.00758886509848225 | 0.0362397296335061 |
| AC245041.2 | 6.60050038088227 | -1.57392203826126 | 0.257439786522633 | -6.11374822641443 | 0.0000000009731780358 | 1.18345526811165E-07 |
| AC002401.4 | 1.86698415419205 | -0.92152995295337 | 0.260241678773907 | -3.54105444329683 | 0.000398531334579548 | 0.00387545358854586 |
| AC015922.3 | 41.9559975832983 | 0.523107007073118 | 0.12464397577057 | 4.1968093831987 | 0.0000270701583455258 | 0.000475712157325839 |
| AC099778.2 | 2.14840822022797 | 0.572947407790424 | 0.147680978090391 | 3.87962901653955 | 0.000104615900578908 | 0.00136183921648885 |
| AL121772.3 | 28.3706918854118 | 0.681376031846552 | 0.127743017195168 | 5.33395912205152 | 9.60941647477641E-08 | 5.07745155706884E-06 |
| MIR8071-2 | 2.675465352854 | -0.850784977048301 | 0.249755027448019 | -3.40647788251379 | 0.000658068821272115 | 0.00568953917295297 |
| AL359736.2 | 0.890096650352711 | 1.03595553098768 | 0.267049667617944 | 3.87926163783802 | 0.000104774013454802 | 0.00136336675406829 |
| AL122125.1 | 1.43690754865274 | -0.639868206788805 | 0.202053120313897 | -3.16683160247536 | 0.00154109531554788 | 0.0108499599036952 |
| AC243829.4 | 3.34223997835632 | -1.00816106495604 | 0.19446664288379 | -5.18423648398407 | 2.16901577790117E-07 | 0.0000097757716502117 |
| AL035420.3 | 2.21632355655609 | 0.514001068041807 | 0.166497025492668 | 3.08714865338206 | 0.00202086509638895 | 0.0132799706334131 |
| AL109976.1 | 12.1027003034892 | -0.531044009501333 | 0.148381917713908 | -3.5788997586972 | 0.000345043772221179 | 0.00347036205432201 |
| AC007996.1 | 62.2371804527112 | -0.785252249451408 | 0.104977105290255 | -7.48022387624647 | 7.41961057566351E-14 | 4.51139303402435E-11 |
| RNVU1-4 | 1.99659041220315 | 0.53111110456457 | 0.196288078750675 | 2.70577361572317 | 0.00681454838677946 | 0.0333956810009787 |
| CCL3 | 179.449930164639 | -0.58593690952967 | 0.135676218304708 | -4.31864122431351 | 0.0000156992750824102 | 0.000306846965111608 |
| AL138960.1 | 5.17476390237392 | 0.824863301001267 | 0.256368481265516 | 3.21749107741123 | 0.00129317049194134 | 0.00945571132028819 |
| SNORD30 | 2.25364934074347 | 0.805358738870151 | 0.29779508470541 | 2.70440574822396 | 0.00684266785856049 | 0.033472355013051 |
| MIR8075 | 1.00947670312423 | 2.28492090817481 | 0.53195962432827 | 4.29529010037197 | 0.0000174465041163451 | 0.000333074766151335 |
| SENP3-EIF4A1 | 5.83045699434539 | -0.720564350245234 | 0.13763811857581 | -5.23520924073337 | 1.64798062603734E-07 | 0.0000078395118201907 |
| AC064801.1 | 1.92097678976634 | -0.576853919819527 | 0.141343693634645 | -4.08121441421094 | 0.0000448010016443805 | 0.000704720177324259 |
| MIR6859-1 | 2.44561175659849 | 0.567060670393458 | 0.201787513635413 | 2.81018711305406 | 0.00495127052034105 | 0.0262826013874993 |
| AC005911.1 | 7.17023352212372 | -0.710302895533705 | 0.119384993030706 | -5.94968326840725 | 2.68661869187738E-09 | 2.6981640162249E-07 |
| AC099518.6 | 3.08257759223202 | -0.596114666977929 | 0.154257734422665 | -3.86440698879045 | 0.000111359412727156 | 0.00143178834310709 |
| AC026120.1 | 1.44241628468336 | -0.590116044280676 | 0.220010045469179 | -2.68222318222895 | 0.00731346504688581 | 0.035221327491065 |
| ZBTB2P1 | 1.06306109075568 | -0.584802616397301 | 0.193351773667174 | -3.02455263432934 | 0.00249001140721914 | 0.0155967337479346 |
| C17orf78 | 27.6536590323687 | -0.811767018956379 | 0.280133203018727 | -2.89778937380055 | 0.00375802876682891 | 0.0212793765696397 |
| ZNF426-DT | 13.0429784212257 | 0.507902204060134 | 0.130450818824027 | 3.89343822168931 | 0.0000988333803132311 | 0.00130330674465105 |
| H4C2 | 4.047520442846 | 0.86785238470869 | 0.216957829522461 | 4.00009710006269 | 0.0000633164988662361 | 0.000914414716959718 |
| AC000403.1 | 14.2419044391187 | -0.592477539810428 | 0.149086532457978 | -3.97405137836596 | 0.0000706603102589608 | 0.000996215048769041 |
| AC007608.4 | 20.6932504028359 | 1.52166281837509 | 0.234231576948526 | 6.49640342347814 | 8.2262881923081E-11 | 1.50329797665119E-08 |
| BANCR | 1.82401555045679 | -0.822649614828292 | 0.223842343140549 | -3.67512957238727 | 0.000237728733370749 | 0.00258708893634383 |
| AC079228.1 | 2.0716917017894 | -0.830623112341989 | 0.191447465863147 | -4.33864772561547 | 0.0000143362105398972 | 0.000286912958034256 |
| AC122713.2 | 1.58048466775423 | -0.637445480897254 | 0.187537507837455 | -3.39902928351671 | 0.000676254754008135 | 0.0058271866744499 |
| AC026310.3 | 0.952496250805143 | -0.662768235743722 | 0.2332425573543 | -2.84154076880988 | 0.0044896111203858 | 0.0244216431070665 |
| AC073592.2 | 2.97123561894487 | -0.606689714036621 | 0.186655947485889 | -3.2503101144553 | 0.00115279224451303 | 0.00871565394842945 |
| AC008985.1 | 1.93927518624897 | 0.672899214815493 | 0.198285376141146 | 3.39358972361382 | 0.000689829565099837 | 0.00592583619729482 |
| AC098476.1 | 19.0350820908149 | -0.761916942274333 | 0.185520860339482 | -4.10690712020262 | 0.0000400992377190833 | 0.000640706501577441 |
| C18orf15 | 0.614718754646364 | 1.13321619569978 | 0.31330305486029 | 3.61699695588706 | 0.00029804080756348 | 0.00309782735310458 |
| LINC01451 | 26.401585424135 | 0.633055988320764 | 0.181799935621771 | 3.48215738446584 | 0.000497391263879309 | 0.00458988925128362 |
| AL118508.2 | 1.32395082478347 | -0.942396979533991 | 0.214268857077017 | -4.39819856413036 | 0.0000109153093816718 | 0.000230012461463055 |
| AL121753.2 | 73.8728765046383 | 0.545299817611387 | 0.0916519900558057 | 5.94967787692728 | 2.68670718677977E-09 | 2.6981640162249E-07 |
| AC068860.1 | 2.81279234948035 | 0.595892697451182 | 0.153507895353648 | 3.8818374525843 | 0.000103670166492836 | 0.00135181360418807 |
| AC092135.1 | 1.04294267596138 | 1.15256118778884 | 0.320877579053552 | 3.59190315256175 | 0.000328271852574678 | 0.00334086040590456 |
| PRAL | 3.88402852169912 | -0.839752940626761 | 0.160381741987593 | -5.23596345955468 | 1.64126424166265E-07 | 7.82334762221706E-06 |
| AC013643.3 | 1.12595028399838 | 0.748188077078567 | 0.249568587152596 | 2.997925682935 | 0.00271823949440249 | 0.0166611739684399 |
| AC002525.1 | 2.93223114750215 | -0.558629275411819 | 0.17254350946493 | -3.23761396267045 | 0.0012053378764239 | 0.00898949805159861 |
| AC127071.1 | 3.3242673651048 | -1.08345079447954 | 0.296849668852751 | -3.6498298908899 | 0.000262414032251881 | 0.00281361015279493 |
| AC008957.3 | 1.60585957583686 | -0.789737521332229 | 0.239674461253229 | -3.29504243882634 | 0.000984068170482463 | 0.00771265914677329 |
| AC099494.1 | 1.09889711803296 | 0.775018204999136 | 0.26056945757745 | 2.97432481997156 | 0.00293634063925227 | 0.0176645266518932 |
| AC004009.2 | 1.90641440632514 | -1.70775849768583 | 0.324928830999089 | -5.2557924528729 | 1.47388433431412E-07 | 7.18507870380946E-06 |
| AL513497.1 | 11.296025496554 | -0.588782595121118 | 0.11845293715402 | -4.97060359386063 | 6.6744787645364E-07 | 0.0000246638584357598 |
| AC127071.2 | 0.818415603973958 | -1.39637211962128 | 0.372098456605674 | -3.75269527414639 | 0.000174943461723183 | 0.00203777751548126 |
| AC138466.4 | 1.04381473842881 | 0.901159638066195 | 0.344356346943668 | 2.61693924350293 | 0.00887221317150075 | 0.0405999661851845 |
| AL845472.1 | 5.74116302801506 | 0.650127537493798 | 0.178732023819814 | 3.63744293607515 | 0.00027535817176956 | 0.00291686030418676 |
| LINC00552 | 3.45294059391087 | 0.958383664263746 | 0.230781644374119 | 4.15277249134301 | 0.0000328471263403039 | 0.000548414178268819 |
| Z95114.3 | 4.03856638302918 | -0.700203450527508 | 0.158160583233295 | -4.42716785821834 | 9.54784308343381E-06 | 0.000205469091632042 |
| AC063926.3 | 2.58577589852693 | -0.881171475461641 | 0.247712552990779 | -3.55723383745693 | 0.000374780520462208 | 0.00369935364973352 |
| AL138688.2 | 1.07759854038029 | 0.570616665702457 | 0.215264631971949 | 2.65076831467983 | 0.00803089100590565 | 0.0377633236806742 |
| AC011676.5 | 3.81117869752907 | -0.644777082706456 | 0.173215892904426 | -3.72238985635238 | 0.000197346003854223 | 0.00223790960906729 |
| AC016168.3 | 0.733533527323211 | -1.03311116248075 | 0.360842222555956 | -2.86305509139952 | 0.00419577603488927 | 0.0231352254177687 |
| AC005274.1 | 1.95793621810542 | 0.767140645178295 | 0.186780173237026 | 4.10718456827206 | 0.0000400511183407261 | 0.000640243546630288 |
| GGT4P | 17.439097545899 | 0.866043780722162 | 0.212847906680633 | 4.068838609823 | 0.0000472480591527275 | 0.000729271791139372 |
| AC012254.5 | 1.82428736200319 | -0.588665943902618 | 0.201502907000653 | -2.92137693031252 | 0.00348487927535266 | 0.0201037317105992 |
| UCKL1-AS1 | 46.3624594583841 | 0.560479655919623 | 0.124890965737564 | 4.48775179701443 | 7.19787161712629E-06 | 0.000163749131033971 |
| AC126564.1 | 3.35305685801941 | -0.851725451316858 | 0.261569330767972 | -3.25621298497104 | 0.00112909020154894 | 0.00857694193687167 |
| AL161658.1 | 1.28562295941248 | -1.65759239863946 | 0.384267921833377 | -4.3136371902472 | 0.0000160590408487247 | 0.000312309375498168 |
| AC009084.2 | 12.9574244061687 | 0.779528585536617 | 0.141666099131923 | 5.5025767654596 | 3.74280170625262E-08 | 0.0000024131085318574 |
| LINC02204 | 2.23869303388976 | 0.623765036301576 | 0.194719207754738 | 3.20340783785054 | 0.00135811501308771 | 0.00982224962536314 |
| FP671120.6 | 1.27448807145023 | 2.23796568861251 | 0.47180038042081 | 4.74345884718537 | 2.10099640628393E-06 | 0.0000615788972996908 |
| AC025048.6 | 1.10323725854164 | -0.563413892459345 | 0.209835734399457 | -2.68502356889696 | 0.00725246917149999 | 0.0350082381687793 |
| LINC01127 | 5.85932456621271 | -1.14791988252427 | 0.191758870311593 | -5.98626744441597 | 2.14711213346106E-09 | 2.25797874110707E-07 |
