# Supplementary figures and images for: Abnormal Expression of N6-Methyladenosine RNA Methylation Regulator IGF2BP3 in Colon Cancer Predicts a Poor Prognosis
Source: Dis Markers. 2022 May 30;2022:5883101. doi: 10.1155/2022/5883101 (PMC9170420; doi:10.1155/2022/5883101)

Age 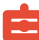 <60 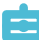 >=60

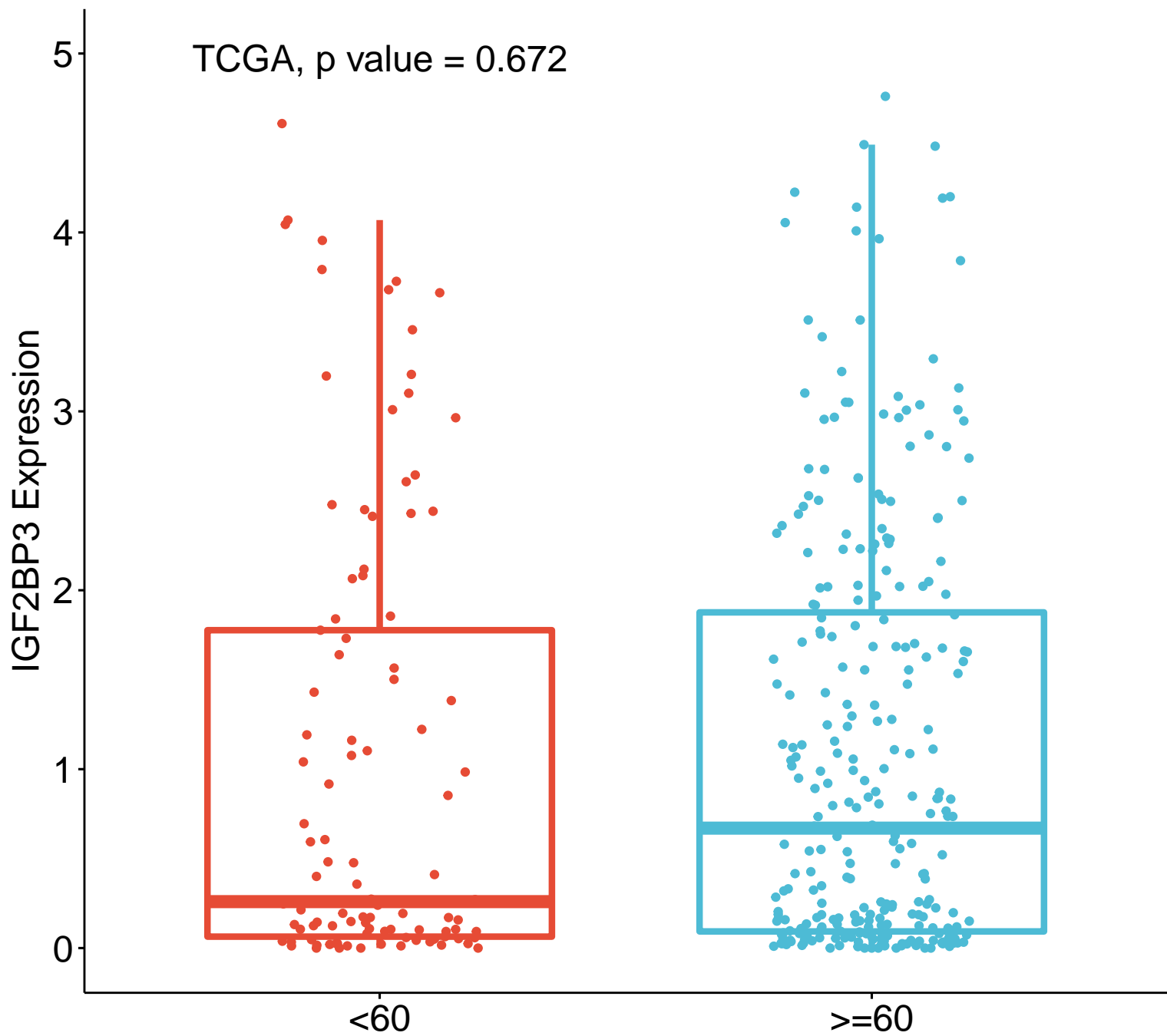

Supplement: Supplementary Materials — See Figures S1 in the Supplementary Material for correlations between expression of IGF2BP3 and clinicopathological features in TCGA database, Figure S2 and Table S4 for the DEGs in groups with different expression of IGF2BP3, Table S1 and Table S2 for the clinical information of the samples, Table S3 for gene coexpressed with IGF2BP3, Table S5 for the genes related to IGF2BP3, and Table S6 for the results of KEGG enrichment analysis. [file 5883101.f1.zip › Figure S1 A.pdf]

CA19.9 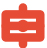 High CA19-9 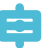 Low CA19-9

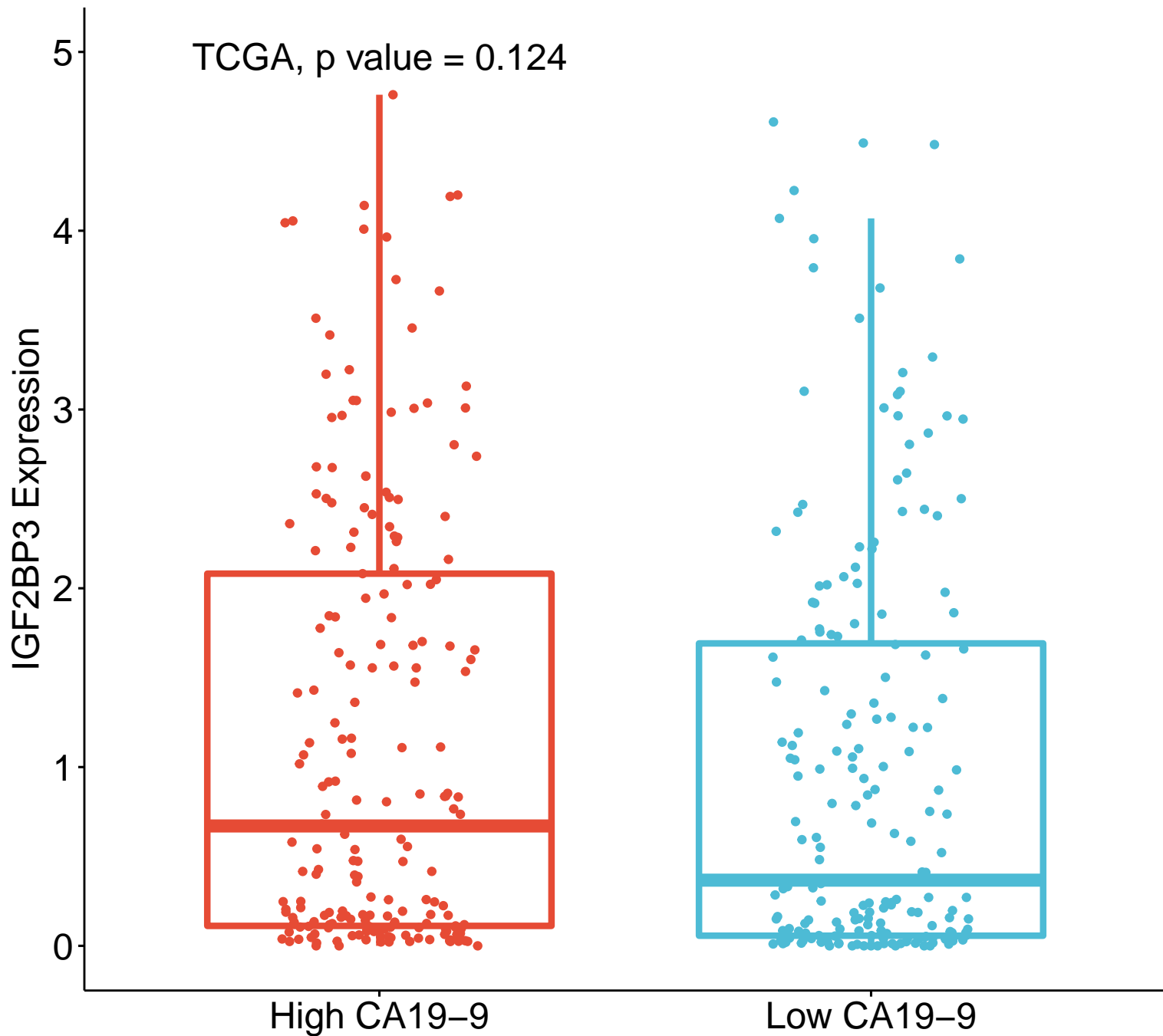

Supplement: Supplementary Materials — See Figures S1 in the Supplementary Material for correlations between expression of IGF2BP3 and clinicopathological features in TCGA database, Figure S2 and Table S4 for the DEGs in groups with different expression of IGF2BP3, Table S1 and Table S2 for the clinical information of the samples, Table S3 for gene coexpressed with IGF2BP3, Table S5 for the genes related to IGF2BP3, and Table S6 for the results of KEGG enrichment analysis. [file 5883101.f1.zip › Figure S1 B.pdf]

Gender Female Male

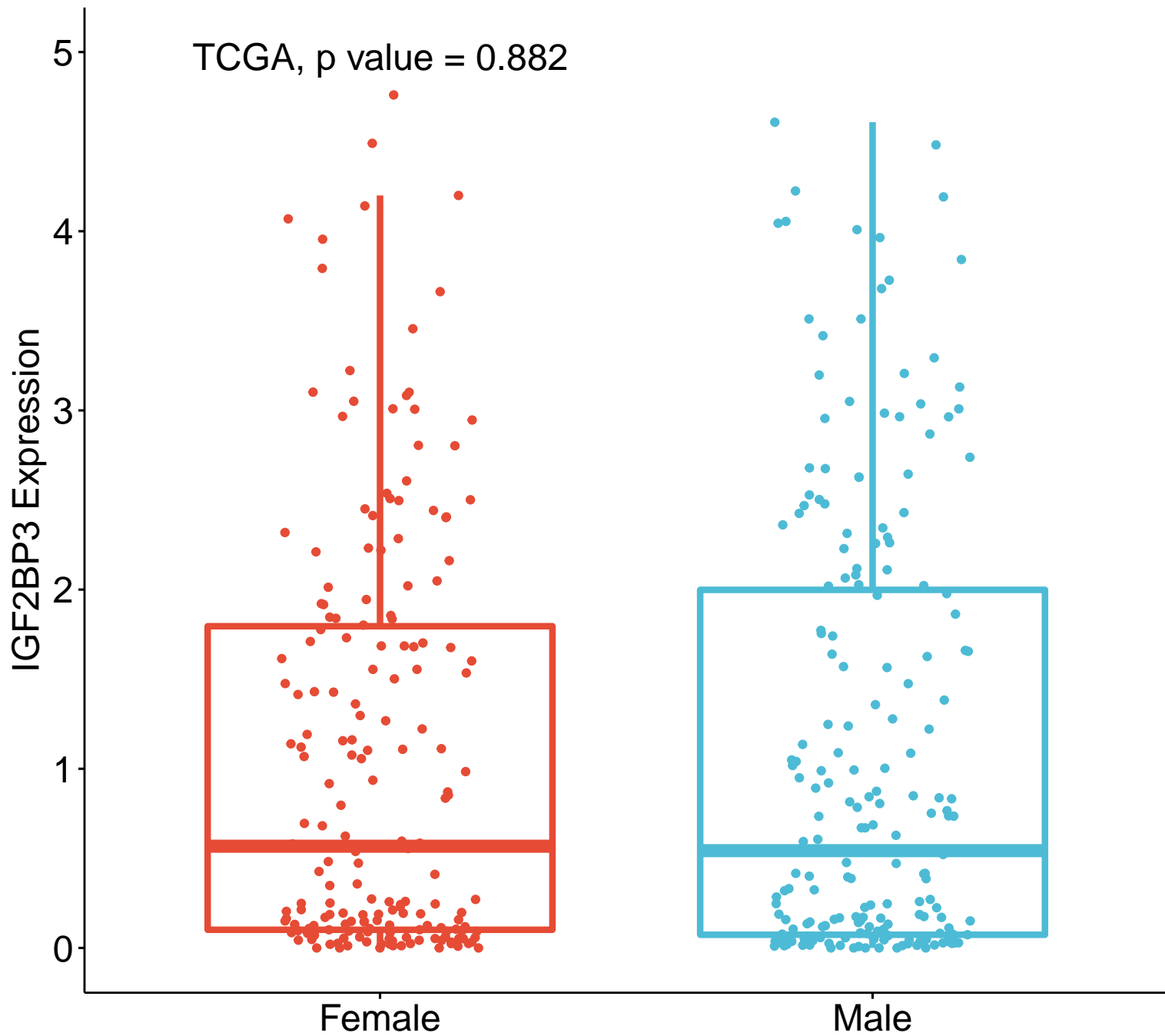

Supplement: Supplementary Materials — See Figures S1 in the Supplementary Material for correlations between expression of IGF2BP3 and clinicopathological features in TCGA database, Figure S2 and Table S4 for the DEGs in groups with different expression of IGF2BP3, Table S1 and Table S2 for the clinical information of the samples, Table S3 for gene coexpressed with IGF2BP3, Table S5 for the genes related to IGF2BP3, and Table S6 for the results of KEGG enrichment analysis. [file 5883101.f1.zip › Figure S1 C.pdf]

M M0 M1

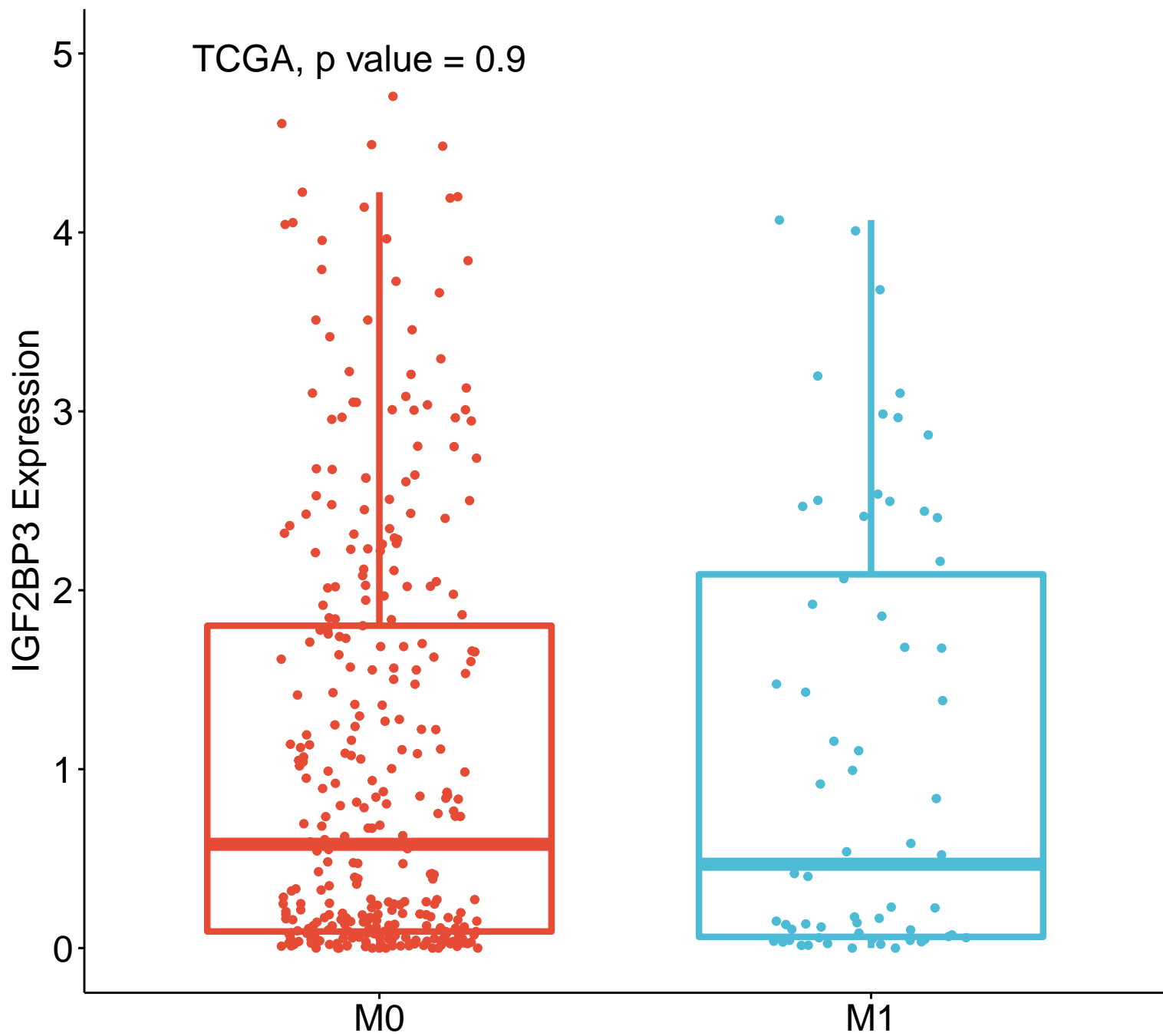

Supplement: Supplementary Materials — See Figures S1 in the Supplementary Material for correlations between expression of IGF2BP3 and clinicopathological features in TCGA database, Figure S2 and Table S4 for the DEGs in groups with different expression of IGF2BP3, Table S1 and Table S2 for the clinical information of the samples, Table S3 for gene coexpressed with IGF2BP3, Table S5 for the genes related to IGF2BP3, and Table S6 for the results of KEGG enrichment analysis. [file 5883101.f1.zip › Figure S1 D.pdf]

N 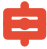 N0 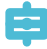 N1-2

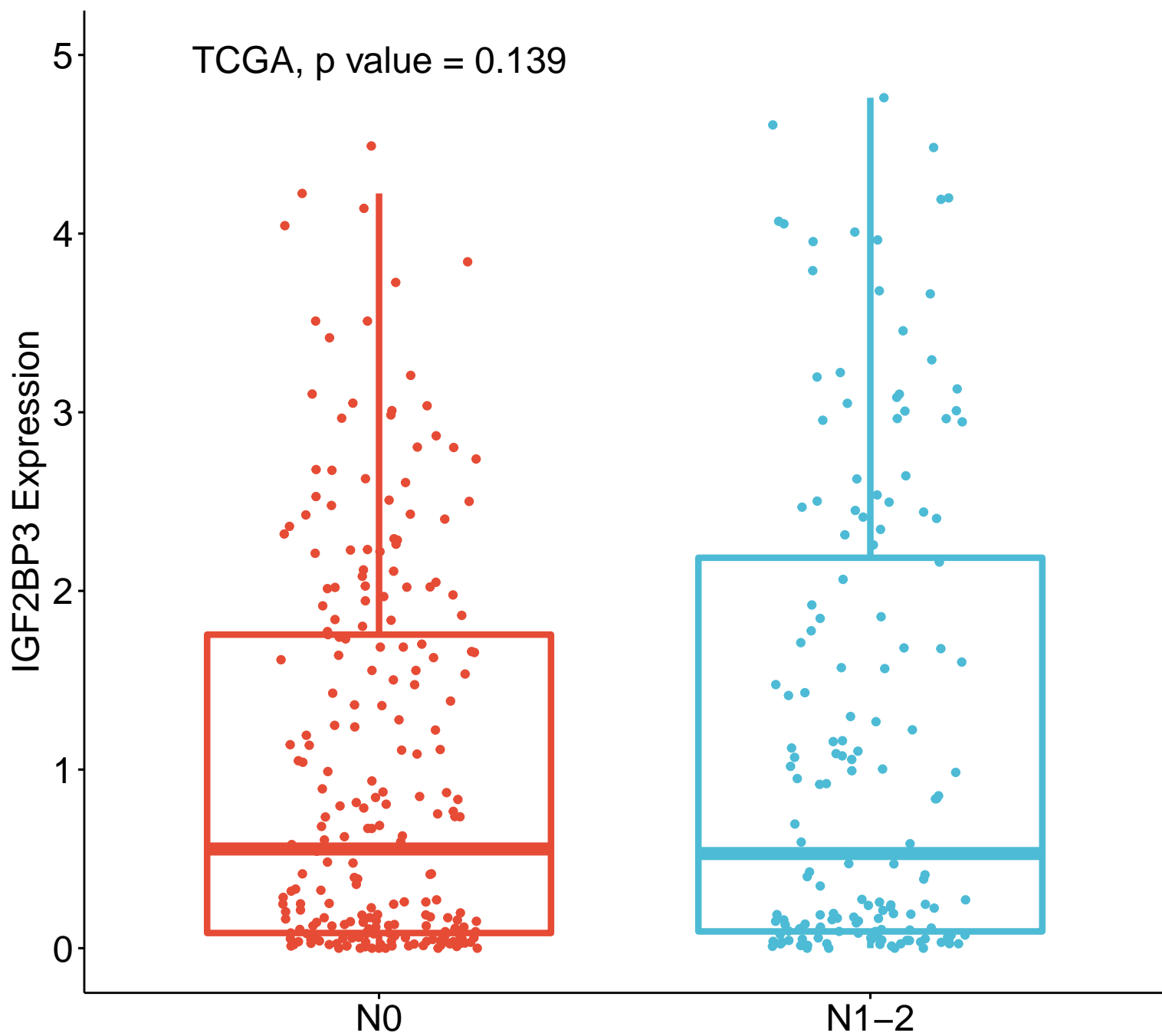

Supplement: Supplementary Materials — See Figures S1 in the Supplementary Material for correlations between expression of IGF2BP3 and clinicopathological features in TCGA database, Figure S2 and Table S4 for the DEGs in groups with different expression of IGF2BP3, Table S1 and Table S2 for the clinical information of the samples, Table S3 for gene coexpressed with IGF2BP3, Table S5 for the genes related to IGF2BP3, and Table S6 for the results of KEGG enrichment analysis. [file 5883101.f1.zip › Figure S1 E.pdf]

Stage 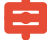 Stage I & Stage II 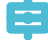 Stage III & Stage IV

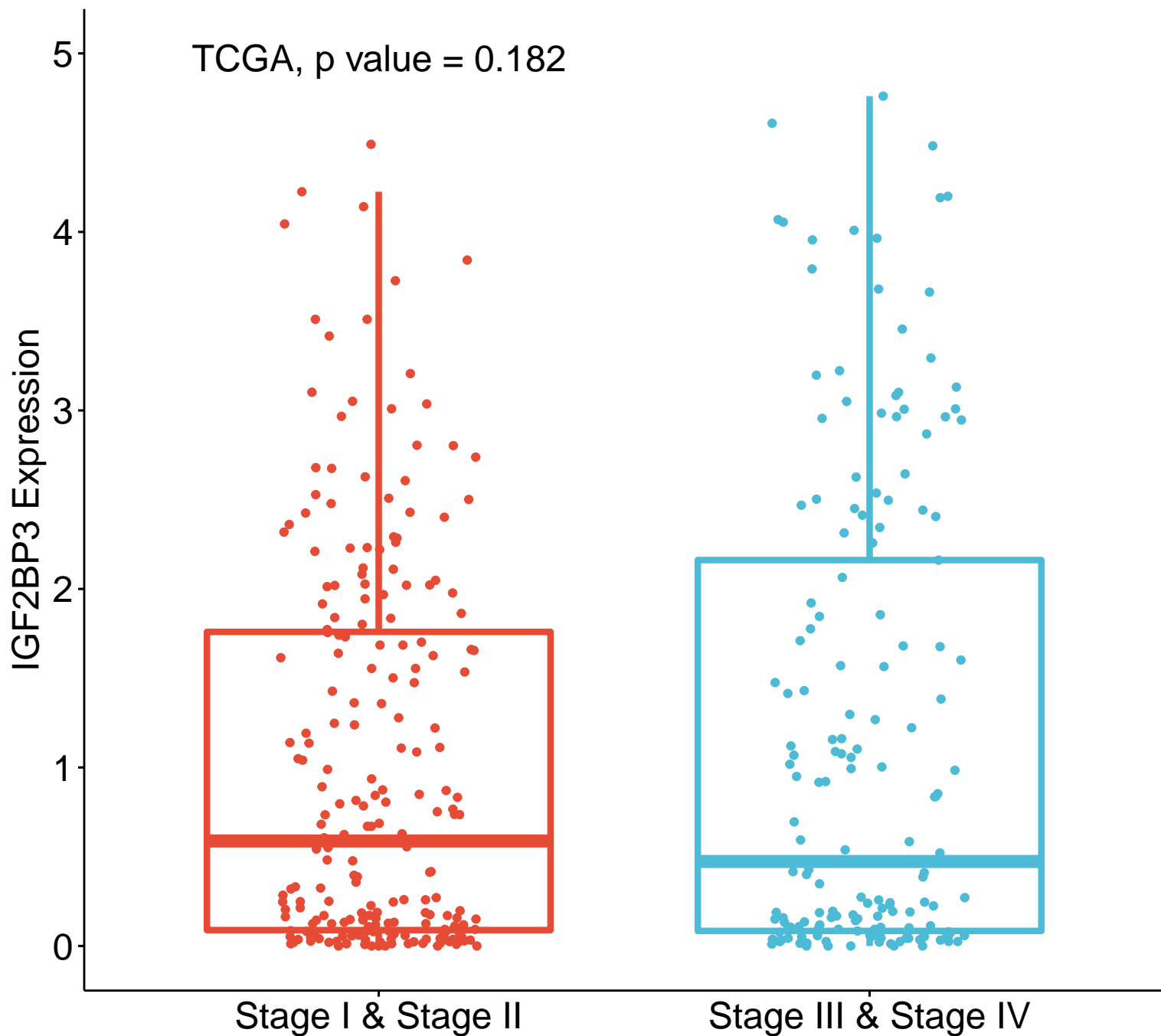

Supplement: Supplementary Materials — See Figures S1 in the Supplementary Material for correlations between expression of IGF2BP3 and clinicopathological features in TCGA database, Figure S2 and Table S4 for the DEGs in groups with different expression of IGF2BP3, Table S1 and Table S2 for the clinical information of the samples, Table S3 for gene coexpressed with IGF2BP3, Table S5 for the genes related to IGF2BP3, and Table S6 for the results of KEGG enrichment analysis. [file 5883101.f1.zip › Figure S1 F.pdf]

T T1-2 T3-4

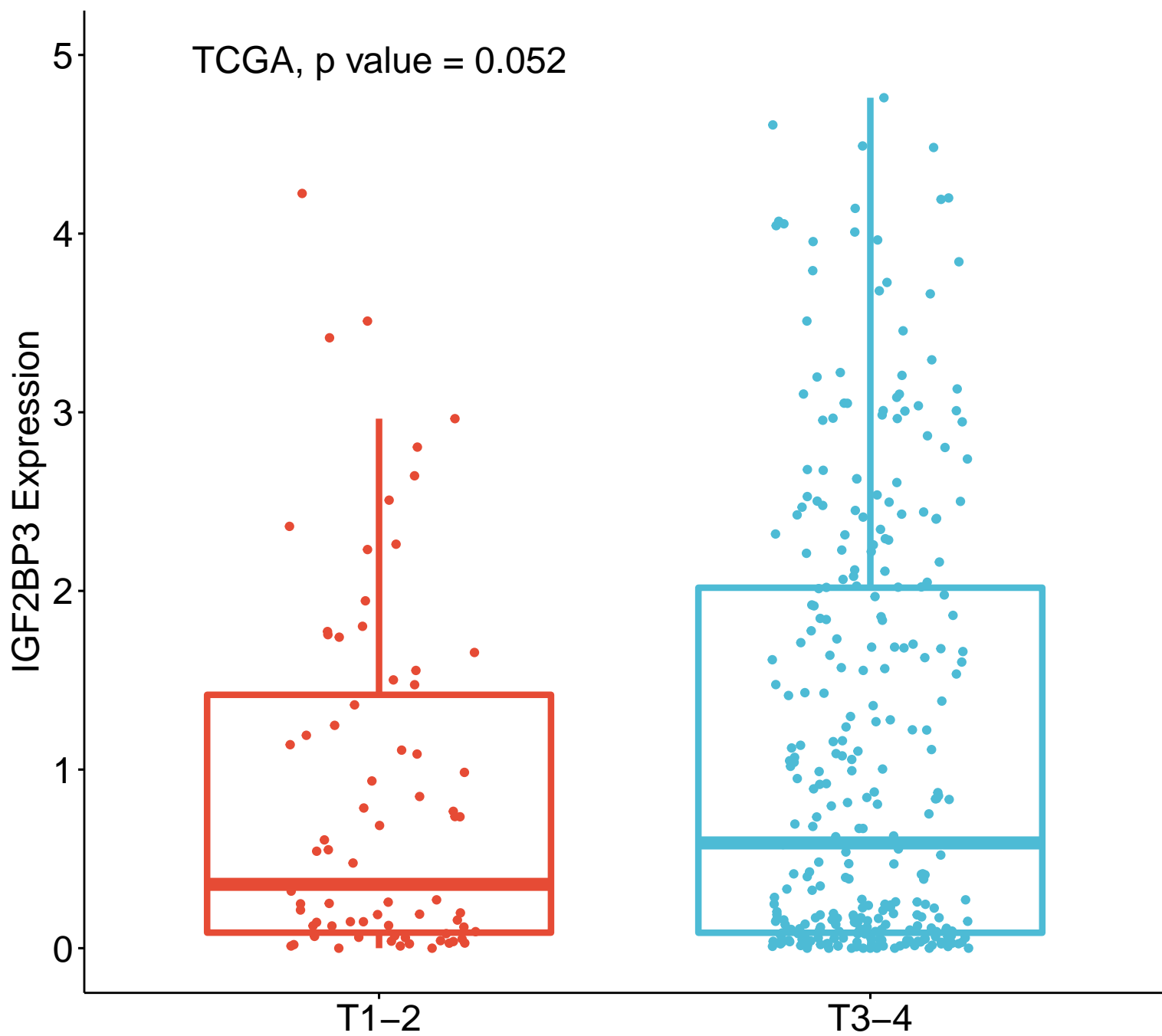

Supplement: Supplementary Materials — See Figures S1 in the Supplementary Material for correlations between expression of IGF2BP3 and clinicopathological features in TCGA database, Figure S2 and Table S4 for the DEGs in groups with different expression of IGF2BP3, Table S1 and Table S2 for the clinical information of the samples, Table S3 for gene coexpressed with IGF2BP3, Table S5 for the genes related to IGF2BP3, and Table S6 for the results of KEGG enrichment analysis. [file 5883101.f1.zip › Figure S1 G.pdf]

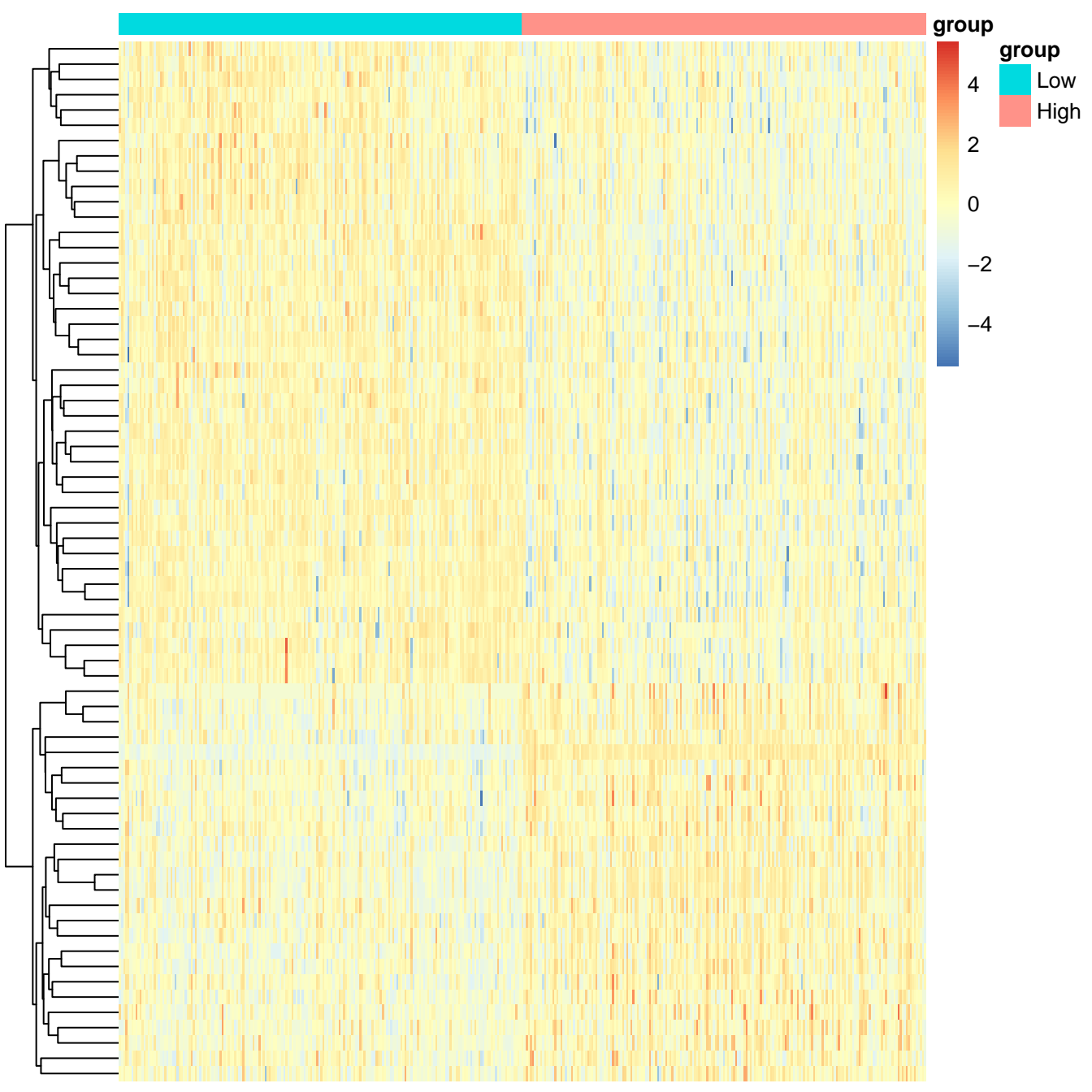

Supplement: Supplementary Materials — See Figures S1 in the Supplementary Material for correlations between expression of IGF2BP3 and clinicopathological features in TCGA database, Figure S2 and Table S4 for the DEGs in groups with different expression of IGF2BP3, Table S1 and Table S2 for the clinical information of the samples, Table S3 for gene coexpressed with IGF2BP3, Table S5 for the genes related to IGF2BP3, and Table S6 for the results of KEGG enrichment analysis. [file 5883101.f1.zip › Figure S2 DE_VennGene_Heatmap.pdf]
